# Synthesis of new tetra- and pentacyclic, methylenedioxy- and ethylenedioxy-substituted derivatives of the dibenzo[*c*,*f*][1,2]thiazepine ring system

**DOI:** 10.3762/bjoc.21.205

**Published:** 2025-12-09

**Authors:** Gábor Berecz, András Dancsó, Mária Tóthné Lauritz, Loránd Kiss, Gyula Simig, Balázs Volk

**Affiliations:** 1 Egis Pharmaceuticals Plc., Directorate of Drug Substance Development, P. O. Box 100, H-1475 Budapest, Hungary; 2 HUN-REN Research Centre for Natural Sciences, Institute of Organic Chemistry, Stereochemistry Research Group, Magyar tudósok krt. 2, H-1117 Budapest, Hungary

**Keywords:** acylation, bridged derivatives, cyclization, Friedel–Crafts reaction, heterocycles, new ring systems

## Abstract

New tetra- and pentacyclic derivatives of the dibenzo[*c*,*f*][1,2]thiazepine ring system have been synthesized. The target compounds contain methylenedioxy or ethylenedioxy substituents linked to the benzene ring. The key step for the construction of the ring systems has been implemented by an intramolecular Friedel–Crafts cyclization. Altogether eight new ring systems are described here, five of them are also characterized by single-crystal X-ray diffraction.

## Introduction

**Figure 1:**
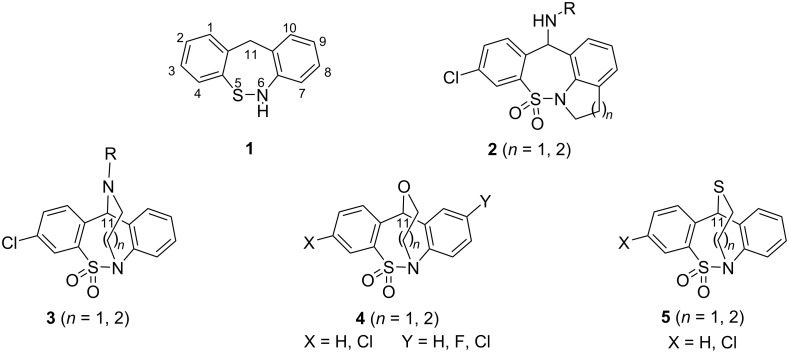
Reported ring systems incorporating the dibenzo[*c*,*f*][1,2]thiazepine (**1**) skeleton.

In our prior report [1] we disclosed the synthesis of new fused ring derivatives of the dibenzo[*c*,*f*][1,2]thiazepine skeleton (**1**, Figure 1) shown by general structure **2**. In recent publications we have also reported the synthesis of new ring systems containing a two or three-carbon linker between the nitrogen atom of the thiazepine ring and the nitrogen (**3**), oxygen (**4**), or sulfur (**5**) atom linked to the C(11) atom [2–3].

**Figure 2:**
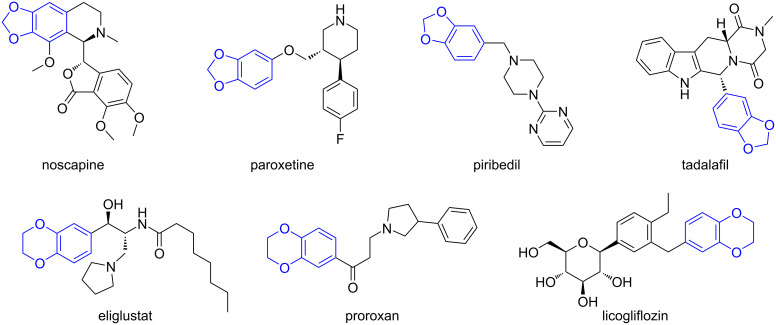
Drugs exhibiting a 1,3-benzodioxole or 1,4-benzodioxane structural unit marketed or in development.

In continuation of our efforts to study new ring systems, we decided to synthesize new tetra- and pentacyclic dibenzo[*c*,*f*][1,2]thiazepine derivatives containing a 1,3-benzodioxole or, with particular emphasis, a 1,4-benzodioxane moiety. The importance of these structural motifs is well demonstrated in medicinal chemistry by marketed drugs and by compounds that have reached the human clinical development stage in various therapeutic fields. Launched drugs possessing a 1,3-benzodioxole element, shown in Figure 2, are among others the cough suppressant benzylisoquinoline alkaloid noscapine [4–5], the antidepressant paroxetine [6–7], antiparkinsonian agent piribedil [8–9], and tadalafil [10–11], for the treatment of male sexual disfunction. A review published in 2020 [12] gives an excellent overview about the medicinal chemistry of compounds incorporating a 1,4-benzodioxane scaffold variously substituted either on the aliphatic or on the aromatic carbon atoms. In connection with our present work, the latter compounds are of interest, e.g., the marketed drugs eliglustat for the treatment of Gaucher’s disease [13–14] and the antihypertensive agent proroxan [15–16]. Licogliflozin is a sodium-glucose transporter 2 inhibitor developed in several indications: obesity [17], polycystic ovary syndrome [18], and non-alcoholic steatohepatitis (NASH) [19].

## Results and Discussion

We first aimed to synthesize a new tetracyclic ring system containing the ethylenedioxy moiety attached to positions 8 and 9 of the dibenzo[*c*,*f*][1,2]thiazepine (**1**) core. Given the structural similarity, some analogous methylenedioxy derivatives have been also prepared.

**Scheme 1:**
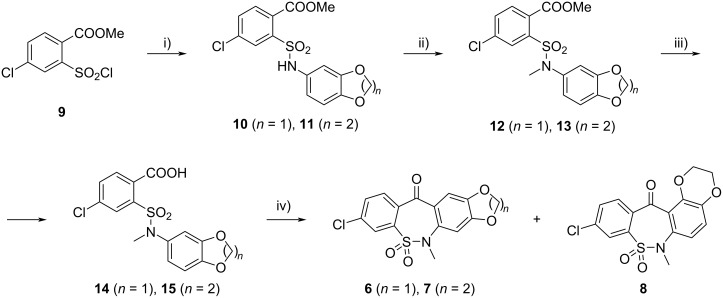
Synthesis of tetracyclic key intermediates **6** and **7**. Conditions: i) 1,3-benzodioxol-5-amine (*n* = 1)/2,3-dihydro-1,4-benzodioxin-6-amine (*n* = 2), PhNEt_2_, MeOH, rt, 1 h, 95%/92%; ii) MeI, K_2_CO_3_, DMF, 4 h/1 h, 97%/98%; iii) NaOH, MeOH/H_2_O, reflux, 1 h, 97%/99%; iv) 1. PCl_5_, DCM, reflux, 8.5 h/9 h; 2. SnCl_4_, 0–5 °C, 2 h/1 h → rt, 2 h; 76%/84% (**8**: 2%).

The synthesis of key intermediates **6** and **7** is presented in Scheme 1. The reaction of sulfonyl chloride **9** with 1,3-benzodioxol-5-amine or 2,3-dihydro-1,4-benzodioxin-6-amine gave sulfonamides **10** or **11**, respectively. *N*-Methylation with methyl iodide via compounds **12** and **13** followed by basic hydrolysis afforded carboxylic acids **14** and **15**. Ring closure of the latter compounds to tetracyclic derivatives **6** and **7** was carried out in one pot by SnCl_4_-catalyzed Friedel–Crafts cyclization of the acid chlorides obtained via reaction of **14** and **15** with phosphorus pentachloride. In the reaction of compound **15**, regioisomer **8** was also isolated in 2% yield, which represents a new ring system, too. It is interesting to mention that the formation of a similar isomer was not observed in the cyclization reaction of compound **14**. The same disparity was observed in the studies of electrophilic substitution reactions of benzo-1,3-dioxoles and benzo-1,4-dioxanes [20].

**Scheme 2:**
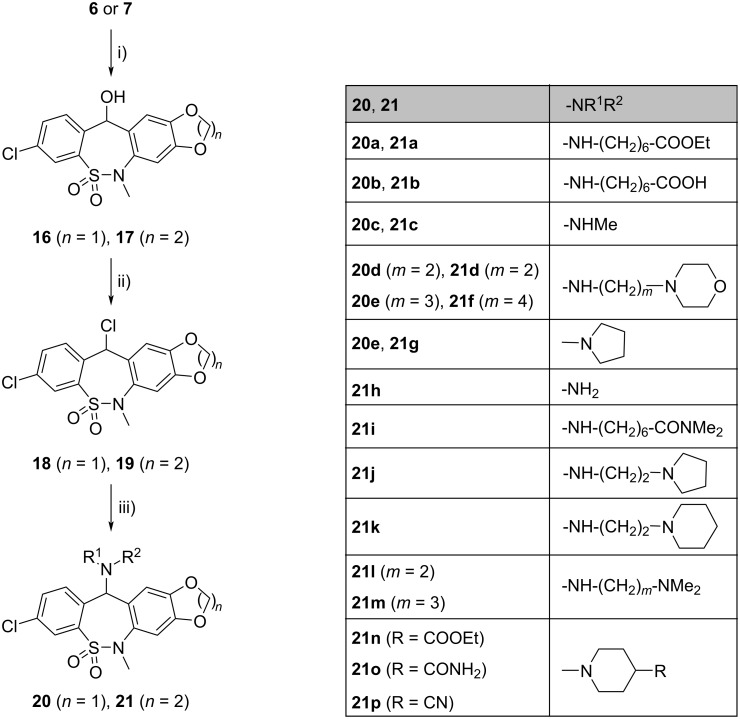
Synthesis of tetracyclic compounds **20** and **21**. Conditions: i) NaBH_4_, DMF, EtOH, rt, 4.5 h/23 h, 95%/98%; ii) SOCl_2_, DCM, rt, 1.5 h/3.5 h, 100%/94%; iii) **20a**,**c**–**e**: HNR^1^R^2^, dioxane or MeCN, rt, 1 h, 56–84%; **21a**,**c**–**p**: HNR^1^R^2^, dioxane or MeCN, rt, 40 min–3.5 h, 48–93%.

**Figure 3:**
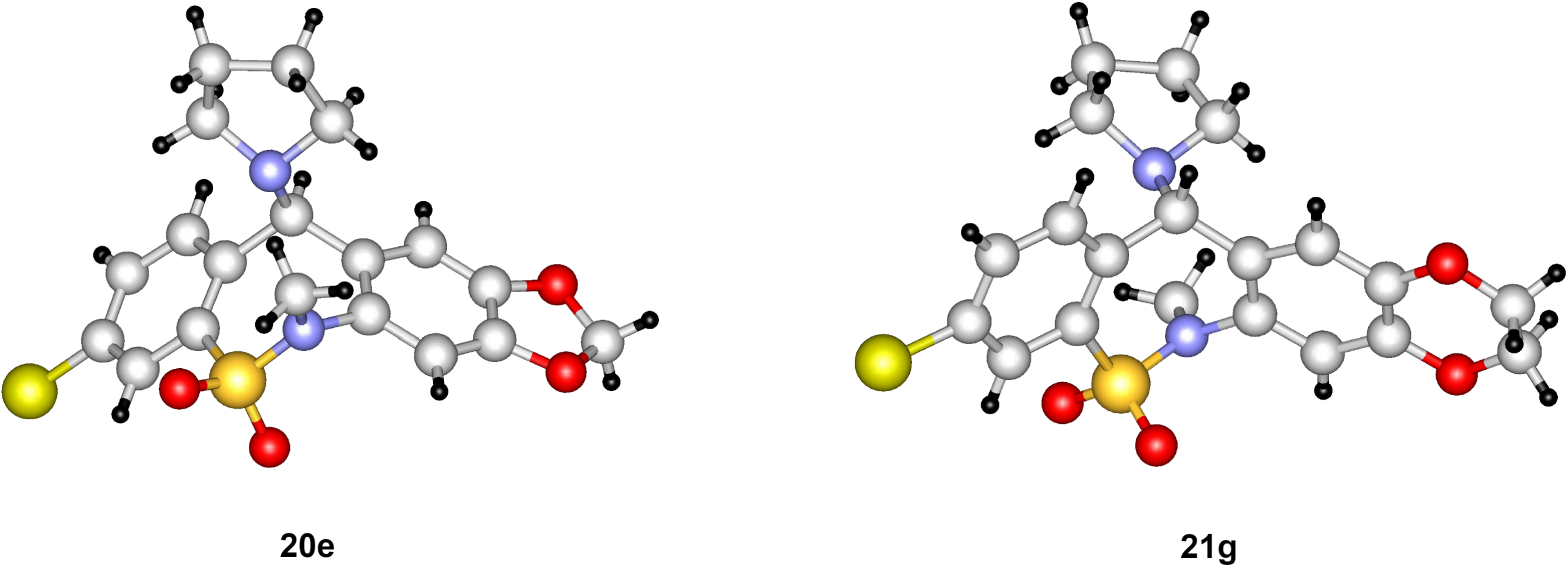
X-ray structures of compounds **20e** and **21g**.

Reduction of ketones **6** and **7** with NaBH_4_ gave alcohols **16** and **17**, which were chlorinated with SOCl_2_ to result in compounds **18** and **19**. Treatment of the latter with the appropriate amines gave amino derivatives **20a**, **20c–e**, and **21a**, **21c–p**, respectively (Scheme 2). The structure of compounds **20e** and **21g** was also confirmed by single-crystal X-ray diffraction (Figure 3).

**Scheme 3:**
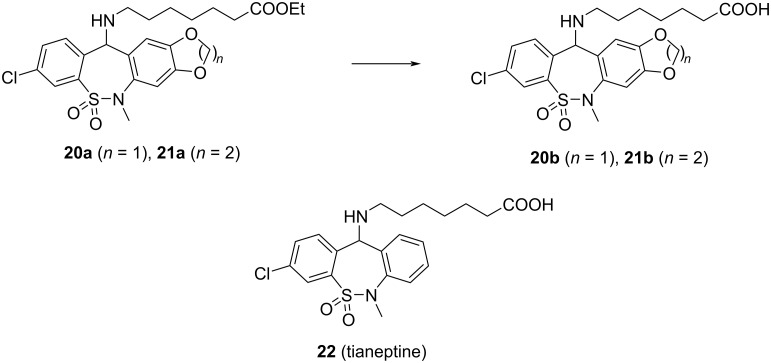
Synthesis of tianeptine analogues **20b** and **21b**. Conditions: **20b**: NaOH, EtOH/H_2_O, rt, 25 h, 80%; **21b**: NaOH, EtOH/H_2_O, rt, 19 h, 73%.

We demonstrated the potential utility of the synthesized new ring systems in the field of medicinal chemistry by the synthesis of tetracyclic analogues **20b** and **21b** of tianeptine (**22**), an atypical antidepressant drug on the market [21–25], by hydrolysis of esters **20a** and **21a** (Scheme 3).

**Scheme 4:**
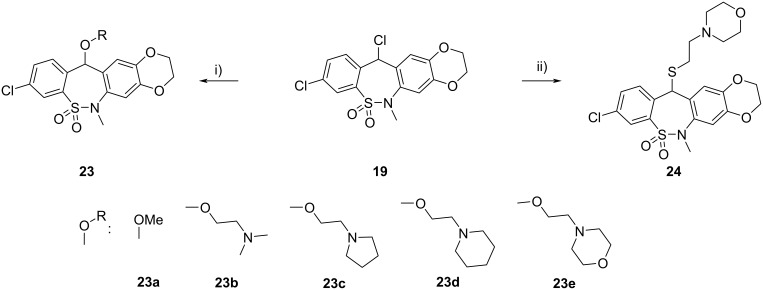
Synthesis of tetracyclic ethers **23** and thioether **24**. Conditions: i) ROH, MeCN, rt, 2–3 h, 38–84%; ii) 2-(morpholin-4-yl)ethanethiol, MeCN, rt, 1 h, 72%.

**Figure 4:**
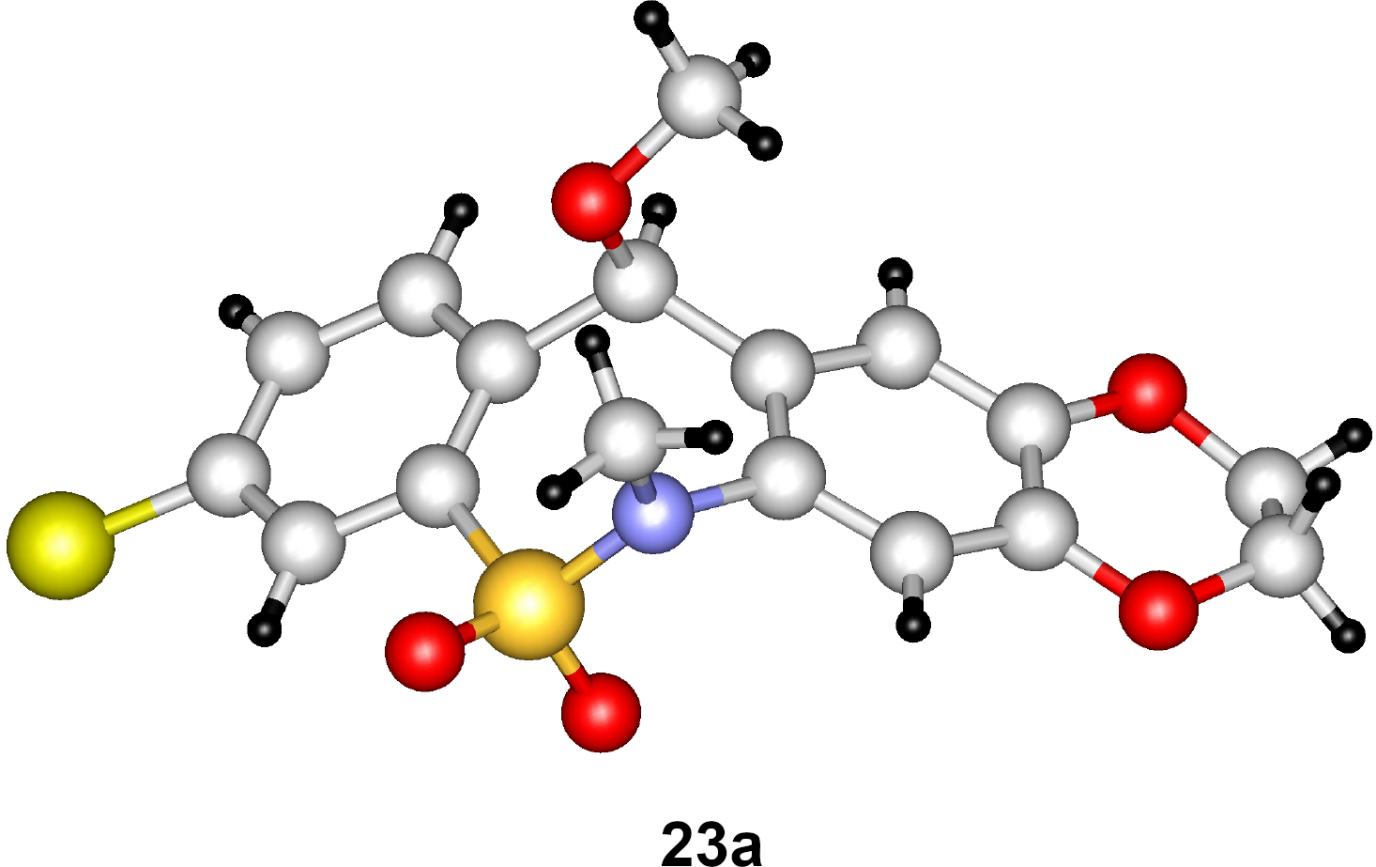
X-ray structure of compound **23a**.

Treatment of chloro derivative **19** with various alcohols afforded ethers **23**, while its reaction with 2-(morpholin-4-yl)ethanethiol resulted in thioether **24** (Scheme 4). The structure of compound **23a** was proven by single-crystal X-ray diffraction (Figure 4).

**Scheme 5:**
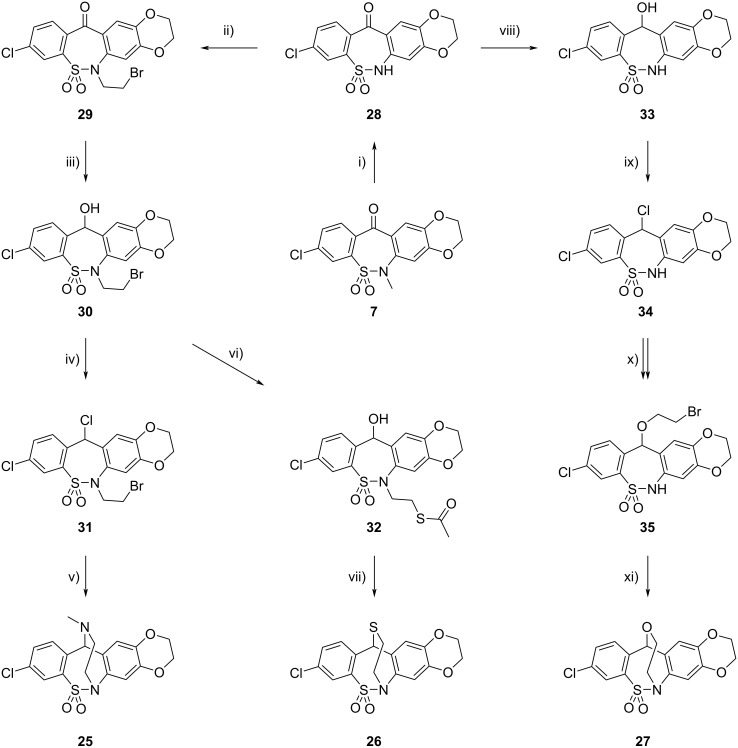
Synthesis of pentacyclic compounds **25**–**27**. Conditions: i) Py·HCl, 180 °C, 27 h, 51%; ii) Br(CH_2_)_2_Br, MeCN, K_2_CO_3_, reflux, 22 h, 74%; iii) NaBH_4_, THF/EtOH, rt, 30 min, 99%; iv) SOCl_2_, DCM, rt, 2.5 h, 90%; v) MeNH_2_, dioxane, 5 °C, 1.5 h, 25 °C, 20 h, 78%; vi) AcSK, MeCN, Bu_4_NBr (cat.), rt, 4 h, 84%; vii) 1. K_2_CO_3_, MeOH; rt, 30 min, 2. PTSA (cat.), DCM, rt, 1 h, 91%; viii) NaBH_4_, NaOH, H_2_O, rt, 69 h, 83%; ix) SOCl_2_, DCM, rt, 2 h, crude: 100%; x) Br(CH_2_)_2_OH, MeCN, rt, 6 h, 61%; xi) K_2_CO_3_, MeCN, rt, 5 h, 71%.

**Figure 5:**
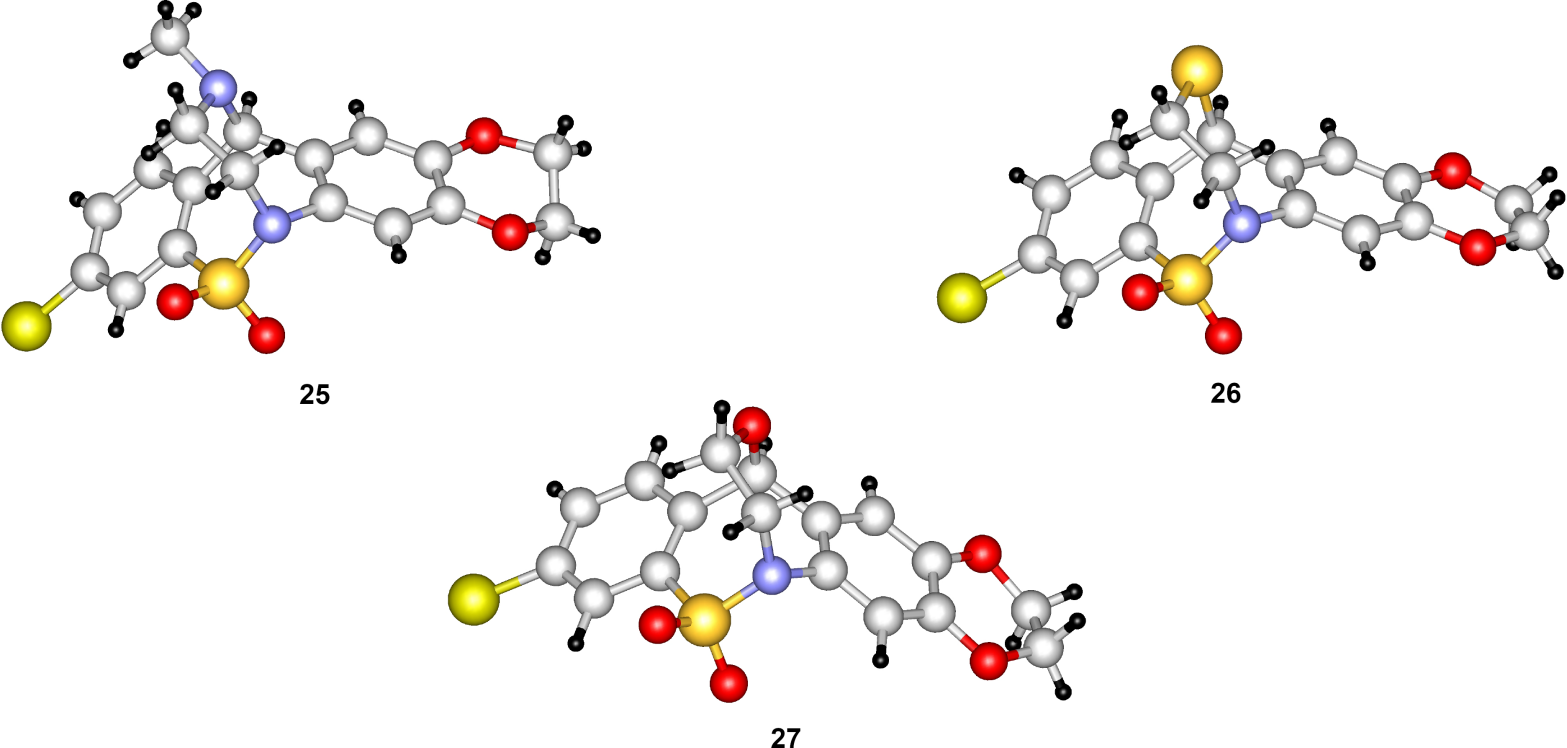
X-ray structures of compounds **25**, **26**, and **27**.

Starting from compound **7**, using methods we have previously developed for the synthesis of related compounds [2–3], we have now prepared new pentacyclic ring systems **25**–**27** (Scheme 5). Demethylation of **7** followed by alkylation of compound **28** with 1,2-dibromoethane (**29**), reduction with NaBH_4_ (**30**), chlorination with SOCl_2_ (**31**) and subsequent treatment with methylamine afforded pentacyclic product **25**. Reaction of bromoalkyl derivative **30** with potassium thioacetate gave acetylthio derivative **32**, which was cyclized after removal of the acetyl group by intramolecular dehydrative thioetherification [3] to the bridged molecule **26**. As regards the synthesis of compound **27**, reduction of ketone **28** with NaBH_4_ (**33**), chlorination with SOCl_2_ (**34**) and treatment with 2-bromoethanol afforded 2-bromoethoxy derivative **35**, which was cyclized to target compound **27** by intramolecular *N*-alkylation reaction. Structures of compounds **25**, **26** and **27** have also been confirmed by single-crystal X-ray diffraction (Figure 5).

**Scheme 6:**
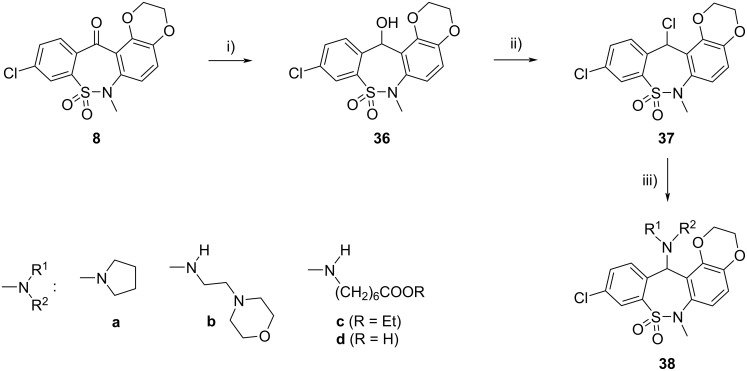
Synthesis of tetracyclic compounds **38**. Conditions: i) NaBH_4_, DMF/EtOH, rt, 3 h, 89%; ii) SOCl_2_, DCM, rt, 1.5 h, 94%; iii) **38a**–**c**: R^1^R^2^NH, MeCN, rt, 1 h, 79%, 78%, 84%; **38d**: **38c**, NaOH, EtOH/H_2_O, rt, 19 h, 82%.

Next, we synthesized some derivatives of new tetracyclic ketone **8** in a way that is already routinely used (Scheme 6): reduction of **8** with NaBH_4_, followed by chlorination of alcohol **36** with SOCl_2_ and substitution of chloro derivative **37** with various amines resulted in compounds **38a**–**c**. Tianeptine analogue **38d** was obtained by hydrolysis of ester **38c**.

**Scheme 7:**
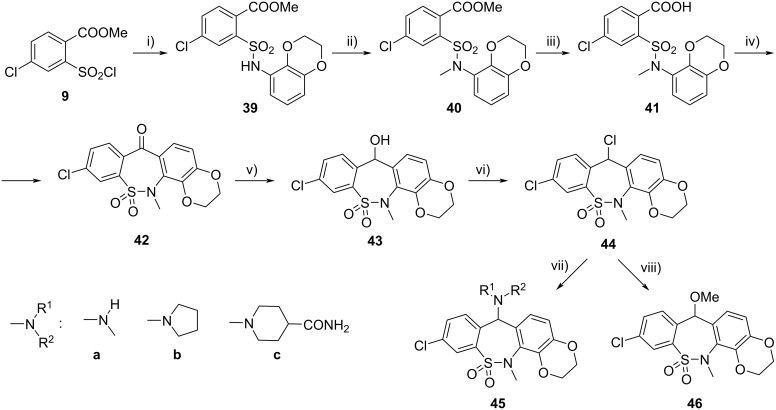
Synthesis of tetracyclic compounds **45** and **46**. Conditions: i) 2,3-dihydro-1,4-benzodioxin-5-amine, PhNEt_2_, MeOH, rt, 1 h, 0–5 °C, 1 h, 82%; ii) MeI, K_2_CO_3_, DMF, rt, 2 h, 87%; iii) NaOH, MeOH/H_2_O, reflux, 1 h, 97%; iv) 1. PCl_5_, DCM, reflux, 9 h; 2. SnCl_4_, 0–5 °C, 15 h, 86%; v) NaBH_4_, DMF, EtOH, rt, 3.5 h, 94%; (vi) SOCl_2_, DCM, rt, 2 h crude: 94%; vii) R^1^R^2^NH, dioxane or MeCN, rt, 1 h, 74–85%; viii) MeOH, DCM, rt, 40 min, 76%.

The synthesis of the new regioisomeric tetracyclic compounds containing the ethylenedioxy moiety attached to positions 7 and 8 of the dibenzo[*c*,*f*][1,2]thiazepine (**2**) core (instead of positions 8 and 9) has also been carried out in the traditional way (Scheme 7). Sulfonamide **39**, obtained in the reaction of sulfonyl chloride **9** with 2,3-dihydro-1,4-benzodioxin-5-amine was transformed via intermediates **40**‒**43** to chloro derivative **44**, which was converted with amines to compounds **45** and with methanol to ether **46**.

**Scheme 8:**
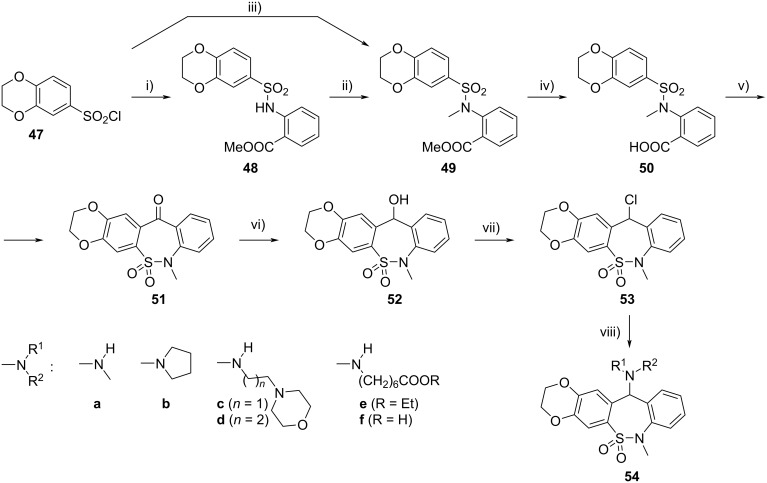
Synthesis of tetracyclic compounds **54**. Conditions: i) methyl anthranilate, pyridine, 0–5 °C, 4 h, rt, 13 h, 95%; ii) method A: MeI, K_2_CO_3_, DMF, rt, 22 h, crude: 99%; iii) method B: methyl *N*-methylanthranilate, pyridine, 0–5 °C, 2 h, rt, 17 h, 71%; iv) NaOH, MeOH/H_2_O, reflux, 1 h, crude: 95%; v) 1. SOCl_2_, DCM, reflux, 8.5 h; 2. AlCl_3_, 0–5 °C, 1.5 h, reflux, 3 h, 26%; vi) NaBH_4_, DMF/EtOH, rt, 2 h, 93%; vii) SOCl_2_, DCM, rt, 2 h, crude: 95%; viii) **54a**–**e**: R^1^R^2^NH, dioxane or MeCN, rt, 1–1.5 h, 74–94%; **54f**: **54e**, NaOH, EtOH/H_2_O, rt, 20 h, 70%.

Finally, we synthesized related tetracyclic derivatives containing the ethylenedioxy moiety attached to positions 2 and 3 of the dibenzo[*c*,*f*][1,2]thiazepine (**1**) core (Scheme 8). While the amidation (**48**), *N*-methylation (**49**), and hydrolysis (**50**), as well as the direct amidation of **47** to **49** worked well, the Friedel–Crafts cyclization via the corresponding acyl chloride to the tetracyclic key intermediate **51** could only be carried out with 26% yield, obviously because of incomplete regioselectivity. Reduction of **51** with NaBH_4_ and subsequent chlorination of alcohol **52** with SOCl_2_ gave chloro derivative **53**, which was treated with amines to afford compounds **54a**–**e**. 7-Aminoheptanoic acid ester derivative **54e** was hydrolyzed to tianeptine analogue **54f**.

## Conclusion

As continuation of our efforts to develop new ring systems that include the characteristic dibenzo[*c*,*f*][1,2]thiazepine tricyclic core, we have synthesized new tetra- and pentacyclic derivatives containing methylenedioxy or ethylenedioxy pharmacophores. The key step of the synthesis of tetracyclic compounds is an intramolecular Friedel–Crafts cyclization. The pentacyclic derivatives were obtained by an ethylene bridging between two heteroatoms of the tetracyclic compounds.

## Experimental

Compounds **9** and **47** are commercially available. Compounds **6**–**8**, **10**–**21**, **23**–**46**, and **48**–**54** are new and they are characterized either below or in Supporting Information File 1 in detail. In cases where a small sample of the material was recrystallized, the solvent of recrystallization is given after the melting point in parentheses. All melting points were determined on a Kofler hot-stage microscope Boëtius PHMK 05 melting point apparatus and are uncorrected. The amorphous character of the compounds exhibiting wide melting ranges (**21b**, **38d**, **54f**) were proven by XRPD measurements performed on a PANalytical Empyrean X-ray powder diffractometer at room temperature. Powder samples (without grinding) were placed between two Mylar foils in the sample holder at a reflection–transmission spinner stage (1 rps sample rotation speed) and measured in transmission mode using Cu Kα (1.541874 Å) radiation in the 2.0000–34.9964° 2θ range with 0.0131° 2θ step size and continuous gonio (θ/θ) scan in 1 measurement cycle. 45 kV accelerating voltage and 40 mA anode heating current were used. Diffraction intensity was measured by a PIXcel 3D 1 × 1 area detector. Thermogravimetric (TG) curves of amorphous samples (**21b**, **38d**, **54f**) were measured by a TA Instruments Discovery TGA thermogravimetric analyzer in the 30–200 °C temperature range with a 10 °C/min heating rate using 11 to 12 mg sample in a 100 μL platinum pan in 25 mL/min nitrogen atmosphere. Melting properties of the amorphous samples (**21b**, **38d**, **54f**) were also studied by a TA Instruments Discovery DSC differential scanning calorimeter in the 25–120 °C temperature range with a 10 °C/min heating rate using 2.5 to 3.0 mg sample in a standard open 40 μL aluminum pan under 50 mL/min nitrogen atmosphere. IR spectra were obtained on a Bruker ALPHA FT-IR spectrometer in KBr pellets or in neat. NMR spectra were recorded at 295 K on a Bruker Avance III HD 600 (600 and 150 MHz for ^1^H and ^13^C NMR spectra, respectively) spectrometer equipped with a Prodigy cryo-probehead or a Varian Unity Inova 500 (500 and 125 MHz for ^1^H and ^13^C NMR spectra, respectively), or a Varian Unity Inova 400 (400 and 100 MHz for ^1^H and ^13^C NMR spectra, respectively), or a Varian Gemini 200 (200 and 50 MHz for ^1^H and ^13^C NMR spectra, respectively) spectrometer. The pulse programs were taken from the Bruker software library (TopSpin 3.5) and full ^1^H and ^13^C assignments were achieved with widely accepted strategies. ^1^H assignments were accomplished using general knowledge of chemical shift dispersion with the aid of the ^1^H–^1^H coupling pattern. CDCl_3_ or DMSO-*d*_6_ was used as the solvent and tetramethylsilane (TMS) as the internal standard. Chemical shifts (δ) and coupling constants (*J*) are given in ppm and in Hz, respectively. Coupling constants reported in the ^1^H NMR spectra are H–H couplings unless otherwise stated (*J* ≡ *J*_H–H_). Three-bond H–H couplings (^3^*J*) are aromatic *ortho* or aliphatic vicinal ones, four-bond ones (^4^*J*) are aromatic *meta* couplings. For the sake of brevity, we use simplified notations unless otherwise stated: COSY for ^1^H–^1^H 2D COSY, NOESY for ^1^H–^1^H 2D NOESY, HSQC for ^1^H–^13^C 2D HSQC, HMQC for ^1^H–^13^C 2D HMQC, and HMBC for ^1^H–^13^C 2D HMBC. High-resolution mass spectra (HRMS) were recorded on a Bruker Q-TOF MAXIS Impact mass spectrometer coupled with a Waters I-Class UPLC system with a diode array detector. HRMS (TOF MS EI) measurements were performed on an Agilent 2750 GC/Q-TOF mass spectrometer with MSD direct inlet probe. Single-crystal X-ray diffraction (SC-XRD) measurements were carried out on a Rigaku R-Axis Spider diffractometer with an imaging plate area detector using graphite monochromatic Cu Kα radiation. Single crystal X-ray structures were deposited at the Cambridge Crystallographic Data Centre under the following numbers: CCDC 2470493 (**20e**), CCDC 2470491 (**21g**), CCDC 2470492 (**23a**), CCDC 2470490 (**25**), CCDC 2470489 (**26**), CCDC 2472402 (**27**). All reagents were purchased from commercial sources and were used without further purification. Reactions were followed by analytical thin-layer chromatography on silica gel 60 F_254_ (Merck 105554). Dry-column flash chromatographic purifications were performed on silica gel 60 H (Merck 107736), silica gel 60 (Merck 115111) or aluminum oxide 60 G neutral (Merck 101090) column [26].

**9-Chloro-6-methyl-2,3-dihydro[1,4]benzodioxino[6,7-*c*]benzo[*f*][1,2]thiazepin-12(6*H*)-one 7,7-dioxide (7) and 10-chloro-7-methyl-2,3-dihydro[1,4]benzodioxino[6,5-*c*]benzo[*f*][1,2]thiazepin-13(7*H*)-one 8,8-dioxide (8):** To the suspension of **15** (31.66 g, 82.5 mmol) in DCM (320 mL), PCl_5_ (20.82 g, 100 mmol) was added in two portions and the solution obtained was refluxed for 9 h. The solution was cooled to 0–5 °C, and SnCl_4_ (47.28 g, 182 mmol, 21.2 mL) was added to the reaction mixture. The red suspension obtained was stirred at 0–5 °C for 1 h, then at room temperature for 2 h. It was poured onto a mixture of crushed ice (1500 g) and conc. hydrochloric acid (200 mL). It was stirred while warming to room temperature (ca 3 h). The crystalline product separated was filtered, washed with diluted hydrochloric acid (5%, 2 × 50 mL) and water (2 × 50 mL), then air-dried to give crude **7** (17.6 g of off-white solid). The two-phase filtrate was separated, the strongly acidic aqueous phase was washed with DCM (100 mL), the combined organic phases were dried over Na_2_SO_4_, evaporated, and the oily residue was triturated with a little amount of acetone to afford a second crop of crude **7** (8.45 g of pale yellow solid). The two crops were purified by dry-column flash chromatography on a short silica gel column (thickness of the stationary phase: 30 mm, eluent: DCM). After evaporation of the solvent, the residue was triturated with Et_2_O (50 mL) to give pure **7** (25.30 g, 84%) as colorless crystals. Mp 223‒225 °C (CH_3_CN/EtOH 1:1 (v/v)); IR (KBr): 1638, 1604, 1574, 1501, 1354, 1300, 1175, 1063, 854 cm^−1^; ^1^H NMR (600 MHz, CDCl_3_): 7.99 (d, ^3^*J* = 8.4 Hz, 1H), 7.95 (d, ^4^*J* = 2.1 Hz, 1H), 7.86 (s, 1H), 7.66 (dd, ^4^*J* = 2.1 Hz, ^3^*J* = 8.3 Hz, 1H), 6.83 (s, 1H), 4.35 (m, 2H), 4.30 (m, 2H), 3.24 (s, 3H); ^13^C NMR (150 MHz, CDCl_3_): 187.20, 149.26, 142.28, 138.57, 138.06, 135.76, 134.05, 133.80, 133.09, 125.83, 125.27, 120.48, 113.73, 64.95, 64.05, 39.39; COSY: 7.99‒7.66‒7.95, 4.35‒4.30; HSQC (140 Hz): 7.99‒133.80, 7.95‒125.83, 7.86‒120.48, 7.66‒133.09, 6.83‒113.73, 4.35‒64.95, 4.30‒64.05, 3.24‒39.39; HMBC (8 Hz, 140 Hz): 7.99‒(187.20, 138.57, 138.06), 7.95‒(138.57, 134.05, 133.09), 7.86‒(187.20, 149.26, 135.76), 7.66‒(138.57, 134.05, 125.83), 6.83‒(142.28, 135.76, 125.27), 4.35‒149.26, 4.30‒142.28, 3.24‒135.76; EIMS (*m*/*z*): [M]^+^ 365 (1 Cl); HRESIMS (*m*/*z*): [M + H]^+^ calcd. for C_16_H_13_ClNO_5_S, 366.0197; found, 366.0199.

The mother liquors of compound **7** were evaporated in vacuo and the residue was subjected to dry-column flash chromatography (short aluminum oxide column, eluent: heptane/DCM 1:1, DCM) and isomer **8** (0.62 g, 2%) was isolated as pale yellow crystals. Mp 254‒256 °C (CH_3_CN); IR (KBr): 1665, 1485, 1344, 1111, 1061, 859 cm^−1^; ^1^H NMR (500 MHz, CDCl_3_): 7.96 (d, ^4^*J* = 2.0 Hz, 1H), 7.94 (d, ^3^*J* = 8.6 Hz, 1H), 7.63 (dd, ^4^*J* = 2.1 Hz, ^3^*J* = 8.5 Hz, 1H), 7.03 (d, ^3^*J* = 8.7 Hz, 1H), 6.92 (d, ^3^*J* = 8.7 Hz, 1H), 4.30 (m, 2H), 4.28 (m, 2H), 3.28 (s, 3H); ^13^C NMR (125 MHz, CDCl_3_): 189.67, 144.54, 141.91, 140.51, 140.19, 132.98, 132.52, 132.24, 129.40, 129.23, 127.41, 121.61, 121.00, 64.39, 63.90, 39.93; NOE: 3.28‒(7.96, 6.92); HSQC (140 Hz): 7.96‒127.41, 7.94‒132.24, 7.63‒132.52, 7.03‒121.00, 6.92‒121.61, 4.30‒63.90, 4.28‒64.39, 3.28‒39.93; HMQC (140 Hz, 8 Hz): 7.96‒(140.19, 132.52), 7.94‒(140.51, 132.98), 7.63‒(140.19, 132.98, 127.41), 7.03‒(141.91, 129.40), 6.92‒(189.67, 144.54, 129.23), 4.30‒144.54, 4.28‒141.91, 3.28‒129.40; EIMS (*m*/*z*): [M]^+^ 365 (1 Cl); HRESIMS (*m*/*z*): [M + H]^+^ calcd. for C_16_H_13_ClNO_5_S, 366.0197; found, 366.0195.

**Ethyl 7-[(9-chloro-6-methyl-7,7-dioxido-2,3,6,12-tetrahydro[1,4]benzodioxino[6,7-*c*]benzo[*f*][1,2]thiazepin-12-yl)amino]heptanoate (21a):** To a solution of ethyl 7-aminoheptanoate (2.29 g, 13.2 mmol) in CH_3_CN (15 mL) crude **19** (2.32 g, 6 mmol) was added, the solution obtained was stirred at room temperature for 40 min. It was evaporated in vacuo, the residue was dissolved in DCM (20 mL), washed with water (2 × 15 mL), dried over Na_2_SO_4_, and evaporated in vacuo. The oily residue was purified by dry-column flash chromatography on a short aluminum oxide column (thickness of stationary phase: 30 mm, eluent: heptane/DCM 1:1, DCM, DCM/MeOH 100:2). After evaporation of solvents in vacuo **21a** (2.87 g, 91%) was obtained as pale yellow oil, which slowly solidified upon standing at ambient temperature. The melt-like hard substance obtained was suspended in DIPE to afford **21a** as colorless crystals. Mp 76‒78 °C; IR (KBr): 3314, 2939, 1728, 1510, 1328, 1064, 853, 599 cm^−1^; ^1^H NMR (600 MHz, CDCl_3_): 7.94 (d, ^4^*J* = 1.9 Hz, 1H), 7.43 (dd, ^4^*J* = 2.1 Hz, ^3^*J* = 8.4 Hz, 1H), 7.39 (d, ^3^*J* = 8.4 Hz, 1H), 6.92 (s, 1H), 6.83 (s, 1H), 4.85 (s, 1H), 4.24 (s, 4H), 4.11 (q, ^3^*J* = 7.1 Hz, 2H), 3.35 (s, 3H), 2.46 (t, ^3^*J* = 7.0 Hz, 2H), 2.27 (t, ^3^*J* = 7.5 Hz, 2H), 1.83 (br s, 1H), 1.60 (m, 2H), 1.47 (m, 2H), 1.30 (m, 2H), 1.29 (m, 2H), 1.25 (t, ^3^*J* = 7.1 Hz, 3H); ^13^C NMR (150 MHz, CDCl_3_): 173.73, 143.63, 143.29, 140.79, 137.13, 134.12, 132.70, 131.96, 131.11, 130.73, 128.39, 117.57, 117.00, 65.35, 64.31, 64.23, 60.17, 48.12, 38.68, 34.20, 29.87, 28.95, 26.92, 24.79, 14.22; COSY: 7.94‒7.43‒7.39, 4.11‒1.25, 2.46‒1.47‒1.30‒1.29‒1.60‒2.27; HSQC (140 Hz): 7.94‒128.39, 7.43‒131.96, 7.39‒130.73, 6.92‒117.00, 6.83‒117.57, 4.85‒65.35, 4.24‒64.31, 4.24‒64.23, 4.11‒60.17, 3.35‒38.68, 2.46‒48.12, 2.27‒34.20, 1.60‒24.79, 1.47‒29.87, 1.30‒26.92, 1.29‒28.95, 1.25‒14.22; HMBC (8 Hz, 140 Hz): 7.94‒(137.13, 134.12, 131.96), 7.43‒(137.13, 134.12, 128.39), 7.39‒(140.79, 134.12, 65.35), 6.92‒(143.29, 132.70), 6.83‒(143.63, 131.11, 65.35), 4.85‒(140.79, 137.13, 132.70, 131.11, 130.73, 111.57, 48.12), 4.24‒(143.63, 64.23), 4.24‒(173.73, 14.22), 3.35‒131.11, 2.46‒(65.35, 29.89, 26.92), 2.27‒(173.73, 28.95, 24.79), 1.60‒(173.73, 34.20, 28.95, 26.92), 1.47‒(48.12, 28.95, 26.92), 1.30‒(29.87, 28.95), 1.29‒26.92, 1.25‒60.17; ESIMS (*m*/*z*): [M + H]^+^ 523 (1 Cl); HRESIMS (*m*/*z*): [M + H]^+^ calcd for C_25_H_32_ClN_2_O_6_S, 523.1664; found, 523.1668.

**7-[(9-Chloro-6-methyl-7,7-dioxido-2,3,6,12-tetrahydro[1,4]benzodioxino[6,7-*c*]benzo[*f*][1,2]thiazepin-12-yl)amino]heptanoic acid (21b):** To a solution of ester **21a** (2.615 g, 5 mmol) in EtOH (30 mL), NaOH (0.240 g, 6 mmol) dissolved in water (7.5 mL) was added and the solution was stirred at room temperature for 19 h. Water (15 mL) was added to the yellow solution and the ethanol was removed by evaporation in vacuo*.* Water (10 mL) was added and the solution was neutralized with aqueous HCl solution (0.50 mL of conc. HCl dissolved in 10 mL of water, containing 6 mmol of HCl). It was stirred at room temperature for 5 h, the solid product obtained was filtered, washed with water (2 × 3 mL), and air-dried. It was dissolved in acetone (20 mL), the solution was treated with charcoal (0.20 g), and evaporated in vacuo*.* The residue obtained was triturated with water (10 mL) and dried in vacuo to afford **21b** (1.81 g, 73%) as a colorless amorphous solid. Melting range (glass-transition range): 84–92 °C (Kofler–Boëtius, 2 °C/min), 72–84 °C (DSC, 10 °C/min); IR (KBr): 3421, 2932, 1710, 1508, 1321, 1066 cm^−1^. ^1^H NMR (500 MHz, CDCl_3_): 7.94 (s, 1H), 7.46 (m, 2H), 6.90 (s, 1H), 6.89 (s, 1H), 6.71 (br s, 2H), 5.05 (s, 1H), 4.23 (m, 4H), 3.27 (s, 3H), 2.49 (m, 1H), 2.42 (m, 1H), 2.24 (t, ^3^*J* = 7.3 Hz, 2H), 1.56 (m, 2H), 1.47 (m, 2H), 1.27 (m, 4H); ^13^C NMR (125 MHz, CDCl_3_): 178.33, 144.09, 143.09, 140.53, 134.88, 134.72, 132.30, 132.18, 131.77, 129.17, 128.29, 118.79, 116.39, 65.14, 64.27, 64.25, 47.30, 38.74, 34.69, 28.92, 28.87, 26.79, 24.89 ; ESIMS (*m*/*z*): [M + H]^+^ 495 (1 Cl); HRESIMS (*m*/*z*): [M + H]^+^ calcd for C_23_H_28_ClN_2_O_6_S, 495.1351; found, 495.1352.

**6-(2-Bromoethyl)-9-chloro-2,3-dihydro[1,4]benzodioxino[6,7-*c*]benzo[*f*][1,2]thiazepin-12(6*H*)-one 7,7-dioxide (29):** To a suspension of **28** (9.85 g, 28 mmol) in CH_3_CN (140 mL), K_2_CO_3_ (11.61 g, 84 mmol, 3 equiv) was added and the yellow suspension was stirred at room temperature for 30 min. 1,2-Dibromoethane (26.3 g, 140 mmol, 12.1 mL, 5 equiv) was added, and the reaction mixture was refluxed for 22 h. The suspension was cooled to room temperature, the insoluble part was filtered, washed with CH_3_CN (2 × 30 mL). After evaporation of the solvent in vacuo, the oily residue was subjected to a dry-column flash chromatography on a short silica gel column (thickness of stationary phase: 40 mm, eluent: heptane, heptane/DCM 1:1, DCM). After evaporation of the solvents, the residue was triturated with Et_2_O to give **29** (9.56 g, 74%) as colorless crystals. Mp dec from 177 °C (CH_3_CN); IR (KBr): 1498, 1362, 1295, 1178, 1071, 580 cm^−1^; ^1^H NMR (500 MHz, CDCl_3_): 8.07 (d, ^3^*J* = 8.4 Hz, 1H), 7.98 (d, ^4^*J* = 2.1 Hz, 1H), 7.85 (s, 1H), 7.67 (dd, ^4^*J* = 2.2 Hz, ^3^*J* = 8.4 Hz, 1H), 6.87 (s, 1H), 4.35 (m, 2H), 4.30 (m, 2H), 4.00 (t, ^3^*J* = 7.1 Hz, 2H), 3.37 (t, ^3^*J* = 7.0 Hz, 2H); ^13^C NMR (125 MHz, CDCl_3_): 186.42, 149.13, 142.84, 139.67, 138.95, 134.24, 133.51, 133.18, 132.94, 126.81, 125.80, 121.08, 113.85, 64.95, 64.05, 53.17, 27.90; EIMS (*m*/*z*): [M]^+^ 457 (1 Br, 1 Cl); HRESIMS (*m*/*z*): [M + H]^+^calcd for C_17_H_14_BrClNO_5_S, 457.9459; found, 457.9459.

**9-Chloro-14-methyl-2,3-dihydro-12*H*-12,6-(epiminoethano)[1,4]benzodioxino[6,7-*c*]benzo[*f*][1,2]thiazepine 7,7-dioxide (25):** To a stirred and cooled solution of MeNH_2_ (1.96 g, 63 mmol, 15 equiv) in dioxane (17.5 g), **31** (2.01 g, 4.2 mmol) was added at 5 °C and the thin suspension obtained was stirred at 5 °C for 1.5 h and at 25 °C for 20 h. After evaporation of the solvent in vacuo, the solid residue was dissolved in DCM (60 mL) and water (30 mL). The phases were separated. The organic phase was washed with water (30 mL), dried over Na_2_SO_4_, and evaporated in vacuo*.* The residue was purified by dry-column flash chromatography on a short silica gel column (thickness of stationary phase: 30 mm, eluent: heptane/DCM 1:1, 1:2, DCM, DCM/MeOH 100:1). After evaporation of the solvents, the residue was triturated with CH_3_CN to afford **25** (1.29 g, 78%) as colorless crystals. Mp dec from 221 °C (CH_3_CN); IR (KBr): 1506, 1335, 1305, 1163, 1064 cm^−1^; ^1^H NMR (400 MHz, CDCl_3_): 7.88 (d, ^4^*J* = 2.2 Hz, 1H), 7.44 (dd, ^4^*J* = 2.2 Hz, ^3^*J* = 8.2 Hz, 1H), 7.23 (d, ^3^*J* = 8.3 Hz, 1H), 6.98 (s, 1H), 6.76 (s, 1H), 4.50 (s, 1H), 4.20 (m, 4H), 4.03 (m, 1H), 3.61 (m, 1H), 3.23 (m, 1H), 2.62 (m, 1H), 2.42 (s, 3H); ^13^C NMR (100 MHz, CDCl_3_): 145.36, 143.81, 143.56, 135.28, 134.84, 133.58, 131.82, 131.46, 130.25, 127.65, 119.37, 117.75, 73.33, 64.27, 64.12, 49.64, 49.42, 43.49; COSY: 7.88‒7.44‒7.23, (4.03, 3.61)‒(3.23, 2.62); NOESY (0.3 s): 7.23‒(7.44, 4.50), 6.76‒4.50; HSQC (145 Hz): 7.88‒127.65, 7.44‒131.82, 7.23‒133.58, 6.98‒119.37, 6.76‒117.75, 4.50‒73.33, 4.20‒(64.27, 64.12), 4.03‒49.66, 3.61‒49.66, 3.23‒49.42, 2.62‒49.42, 2.42‒43.49; HMBC (145 Hz, 8 Hz): 7.88‒(135.28, 131.82, 130.25), 7.44‒(135.28, 133.58, 130.25, 127.65), 7.23‒(145.36, 135.28, 131.82, 130.25, 127.65, 73.33), 6.98‒(143.81, 134.84, 131.46), 6.76‒(143.81, 143.56, 73.33), 4.50‒(145.36, 134.84, 133.58, 131.46, 130.25, 117.75, 49.42, 43.49), 4.20‒(143.81, 143.56, 64.27, 64.12), 4.03‒131.46, 3.61‒(145.36, 131.46, 49.42), 3.23‒(73.33, 49.66, 43.49), 2.62‒(73.33, 43.49), 2.42‒(73.33, 49.42); NOE (500 MHz, 2 s): 7.88‒(7.44, 7.23, 6.98, 6.76, 4.50, 4.02, 3.23), 7.23‒(7.44, 4.50, 3.23, 2.42); ESIMS (*m*/*z*): [M + H]^+^ 393 (1 Cl); HRESIMS (*m*/*z*): [M + H]^+^ calcd for C_18_H_18_ClN_2_O_4_S, 393.0670; found, 393.0675.

## Supporting Information

File 1 Experimental procedures and characterization of compounds **6**, **10**–**19**, **20a**–**e**, **21c**–**p**, **23a**–**e**, **24**, **26**–**28**, **30**–**37**, **38a**–**d**, **39**–**44**, **45a**–**c**, **46**–**53**, **54a**–**f**. ^1^H and ^13^C NMR spectra of all new compounds. 2D NMR spectra of compounds **6**–**8**, **12**, **21a**, **21b**, **25**–**27**, **30**, **32**, **38b**, **42**, **50**, **51**, **54e**.

File 2 Crystallographic information files, checkcif and structure report files for compounds **20e**, **21g**, **23a**, **25**–**27**.

## Data Availability

All data that supports the findings of this study is available in the published article and/or the supporting information of this article.
