# Supplementary material for: Synthesis of new tetra- and pentacyclic, methylenedioxy- and ethylenedioxy-substituted derivatives of the dibenzo[c,f][1,2]thiazepine ring system
Source: Beilstein J Org Chem. 2025 Dec 9;21:2645–56. doi: 10.3762/bjoc.21.205 (PMC12706374; doi:10.3762/bjoc.21.205)

BEILSTEIN JOURNAL OF ORGANIC CHEMISTRY

Supporting Information

for

Synthesis of new tetra- and pentacyclic, methylenedioxy- and ethylenedioxy-substituted derivatives of the dibenzo[*c*,*f*][1,2]thiazepine ring system

Gábor Berecz, András Dancsó, Mária Tóthné Lauritz, Loránd Kiss, Gyula Simig and Balázs Volk

Beilstein J. Org. Chem. **2025,** *21,* 2645–2656. doi:10.3762/bjoc.21.205

Experimental procedures and characterization of compounds 6, 10–19, 20a–e, 21c–p, 23a–e, 24, 26–28, 30–37, 38a–d, 39–44, 45a–c, 46–53, 54a–f. ^{1}H and ^{13}C NMR spectra of all new compounds. 2D NMR spectra of compounds 6–8, 12, 21a, 21b, 25–27, 30, 32, 38b, 42, 50, 51, 54e

License and Terms: This is a supporting information file under the terms of the Creative Commons Attribution License (https://creativecommons.org/licenses/by/4.0). Please note that the reuse, redistribution and reproduction in particular requires that the author(s) and source are credited and that individual graphics may be subject to special legal provisions.
The license is subject to the *Beilstein Journal of Organic Chemistry* terms and conditions: (https://www.beilstein-journals.org/bjoc/terms)

Table of contents

Experimental procedures and characterization of compounds **6**, **10–19**, **20a–e**, **21c–p**, **23a–e**, **24**, **26–28**, **30–37**, **38a–d**, **39−44**, **45a–c**, **46**, **48−53**, **54a–f** S2

^{1}H and ^{13}C NMR spectra of compounds **6–8**, **10–19**, **20a−e**, **21a−p**, **23a−e**, **24–37**, **38a−d**, **39−43**, **45a−c**, **46**, **48–53**, **54a−f** (including 2D NMR spectra of compounds **6–8**, **12**, **21a**, **21b**, **25–27**, **30**, **32**, **38b**, **42**, **50**, **51**, **54e**) S39

Methyl 2-(1,3-benzodioxol-5-ylsulfamoyl)-4-chlorobenzoate (10). To a vigorously stirred solution of 1,3-benzodioxol-5-amine (14.40 g, 105 mmol) and *N*,*N*-diethylaniline (15.67 g, 105 mmol, 16.7 mL) in MeOH (150 mL), methyl 4-chloro-2-chlorosulfonylbenzoate (**9**, 26.91 g, 100 mmol) was added over a period of 5 min while cooling the reaction mixture with tap water. It was stirred at room temperature for 1 h and at 0–5 °C for 1 h. The crystalline product separated was filtered and purified by dry-column flash chromatography on a short silica gel column (thickness of stationary phase: 30 mm, eluent: heptane/DCM 1:1, DCM). After evaporation of the solvents, the solid residue was triturated with MeOH, filtered and air-dried to afford **10** (35.10 g, 95%) as colorless crystals. M. p. 166.5–168.5 °C (CH_3CN). IR (KBr): 3227, 1713, 1292, 1171, 1031, 690 cm^{-1}. ^{1}H NMR (500 MHz, DMSO-d_6): 10.05 (br s, 1H), 7.86 (d, ^4J = 2.1 Hz, 1H), 7.79 (dd, ^4J = 2.1 Hz, ^3J = 8.3 Hz, 1H), 7.68 (d, ^3J = 8.3 Hz, 1H), 6.82 (d, ^3J = 8.3 Hz, 1H), 6.68 (d, ^4J = 2.2 Hz, 1H), 6.53 (dd, ^4J = 2.2 Hz, ^3J = 8.3 Hz, 1H), 5.99 (s, 2H), 3.83 (s, 3H). ^{13}C NMR (125 MHz, DMSO-d_6): 166.72, 147.71, 145.10, 138.78, 135.52, 133.08, 131.35, 131.04, 130.51, 128.25, 115.57, 108.50, 104.13, 101.63, 53.31. MS (EI): M^+=369 (1 Cl). HRMS (ESI): m/z calcd. for $C_{15}H_{13}ClNO_6S$ $[M+H]^+$: 370.0146, found: 370.0151.

Methyl 4-chloro-2-(2,3-dihydro-1,4-benzodioxin-6-ylsulfamoyl)benzoate (11). To a vigorously stirred solution of 2,3-dihydro-1,4-benzodioxin-6-amine (29.03 g, 192 mmol) and *N*,*N*-diethylaniline (28.65 g, 192 mmol, 30.6 mL) in MeOH (300 mL), methyl 4-chloro-2-chlorosulfonylbenzoate (**9**, 50.05 g, 186 mmol) was added over a period of 10 min while cooling the reaction mixture with tap water. An exothermic reaction took place, and the product started to crystallize. The brown suspension was stirred at room temperature for 1 h, then at 0–5 °C for 1 h. The crystalline product was filtered and purified by dry-column flash chromatography on a short silica gel column (thickness of stationary phase: 30 mm, eluent: DCM). After evaporation of the solvent, the solid residue was triturated with MeOH, filtered and air-dried to afford **11** (66.0 g, 92%) as colorless crystals. M. p. 153–154 °C (MeOH). IR (KBr): 3266, 1715, 1504, 1175 cm^{-1}. ^{1}H NMR (200 MHz, $CDCl_3$): 7.96 (br s, 1H), 7.83 (d, ^4J = 1.8 Hz, 1H), 7.80 (d, ^3J = 7.7 Hz, 1H), 7.54 (dd, ^4J = 2.2 Hz, ^3J = 8.4 Hz, 1H), 6.70 (d, ^3J = 8.4 Hz, 1H), 6.70 (d, ^4J = 2.6 Hz, 1H), 6.59 (dd, ^4J = 2.6 Hz, ^3J = 8.8 Hz, 1H), 4.20 (s, 4H), 4.02 (s, 3H). ^{13}C NMR (50 MHz, $CDCl_3$): 167.26, 143.60, 142.25, 140.17, 138.18, 132.42, 132.15, 130.64, 129.27, 128.45, 117.48, 116.99, 113.12, 64.21, 64.11, 53.63. MS (ESI): $[M-H]^-$ = 382 (1 Cl). HRMS (ESI): m/z calcd. for $C_{16}H_{15}ClNO_6S$ $[M+H]^+$: 384.0303, found: 384.0309.

Methyl 2-[1,3-benzodioxol-5-yl(methyl)sulfamoyl]-4-chlorobenzoate (12). To a solution of compound **10** (31.08 g, 84 mmol) in DMF (75 mL), anhydrous K_2CO_3 (15.20 g, 110 mmol) was added. To the vigorously stirred suspension iodomethane (15.61 g, 110 mmol, 6.9 mL) was added dropwise and the reaction mixture was stirred at room temperature for 4 h. Water (450 mL) was added and a thick oil separated. The supernatant was decanted, the oil was dissolved in DCM (100 mL), the solution was washed with water (2 × 100 mL), the organic phase was dried over Na_2SO_4 and evaporated in vacuo. The residual dense orange oil was dissolved in a heptane/DCM mixture 2:1 (150 mL), silica gel (20 g, Kieselgel 60 H) was added, and it was stirred at room temperature for 30 min. The filtrate was evaporated in vacuo to afford **12** (31.4 g of dense yellow oil, yield 97%). The TLC pure intermediate was transformed in the next step without further purification. IR (film): 1739, 1484, 1354, 1292, 1253, 1162, 1120, 1057, 1037, 940, 836 cm^{-1}. ^{1}H NMR (600 MHz, $CDCl_3$): 7.56 (dd, ^{4}J = 2.1 Hz, ^{3}J = 8.2 Hz, 1H), 7.46 (d, ^{4}J = 2.0 Hz, 1H), 7.44 (d, ^{3}J = 8.2 Hz, 1H), 6.73 (d, ^{3}J = 8.3 Hz, 1H), 6.72 (d, ^{4}J = 2.2 Hz, 1H), 6.60 (dd, ^{4}J = 2.2 Hz, ^{3}J = 8.3 Hz, 1H), 6.00 (s, 2H), 3.89 (s, 3H), 3.26 (s, 3H). ^{13}C NMR (150 MHz, $CDCl_3$): 167.44, 147.94, 147.29, 136.96, 135.97, 134.40, 132.44, 131.66, 129.92, 129.51, 121.11, 109.04, 108.07, 101.74, 53.27, 39.43. COSY: 7.46–7.56–7.44, 6.73–6.60–6.72. HSQC (140 Hz): 7.56–132.44, 7.46–129.92, 7.44–129.51, 6.73–108.07, 6.72–109.04, 6.60–121.11, 6.00–101.72, 3.89–53.27, 3.26–39.43. HMBC (8 Hz, 140 Hz): 7.56–131.66, 7.46–(135.97, 132.44, 131.66), 7.44–(167.44, 136.96, 135.97), 6.73–(147.94, 134.40), 6.72–(147.29, 121.11), 6.60–(147.29, 109.04), 6.00–(147.94, 147.29), 3.89–167.44, 3.26–134.40. HRMS (ESI): m/z calcd. for $C_{16}H_{15}ClNO_6S$ $[M+H]^+$: 384.0304, found: 384.0307.

Methyl 4-chloro-2-[2,3-dihydro-1,4-benzodioxin-6-yl(methyl)sulfamoyl]benzoate (13). To a solution of compound **11** (46.06 g, 120 mmol) in DMF (100 mL), anhydrous K_2CO_3 (21.56 g, 156 mmol) was added, and the suspension was stirred at room temperature for 15 min. To the vigorously stirred suspension iodomethane (22.14 g, 156 mmol, 9.7 mL) was added dropwise while cooling the reaction mixture with tap water. The reaction mixture was stirred at room temperature for 1 h. Water (700 mL) was added and the suspension obtained was stirred at room temperature for 2 h. The product separated was filtered, washed with water (2 × 100 mL), and air-dried to give **13** (47.05 g, 98%) as colorless crystals, suitable to transform in the next step without further purification. A small sample was subjected to dry-column chromatography on a short silica gel column (thickness of stationary phase: 30 mm, eluent: heptane/DCM 2:1, 1:1, DCM). After evaporation of solvents, the oily residue was triturated with cold DIPE to afford pure **13** as colorless crystals. M. p. 97–99 °C (MeOH). IR (KBr): 1733, 1498, 1345, 1294, 1059 cm^{-1}. ^{1}H NMR (200 MHz, $CDCl_3$): 7.54 (dd, ^{4}J = 1.8 Hz, ^{3}J = 8.1 Hz, 1H), 7.43 (d, ^{3}J = 8.1 Hz,

1H), 7.43 (d, ^4J = 2.2 Hz, 1H), 6.80 (d, ^3J = 8.8 Hz, 1H), 6.72 (d, ^4J = 2.6 Hz, 1H), 6.65 (dd, ^4J = 2.6 Hz, ^3J = 8.8 Hz, 1H), 4.25 (s, 4H), 3.87 (s, 3H), 3.24 (s, 3H). ^{13}C NMR (50 MHz, $CDCl_3$): 167.39, 143.48, 143.33, 136.98, 135.85, 133.80, 132.32, 131.64, 129.83, 129.45, 120.53, 117.31, 116.71, 64.26, 64.16, 53.16, 39.13. MS (GC-EI): M^+ = 397 (1 Cl). HRMS (ESI): m/z calcd. for $C_{17}H_{17}ClNO_6S$ $[M+H]^+$: 398.0459, found: 398.0467.

2-[1,3-Benzodioxol-5-yl(methyl)sulfamoyl]-4-chlorobenzoic acid (14). A solution of NaOH (8.00 g, 200 mmol) in water (150 mL) was added to the solution of compound **12** (30.70 g, 80 mmol) in MeOH (150 mL) and it was refluxed for 1 h. Water (750 mL) was added, the solution was cooled with ice-water, and an aqueous HCl solution (5%, 150 mL, 205 mmol HCl) was added in 20 min (pH 1). The colorless suspension obtained was stirred for 30 min while cooling with ice-water, it was filtered, washed with water, and dried over P_2O_5 to afford **14** (28.6 g, 97%) as off-white crystals. M. p. 153–155 °C (CH_3CN, colorless crystals). IR (KBr): 2700, 1706, 1337, 844 cm^{-1}. ^{1}H NMR (500 MHz, DMSO-d_6): 13.7 (br s, 1H), 7.81 (dd, ^4J = 2.0 Hz, ^3J = 8.2 Hz, 1H), 7.66 (d, ^3J = 8.2 Hz, 1H), 7.32 (d, ^4J = 1.8 Hz, 1H), 6.88 (d, ^3J = 8.2 Hz, 1H), 6.82 (d, ^4J = 1.9 Hz, 1H), 6.64 (dd, ^4J = 2.2 Hz, ^3J = 8.2 Hz, 1H), 6.06 (s, 2H), 3.19 (s, 3H). ^{13}C NMR (125 MHz, DMSO-d_6): 168.03, 147.63, 146.92, 135.70, 134.34, 134.08, 133.88, 133.26, 130.22, 128.85, 121.00, 108.70, 108.20, 101.93, 39.14. MS (EI): M^+ = 369 (1 Cl). HRMS (ESI): m/z calcd. for $C_{15}H_{13}ClNO_6S$ $[M+H]^+$: 370.0146, found: 370.0146.

4-Chloro-2-[2,3-dihydro-1,4-benzodioxin-6-yl(methyl)sulfamoyl]benzoic acid (15). A solution of NaOH (11.80 g, 295 mmol) in water (200 mL) was added to the suspension of compound **13** (46.94 g, 118 mmol) in MeOH (200 mL) and the mixture was refluxed for 1 h. The solution obtained was diluted with water (750 mL), cooled with ice-water, and an aqueous HCl solution (5%, 225 mL, 308 mmol HCl) was added in 20 min (pH 1). The partly oily product separated was stirred at room temperature for 3 h. The suspension obtained was filtered, washed thoroughly with water (3 × 100 mL), and dried over P_2O_5 to afford **15** (44.8 g, 99%) as off-white crystals. M. p. 156–158 °C (CH_3CN, colorless crystals). IR (KBr): 3251, 1740, 1504, 1337, 1232, 1065 cm^{-1}. ^{1}H NMR (200 MHz, DMSO-d_6): 13.68 (br s, 1H), 7.81 (dd, ^4J = 1.8 Hz, ^3J = 8.2 Hz, 1H), 7.67 (d, ^3J = 8.2 Hz, 1H), 7.29 (d, ^4J = 1.8 Hz, 1H), 6.85 (d, ^3J = 8.5 Hz, 1H), 6.73 (d, ^4J = 2.1 Hz, 1H), 6.65 (dd, ^4J = 2.4 Hz, ^3J = 8.6 Hz, 1H), 4.24 (s, 4H), 3.19 (s, 3H). ^{13}C NMR (50 MHz, DMSO-d_6): 168.05, 143.40, 143.23, 135.82, 134.16, 133.69, 133.63, 133.24, 130.22, 128.83, 120.15, 117.31, 116.39, 64.23, 38.97. MS (EI): M^+ = 383 (1 Cl). HRMS (ESI): m/z calcd. for $C_{16}H_{15}ClNO_6S$ $[M+H]^+$: 384.0303, found: 384.0310.

8-Chloro-5-methyl[1,3]benzodioxolo[5,6-*c*]benzo[*f*][1,2]thiazepin-11(5*H*)-one 6,6-dioxide (6). To the suspension of **14** (24.04 g, 65 mmol) in DCM (200 mL), PCl_5 (16.24 g, 78 mmol) was added in portions, and the solution obtained was refluxed for 8.5 h. It was cooled to 0–5 °C, and $SnCl_4$ (37.25 g, 143 mmol, 16.7 mL) was added dropwise over a period of 10 min. The thick suspension obtained was stirred at 0–5 °C for 2 h, then it was allowed to warm to room temperature. The orange suspension was poured onto a mixture of crushed ice (600 g) and conc hydrochloric acid (50 mL) and DCM (100 mL). The mixture was stirred until warming to room temperature (1 h), the product separated was filtered, washed with diluted hydrochloric acid (5%, 2 × 50 mL) and water (2 × 50 mL), and air-dried to give crude **6** (17.17 g of yellow solid). The two-phase filtrate was separated, the strongly acidic aqueous phase was washed with DCM (2 × 50 mL), the combined organic phases were washed with water (100 mL), dried over Na_2SO_4, evaporated, and the oily residue was triturated with ether (50 mL) to afford a second crop of **6** (1.94 g of brown solid). The two crops were purified by dry-column flash chromatography on a short silica gel column (thickness of the stationary phase: 30 mm, eluent: DCM). After evaporation of the solvent, the residue was triturated with Et_2O (50 mL) to give **6** (17.34 g, 76%) as pale yellow crystals. M. p. 268–271 °C (CH_3CN, decomp.). IR (KBr): 1639, 1623, 1482, 1353, 1245, 1033 cm^{-1}. ^1H NMR (600 MHz, $CDCl_3$): 8.01 (d, ^3J = 8.4 Hz, 1H), 7.95 (d, ^4J = 2.2 Hz, 1H), 7.74 (s, 1H), 7.67 (dd, ^4J = 2.2 Hz, ^3J = 8.4 Hz, 1H), 6.79 (s, 1H), 6.11 (s, 2H), 3.24 (s, 3H). ^{13}C NMR (150 MHz, $CDCl_3$): 186.97, 153.31, 147.00, 138.76, 137.99, 137.95, 133.99, 133.81, 133.18, 126.34, 125.87, 109.94, 105.60, 102.88, 39.38. COSY: 8.01–7.67–7.95. HSQC (140 Hz): 8.01–133.99, 7.95–125.878, 7.74–109.94, 7.67–133.18, 6.79–105.60, 6.11–102.88, 3.24–39.38. HMBC (8 Hz, 140 Hz): 8.01–(186.97, 138.76, 137.99), 7.95–(133.81, 133.18), 7.74–(186.97, 153.31, 137.95), 7.67–(133.81, 125.87), 6.79–(153.31, 147.00, 137.95, 126.34), 6.11–(153.31, 147.00), 3.24–137.95. MS (EI): M^+ = 351 (1 Cl). HRMS (ESI): m/z calcd. for $C_{15}H_{11}ClNO_5S$ $[M+H]^+$: 352.0041, found: 352.0043.

8-Chloro-5-methyl-5,11-dihydro[1,3]benzodioxolo[5,6-*c*]benzo[*f*][1,2]thiazepin-11-ol 6,6-dioxide (16). To a suspension of **6** (7.04 g, 20 mmol) in DMF (100 mL) and EtOH (100 mL), $NaBH_4$ (1.51 g, 40 mmol) was added, and the reaction mixture was stirred at room temperature for 4.5 h. Water (800 mL) was added dropwise in 30 min. The separated product was filtered, washed with water, and dried in vacuo over P_2O_5 to give **16** (6.72 g, 95%) as colorless crystals. M. p. decomp. from 200 °C (CH_3CN). IR (KBr): 3452, 1503, 1481, 1308, 1141, 1036 cm^{-1}. ^1H NMR (500 MHz, DMSO-d_6): 7.86 (dd, ^4J = 0.6 Hz, ^3J = 8.7 Hz, 1H), 7.76 (d, ^4J = 2.2 Hz, 1H), 7.70 (dd, ^4J = 2.2 Hz, ^3J = 8.4 Hz, 1H), 7.18 (s, 1H), 7.04 (s, 1H), 6.60 (d, ^3J = 5.0 Hz, 1H), 6.16 (d, ^3J = 4.9 Hz, 1H), 6.09 (d, ^4J = 1.0 Hz, 1H), 6.04 (d, ^4J = 1.0 Hz, 1H), 3.37 (s, 3H).

^{13}C NMR (125 MHz, DMSO-d_6): 147.58, 147.33, 140.39, 139.82, 138.34, 132.83, 132.12, 129.01, 128.07, 126.66, 108.70, 105.10, 102.15, 67.51, 37.20. MS (EI): $M^+ = 353$ (1 Cl). HRMS (ESI): m/z calcd. for $C_{15}H_{12}ClNNaO_5S$ $[M+Na]^+$: 376.0017, found: 376.0022.

9-Chloro-6-methyl-2,3,6,12-tetrahydro[1,4]benzodioxino[6,7-*c*]benzo[*f*][1,2]thiazepin-12-ol 7,7-dioxide (17). To a suspension of **7** (15.00 g, 41 mmol) in DMF (100 mL) and EtOH (100 mL), $NaBH_4$ (3.10 g, 82 mmol) was added, and the reaction mixture was stirred at room temperature for 23 h. Water (800 mL) was added dropwise in 30 min. The product separated was filtered, washed with water, and dried in vacuo over P_2O_5 to give **17** (14.75 g, 98%) as colorless crystals. M. p. decomp. from 180 °C (CH_3CN). IR (KBr): 3531, 1500, 1303, 1134, 1064 cm^{-1}. ^1H NMR (200 MHz, DMSO-d_6): 7.85 (d, $^3J = 8.4$ Hz, 1H), 7.75 (d, $^4J = 1.8$ Hz, 1H), 7.70 (dd, $^4J = 2.2$ Hz, $^3J = 8.4$ Hz, 1H), 7.05 (s, 1H), 7.02 (d, $^4J = 0.7$ Hz, 1H), 6.53 (d, $^3J = 3.3$ Hz, 1H), 6.10 (bd, $^4J = 2.6$ Hz, 1H), 4.24 (s, 4H), 3.33 (s, 3H). ^{13}C NMR (50 MHz, DMSO-d_6): 143.53, 143.23, 140.34, 139.44, 136.21, 132.76, 132.23, 128.95, 128.65, 126.67, 117.01, 114.25, 67.95, 64.33, 64.24, 37.88. MS (EI): $M^+ = 367$ (1 Cl). HRMS (ESI): m/z calcd. for $C_{16}H_{14}ClNNaO_5S$ $[M+Na]^+$: 390.0173, found: 390.0176.

8,11-Dichloro-5-methyl-5,11-dihydro[1,3]benzodioxolo[5,6-*c*]benzo[*f*][1,2]thiazepine 6,6-dioxide (18). To a vigorously stirred suspension of **16** (6.19 g, 17.5 mmol) in DCM (40 mL), $SOCl_2$ (1.52 mL, 2.50 g, 21 mmol) was added dropwise at room temperature. The solution obtained was stirred at room temperature for 1.5 h, then evaporated in vacuo. The residue was triturated with DIPE (20 mL), filtered and dried in vacuo to give crude **18** (6.51 g, 100%) as colorless crystals. M. p. decomp. from 164 °C. The product is of suitable quality for use in the next step. IR (KBr): 1503, 1490, 1340, 1229, 1031 cm^{-1}. ^1H NMR (500 MHz, $CDCl_3$): 7.96 (d, $^4J = 2.1$ Hz, 1H), 7.48 (dd, $^4J = 2.2$ Hz, $^3J = 8.3$ Hz, 1H), 7.42 (d, $^3J = 8.3$ Hz, 1H), 6.94 (s, 1H), 6.82 (s, 1H), 6.06 (d, $^4J = 1.4$ Hz, 1H), 6.02 (d, $^4J = 1.3$ Hz, 1H), 5.98 (s, 1H), 3.52 (s, 3H). ^{13}C NMR (125 MHz, $CDCl_3$): 149.78, 147.65, 141.50, 136.17, 133.54, 133.11, 132.49, 132.33, 131.19, 127.89, 109.51, 108.97, 102.53, 63.30, 38.98. MS (EI): $M^+ = 371$ (2 Cl). HRMS (TOF MS EI): m/z calcd. for $[C_{15}H_{11}Cl_2NO_4S]^+$: 370.9786, found: 370.9796.

9,12-Dichloro-6-methyl-2,3,6,12-tetrahydro[1,4]benzodioxino[6,7-*c*]benzo[*f*][1,2]thiazepine 7,7-dioxide (19). To a vigorously stirred suspension of **17** (14.71 g, 40 mmol) in DCM (80 mL), $SOCl_2$ (3.5 mL, 5.71 g, 48 mmol) was added dropwise at room temperature. The solution obtained was stirred at room temperature for 3.5 h. Hexane (240 mL) was added to the suspension formed, the product was filtered, washed with hexane (2 × 30 mL) and dried in vacuo to give crude **19** (14.50 g, 94%) as colorless crystals. M. p. decomp. from 160 °C. The product is of suitable quality for use in the next step. IR (KBr): 1512, 1327, 1142,

1065 cm^{-1}. ^{1}H NMR (200 MHz, $CDCl_3$): 7.97 (d, ^{4}J = 1.8 Hz, 1H), 7.48 (dd, ^{4}J = 2.0 Hz, ^{3}J = 8.4 Hz, 1H), 7.43 (d, ^{3}J = 8.4 Hz, 1H), 7.02 (s, 1H), 6.90 (s, 1H), 5.99 (s, 1H), 4.26 (s, 4H), 3.52 (s, 3H). ^{13}C NMR (50 MHz, $CDCl_3$): 145.42, 143.38, 141.71, 136.08, 133.52, 132.69, 132.32, 132.11, 130.27, 127.98, 118.30, 118.25, 64.32, 64.25, 63.39, 39.26. MS (EI): M^{+} = 385 (2 Cl). HRMS (TOF MS EI): m/z calcd. for $[C_{16}H_{13}Cl_2NO_4S]^+$: 384.9942, found: 384.9945.

Ethyl 7-[(8-chloro-5-methyl-6,6-dioxido-5,11-dihydro[1,3]benzodioxolo[5,6-*c*]benzo[*f*][1,2]thiazepin-11-yl)amino]heptanoate (20a). To a solution of ethyl 7-aminoheptanoate (2.77 g, 16 mmol) in CH_3CN (15 mL) crude **18** (2.60 g, 7 mmol) was added, the solution obtained in 5 min was stirred at room temperature for 1 h. It was evaporated in vacuo, the residue was dissolved in DCM (40 mL), washed with water (2 × 20 mL), dried over Na_2SO_4, and evaporated in vacuo. The oily residue (4.3 g) was purified by dry-column chromatography on a short silica gel column (thickness of stationary phase: 30 mm, eluent: heptane/DCM 1:1, DCM, DCM/MeOH 100:1, 100:2, 100:4). After evaporation of solvents in vacuo **20a** (2.97 g, 83%) was obtained as a pale yellow oil. IR (film): 2932, 1731, 1487, 1328, 1153, 1037 cm^{-1}. ^{1}H NMR (500 MHz, $CDCl_3$): 7.92 (d, ^{4}J = 1.7 Hz, 1H), 7.42 (m, 2H), 6.87 (s, 1H), 6.80 (s, 1H), 4.93 (s, 1H), 4.11 (q, ^{3}J = 7.2 Hz, 2H), 3.38 (s, 3H), 2.50 (t, ^{3}J = 7.1 Hz, 2H), 2.28 (t, ^{3}J = 7.5 Hz, 2H), 1.76 (br s, 1H), 1.61 (~qn, ^{3}J = 7.2 Hz, 2H), 1.50 (m, 2H), 1.32 (m, 4H), 1.24 (t, ^{3}J = 7.1 Hz, 3H). ^{13}C NMR (125 MHz, $CDCl_3$): 173.63, 147.88, 147.62, 140.73, 137.39, 134.44, 134.13, 131.87, 131.19, 129.90, 128.26, 108.54, 107.98, 101.96, 64.55, 60.11, 48.18, 38.13, 34.15, 29.85, 28.90, 26.88, 24.75, 14.18. MS (ESI): [M+H]$^{+}$: 509 (1 Cl). HRMS (ESI): m/z calcd. for $C_{24}H_{30}ClN_2O_6S$ [M+H]$^{+}$: 509.1508, found: 509.1505.

7-[(8-Chloro-5-methyl-6,6-dioxido-5,11-dihydro[1,3]benzodioxolo[5,6-*c*]benzo[*f*][1,2]thiazepin-11-yl)amino]heptanoic acid (20b). To a solution of ester **20a** (2.55 g, 5 mmol) in EtOH (30 mL), a solution of NaOH (0.240 g, 6 mmol) in water (7.5 mL) was added at room temperature and it was stirred at room temperature for 25 h. Water (15 mL) was added and the ethanol was removed by evaporation in vacuo. Water (8 mL) was added, and the solution was neutralized with an aqueous HCl solution (0.50 mL of cc. HCl dissolved in 10 mL of water, containing 6 mmol of HCl). The milky reaction mixture was stirred at room temperature for 3 days, the solid product obtained was filtered, washed with water (2 × 3 mL), air-dried and subjected to dry-column chromatography on a short silica gel column (thickness of stationary phase: 25 mm, eluent: DCM, DCM/MeOH 100:2, 100:4). After evaporation of solvents the solid residue was triturated with CH_3CN (5 mL) to afford **20b** (1.92 g, 80%) as colorless crystals. M. p. 126–128 °C (CH_3CN). IR (KBr): 3081, 1712, 1493, 1326, 1224, 1034 cm^{-1}. ^{1}H NMR (500 MHz, DMSO-d_6): 7.75 (d, ^{4}J = 1.8 Hz, 1H), 7.68 (dd, ^{4}J = 2.2 Hz, ^{3}J =

8.5 Hz, 1H), 7.65 (d, ^3J = 8.6 Hz, 1H), 7.15 (s, 1H), 7.04 (s, 1H), 6.08 (d, ^4J = 0.7 Hz, 1H), 6.06 (d, ^4J = 0.6 Hz, 1H), 5.11 (s, 1H), 3.37 (s, 3H), 2.44 (m, 2H), 2.18 (t, ^3J = 7.3 Hz, 2H), 1.47 (m, 4H), 1.26 (m, 4H). ^{13}C NMR (125 MHz, DMSO-*d*$_6$): 174.68, 147.41, 147.38, 141.30, 138.55, 135.93, 132.77, 131.96, 130.82, 126.86, 109.08, 107.38, 102.18, 61.99, 47.74, 37.83, 33.81, 29.47, 28.69, 26.73, 24.66. MS (ESI): $[M+H]^+$: 481 (1 Cl). HRMS (ESI): m/z calcd. for $C_{22}H_{26}ClN_2O_6S$ $[M+H]^+$: 481.1195, found: 481.1201.

8-Chloro-*N*,5-dimethyl-5,11-dihydro[1,3]benzodioxolo[5,6-*c*]benzo[*f*][1,2]thiazepin-11-amine 6,6-dioxide (20c). To the solution of methylamine (1.40 g, 45 mmol) in dioxane (45 mL) crude **18** (3.35 g, 9 mmol) was added, and the suspension obtained was stirred at room temperature for 1 h. Water (150 mL) was added dropwise, the product precipitated was filtered, washed with water (2 × 10 mL), air-dried and purified by dry-column chromatography on a short silica gel column (thickness of stationary phase: 30 mm, eluent: heptane/DCM 1:1, 1:2, DCM, DCM/MeOH 100:1, 100:2, 100:4). After evaporation of solvents, the solid residue was triturated with CH_3CN (15 mL) to afford **20c** (1.86 g, 56%) as colorless crystals. M. p. decomp. from 218 °C (CH_3CN). IR (KBr): 1501, 1485, 1325, 1154, 1036 cm^{-1}. ^{1}H NMR (500 MHz, $CDCl_3$): 7.93 (d, ^4J = 2.1 Hz, 1H), 7.43 (dd, ^4J = 2.0 Hz, ^3J = 8.4 Hz, 1H), 6.87 (s, 1H), 6.81 (s, 1H), 5.98 (d, ^4J = 1.1 Hz, 1H), 5.97 (d, ^4J = 1.1 Hz, 1H), 4.82 (s, 1H), 3.37 (s, 3H), 2.38 (s, 3H), 1.86 (br s, 1H). ^{13}C NMR (125 MHz, $CDCl_3$): 147.96, 147.64, 140.69, 136.96, 134.24, 133.82, 131.90, 131.34, 130.11, 128.35, 108.51, 108.23, 101.99, 66.52, 38.21, 34.98. MS (ESI): $[M+H]^+$: 367 (1 Cl). HRMS (ESI): m/z calcd. for $C_{16}H_{16}ClN_2O_4S$ $[M+H]^+$: 367.0514, found: 367.0516.

8-Chloro-5-methyl-*N*-(2-morpholin-4-ylethyl)-5,11-dihydro[1,3]benzodioxolo[5,6-*c*]benzo[*f*][1,2]thiazepin-11-amine 6,6-dioxide (20d). To the solution of 4-(2-aminoethyl)morpholine (1.15 g, 8.8 mmol) in CH_3CN (15 mL) crude **18** (1.49 g, 4 mmol) was added, and the suspension obtained was stirred at room temperature for 1 h. Water (50 mL) was added dropwise, the product separated was filtered, washed with water (3 × 3 mL) air-dried and purified by dry-column chromatography on a short silica gel column (thickness of stationary phase: 25 mm, eluent: DCM, DCM/MeOH 100:2). After evaporation of solvents, the solid residue was triturated with CH_3CN to afford **20d** (1.58 g, 84%) as colorless crystals. M. p. 207–209 °C (CH_3CN, decomp.). IR (KBr): 3331, 1488, 1326, 1116, 1029 cm^{-1}. ^{1}H NMR (500 MHz, $CDCl_3$): 7.93 (m, 1H), 7.42 (m, 2H), 6.89 (s, 1H), 6.81 (s, 1H), 5.98 (d, ^4J = 1.3 Hz, 1H), 5.97 (d, ^4J = 1.4 Hz, 1H), 4.93 (s, 1H), 3.67 (m, 4H), 3.42 (s, 3H), 2.63 (m, 2H), 2.50 (m, 2H), 2.37 (m, 4H). ^{13}C NMR (125 MHz, $CDCl_3$): 147.96, 147.70, 140.84, 137.43, 134.77, 134.24, 131.82, 131.17, 129.80, 128.31, 108.51, 107.75, 102.00, 66.88 (2C), 64.38, 58.05, 53.68 (2C), 44.27,

37.97. MS (ESI): [M+H]$^+$: 466 (1 Cl). HRMS (ESI): m/z calcd. for $C_{21}H_{25}ClN_3O_5S$ [M+H]$^+$: 466.1198, found: 466.1202.

8-Chloro-5-methyl-11-pyrrolidin-1-yl-5,11-dihydro[1,3]benzodioxolo[5,6-*c*]benzo[*f*][1,2]thiazepine 6,6-dioxide (20e). To the cooled solution of pyrrolidine (0.71 g, 10 mmol, 0.83 mL) in CH_3CN (10 mL) crude **18** (1.49 g, 4 mmol) was added. The suspension formed was stirred at room temperature for 1 h. Water (40 mL) was added dropwise, the product separated was filtered, washed with water (3 × 3 mL), air-dried and purified by dry-column chromatography on a short silica gel column (thickness of stationary phase: 25 mm, eluent: heptane/DCM 2:1, 1:1, DCM, DCM/MeOH 100:1). After evaporation of solvents, the solid residue was triturated with CH_3CN (5 mL) to afford TLC pure **20e** (1.11 g, 68%) as colorless crystals. M. p. decomp. from 215 °C (CH_3CN). IR (KBr): 1483, 1316, 1226, 1033 cm^{-1}. ^{1}H NMR (500 MHz, $CDCl_3$): 7.92 (d, ^4J = 2.2 Hz, 1H), 7.36 (dd, ^4J = 2.2 Hz, ^3J = 8.3 Hz, 1H), 7.30 (d, ^3J = 8.3 Hz, 1H), 6.89 (s, 1H), 6.75 (s, 1H), 6.01 (d, ^4J = 1.3 Hz, 1H), 5.95 (d, ^4J = 1.5 Hz, 1H), 4.05 (br s, 1H), 3.46 (s, 3H), 2.27 (m, 2H), 2.18 (m, 2H), 1.70 (m, 4H). ^{13}C NMR (125 MHz, $CDCl_3$): 148.20, 147.64, 142.76, 135.75, 135.52, 134.88, 132.60, 131.18, 131.07, 128.22, 110.64, 109.34, 102.06, 75.99, 53.71 (2C), 39.58, 23.19 (2C). MS (EI): [M]$^+$: 406 (1 Cl). HRMS (ESI): m/z calcd. for $C_{19}H_{20}ClN_2O_4S$ [M+H]$^+$: 407.0827, found: 407.0831.

9-Chloro-*N*,6-dimethyl-2,3,6,12-tetrahydro[1,4]benzodioxino[6,7-*c*]benzo[*f*][1,2]thiazepin-12-amine 7,7-dioxide (21c). To the cold solution of methylamine (0.745 g, 24 mmol) in dioxane (17 mL) crude **19** (1.85 g, 4.8 mmol) was added, and the suspension obtained was stirred at room temperature for 45 min. After evaporation in vacuo the solid residue was triturated with water (15 mL), filtered, air-dried and purified by dry-column flash chromatography on a short silica gel column (thickness of stationary phase: 25 mm, eluent: DCM, DCM/MeOH 100:1). After evaporation of solvents, the solid residue was triturated with DIPE (10 mL) to afford **21c** (1.71 g, 93%) as colorless crystals. M. p. decomp. from 212 °C (CH_3CN). IR (KBr): 1508, 1324, 1064, 594 cm^{-1}. ^{1}H NMR (500 MHz, $CDCl_3$): 7.95 (d, ^4J = 2.2 Hz, 1H), 7.44 (dd, ^4J = 2.2 Hz, ^3J = 8.2 Hz, 1H), 7.39 (d, ^3J = 8.3 Hz, 1H), 6.92 (s, 1H), 6.85 (s, 1H), 4.73 (s, 1H), 4.24 (s, 4H), 3.33 (s, 3H), 2.34 (s, 3H). ^{13}C NMR (125 MHz, $CDCl_3$): 143.71, 143.29, 140.74, 136.68, 134.20, 132.02, 131.95, 131.28, 130.99, 128.45, 117.90, 116.92, 67.34, 64.32, 64.27, 38.75, 34.91. MS (EI): [M]$^+$: 380 (1 Cl). HRMS (ESI): m/z calcd. for $C_{17}H_{18}ClN_2O_4S$ [M+H]$^+$: 381.0670, found: 381.0677.

9-Chloro-6-methyl-*N*-(2-morpholin-4-ylethyl)-2,3,6,12-tetrahydro[1,4]benzodioxino[6,7-*c*]benzo[*f*][1,2]thiazepin-12-amine 7,7-dioxide (21d). To the solution of 4-(2-aminoethyl)morpholine (1.15 g, 8.8 mmol) in CH_3CN (15 mL) crude **19** (1.55 g, 4 mmol) was

added. The solution obtained was stirred at room temperature for 1 h. Water (50 mL) was added dropwise in 30 min, the product separated was filtered, washed with water (2 × 5 mL), air-dried and purified by dry-column chromatography on a short silica gel column (thickness of stationary phase: 30 mm, eluent: DCM, DCM/MeOH 100:2). After evaporation of solvents, the solid residue was triturated with CH_3CN (10 mL), filtered and recrystallized from CH_3CN (85 mL) to afford **21d** (1.46 g, 75%) as colorless crystals. M. p. decomp. from 217 °C (CH_3CN). IR (KBr): 1509, 1323, 1147, 1065 cm^{-1}. ^{1}H NMR (500 MHz, CDCl3): 7.94 (d, ^4J = 2.1 Hz, 1H), 7.43 (dd, ^4J = 2.1 Hz, ^3J = 8.3 Hz, 1H), 7.39 (d, ^3J = 8.3 Hz, 1H), 6.94 (s, 1H), 6.85 (s, 1H), 4.85 (s, 1H), 4.23 (s, 4H), 3.67 (m, 4H), 3.39 (s, 3H), 2.58 (m, 2H), 2.47 (m, 2H), 2.46 (br s, 1H), 2.35 (m, 4H). ^{13}C NMR (125 MHz, $CDCl_3$): 143.65, 143.33, 140.95, 137.18, 134.12, 133.06, 131.82, 130.97, 130.54, 128.30, 117.20, 116.96, 66.85 (2C), 64.96, 64.29, 64.20, 58.04, 53.63 (2C), 44.17, 38.41. MS (EI): $[M]^+$: 479 (1 Cl). HRMS (ESI): m/z calcd. for $C_{22}H_{27}ClN_3O_5S$ $[M+H]^+$: 480.1354, found: 480.1353.

9-Chloro-6-methyl-*N*-(3-morpholin-4-ylpropyl)-2,3,6,12-tetrahydro[1,4]benzodioxino[6,7-*c*]benzo[*f*][1,2]thiazepin-12-amine 7,7-dioxide (21e). To the solution of 4-(3-aminopropyl)morpholine (1.27 g, 8.8 mmol) in CH_3CN (15 mL) crude **19** (1.55 g, 4 mmol) was added. The solution obtained was stirred at room temperature for 1.5 h. Water (100 mL) was added dropwise, the oily product separated was extracted with DCM (2 × 25 mL), the combined organic phases were washed with water (20 mL), dried over Na_2SO_4, and partly evaporated in vacuo (to a volume of 20 mL). The solution obtained was purified by dry-column chromatography on a short silica gel column (thickness of stationary phase: 30 mm, eluent: DCM, DCM/MeOH 100:2, 100:4). After evaporation of solvents, the oily residue was triturated with water (30 mL), filtered and recrystallized from CH_3CN to afford **21e** (1.29 g, 65%) as bright colorless crystals. M. p. 164–166 °C (CH_3CN). IR (KBr): 1500, 1304, 1146, 1116, 1069 cm^{-1}. ^{1}H NMR (500 MHz, $CDCl_3$): 7.93 (d, ^4J = 0.7 Hz, 1H), 7.43 (m, 2H), 6.93 (s, 1H), 6.85 (s, 1H), 4.92 (s, 1H), 4.23 (s, 4H), 3.66 (m, 4H), 3.35 (s, 3H), 2.57 (m, 2H), 2.42 (m, 4H), 2.40 (t, ^3J = 7.0 Hz, 2H), 2.35 (br s, 1H), 1.69 (m, 2H). ^{13}C NMR (125 MHz, $CDCl_3$): 143.55, 143.35, 140.68, 137.45, 134.00, 133.13, 131.87, 130.97, 130.04, 128.36, 116.97, 116.93, 66.89 (2C), 64.32, 64.30, 64.21, 57.31, 53.74 (2C), 46.90, 38.39, 26.37. MS (ESI): $[M+H]^+$: 494 (1 Cl). HRMS (ESI): m/z calcd. for $C_{23}H_{29}ClN_3O_5S$ $[M+H]^+$: 494.1511, found: 494.1513.

9-Chloro-6-methyl-*N*-(4-morpholin-4-ylbutyl)-2,3,6,12-tetrahydro[1,4]benzodioxino[6,7-*c*]benzo[*f*][1,2]thiazepin-12-amine 7,7-dioxide (21f). To the solution of 4-(4-aminobutyl)morpholine (1.39 g, 8.8 mmol) in CH_3CN (15 mL) crude **19** (1.55 g, 4 mmol) was added. The solution obtained was stirred at room temperature for 1 h. Water (60 mL) was added

dropwise in 30 min, the oily product separated was extracted with DCM (3 × 25 mL), the combined organic phases were washed with water (25 mL), dried over Na_2SO_4, and evaporated in vacuo. The residue was purified by dry-column chromatography on a short silica gel column (thickness of stationary phase: 30 mm, eluent: heptane/DCM 1:1, 1:2, DCM, DCM/MeOH 100:2, 100:4). After evaporation of solvents, the foamy residue was dissolved in CH_3CN (20 mL), the opalescent solution was treated with charcoal, the clear solution obtained was evaporated in vacuo to afford **21f** (1.43 g, 70%) as colorless foam. IR (KBr): 3332, 2936, 1507, 1321, 1116, 1066 cm^{-1}. ^1H NMR (500 MHz, $CDCl_3$): 7.93 (d, ^4J = 2.0 Hz, 1H), 7.42 (dd, ^4J = 2.1 Hz, ^3J = 8.3 Hz, 1H), 7.39 (d, ^3J = 8.3 Hz, 1H), 6.91 (s, 1H), 6.84 (s, 1H), 4.86 (s, 1H), 4.22 (s, 4H), 3.68 (m, 4H), 3.34 (s, 3H), 2.49 (~q, ^3J = 3.8 Hz, 2H), 2.39 (m, 4H), 2.29 (t, ^3J = 6.8 Hz, 2H), 1.94 (br s, 1H), 1.50 (m, 4H). ^{13}C NMR (125 MHz, $CDCl_3$): 143.56, 143.21, 140.71, 137.06, 133.99, 132.52, 131.84, 131.04, 130.65, 128.27, 117.50, 116.88, 66.83 (2C), 65.14, 64.22, 64.15, 58.65, 53.61 (2C), 47.89, 38.62, 27.84, 24.18. MS (ESI): $[M+H]^+$: 508 (1 Cl). HRMS (ESI): m/z calcd. for $C_{24}H_{31}ClN_3O_5S$ $[M+H]^+$: 508.1667, found: 508.1669.

9-Chloro-6-methyl-12-pyrrolidin-1-yl-2,3,6,12-tetrahydro[1,4]benzodioxino[6,7-*c*]benzo[*f*][1,2]thiazepine 7,7-dioxide (21g). To the cooled suspension of crude **19** (1.74 g, 4.5 mmol) in CH_3CN (15 mL) pyrrolidine (0.80 g, 11.3 mmol, 0.94 mL) was added in one portion. An exothermic reaction took place, a solution was formed, which was followed by rapid precipitation. The suspension obtained was stirred at room temperature for 1 h. Water (60 mL) was added dropwise, the product separated was filtered, washed with water (3 × 3 mL), and air-dried. It was purified by dry-column chromatography on a short silica gel column (thickness of stationary phase: 30 mm, eluent: DCM). After evaporation of solvent, the solid residue triturated with Et_2O (5 mL) to give **21g** (1.30 g, 69%) as colorless crystals. M. p. decomp. from 216 °C (CH_3CN). IR (KBr): 2933, 1586, 1506, 1320, 1138, 1067 cm^{-1}. ^1H NMR (500 MHz, $CDCl_3$): 7.93 (d, ^4J = 2.1 Hz, 1H), 7.36 (dd, ^4J = 2.2 Hz, ^3J = 8.3 Hz, 1H), 7.29 (d, ^3J = 8.3 Hz, 1H), 6.97 (s, 1H), 6.79 (s, 1H), 4.23 (m, 4H), 4.03 (s, 1H), 3.47 (s, 3H), 2.25 (m, 2H), 2.17 (m, 2H), 1.68 (m, 4H). ^{13}C NMR (125 MHz, $CDCl_3$): 143.84, 143.44, 143.09, 135.79, 134.80, 134.64, 132.72, 131.02, 130.65, 128.33, 119.37, 118.26, 75.64, 64.32, 64.17, 53.68 (2C), 39.93, 23.19 (2C). MS (EI): $[M]^+$: 420 (1 Cl). HRMS (ESI): m/z calcd. for $C_{20}H_{22}ClN_2O_4S$ $[M+H]^+$: 421.0983, found: 421.0985.

9-Chloro-6-methyl-2,3,6,12-tetrahydro[1,4]benzodioxino[6,7-*c*]benzo[*f*][1,2]thiazepin-12-amine 7,7-dioxide (21h). To a cold, 1 m/m % ammonia solution in dioxane (204 g, containing 2.04 g, 120 mmol NH_3) crude **19** (4.63 g, 12 mmol) was added, the thin suspension was stirred at room temperature for 2 h, then evaporated in vacuo. The solid residue (7.1 g) was triturated

with water (20 mL), filtered, washed with water (2 × 10 mL), dried in vacuo and purified by dry-column chromatography on a short silica gel column (thickness of stationary phase: 30 mm, eluent: heptane/DCM 1:1, 1:2, DCM, DCM/MeOH 100:1). After evaporation of solvents, the foamy residue was triturated with DIPE (20 mL), filtered, washed with DIPE (5 mL) to afford **21h** (2.10 g, 48%) as colorless crystals. M. p. decomp. from 200 °C (EtOH/CH_3CN). IR (KBr): 3368, 1503, 1322, 1143, 1062 cm^{-1}. ^1H NMR (500 MHz, DMSO-d_6): 7.79 (d, ^3J = 8.5 Hz, 1H), 7.73 (d, ^4J = 2.3 Hz, 1H), 7.69 (dd, ^4J = 2.3 Hz, ^3J = 8.6 Hz, 1H), 7.05 (s, 1H), 7.03 (s, 1H), 5.47 (s, 1H), 4.23 (s, 4H), 3.32 (s, 3H), 2.57 (br s, 2H). ^{13}C NMR (125 MHz, DMSO-d_6): 143.41, 142.90, 140.85, 140.61, 137.22, 132.27, 132.11, 129.57, 129.34, 126.73, 116.56, 115.04, 64.32, 64.22, 53.17, 37.59. MS (EI): $[M]^+$: 366 (1 Cl). HRMS (ESI): m/z calcd. for $C_{16}H_{16}ClN_2O_4S$ $[M+H]^+$: 367.0514, found: 367.0519.

7-[(9-Chloro-6-methyl-7,7-dioxido-2,3,6,12-tetrahydro[1,4]benzodioxino[6,7-*c*]benzo[*f*][1,2]thiazepin-12-yl)amino]-*N*,*N*-dimethylheptanamide (21i). To a solution of 7-amino-*N*,*N*-dimethylheptanamide (1.45 g, 8.4 mmol) in CH_3CN (20 mL) crude **19** (1.545 g, 4 mmol) was added and the solution obtained was stirred at room temperature for 3.5 h. It was evaporated in vacuo, the residue was dissolved in DCM (50 mL), washed with water (3 × 20 mL), dried over Na_2SO_4, and evaporated in vacuo. The oily residue was purified by dry-column chromatography on a short silica gel column (thickness of stationary phase: 40 mm, eluent: DCM, DCM/MeOH 100:1, 100:2). After evaporation of solvents in vacuo **21i** (1.47 g, 70%) was obtained as a colorless foam. IR (KBr): 3446, 2929, 1641, 1507, 1320, 1143, 1066 cm^{-1}. ^1H NMR (500 MHz, $CDCl_3$): 7.92 (d, ^4J = 2.0 Hz, 1H), 7.43 (dd, ^4J = 2.0 Hz, ^3J = 8.2 Hz, 1H), 7.40 (d, ^3J = 8.4 Hz, 1H), 6.91 (s, 1H), 6.83 (s, 1H), 4.85 (s, 1H), 4.23 (s, 4H), 3.35 (s, 3H), 2.98 (s, 3H), 2.93 (s, 3H), 2.47 (m, 2H), 2.28 (t, ^3J = 7.5 Hz, 2H), 1.87 (br s, 1H), 1.61 (m, 2H), 1.48 (m, 2H), 1.32 (m, 4H). ^{13}C NMR (125 MHz, $CDCl_3$): 172.92, 143.59, 143.27, 140.85, 137.20, 134.01, 132.77, 131.87, 131.08, 130.71, 128.27, 117.51, 116.98, 65.25, 64.26, 64.19, 48.06, 38.63, 37.16, 35.25, 33.13, 29.85, 29.23, 27.04, 24.94. MS (ESI): $[M+H]^+$: 522 (1 Cl). HRMS (ESI): m/z calcd. for $C_{25}H_{33}ClN_3O_5S$ $[M+H]^+$: 522.1824, found: 522.1829.

9-Chloro-6-methyl-*N*-(2-pyrrolidin-1-ylethyl)-2,3,6,12-tetrahydro[1,4]benzodioxino[6,7-*c*]benzo[*f*][1,2]thiazepin-12-amine 7,7-dioxide (21j). To the solution of 4-(2-aminoethyl)pyrrolidine (1.01 g, 8.8 mmol) in CH_3CN (15 mL) crude **19** (1.55 g, 4 mmol) was added. The solution obtained was stirred at room temperature for 1 h. Water (60 mL) was added, the oily product separated was extracted with DCM (30 + 15 mL), the combined organic phases were washed with water (15 mL), dried over Na_2SO_4, and evaporated in vacuo. The oily residue was purified by dry-column chromatography on a short silica gel column (thickness of

stationary phase: 25 mm, eluent: DCM, DCM/MeOH 100:1, 100:2, 100:4). After evaporation of solvents, the solid residue was recrystallized from CH_3CN (50 mL) to afford **21j** (1.35 g, 72%) as colorless crystals. M. p. decomp. from 190 °C (CH_3CN). IR (KBr): 3281, 2788, 1509, 1325, 1159, 1061 cm^{-1}. ^1H NMR (500 MHz, $CDCl_3$): 7.93 (br s, 1H), 7.41 (m, 2H), 6.94 (s, 1H), 6.85 (s, 1H), 4.86 (s, 1H), 4.22 (s, 4H), 3.40 (s, 3H), 2.59 (m, 4H), 2.41 (m, 4H), 2.35 (br s, 1H), 1.73 (m, 4H). ^{13}C NMR (125 MHz, $CDCl_3$): 143.59, 143.31, 141.14, 137.29, 134.04, 133.32, 131.80, 130.89, 130.69, 128.14, 117.31, 117.03, 65.12, 64.27, 64.18, 55.56, 54.08 (2C), 46.73, 38.32, 23.39 (2C). MS (ESI): $[M+H]^+$: 464 (1 Cl). HRMS (ESI): m/z calcd. for $C_{22}H_{27}ClN_3O_4S$ $[M+H]^+$: 464.1405, found: 464.1407.

9-Chloro-6-methyl-*N*-(2-piperidin-1-ylethyl)-2,3,6,12-tetrahydro[1,4]benzodioxino[6,7-*c*]benzo[*f*][1,2]thiazepin-12-amine 7,7-dioxide (21k). To the solution of 4-(2-aminoethyl)piperidine (1.13 g, 8.8 mmol) in CH_3CN (15 mL) crude **19** (1.55 g, 4 mmol) was added. The thin suspension obtained was stirred at room temperature for 1 h. Water (60 mL) was added dropwise in 30 min, the oily product separated was extracted with DCM (30 + 15 mL), the combined organic phases were washed with water (15 mL), dried over Na_2SO_4, and evaporated in vacuo. The oily residue was purified by dry-column chromatography on a short silica gel column (thickness of stationary phase: 25 mm, eluent: DCM, DCM/MeOH 100:1, 100:2). After evaporation of solvents, the foamy residue was dissolved in CH_3CN (5 mL), crystallization occurred when cooling it at 5 °C for 3 days to afford **21k** (1.455 g, 76%) as colorless crystals. M. p. decomp. from 141 °C (CH_3CN). IR (KBr): 3302, 2937, 1508, 1323, 1148, 1065 cm^{-1}. ^1H NMR (500 MHz, $CDCl_3$): 7.93 (m, 1H), 7.41 (m, 2H), 6.94 (s, 1H), 6.84 (s, 1H), 4.86 (d, ^3J = 3.8 Hz, 1H), 4.22 (s, 4H), 3.42 (s, 3H), 2.58 (~q, ^3J = 5.0 Hz, 2H), 2.41 (t, ^3J = 6.0 Hz, 2H), 2.37 (~bq, ^3J = 5.5 Hz, 1H), 2.28 (br s, 4H), 1.52 (~qn, ^3J = 5.6 Hz, 4H), 1.40 (m, 2H). ^{13}C NMR (125 MHz, $CDCl_3$): 143.58, 143.37, 141.14, 137.38, 134.04, 133.54, 131.76, 130.88, 130.48, 128.20, 117.13, 117.08, 64.85, 64.28, 64.19, 58.34, 54.70 (2C), 44.86, 38.41, 25.95 (2C), 24.35. MS (ESI): $[M+H]^+$: 478 (1 Cl). HRMS (ESI): m/z calcd. for $C_{23}H_{29}ClN_3O_4S$ $[M+H]^+$: 478.1562, found: 478.1564.

***N*'-(9-Chloro-6-methyl-7,7-dioxido-2,3,6,12-tetrahydro[1,4]benzodioxino[6,7-*c*]benzo[*f*][1,2]thiazepin-12-yl)-*N*,*N*-dimethylethane-1,2-diamine (21l).** To the solution of 2-(dimethylamino)ethylamine (0.88 g, 10 mmol) in CH_3CN (15 mL) crude **19** (1.70 g, 4.4 mmol) was added. The thick suspension obtained was stirred at room temperature for 1 h. Water (60 mL) was added dropwise, the separated product was filtered, washed with water (2 × 5 mL). The wet crude product was dissolved in DCM (35 mL), water was separated, the organic phase was dried over Na_2SO_4 and evaporated in vacuo. The residue was purified by dry-column

chromatography on a short silica gel column (thickness of stationary phase: 25 mm, eluent: DCM, DCM/MeOH 100:2, 100:4). After evaporation of solvents, the solid residue was crystallized from CH_3CN (30 mL) to afford **21l** (1.54 g, 80%) as colorless crystals. M. p. decomp. from 190 °C (CH_3CN). IR (KBr): 2944, 1509, 1325, 1147, 1060 cm^{-1}. ^1H NMR (500 MHz, $CDCl_3$): 7.92 (br s, 1H), 7.42 (m, 2H), 6.94 (s, 1H), 6.85 (s, 1H), 4.88 (s, 1H), 4.22 (s, 4H), 3.41 (s, 3H), 2.56 (t, ^3J = 5.7 Hz, 2H), 2.39 (t, ^3J = 5.6 Hz, 2H), 2.31 (br s, 1H), 2.15 (s, 6H). ^{13}C NMR (125 MHz, $CDCl_3$): 143.57, 143.32, 141.14, 137.34, 134.02, 133.46, 131.81, 130.84, 130.49, 128.12, 117.14, 117.08, 64.86, 64.26, 64.17, 58.80, 45.52, 45.41 (2C), 38.28. MS (ESI): $[M+H]^+$: 438 (1 Cl). HRMS (ESI): m/z calcd. for $C_{20}H_{25}ClN_3O_4S$ $[M+H]^+$: 438.1249, found: 438.1253.

***N'*-(9-Chloro-6-methyl-7,7-dioxido-2,3,6,12-tetrahydro[1,4]benzodioxino[6,7-*c*]benzo[*f*][1,2]thiazepin-12-yl)-*N*,*N*-dimethylpropane-1,3-diamine (21m).** To the solution of 3-(dimethylamino)-1-propylamine (0.90 g, 8.8 mmol) in CH_3CN (15 mL) crude **19** (1.55 g, 4 mmol) was added. The solution obtained was stirred at room temperature for 1 h. Water (60 mL) was added dropwise, the oily product separated was extracted with DCM (30 + 15 mL), the combined organic phases were washed with water (15 mL), dried over Na_2SO_4, and evaporated in vacuo. The oily residue was purified by dry-column chromatography on a short silica gel column (thickness of stationary phase: 25 mm, eluent: DCM, DCM/MeOH 100:2, 100:4). After evaporation of solvents, the foamy residue was dissolved in CH_3CN (5 mL), crystallization occurred when cooling it at 5 °C overnight to afford **21m** (1.355 g, 75%) as colorless crystals. M. p. 154–156 °C (CH_3CN). IR (KBr): 3199, 1508, 1319, 1066 cm^{-1}. ^1H NMR (500 MHz, $CDCl_3$): 7.92 (br s, 1H), 7.42 (m, 2H), 6.92 (s, 1H), 6.84 (s, 1H), 4.90 (s, 1H), 4.22 (s, 4H), 3.36 (s, 3H), 2.56 (m, 2H), 2.30 (t, ^3J = 7.0 Hz, 2H), 2.22 (br s, 1H), 2.20 (s, 6H), 1.66 (m, 2H). ^{13}C NMR (125 MHz, $CDCl_3$): 143.52, 143.33, 140.76, 137.41, 133.94, 133.18, 131.86, 130.90, 130.22, 128.26, 117.08, 116.92, 64.53, 64.26, 64.17, 58.04, 46.86, 45.49 (2C), 38.36, 27.81. MS (ESI): $[M+H]^+$: 452 (1 Cl). HRMS (ESI): m/z calcd. for $C_{21}H_{27}ClN_3O_4S$ $[M+H]^+$: 452.1405, found: 452.1406.

Ethyl 1-(9-chloro-6-methyl-7,7-dioxido-2,3,6,12-tetrahydro[1,4]benzodioxino[6,7-*c*]benzo[*f*][1,2]thiazepin-12-yl)piperidine-4-carboxylate (21n). To the solution of ethyl piperidine-4-carboxylate (1.04 g, 6.6 mmol) in CH_3CN (10 mL) crude **19** (1.16 g, 3 mmol) was added. The thick suspension obtained was diluted with CH_3CN (5 mL) and stirred at room temperature for 1 h. Water (70 mL) was added dropwise, the product separated was filtered, washed with water (2 × 5 mL), air-dried and purified by dry-column chromatography on a short silica gel column (thickness of stationary phase: 25 mm, eluent: DCM, DCM/MeOH 100:1,

100:2). After evaporation of solvents, the solid residue was recrystallized from CH_3CN to afford **21n** (1.175 g, 77%) as colorless crystals. M. p. 229–231 °C (CH_3CN). IR (KBr): 2949, 1730, 1508, 1318, 1141, 1068 cm^{-1}. ^1H NMR (500 MHz, $CDCl_3$): 7.94 (d, ^4J = 2.2 Hz, 1H), 7.35 (dd, ^4J = 2.3 Hz, ^3J = 8.3 Hz, 1H), 7.22 (d, ^3J = 8.3 Hz, 1H), 6.98 (s, 1H), 6.74 (s, 1H), 4.24 (m, 2H), 4.21 (m, 2H), 4.11 (q, ^3J = 7.1 Hz, 2H), 3.95 (s, 1H), 3.49 (s, 3H), 2.64 (m, 1H), 2.47 (m, 1H), 2.26 (m, 1H), 1.88 (m, 2H), 1.81 (m, 2H), 1.64 (m, 2H), 1.23 (t, ^3J = 7.1 Hz, 3H). ^{13}C NMR (125 MHz, $CDCl_3$): 174.79, 144.00, 143.47, 143.16, 135.09, 134.41, 133.61, 133.47, 131.02, 130.29, 128.73, 119.65, 119.00, 76.70, 64.29, 64.15, 60.35, 52.23, 52.01, 40.73, 40.41, 28.35, 27.94, 14.15. MS (ESI): $[M+H]^+$: 507 (1 Cl). HRMS (ESI): m/z calcd. for $C_{24}H_{28}ClN_2O_6S$ $[M+H]^+$: 507.1351, found: 507.1350.

1-(9-Chloro-6-methyl-7,7-dioxido-2,3,6,12-tetrahydro[1,4]benzodioxino[6,7-*c*]benzo[*f*][1,2]thiazepin-12-yl)piperidine-4-carboxamide (21o). To the solution of piperidine-4-carboxamide (isonipecotamide, 1.41 g, 11 mmol) in CH_3CN (175 mL) crude **19** (1.93 g, 5 mmol) was added and the suspension obtained was stirred at room temperature for 1.5 h. Water (350 mL) was added dropwise in 30 min, the product separated was filtered, washed with water (2 × 15 mL), air-dried and purified by dry-column chromatography on a short silica gel column (thickness of stationary phase: 25 mm, eluent: DCM, DCM/MeOH 100:3). After evaporation of solvents, the TLC pure solid residue was triturated with CH_3CN (15 mL) to afford **21o** (2.02 g, 84%) as colorless crystals. M. p. 272–275 °C (CH_3CN, decomp.) IR (KBr): 3437, 3159, 2944, 1685, 1506, 1319, 1065 cm^{-1}. ^1H NMR (500 MHz, DMSO-d_6): 7.77 (d, ^4J = 2.3 Hz, 1H), 7.64 (dd, ^4J = 2.2 Hz, ^3J = 8.3 Hz, 1H), 7.53 (d, ^3J = 8.6 Hz, 1H), 7.21 (br s, 1H), 7.03 (s, 1H), 6.96 (s, 1H), 6.73 (br s, 1H), 4.25 (m, 4H), 4.24 (s, 1H), 3.43 (s, 3H), 2.54 (m, 1H), 2.31 (m, 1H), 2.04 (m, 1H), 1.83 (m, 2H), 1.61 (m, 2H), 1.44 (m, 2H). ^{13}C NMR (125 MHz, DMSO-d_6): 176.54, 143.76, 143.39, 143.37, 135.12, 134.99, 133.86, 133.66, 131.43, 129.97, 127.42, 119.42, 119.16, 74.61, 64.29, 64.23, 52.08, 51.78, 41.58, 40.28, 28.80, 28.33. MS (ESI): $[M+H]^+$: 478 (1 Cl). HRMS (ESI): m/z calcd. for $C_{22}H_{25}ClN_3O_5S$ $[M+H]^+$: 478.1198, found: 478.1199.

1-(9-Chloro-6-methyl-7,7-dioxido-2,3,6,12-tetrahydro[1,4]benzodioxino[6,7-*c*]benzo[*f*][1,2]thiazepin-12-yl)piperidine-4-carbonitrile (21p). To the solution of piperidine-4-carbonitrile (4-cyanopiperidine) (0.99 g, 9.0 mmol) in CH_3CN (15 mL) crude **19** (1.55 g, 4 mmol) was added. The suspension obtained was stirred at room temperature for 2.5 h. Water (70 mL) was added dropwise, the product separated was filtered, washed with water (2 × 5 mL), air-dried and purified by dry-column chromatography on a short silica gel column (thickness of stationary phase: 30 mm, eluent: DCM, DCM/MeOH 100:1, 100:2). After

evaporation of solvents, the residue was triturated with CH_3CN, filtered and dried in vacuo (0.1 mm Hg, at 90 °C for 24 h) to afford **21p** (1.60 g, 87%) as colorless crystals. M. p. 229–232 °C (CH_3CN, decomp.). IR (KBr): 2928, 2240, 1508, 1320, 1143, 1067 cm^{-1}. ^1H NMR (500 MHz, $CDCl_3$): 7.94 (d, ^4J = 2.2 Hz, 1H), 7.37 (dd, ^4J = 2.2 Hz, ^3J = 8.3 Hz, 1H), 7.24 (d, ^3J = 8.3 Hz, 1H), 6.98 (s, 1H), 6.75 (s, 1H), 4.22 (m, 4H), 4.01 (s, 1H), 3.46 (s, 3H), 2.65 (m, 1H), 2.47 (m, 1H), 2.37 (m, 1H), 2.24 (m, 1H), 2.13 (m, 1H), 1.80 (m, 4H). ^{13}C NMR (125 MHz, $CDCl_3$): 144.15, 143.54, 143.08, 135.29, 133.81, 133.51, 132.87, 131.17, 130.13, 128.73, 121.29, 119.51, 119.06, 76.34, 64.25, 64.12, 50.30 (br), 40.10, 28.61, 25.81. MS (ESI): $[M+H]^+$: 460 (1 Cl). HRMS (ESI): m/z calcd. for $C_{22}H_{23}ClN_3O_4S$ $[M+H]^+$: 460.1092, found: 460.1096.

9-Chloro-12-methoxy-6-methyl-2,3,6,12-tetrahydro[1,4]benzodioxino[6,7-*c*]benzo[*f*][1,2]thiazepine 7,7-dioxide (23a). The suspension of crude **19** (1.74 g, 4.5 mmol) in MeOH (15 mL) was stirred at room temperature for 2 h, while it was transformed to the suspension of the product. It was filtered, washed with MeOH (2 × 2 mL) and subjected to dry-column chromatography on a short silica gel column (thickness of stationary phase: 25 mm, eluent: heptane/DCM 1:1, DCM). After evaporation of solvents, the solid residue was triturated with MeOH to afford **23a** (1.45 g, 84%) as colorless crystals. M. p. 218–220 °C (CH_3CN). IR (KBr): 1507, 1322, 1068, 596 cm^{-1}. ^1H NMR (500 MHz, $CDCl_3$): 7.91 (d, ^4J = 2.1 Hz, 1H), 7.58 (d, ^3J = 8.4 Hz, 1H), 7.44 (dd, ^4J = 2.2 Hz, ^3J = 8.5 Hz, 1H), 6.99 (s, 1H), 6.91 (s, 1H), 5.49 (s, 1H), 4.23 (m, 4H), 3.46 (s, 3H), 3.40 (m, 3H). ^{13}C NMR (125 MHz, $CDCl_3$): 143.93, 143.73, 140.42, 135.84, 134.57, 132.88, 131.87, 129.87, 128.53, 128.00, 117.53, 115.36, 80.51, 64.31, 64.19, 57.85, 38.00. MS (EI): M^+ = 381 (1 Cl). HRMS (ESI): m/z calcd. for $C_{17}H_{16}ClNNaO_5S$ $[M+Na]^+$: 404.0330, found: 404.0331.

2-[(9-Chloro-6-methyl-7,7-dioxido-2,3,6,12-tetrahydro[1,4]benzodioxino[6,7-*c*]benzo[*f*][1,2]thiazepin-12-yl)oxy]-*N*,*N*-dimethylethanamine (23b). To the solution of 2-(dimethylamino)ethanol (0.89 g, 10 mmol) in CH_3CN (15 mL) crude **19** (1.70 g, 4.4 mmol) was added, and the solution obtained was stirred at room temperature for 3 h. Water (60 mL) was added dropwise, the precipitate was collected by filtration, washed with water (2 × 3 mL), air-dried and purified by dry-column chromatography on a short aluminum oxide column (thickness of stationary phase: 30 mm, eluent: heptane/DCM 1:1, 1:2, DCM, DCM/MeOH 100:1, 100:2). After evaporation of solvents, the solid residue was recrystallized from CH_3CN (14 mL) to afford **23b** (1.14 g, 59%) as colorless crystals. M. p. 175–178 °C (decomp.). IR (KBr): 1508, 1323, 1147, 1060 cm^{-1}. ^1H NMR (500 MHz, $CDCl_3$): 7.89 (d, ^4J = 2.2 Hz, 1H), 7.63 (d, ^3J = 8.4 Hz, 1H), 7.43 (dd, ^4J = 2.2 Hz, ^3J = 8.4 Hz, 1H), 6.98 (s, 1H), 6.94 (s, 1H), 4.23 (m, 4H), 3.68 (m, 1H), 3.62 (m, 1H), 3.41 (s, 3H), 2.61 (m, 2H), 2.28 (s, 6H). ^{13}C NMR

(125 MHz, $CDCl_3$): 143.83, 143.73, 140.29, 136.22, 134.41, 133.37, 131.87, 129.69, 128.33, 127.94, 117.33, 115.15, 78.67, 68.60, 64.29, 64.18, 58.93, 46.02 (2 Me), 37.86. MS (ESI): $[M+H]^+$ = 439 (1 Cl). HRMS (ESI): m/z calcd. for $C_{20}H_{24}ClN_2O_5S$ $[M+H]^+$: 439.1089, found: 439.1091.

9-Chloro-6-methyl-12-(2-pyrrolidin-1-ylethoxy)-2,3,6,12-tetrahydro[1,4]benzodioxino[6,7-*c*]benzo[*f*][1,2]thiazepine 7,7-dioxide (23c). To the solution of 1-(2-hydroxyethyl)pyrrolidine (2.30 g, 20 mmol) in CH_3CN (30 mL) crude **19** (3.40 g, 8.8 mmol) was added, and the solution obtained was stirred at room temperature for 2.5 h. Water (120 mL) was added dropwise, the oily product separated was extracted with DCM (2 × 50 mL). The combined organic phases were washed with water (50 mL), dried over Na_2SO_4, and evaporated in vacuo. The oily residue was purified by dry-column chromatography on a short silica gel column (thickness of stationary phase: 30 mm, eluent: heptane/DCM 1:1, 1:2, DCM, DCM/MeOH 100:1, 100:2, 100:4). After evaporation of solvents, the solid residue was triturated with EtOH to afford **23c** (1.56 g, 38%). M. p. 145–146 °C (CH_3CN, colorless crystals). IR (KBr): 2946, 1509, 1325, 1160, 1060 cm^{-1}. ^1H NMR (500 MHz, $CDCl_3$): 7.89 (d, ^4J = 2.2 Hz, 1H), 7.64 (d, ^3J = 8.5 Hz, 1H), 7.43 (dd, ^4J = 2.2 Hz, ^3J = 8.4 Hz, 1H), 6.97 (s, 1H), 6.95 (s, 1H), 4.22 (s, 4H), 3.71 (m, 1H), 3.65 (m, 1H), 3.40 (s, 3H), 2.79 (t, ^3J = 6.2 Hz, 2H), 2.55 (m, 4H), 1.77 (m, 4H). ^{13}C NMR (125 MHz, $CDCl_3$): 143.81, 143.70, 140.29, 136.23, 134.38, 133.33, 131.84, 129.69, 128.39, 127.91, 117.30, 115.22, 78.70, 69.53, 64.27, 64.17, 55.51, 54.72 (2C), 37.89, 23.47 (2C). MS (ESI): $[M+H]^+$ = 465 (1 Cl). HRMS (ESI): m/z calcd. for $C_{22}H_{26}ClN_2O_5S$ $[M+H]^+$: 465.1245, found: 465.1249.

9-Chloro-6-methyl-12-(2-piperidin-1-ylethoxy)-2,3,6,12-tetrahydro[1,4]benzodioxino[6,7-*c*]benzo[*f*][1,2]thiazepine 7,7-dioxide (23d). To the solution of 1-(2-hydroxyethyl)piperidine (1.29 g, 10 mmol) in CH_3CN (15 mL) crude **19** (1.70 g, 4.4 mmol) was added, and the solution obtained was stirred at room temperature for 2 h. Water (60 mL) was added dropwise, the oily product separated was extracted with DCM (2 × 30 mL). The combined organic phases were washed with water (30 mL), dried over Na_2SO_4, and evaporated in vacuo. The oily residue was purified by dry-column chromatography on a short aluminum oxide column (thickness of stationary phase: 30 mm, eluent: heptane/DCM 2:1, 1:1, DCM, DCM/MeOH 100:1). After evaporation of solvents the residue was triturated with CH_3CN to afford **23d** (1.40 g, 66%) as colorless crystals. M. p. 133–135 °C (CH_3CN). IR (KBr): 1494, 1319, 1144, 1066 cm^{-1}. ^1H NMR (500 MHz, $CDCl_3$): 7.89 (d, ^4J = 2.2 Hz, 1H), 7.66 (d, ^3J = 8.6 Hz, 1H), 7.43 (dd, ^4J = 2.1 Hz, ^3J = 8.4 Hz, 1H), 6.97 (s, 1H), 6.96 (s, 1H), 5.69 (s, 1H), 4.22 (s, 4H), 3.69 (m, 1H), 3.64 (m, 1H), 3.40 (s, 3H), 2.63 (m, 2H), 2.42 (m, 4H), 1.57 (m,

4H), 1.43 (m, 2H). ^{13}C NMR (125 MHz, $CDCl_3$): 143.79, 143.67, 140.29, 136.25, 134.35, 133.28, 131.82, 129.74, 128.53, 127.86, 117.27, 115.29, 78.70, 68.17, 64.26, 64.16, 58.52, 55.08 (2C), 37.97, 25.95 (2C), 24.16. MS (ESI): $[M+H]^+ = 479$ (1 Cl). HRMS (ESI): m/z calcd. for $C_{23}H_{28}ClN_2O_5S$ $[M+H]^+$: 479.1402, found: 479.1403.

9-Chloro-6-methyl-12-(2-morpholin-4-ylethoxy)-2,3,6,12-tetrahydro[1,4]benzodioxino[6,7-*c*]benzo[*f*][1,2]thiazepine 7,7-dioxide (23e). To the solution of 4-(2-hydroxyethyl)morpholine (1.15 g, 8.8 mmol) in CH_3CN (15 mL) crude **19** (1.55 g, 4 mmol) was added and the solution obtained was stirred at room temperature for 2 h. Water (60 mL) was added dropwise, the product separated was filtered, washed with water (2 × 3 mL), air-dried and purified by dry-column chromatography on a short silica gel column (thickness of stationary phase: 30 mm, eluent: heptane/DCM 1:1, 1:2, DCM, DCM/MeOH 100:1, 100:2). After evaporation of solvents the residue was triturated with CH_3CN to afford **23e** (1.61 g, 84%) as colorless crystals. M. p. 191–194 °C (CH_3CN, decomp.). IR (KBr): 1510, 1325, 1146, 1066 cm^{-1}. ^{1}H NMR (500 MHz, $CDCl_3$): 7.89 (d, ^4J = 2.1 Hz, 1H), 7.65 (d, ^3J = 8.4 Hz, 1H), 7.44 (dd, ^4J = 2.0 Hz, ^3J = 8.4 Hz, 1H), 6.98 (s, 1H), 6.97 (s, 1H), 5.70 (s, 1H), 4.22 (s, 4H), 3.71 (m, 1H), 3.70 (m, 4H), 3.65 (m, 1H), 3.40 (s, 3H), 2.68 (t, ^3J = 5.7 Hz, 2H), 2.50 (m, 4H). ^{13}C NMR (125 MHz, $CDCl_3$): 143.82, 143.66, 140.18, 136.15, 134.39, 133.05, 131.85, 129.73, 128.25, 127.90, 117.19, 115.11, 78.55, 67.74, 66.86 (2C), 64.25, 64.15, 58.18, 54.06 (2C), 37.91. MS (ESI): $[M+H]^+ = 481$ (1 Cl). HRMS (ESI): m/z calcd. for $C_{22}H_{26}ClN_2O_6S$ $[M+H]^+$: 481.1195, found: 481.1200.

9-Chloro-6-methyl-12-[(2-morpholin-4-ylethyl)sulfanyl]-2,3,6,12-tetrahydro[1,4]benzodioxino[6,7-*c*]benzo[*f*][1,2]thiazepine 7,7-dioxide (24). To the solution of 2-(morpholin-4-yl)ethanethiol (1.30 g, 8.8 mmol) in CH_3CN (15 mL) crude **19** (1.55 g, 4 mmol) was added and it was stirred at room temperature for 1 h. Water (60 mL) was added dropwise, the product separated was filtered, washed with water (2 × 3 mL), and air-dried to give crude **24**. It was purified by dry-column chromatography on a short silica gel column (thickness of stationary phase: 30 mm, eluent: DCM, DCM/MeOH 100:2). After evaporation of solvents, the solid residue was triturated with CH_3CN (10 mL) and filtered to afford crude **24** (1.93 g), which was recrystallized from CH_3CN to give **24** (1.44 g, 72%) as colorless crystals. M. p. decomp. from 240 °C (CH_3CN). IR (KBr): 1511, 1326, 1301, 1114, 1063 cm^{-1}. ^{1}H NMR (500 MHz, $CDCl_3$): 7.94 (d, ^3J = 8.2 Hz, 1H), 7.40 (dd, ^4J = 2.2 Hz, ^3J = 8.3 Hz, 1H), 7.31 (d, ^3J = 8.3 Hz, 1H), 6.99 (s, 1H), 6.75 (s, 1H), 4.95 (s, 1H), 4.25 (m, 4H), 3.69 (m, 4H), 3.51 (s, 3H), 2.60 (m, 1H), 2.50 (m, 2H), 2.46 (m, 1H), 2.40 (m, 4H). ^{13}C NMR (125 MHz, $CDCl_3$): 144.32, 143.05, 142.31, 134.63, 134.55, 132.50, 131.89, 131.72, 131.44, 127.79, 118.10, 117.90, 66.82 (2C), 64.33,

64.26, 58.22, 54.34, 53.54 (2C), 39.35, 30.28. MS (ESI): $[M+H]^+$ = 497 (1 Cl). HRMS (ESI): m/z calcd. for $C_{22}H_{26}ClN_2O_5S_2$ $[M+H]^+$: 497.0966, found: 497.0970.

9-Chloro-2,3-dihydro[1,4]benzodioxino[6,7-*c*]benzo[*f*][1,2]thiazepin-12(6*H*)-one 7,7-dioxide (28). Compound **7** (14.89 g, 40.7 mmol) was mixed with pyridine hydrochloride (47 g, 407 mol) and the mixture was melted. The melt was stirred at 180 °C for 27 h. Water (230 mL) was added to the cooled reaction mixture (dark brown melt), the suspension obtained was stirred for 2 h, the product was filtered and washed thoroughly with water. The wet crude product on the sintered glass filter was treated with aqueous NaOH solution (80 + 30 mL, 116 g of 5 m/m % solution, containing 5.8 g, ca. 145 mmol NaOH). The filtrate was acidified with AcOH (10 mL, 173 mmol), the product precipitated was collected by filtration, washed with water (3 × 30 mL), air-dried and purified by dry-column flash chromatography on a short silica gel column (thickness of stationary phase: 35 mm, eluent: $CHCl_3$, $CHCl_3$/MeOH 100:3) After evaporation of the solvents the solid residue was triturated with CH_3CN (40 mL) to afford **28** (6.27 g, 51%) as yellow solid. M. p. 276–278 °C (CH_3CN, bright yellow crystals). IR (KBr): 3146, 1600, 1503, 1289 cm^{-1}. ^{1}H NMR (500 MHz, DMSO-d_6): 11.72 (br s, 1H), 7.90 (dd, ^4J = 2.1 Hz, ^3J = 8.2 Hz, 1H), 7.88 (dd, ^4J = 0.4 Hz, ^4J = 2.1 Hz, 1H), 7.83 (dd, ^4J = 0.5 Hz, ^3J = 8.2 Hz, 1H), 7.51 (s, 1H), 6.70 (s, 1H), 4.36 (m, 2H), 4.29 (m, 2H). ^{13}C NMR (125 MHz, DMSO-d_6): 189.20, 149.53, 140.53, 139.65, 137.14, 134.46, 133.51, 132.97, 132.40, 122.01, 120.37, 118.36, 108.75, 65.18, 64.07. MS (EI): M^+ = 351 (1 Cl). HRMS (ESI): m/z calcd. for $C_{15}H_{11}ClNO_5S$ $[M+H]^+$: 352.0041, found: 352.0046.

6-(2-Bromoethyl)-9-chloro-2,3,6,12-tetrahydro[1,4]benzodioxino[6,7-*c*]benzo[*f*][1,2]thiazepin-12-ol 7,7-dioxide (30). To the suspension of **29** (9.175 g, 20 mmol) in a mixture of THF (20 mL) and EtOH (20 mL), $NaBH_4$ (0.91 g, 24 mmol) was added in portions and the stirring was continued at room temperature for 30 min. Water (200 mL) was added, and the oily product separated was extracted with DCM (2 × 100 mL). The combined organic phases were washed with water (50 mL), dried over Na_2SO_4, and evaporated in vacuo to give crude **30** (9.09 g, 99%) as colorless foam suitable for next reaction step. IR (KBr): 3484, 1499, 1327, 1304, 1141, 1065 cm^{-1}. ^{1}H NMR (600 MHz, $CDCl_3$): 7.89 (d, ^4J = 2.2 Hz, 1H), 7.65 (d, ^3J = 8.4 Hz, 1H), 7.49 (dd, ^4J = 2.1 Hz, ^3J = 8.4 Hz, 1H), 7.04 (s, 1H), 6.94 (s, 1H), 6.07 (d, ^3J = 6.1 Hz, 1H), 4.25 (s, 4H), 4.03 (m, 2H), 3.54 (m, 1H), 3.47 (m, 1H), 3.38 (d, ^3J = 6.4 Hz, 1H). ^{13}C NMR (150 MHz, $CDCl_3$): 143.99, 143.97, 139.94, 136.91, 134.70, 133.42, 132.65, 129.22, 128.05, 127.92, 117.26, 116.74, 72.29, 64.31, 64.26, 52.83, 29.10. COSY: 7.89–7.49–7.65, 6.07–3.38, 4.03–(3.54, 3.47). HSQC (140 Hz): 7.89–127.92, 7.65–129.22, 7.49–132.65, 7.04–116.74, 6.94–117.26, 6.07–72.29, 4.25–64.31, 4.25–64.26, 4.03–52.83,

3.54–29.10, 3.47–29.10. HMBC (8 Hz, 140 Hz): 7.89–(136.91, 134.70, 132.65), 7.65–(139.94, 134.70, 72.29), 7.49–(136.91, 134.70, 127.92), 7.04–(143.97, 128.05, 72.29), 6.94–(143.99, 133.42), 6.07–(139.94, 136.91, 133.42, 129.22, 128.05, 116.74), 4.25–(143.99, 64.26), 4.25–(143.97, 64.31), 4.03–(128.05, 29.10), (3.54, 3.47)–52.83, 3.38–(136.91, 133.42, 72.29). MS (EI): M^+ = 459 (1 Br, 1 Cl). HRMS (ESI): m/z calcd. for $C_{17}H_{15}BrClNNaO_5S$ $[M+Na]^+$: 481.9435, found: 481.9438.

6-(2-Bromoethyl)-9,12-dichloro-2,3,6,12-tetrahydro[1,4]benzodioxino[6,7-*c*]benzo[*f*][1,2]thiazepine 7,7-dioxide (31). To a vigorously stirred yellow solution of **30** (8.75 g, 19 mmol) in DCM (100 mL), $SOCl_2$ (4.52 g, 38 mmol, 2.76 mL) was added dropwise at room temperature and stirring was continued for 2.5 h. To the solution hexane (400 mL) was added dropwise, the product precipitated was filtered, washed with hexane (2 × 20 mL), and dried in vacuo to give **31** (6.02 g, 66%) as beige crystals. M. p. decomp. from 144 °C. The mother liquor of this product was evaporated in vacuo, the solid residue was triturated with a little cold Et_2O to give a second crop of crude **31** (2.21 g, 24%) as beige crystals. Total yield: 8.23 g, 90%. IR (KBr): 1510, 1332, 1303, 1160, 1064 cm^{-1}. ^1H NMR (400 MHz, $CDCl_3$): 7.96 (d, ^4J = 2.1 Hz, 1H), 7.48 (dd, ^4J = 2.1 Hz, ^3J = 8.4 Hz, 1H), 7.44 (d, ^3J = 8.4 Hz, 1H), 7.10 (s, 1H), 6.92 (s, 1H), 6.04 (s, 1H), 4.33 (m, 1H), 4.26 (m, 4H), 4.01 (m, 1H), 3.73 (t, ^3J = 7.4 Hz, 2H). ^{13}C NMR (100 MHz, $CDCl_3$): 145.25, 143.97, 141.40, 136.19, 133.33, 132.89, 132.60, 130.58, 130.34, 128.20, 119.97, 118.87, 64.31, 64.29, 63.08, 54.27, 29.60. HRMS (TOF MS EI): m/z calcd. for $[C_{17}H_{14}BrCl_2NO_4S]^+$: 476.9204, found: 476.9200.

***S*-[2-(9-Chloro-12-hydroxy-7,7-dioxido-2,3-dihydro[1,4]benzodioxino[6,7-*c*]benzo[*f*][1,2]thiazepin-6(12*H*)-yl)ethyl] ethanethioate (32).** To a solution of **30** (1.015 g, 2.2 mmol) in CH_3CN (10 mL), potassium thioacetate (0.43 g, 3.75 mmol, 1.7 equiv) and tetrabutylammonium bromide (0.032 g, 0.1 mmol, 4.5 mol%) were added, and the thin suspension was vigorously stirred at room temperature for 4 h. To the thin suspension obtained, water (50 mL) and EtOAc (25 mL) were added with stirring (25 min). The phases were separated, the aqueous phase was washed with EtOAc (10 mL), the combined organic phases were washed with water (15 mL), dried over Na_2SO_4, and evaporated in vacuo. The residue was subjected to dry-column flash chromatography on a short silica gel column (thickness of stationary phase: 30 mm, eluent: heptane/DCM 1:1, 1:2, DCM, DCM/MeOH 100:1, 100:2). After evaporation of the solvents, the residue was dried in vacuo to afford **32** (0.84 g, 84%) as colorless foam. IR (KBr): 3479, 1692, 1500, 1306, 1155, 1139, 1064 cm^{-1}. ^1H NMR (600 MHz, $CDCl_3$): 7.92 (d, ^4J = 1.9 Hz, 1H), 7.64 (d, ^3J = 8.3 Hz, 1H), 7.51 (dd, ^4J = 1.9 Hz, ^3J = 8.3 Hz, 1H), 7.05 (s, 1H), 6.98 (s, 1H), 5.96 (d, ^3J = 7.3 Hz, 1H), 4.26 (s, 4H), 3.77 (t, ^3J = 7.3 Hz, 2H),

3.54 (d, ^3J = 7.3 Hz, 1H), 3.03 (m, 2H), 2.30 (s, 3H). ^{13}C NMR (150 MHz, $CDCl_3$): 195.08, 144.10, 143.67, 140.01, 136.61, 134.70, 132.70, 132.08, 129.76, 128.68, 127.94, 117.39, 116.81, 73.23, 64.32 (2C), 50.75, 30.58, 28.07. COSY: 7.64–7.51, 5.96–3.54, 3.77–3.03. HSQC (140 Hz): 7.92–127.94, 7.64–129.76, 7.51–132.70, 7.05–117.39, 6.98–116.81, 5.96–73.23, 4.26–64.32, 3.77–50.75, 3.03–28.07, 2.30–30.58. HMBC (140 Hz, 8 Hz): 7.92–(136.61, 134.70, 132.70), 7.64–(140.01, 134.70, 73.23), 7.51–(136.61, 127.94), 7.05–(144.10, 128.68, 73.23), 6.98–(143.67, 132.08), 5.96–(140.01, 136.61, 132.08, 129.76, 128.68, 117.39), 4.26–(143.67, 140.01, 64.32), 3.77–(128.68, 28.07), 3.54–(136.61, 132.08, 73.23), 3.03–(195.08, 50.75), 2.30–195.08. HRMS (ESI): m/z calcd. for $C_{19}H_{18}ClNNaO_6S_2$ $[M+Na]^+$: 478.0156, found: 478.0158.

9-Chloro-2,3-dihydro-12*H*-12,6-(epithioethano)[1,4]benzodioxino[6,7-*c*]benzo[*f*][1,2]thiazepine 7,7-dioxide (26). To the solution of compound **32** (0.64 g, 1.4 mmol) in MeOH (12 mL), K_2CO_3 (0.31 g, 2.24 mmol) was added, and the reaction mixture was stirred under argon atmosphere at room temperature for 30 min. The solution obtained was diluted with water (30 mL), and aqueous HCl solution (5 m/m%, 4.2 mL) was added dropwise. The thiol intermediate separated was dissolved by adding DCM (40 mL), the layers were separated, and the aqueous layer was washed with DCM (10 mL). The combined organic phases were washed with water (15 mL), dried over Na_2SO_4 and evaporated in vacuo to give crude thiol intermediate as a dense colorless oil. It was dissolved in DCM (100 mL), *p*-TsOH·H_2O (0.057 g, 0.3 mmol, 21 mol %) was added and the reaction mixture was stirred under argon atmosphere at room temperature for 1 h. Then it was evaporated to 30 mL of volume and purified by passing it through a short silica gel column (thickness of stationary phase: 30 mm, eluent: DCM). After evaporation of the solvent, the partly crystalline residue was triturated with DIPE (5 mL) to afford **26** (0.504 g, 91%) as colorless crystals. M. p. 258–259.5 °C (CH_3CN). IR (KBr): 3449, 1506, 1338, 1304, 1159, 1062, 595 cm^{-1}. ^{1}H NMR (600 MHz, $CDCl_3$): 7.90 (d, ^4J = 2.2 Hz, 1H), 7.46 (dd, ^4J = 2.2 Hz, ^3J = 8.4 Hz, 1H), 7.32 (d, ^3J = 8.4 Hz, 1H), 6.99 (s, 1H), 6.78 (s, 1H), 4.78 (s, 1H), 4.47 (dt, ^3J = 14.6 Hz, ^3J = 3.0 Hz, 1H), 4.24 (m, 4H), 3.50 (~t, ^3J = 14.6 Hz, 1H), 3.29 (~t, ^3J = 15.3 Hz, 1H), 2.59 (dt, ^3J = 15.3 Hz, ^4J = 2.9 Hz, 1H). ^{13}C NMR (150 MHz, $CDCl_3$): 143.93, 143.65, 143.57, 137.04, 134.65, 133.07, 132.81, 132.15, 131.20, 127.24, 120.19, 116.13, 64.34, 64.15, 51.70, 51.52, 27.79. COSY: 7.46–7.32, (4.47, 3.50)–(3.29, 2.59). HSQC (140 Hz): 7.90–127.24, 7.46–132.81, 7.32–132.15, 6.99–120.19, 6.78–116.13, 4.78–51.52, (4.47, 3.50)–51.70, 4.24–64.34/64.15, (3.29, 2.59)–27.79. HMBC (140 Hz, 8 Hz): 7.90–(134.65, 132.81), 7.46–(134.65, 127.24), 7.32–(143.65, 133.07, 51.52), 6.99–(143.93, 137.04, 132.10), 6.78–(143.57, 131.20, 51.52), 4.78–(143.65, 137.04, 131.20,

116.13, 27.79), (4.47, 3.50)–(131.20, 27.79), 4.24–(143.93, 143.57), (3.29, 2.59)–(51.70, 51.52). HRMS (ESI): m/z calcd. for $C_{17}H_{15}ClNO_4S_2$ $[M+H]^+$: 396.0126, found: 396.0128.

9-Chloro-2,3,6,12-tetrahydro[1,4]benzodioxino[6,7-*c*]benzo[*f*][1,2]thiazepin-12-ol 7,7-dioxide (33). To a solution of **28** (9.74 g, 27.7 mmol) in an aqueous NaOH solution (5.54 g, 138.5 mmol NaOH dissolved in 250 mL of water), $NaBH_4$ (5.24 g, 138.5 mmol) was added in several portions while cooling the reaction mixture with tap water. After stirring at room temperature for 69 h (weekend), the yellow solution was cooled with ice-water, and an aqueous HCl solution (6 M, 60 mL) was added dropwise over a period of 30 min. The oily product separated was dissolved by adding DCM (300 mL), the phases were separated, the organic phase was dried over Na_2SO_4 and purified by passing through a short silica gel column (thickness of stationary phase: 35 mm, eluent: DCM, DCM/MeOH 100:1). After evaporation of the solvents, the residue (10.9 g of greyish foam) was triturated with CH_3CN to afford **33** (8.18 g, 83%) as pale pink crystals. M. p. decomp. from 180 °C. IR (KBr): 3488, 3238, 1509, 1322, 1138, 1066 cm^{-1}. ^1H NMR (500 MHz, DMSO-d_6): 10.37 (br s, 1H), 7.87 (m, 1H), 7.71 (m, 2H), 7.10 (s, 1H), 6.70 (s, 1H), 6.66 (br s, 1H), 6.49 (s, 1H), 4.21 (m, 4H). ^{13}C NMR (125 MHz, DMSO-d_6): 142.72, 142.26, 139.71, 139.41, 132.61, 132.42, 132.07, 127.44, 125.58, 125.27, 115.06, 113.64, 65.80, 64.32, 64.31. MS (ESI): $[M-H]^-$ = 352 (1 Cl). HRMS (ESI): m/z calcd. for $C_{15}H_{12}ClNNaO_5S$ $[M+Na]^+$: 376.0017, found: 376.0020.

9,12-Dichloro-2,3,6,12-tetrahydro[1,4]benzodioxino[6,7-*c*]benzo[*f*][1,2]thiazepine 7,7-dioxide (34). To a vigorously stirred suspension of **33** (2.12 g, 6 mmol) in DCM (15 mL), $SOCl_2$ (0.95 g, 8 mmol, 0.58 mL) was added at room temperature. After 5 min of stirring the starting material was completely dissolved, and the product began to crystallize. Stirring was continued for 2 h, hexane (80 mL) was added dropwise to the suspension. The product was filtered, washed with hexane, and dried in vacuo to give crude **34** (2.23 g, 100%) as beige crystals. M. p. decomp. from 105 °C. IR (KBr): 3263, 1512, 1328, 1161, 1061 cm^{-1}. ^1H NMR (500 MHz, $CDCl_3$): 7.91 (d, ^4J = 2.2 Hz, 1H), 7.46 (dd, ^4J = 2.2 Hz, ^3J = 8.3 Hz, 1H), 7.36 (d, ^3J = 8.4 Hz, 1H), 7.23 (br s, 1H), 7.08 (s, 1H), 6.88 (s, 1H), 5.86 (s, 1H), 4.25 (m, 4H). ^{13}C NMR (125 MHz, $CDCl_3$): 145.13, 143.80, 141.92, 136.82, 133.38, 132.49, 131.79, 130.76, 129.06, 126.21, 121.30, 117.65, 64.30, 64.24, 63.27. MS (ESI): $[M-Cl+OH-H]^-$ = 352 (1 Cl) (after a rapid hydrolysis of **34** the compound **33** was detected).

12-(2-Bromoethoxy)-9-chloro-2,3,6,12-tetrahydro[1,4]benzodioxino[6,7-*c*]benzo[*f*][1,2]thiazepine 7,7-dioxide (35). To a stirred solution of 2-bromoethanol (4.50 g, 36 mmol, 2.56 mL) in CH_3CN (15 mL), **34** (2.23 g, 6 mmol) was added in portions at 20 °C. The thin suspension obtained was stirred at room temperature for 6 h, then it was evaporated in

vacuo. The oily residue was purified by dry-column flash chromatography on a short silica gel column (thickness of stationary phase: 30 mm, eluent: heptane/DCM 1:1, 1:2, DCM). After evaporation of the solvents the residue was triturated with Et_2O (5 mL), filtered and dried in vacuo to give **35** (1.69 g, 61%) as off-white crystals. M. p. decomp. from 180 °C. IR (KBr): 3210, 1498, 1320, 1158, 1064 cm^{-1}. ^1H NMR (500 MHz, $CDCl_3$): 7.89 (d, J^4J = 2.2 Hz, 1H), 7.67 (br s, 1H), 7.43 (dd, J_1 =2.2 Hz, ^3J = 8.3 Hz, 1H), 7.37 (d, ^3J = 8.3 Hz, 1H), 7.01 (s, 1H), 6.83 (s, 1H), 5.29 (br s, 1H), 4.22 (m, 4H), 3.84 (m, 1H), 3.79 (m, 1H), 3.50 (m, 2H). ^{13}C NMR (125 MHz, $CDCl_3$): 144.24, 143.55, 142.13, 136.24, 133.77, 132.02, 131.12, 130.59, 129.03, 125.84, 120.05, 116.90, 83.67, 69.27, 64.31, 64.19, 30.51. MS (ESI): $[M-H]^-$ = 458 (1 Br, 1 Cl). HRMS (ESI): m/z calcd. for $C_{17}H_{15}BrClNNaO_5S$ $[M+Na]^+$: 481.9435, found: 481.9431.

9-Chloro-2,3-dihydro-12*H*-12,6-(epoxyethano)[1,4]benzodioxino[6,7-*c*]benzo[*f*][1,2]thiazepine 7,7-dioxide (27). To a solution of **35** (1.475 g, 3.2 mmol) in CH_3CN (40 mL), K_2CO_3 (1.38 g, 10 mmol) was added, and the suspension obtained was stirred at room temperature for 5 h. Water (200 mL) was added dropwise. The product precipitated was filtered, washed with water (2 × 5 mL), air-dried and purified by dry-column flash chromatography on a short aluminum oxide column (thickness of stationary phase: 30 mm, eluent: hexane/DCM 1:1, 1:2, DCM). After evaporation of the solvents the residue was recrystallized from CH_3CN (12 mL) to afford **27** (0.86 g, 71%) as colorless crystals. M. p. 228–229 °C (CH_3CN). IR (KBr): 1506, 1338, 1164, 1061, 612 cm^{-1}. ^1H NMR (500 MHz, $CDCl_3$): 7.89 (d, ^4J = 2.1 Hz, 1H), 7.47 (dd, ^4J = 2.2 Hz, ^3J = 8.3 Hz, 1H), 7.33 (d, ^3J = 8.2 Hz, 1H), 7.03 (s, 1H), 6.81 (s, 1H), 5.48 (s, 1H), 4.23 (m, 2H), 4.21 (m, 2H), 4.18 (m, 1H), 4.10 (m, 1H), 3.74 (m, 1H), 3.56 (m, 1H). ^{13}C NMR (125 MHz, $CDCl_3$): 144.71, 144.18, 144.03, 136.35, 134.31, 133.07, 132.47, 131.44, 130.43, 127.94, 119.70, 117.28, 83.18, 64.29, 64.17, 61.88, 52.62. NOE: 3.74–(7.03, 6.81, 5.48, 4.10, 3.56), 3.56–(7.03, 6.81, 5.48, 4.18, 3.74). COSY: 7.89–7.47–7.33, 4.23–4.21, (4.18, 3.56)–(4.10, 3.74). HSQC (140 Hz): 7.89–127.94, 7.47–132.47, 7.33–133.09, 7.03–119.70, 6.81–117.28, 5.48–83.18, 4.23–64.17, 4.21–64.29, 4.18–52.62, 4.10–61.88, 3.74–61.88, 3.56–52.62. HMQC (140 Hz, 8 Hz): 7.89–(136.35, 132.47, 131.44), 7.47–(136.35, 131.44, 127.94), 7.33–(144.71, 136.35, 83.18), 7.03–(144.03, 134.31, 130.43), 6.81–(144.18, 130.43, 119.70, 83.18), 5.48–(144.71, 134.31, 130.43, 117.28, 61.88), 4.23–(144.18, 64.29), 4.21–(144.03, 64.17), 4.18–(130.43, 83.18), 4.10–83.18, 3.74–83.18, 3.56–61.88. MS (ESI): $[M+H]^+$ = 380 (1 Cl). HRMS (ESI): m/z calcd. for $C_{17}H_{15}ClNO_5S$ $[M+H]^+$: 380.0354, found: 380.0354.

10-Chloro-7-methyl-2,3,7,13-tetrahydro[1,4]benzodioxino[6,5-*c*]benzo[*f*][1,2]thiazepin-13-ol 8,8-dioxide (36). To a suspension of **8** (2.23 g, 6.1 mmol) in DMF (15 mL) and EtOH (15 mL), $NaBH_4$ (0.46 g, 12.2 mmol) was added, and the reaction mixture was stirred at room

temperature for 3 h. To the solution obtained water (150 mL) was added dropwise. The separated product was filtered, washed with water, and dried in vacuo to give **36** (2.00 g, 89%) as colorless crystals. The quality of the rigorously dried compound was suitable for the next reaction step. A sample of **36** (0.65 g) was purified by dry-column chromatography on a short silica gel column (thickness of stationary phase: 20 mm, eluent: DCM, DCM/MeOH 100:1). After evaporation of solvents, the oily residue was triturated with DIPE, filtered and dried in vacuo to afford **36** (0.585 g, 84%) as colorless crystals. M. p. decomp. from 155 °C. IR (KBr): 3544, 1490, 1346, 847, 589 cm^{-1}. ^1H NMR (500 MHz, DMSO-d_6): 7.77 (d, ^4J = 2.2 Hz, 1H), 7.68 (dd, ^4J = 2.3 Hz, ^3J = 8.3 Hz, 1H), 7.61 (d, ^3J = 8.3 Hz, 1H), 6.99 (d, ^3J = 8.5 Hz, 1H), 6.92 (d, ^3J = 8.5 Hz, 1H), 6.22 (d, ^3J = 4.3 Hz, 1H), 6.14 (d, ^3J = 4.2 Hz, 1H), 4.33 (m, 2H), 4.26 (m, 2H), 3.41 (s, 3H). ^{13}C NMR (125 MHz, DMSO-d_6): 143.50, 142.88, 140.77, 136.68, 134.35, 133.90, 132.16, 131.50, 130.18, 126.93, 122.62, 117.62, 66.79, 64.51, 63.89, 40.72. MS (EI): $[M]^+$ = 367 (1 Cl). HRMS (ESI): m/z calcd. for $C_{16}H_{14}ClNNaO_5S$ $[M+Na]^+$: 390.0173, found: 390.0179.

10,13-Dichloro-7-methyl-2,3,7,13-tetrahydro[1,4]benzodioxino[6,5-*c*]benzo[*f*][1,2]thiazepine 8,8-dioxide (37). To a vigorously stirred suspension of **36** (2.39 g, 6.5 mmol) in DCM (25 mL), $SOCl_2$ (0.93 g, 7.8 mmol, 0.57 mL) was added dropwise at room temperature. The solution obtained was stirred at room temperature for 1.5 h. To the suspension obtained hexane (75 mL) was added, the product was filtered, washed with hexane (2 × 8 mL) and dried in vacuo to give crude **37** (2.36 g, 94%) as colorless crystals. M. p. decomp. from 90 °C. The product was used without purification for the next reaction step. IR (KBr): 1497, 1337, 1161, 1061, 854, 577 cm^{-1}. ^1H NMR (500 MHz, $CDCl_3$): 7.97 (m, 1H), 7.47 (m, 2H), 7.00 (d, ^3J = 8.7 Hz, 1H), 6.96 (d, ^3J = 8.7 Hz, 1H), 6.72 (s, 1H), 4.20–4.40 (m, 4H), 3.54 (s, 3H). ^{13}C NMR (125 MHz, $CDCl_3$): 143.45, 142.21, 140.50, 136.13, 133.43, 133.20, 132.73, 132.25, 127.87, 126.13, 121.52, 119.34, 64.64, 63.79, 53.59, 39.44. MS (EI): $[M]^+$ = 385 (2 Cl). HRMS (TOF MS EI): m/z calcd. for $[C_{16}H_{13}Cl_2NO_4S]^+$: 384.9942, found: 384.9940.

10-Chloro-7-methyl-13-pyrrolidin-1-yl-2,3,7,13-tetrahydro[1,4]benzodioxino[6,5-*c*]benzo[*f*][1,2]thiazepine 8,8-dioxide (38a). To the cooled solution of pyrrolidine (0.106 g, 1.5 mmol) in CH_3CN (2 mL) **37** (0.1785 g, 0.46 mmol) was added. The solution obtained was stirred at room temperature for 1 h. Water (10 mL) was added dropwise, the product separated was filtered, washed with water (2 × 1 mL), air-dried and purified by dry-column chromatography on a short silica gel column (thickness of stationary phase: 20 mm, eluent: DCM, DCM/MeOH 100:2). After evaporation of solvents, the residue (0.19 g of colorless oil) was triturated with DIPE (2 mL) to afford **38a** (0.153 g, 79%) as colorless crystals. M. p.

decomp. from 213 °C (CH_3CN, bright colorless crystals). IR (KBr): 1491, 1324, 1154, 1109, 1067 cm^{-1}. ^1H NMR (500 MHz, $CDCl_3$): 7.92 (m, 1H), 7.36 (m, 2H), 6.95 (d, ^3J = 8.6 Hz, 1H), 6.84 (d, ^3J = 8.7 Hz, 1H), 4.87 (s, 1H), 4.26 (m, 2H), 4.25 (m, 1H), 4.20 (m, 1H), 3.49 (s, 3H), 2.27 (m, 2H), 2.20 (m, 2H), 1.67 (m, 4H). ^{13}C NMR (125 MHz, $CDCl_3$): 143.80, 143.56, 141.29, 135.17, 134.87, 133.62, 131.40, 130.91, 129.94, 128.12, 122.94, 117.69, 64.74, 64.20, 63.91, 53.24 (2C), 40.07, 23.17 (2C). MS (ESI): $[M+H]^+$ = 421 (1 Cl). HRMS (ESI): m/z calcd. for $C_{20}H_{22}ClN_2O_4S$ $[M+H]^+$: 421.0983, found: 421.0985.

10-Chloro-7-methyl-*N*-(2-morpholin-4-ylethyl)-2,3,7,13-tetrahydro[1,4]benzodioxino[6,5-*c*]benzo[*f*][1,2]thiazepin-13-amine 8,8-dioxide (38b). To the solution of 4-(2-aminoethyl)morpholine (0.664 g, 5.1 mmol) in CH_3CN (10 mL) **37** (0.608 g, 1.57 mmol) was added. The solution obtained was stirred at room temperature for 1 h. Water (50 mL) was added dropwise in 30 min, the product separated was filtered, washed with water (2 × 5 mL), air-dried and purified by dry-column chromatography on a short silica gel column (thickness of stationary phase: 25 mm, eluent: DCM, DCM/MeOH 100:2). After evaporation of solvents, the residue was triturated with CH_3CN to afford **38b** (0.588 g, 78%) as colorless crystals. M. p. 203–205 °C (CH_3CN). IR (KBr): 3350, 1493, 1313, 1119, 1062 cm^{-1}. ^1H NMR (600 MHz, $CDCl_3$): 7.95 (d, ^4J = 2.2 Hz, 1H), 7.42 (dd, ^4J = 2.2 Hz, ^3J = 8.2 Hz, 1H), 6.96 (d, ^3J = 8.8 Hz, 1H), 6.87 (d, ^3J = 8.7 Hz, 1H), 5.25 (s, 1H), 4.28 (m, 2H), 4.25 (m, 1H), 4.22 (m, 1H), 3.63 (m, 4H), 3.49 (s, 3H), 2.80 (br s, 1H), 2.60 (m, 2H), 2.50 (m, 1H), 2.35 (m, 1H), 2.24 (m, 4H). ^{13}C NMR (150 MHz, $CDCl_3$): 143.32, 142.17, 140.80, 136.04, 134.53, 133.22, 131.56, 131.48, 128.69, 128.20, 120.32, 117.77, 66.81 (2C), 64.37, 63.83, 57.90 (2C), 53.44 (2C), 43.54, 38.51. COSY: 7.95–7.42–7.33, 6.96–6.87, 4.28–(4.25, 4.22), 3.63–2.24, 2.60–(2.50, 2.35). HSQC (140 Hz): 7.95–128.20, 7.42–131.48, 7.33–133.22, 6.96–120.32, 6.87–117.77, 5.25–57.90, 4.28–64.37, 4.25–63.83, 4.22–63.83, 3.63–66.81, 3.49–38.51, 2.60–43.54, 2.50–57.90, 2.35–57.90, 2.24–53.44. HMBC (8 Hz, 140 Hz): 7.95–(136.04, 134.53, 131.48), 7.42–(136.04, 134.53, 128.20), 7.33–(142.17, 134.53, 57.90), 6.96–(143.32, 128.69), 6.87–(140.83, 131.56), 5.25–(142.17, 140.83, 136.04, 133.22, 131.56, 128.69, 43.54), 4.28–(140.83, 63.83), (4.25, 4.22)–(143.32, 64.37), 3.63–66.81, 3.49–131.56, 2.60–57.90, (2.50, 2.35)–(53.44, 43.54), 2.24–53.44. MS (EI): $[M]^+$ = 479 (1 Cl). HRMS (ESI): m/z calcd. for $C_{22}H_{27}ClN_3O_5S$ $[M+H]^+$: 480.1354, found: 480.1357.

Ethyl 7-[(10-chloro-7-methyl-8,8-dioxido-2,3,7,13-tetrahydro[1,4]benzodioxino[6,5-*c*]benzo[*f*][1,2]thiazepin-13-yl)amino]heptanoate (38c). To a solution of ethyl 7-aminoheptanoate (1.73 g, 10 mmol) in CH_3CN (15 mL) **37** (1.425 g, 3.7 mmol) was added, the solution obtained was stirred at room temperature for 1 h. The solution was evaporated in vacuo,

the oily residue was dissolved in DCM (40 mL), washed with water (2 × 20 mL), dried over Na_2SO_4, and evaporated in vacuo. The oily residue was purified by dry-column flash chromatography on a short silica gel column (thickness of stationary phase: 30 mm, eluent: heptane/DCM 2:1, 1:1, DCM, DCM/MeOH 100:1). After evaporation of solvents in vacuo **38c** was obtained (1.63 g, 84%) as dense colorless oil which slowly solidified when stored at 5 °C. A small sample (0.20 g) was triturated with a little EtOH to afford **38c** (0.13 g) as colorless crystals. M. p. 81–83 °C. IR (KBr): 3326, 2928, 1726, 1493, 1322, 1146 cm^{-1}. ^1H NMR (500 MHz, $CDCl_3$): 7.94 (d, ^4J = 2.2 Hz, 1H), 7.43 (dd, ^4J = 2.2 Hz, ^3J = 8.3 Hz, 1H), 7.36 (d, ^3J = 8.3 Hz, 1H), 6.93 (d, ^3J = 8.7 Hz, 1H), 6.86 (d, ^3J = 8.7 Hz, 1H), 5.30 (s, 1H), 4.19-4.33 (m, 4H), 4.11 (q, ^3J = 7.2 Hz, 2H), 3.40 (s, 3H), 2.46 (t, ^3J = 7.1 Hz, 2H), 2.25 (t, ^3J = 7.5 Hz, 2H), 2.02 (br s, 1H), 1.58 (m, 2H), 1.44 (m, 2H), 1.27 (m, 4H), 1.24 (t, ^3J = 7.1 Hz, 3H). ^{13}C NMR (125 MHz, $CDCl_3$): 173.65, 143.18, 141.91, 141.24, 136.13, 134.41, 133.17, 131.78, 131.67, 128.22, 128.13, 120.44, 117.67, 64.32, 63.83, 60.11, 58.21, 47.79, 38.72, 34.17, 29.75, 28.89, 26.88, 24.77, 14.19. MS (ESI): $[M+H]^+$ = 523 (1 Cl). HRMS (ESI): m/z calcd. for $C_{25}H_{32}ClN_2O_6S$ $[M+H]^+$: 523.1664, found: 523.1671.

7-[(10-Chloro-7-methyl-8,8-dioxido-2,3,7,13-tetrahydro[1,4]benzodioxino[6,5-*c*]benzo[*f*][1,2]thiazepin-13-yl)amino]heptanoic acid (38d). To a solution of ester **38c** (1.41 g, 2.7 mmol) in EtOH (20 mL), NaOH (0.130 g, 3.25 mmol) dissolved in water (5 mL) was added at room temperature and the reaction mixture (gradually diluting suspension, later solution) was stirred at room temperature for 19 h (overnight stirring). Water (15 mL) was added, and the reaction mixture was partly evaporated in vacuo to remove ethanol. The solution obtained was neutralized with aqueous HCl solution (0.27 mL of cc. HCl dissolved in 2.7 mL of water, containing 3.25 mmol of HCl). It was stirred at room temperature for 4 h, the crystalline product obtained was filtered, washed with water (2 × 3 mL), and dried in vacuo to give **38d** (0.804, 60%) as an off-white amorphous solid. Melting range (glass-transition range): 76–85 °C. The aqueous mother liquor was extracted with EtOAc (2 × 20 mL), the combined organic phases were dried over Na_2SO_4, evaporated in vacuo, the residue was triturated with water (2 mL), the wet product was dried in vacuo to give a second crop of **38d** (0.30 g, 22%) as an off-white amorphous solid. Melting range (glass-transition range): 76–85 °C. Total yield: 1.10 g, 82%. IR (KBr): 2932, 1713, 1493, 1330, 1109, 1067 cm^{-1}. ^1H NMR (500 MHz, $CDCl_3$): 7.93 (d, ^4J = 1.5 Hz, 1H), 7.42 (m, 2H), 6.93 (d, ^3J = 8.8 Hz, 1H), 6.88 (d, ^3J = 8.7 Hz, 1H), 6.76 (br s, 2H), 5.43 (s, 1H), 4.28 (m, 3H), 4.23 (m, 1H), 3.38 (s, 3H), 2.50 (m, 2H), 2.18 (t, ^3J = 7.3 Hz, 2H), 1.52 (m, 2H), 1.45 (m, 2H), 1.24 (m, 4H). ^{13}C NMR (125 MHz, $CDCl_3$): 177.84, 143.14, 141.78, 141.31, 134.93, 134.62, 133.69, 131.89, 131.84, 128.10, 126.14,

119.69, 118.08, 64.39, 63.85, 57.33, 47.14, 38.08, 34.44, 28.93, 28.84, 26.83, 24.83. MS (ESI): $[M+H]^+$ = 495 (1 Cl). HRMS (ESI): m/z calcd. for $C_{23}H_{28}ClN_2O_6S$ $[M+H]^+$: 495.1351, found: 495.1355.

Methyl 4-chloro-2-(2,3-dihydro-1,4-benzodioxin-5-ylsulfamoyl)benzoate (39). To a vigorously stirred solution of 2,3-dihydro-1,4-benzodioxin-5-amine (11.50 g, 76 mmol) and *N*,*N*-diethylaniline (11.34 g, 76 mmol, 12.1 mL) in MeOH (100 mL), methyl 4-chloro-2-chlorosulfonylbenzoate (**9**, 19.38 g, 72 mmol) was added over a period of 10 min while cooling the reaction mixture with tap water. The suspension formed was stirred at room temperature for 1 h, then at 0–5 °C for 1 h. The crystalline product was filtered and purified by dry-column flash chromatography on a short silica gel column (thickness of stationary phase: 30 mm, eluent: heptane/DCM 1:1, DCM). After evaporation of solvents, the solid residue was triturated with MeOH, filtered and air-dried to afford **39** (22.76 g, 82%) as colorless crystals. M. p. 134–136 °C (MeOH, bright colorless crystals). IR (KBr): 3288, 1727, 1300, 1169, 1087 cm^{-1}. ^1H NMR (500 MHz, $CDCl_3$): 8.30 (br s, 1H), 7.91 (d, ^4J = 2.2 Hz, 1H), 7.81 (d, ^3J = 8.3 Hz, 1H), 7.54 (dd, ^4J = 2.2 Hz, ^3J = 8.3 Hz, 1H), 7.15 (dd, ^4J = 1.5 Hz, ^3J = 8.2 Hz, 1H), 6.79 (~t, ^3J = 8.2 Hz, 1H), 6.66 (dd, ^4J = 1.5 Hz, ^3J = 8.3 Hz, 1H), 4.10 (m, 2H), 4.01 (s, 3H), 4.00 (m, 2H). ^{13}C NMR (125 MHz, $CDCl_3$): 166.37, 143.50, 140.97, 137.88, 135.52, 132.28, 132.10, 129.89, 128.53, 125.26, 120.82, 115.60, 114.30, 64.28, 63.90, 53.39. MS (ESI): $[M-H]^-$ = 382 (1 Cl). HRMS (ESI): m/z calcd. for $C_{16}H_{15}ClNO_6S$ $[M+H]^+$: 384.0303, found: 384.0299.

Methyl 4-chloro-2-[2,3-dihydro-1,4-benzodioxin-5-yl(methyl)sulfamoyl]benzoate (40). To a solution of compound **39** (22.26 g, 58 mmol) in DMF (100 mL), anhydrous K_2CO_3 (10.42 g, 75.4 mmol) was added, and the suspension was stirred at room temperature for 15 min. To the vigorously stirred suspension iodomethane (10.70 g, 75.4 mmol, 4.7 mL) was added dropwise while cooling the reaction mixture with tap water. It was stirred at room temperature for 2 h, then water (500 mL) was added. The oily product separated, the supernatant was decanted, the oil was dissolved in DCM (100 mL), the solution was washed with water (2 × 80 mL), dried over Na_2SO_4 and diluted with heptane (125 mL). Kieselgel 60 H (for TLC, 5–40 µm, 7 g) was added and the mixture was stirred for half an hour. The adsorbent was removed by filtration, and the filtrate was evaporated in vacuo. The residue was triturated with MeOH (50 mL), the product was filtered, washed with MeOH and air-dried to afford **40** (20.00 g, 87%) as bright colorless crystals. M. p. 103–105 °C (MeOH). IR (KBr): 1745, 1477, 1350, 1283, 1117, 1079 cm^{-1}. ^1H NMR (500 MHz, $CDCl_3$): 7.73 (d, ^4J = 2.0 Hz, 1H), 7.53 (dd, ^4J = 2.1 Hz, ^3J = 8.2 Hz, 1H), 7.41 (d, ^3J = 8.2 Hz, 1H), 6.90 (dd, ^4J = 1.7 Hz, ^3J = 7.7 Hz, 1H), 6.88 (dd, ^4J = 1.7 Hz, ^3J = 8.2 Hz, 1H), 6.82 (~t, ^3J = 8.0 Hz, 1H), 4.14 (m, 2H), 3.92 (m, 2H), 3.77 (s, 3H), 3.29 (s,

3H). ^{13}C NMR (125 MHz, $CDCl_3$): 167.48, 144.37, 141.08, 139.11, 135.63, 131.80, 131.51, 129.34, 129.15, 128.32, 123.71, 120.44, 117.65, 64.05, 63.81, 53.06, 38.21. MS (EI): $[M]^+$ = 397 (1 Cl). HRMS (ESI): m/z calcd. for $C_{17}H_{17}ClNO_6S$ $[M+H]^+$: 398.0460, found: 398.0461.

4-Chloro-2-[2,3-dihydro-1,4-benzodioxin-5-yl(methyl)sulfamoyl]benzoic acid (41). A solution of NaOH (5.48 g, 137 mmol) in water (100 mL) was added to the suspension of compound **40** (21.80 g, 54.8 mmol) in MeOH (100 mL) and the mixture was refluxed for 1 h. The solution thus obtained was diluted with water (600 mL), aqueous HCl solution (5 m/m %, 100 mL, 105 g, 144 mmol HCl) was added while cooling the reaction mixture with tap water. The sticky oily product separated from the reaction mixture (pH 1) slowly crystallized while stirring at room temperature for 1 h. It was filtered, washed with water (3 × 60 mL), and dried over P_2O_5 to afford crude **41** (20.45 g, 97%) as off-white crystals of suitable quality for next reaction step. M. p. 188–191 °C (CH_3CN, decomp.). IR (KBr): 2883, 1707, 1353, 1303, 1169, 1080, 1060, 979 cm^{-1}. ^1H NMR (500 MHz, DMSO-d_6): 13.57 (br s, 1H), 7.80 (dd, ^4J = 2.2 Hz, ^3J = 8.2 Hz, 1H), 7.64 (d, ^3J = 8.2 Hz, 1H), 7.52 (d, ^4J = 2.0 Hz, 1H), 6.90 (dd, ^4J = 1.8 Hz, ^3J = 8.1 Hz, 1H), 6.83 (~t, ^3J = 8.0 Hz, 1H), 6.79 (dd, ^4J = 2.0 Hz, ^3J = 8.1 Hz, 1H), 4.15 (m, 2H), 3.93 (m, 2H), 3.20 (s, 3H). ^{13}C NMR (125 MHz, DMSO-$d6$): 168.21, 144.50, 141.21, 137.75, 133.97, 133.44, 132.77, 129.92, 128.49, 128.35, 123.24, 120.39, 117.60, 64.23, 63.83, 38.17. MS (ESI): $[M-H]^-$ = 382 (1 Cl). HRMS (ESI): m/z calcd. for $C_{16}H_{15}ClNO_6S$ $[M+H]^+$: 384.0303, found: 384.0302.

10-Chloro-13-methyl-2,3-dihydro[1,4]benzodioxino[5,6-*c*]benzo[*f*][1,2]thiazepin-7(13*H*)-one 12,12-dioxide (42). To the thin suspension of **41** (19.80 g, 51.6 mmol) in DCM (165 mL), PCl_5 (12.91 g, 62 mmol, 1.2 equiv) was added in two portions (effervescence!), and the solution obtained was refluxed for 9 h. The solution was cooled to 0–5 °C, and $SnCl_4$ (29.60 g, 113.5 mmol, 13.3 mL, 2.2 equiv) was added. The suspension obtained was stirred at 0–5 °C for 1.5 h, then it was allowed to warm to room temperature (approx. 1 h). It was diluted with DCM (165 mL), poured onto a mixture of crushed ice (600 g) and conc. hydrochloric acid (85 mL, 100 g, 1 mol of HCl). The crystalline product separated was filtered, washed with diluted hydrochloric acid (5%, 2 × 50 mL) and water (2 × 50 mL), and air-dried to give crude **42** (10.93 g of off-white solid). The two-phase filtrate was separated, the strongly acidic aqueous phase was washed with DCM (100 mL), the combined organic phases were washed with aqueous HCl solution (5 m/m %, 100 mL), dried over Na_2SO_4, evaporated in vacuo, and the residue was triturated with DIPE to afford a second crop of crude **42** (6.88 g of pale pink solid). The two crops combined were purified by dry-column flash chromatography on a short silica gel column (thickness of the stationary phase: 30 mm, eluent: DCM). After evaporation of the

solvent, the residue was triturated with CH_3CN (50 mL) to give pure **42** (16.28 g, 86%) as colorless crystals. M. p. 265–267 °C (CH_3CN/EtOH 1:1). IR (KBr): 1643, 1601, 1578, 1354, 1278, 1170, 1078, 996 cm^{-1}. ^{1}H NMR (500 MHz, $CDCl_3$+CD_3OD): 8.01 (d, ^{3}J = 8.4 Hz, 1H), 7.98 (d, ^{4}J = 2.1 Hz, 1H), 7.89 (d, ^{3}J = 9.0 Hz, 1H), 7.70 (dd, ^{4}J = 2.1 Hz, ^{3}J = 8.3 Hz, 1H), 6.98 (d, ^{3}J = 9.0 Hz, 1H), 4.41 (m, 2H), 4.39 (m, 2H), 3.10 (s, 3H). ^{13}C NMR (125 MHz, $CDCl_3$+CD_3OD): 187.60, 149.34, 138.61, 138.50, 137.56, 134.08, 133.85, 133.11, 130.50, 126.43, 125.94, 124.37, 116.27, 64.33, 64.10, 37.74. HSQC (140 Hz): 8.01–133.85, 7.98–126.43, 7.89–124.37, 7.70–133.11, 6.98–116.27, 4.41–64.10, 4.39–64.33, 3.10–37.74. HMQC (140 Hz, 8 Hz): 8.01–(187.60, 138.50, 137.56), 7.98–(138.50, 133.11), 7.89–(187.60, 149.34, 130.50), 7.70–(138.50, 134.08, 126.43), 6.98–(149.34, 138.61, 125.94), 4.41–(138.61, 64.33), 4.39–(149.34, 64.10), 3.10–130.50. MS (EI): $[M]^+$ = 365 (1 Cl). HRMS (ESI): m/z calcd. for $C_{16}H_{13}ClNO_5S$ $[M+H]^+$: 366.0197, found: 366.0200.

10-Chloro-13-methyl-2,3,7,13-tetrahydro[1,4]benzodioxino[5,6-*c*]benzo[*f*][1,2]thiazepin-7-ol 12,12-dioxide (43). To a suspension of **42** (5.49 g, 15 mmol) in DMF (30 mL) and EtOH (30 mL), $NaBH_4$ (1.135 g, 30 mmol) was added, and the reaction mixture was stirred at room temperature for 4.5 h. Water (300 mL) was added dropwise over 30 min. The separated product was filtered, washed with water and Et_2O, and air-dried to give TLC pure crude **43** (5.32 g, 96%) as colorless crystals. M. p. decomp. from 208 °C (CH_3CN/EtOH 1:1). IR (KBr): 3484, 1493, 1304, 1070, 984 cm^{-1}. This compound is a 6:4 mixture of two conformers in DMSO-d_6 solution at 25 °C. ^{1}H NMR (500 MHz, DMSO-d_6): 7.92 (m, 0.6H), 7.80–7.65 (m, 2.2H), 7.05–6.98 (d, 1H), 6.96–6.86 (m, 1H), 6.57 (br s, 0.6H), 6.24 (br s, 0.4H), 6.18 (br s, 0.6H), 5.73 (br s, 0.4H), 4.40–4.20 (m, 4H), 3.40–3.20 (m, 3H, overlapped with water signal of DMSO-d_6). ^{13}C NMR (125 MHz, DMSO-d_6): 144.49, 143.49, 141.62, 140.83, 140.00, 138.14, 137.42, 134.06, 133.83, 133.45, 132.53, 132.29, 131.98, 127.06, 126.65 (126.60, shoulder), 122.57, 121.16, 117.54, 116.68, 115.94, 75.41, 65.97, 64.58, 64.08, 40.30, 40.13, 39.96, 39.79, 38.90, 36.02. MS (EI): $[M]^+$: 367 (1 Cl). HRMS (ESI): m/z calcd. for $C_{16}H_{14}ClNNaO_5S$ $[M+Na]^+$: 390.0173, found: 390.0170.

7,10-Dichloro-13-methyl-2,3,7,13-tetrahydro[1,4]benzodioxino[5,6-*c*]benzo[*f*][1,2]thiazepine 12,12-dioxide (44). To a vigorously stirred suspension of **43** (5.74 g, 15.6 mmol) in DCM (250 mL), $SOCl_2$ (7.43 g, 62.4 mmol, 4.5 mL, 4 equiv) was added dropwise at room temperature. The suspension was stirred at room temperature for 2 h (no solution was obtained). To the suspension hexane (500 mL) was added, the product was filtered, washed with hexane (2 × 30 mL) and dried in vacuo to give crude **44** (5.65 g, 94%). M. p. decomp. from 250 °C. The compound is of suitable quality for the next step. IR (KBr): 1504,

1477, 1336, 1319, 1160, 670 cm^{-1}. No NMR spectra of **44** could be recorded due to the extremely low solubility of this compound in the usual NMR solvents and their mixtures as well.

10-Chloro-*N*,13-dimethyl-2,3,7,13-tetrahydro[1,4]benzodioxino[5,6-*c*]benzo[*f*][1,2]thiazepin-7-amine 12,12-dioxide (45a). To the cold solution of methylamine (0.62 g, 20 mmol) in dioxane (15 mL) crude **44** (1.545 g, 4 mmol) was added, and the suspension obtained was stirred at 5–10 °C for 2 h and at room temperature for 1 h. To the thin suspension water (50 mL) was added dropwise, the product precipitated was filtered, washed with water (2 × 5 mL), air-dried and purified by dry-column flash chromatography on a short silica gel column (thickness of stationary phase: 25 mm, eluent: DCM, DCM/MeOH 100:1, 100:2). After evaporation of solvents, the solid residue was triturated with CH_3CN (5 mL) to afford **45a** (1.30 g, 85%) as colorless crystals. M. p. 187–189 °C (CH_3CN). IR (KBr): 3323, 1498, 1327, 1157, 1070 cm^{-1}. This compound is a 16:9 mixture of two conformers in DMSO-d_6 solution at 25 °C. At elevated temperature (352 K) the signals of the two species coalesced. ^{1}H NMR (500 MHz, $CDCl_3$): 7.77–7.64 (m, 2.2H), 7.58 (d, ^3J = 8.4 Hz, 0.64H), 6.93–6.86 (m, 2H), 5.12 (d, ^3J = 8.4 Hz, 0.35H), 4.67 (s, 0.63H), 4.40–4.20 (m, 4H), 3.37 (s, 1.9H), 3.24 (s, 1.06H), 3.01 (m, 0.4H), 2.33 (d, ^3J = 4.4 Hz, 1.1H), 2.28 (br s, 0.64H), 2.09 (br s, 2H). ^{13}C NMR (125 MHz, $CDCl_3$): (33 signals) 144.42, 143.28, 143.20, 142.03, 141.41, 140.39, 138.77, 136.87, 134.65, 133.52, 133.31, 132.27, 132.11, 131.72, 128.81, 127.36, 126.51, 126.13, 125.01, 122.58, 117.81, 117.23, 116.79, 70.22, 64.53, 64.51, 64.07, 64.03, 60.40, 38.41, 36.49, 34.90, 34.59. MS (ESI): [M+H]$^+$ = 381 (1 Cl). HRMS (ESI): m/z calcd. for $C_{17}H_{18}ClN_2O_4S$ [M+H]$^+$: 381.0670, found: 381.0668.

10-Chloro-13-methyl-7-pyrrolidin-1-yl-2,3,7,13-tetrahydro[1,4]benzodioxino[5,6-*c*]benzo[*f*][1,2]thiazepine 12,12-dioxide (45b). To the solution of pyrrolidine (0.85 g, 12 mmol, 1.0 mL) in CH_3CN (30 mL) crude **44** (1.55 g, 4 mmol) was added. The solution obtained was stirred at room temperature for 1 h. Water (150 mL) was added dropwise, the product separated was filtered, washed with water (3 × 5 mL), air-dried and purified by dry-column flash chromatography on a short silica gel column (thickness of stationary phase: 25 mm, eluent: DCM, DCM/MeOH 100:1). After evaporation of solvents, the residue was triturated with Et_2O (5 mL) to afford **45b** (1.24 g, 74%) as colorless crystals. M. p. 189–191 °C (CH_3CN). IR (KBr): 2937, 1496, 1306, 1153, 1069 cm^{-1}. ^{1}H NMR (500 MHz, $CDCl_3$): 7.94 (d, ^4J = 2.2 Hz, 1H), 7.35 (dd, ^4J = 2.2 Hz, ^3J = 8.3 Hz, 1H), 7.29 (d, ^3J = 8.3 Hz, 1H), 6.79 (d, ^3J = 8.4 Hz, 1H), 6.73 (d, ^3J = 8.6 Hz, 1H), 4.37 (m, 1H), 4.35 (m, 1H), 4.28 (m, 2H), 4.04 (s, 1H), 3.42 (s, 3H), 2.21 (m, 2H), 2.14 (m, 2H), 1.67 (m, 4H). ^{13}C NMR (125 MHz, $CDCl_3$): 144.58,

143.45, 142.35, 135.64, 134.82, 134.77, 133.05, 130.88, 128.09, 125.66, 121.29, 117.12, 76.53, 64.48, 64.11, 53.65 (2C), 38.11, 23.16 (2C). MS (ESI): $[M+H]^+$ = 421 (1 Cl). HRMS (ESI): m/z calcd. for $C_{20}H_{22}ClN_2O_4S$ $[M+H]^+$: 421.0983, found: 421.0983.

1-(10-Chloro-13-methyl-12,12-dioxido-2,3,7,13-tetrahydro[1,4]benzodioxino[5,6-*c*]benzo[*f*][1,2]thiazepin-7-yl)piperidine-4-carboxamide (45c). To the solution of piperidine-4-carboxamide (isonipecotamide, 1.13 g, 8.8 mmol) in CH_3CN (140 mL) crude **44** (1.545 g, 4 mmol) was added. The suspension obtained was stirred at room temperature for 1 h. Water (300 mL) was added dropwise, the product separated was collected by filtration, washed with water (2 × 10 mL), air-dried and purified by dry-column flash chromatography on a short silica gel column (thickness of stationary phase: 25 mm, eluent: DCM/MeOH 100:3). After evaporation of solvents, the solid residue was triturated with CH_3CN (15 mL) to afford **45c** (1.60 g, 84%) as colorless crystals. M. p. decomp. from 272 °C (CH_3CN). IR (KBr): 3419, 3198, 1682, 1494, 1307, 1151 cm^{-1}. ^1H NMR (500 MHz, DMSO-d_6): 7.77 (d, ^4J = 2.2 Hz, 1H), 7.64 (dd, ^4J = 2.2 Hz, J_2 =8.3 Hz, 1H), 7.56 (d, ^3J = 8.4 Hz, 1H), 7.20 (br s, 1H), 6.88 (d, ^3J = 8.4 Hz, 1H), 6.86 (d, ^3J = 8.4 Hz, 1H), 6.72 (br s, 1H), 4.39 (m, 1H), 4.31 (m, 2H), 4.26 (s, 1H), 4.25 (m, 1H), 3.37 (s, 3H), 2.50 (m, 1H), 2.28 (m, 1H), 2.04 (m, 1H), 1.82 (m, 2H), 1.59 (m, 2H), 1.43 (m, 2H). ^{13}C NMR (125 MHz, DMSO-d_6): 176.52, 144.54, 143.44, 142.54, 135.11, 135.04, 133.95, 133.88, 131.42, 127.31, 125.04, 122.28, 117.11, 75.35, 64.52, 64.07, 52.14, 51.82, 41.57, 38.70, 28.85, 28.38. MS (ESI): $[M+H]^+$ = 478 (1 Cl). HRMS (ESI): m/z calcd. for $C_{22}H_{25}ClN_3O_5S$ $[M+H]^+$: 478.1198, found: 478.1201.

10-Chloro-7-methoxy-13-methyl-2,3,7,13-tetrahydro[1,4]benzodioxino[5,6-*c*]benzo[*f*][1,2]thiazepine 12,12-dioxide (46). To a suspension of crude **44** (0.309 g, 0.8 mmol) in DCM (3 mL) MeOH (3 mL) was added and the solution obtained in 5 min was stirred at room temperature for further 40 min. The solution was evaporated in vacuo, the residue was subjected to dry-column flash chromatography on a short silica gel column (thickness of stationary phase: 25 mm, eluent: heptane/DCM 1:2, DCM). After evaporation of solvents, the solid residue was triturated with Et_2O to afford **46** (0.233 g, 76%) as colorless crystals. M. p. decomp. from 185 °C (MeOH). IR (KBr): 2937, 1493, 1319, 1302, 1074, 982 cm^{-1}. This compound is a 6:4 mixture of two conformers in $CDCl_3$ solution at 25 °C. ^1H NMR (500 MHz, $CDCl_3$): 7.97 (br s, 0.40H), 7.87 (br s, 0.32H), 7.71 (m, 0.43H), 7.48–7.35 (m, 1.5H), 6.85 (m, 2H), 5.75 (br s, 0.43H), 5.00 (br s, 0.55H), 4.42–4.25 (m, 4H), 3.58 (br s, 1.33H), 3.47 (br s, 1.33H), 3.37 (br s, 1.67H), 3.22 (br s, 1.67H). ^{13}C NMR (125 MHz, $CDCl_3$): (31 signals) 145.32, 143.88, 142.60, 142.31, 142.20, 139.96, 137.49, 135.52, 135.30, 133.88, 133.39, 133.26, 131.74, 131.62, 131.13, 128.01, 127.89, 126.21, 125.43, 123.09, 122.14, 118.00,

117.00, 115.42, 87.33, 64.47, 64.11, 58.46, 56.80, 38.31, 35.69. MS (EI): $[M]^+$ = 381 (1 Cl). HRMS (ESI): m/z calcd. for $C_{17}H_{20}ClN_2O_5S$ $[M+NH_4]^+$: 399.0776, found: 399.0775.

Methyl 2-[(2,3-dihydro-1,4-benzodioxin-6-ylsulfonyl)amino]benzoate (48). To the solution of 2,3-dihydro-1,4-benzodioxine-6-sulfonyl chloride (**47**, 23.47 g, 100 mmol) in pyridine (100 mL) methyl anthranilate (13.61 g, 90 mmol, 11.6 mL) was added at 0—5 °C, and the reaction mixture (suspension after 10 min) was stirred at 0–5 °C for 4 h, then at room temperature for 13 h. Water (600 mL) was added dropwise, the product was filtered, washed with water (3 × 80 mL) to give crude **48** (29.78 g, 95%) as colorless crystals. M. p. 158–160 °C (MeOH). IR (KBr): 3151, 1690, 1499, 1286, 1254, 1154, 1085 cm^{-1}. ^1H NMR (500 MHz, $CDCl_3$): 10.62 (br s, 1H), 7.92 (m, 1H), 7.66 (m, 1H), 7.45 (m, 1H), 7.38 (d, ^4J = 2.2 Hz, 1H), 7.34 (dd, ^4J = 2.3 Hz, ^3J = 8.5 Hz, 1H), 7.03 (m, 1H), 6.86 (d, ^3J = 8.5 Hz, 1H), 4.26 (m, 2H), 4.23 (m, 2H), 3.88 (s, 3H). ^{13}C NMR (125 MHz, $CDCl_3$): 168.25, 147.63, 143.40, 140.48, 134.45, 131.72, 131.09, 122.65, 120.96, 118.71, 117.60, 116.86, 115.67, 64.43, 64.03, 52.41. MS (EI): $[M]^+$ = 349. HRMS (ESI): m/z calcd. for $C_{16}H_{16}NO_6S$ $[M+H]^+$: 350.0693, found: 350.0698.

Methyl 2-[(2,3-dihydro-1,4-benzodioxin-6-ylsulfonyl)(methyl)amino]benzoate (49). Method A: To a suspension of compound **48** (29.70 g, 85 mmol) in DMF (85 mL), anhydrous K_2CO_3 (15.27 g, 110.5 mmol, 1.3 equiv) was added, and the suspension was stirred at room temperature for 15 min. Iodomethane (15.68 g, 110.5 mmol, 6.9 mL, 1.3 equiv) was added dropwise while cooling the reaction mixture with tap water. The suspension was stirred at room temperature for 22 h. Water (425 mL) was added, the product precipitated was filtered, washed with water, and air-dried to afford crude **49** (30.73 g, 99%) as off-white crystals. The product was of suitable quality for the next reaction step. Method B: To the ice-cooled solution of 2,3-dihydro-1,4-benzodioxine-6-sulfonyl chloride (**47**, 2.35 g, 10 mmol) in abs. pyridine (10 mL) methyl *N*-methylanthranilate (1.65 g, 10 mmol, 1.47 mL) was added dropwise. The reaction mixture was stirred at 0–5 °C for 2 h, then at room temperature for 17 h (overnight). The suspension obtained was added to an aqueous HCl solution (5 m/m %, 100 mL). The oily product was separated, DCM (50 mL) was added, the phases were separated, the aqueous phase was washed with DCM (40 mL), the combined organic phases were washed with aqueous HCl solution (5 m/m %, 20 mL), dried over Na_2SO_4, and evaporated in vacuo. The residue was purified by dry-column flash chromatography on a short silica gel column (thickness of stationary phase: 30 mm, eluent: heptane/DCM 1:1, DCM, DCM/MeOH 100:1). After evaporation of solvents, the oily residue was triturated with MeOH to afford **49** (2.60 g, 71%) as colorless crystals. M. p. 96–98 °C (MeOH). IR (KBr): 1734, 1496, 1338, 1290, 1255, 1064

cm^{-1}. ^{1}H NMR (500 MHz, $CDCl_3$): 7.83 (dd, ^4J = 1.8 Hz, ^3J = 7.7 Hz, 1H), 7.43 (m, 1H), 7.37 (m, 1H), 7.21 (d, ^4J = 2.2 Hz, 1H), 7.11 (dd, ^4J = 2.2 Hz, ^3J = 8.5 Hz, 1H), 6.98 (dd, ^4J = 1.3 Hz, ^3J = 7.7 Hz, 1H), 6.90 (d, ^3J = 8.6 Hz, 1H), 4.31 (m, 2H), 4.29 (m, 2H), 3.88 (s, 3H), 3.25 (s, 3H). ^{13}C NMR (150 MHz, $CDCl_3$): 166.87, 147.27, 142.32, 140.15, 132.22, 132.12, 130.97, 128.57, 128.02, 121.36, 117.41, 117.16, 64.49, 64.11, 52.36, 38.79. MS (EI): $[M]^+$ = 363. HRMS (ESI): m/z calcd. for $C_{17}H_{18}NO_6S$ $[M+H]^+$: 364.0849, found: 364.0850.

2-[(2,3-Dihydro-1,4-benzodioxin-6-ylsulfonyl)(methyl)amino]benzoic acid (50). A solution of NaOH (8.46 g, 211.5 mmol) in water (140 mL) was added to the suspension of crude **49** (30.71 g, 84.5 mmol) in MeOH (140 mL) and the mixture was refluxed for 1 h. The solution thus obtained was diluted with water (700 mL), aqueous HCl solution (5 m/m %, 200 mL, 210 g, 288 mmol HCl) was added over 20 min while cooling the reaction mixture with ice water. The sticky oily product separated slowly crystallized while stirring at room temperature for 1 h. It was collected by filtration, washed with water (3 × 60 mL) and dried in vacuo over P_2O_5 to afford crude **50** (29.01 g, 95%) as colorless crystals. M. p. 171–173 °C (CH_3CN, decomp.). The quality of the dried product was suitable for the next reaction step. IR (KBr): 1679, 1496, 1346, 1290, 1062 cm^{-1}. ^{1}H NMR (600 MHz, DMSO-d_6): 12.94 (br s, 1H), 7.74 (m, 1H), 7.48 (m, 1H), 7.44 (m, 1H), 7.04 (m, 1H), 7.04 (m, 1H), 7.03 (m, 1H), 6.91 (m, 1H), 4.35 (m, 2H), 4.31 (m, 2H), 3.14 (s, 3H). ^{13}C NMR (150 MHz, DMSO-d_6): 167.62, 147.54, 143.50, 139.65, 133.66, 132.09, 130.43, 130.35, 128.35, 128.29, 121.13, 117.81, 116.44, 64.64, 64.25, 38.87. COSY: 7.74–7.44–7.48-6.91, 7.04–7.04–7.03, 4.35–4.31. HSQC (140 Hz): 7.74–130.43, 7.48–132.09, 7.44–128.35, 7.04–121.13, 7.04–117.81, 7.03–116.44, 6.91–128.29, 4.35–64.64, 4.31–64.25, 3.14–38.87. HMBC (8 Hz, 140 Hz): 7.74–(167.62, 139.65, 132.09), 7.48–(139.65, 130.43), 7.44–(133.66, 128.29), 7.04–(147.54, 116.44), 7.04–(143.50, 130.35), 7.03–(147.54, 121.13), 6.91–(133.66, 128.35), 4.35–147.54, 4.31–143.50, 3.14–139.65. MS (EI): $[M]^+$ = 349. HRMS (ESI): m/z calcd. for $C_{16}H_{16}NO_6S$ $[M+H]^+$: 350.0693, found: 350.0694.

7-Methyl-2,3-dihydrobenzo[*c*][1,4]benzodioxino[6,7-*f*][1,2]thiazepin-12(7*H*)-one 6,6-dioxide (51). To the suspension of **50** (17.47 g, 50 mmol) in DCM (250 mL), $SOCl_2$ (8.93 g, 75 mmol, 5.5 mL) was added and the reaction mixture was refluxed for 8.5 h. The solution obtained was cooled to 0–5 °C, and anhydrous $AlCl_3$ (10.00 g, 75 mmol) was added. The solution was stirred at 0–5 °C for 1.5 h, then it was refluxed for 3 h. The brown solution was poured onto a mixture of crushed ice (300 g) and diluted aqueous hydrochloric acid (5 m/m %, 100 mL). After the ice melted the phases were separated, the aqueous phase was washed with DCM (100 mL), the combined organic phases were washed with water (100 mL), dried over Na_2SO_4, and evaporated in vacuo. The residue was subjected to dry-column flash

chromatography on a short silica gel column (thickness of the stationary phase: 30 mm, eluent: heptane/DCM 2:1, 1:1, 1:2, DCM, DCM/MeOH 100:1, 100:2). After evaporation of the solvents, the solid residue was triturated with Et_2O (20 mL) to give pure **51** (4.26 g, 26%) as pale yellow crystals. M. p. 194–196 °C (CH_3CN). IR (KBr): 1648, 1350, 1299, 1159, 1066, 894 cm^{-1}. ^1H NMR (500 MHz, $CDCl_3$): 8.29 (m, 1H), 7.61 (m, 1H), 7.55 (s, 1H), 7.48 (s, 1H), 7.36 (m, 1H), 7.34 (m, 1H), 4.36 (m, 2H), 4.34 (m, 2H), 3.30 (s, 3H). ^{13}C NMR (125 MHz, $CDCl_3$): 188.81, 147.09, 146.39, 141.32, 134.49, 132.13, 131.70, 130.42, 129.60, 126.18, 125.32, 121.32, 115.48, 64.63, 64.36, 39.19. NOE: 3.30–(7.48, 7.34). COSY: 8.29–7.36–7.61–7.34, 4.36–4.34. HSQC (140 Hz): 8.29–132.13, 7.61–134.49, 7.55–121.32, 7.48–115.48, 7.36–126.18, 7.34–125.32, 4.36–64.36, 4.34–64.63, 3.30–39.19. HMQC (140 Hz, 8 Hz): 8.29–(188.81, 141.32, 134.49), 7.61–(141.32, 132.13), 7.55–(188.81, 146.39, 130.42), 7.48–(147.09, 129.60), 7.36–(131.70, 125.32), 7.34–(131.70, 126.18), 4.36–(146.39, 64.63), 4.34–(147.09, 64.36), 3.30–141.32. MS (EI): $[M]^+$ = 331. HRMS (ESI): m/z calcd. for $C_{16}H_{14}NO_5S$ $[M+H]^+$: 332.0587, found: 332.0582.

7-Methyl-2,3,7,12-tetrahydrobenzo[*c*][1,4]benzodioxino[6,7-*f*][1,2]thiazepin-12-ol 6,6-dioxide (52). To a suspension of **51** (4.10 g, 12.4 mmol) in DMF (25 mL) and abs. EtOH (25 mL), $NaBH_4$ (0.91 g, 24 mmol) was added, and the reaction mixture was stirred at room temperature for 4 h. To the solution obtained water (400 mL) was added dropwise. The product separated was filtered, washed with water, air-dried and purified by dry-column flash chromatography on a short silica gel column (thickness of stationary phase: 25 mm, eluent: DCM, DCM/MeOH 200:1). After evaporation of solvents, the residue was triturated with DIPE (30 mL) to afford **52** (3.86 g, 93%) as colorless crystals. M. p. decomp. from 180 °C. IR (KBr): 3485, 1570, 1493, 1291, 1134, 1061 cm^{-1}. ^1H NMR (500 MHz, DMSO-d_6): 7.58 (m, 1H), 7.46 (m, 1H), 7.36 (m, 1H), 7.34 (m, 1H), 7.28 (d, ^4J = 0.9 Hz, 1H), 7.19 (s, 1H), 6.44 (d, ^3J = 5.1 Hz, 1H), 6.18 (d, ^3J = 5.1 Hz, 1H), 4.30 (m, 2H), 4.27 (m, 2H), 3.32 (s, 3H). ^{13}C NMR (125 MHz, DMSO-d_6): 146.54, 143.21, 142.67, 136.92, 134.90, 129.73, 128.71, 128.04, 127.55, 125.62, 116.13, 114.37, 67.70, 64.75, 64.36, 37.07. MS (EI): $[M]^+$ = 333. HRMS (ESI): m/z calcd. for $C_{16}H_{16}NO_5S$ $[M+H]^+$: 334.0744, found: 334.0741.

12-Chloro-7-methyl-2,3,7,12-tetrahydrobenzo[*c*][1,4]benzodioxino[6,7-*f*][1,2]thiazepine 6,6-dioxide (53). To a vigorously stirred suspension of **52** (3.50 g, 10.5 mmol) in DCM (25 mL), $SOCl_2$ (1.50 g, 12.6 mmol, 0.92 mL) was added dropwise at room temperature. The solution obtained turned to a thick suspension. It was stirred at room temperature for 2 h, then hexane (50 mL) was added, the product was filtered, washed with hexane (2 × 10 mL) and dried in vacuo to give crude **53** (3.50 g, 95%) as colorless crystals. M. p. decomp. from 175 °C. The

product was suitable for the next reaction step without further purification. IR (KBr): 1499, 1325, 1299, 1134, 1064, 926, 890 cm^{-1}. ^{1}H NMR (500 MHz, $CDCl_3$): 7.51 (s, 1H), 7.50 (m, 1H), 7.47 (m, 1H), 7.39 (m, 1H), 7.32 (m, 1H), 7.03 (s, 1H), 6.06 (s, 1H), 4.29 (m, 4H), 3.54 (s, 3H). ^{13}C NMR (125 MHz, $CDCl_3$): 146.22, 144.50, 139.38, 137.89, 133.03, 131.16, 129.74, 129.21, 128.57, 128.49, 119.78, 117.25, 64.56, 64.36, 64.15, 38.90. MS (ESI): [M-Cl-OH-H]$^{-}$ = 332, [M+CH_3CN+Na]$^{+}$ = 397 (after a rapid hydrolysis of **53**, compound **52** was detected). HRMS (TOF MS EI): m/z calcd. for [$C_{16}H_{14}ClNO_4S$]$^{+}$: 351.0332, found: 351.0331.

***N*,7-Dimethyl-2,3,7,12-tetrahydrobenzo[*c*][1,4]benzodioxino[6,7-*f*][1,2]thiazepin-12-amine 6,6-dioxide (54a).** To the solution of methylamine (0.78 g, 25 mmol) in dioxane (23 mL) crude **53** (1.76 g, 5 mmol) was added, and the suspension obtained was stirred at room temperature for 1.5 h. Water (75 mL) was added dropwise, the product separated was filtered, washed with water (2 × 10 mL), air-dried and purified by dry-column flash chromatography on a short silica gel column (thickness of stationary phase: 25 mm, eluent: DCM, DCM/MeOH 100:1). After evaporation of solvents, the residue was triturated with DIPE (10 mL), filtered and recrystallized from CH_3CN (9 mL) to afford **54a** (1.29 g, 75%) as colorless crystals. M. p. 182–184 °C (CH_3CN). IR (KBr): 3362, 1573, 1494, 1287, 1066, 895, 562 cm^{-1}. ^{1}H NMR (500 MHz, $CDCl_3$): 7.51 (s, 1H), 7.39 (m, 1H), 7.32 (m, 2H), 7.26 (m, 1H), 6.96 (s, 1H), 4.78 (s, 1H), 4.29 (m, 2H), 4.27 (m, 2H), 3.27 (s, 3H), 2.35 (s, 3H), 2.30 (br s, 1H). ^{13}C NMR (125 MHz, $CDCl_3$): 146.30, 142.65, 139.19, 138.07, 131.82, 130.47, 130.41, 128.89, 127.50, 127.48, 118.72, 118.30, 67.97, 64.61, 64.20, 38.55, 34.80. MS (ESI): [M+H]$^{+}$ = 347. HRMS (ESI): m/z calcd. for $C_{17}H_{19}N_2O_4S$ [M+H]$^{+}$: 347.1060, found: 347.1060.

7-Methyl-12-pyrrolidin-1-yl-2,3,7,12-tetrahydrobenzo[*c*][1,4]benzodioxino[6,7-*f*][1,2]thiazepine 6,6-dioxide (54b). To the solution of pyrrolidine (0.78 g, 11 mmol) in CH_3CN (15 mL) crude **53** (1.76 g, 5 mmol) was added. The suspension obtained was stirred at room temperature for 1 h. Water (30 mL) was added dropwise, the product separated was filtered, washed with water (2 × 5 mL), air-dried, and purified by dry-column chromatography on a short aluminum oxide column (thickness of stationary phase: 25 mm, eluent: heptane/DCM 1:1, DCM). After evaporation of solvents, the residue was triturated with DIPE (10 mL) to afford TLC-pure **54b** (1.53 g, 79%) as colorless crystals. M. p. decomp. from 210 °C (CH_3CN). IR (KBr): 1503, 1311, 1293, 1135, 1066, 562 cm^{-1}.^{1}H NMR (500 MHz, $CDCl_3$): 7.47 (s, 1H), 7.43 (m, 1H), 7.34 (m, 1H), 7.29 (m, 1H), 7.25 (m, 1H), 6.89 (s, 1H), 4.27 (m, 1H), 4.24 (m, 1H), 4.23 (m, 2H), 4.11 (s, 1H), 3.50 (s, 3H), 2.25 (m, 4H), 1.69 (m, 4H). ^{13}C NMR (125 MHz, $CDCl_3$): 145.36, 143.45, 142.18, 138.27, 133.73, 131.56, 130.38, 129.74, 129.51, 128.40,

119.27, 117.50, 75.80, 64.59, 64.21, 53.67 (2C), 39.44, 23.26 (2C). MS (ESI): $[M+H]^+$ = 387. HRMS (ESI): m/z calcd. for $C_{20}H_{23}N_2O_4S$ $[M+H]^+$: 387.1373, found: 387.1376.

7-Methyl-*N*-(2-morpholin-4-ylethyl)-2,3,7,12-tetrahydrobenzo[*c*][1,4]benzodioxino[6,7-*f*][1,2]thiazepin-12-amine 6,6-dioxide (54c). To the solution of 4-(2-aminoethyl)morpholine (1.15 g, 8.8 mmol) in CH_3CN (15 mL) crude **53** (1.41 g, 4 mmol) was added. The suspension formed was stirred at room temperature for 1.5 h. Water (50 mL) was added dropwise, and an oily product separated. Water (20 mL) and DCM (20 mL) were added, and stirring was continued for 10 min. The phases were separated, the aqueous phase was washed with DCM (20 mL), the combined organic phase was washed with water (20 mL), dried over Na_2SO_4, and evaporated in vacuo. The residue was purified by dry-column flash chromatography first on a short silica gel column (thickness of stationary phase: 25 mm, eluent: DCM, DCM/MeOH 100:1, 100:2). After evaporation of solvents, the residue was purified again by dry-column flash chromatography on a short aluminum oxide column (thickness of stationary phase: 25 mm, eluent: heptane/DCM 1:1, 1:2, DCM, DCM/MeOH 100:1). After evaporation of solvents, the residue was crystallized from CH_3CN to afford **54c** (1.31 g, 74%) as colorless crystals. M. p. 142–144 °C (CH_3CN). IR (KBr): 3347, 1575, 1333, 1308, 1287, 1118, 1066, 896 cm^{-1}. ^1H NMR (500 MHz, $CDCl_3$): 7.49 (s, 1H), 7.36 (m, 2H), 7.32 (m, 1H), 7.25 (m, 1H), 6.97 (s, 1H), 4.92 (s, 1H), 4.28 (m, 2H), 4.25 (m, 2H), 3.67 (m, 4H), 3.36 (s, 3H), 2.73 (br s, 1H), 2.62 (m, 2H), 2.52 (m, 1H), 2.46 (m, 1H), 2.35 (m, 4H). ^{13}C NMR (125 MHz, $CDCl_3$): 146.21, 142.72, 139.51, 138.84, 132.63, 130.77, 129.33, 128.84, 127.65, 127.40, 118.06, 117.86, 66.87 (2C), 65.39, 64.60, 64.16, 58.19, 53.63 (2C), 44.11, 37.95. MS (EI): $[M]^+$ = 445. HRMS (ESI): m/z cald. for $C_{22}H_{28}N3O_5S$ $[M+H]^+$: 446.1744, found: 446.1740.

7-Methyl-*N*-(3-morpholin-4-ylpropyl)-2,3,7,12-tetrahydrobenzo[*c*][1,4]benzodioxino[6,7-*f*][1,2]thiazepin-12-amine 6,6-dioxide (54d). To the solution of 4-(3-aminopropyl)morpholine (1.27 g, 8.8 mmol) in CH_3CN (15 mL) crude **53** (1.41 g, 4 mmol) was added. The solution obtained was stirred at room temperature for 1 h, then it was evaporated in vacuo. The residue was dissolved in DCM (30 mL), the solution was washed with water (2 × 20 mL), dried over Na_2SO_4, and evaporated in vacuo. The residue was purified by dry-column flash chromatography firstly on a short silica gel column (thickness of stationary phase: 25 mm, eluent: DCM, DCM/MeOH 100:1, 100:2, 100:3). After evaporation of solvents, the residue was purified again by dry-column flash chromatography on a short aluminum oxide column (thickness of stationary phase: 25 mm, eluent: heptane/DCM 1:1, DCM, DCM/MeOH 100:1). After evaporation of solvents, the residue was triturated with CH_3CN to afford **54d** (1.51 g, 82%) as colorless crystals. M. p. 149–151 °C (CH_3CN). IR (KBr): 3354, 1574, 1493, 1333,

1060, 896, 708 cm^{-1}. ^{1}H NMR (500 MHz, CDCl3): 7.49 (s, 1H), 7.37 (m, 1H), 7.35 (m, 1H), 7.31 (m, 1H), 7.27 (m, 1H), 7.00 (m, 1H), 4.99 (s, 1H), 4.28 (m, 2H), 4.24 (m, 2H), 3.67 (m, 4H), 3.32 (s, 3H), 2.58 (m, 3H), 2.42 (m, 6H), 1.70 (~qn, ^{3}J = 6.7 Hz, 2H). ^{13}C NMR (125 MHz, $CDCl_3$): 146.32, 142.58, 139.55, 138.83, 132.92, 130.39, 129.07, 128.67, 127.53, 127.35, 118.13, 117.39, 66.88 (2C), 64.59, 64.13, 57.24, 53.71 (2C), 46.61, 37.92, 26.47. MS (ESI): $[M+H]^+$ = 460. HRMS (ESI): m/z calcd. for $C_{23}H_{30}N_3O_5S$ $[M+H]^+$: 460.1901, found: 460.1896.

Ethyl 7-[(7-methyl-6,6-dioxido-2,3,7,12-tetrahydrobenzo[*c*][1,4]benzodioxino[6,7-*f*][1,2]thiazepin-12-yl)amino]heptanoate (54e). To a solution of ethyl 7-aminoheptanoate (3.47 g, 20 mmol) in CH_3CN (25 mL) crude **53** (3.17 g, 9 mmol) was added which led to a slightly exothermic reaction. The solution obtained was stirred at room temperature for 1 h, then it was evaporated in vacuo. The residue was dissolved in DCM (50 mL), washed with water (2 × 25 mL), dried over Na_2SO_4, and partially evaporated in vacuo. The solution was purified by dry-column flash chromatography on a short aluminum oxide column (thickness of stationary phase: 40 mm, eluent: heptane/DCM 1:1, DCM). After evaporation of solvents in vacuo **54e** (4.16 g, 94%) was obtained as a pale yellow oil. IR (film): 2933, 1732, 1574, 1495, 1295, 1068, 895, 712 cm^{-1}. ^{1}H NMR (600 MHz, $CDCl_3$): 7.50 (s, 1H), 7.36 (m, 1H), 7.34 (m, 1H), 7.32 (m, 1H), 7.26 (m, 1H), 6.95 (s, 1H), 4.91 (s, 1H), 4.29 (m, 2H), 4.27 (m, 2H), 4.11 (q, ^{3}J = 7.1 Hz, 2H), 3.31 (s, 3H), 2.48 (m, 2H), 2.27 (t, ^{3}J = 7.5 Hz, 2H), 2.06 (br s, 1H), 1.60 (m, 2H), 1.49 (m, 2H), 1.31 (m, 2H), 1.30 (m, 2H), 1.24 (t, ^{3}J = 7.1 Hz, 3H). ^{13}C NMR (150 MHz, $CDCl_3$): 173.77, 146.30, 142.65, 138.96 (2C), 132.57, 130.56, 129.85, 128.83, 127.60, 127.48, 118.17 (2C), 65.71, 64.60, 64.17, 60.15, 47.92, 38.27, 34.24, 29.89, 28.96, 26.92, 24.82, 14.22. COSY: 7.36–7.26–7.32–7.34, 4.29–4.27, 4.11–1.24, 2.48–1.49–1.31–1.30–1.60–2.27. HSQC (140 Hz): 7.50–118.17, 7.36–129.85, 7.34–127.48, 7.32–128.83, 7.26–127.60, 6.95–118.17, 4.91–65.71, 4.29–64.60, 4.27–64.17, 4.11–60.15, 3.31–38.27, 2.48–47.92, 2.27–34.24, 1.60–24.82, 1.49–29.89, 1.31–26.92, 1.30–28.96, 1.24–14.22. HMBC (8 Hz, 140 Hz): 7.50–(146.30, 132.57), 7.36–(138.96, 128.83), 7.34–(138.96, 127.60), 7.32–(138.96, 129.85), 7.26–(138.96, 127.48), 6.95–(142.65, 130.56), 4.91–(138.96, 132.57, 130.56, 129.85, 118.17, 47.92), 4.29–146.30, 4.27–142.65, 4.11–(173.77, 14.22), 3.31–138.96, 2.27–(173.77, 28.96, 24.82), 1.60–(173.77, 34.24, 28.96, 26.92), 1.49–(47.92, 26.92), 1.31–28.96, 1.30–26.92, 1.24–60.15. HRMS (ESI): m/z calcd. for $C_{25}H_{33}N_2O_6S$ $[M+H]^+$: 489.2054, found: 489.2055.

7-[(7-Methyl-6,6-dioxido-2,3,7,12-tetrahydrobenzo[*c*][1,4]benzodioxino[6,7-*f*][1,2]thiazepin-12-yl)amino]heptanoic acid (54f). To a solution of ester **54e** (3.91 g, 8 mmol) in EtOH (40 mL), NaOH (0.384 g, 9.6 mmol) dissolved in water (10 mL) was added at room temperature and the solution was stirred at room temperature for 20 h. Water (30 mL) was

added, and the reaction mixture was partly evaporated in vacuo to remove ethanol. The solution obtained was neutralized with aqueous HCl solution (1.00 g of cc. HCl dissolved in 9 mL of water, containing 10 mmol of HCl). DCM (50 mL) was added to the reaction mixture, after a short stirring the phases were separated, the aqueous phase was washed with DCM (50 mL), the combined organic phases were dried over Na_2SO_4 and partially evaporated in vacuo. The solution obtained was subjected to dry-column flash chromatography on a short silica gel column (thickness of stationary phase: 25 mm, eluent: DCM, DCM/MeOH 100:2, 100:4). After evaporation of solvents the oily residue was triturated with water (40 mL) to afford **54f** (2.58 g, 70%) as a colorless amorphous solid. Melting range (glass-transition range): 80–95 °C (Kofler–Boëtius, 2 °C/min), 63–84 °C (DSC, 10 °C/min). IR (KBr): 2931, 1716, 1500, 1296, 1137, 1064 cm^{-1}. ^{1}H NMR (500 MHz, $CDCl_3$): 8.10 (br s, 2H), 7.49 (s, 1H), 7.47 (m, 1H), 7.34 (m, 1H), 7.30 (m, 1H), 7.24 (m, 1H), 7.07 (s, 1H), 5.23 (s, 1H), 4.28 (m, 4H), 3.21 (s, 3H), 2.55 (m, 1H), 2.41 (m, 1H), 2.20 (t, ^{3}J = 7.3 Hz, 2H), 1.54 (m, 2H), 1.48 (m, 2H), 1.25 (m, 4H). ^{13}C NMR (125 MHz, $CDCl_3$): 178.16, 146.61, 143.19, 139.78, 134.16, 131.69, 130.39, 129.64, 128.82, 127.40, 127.14, 120.35, 118.13, 65.58, 64.51, 64.20, 46.82, 38.83, 35.16, 28.93, 28.58, 26.79, 25.12. MS (ESI): $[M+H]^+$ = 461. HRMS (ESI): m/z calcd. for $C_{23}H_{29}N_2O_6S$ $[M+H]^+$: 461.1741, found: 461.1739.

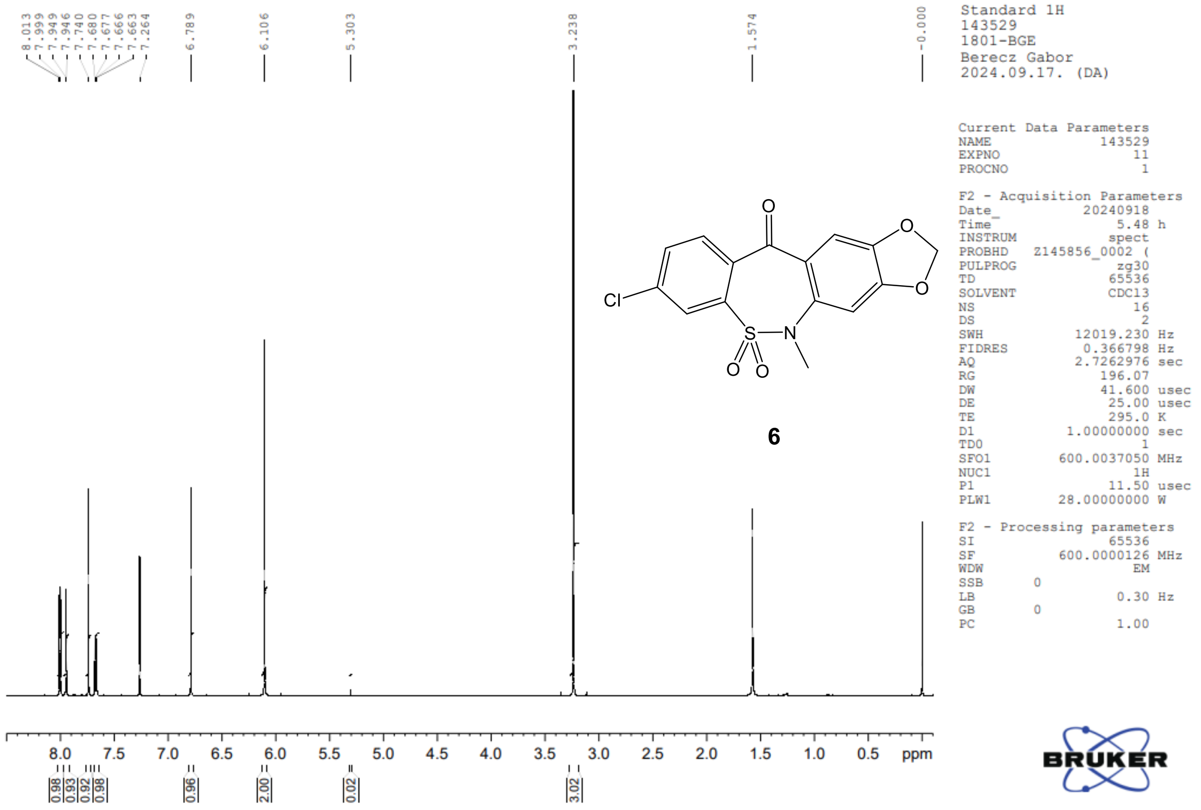
8.013 7.999 7.949 7.946 7.740 7.680 7.677 7.666 7.663 7.264
6.789
6.106
5.303
3.238
1.574
-0.000
Standard 1H
143529
1801-BGE
Berecz Gabor
2024.09.17. (DA)
Current Data Parameters
NAME 143529
EXPNO 11
PROCNO 1
F2 - Acquisition Parameters
Date_ 20240918
Time 5.48 h
INSTRUM spect
PROBHD Z145856_0002 (
PULPROG zg30
TD 65536
SOLVENT CDCl3
NS 16
DS 2
SWH 12019.230 Hz
FIDRES 0.366798 Hz
AQ 2.7262976 sec
RG 196.07
DW 41.600 usec
DE 25.00 usec
TE 295.0 K
D1 1.00000000 sec
TD0 1
SFO1 600.0037050 MHz
NUC1 1H
P1 11.50 usec
PLW1 28.00000000 W
F2 - Processing parameters
SI 65536
SF 600.0000126 MHz
WDW EM
SSB 0
LB 0.30 Hz
GB 0
PC 1.00
6
ppm
0.98 0.93 0.92 0.98 0.96 2.00 0.02 3.02
BRUKER

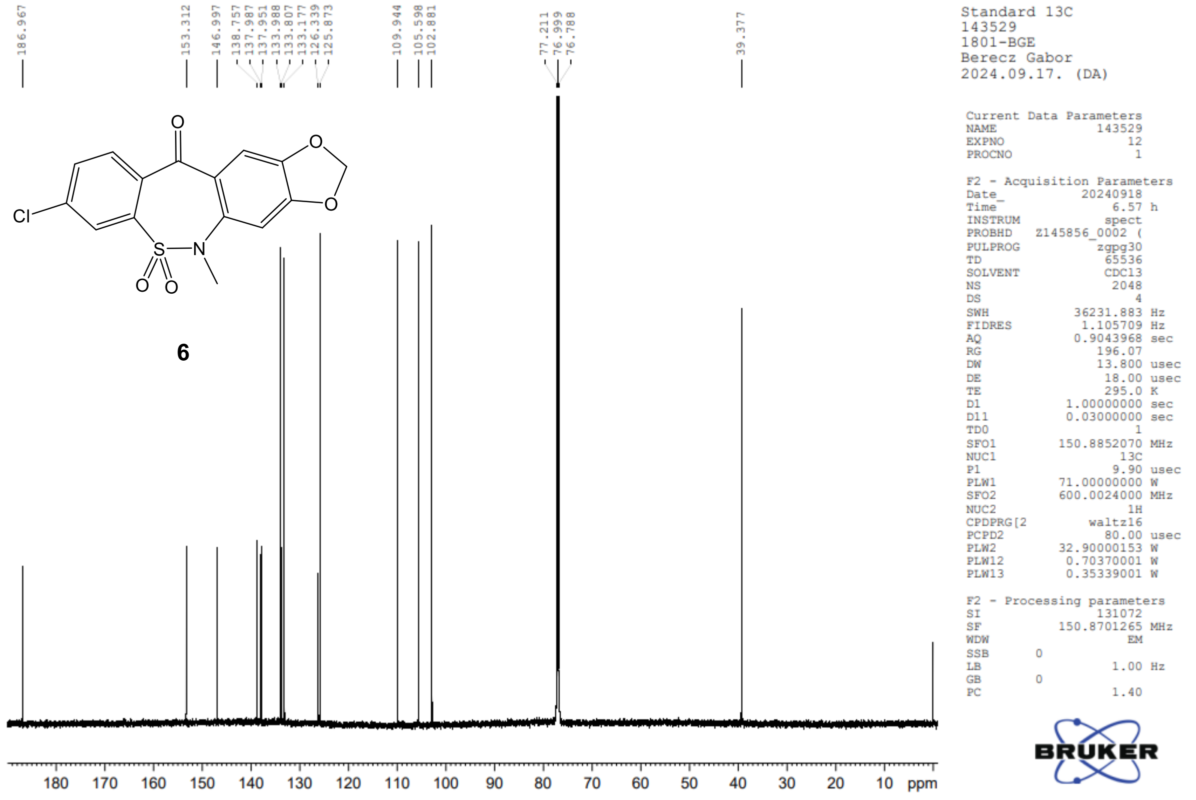
186.967
153.312
146.997
138.757 137.987 137.951 133.988 133.807 133.177 126.339 125.873
109.944
105.598
102.881
77.211 76.999 76.788
39.377
Standard 13C
143529
1801-BGE
Berecz Gabor
2024.09.17. (DA)
Current Data Parameters
NAME 143529
EXPNO 12
PROCNO 1
F2 - Acquisition Parameters
Date_ 20240918
Time 6.57 h
INSTRUM spect
PROBHD Z145856_0002 (
PULPROG zgpg30
TD 65536
SOLVENT CDCl3
NS 2048
DS 4
SWH 36231.883 Hz
FIDRES 1.105709 Hz
AQ 0.9043968 sec
RG 196.07
DW 13.800 usec
DE 18.00 usec
TE 295.0 K
D1 1.00000000 sec
D11 0.03000000 sec
TD0 1
SFO1 150.8852070 MHz
NUC1 13C
P1 9.90 usec
PLW1 71.00000000 W
SFO2 600.0024000 MHz
NUC2 1H
CPDPRG[2 waltz16
PCPD2 80.00 usec
PLW2 32.90000153 W
PLW12 0.70370001 W
PLW13 0.35339001 W
F2 - Processing parameters
SI 131072
SF 150.8701265 MHz
WDW EM
SSB 0
LB 1.00 Hz
GB 0
PC 1.40
6
ppm
BRUKER

6

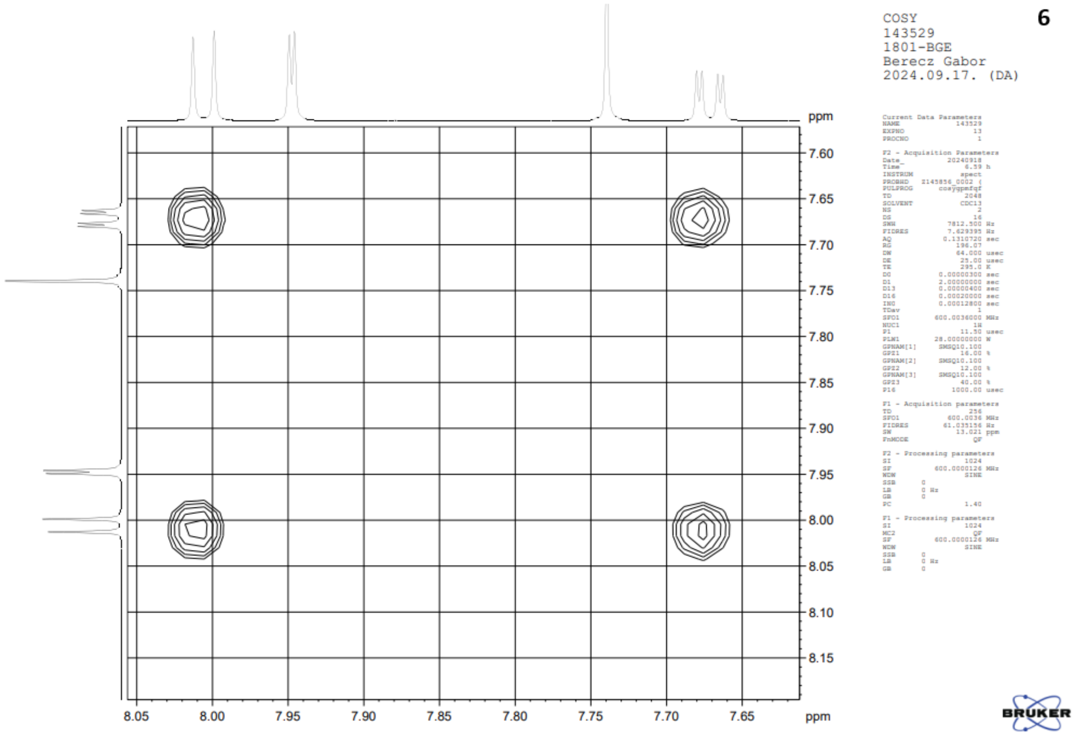
COSY
143529
1801-BGE
Berecz Gabor
2024.09.17. (DA)
ppm
7.60
7.65
7.70
7.75
7.80
7.85
7.90
7.95
8.00
8.05
8.10
8.15
8.05 8.00 7.95 7.90 7.85 7.80 7.75 7.70 7.65 ppm
BRUKER

6

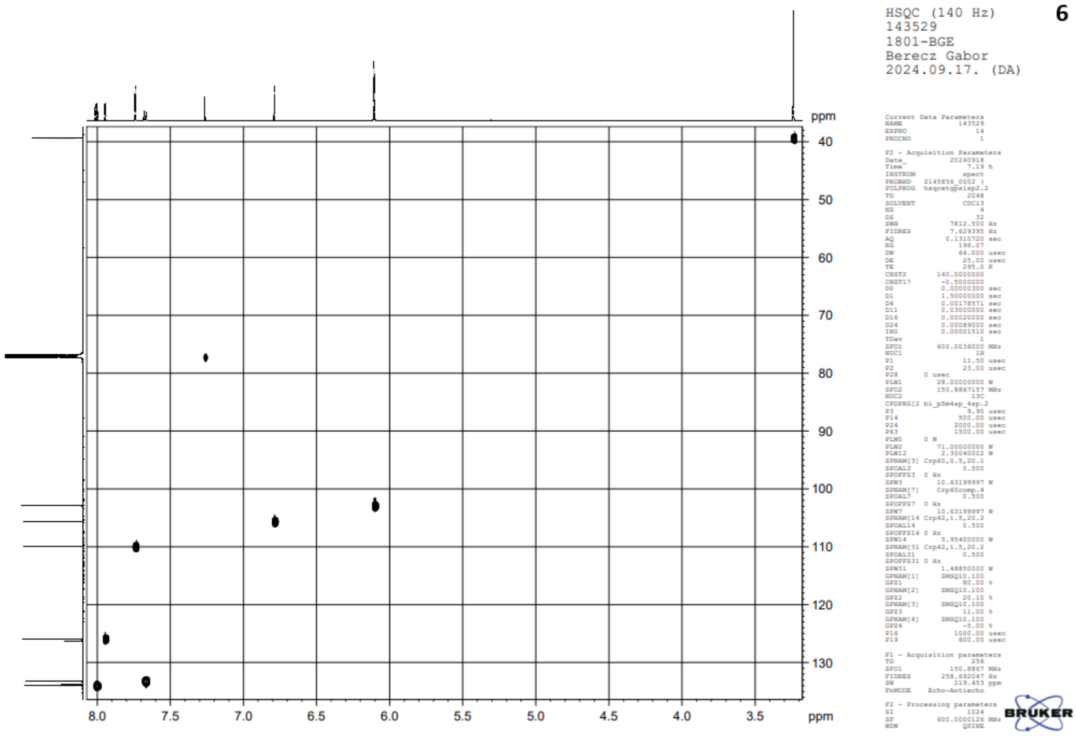
HSQC (140 Hz)
143529
1801-BGE
Berecz Gabor
2024.09.17. (DA)
ppm
40
50
60
70
80
90
100
110
120
130
8.0 7.5 7.0 6.5 6.0 5.5 5.0 4.5 4.0 3.5 ppm
BRUKER

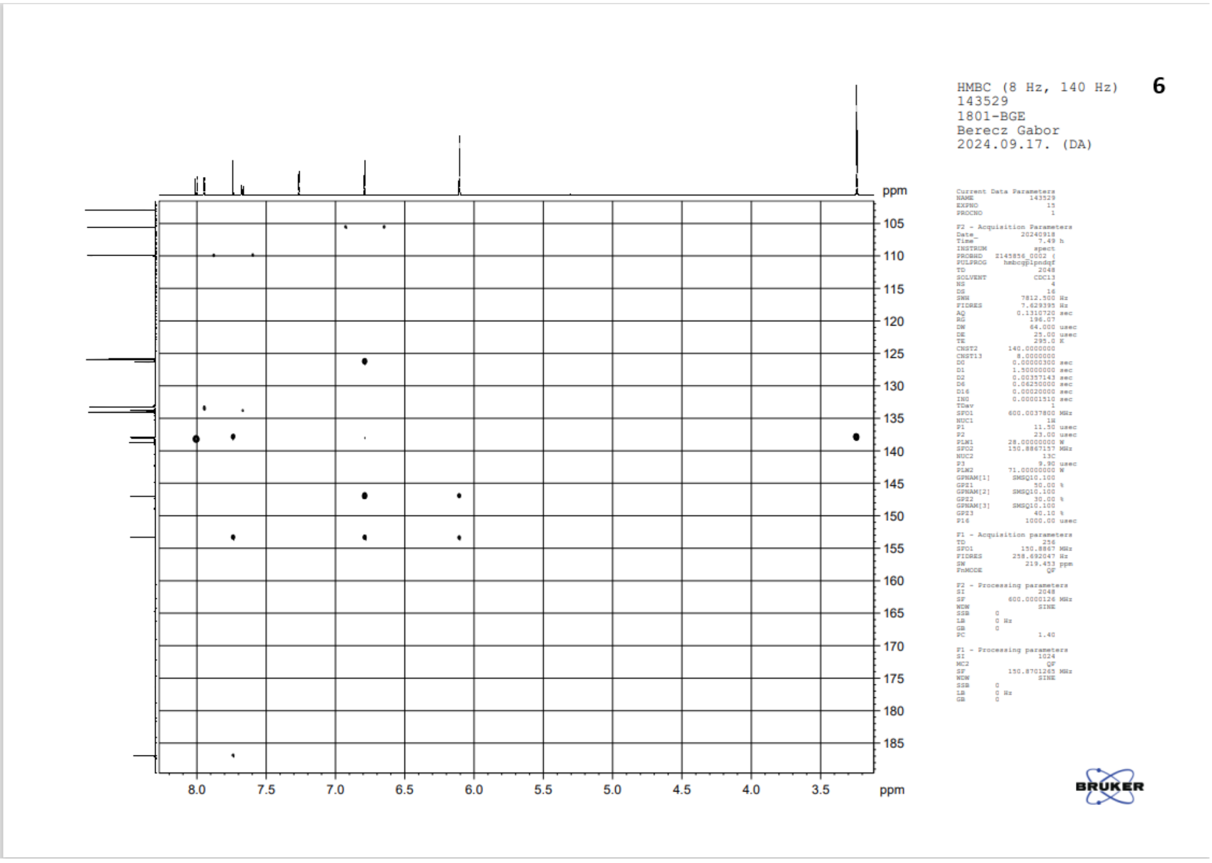
HMBC (8 Hz, 140 Hz)
143529
1801-BGE
Berecz Gabor
2024.09.17. (DA)
ppm

6

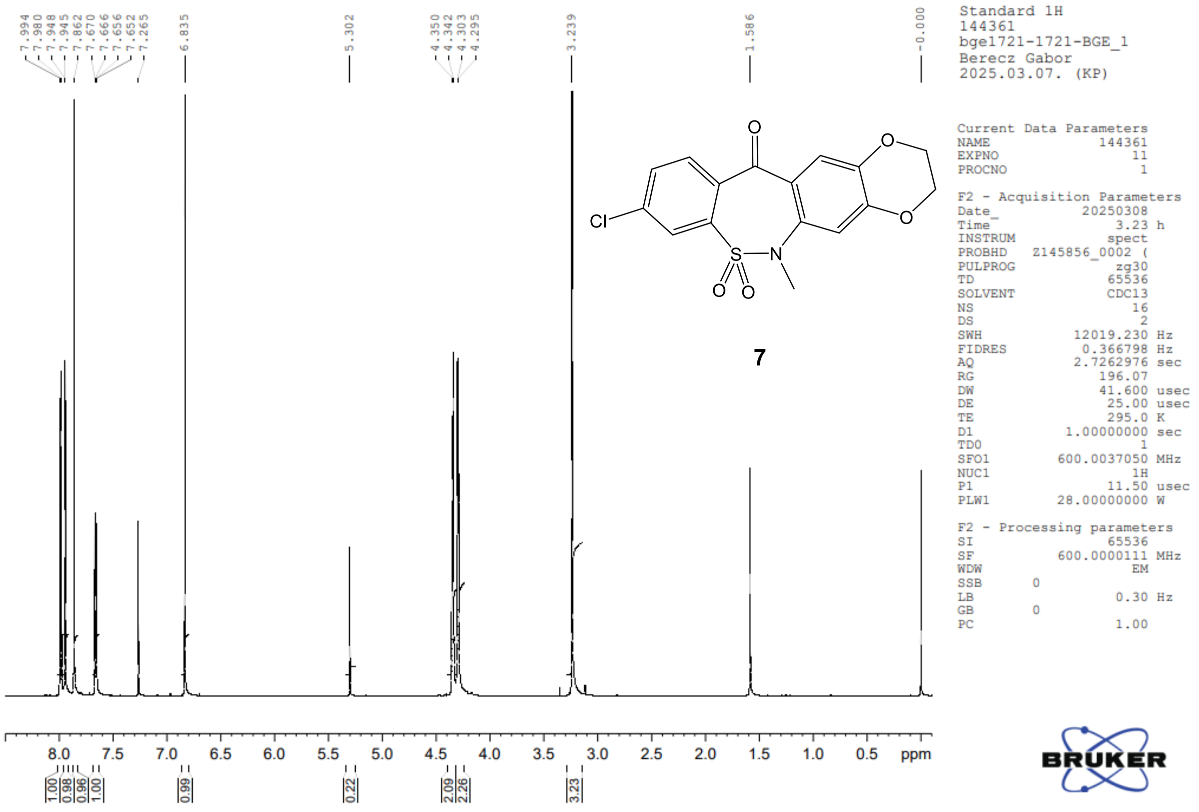
Standard 1H
144361
bge1721-1721-BGE_1
Berecz Gabor
2025.03.07. (KP)
Current Data Parameters
NAME 144361
EXPNO 11
PROCNO 1
F2 - Acquisition Parameters
Date_ 20250308
Time 3.23 h
INSTRUM spect
PROBHD Z145856_0002 (
PULPROG zg30
TD 65536
SOLVENT CDCl3
NS 16
DS 2
SWH 12019.230 Hz
FIDRES 0.366798 Hz
AQ 2.7262976 sec
RG 196.07
DW 41.600 usec
DE 25.00 usec
TE 295.0 K
D1 1.00000000 sec
TD0 1
SFO1 600.0037050 MHz
NUC1 1H
P1 11.50 usec
PLW1 28.00000000 W
F2 - Processing parameters
SI 65536
SF 600.0000111 MHz
WDW EM
SSB 0
LB 0.30 Hz
GB 0
PC 1.00
ppm

7

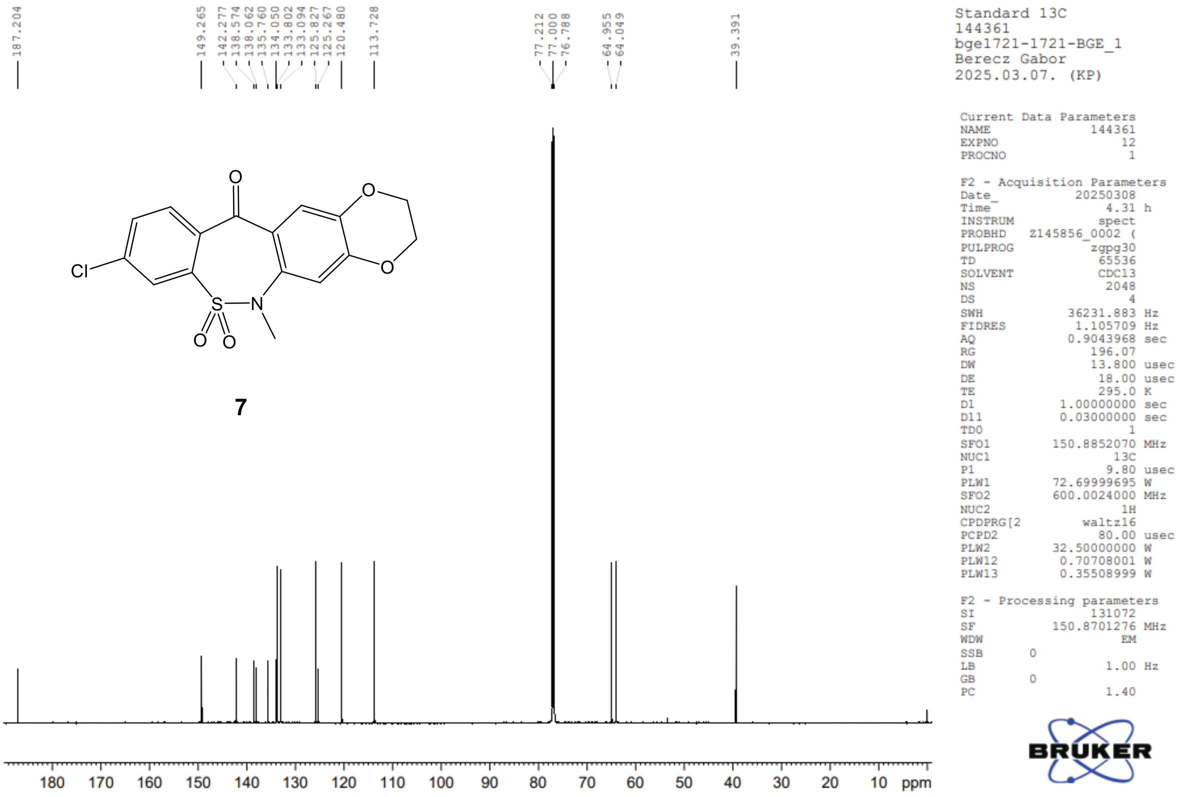
187.204
149.265
142.277
138.574
138.062
135.760
134.050
133.802
133.094
125.827
125.267
120.480
113.728
77.212
77.000
76.788
64.955
64.049
39.391
O
O
O
Cl
S
N
O
O
7
Standard 13C
144361
bge1721-1721-BGE_1
Berecz Gabor
2025.03.07. (KP)
Current Data Parameters
NAME 144361
EXPNO 12
PROCNO 1
F2 - Acquisition Parameters
Date_ 20250308
Time 4.31 h
INSTRUM spect
PROBHD Z145856_0002 (
PULPROG zgpg30
TD 65536
SOLVENT CDCl3
NS 2048
DS 4
SWH 36231.883 Hz
FIDRES 1.105709 Hz
AQ 0.9043968 sec
RG 196.07
DW 13.800 usec
DE 18.00 usec
TE 295.0 K
D1 1.00000000 sec
D11 0.03000000 sec
TD0 1
SFO1 150.8852070 MHz
NUC1 13C
P1 9.80 usec
PLW1 72.69999695 W
SFO2 600.0024000 MHz
NUC2 1H
CPDPRG[2 waltz16
PCPD2 80.00 usec
PLW2 32.50000000 W
PLW12 0.70708001 W
PLW13 0.35508999 W
F2 - Processing parameters
SI 131072
SF 150.8701276 MHz
WDW EM
SSB 0
LB 1.00 Hz
GB 0
PC 1.40
BRUKER
180 170 160 150 140 130 120 110 100 90 80 70 60 50 40 30 20 10 ppm

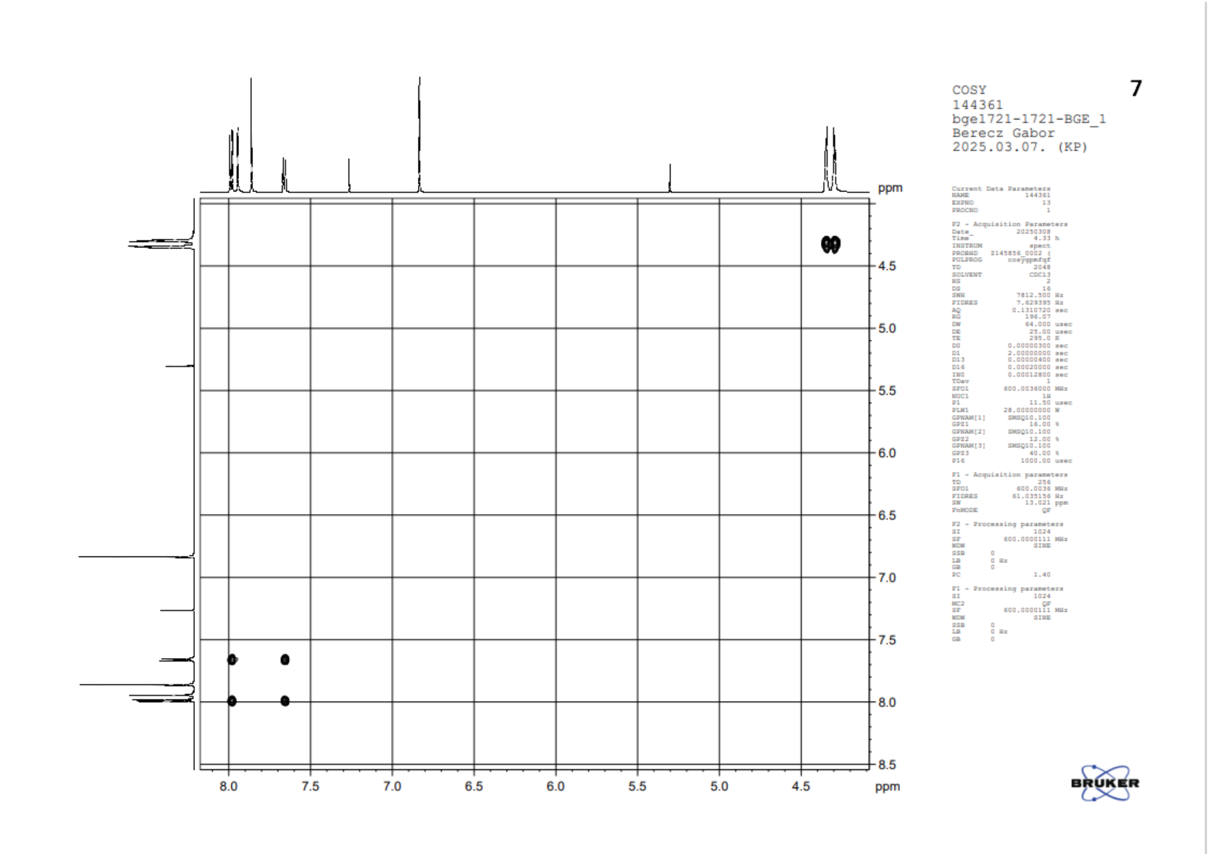
COSY
7
144361
bge1721-1721-BGE_1
Berecz Gabor
2025.03.07. (KP)
ppm
4.5
5.0
5.5
6.0
6.5
7.0
7.5
8.0
8.5
8.0 7.5 7.0 6.5 6.0 5.5 5.0 4.5 ppm
BRUKER

7

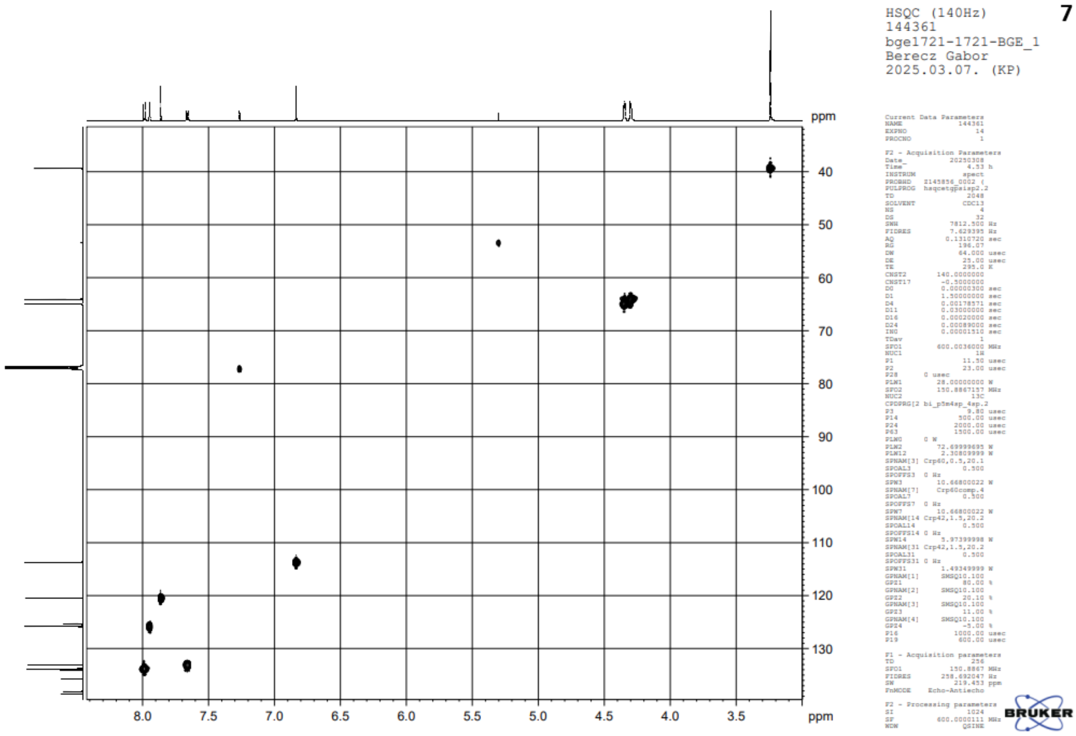
HSQC (140Hz)
144361
bge1721-1721-BGE_1
Berecz Gabor
2025.03.07. (KP)
ppm
BRUKER

7

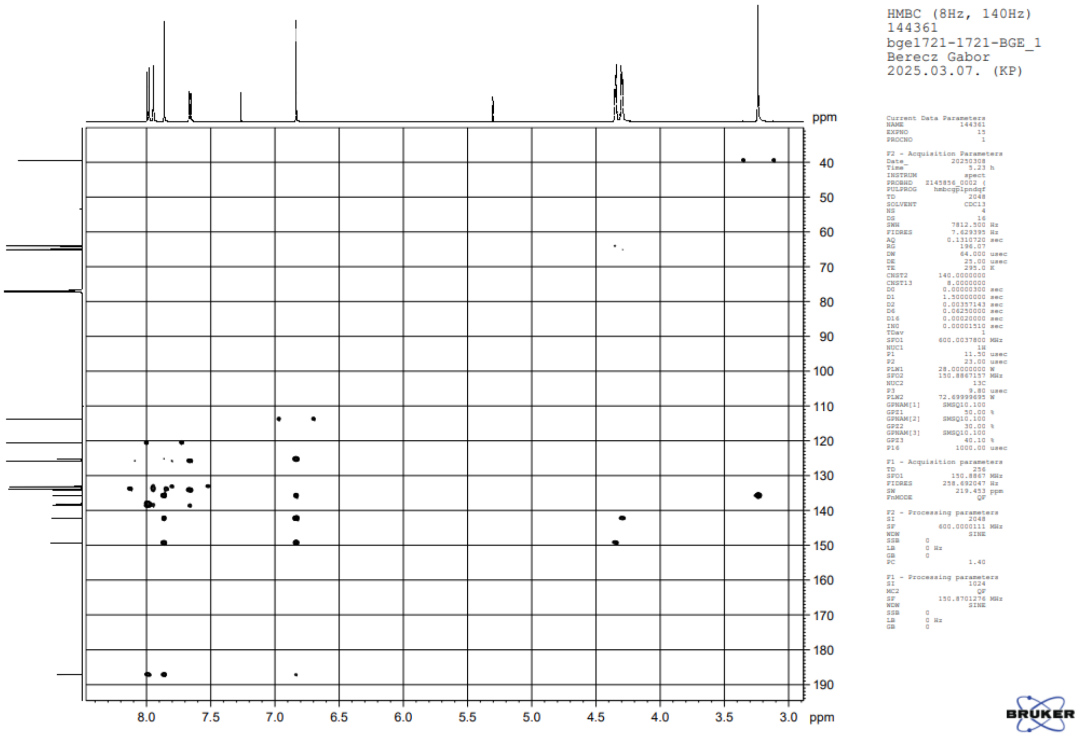
HMBC (8Hz, 140Hz)
144361
bge1721-1721-BGE_1
Berecz Gabor
2025.03.07. (KP)
ppm
BRUKER

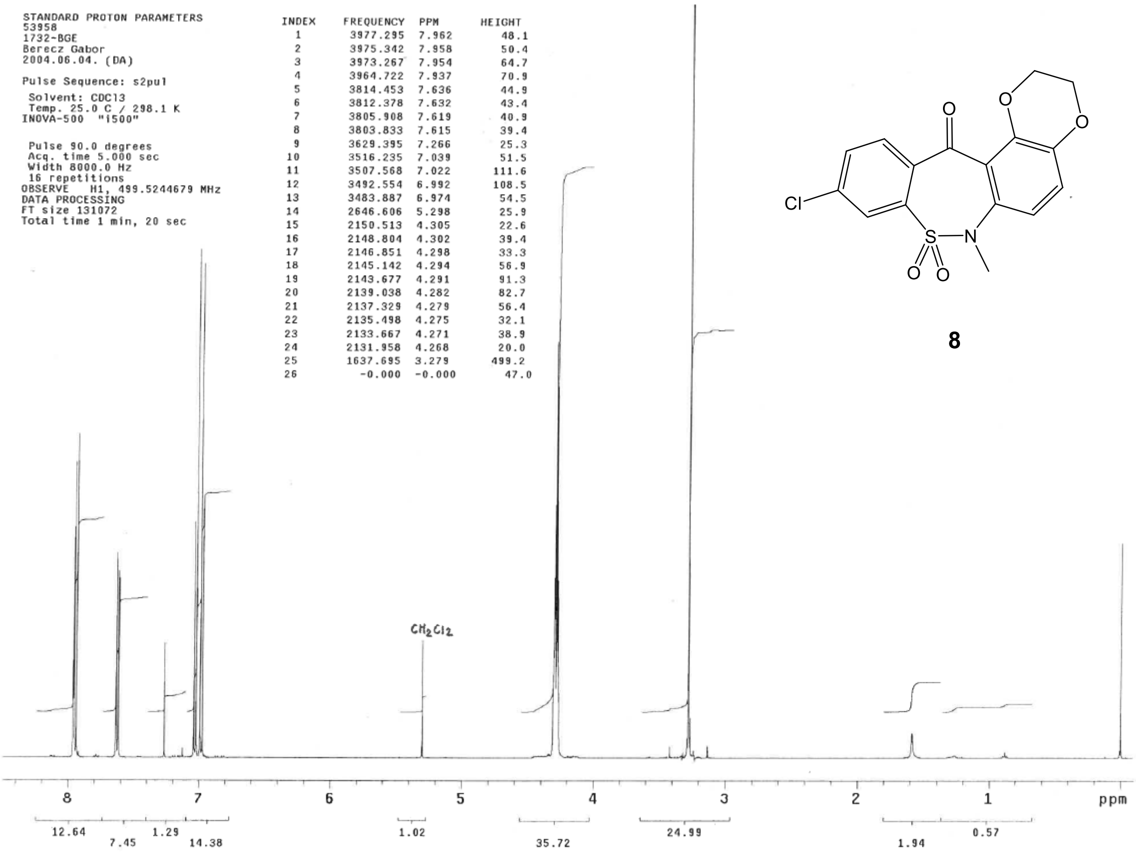
STANDARD PROTON PARAMETERS
53958
1732-BGE
Berecz Gabor
2004.06.04. (DA)
Pulse Sequence: s2pul
Solvent: CDCl3
Temp. 25.0 C / 298.1 K
INOVA-500 "i500"
Pulse 90.0 degrees
Acq. time 5.000 sec
Width 8000.0 Hz
16 repetitions
OBSERVE H1, 499.5244679 MHz
DATA PROCESSING
FT size 131072
Total time 1 min, 20 sec
INDEX FREQUENCY PPM HEIGHT
1 3977.295 7.962 48.1
2 3975.342 7.958 50.4
3 3973.267 7.954 64.7
4 3964.722 7.937 70.9
5 3814.453 7.636 44.9
6 3812.378 7.632 43.4
7 3805.908 7.619 40.9
8 3803.833 7.615 39.4
9 3629.395 7.266 25.3
10 3516.235 7.039 51.5
11 3507.568 7.022 111.6
12 3492.554 6.992 108.5
13 3483.887 6.974 54.5
14 2646.606 5.298 25.9
15 2150.513 4.305 22.6
16 2148.804 4.302 39.4
17 2146.851 4.298 33.3
18 2145.142 4.294 56.9
19 2143.677 4.291 91.3
20 2139.038 4.282 82.7
21 2137.329 4.279 56.4
22 2135.498 4.275 32.1
23 2133.667 4.271 38.9
24 2131.958 4.268 20.0
25 1637.695 3.279 499.2
26 -0.000 -0.000 47.0
CH2Cl2
8 7 6 5 4 3 2 1 ppm
12.64 7.45 1.29 14.38 1.02 35.72 24.99 1.94 0.57
O
O
O
Cl
S
N
O O

8

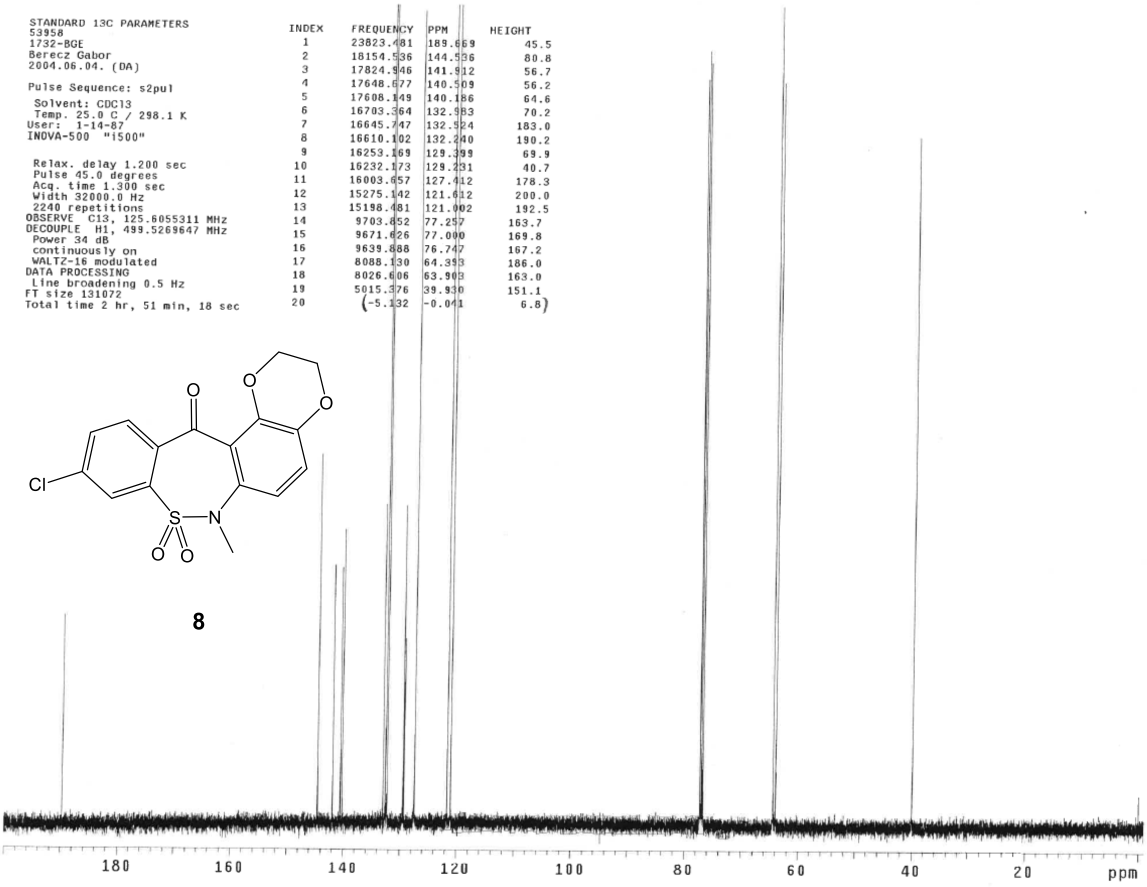
STANDARD 13C PARAMETERS
53958
1732-BGE
Berecz Gabor
2004.06.04. (DA)
Pulse Sequence: s2pul
Solvent: CDCl3
Temp. 25.0 C / 298.1 K
User: 1-14-87
INOVA-500 "i500"
Relax. delay 1.200 sec
Pulse 45.0 degrees
Acq. time 1.300 sec
Width 32000.0 Hz
2240 repetitions
OBSERVE C13, 125.6055311 MHz
DECOUPLE H1, 499.5269647 MHz
Power 34 dB
continuously on
WALTZ-16 modulated
DATA PROCESSING
Line broadening 0.5 Hz
FT size 131072
Total time 2 hr, 51 min, 18 sec
INDEX FREQUENCY PPM HEIGHT
1 23823.481 189.669 45.5
2 18154.536 144.536 80.8
3 17824.946 141.912 56.7
4 17648.677 140.509 56.2
5 17608.149 140.186 64.6
6 16703.364 132.983 70.2
7 16645.747 132.524 183.0
8 16610.102 132.240 190.2
9 16253.169 129.399 69.9
10 16232.173 129.231 40.7
11 16003.657 127.412 178.3
12 15275.142 121.612 200.0
13 15198.481 121.002 192.5
14 9703.852 77.257 163.7
15 9671.626 77.000 169.8
16 9639.888 76.747 167.2
17 8088.130 64.393 186.0
18 8026.606 63.903 163.0
19 5015.376 39.930 151.1
20 (-5.132 -0.041 6.8)
180 160 140 120 100 80 60 40 20 ppm
O
O
O
Cl
S
N
O O

8

GHSQC_DA (140 Hz)
53958
1732-BGE
Berecz Gabor
2004.06.04. (DA)

Pulse Sequence: ghsqc_da

Solvent: CDCl3
Temp. 25.0 C / 298.1 K
INOVA-500 "i500"

Relax. delay 1.500 sec
Acq. time 0.098 sec
Width 5219.9 Hz
2D Width 26367.8 Hz
2 repetitions
2 x 256 increments
OBSERVE H1, 499.5244683 MHz
DECOUPLE C13, 125.6174881 MHz
Power 50 dB
on during acquisition
off during delay
GARP-1 modulated
DATA PROCESSING
Sq. sine bell 0.200 sec
Shifted by -0.200 sec
F1 DATA PROCESSING
Line broadening 0.3 Hz
Sq. sine bell 0.005 sec
Shifted by -0.005 sec
FT size 2048 x 512
Total time 28 min, 35 sec

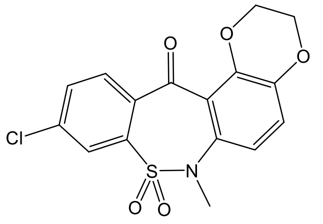

8

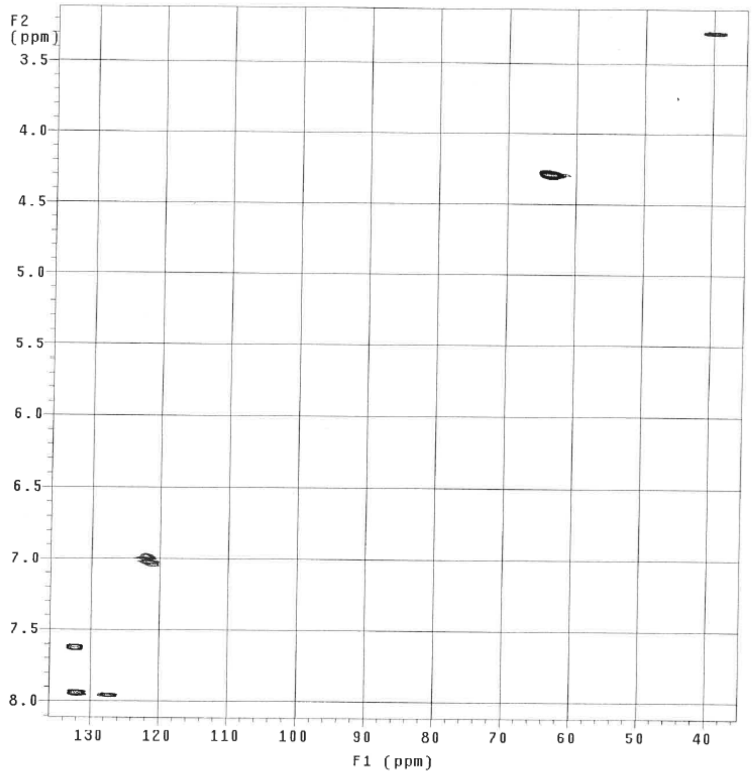

GHMQC_DA (140 Hz, 8 Hz)
53958
1732-BGE
Berecz Gabor
2004.06.04. (DA)

Pulse Sequence: ghmqc_da

Solvent: CDCl3
Temp. 25.0 C / 298.1 K
INOVA-500 "i500"

Relax. delay 1.500 sec
Acq. time 0.098 sec
Width 5219.9 Hz
2D Width 26367.8 Hz
4 repetitions
256 increments
OBSERVE H1, 499.5244681 MHz
DATA PROCESSING
Sine bell 0.098 sec
F1 DATA PROCESSING
Sine bell 0.002 sec
FT size 2048 x 512
Total time 29 min, 9 sec

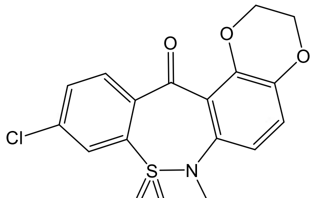

8

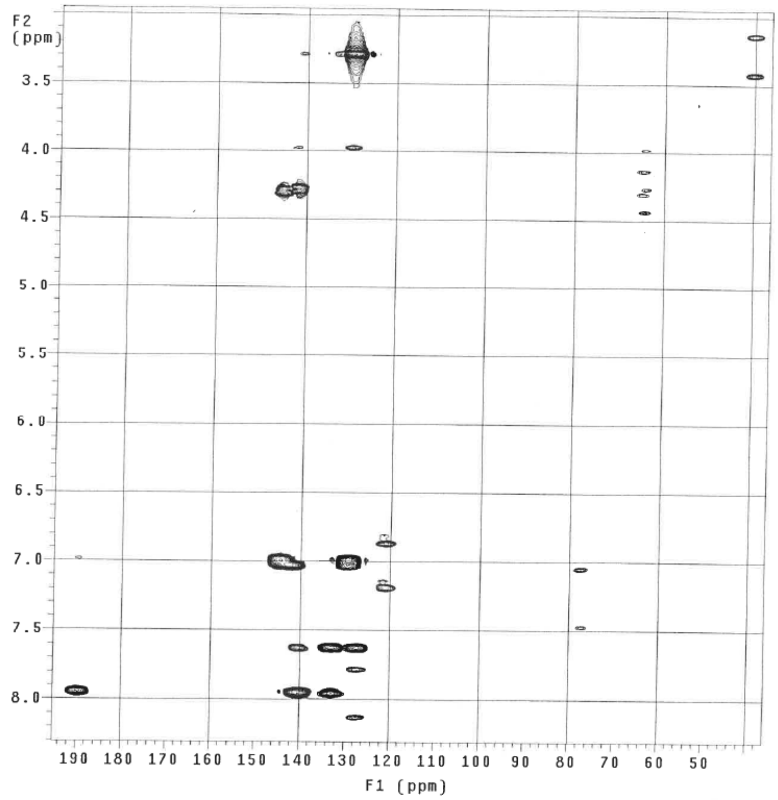

STANDARD PROTON PARAMETERS
56253
1799-BGE
Berecz Gabor
2005.01.11. (BT)
Solvent: DMSO
Temp. 25.0 C / 298.1 K
INOVA-500 "i500"
PULSE SEQUENCE
Relax. delay 3.000 sec
Pulse 90.0 degrees
Acq. time 5.000 sec
Width 8000.0 Hz
16 repetitions
OBSERVE H1, 499.5268308 MHz
DATA PROCESSING
FT size 131072
Total time 2 minutes

INDEX	FREQUENCY	PPM	HEIGHT
1	5018.799	10.047	15.1
2	3929.199	7.866	93.6
3	3927.124	7.862	106.7
4	3898.682	7.805	62.5
5	3896.606	7.801	52.4
6	3890.381	7.788	78.2
7	3888.306	7.784	72.8
8	3838.867	7.685	115.1
9	3830.566	7.668	90.7
10	3407.593	6.822	96.5
11	3399.292	6.805	109.2
12	3337.646	6.682	95.2
13	3335.449	6.677	102.4
14	3267.578	6.541	69.0
15	3265.381	6.537	62.8
16	3259.277	6.525	62.6
17	3257.080	6.520	60.0
18	2989.624	5.985	382.1
19	1913.208	3.830	556.9
20	1677.002	3.357	7.0
21	1256.958	2.516	10.5
22	1255.127	2.513	14.4
23	1253.296	2.509	9.9
24	-0.000	-0.000	14.1

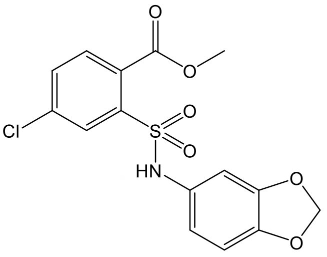

10

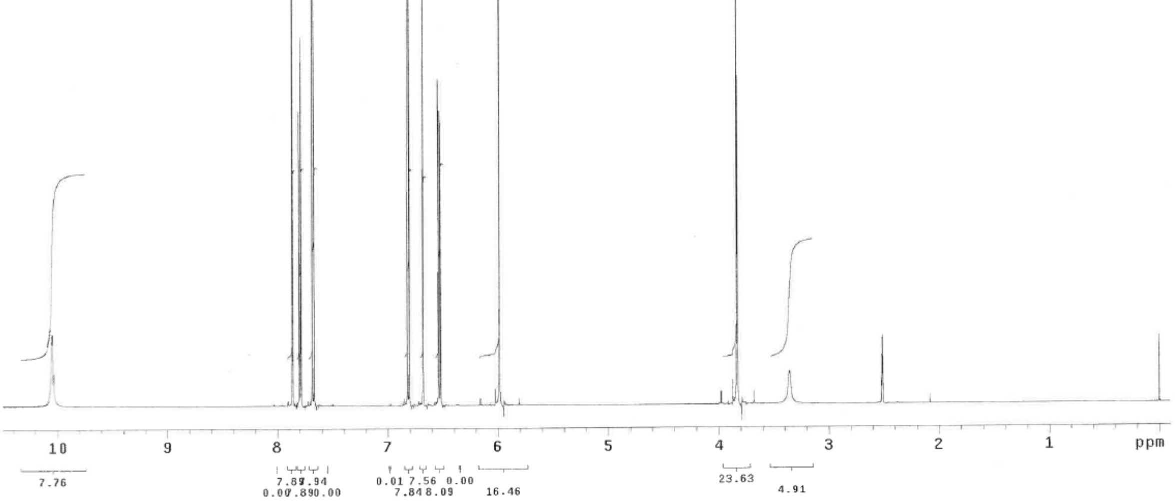

STANDARD CARBON PARAMETERS
56253
1799-BGE
Berecz Gabor
2005.01.11. (BT)
Solvent: DMSO
Temp. 25.0 C / 298.1 K
User: 1-14-87
INOVA-500 "i500"
PULSE SEQUENCE
Relax. delay 3.000 sec
Pulse 45.0 degrees
Acq. time 1.300 sec
Width 32000.0 Hz
128 repetitions
OBSERVE C13, 125.6061577 MHz
DECOUPLE H1, 499.5293374 MHz
Power 32 dB
continuously on
WALTZ-16 modulated
DATA PROCESSING
Line broadening 0.5 Hz
FT size 131072
Total time 9 minutes

INDEX	FREQUENCY	PPM	HEIGHT
1	20941.352	166.716	73.2
2	18553.657	147.707	94.8
3	18226.509	145.103	73.9
4	17432.563	138.782	79.9
5	17022.407	135.517	75.5
6	16716.255	133.079	91.5
7	16499.458	131.353	83.1
8	16460.395	131.042	89.4
9	16393.989	130.514	98.2
10	16109.321	128.248	89.1
11	14517.036	115.571	110.5
12	13628.364	108.496	120.0
13	13079.536	104.127	116.6
14	12766.059	101.632	113.5
15	6696.723	53.313	142.5
16	5028.755	40.034	86.1
17	5008.247	39.871	172.2
18	4987.251	39.704	200.0
19	4966.255	39.537	170.3
20	4945.259	39.370	84.5

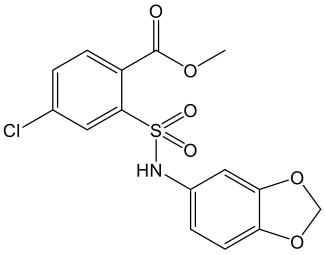

10

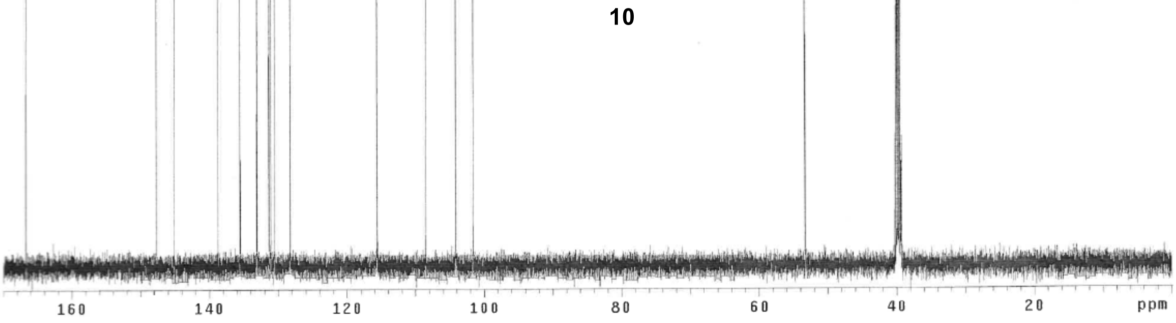

STANDARD 1H OBSERVE
52613
1699-BGE
Berecz Gabor
04/03/09(NM)

Solvent: CDCl3
Temp. 25.0 C / 298.1 K
Mercury-200 "mp200"

PULSE SEQUENCE
Relax. delay 5.000 sec
Pulse 31.5 degrees
Acq. time 1.993 sec
Width 3003.0 Hz
16 repetitions
OBSERVE H1, 200.0557670 MHz
DATA PROCESSING
FT size 16384
Total time 1 minutes

INDEX	FREQUENCY	PPM	HEIGHT
1	1592.779	7.962	30.1
2	1566.752	7.832	52.7
3	1564.919	7.822	78.5
4	1557.221	7.784	59.6
5	1512.865	7.562	39.8
6	1510.666	7.551	38.6
7	1504.434	7.520	29.1
8	1502.235	7.509	24.3
9	1453.480	7.265	18.6
10	1344.973	6.723	31.1
11	1342.040	6.708	42.5
12	1339.474	6.696	53.1
13	1336.542	6.681	70.9
14	1323.345	6.615	43.6
15	1320.779	6.602	31.0
16	1314.547	6.571	17.8
17	1312.348	6.560	16.0
18	1059.409	5.296	8.6
19	839.463	4.196	419.7
20	804.638	4.022	321.8
21	325.521	1.627	20.2
22	0.000	0.000	23.1

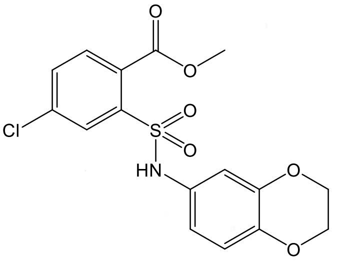

11

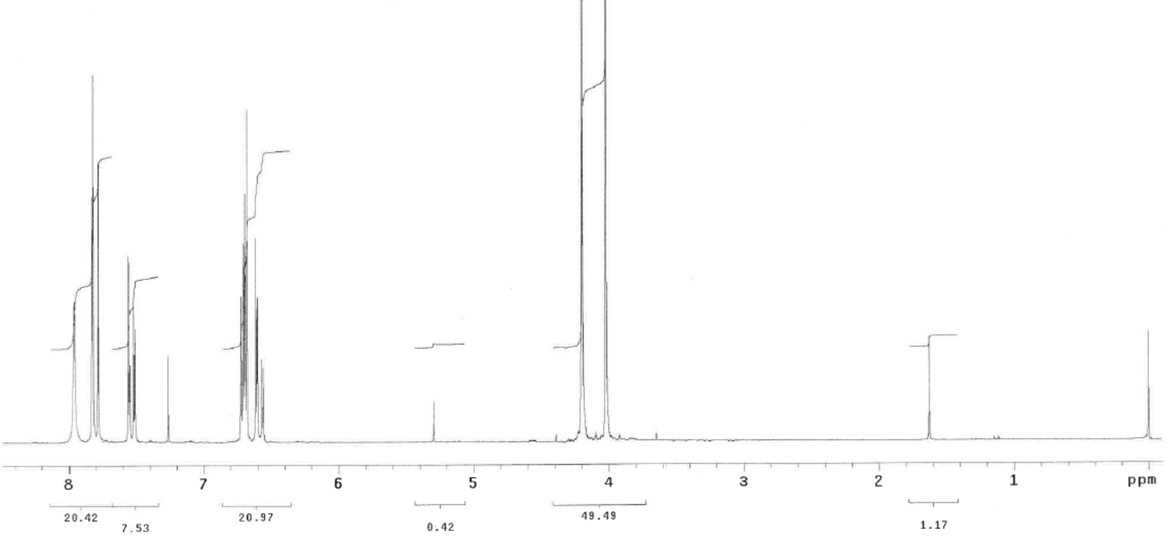

STANDARD 13C OBSERVE
52613
1699-BGE
Berecz Gabor
04/03/09(NM)

Solvent: CDCl3
Temp. 25.0 C / 298.1 K
Mercury-200 "mp200"

PULSE SEQUENCE
Relax. delay 3.000 sec
Pulse 60.0 degrees
Acq. time 1.497 sec
Width 12484.4 Hz
144 repetitions
OBSERVE C13, 50.3040647 MHz
DECOUPLE H1, 200.0567131 MHz
Power 36 dB
continuously on
WALTZ-16 modulated
DATA PROCESSING
Line broadening 1.0 Hz
FT size 65536
Total time 10 minutes

INDEX	FREQUENCY	PPM	HEIGHT
1	8414.392	167.258	35.2
2	7224.168	143.599	52.3
3	7156.351	142.251	44.1
4	7051.578	140.168	43.0
5	6951.377	138.177	49.5
6	6661.822	132.421	86.8
7	6648.106	132.148	76.9
8	6572.288	130.641	80.9
9	6503.328	129.270	56.4
10	6461.800	128.445	38.0
11	5910.121	117.479	80.0
12	5885.738	116.994	76.8
13	5691.050	113.124	67.0
14	3905.715	77.636	157.3
15	3873.711	77.000	162.0
16	3841.708	76.364	157.7
17	3230.213	64.209	77.0
18	3225.260	64.110	76.0
19	2697.965	53.629	65.4

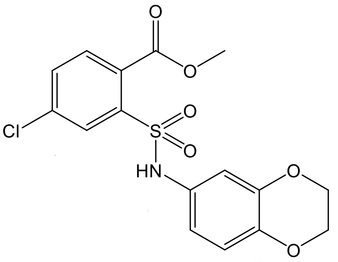

11

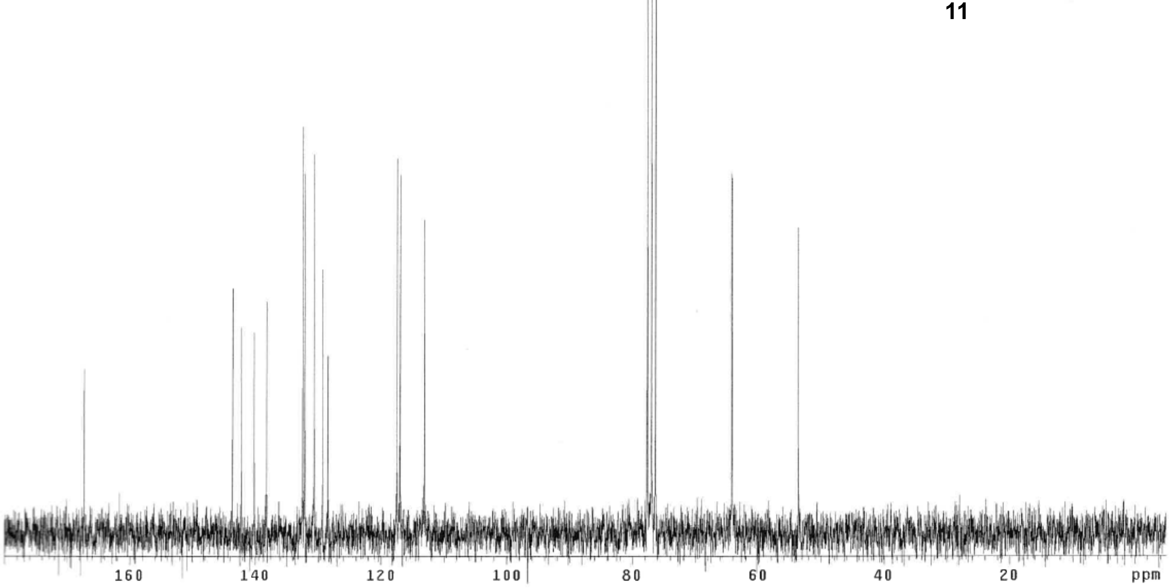

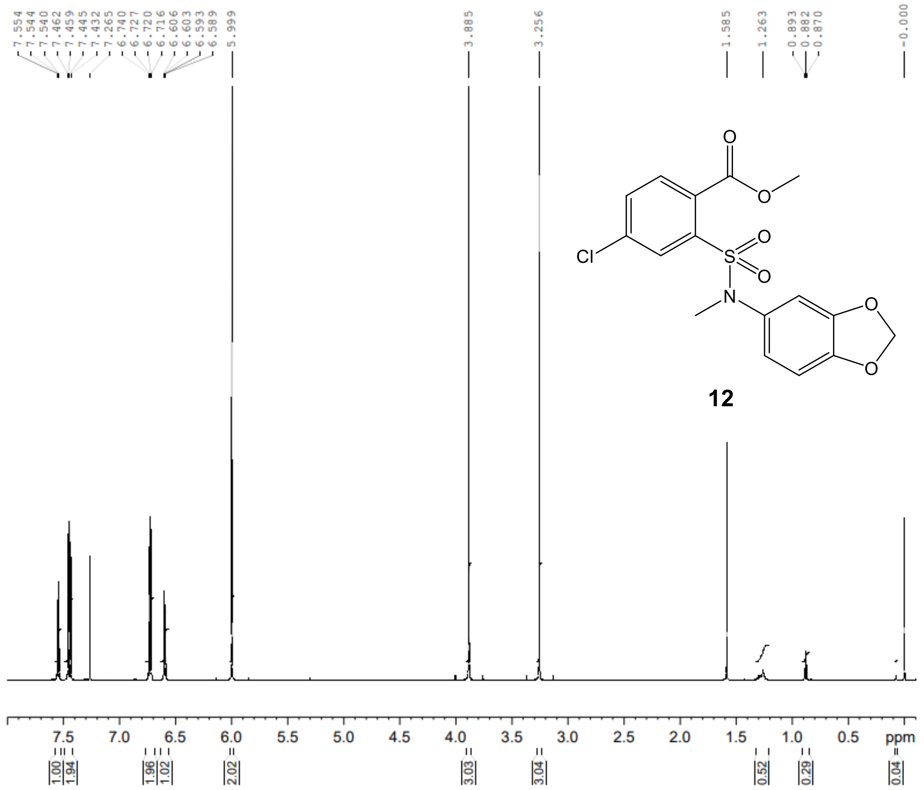

Standard 1H
143511
BEG0702_1
Berecz Gabor
2024.09.13. (KP)

Current Data Parameters
NAME 143511
EXPNO 11
PROCNO 1

F2 - Acquisition Parameters
Date_ 20240913
Time 21.39 h
INSTRUM spect
PROBHD Z145856_0002 (
PULPROG zg30
TD 65536
SOLVENT CDCl3
NS 16
DS 2
SWH 12019.230 Hz
FIDRES 0.366798 Hz
AQ 2.7262976 sec
RG 196.07
DW 41.600 usec
DE 25.00 usec
TE 295.0 K
D1 1.00000000 sec
TD0 1
SFO1 600.0037050 MHz
NUC1 1H
P1 11.50 usec
PLW1 28.00000000 W

F2 - Processing parameters
SI 65536
SF 600.0000121 MHz
WDW EM
SSB 0
LB 0.30 Hz
GB 0
PC 1.00

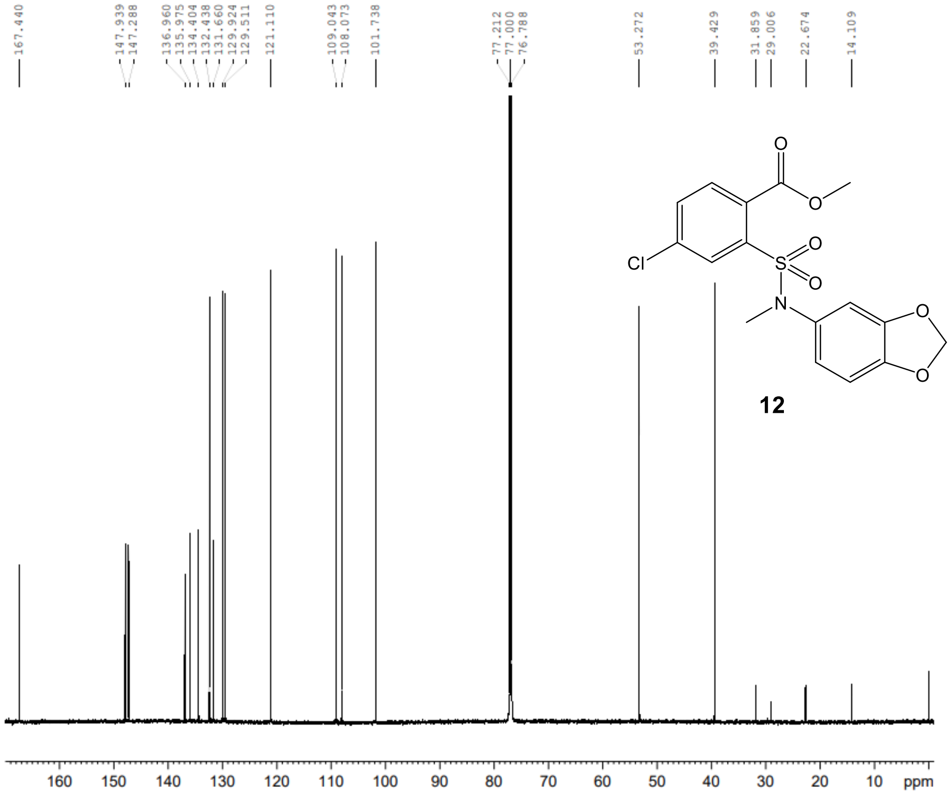

Standard 13C
143511
BEG0702_1
Berecz Gabor
2024.09.13. (KP)

Current Data Parameters
NAME 143511
EXPNO 12
PROCNO 1

F2 - Acquisition Parameters
Date_ 20240913
Time 22.48 h
INSTRUM spect
PROBHD Z145856_0002 (
PULPROG zgpg30
TD 65536
SOLVENT CDCl3
NS 2048
DS 4
SWH 36231.883 Hz
FIDRES 1.105709 Hz
AQ 0.9043968 sec
RG 196.07
DW 13.800 usec
DE 18.00 usec
TE 295.0 K
D1 1.00000000 sec
D11 0.03000000 sec
TD0 1
SFO1 150.8852070 MHz
NUC1 13C
P1 9.90 usec
PLW1 71.00000000 W
SFO2 600.0024000 MHz
NUC2 1H
CPDPRG[2 waltz16
PCPD2 80.00 usec
PLW2 32.90000153 W
PLW12 0.70370001 W
PLW13 0.35339001 W

F2 - Processing parameters
SI 131072
SF 150.8701269 MHz
WDW EM
SSB 0
LB 1.00 Hz
GB 0
PC 1.40

12

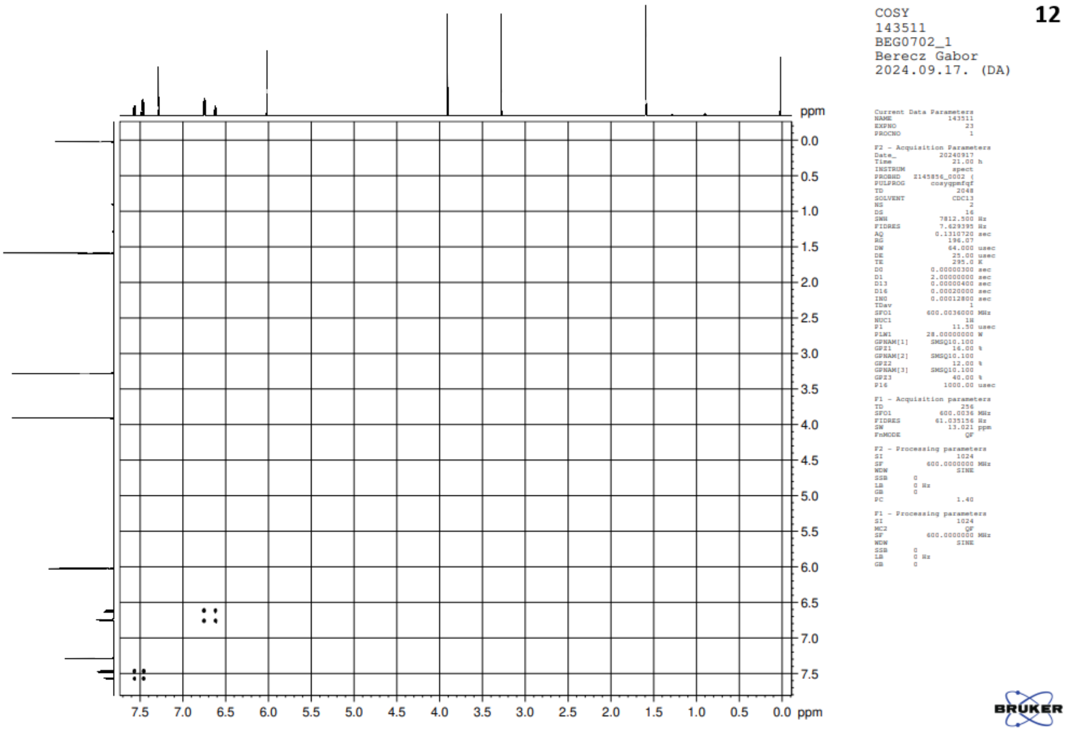

COSY
143511
BEG0702_1
Berecz Gabor
2024.09.17. (DA)
ppm
BRUKER

12

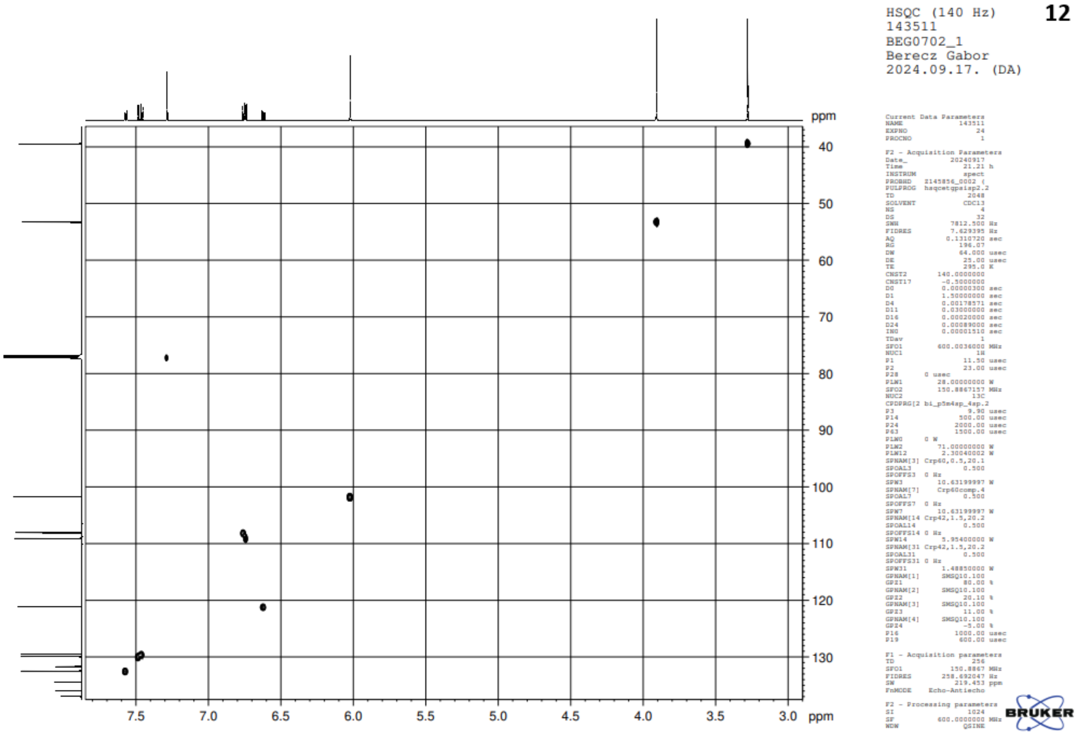

HSQC (140 Hz)
143511
BEG0702_1
Berecz Gabor
2024.09.17. (DA)
ppm
BRUKER

12

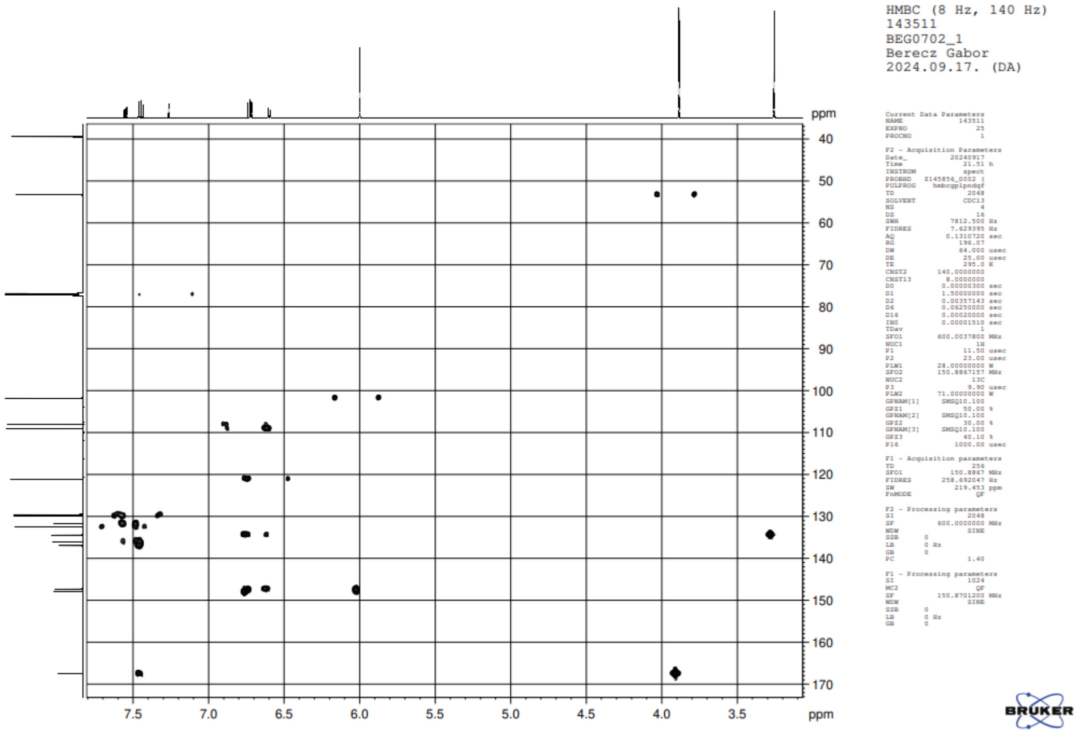
HMBC (8 Hz, 140 Hz)
143511
BEG0702_1
Berecz Gabor
2024.09.17. (DA)
ppm
40
50
60
70
80
90
100
110
120
130
140
150
160
170
7.5
7.0
6.5
6.0
5.5
5.0
4.5
4.0
3.5
ppm
BRUKER

STANDARD 1H OBSERVE
52745
1702-BGE
Berecz Gabor
04/03/16(NM)

Solvent: CDCl3
Temp. 25.0 C / 298.1 K
Mercury-200 "mp200"

PULSE SEQUENCE
Relax. delay 5.000 sec
Pulse 31.5 degrees
Acq. time 1.993 sec
Width 3003.0 Hz
16 repetitions
OBSERVE H1, 200.0557652 MHz
DATA PROCESSING
FT size 16384
Total time 1 minutes

INDEX	FREQUENCY	PPM	HEIGHT
1	1513.599	7.566	19.7
2	1511.766	7.557	23.2
3	1505.534	7.526	41.5
4	1503.701	7.516	43.9
5	1490.138	7.449	71.1
6	1487.205	7.434	60.2
7	1485.006	7.423	49.6
8	1482.073	7.408	33.3
9	1454.946	7.273	12.4
10	1364.768	6.822	38.5
11	1355.970	6.778	65.6
12	1344.973	6.723	45.2
13	1342.407	6.710	63.7
14	1335.075	6.674	43.7
15	1332.509	6.661	29.4
16	1326.277	6.630	26.7
17	1323.711	6.617	19.7
18	1059.776	5.297	3.9
19	853.392	4.266	17.3
20	849.360	4.246	248.8
21	844.961	4.224	14.0
22	841.295	4.205	3.5
23	774.945	3.874	333.4
24	648.476	3.241	363.2
25	333.952	1.669	12.6
26	-0.000	-0.000	14.2

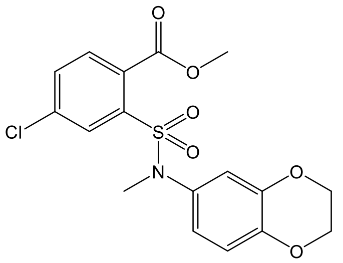

13

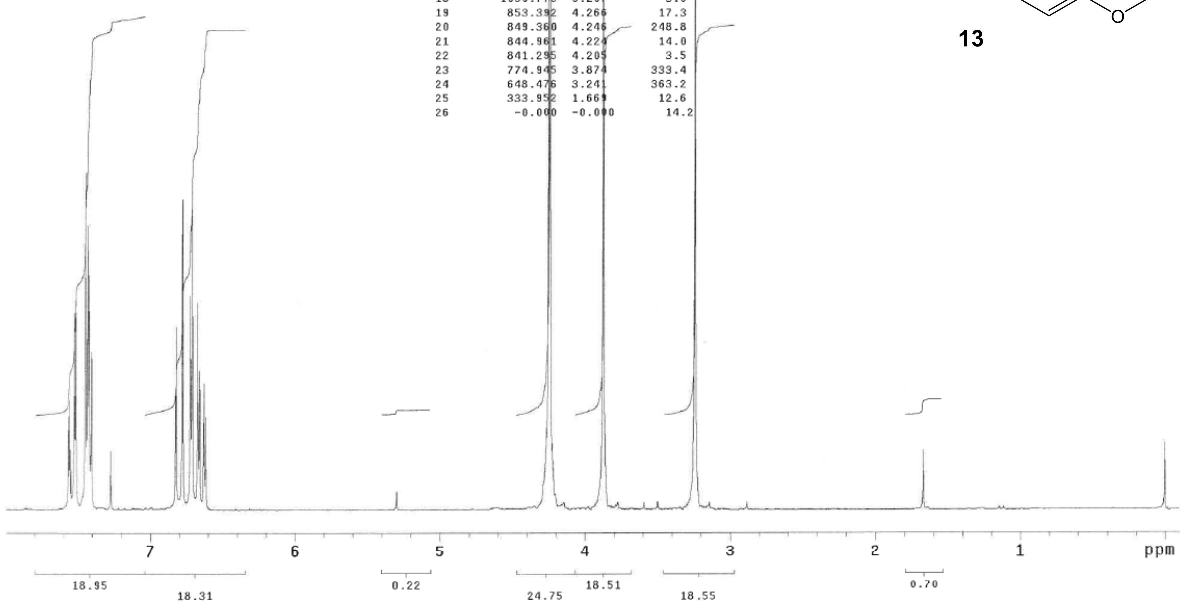

STANDARD 13C OBSERVE
52745
1702-BGE
Berecz Gabor
04/03/16(NM)

Solvent: CDCl3
Temp. 25.0 C / 298.1 K
Mercury-200 "mp200"

PULSE SEQUENCE
Relax. delay 3.000 sec
Pulse 60.0 degrees
Acq. time 1.497 sec
Width 12484.4 Hz
96 repetitions
OBSERVE C13, 50.3040655 MHz
DECOUPLE H1, 200.0567131 MHz
Power 36 dB
continuously on
WALTZ-16 modulated
DATA PROCESSING
Line broadening 1.0 Hz
FT size 65536
Total time 7 minutes

INDEX	FREQUENCY	PPM	HEIGHT
1	8420.869	167.386	51.9
2	7218.072	143.478	67.0
3	7210.833	143.334	61.6
4	6891.180	136.980	64.0
5	6834.412	135.852	75.2
6	6731.162	133.799	69.5
7	6656.869	132.322	115.0
8	6622.579	131.641	57.5
9	6531.522	129.831	104.5
10	6512.091	129.445	115.0
11	6063.662	120.531	97.9
12	5901.740	117.312	102.3
13	5871.260	116.706	91.2
14	3905.715	77.636	162.0
15	3873.711	77.000	159.7
16	3842.089	76.371	155.1
17	3232.880	64.262	98.4
18	3227.927	64.163	94.3
19	2674.344	53.159	104.9
20	1968.744	39.134	87.3

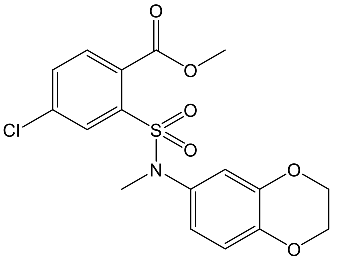

13

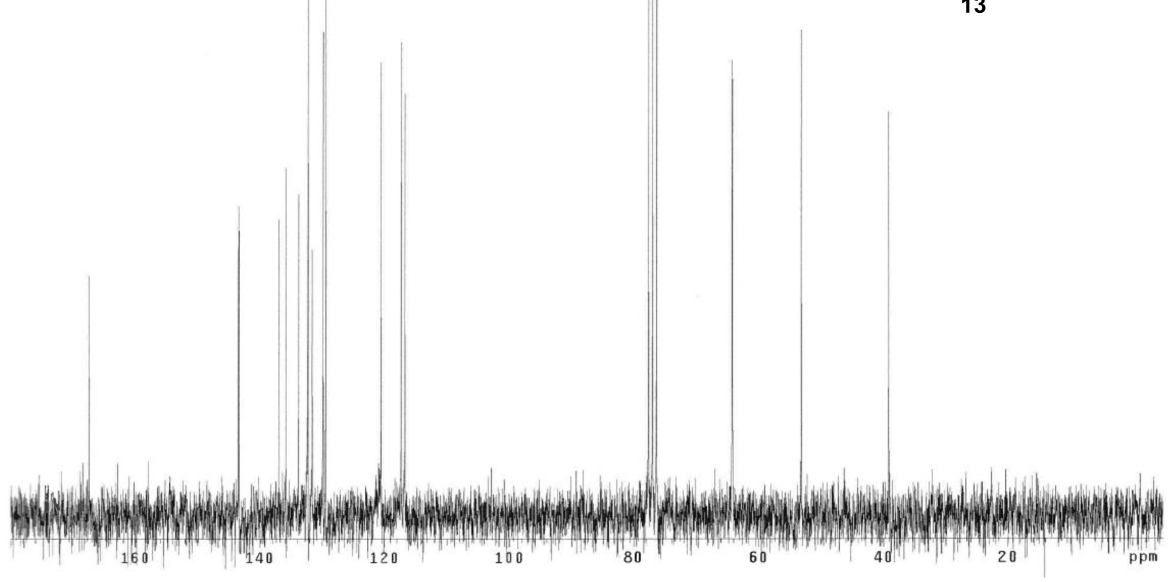

STANDARD PROTON PARAMETERS
56386
1800-BGE
Berecz Gabor
2005/01/19 (SzA)

Solvent: DMSO
Temp. 25.0 C / 298.1 K
INOVA-500 "i500"

PULSE SEQUENCE
Relax. delay 3.000 sec
Pulse 90.0 degrees
Acq. time 5.000 sec
Width 11999.4 Hz
16 repetitions
OBSERVE H1, 499.5268292 MHz
DATA PROCESSING
FT size 131072
Total time 2 minutes

INDEX	FREQUENCY	PPM	HEIGHT	INDEX	FREQUENCY	PPM	HEIGHT	INDEX	FREQUENCY	PPM	HEIGHT
1	6856.041	13.725	0.7	40	3404.310	6.815	142.7	79	1252.013	2.506	18.7
2	3905.261	7.818	83.2	41	3386.183	6.779	-0.5	80	1250.182	2.503	8.4
3	3903.247	7.814	80.5	42	3383.986	6.774	-0.7	81	1038.156	2.078	0.7
4	3897.022	7.801	100.6	43	3351.029	6.708	0.6	82	0.000	0.000	7.5
5	3895.008	7.797	102.6	44	3325.761	6.658	0.9				
6	3891.163	7.790	1.1	45	3320.269	6.647	95.7				
7	3874.867	7.757	-0.5	46	3318.071	6.642	89.3				
8	3849.783	7.707	0.6	47	3315.142	6.637	3.1				
9	3841.544	7.690	0.6	48	3312.029	6.630	86.4				
10	3835.135	7.678	1.4	49	3309.832	6.626	84.1				
11	3833.854	7.675	1.8	50	3306.536	6.619	1.5				
12	3829.643	7.667	150.0	51	3304.339	6.615	0.9				
13	3825.431	7.658	2.9	52	3112.637	6.231	1.7				
14	3821.403	7.650	124.5	53	3046.723	6.099	1.8				
15	3817.009	7.641	1.7	54	3026.216	6.058	495.9				
16	3815.727	7.639	1.4	55	3014.498	6.035	1.3				
17	3801.079	7.609	-0.6	56	3006.075	6.018	-2.0				
18	3745.052	7.497	0.9	57	3002.413	6.011	0.8				
19	3680.602	7.368	0.6	58	2937.048	5.880	1.8				
20	3660.278	7.327	135.2	59	1673.500	3.350	0.8				
21	3658.447	7.324	131.5	60	1662.698	3.329	4.0				
22	3640.138	7.287	-0.6	61	1633.036	3.269	0.7				
23	3637.941	7.283	-0.6	62	1613.811	3.231	2.8				
24	3488.717	6.984	0.6	63	1613.445	3.230	2.8				
25	3486.703	6.980	0.6	64	1612.346	3.228	-0.7				
26	3463.633	6.934	0.6	65	1593.304	3.190	762.2				
27	3455.211	6.917	0.6	66	1581.586	3.166	2.8				
28	3447.154	6.901	1.7	67	1573.164	3.149	-2.3				
29	3443.309	6.893	138.1	68	1569.319	3.142	2.0				
30	3439.464	6.885	3.2	69	1564.375	3.132	0.9				
31	3438.732	6.884	3.2	70	1563.459	3.130	0.9				
32	3435.070	6.877	157.5	71	1560.347	3.124	0.8				
33	3431.225	6.869	1.9	72	1553.755	3.110	1.0				
34	3430.309	6.867	1.5	73	1549.178	3.101	0.6				
35	3426.648	6.860	0.9	74	1543.502	3.090	1.1				
36	3424.450	6.855	0.7	75	1521.347	3.046	4.0				
37	3423.169	6.853	-0.6	76	1257.505	2.517	8.1				
38	3414.746	6.836	-0.6	77	1255.675	2.514	18.6				
39	3406.324	6.819	137.6	78	1253.844	2.510	26.3				

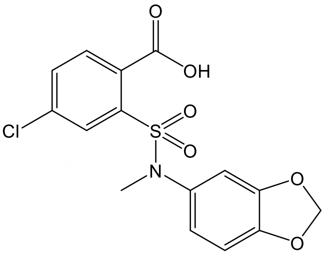

14

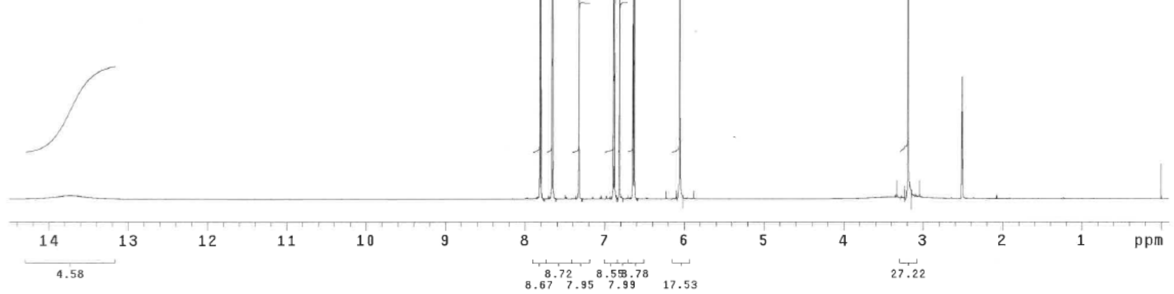

STANDARD CARBON PARAMETERS
56386
* 1800-BGE
Berecz Gabor
2005/01/19 (SzA)

Solvent: DMSO
Temp. 25.0 C / 298.1 K
User: 1-14-87
INOVA-500 "i500"

PULSE SEQUENCE
Relax. delay 3.000 sec
Pulse 45.0 degrees
Acq. time 1.300 sec
Width 32000.0 Hz
928 repetitions
OBSERVE C13, 125.6061563 MHz
DECOUPLE H1, 499.5293374 MHz
Power 32 dB
continuously on
WALTZ-16 modulated
DATA PROCESSING
Line broadening 0.5 Hz
FT size 131072
Total time 66 minutes

INDEX	FREQUENCY	PPM	HEIGHT
1	21106.880	168.033	115.0
2	18543.891	147.629	98.5
3	18454.536	146.918	110.3
4	17045.844	135.703	84.4
5	16874.946	134.343	114.2
6	16841.743	134.078	85.5
7	16816.352	133.876	54.4
8	16739.204	133.262	109.5
9	16357.368	130.222	101.7
10	16185.005	128.850	104.6
11	15199.165	121.002	127.1
12	13654.243	108.702	142.1
13	13590.766	108.197	138.0
14	12803.657	101.931	125.7
15	5049.751	40.201	46.2
16	5028.755	40.034	140.6
17	5007.758	39.867	282.5
18	4986.762	39.700	333.3
19	4965.766	39.533	282.9
20	4944.770	39.366	140.7
21	4923.774	39.199	47.5
22	4915.962	39.136	193.7

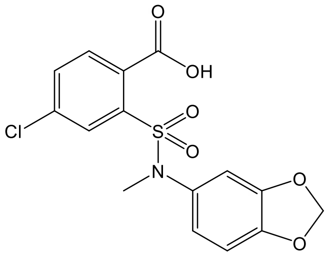

14

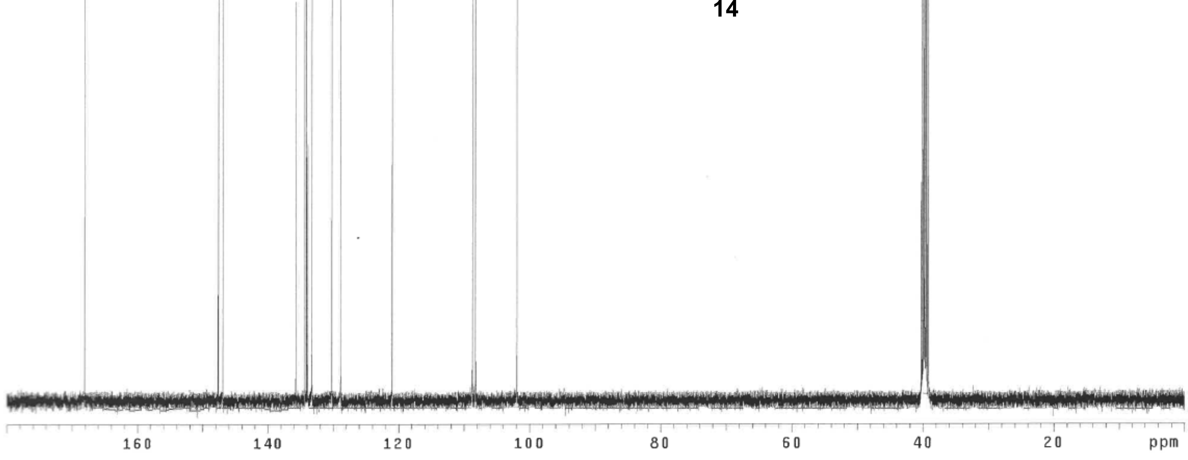

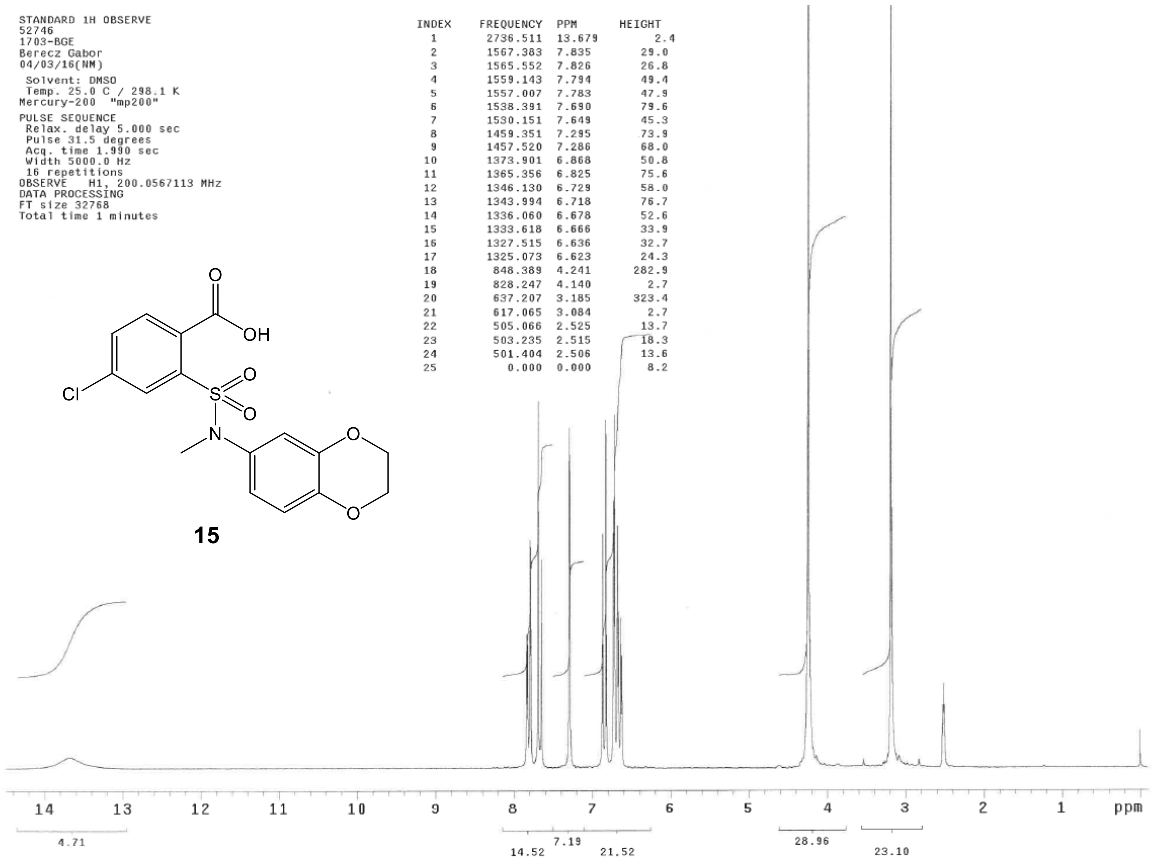

STANDARD 1H OBSERVE
52746
1703-BGE
Berecz Gabor
04/03/16(NM)
Solvent: DMSO
Temp. 25.0 C / 298.1 K
Mercury-200 "mp200"
PULSE SEQUENCE
Relax. delay 5.000 sec
Pulse 31.5 degrees
Acq. time 1.990 sec
Width 5000.0 Hz
16 repetitions
OBSERVE H1, 200.0567113 MHz
DATA PROCESSING
FT size 32768
Total time 1 minutes

INDEX	FREQUENCY	PPM	HEIGHT
1	2736.511	13.679	2.4
2	1567.383	7.835	29.0
3	1565.552	7.826	26.8
4	1559.143	7.794	49.4
5	1557.007	7.783	47.9
6	1538.391	7.690	79.6
7	1530.151	7.649	45.3
8	1459.351	7.295	73.9
9	1457.520	7.286	68.0
10	1373.901	6.868	50.8
11	1365.356	6.825	75.6
12	1346.130	6.729	58.0
13	1343.994	6.718	76.7
14	1336.060	6.678	52.6
15	1333.618	6.666	33.9
16	1327.515	6.636	32.7
17	1325.073	6.623	24.3
18	848.389	4.241	282.9
19	828.247	4.140	2.7
20	637.207	3.185	323.4
21	617.065	3.084	2.7
22	505.066	2.525	13.7
23	503.235	2.515	18.3
24	501.404	2.506	13.6
25	0.000	0.000	8.2

15

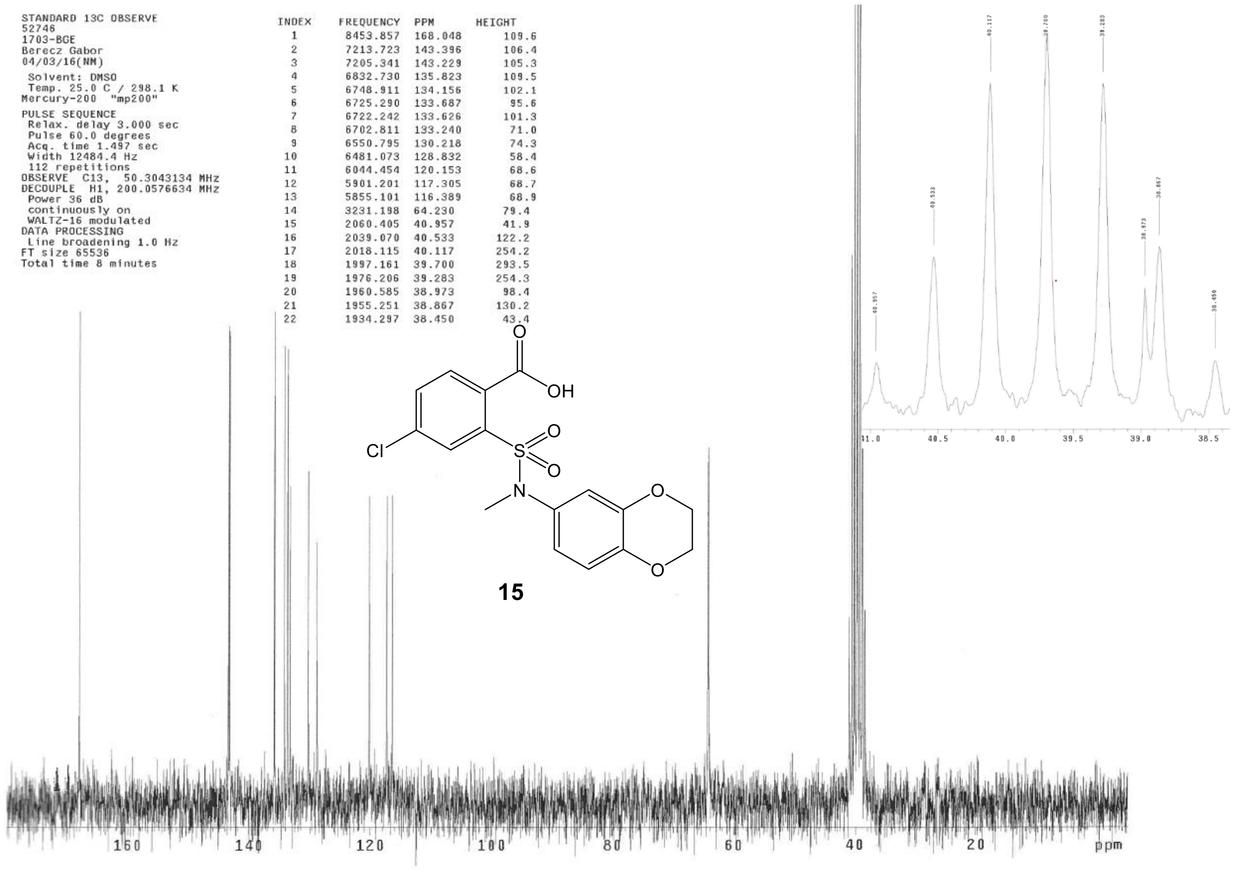

STANDARD 13C OBSERVE
52746
1703-BGE
Berecz Gabor
04/03/16(NM)
Solvent: DMSO
Temp. 25.0 C / 298.1 K
Mercury-200 "mp200"
PULSE SEQUENCE
Relax. delay 3.000 sec
Pulse 60.0 degrees
Acq. time 1.497 sec
Width 12484.4 Hz
112 repetitions
OBSERVE C13, 50.3043134 MHz
DECOUPLE H1, 200.0576634 MHz
Power 36 dB
continuously on
WALTZ-16 modulated
DATA PROCESSING
Line broadening 1.0 Hz
FT size 65536
Total time 8 minutes

INDEX	FREQUENCY	PPM	HEIGHT
1	8453.857	168.048	109.6
2	7213.723	143.396	106.4
3	7205.341	143.229	105.3
4	6832.730	135.823	109.5
5	6748.911	134.156	102.1
6	6725.290	133.687	95.6
7	6722.242	133.626	101.3
8	6702.811	133.240	71.0
9	6550.795	130.218	74.3
10	6481.073	128.832	58.4
11	6044.454	120.153	68.6
12	5901.201	117.305	68.7
13	5855.101	116.389	68.9
14	3231.198	64.230	79.4
15	2060.405	40.957	41.9
16	2039.070	40.533	122.2
17	2018.115	40.117	254.2
18	1997.161	39.700	293.5
19	1976.206	39.283	254.3
20	1960.585	38.973	98.4
21	1955.251	38.867	130.2
22	1934.297	38.450	43.4

15

STANDARD PROTON PARAMETERS
56570
1805-BGE
Berecz Gabor
2005.02.01. (BM)
Solvent: DMSO
Temp. 25.0 C / 298.1 K
INOVA-500 "i500"
PULSE SEQUENCE
Relax. delay 3.000 sec
Pulse 90.0 degrees
Acq. time 5.000 sec
Width 8000.0 Hz
16 repetitions
OBSERVE H1, 499.5268281 MHz
DATA PROCESSING
FT size 131072
Total time 2 minutes

INDEX	FREQUENCY	PPM	HEIGHT
1	3931.152	7.870	27.6
2	3930.542	7.869	25.6
3	3922.485	7.852	33.1
4	3921.875	7.851	32.2
5	3874.268	7.756	41.6
6	3872.070	7.751	47.9
7	3850.342	7.708	30.4
8	3848.145	7.704	25.8
9	3841.919	7.691	25.2
10	3839.600	7.686	22.2
11	3584.351	7.175	105.4
12	3516.724	7.040	74.5
13	3300.781	6.608	39.9
14	3295.776	6.598	40.1
15	3080.200	6.166	24.3
16	3075.317	6.156	23.6
17	3044.067	6.094	62.2
18	3043.091	6.092	60.3
19	3015.259	6.036	64.2
20	3014.282	6.034	58.1
21	1684.448	3.372	277.6
22	1676.025	3.355	31.7
23	1254.639	2.512	6.0
24	1252.808	2.508	8.1
25	1250.977	2.504	5.6
26	-0.000	-0.000	7.4

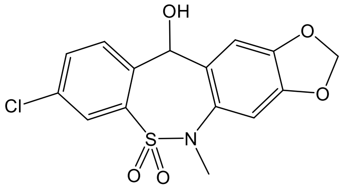

16

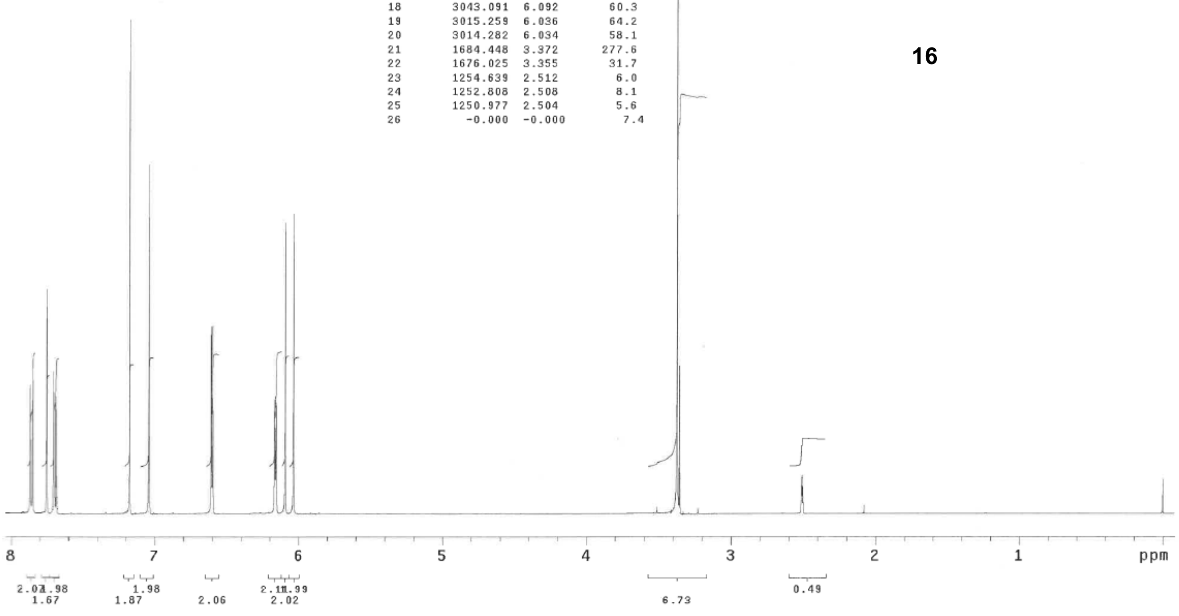

STANDARD 13C PARAMETERS
56570
1805-BGE
Berecz Gabor
2005.02.01. (BM)
Solvent: DMSO
Temp. 25.0 C / 298.1 K
User: 1-14-87
INOVA-500 "i500"
PULSE SEQUENCE
Relax. delay 3.000 sec
Pulse 45.0 degrees
Acq. time 1.300 sec
Width 32000.0 Hz
250 repetitions
OBSERVE C13, 125.6061563 MHz
DECOUPLE H1, 499.5293374 MHz
Power 32 dB
continuously on
WALTZ-16 modulated
DATA PROCESSING
Line broadening 1.0 Hz
FT size 131072
Total time 17 minutes

INDEX	FREQUENCY	PPM	HEIGHT
1	18537.056	147.575	49.3
2	18506.294	147.330	44.9
3	17634.224	140.387	41.2
4	17562.935	139.820	43.1
5	17376.899	138.339	45.0
6	16685.493	132.834	43.9
7	16595.161	132.115	55.3
8	16204.536	129.006	45.4
9	16086.860	128.069	53.5
10	15910.103	126.662	55.6
11	13653.755	108.699	58.6
12	13202.095	105.103	55.1
13	12831.001	102.149	54.5
14	8479.439	67.505	55.6
15	5051.216	40.213	21.1
16	5030.220	40.046	65.7
17	5009.224	39.879	130.8
18	4988.228	39.712	154.6
19	4967.232	39.545	130.4
20	4946.235	39.377	64.5
21	4925.239	39.210	20.7
22	4672.310	37.197	75.3

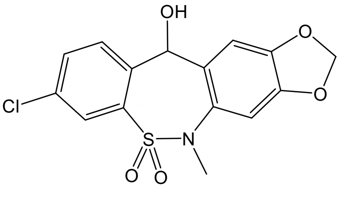

16

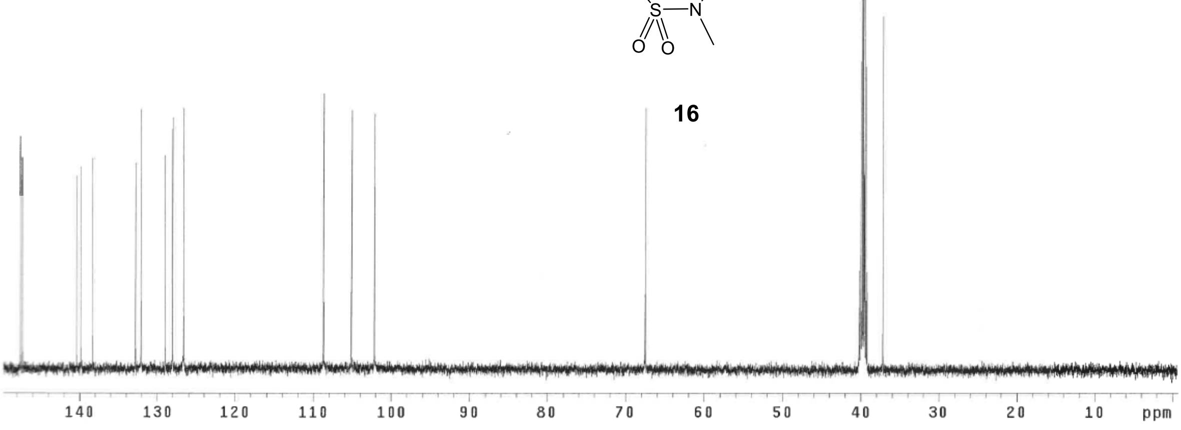

STANDARD 1H OBSERVE
53986
1733-BGE
Berecz Gabor
04/06/07(NM)
Solvent: DMSO
Temp. 25.0 C / 298.1 K
Mercury-200 "mp200"
PULSE SEQUENCE
Relax. delay 5.000 sec
Pulse 31.5 degrees
Acq. time 1.993 sec
Width 3003.0 Hz
16 repetitions
OBSERVE H1, 200.0567147 MHz
DATA PROCESSING
FT size 16384
Total time 1 minutes

INDEX	FREQUENCY	PPM	HEIGHT
1	1574.450	7.870	19.7
2	1566.019	7.828	34.9
3	1550.623	7.751	33.4
4	1548.790	7.742	59.0
5	1545.857	7.727	48.0
6	1543.658	7.716	20.6
7	1537.426	7.685	21.5
8	1535.227	7.674	15.6
9	1410.590	7.051	121.6
10	1404.725	7.022	63.0
11	1403.992	7.018	57.2
12	1308.682	6.542	15.9
13	1305.383	6.525	16.3
14	1221.070	6.104	15.1
15	1218.504	6.091	14.0
16	848.627	4.242	177.7
17	672.303	3.361	3.0
18	671.203	3.355	4.3
19	668.271	3.340	139.7
20	665.705	3.328	292.5
21	662.039	3.309	4.3
22	504.777	2.523	9.0
23	502.944	2.514	19.3
24	501.111	2.505	25.8
25	499.279	2.496	17.2
26	497.079	2.485	7.3
27	415.332	2.076	8.2
28	0.000	0.000	42.4
29	-0.367	-0.002	-4.0

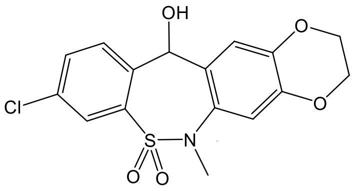

17

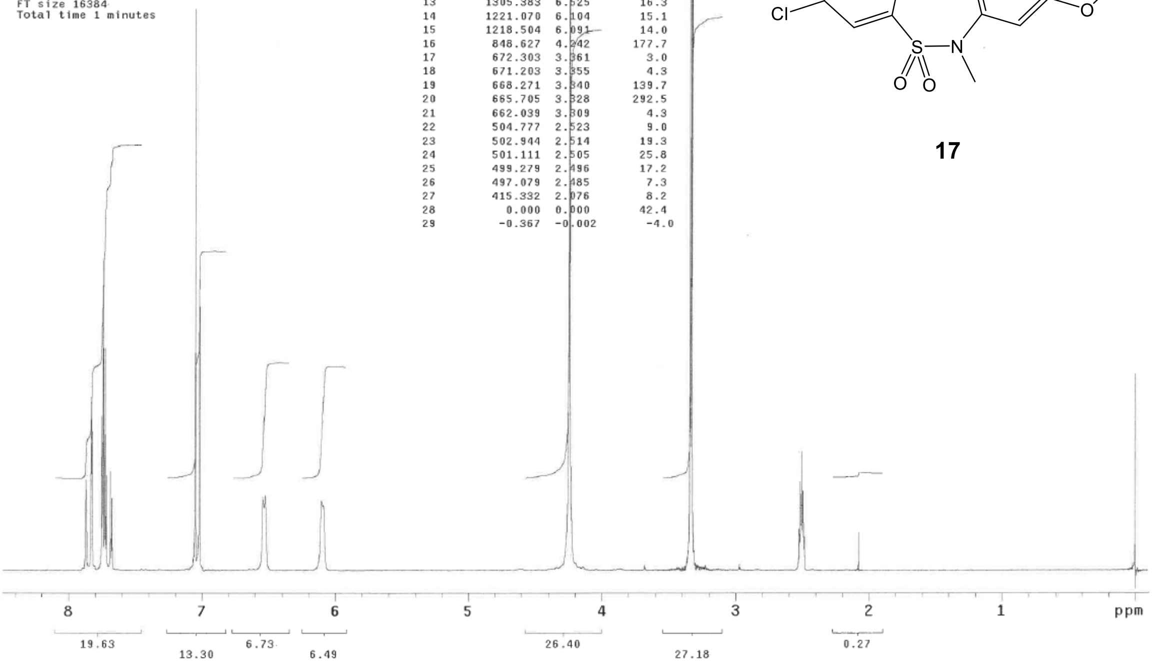

STANDARD 13C OBSERVE
53986
1733-BGE
Berecz Gabor
04/06/07(NM)
Solvent: DMSO
Temp. 25.0 C / 298.1 K
Mercury-200 "mp200"
PULSE SEQUENCE
Relax. delay 3.000 sec
Pulse 60.0 degrees
Acq. time 1.497 sec
Width 12484.4 Hz
1300 repetitions
OBSERVE C13, 50.3043156 MHz
DECOUPLE H1, 200.0576634 MHz
Power 36 dB
continuously on
WALTZ-16 modulated
DATA PROCESSING
Line broadening 1.0 Hz
FT size 65536
Total time 97 minutes

INDEX	FREQUENCY	PPM	HEIGHT
1	7220.200	143.525	97.5
2	7205.342	143.229	87.6
3	7059.802	140.336	114.7
4	7014.464	139.435	100.2
5	6852.161	136.209	119.5
6	6678.809	132.763	116.1
7	6651.758	132.225	76.7
8	6486.788	128.946	90.0
9	6471.929	128.650	70.8
10	6372.490	126.674	77.3
11	5886.342	117.010	68.0
12	5747.660	114.253	68.5
13	3418.266	67.949	75.1
14	3236.151	64.329	60.4
15	3231.580	64.238	58.9
16	2060.025	40.950	88.4
17	2039.070	40.533	264.5
18	2018.115	40.117	527.0
19	1997.161	39.700	619.5
20	1976.206	39.283	524.2
21	1955.251	38.867	264.0
22	1933.916	38.443	85.2
23	1905.722	37.882	88.8

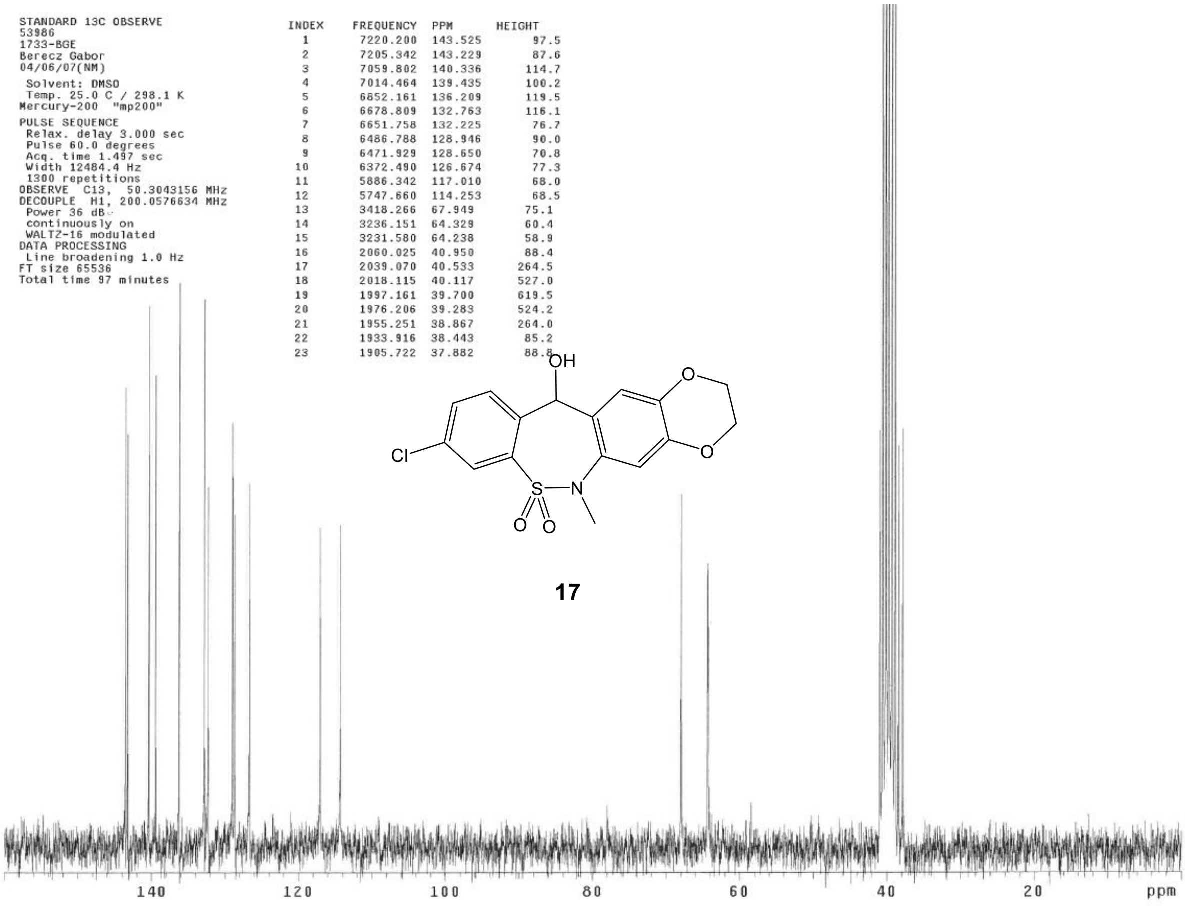

17

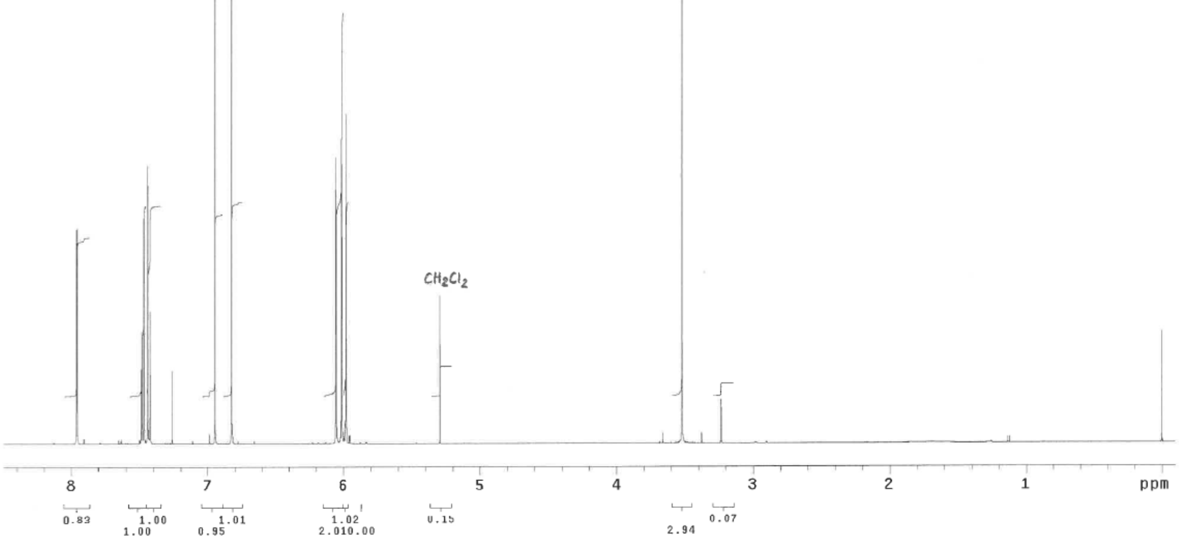

INDEX	FREQUENCY	PPM	HEIGHT
1	3976.929	7.961	45.6
2	3974.854	7.957	45.9
3	3738.770	7.485	23.8
4	3736.572	7.480	22.1
5	3730.469	7.468	48.0
6	3728.271	7.464	49.6
7	3715.698	7.438	59.2
8	3707.397	7.422	28.1
9	3625.854	7.259	15.5
10	3469.482	6.946	115.5
11	3408.203	6.823	106.6
12	3025.146	6.056	54.0
13	3023.804	6.053	61.0
14	3004.761	6.015	62.1
15	3003.418	6.013	52.3
16	2988.281	5.982	70.1
17	2643.433	5.292	31.5
18	1758.667	3.521	423.4
19	1614.990	3.233	9.3
20	0.000	0.000	23.8

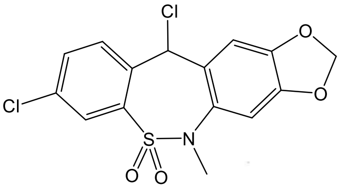

18

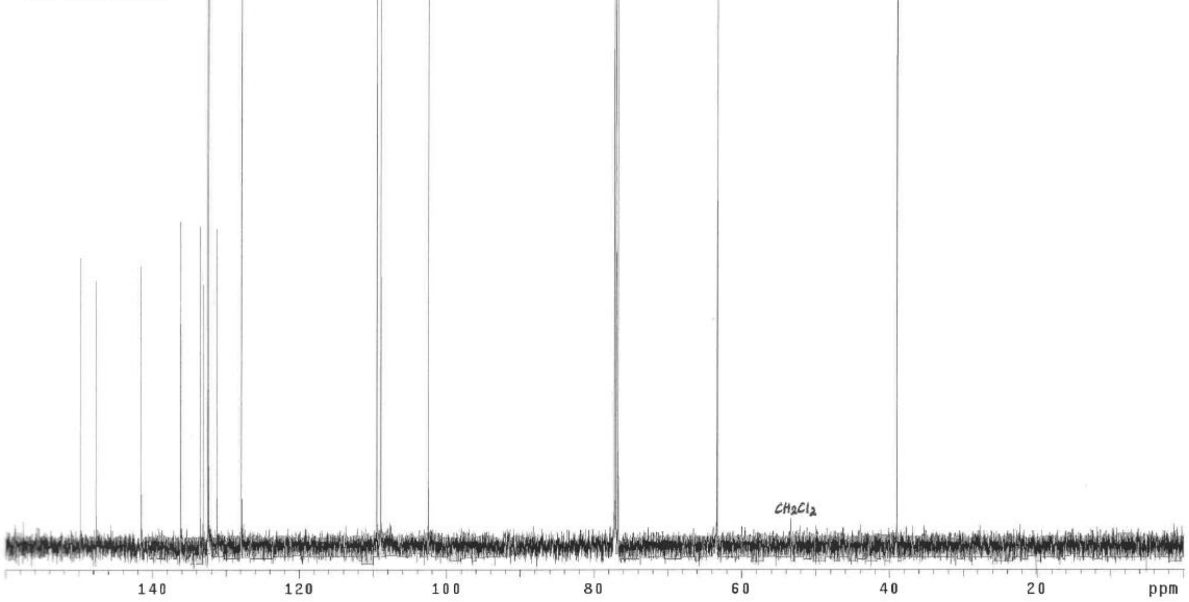

INDEX	FREQUENCY	PPM	HEIGHT
1	18814.461	149.779	61.2
2	18547.371	147.652	56.3
3	17774.910	141.503	59.4
4	17104.988	136.170	68.8
5	16774.422	133.538	67.9
6	16721.199	133.114	55.4
7	16642.586	132.489	137.7
8	16623.054	132.333	153.2
9	16479.011	131.186	67.2
10	16065.437	127.894	156.0
11	13756.355	109.512	162.1
12	13688.484	108.972	166.5
13	12879.402	102.531	168.2
14	9704.597	77.257	168.8
15	9672.371	77.000	166.9
16	9640.633	76.747	169.6
17	7951.668	63.302	121.4
18	4896.980	38.984	122.7

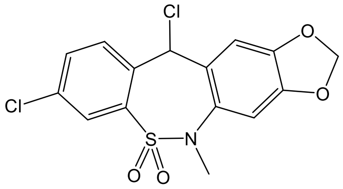

18

STANDARD 1H OBSERVE
54080
1734-BGE
Berecz Gabor
04/06/10(NM)

Solvent: CDCl3
Temp. 25.0 C / 298.1 K
Mercury-200 "mp200"

PULSE SEQUENCE
Relax. delay 5.000 sec
Pulse 31.5 degrees
Acq. time 1.993 sec
Width 3003.0 Hz
16 repetitions
OBSERVE H1, 200.0557681 MHz
DATA PROCESSING
FT size 16384
Total time 1 minutes

INDEX	FREQUENCY	PPM	HEIGHT
1	1595.712	7.976	20.7
2	1593.879	7.967	22.0
3	1502.601	7.511	5.9
4	1500.402	7.500	5.7
5	1494.170	7.469	36.0
6	1491.970	7.458	29.7
7	1489.771	7.447	35.0
8	1481.340	7.405	5.7
9	1452.380	7.260	21.7
10	1404.358	7.020	58.8
11	1380.897	6.903	53.9
12	1197.242	5.985	36.3
13	1059.042	5.294	11.2
14	857.791	4.288	3.9
15	856.325	4.280	6.3
16	852.659	4.262	134.8
17	850.093	4.249	10.3
18	848.994	4.244	5.3
19	847.527	4.236	3.6
20	708.228	3.540	2.2
21	706.395	3.531	4.6
22	704.562	3.522	237.6
23	703.462	3.516	10.6
24	701.996	3.509	4.3
25	700.530	3.502	2.2
26	629.780	3.148	7.7
27	0.000	0.000	24.3

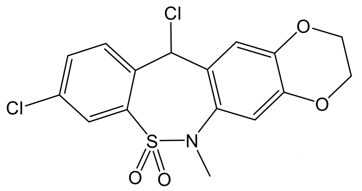

19

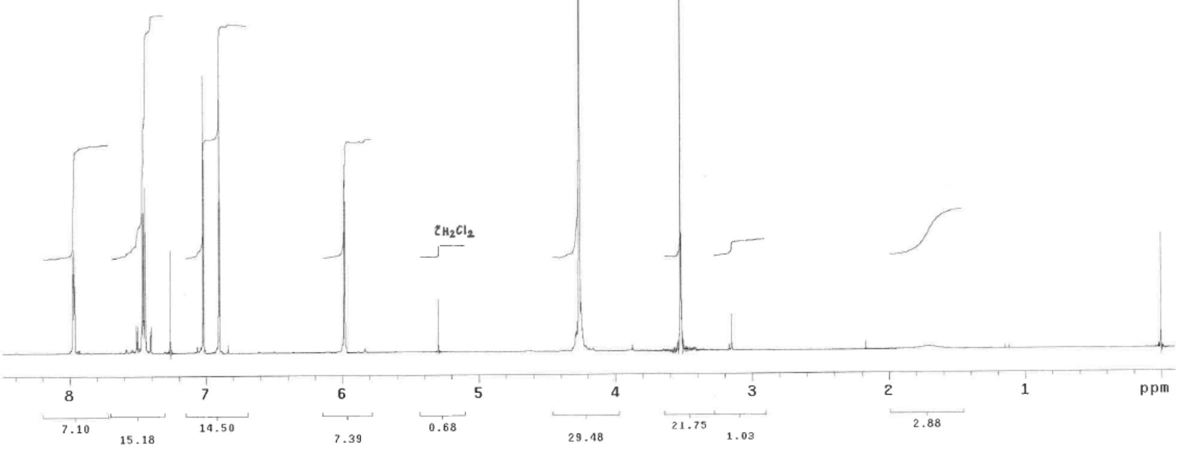

STANDARD 13C OBSERVE
54080
1734-BGE
Berecz Gabor
04/06/10(NM)

Solvent: CDCl3
Temp. 25.0 C / 298.1 K
Mercury-200 "mp200"

PULSE SEQUENCE
Relax. delay 3.000 sec
Pulse 60.0 degrees
Acq. time 1.497 sec
Width 12484.4 Hz
265 repetitions
OBSERVE C13, 50.3040643 MHz
DECOUPLE H1, 200.0567131 MHz
Power 36 dB
continuously on
WALTZ-16 modulated
DATA PROCESSING
Line broadening 1.0 Hz
FT size 65536
Total time 19 minutes

INDEX	FREQUENCY	PPM	HEIGHT
1	7315.606	145.417	54.8
2	7213.119	143.379	54.5
3	7128.920	141.706	47.6
4	6845.841	136.079	61.0
5	6717.066	133.519	71.2
6	6675.156	132.686	99.7
7	6656.869	132.322	103.2
8	6646.201	132.110	47.7
9	6553.619	130.270	63.9
10	6438.559	127.983	97.3
11	5951.269	118.297	88.4
12	5948.983	118.251	81.2
13	3905.715	77.636	256.9
14	3873.711	77.000	263.2
15	3841.708	76.364	249.8
16	3235.928	64.322	78.6
17	3232.499	64.254	80.9
18	3189.066	63.391	84.9
19	1974.840	39.255	77.8

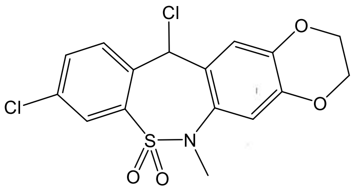

19

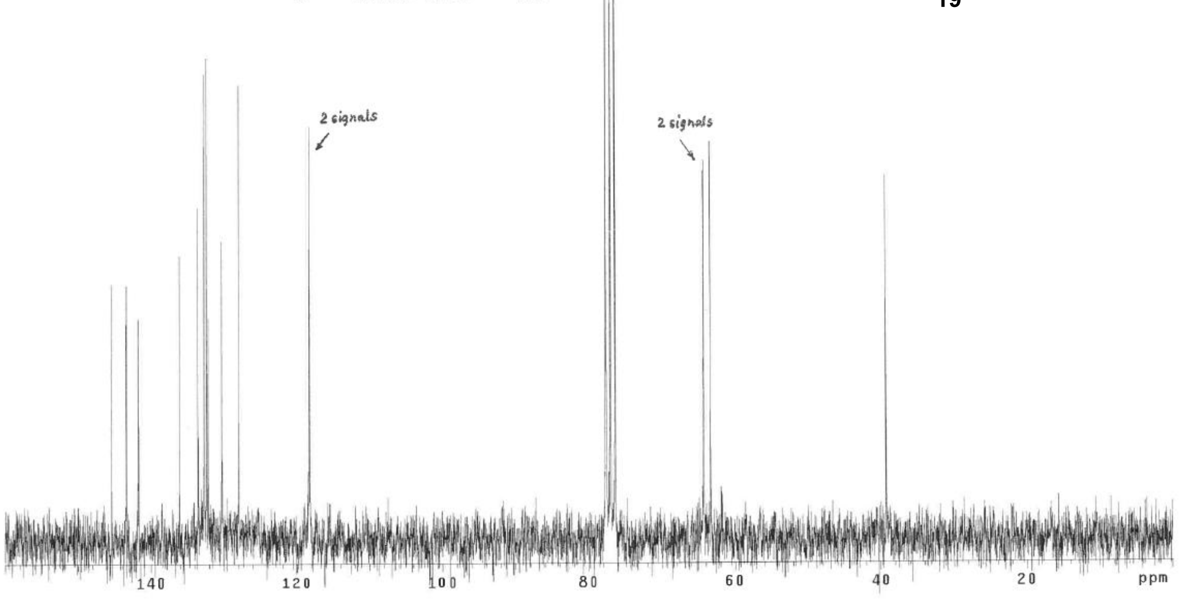

STANDARD 1H
63089
1955-BGE
Berecz Gabor
2006.04.20. (BT)
Solvent: CDCl3
Temp. 25.0 C / 298.1 K
INOVA-500 "i500"
PULSE SEQUENCE
Relax. delay 3.000 sec
Pulse 6.4 usec
Acq. time 5.000 sec
Width 8000.0 Hz
16 repetitions
OBSERVE H1, 499.5244640 MHz
DATA PROCESSING
FT size 131072
Total time 2 minutes

INDEX	FREQUENCY	PPM	HEIGHT
1	3954.712	7.917	22.0
2	3953.003	7.914	21.9
3	3705.688	7.418	30.1
4	3703.735	7.415	45.5
5	3633.179	7.273	7.5
6	3431.885	6.870	55.3
7	3394.409	6.795	49.1
8	2986.694	5.979	31.8
9	2985.352	5.976	36.9
10	2981.201	5.968	39.9
11	2979.858	5.965	25.7
12	2646.118	5.297	13.3
13	2645.264	5.296	31.6
14	2464.600	4.934	17.9
15	2065.674	4.135	14.0
16	2058.472	4.121	43.4
17	2051.392	4.107	43.8
18	2044.189	4.092	14.4
19	1688.110	3.379	148.5
20	1253.784	2.510	12.8
21	1246.582	2.496	25.3
22	1239.502	2.481	14.1
23	1143.311	2.289	21.8
24	1135.864	2.274	40.7
25	1128.296	2.259	23.6
26	880.859	1.763	18.9
27	809.204	1.620	10.3
28	802.002	1.606	13.8
29	794.800	1.591	9.6
30	757.690	1.517	7.2
31	750.610	1.503	11.9
32	743.530	1.488	9.6
33	661.621	1.325	26.1
34	658.081	1.317	23.0
35	654.419	1.310	22.1
36	651.489	1.304	11.5
37	628.662	1.259	50.4
38	622.314	1.246	47.4
39	621.582	1.244	98.8
40	614.380	1.230	48.9
41	0.000	0.000	7.8

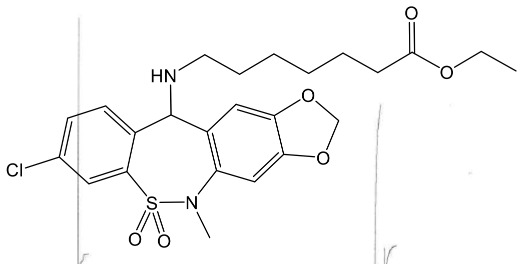

20a

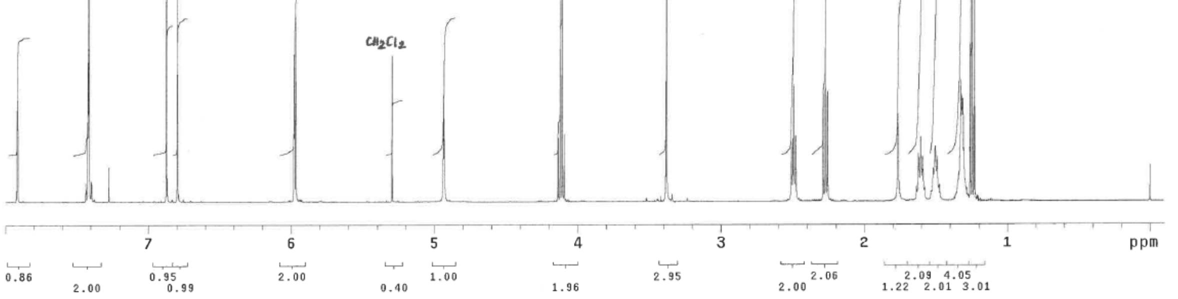

STANDARD 13C
63089
1955-BGE
Berecz Gabor
2006.04.20. (BT)
Solvent: CDCl3
Temp. 25.0 C / 298.1 K
User: 1-14-87
INOVA-500 "i500"
PULSE SEQUENCE
Relax. delay 3.000 sec
Pulse 6.0 usec
Acq. time 1.300 sec
Width 32000.0 Hz
480 repetitions
OBSERVE C13, 125.6055352 MHz
DECOUPLE H1, 499.5269647 MHz
Power 32 dB
continuously on
WALTZ-16 modulated
DATA PROCESSING
Line broadening 0.5 Hz
FT size 131072
Total time 34 minutes

INDEX	FREQUENCY	PPM	HEIGHT	
1	21810.066	173.626	47.7	
2	18575.691	147.878	49.7	
3	18543.465	147.621	46.1	
4	17677.742	140.729	27.6	
5	17258.308	137.390	19.4	
6	16887.215	134.436	8.7	
7	16848.152	134.125	45.9	
8	16565.437	131.874	81.5	
9	16479.012	131.186	23.9	
10	16317.391	129.900	11.0	
11	16111.336	128.259	80.7	
12	13634.285	108.540	74.9	
13	13564.461	107.984	14.1	
14	12807.137	101.955	74.3	
15	9704.598	77.257	117.2	
16	9672.371	77.000	118.0	
17	9640.633	76.747	117.7	
18	8107.918	64.546	4.8	
19	7551.277	60.114	78.3	
20	6706.062	53.386	6.2	← DCM
21	6052.254	48.181	80.0	
22	4790.047	38.133	35.0	
23	4290.047	34.152	117.4	
24	3750.008	29.853	87.2	
25	3629.891	28.897	120.7	
26	3376.473	26.879	114.8	
27	3108.894	24.749	126.0	
28	1781.258	14.180	90.6	

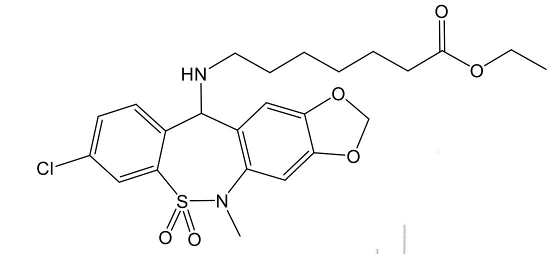

20a

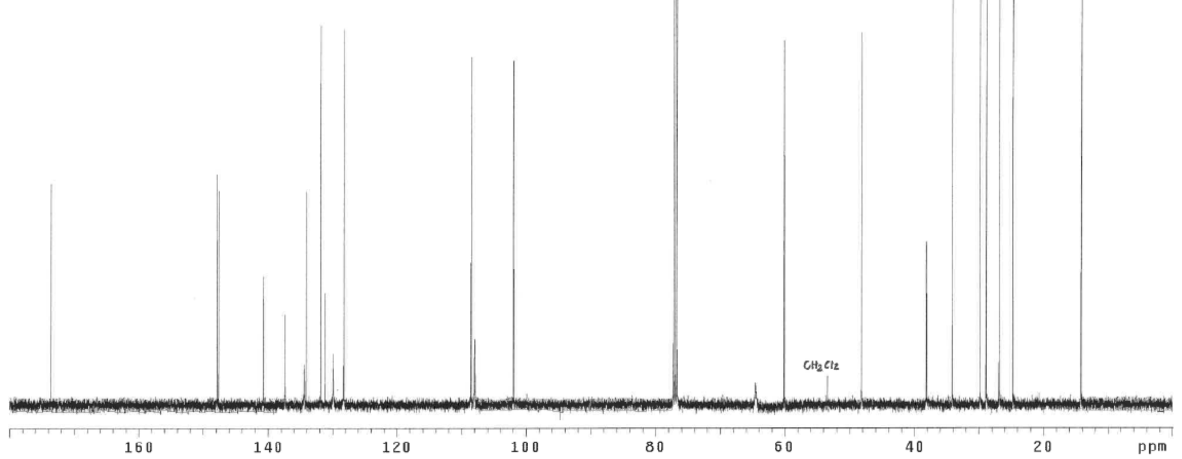

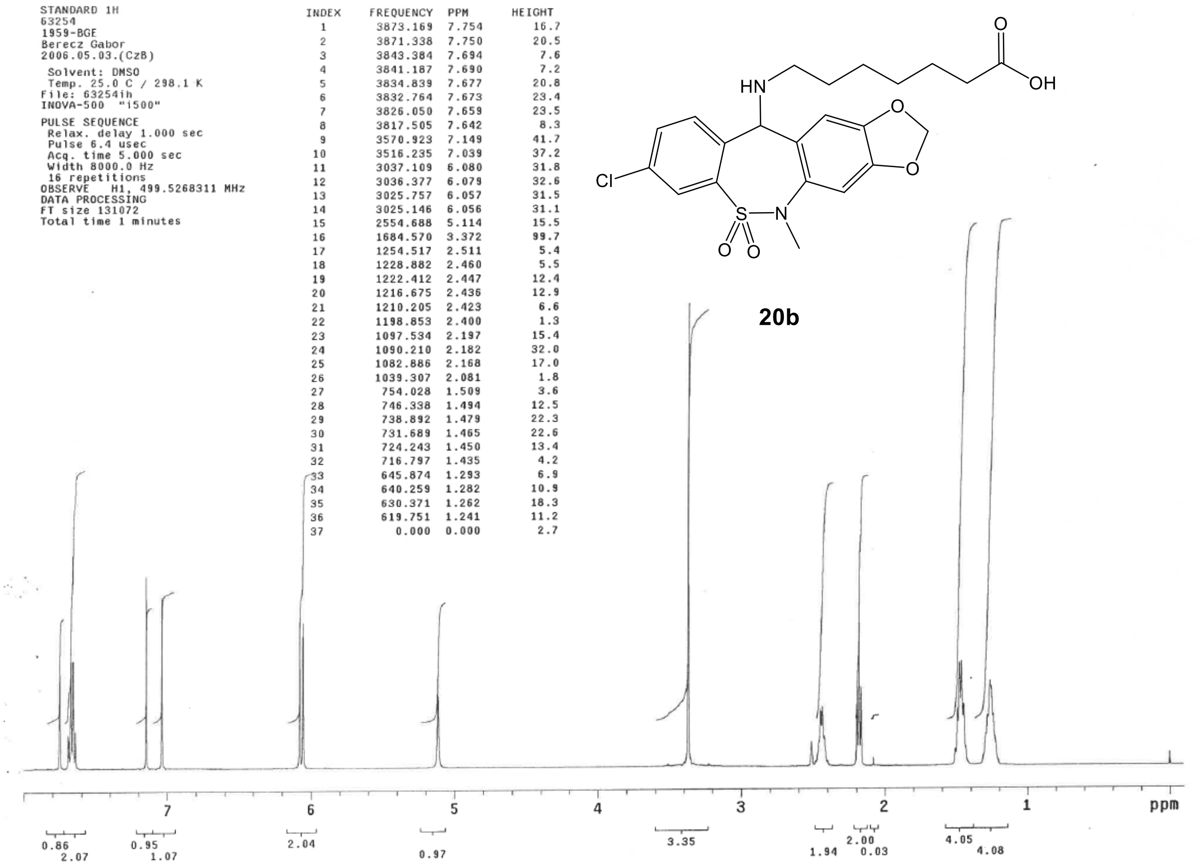
STANDARD 1H
63254
1959-BGE
Berecz Gabor
2006.05.03.(CzB)
Solvent: DMSO
Temp. 25.0 C / 298.1 K
File: 63254ih
INOVA-500 "i500"
PULSE SEQUENCE
Relax. delay 1.000 sec
Pulse 6.4 usec
Acq. time 5.000 sec
Width 8000.0 Hz
16 repetitions
OBSERVE H1, 499.5268311 MHz
DATA PROCESSING
FT size 131072
Total time 1 minutes
INDEX FREQUENCY PPM HEIGHT
1 3873.169 7.754 16.7
2 3871.338 7.750 20.5
3 3843.384 7.694 7.6
4 3841.187 7.690 7.2
5 3834.839 7.677 20.8
6 3832.764 7.673 23.4
7 3826.050 7.659 23.5
8 3817.505 7.642 8.3
9 3570.923 7.149 41.7
10 3516.235 7.039 37.2
11 3037.109 6.080 31.8
12 3036.377 6.079 32.6
13 3025.757 6.057 31.5
14 3025.146 6.056 31.1
15 2554.688 5.114 15.5
16 1684.570 3.372 99.7
17 1254.517 2.511 5.4
18 1228.882 2.460 5.5
19 1222.412 2.447 12.4
20 1216.675 2.436 12.9
21 1210.205 2.423 6.6
22 1198.853 2.400 1.3
23 1097.534 2.197 15.4
24 1090.210 2.182 32.0
25 1082.886 2.168 17.0
26 1039.307 2.081 1.8
27 754.028 1.509 3.6
28 746.338 1.494 12.5
29 738.892 1.479 22.3
30 731.689 1.465 22.6
31 724.243 1.450 13.4
32 716.797 1.435 4.2
33 645.874 1.293 6.9
34 640.259 1.282 10.9
35 630.371 1.262 18.3
36 619.751 1.241 11.2
37 0.000 0.000 2.7
O
HN
OH
O
O
Cl
S
N
O O
20b
7
6
5
4
3
2
1
ppm
0.86
2.07
0.95
1.07
2.04
0.97
3.35
1.94
2.00
0.03
4.05
4.08

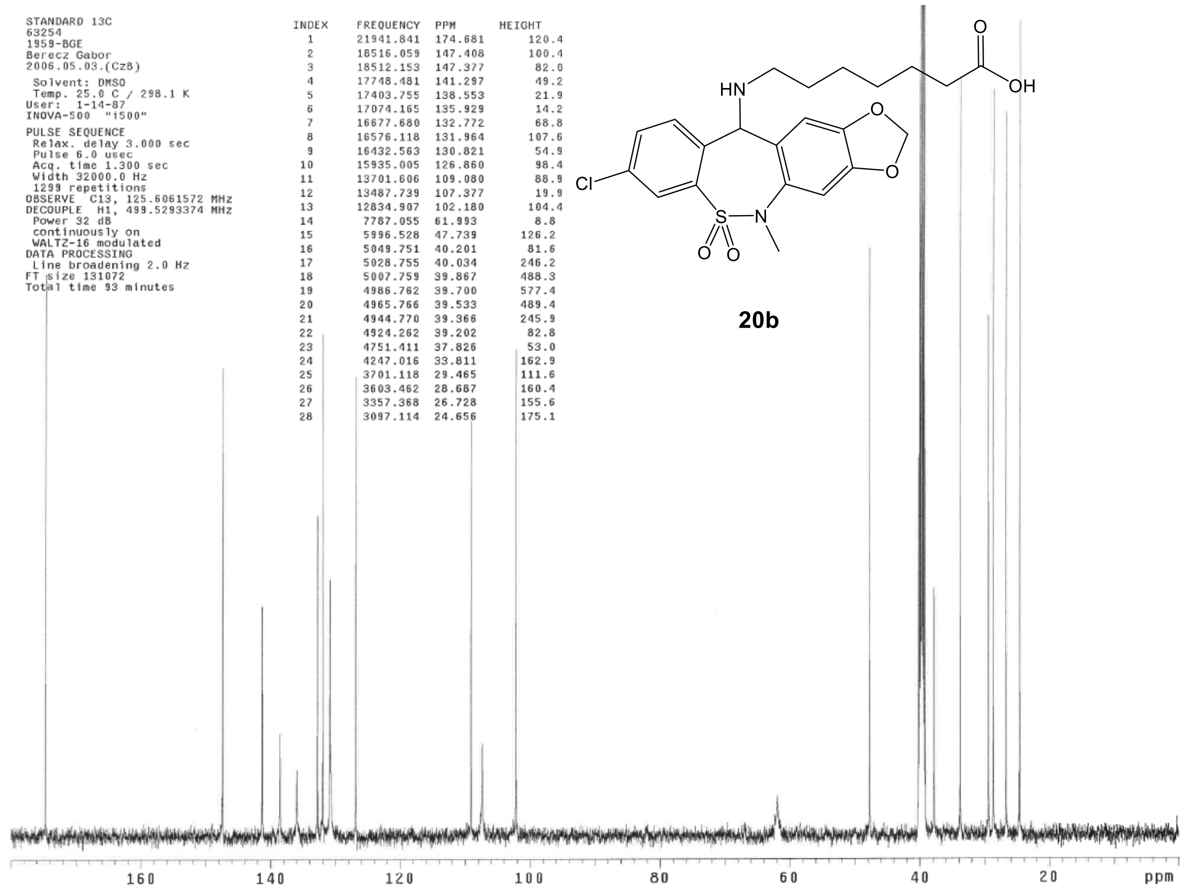
STANDARD 13C
63254
1959-BGE
Berecz Gabor
2006.05.03.(CzB)
Solvent: DMSO
Temp. 25.0 C / 298.1 K
User: 1-14-87
INOVA-500 "i500"
PULSE SEQUENCE
Relax. delay 3.000 sec
Pulse 6.0 usec
Acq. time 1.300 sec
Width 32000.0 Hz
1299 repetitions
OBSERVE C13, 125.6061572 MHz
DECOUPLE H1, 499.5293374 MHz
Power 32 dB
continuously on
WALTZ-16 modulated
DATA PROCESSING
Line broadening 2.0 Hz
FT size 131072
Total time 93 minutes
INDEX FREQUENCY PPM HEIGHT
1 21941.841 174.681 120.4
2 18516.059 147.408 100.4
3 18512.153 147.377 82.0
4 17748.481 141.297 49.2
5 17403.755 138.553 21.9
6 17074.165 135.929 14.2
7 16677.680 132.772 68.8
8 16576.118 131.964 107.6
9 16432.563 130.821 54.9
10 15935.005 126.860 98.4
11 13701.606 109.080 88.9
12 13487.739 107.377 19.9
13 12834.907 102.180 104.4
14 7787.055 61.993 8.8
15 5996.528 47.739 126.2
16 5049.751 40.201 81.6
17 5028.755 40.034 246.2
18 5007.759 39.867 488.3
19 4986.762 39.700 577.4
20 4965.766 39.533 489.4
21 4944.770 39.366 245.9
22 4924.262 39.202 82.8
23 4751.411 37.826 53.0
24 4247.016 33.811 162.9
25 3701.118 29.465 111.6
26 3603.462 28.687 160.4
27 3357.368 26.728 155.6
28 3097.114 24.656 175.1
O
HN
OH
O
O
Cl
S
N
O O
20b
160
140
120
100
80
60
40
20
ppm

STANDARD 1H
59563
1887-BGE
Berecz Gabor
2005.09.12. (CzB)
Solvent: CDCl3
Temp. 25.0 C / 298.1 K
INOVA-500 "i500"
PULSE SEQUENCE
Relax. delay 3.000 sec
Pulse 6.4 usec
Acq. time 5.000 sec
Width 8000.0 Hz
16 repetitions
OBSERVE H1, 499.5244691 MHz
DATA PROCESSING
FT size 131072
Total time 2 minutes

INDEX	FREQUENCY	PPM	HEIGHT
1	3960.571	7.929	22.2
2	3958.496	7.925	21.6
3	3717.529	7.442	7.6
4	3715.454	7.438	6.8
5	3709.106	7.425	23.5
6	3707.153	7.421	23.7
7	3701.294	7.410	34.6
8	3692.993	7.393	10.9
9	3629.150	7.265	6.9
10	3431.152	6.869	47.8
11	3399.902	6.806	48.8
12	2987.915	5.982	32.8
13	2986.816	5.979	37.6
14	2981.445	5.969	40.0
15	2980.225	5.966	28.4
16	2406.982	4.819	29.7
17	1681.396	3.366	151.2
18	1186.157	2.375	143.2
19	998.047	1.998	3.4
20	928.711	1.859	5.7
21	928.955	1.860	5.7
22	0.000	0.000	4.2

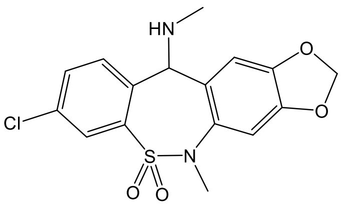

20c

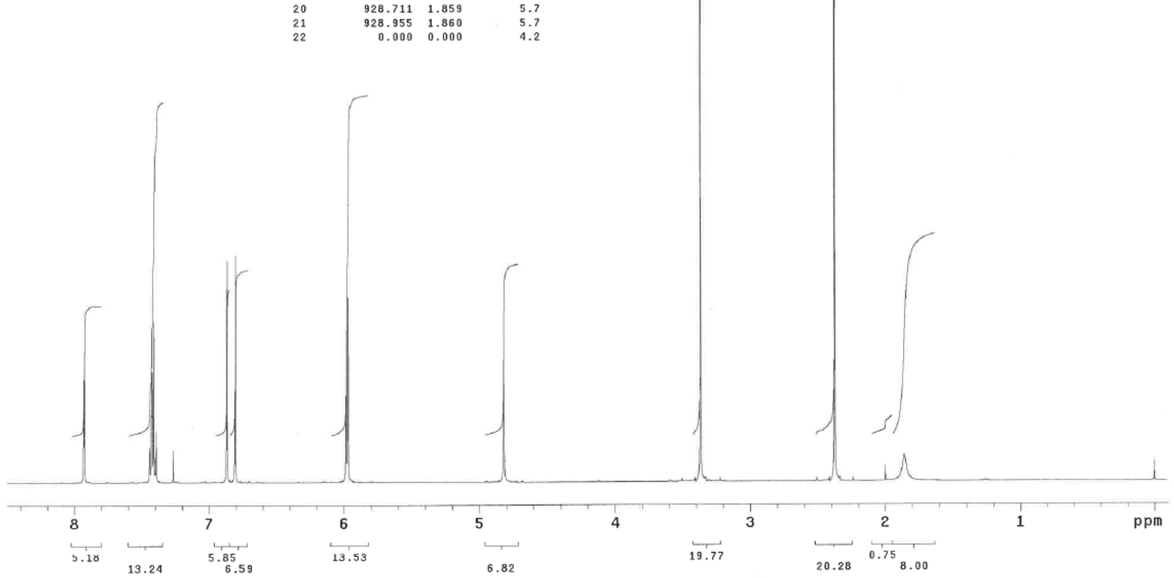

STANDARD 13C
59563
1887-BGE
Berecz Gabor
2005.09.12. (CzB)
Solvent: CDCl3
Temp. 25.0 C / 298.1 K
User: 1-14-87
INOVA-500 "i500"
PULSE SEQUENCE
Relax. delay 3.000 sec
Pulse 6.0 usec
Acq. time 1.300 sec
Width 32000.0 Hz
752 repetitions
OBSERVE C13, 125.6055328 MHz
DECOUPLE H1, 499.5269647 MHz
Power 32 dB
continuously on
WALTZ-16 modulated
DATA PROCESSING
Line broadening 0.5 Hz
FT size 131072
Total time 53 minutes

INDEX	FREQUENCY	PPM	HEIGHT
1	18586.433	147.963	43.6
2	18545.906	147.641	51.9
3	17672.371	140.687	31.4
4	17204.109	136.959	32.4
5	16862.800	134.242	51.1
6	16810.066	133.822	14.0
7	16568.367	131.898	110.4
8	16498.543	131.342	32.1
9	16343.269	130.106	26.0
10	16123.054	128.353	117.5
11	13629.890	108.505	112.6
12	13595.222	108.229	34.8
13	12811.531	101.990	102.8
14	9704.597	77.257	123.3
15	9672.859	77.004	122.4
16	9640.633	76.747	124.2
17	8355.476	66.516	13.9
18	4799.324	38.207	57.4
19	4394.539	34.984	118.7

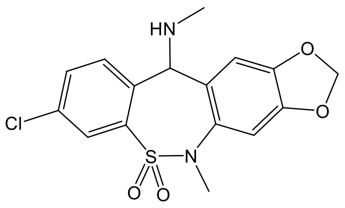

20c

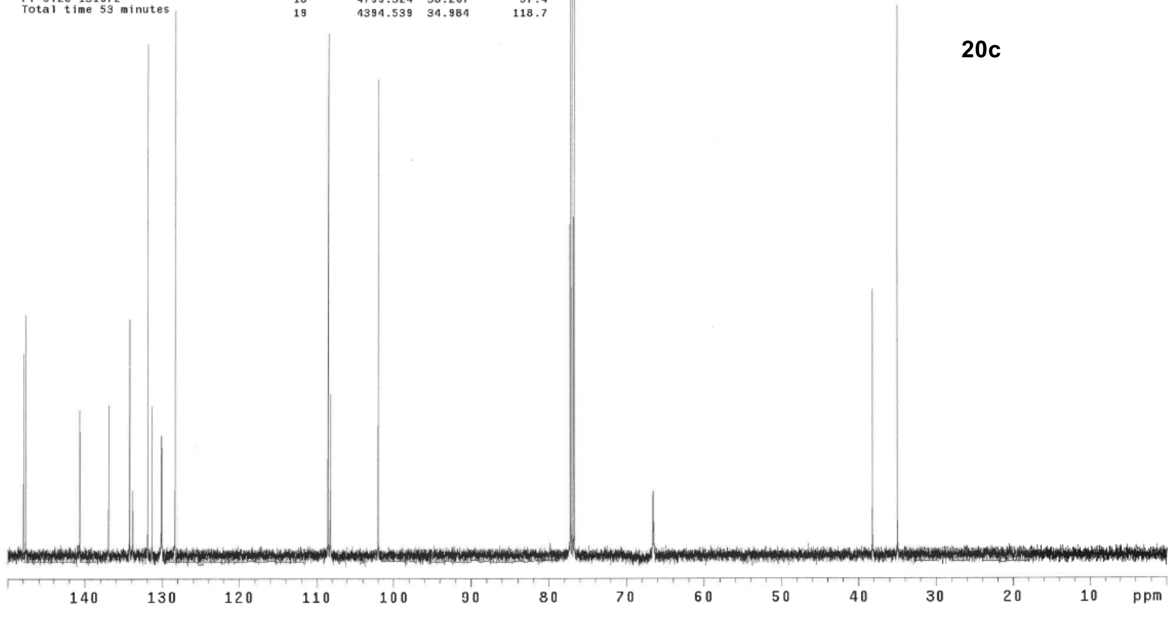

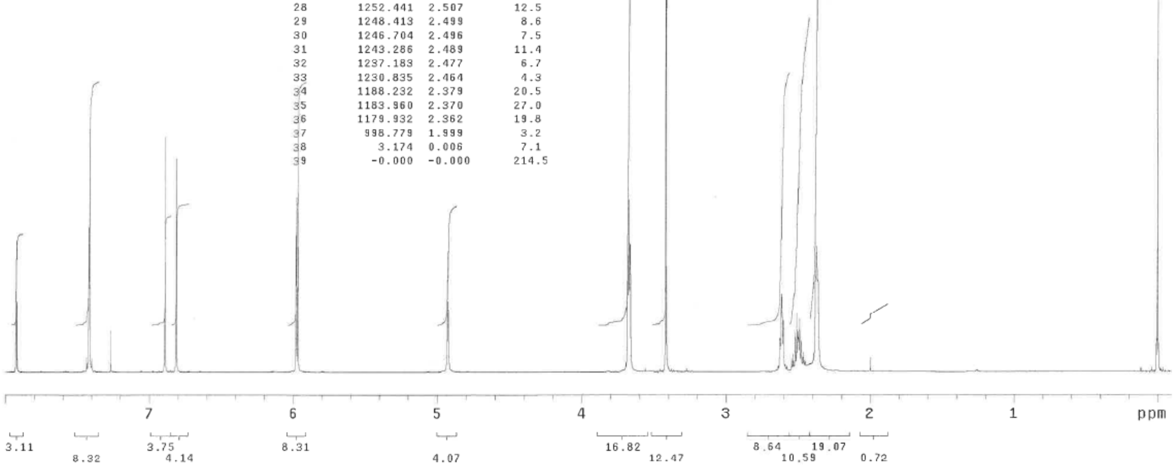

STANDARD 1H
60193
1906-BGE
Berecz Gabor
2005.10.24. (CzB)
Solvent: CDCl3
Temp. 25.0 C / 298.1 K
INOVA-500 "i500"
PULSE SEQUENCE
Relax. delay 3.000 sec
Pulse 6.4 usec
Acq. time 5.000 sec
Width 8000.0 Hz
16 repetitions
OBSERVE H1, 499.5244669 MHz
DATA PROCESSING
FT size 131072
Total time 2 minutes

INDEX	FREQUENCY	PPM	HEIGHT
1	3959.595	7.927	16.4
2	3958.984	7.926	15.2
3	3958.008	7.924	17.2
4	3715.332	7.438	3.1
5	3707.031	7.421	31.1
6	3705.200	7.417	54.2
7	3696.655	7.400	2.8
8	3630.249	7.267	8.9
9	3442.261	6.891	50.5
10	3402.466	6.811	45.7
11	2987.671	5.981	29.4
12	2986.328	5.978	37.4
13	2981.567	5.969	38.3
14	2980.225	5.966	26.8
15	2460.083	4.925	15.0
16	1839.478	3.682	27.2
17	1834.961	3.673	37.0
18	1830.322	3.664	27.4
19	1707.275	3.418	150.3
20	1312.134	2.627	7.3
21	1310.547	2.624	7.2
22	1306.152	2.615	16.7
23	1300.171	2.603	11.1
24	1299.316	2.601	11.0
25	1270.874	2.544	2.9
26	1264.771	2.532	3.8
27	1258.545	2.519	8.5
28	1252.441	2.507	12.5
29	1248.413	2.499	8.6
30	1246.704	2.496	7.5
31	1243.286	2.489	11.4
32	1237.183	2.477	6.7
33	1230.835	2.464	4.3
34	1188.232	2.379	20.5
35	1183.960	2.370	27.0
36	1179.932	2.362	19.8
37	998.779	1.999	3.2
38	3.174	0.006	7.1
39	-0.000	-0.000	214.5

INDEX	FREQUENCY	PPM	HEIGHT
40	-2.563	-0.005	4.3
41	-3.418	-0.007	7.2

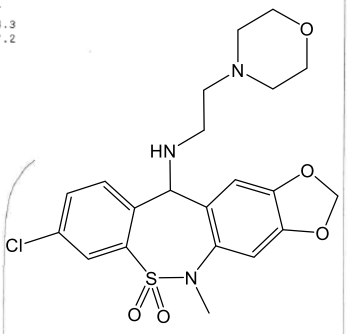

20d

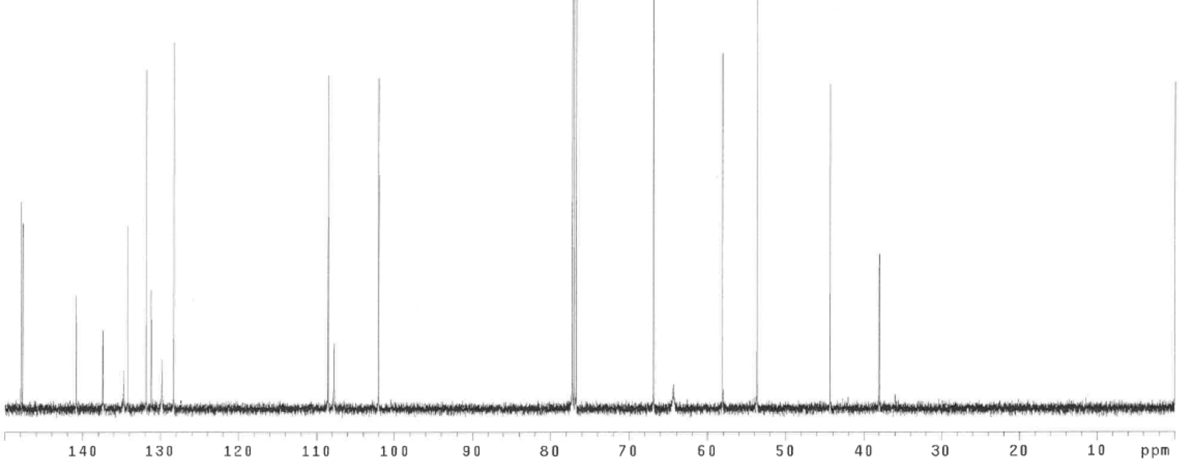

STANDARD 13C
60193
1906-BGE
Berecz Gabor
2005.10.24. (CzB)
Solvent: CDCl3
Temp. 25.0 C / 298.1 K
User: 1-14-87
INOVA-500 "i500"
PULSE SEQUENCE
Relax. delay 3.000 sec
Pulse 6.0 usec
Acq. time 1.300 sec
Width 32000.0 Hz
912 repetitions
OBSERVE C13, 125.6055318 MHz
DECOUPLE H1, 499.5269647 MHz
Power 32 dB
continuously on
WALTZ-16 modulated
DATA PROCESSING
Line broadening 0.5 Hz
FT size 131072
Total time 65 minutes

INDEX	FREQUENCY	PPM	HEIGHT
1	18585.457	147.955	44.1
2	18552.742	147.695	39.4
3	17691.414	140.838	24.1
4	17262.703	137.425	16.8
5	16928.718	134.766	8.2
6	16862.312	134.238	38.9
7	16559.089	131.824	72.4
8	16476.570	131.167	25.3
9	16305.183	129.803	10.5
10	16117.195	128.306	78.1
11	13630.867	108.513	71.4
12	13534.675	107.747	14.0
13	12812.996	102.002	70.6
14	9704.109	77.253	112.9
15	9672.371	77.000	115.7
16	9640.144	76.743	113.7
17	8400.886	66.878	150.9
18	8086.433	64.375	5.1
19	7291.511	58.046	76.0
20	6742.683	53.677	124.5
21	5561.531	44.274	69.4
22	4769.050	37.966	33.0
23	-5.364	-0.043	69.9

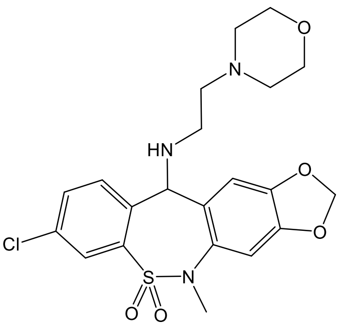

20d

```
STANDARD PROTON PARAMETERS
56634
1807-BGE
Berecz Gabor
2004.02.07. (DA)
 Solvent: CDCl3
 Temp. 25.0 C / 298.1 K
INOVA-500  "i500"
PULSE SEQUENCE
 Pulse 90.0 degrees
 Acq. time 5.000 sec
 Width 8000.0 Hz
 16 repetitions
OBSERVE   H1, 499.5244681 MHz
DATA PROCESSING
FT size 131072
Total time 1 minute
```

INDEX	FREQUENCY	PPM	HEIGHT
1	3958.740	7.925	41.1
2	3956.543	7.921	41.7
3	3680.908	7.369	26.9
4	3678.711	7.364	26.0
5	3672.607	7.352	46.1
6	3670.410	7.348	46.2
7	3649.170	7.305	34.9
8	3640.869	7.289	21.2
9	3627.808	7.263	11.9
10	3441.772	6.890	101.7
11	3370.850	6.748	97.9
12	3002.319	6.010	66.8
13	3000.977	6.008	69.6
14	2973.022	5.952	88.1
15	2971.558	5.949	80.6
16	2023.438	4.051	9.4
17	1798.828	3.601	2.1
18	1749.146	3.502	4.5
19	1729.126	3.462	421.1
20	1657.104	3.317	2.1
21	1131.104	2.264	20.7
22	1093.750	2.190	18.6
23	997.070	1.996	2.5
24	861.328	1.724	3.4
25	853.394	1.708	30.7
26	849.854	1.701	48.7
27	847.046	1.696	91.6
28	844.116	1.690	48.1
29	840.576	1.683	29.5
30	832.886	1.667	3.5
31	799.438	1.600	4.4
32	0.000	0.000	25.6

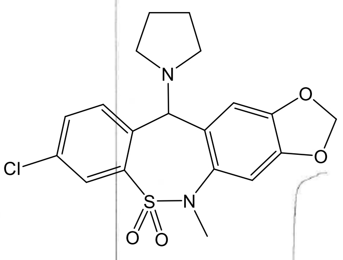

20e

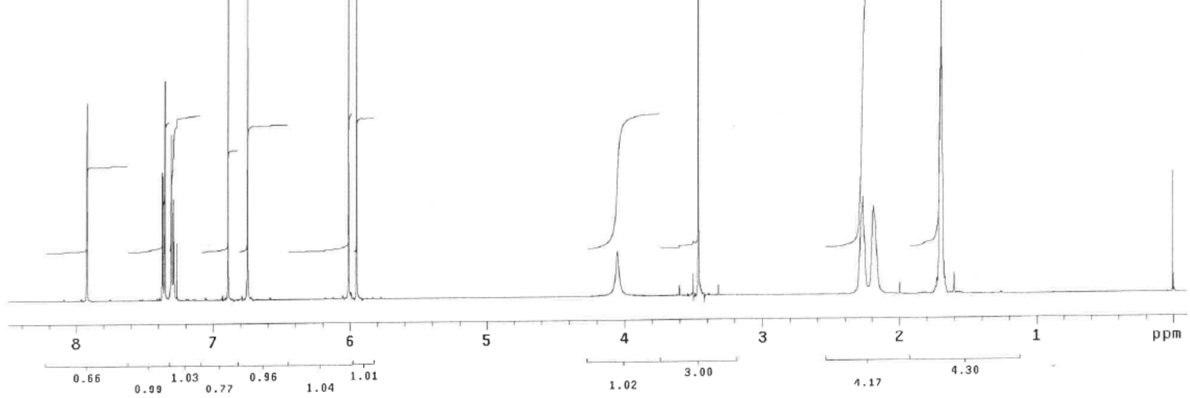


```
STANDARD 13C PARAMETERS
56634
1807-BGE
Berecz Gabor
2004.02.07. (DA)
 Solvent: CDCl3
 Temp. 25.0 C / 298.1 K
User:  1-14-87
INOVA-500  "i500"
PULSE SEQUENCE
 Relax. delay 3.000 sec
 Pulse 45.0 degrees
 Acq. time 1.300 sec
 Width 32000.0 Hz
 1648 repetitions
OBSERVE  C13, 125.6055323 MHz
DECOUPLE  H1, 499.5269647 MHz
 Power 32 dB
 continuously on
 WALTZ-16 modulated
DATA PROCESSING
 Line broadening 1.0 Hz
FT size 131072
Total time 118 minutes
```

INDEX	FREQUENCY	PPM	HEIGHT
1	18615.730	148.196	33.9
2	18545.418	147.637	69.8
3	17933.113	142.762	5.4
4	17051.765	135.746	6.0
5	17023.445	135.521	14.4
6	16942.879	134.879	25.8
7	16656.746	132.601	9.3
8	16478.523	131.183	25.4
9	16463.875	131.066	96.3
10	16106.453	128.221	149.9
11	13898.445	110.643	30.6
12	13734.871	109.341	23.1
13	12819.832	102.056	149.0
14	9704.597	77.257	167.6
15	9672.371	77.000	177.4
16	9640.632	76.747	170.3
17	9545.418	75.989	4.8
18	6746.590	53.708	300.9
19	4971.199	39.575	13.6
20	2912.605	23.187	289.9
21	-4.875	-0.039	5.7

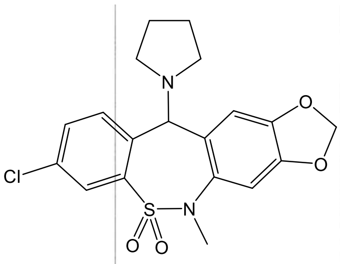

20e

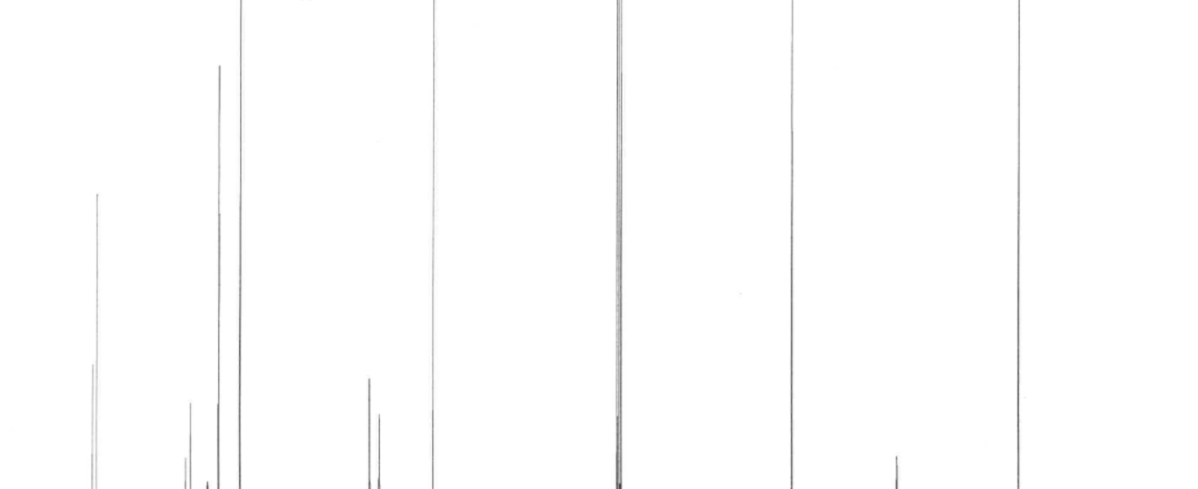

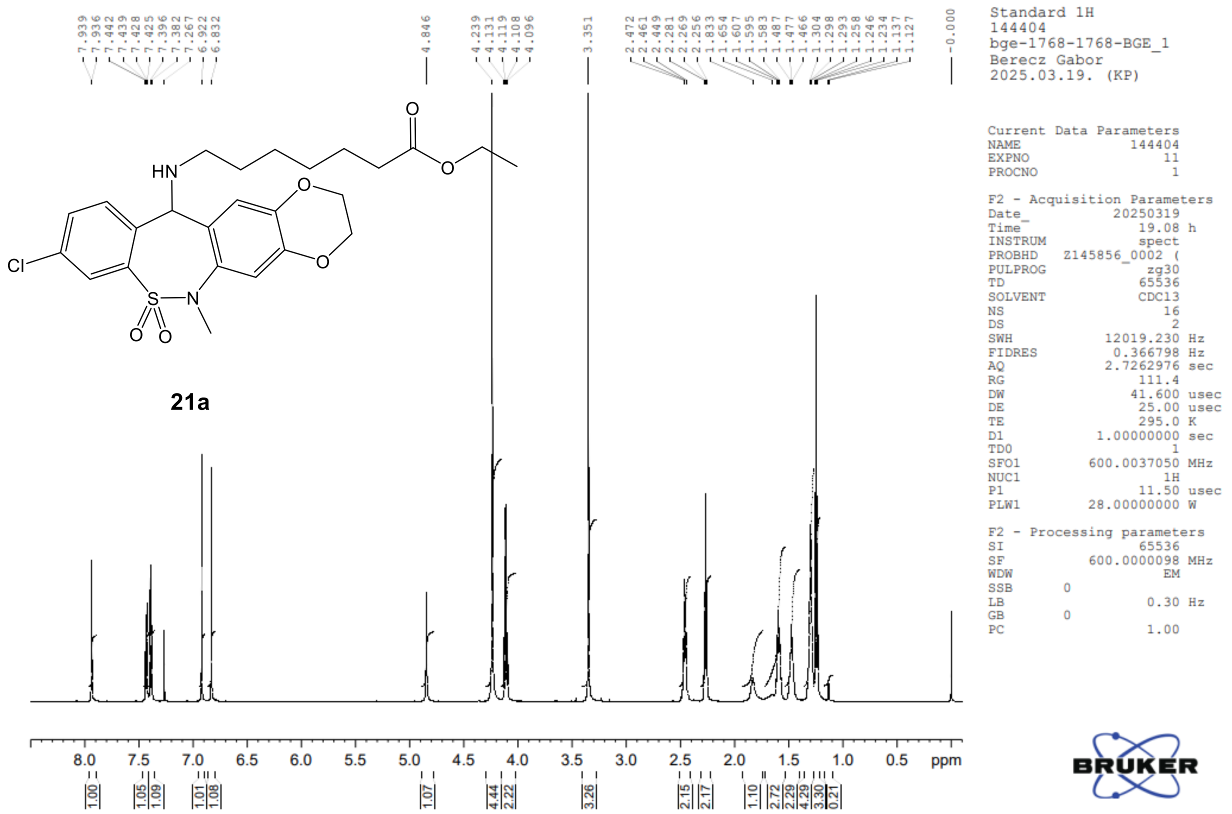
7.939 7.936 7.442 7.439 7.428 7.425 7.396 7.382 7.267 6.922 6.832
4.846
4.239 4.131 4.119 4.108 4.096
3.351
2.472 2.461 2.449 2.281 2.269 2.256 1.833 1.654 1.607 1.595 1.583 1.487 1.477 1.466 1.304 1.298 1.293 1.258 1.246 1.234 1.137 1.127
-0.000
21a
Standard 1H
144404
bge-1768-1768-BGE_1
Berecz Gabor
2025.03.19. (KP)
Current Data Parameters
NAME 144404
EXPNO 11
PROCNO 1
F2 - Acquisition Parameters
Date_ 20250319
Time 19.08 h
INSTRUM spect
PROBHD Z145856_0002 (
PULPROG zg30
TD 65536
SOLVENT CDCl3
NS 16
DS 2
SWH 12019.230 Hz
FIDRES 0.366798 Hz
AQ 2.7262976 sec
RG 111.4
DW 41.600 usec
DE 25.00 usec
TE 295.0 K
D1 1.00000000 sec
TD0 1
SFO1 600.0037050 MHz
NUC1 1H
P1 11.50 usec
PLW1 28.00000000 W
F2 - Processing parameters
SI 65536
SF 600.0000098 MHz
WDW EM
SSB 0
LB 0.30 Hz
GB 0
PC 1.00
8.0 7.5 7.0 6.5 6.0 5.5 5.0 4.5 4.0 3.5 3.0 2.5 2.0 1.5 1.0 0.5 ppm
1.00 1.05 1.09 1.01 1.08 1.07 4.44 2.22 3.26 2.15 2.17 1.10 2.72 2.29 4.29 3.30 0.21

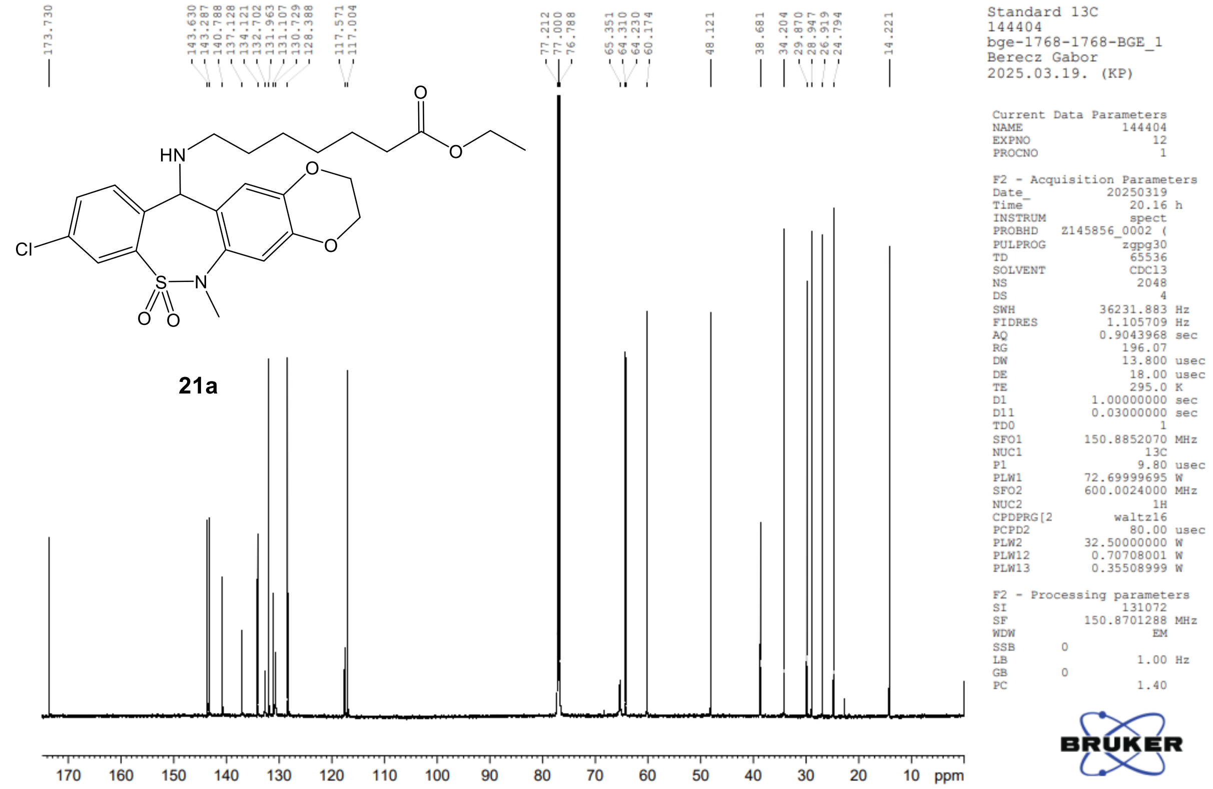
173.730
143.630 143.287 140.788 137.128 134.121 132.702 131.963 131.107 130.729 128.388
117.571 117.004
77.212 77.000 76.788
65.351 64.310 64.230 60.174
48.121
38.681
34.204 29.870 28.947 26.919 24.794
14.221
21a
Standard 13C
144404
bge-1768-1768-BGE_1
Berecz Gabor
2025.03.19. (KP)
Current Data Parameters
NAME 144404
EXPNO 12
PROCNO 1
F2 - Acquisition Parameters
Date_ 20250319
Time 20.16 h
INSTRUM spect
PROBHD Z145856_0002 (
PULPROG zgpg30
TD 65536
SOLVENT CDCl3
NS 2048
DS 4
SWH 36231.883 Hz
FIDRES 1.105709 Hz
AQ 0.9043968 sec
RG 196.07
DW 13.800 usec
DE 18.00 usec
TE 295.0 K
D1 1.00000000 sec
D11 0.03000000 sec
TD0 1
SFO1 150.8852070 MHz
NUC1 13C
P1 9.80 usec
PLW1 72.69999695 W
SFO2 600.0024000 MHz
NUC2 1H
CPDPRG[2 waltz16
PCPD2 80.00 usec
PLW2 32.50000000 W
PLW12 0.70708001 W
PLW13 0.35508999 W
F2 - Processing parameters
SI 131072
SF 150.8701288 MHz
WDW EM
SSB 0
LB 1.00 Hz
GB 0
PC 1.40
170 160 150 140 130 120 110 100 90 80 70 60 50 40 30 20 10 ppm

21a

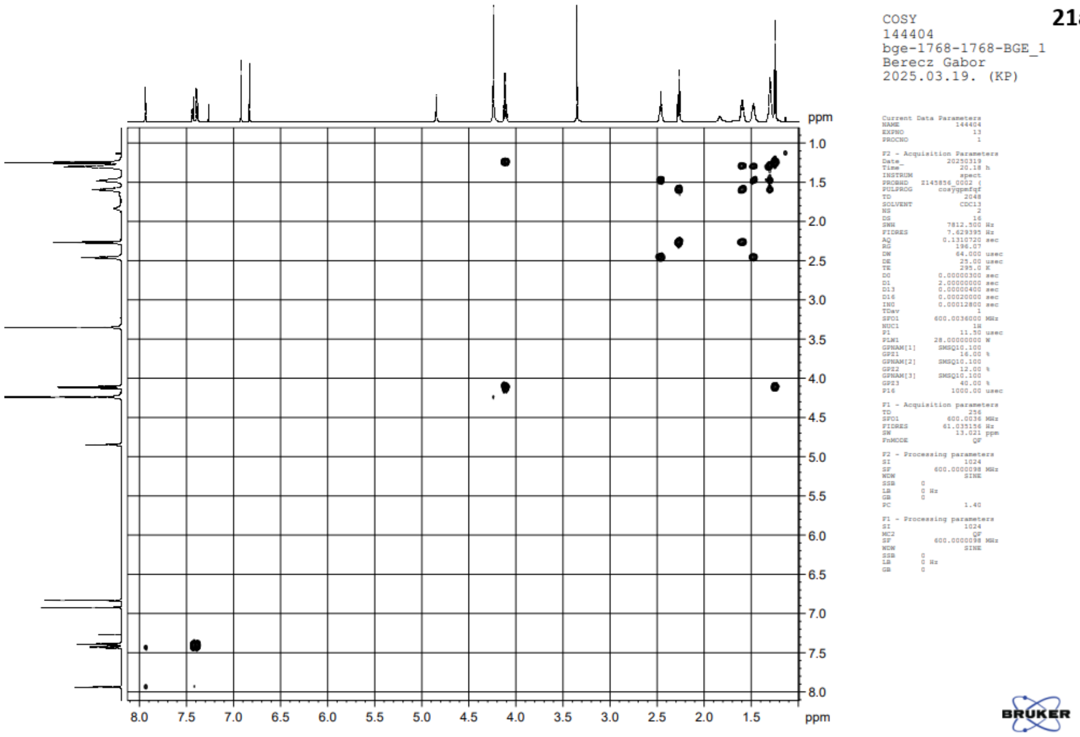

COSY
144404
bge-1768-1768-BGE_1
Berecz Gabor
2025.03.19. (KP)
Current Data Parameters
NAME 144404
EXPNO 13
PROCNO 1
F2 - Acquisition Parameters
Date_ 20250319
Time 20.18 h
INSTRUM spect
PROBHD Z145856_0002 (
PULPROG cosygpmfqf
TD 2048
SOLVENT CDCl3
NS 2
DS 16
SWH 7812.500 Hz
FIDRES 7.629395 Hz
AQ 0.1310720 sec
RG 196.07
DW 64.000 usec
DE 25.00 usec
TE 295.0 K
D0 0.00000300 sec
D1 2.00000000 sec
D13 0.00000400 sec
D16 0.00020000 sec
IN0 0.00012800 sec
TDav 1
SFO1 600.0036000 MHz
NUC1 1H
P1 11.50 usec
PLW1 28.00000000 W
GPNAM[1] SMSQ10.100
GPZ1 16.00 %
GPNAM[2] SMSQ10.100
GPZ2 12.00 %
GPNAM[3] SMSQ10.100
GPZ3 40.00 %
P16 1000.00 usec
F1 - Acquisition parameters
TD 256
SFO1 600.0036 MHz
FIDRES 61.035156 Hz
SW 13.021 ppm
FnMODE QF
F2 - Processing parameters
SI 1024
SF 600.0000098 MHz
WDW SINE
SSB 0
LB 0 Hz
GB 0
PC 1.40
F1 - Processing parameters
SI 1024
MC2 QF
SF 600.0000098 MHz
WDW SINE
SSB 0
LB 0 Hz
GB 0
ppm
8.0 7.5 7.0 6.5 6.0 5.5 5.0 4.5 4.0 3.5 3.0 2.5 2.0 1.5
1.0 1.5 2.0 2.5 3.0 3.5 4.0 4.5 5.0 5.5 6.0 6.5 7.0 7.5 8.0
BRUKER

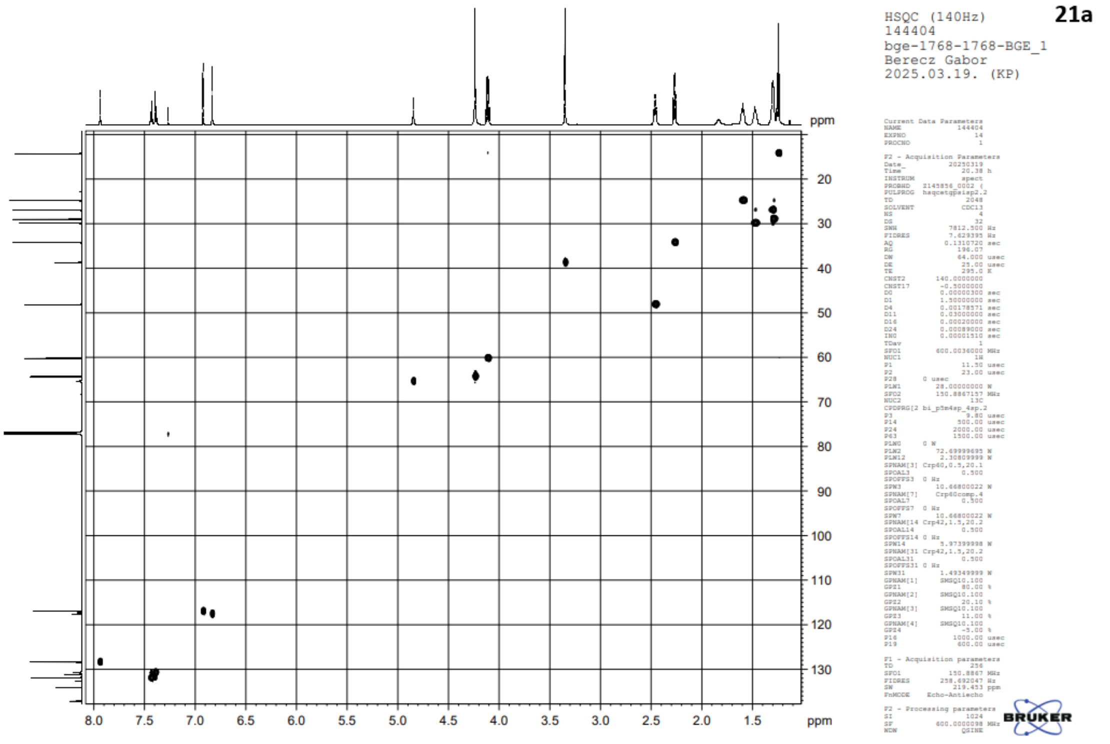
HSQC (140Hz)
144404
bge-1768-1768-BGE_1
Berecz Gabor
2025.03.19. (KP)
21a
BRUKER

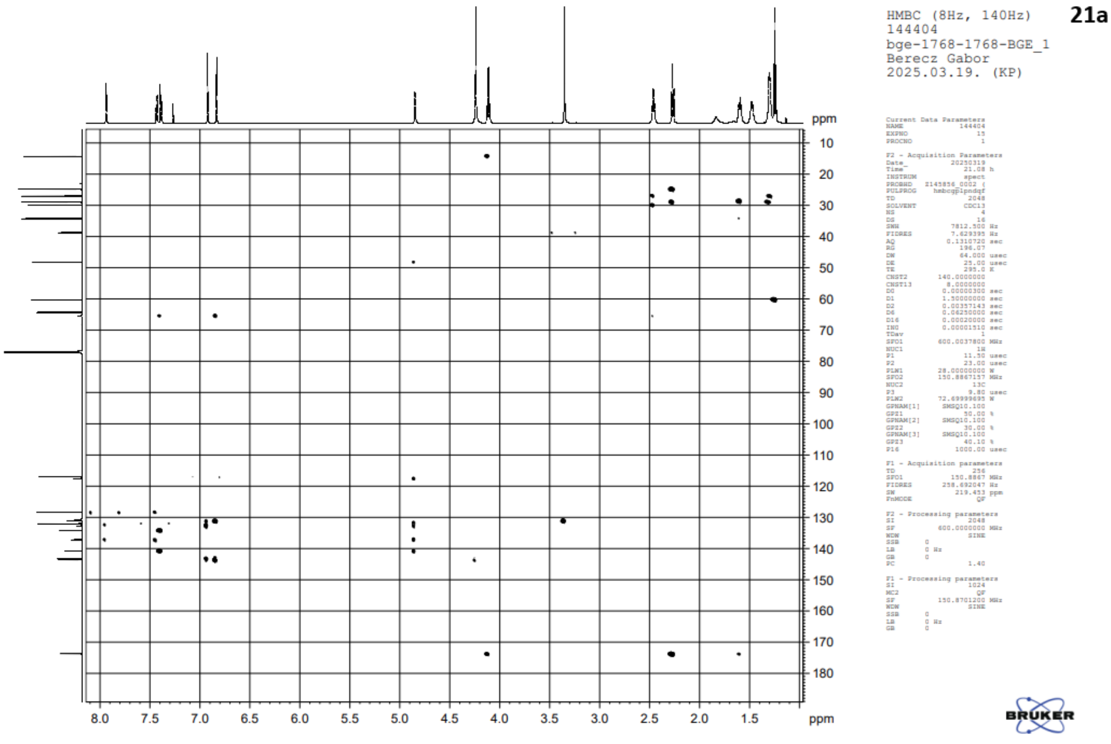
HMBC (8Hz, 140Hz)
144404
bge-1768-1768-BGE_1
Berecz Gabor
2025.03.19. (KP)
21a
BRUKER

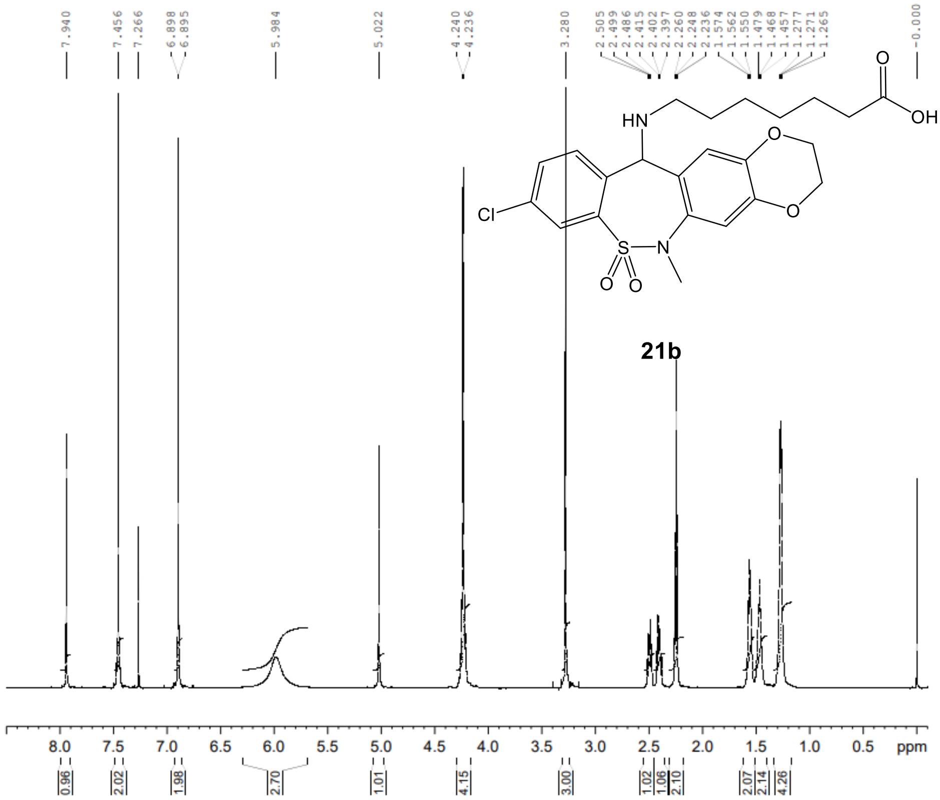

Standard 1H
144419
bge1778-1974-BGE_1
Berecz Gabor
2025.03.21. (KP)

Current Data Parameters
NAME 144419
EXPNO 11
PROCNO 1

F2 - Acquisition Parameters
Date_ 20250321
Time 15.45 h
INSTRUM spect
PROBHD Z145856_0002 (
PULPROG zg30
TD 65536
SOLVENT CDCl3
NS 16
DS 2
SWH 12019.230 Hz
FIDRES 0.366798 Hz
AQ 2.7262976 sec
RG 119.07
DW 41.600 usec
DE 25.00 usec
TE 295.0 K
D1 1.00000000 sec
TD0 1
SFO1 600.0037050 MHz
NUC1 1H
P1 11.50 usec
PLW1 28.00000000 W

F2 - Processing parameters
SI 65536
SF 600.0000100 MHz
WDW EM
SSB 0
LB 0.30 Hz
GB 0
PC 1.00

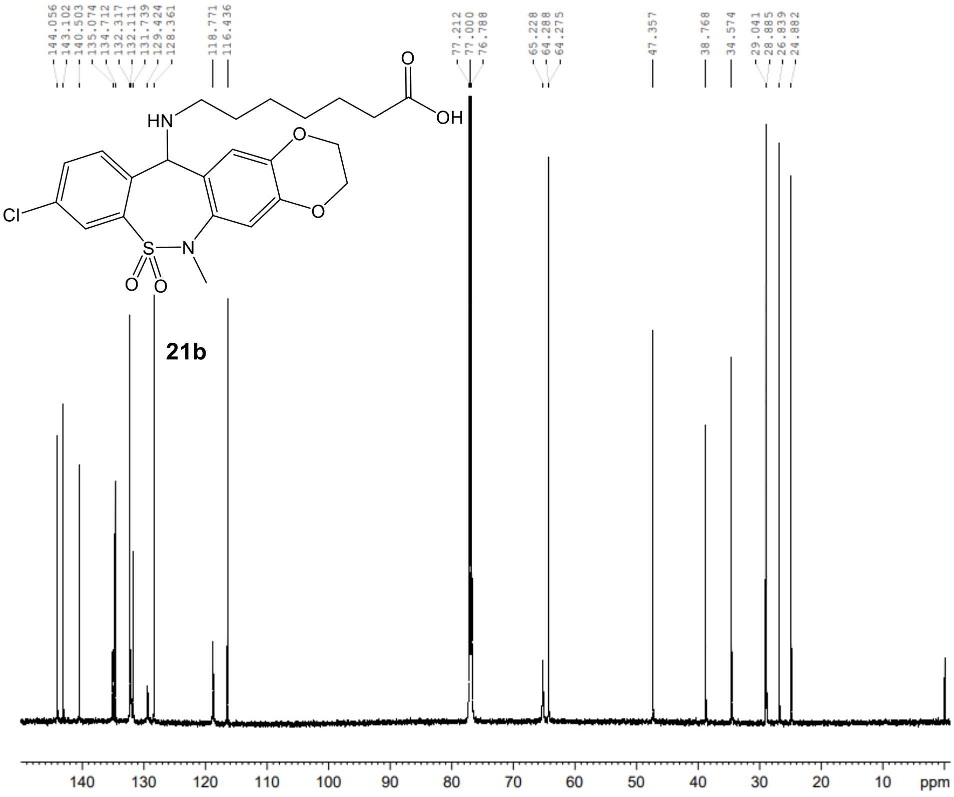

Standard 13C
144419
bge1778-1974-BGE_1
Berecz Gabor
2025.03.21. (KP)

Current Data Parameters
NAME 144419
EXPNO 12
PROCNO 1

F2 - Acquisition Parameters
Date_ 20250321
Time 16.53 h
INSTRUM spect
PROBHD Z145856_0002 (
PULPROG zgpg30
TD 65536
SOLVENT CDCl3
NS 2048
DS 4
SWH 36231.883 Hz
FIDRES 1.105709 Hz
AQ 0.9043968 sec
RG 196.07
DW 13.800 usec
DE 18.00 usec
TE 295.0 K
D1 1.00000000 sec
D11 0.03000000 sec
TD0 1
SFO1 150.8852070 MHz
NUC1 13C
P1 9.80 usec
PLW1 72.69999695 W
SFO2 600.0024000 MHz
NUC2 1H
CPDPRG[2 waltz16
PCPD2 80.00 usec
PLW2 32.50000000 W
PLW12 0.70708001 W
PLW13 0.35508999 W

F2 - Processing parameters
SI 131072
SF 150.8701283 MHz
WDW EM
SSB 0
LB 1.00 Hz
GB 0
PC 1.40

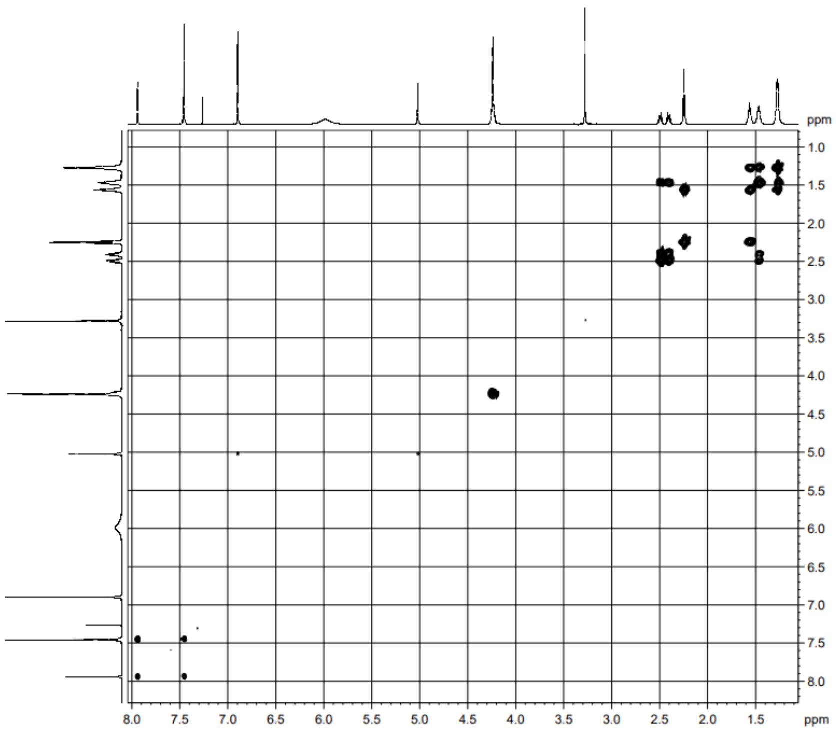

COSY
144419
bge1778-1974-BGE_1
Berecz Gabor
2025.03.21. (KP)

21b

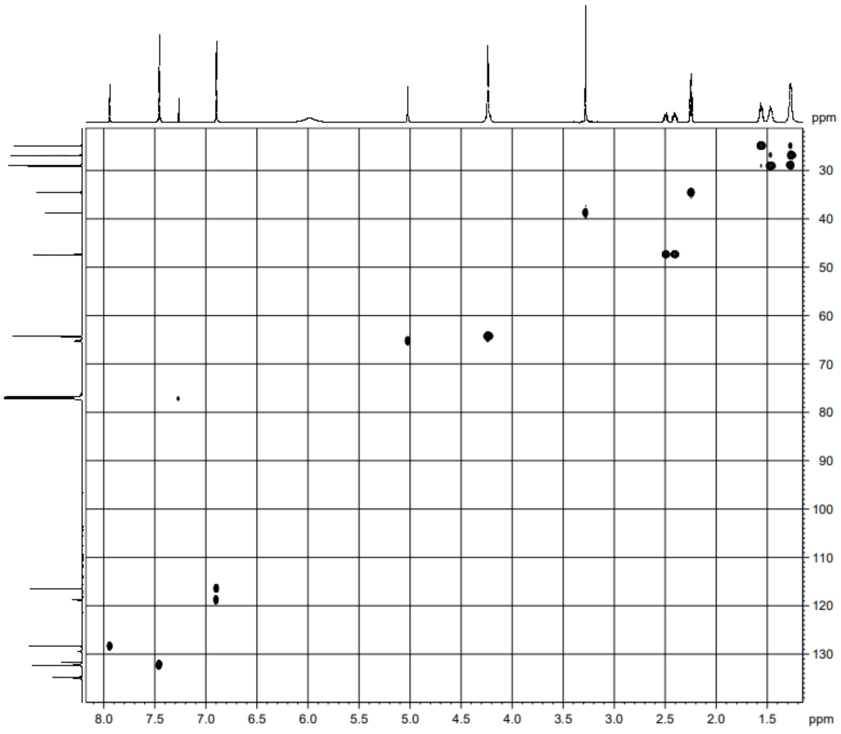

HSQC (140Hz)
144419
bge1778-1974-BGE_1
Berecz Gabor
2025.03.21. (KP)

21b

21b

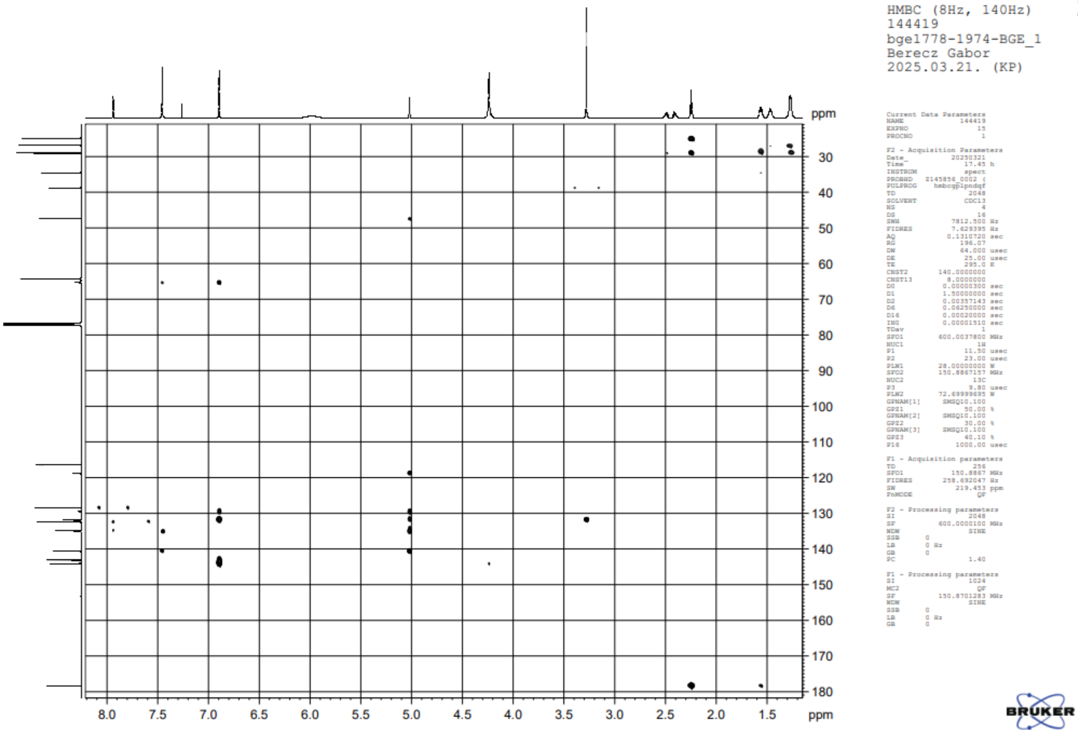

HMBC (8Hz, 140Hz)
144419
bge1778-1974-BGE_1
Berecz Gabor
2025.03.21. (KP)
ppm
30
40
50
60
70
80
90
100
110
120
130
140
150
160
170
180
8.0
7.5
7.0
6.5
6.0
5.5
5.0
4.5
4.0
3.5
3.0
2.5
2.0
1.5
ppm

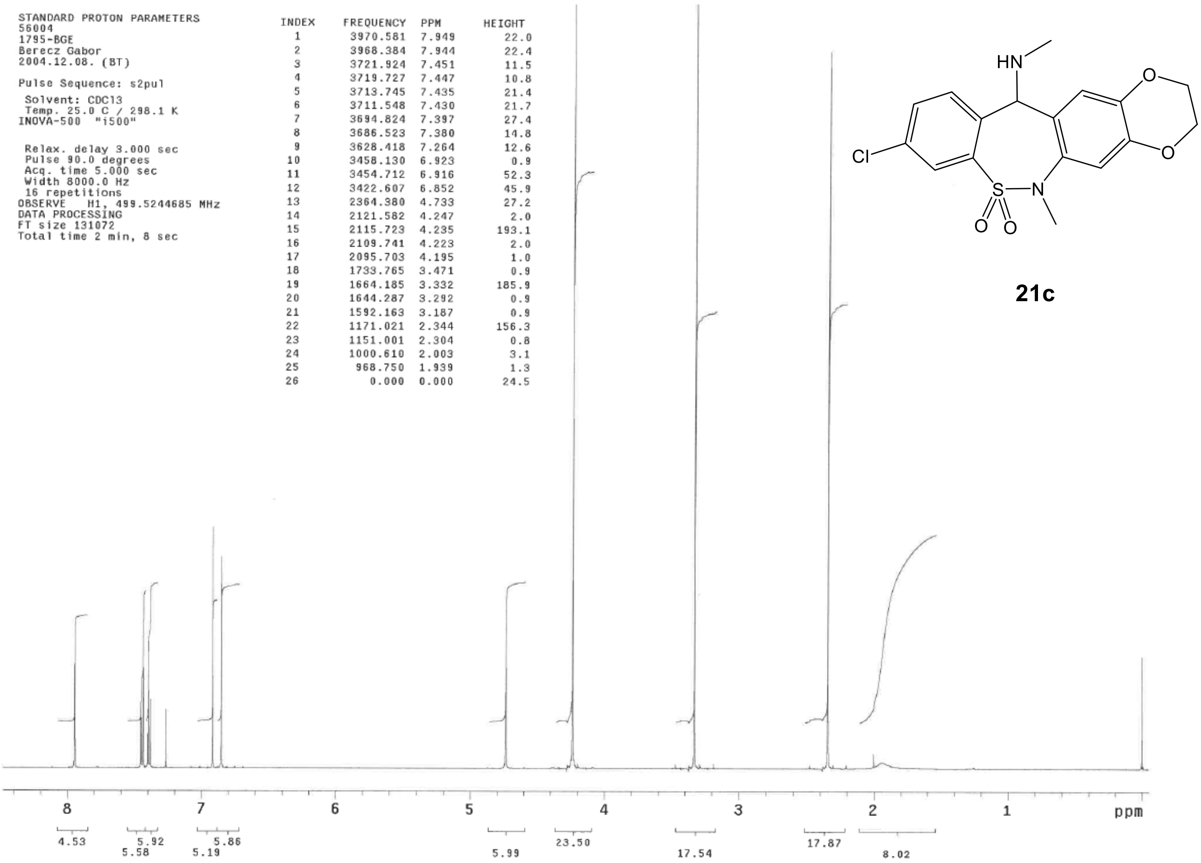
STANDARD PROTON PARAMETERS
56004
1795-BGE
Berecz Gabor
2004.12.08. (BT)
Pulse Sequence: s2pul
Solvent: CDCl3
Temp. 25.0 C / 298.1 K
INOVA-500 "i500"
Relax. delay 3.000 sec
Pulse 90.0 degrees
Acq. time 5.000 sec
Width 8000.0 Hz
16 repetitions
OBSERVE H1, 499.5244685 MHz
DATA PROCESSING
FT size 131072
Total time 2 min, 8 sec
INDEX FREQUENCY PPM HEIGHT
1 3970.581 7.949 22.0
2 3968.384 7.944 22.4
3 3721.924 7.451 11.5
4 3719.727 7.447 10.8
5 3713.745 7.435 21.4
6 3711.548 7.430 21.7
7 3694.824 7.397 27.4
8 3686.523 7.380 14.8
9 3628.418 7.264 12.6
10 3458.130 6.923 0.9
11 3454.712 6.916 52.3
12 3422.607 6.852 45.9
13 2364.380 4.733 27.2
14 2121.582 4.247 2.0
15 2115.723 4.235 193.1
16 2109.741 4.223 2.0
17 2095.703 4.195 1.0
18 1733.765 3.471 0.9
19 1664.185 3.332 185.9
20 1644.287 3.292 0.9
21 1592.163 3.187 0.9
22 1171.021 2.344 156.3
23 1151.001 2.304 0.8
24 1000.610 2.003 3.1
25 968.750 1.939 1.3
26 0.000 0.000 24.5
8
7
6
5
4
3
2
1
ppm
4.53
5.92
5.58
5.86
5.19
5.99
23.50
17.54
17.87
8.02
HN
O
O
Cl
S
N
O
O

21c

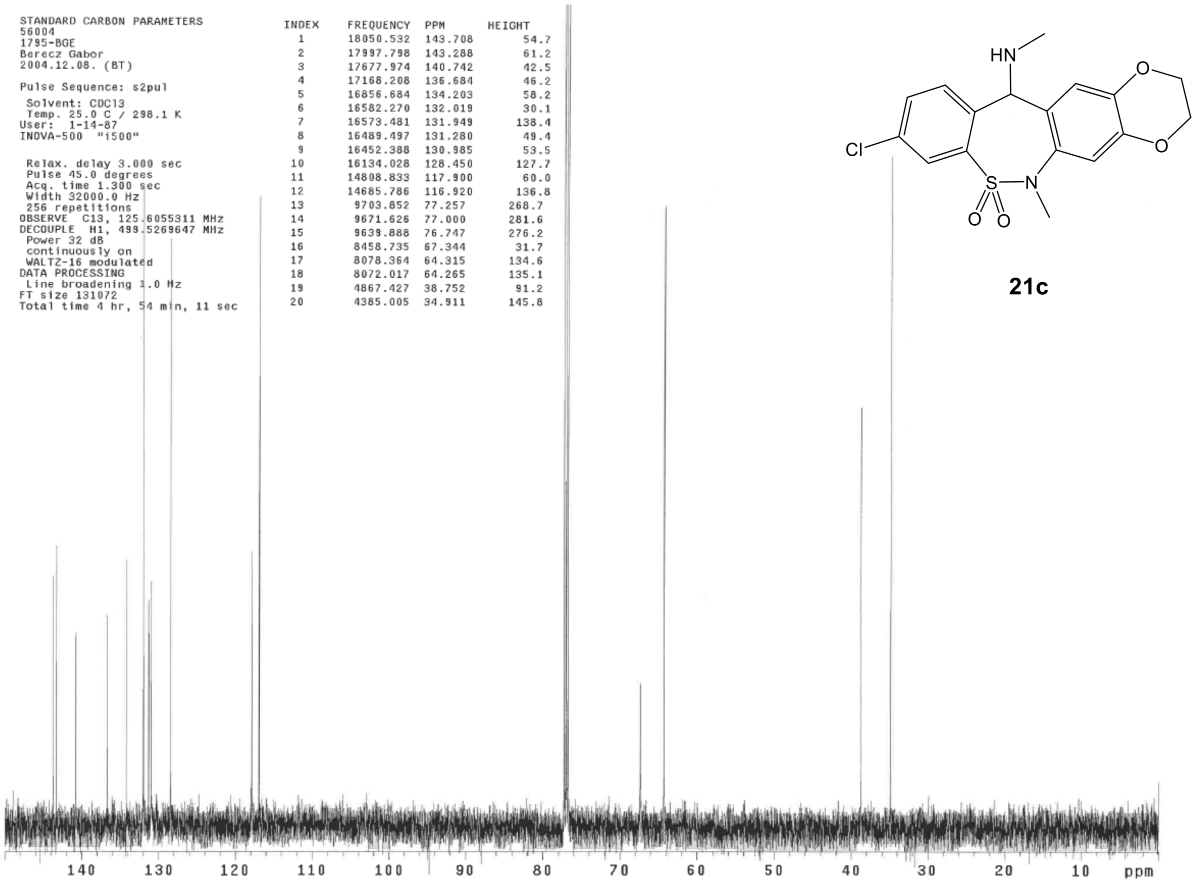
STANDARD CARBON PARAMETERS
56004
1795-BGE
Berecz Gabor
2004.12.08. (BT)
Pulse Sequence: s2pul
Solvent: CDCl3
Temp. 25.0 C / 298.1 K
User: 1-14-87
INOVA-500 "i500"
Relax. delay 3.000 sec
Pulse 45.0 degrees
Acq. time 1.300 sec
Width 32000.0 Hz
256 repetitions
OBSERVE C13, 125.6055311 MHz
DECOUPLE H1, 499.5269647 MHz
Power 32 dB
continuously on
WALTZ-16 modulated
DATA PROCESSING
Line broadening 1.0 Hz
FT size 131072
Total time 4 hr, 54 min, 11 sec
INDEX FREQUENCY PPM HEIGHT
1 18050.532 143.708 54.7
2 17997.798 143.288 61.2
3 17677.974 140.742 42.5
4 17168.208 136.684 46.2
5 16856.684 134.203 58.2
6 16582.270 132.019 30.1
7 16573.481 131.949 138.4
8 16489.497 131.280 49.4
9 16452.388 130.985 53.5
10 16134.028 128.450 127.7
11 14808.833 117.900 60.0
12 14685.786 116.920 136.8
13 9703.852 77.257 268.7
14 9671.626 77.000 281.6
15 9639.888 76.747 276.2
16 8458.735 67.344 31.7
17 8078.364 64.315 134.6
18 8072.017 64.265 135.1
19 4867.427 38.752 91.2
20 4385.005 34.911 145.8
140
130
120
110
100
90
80
70
60
50
40
30
20
10
ppm
HN
O
O
Cl
S
N
O
O

21c

STANDARD 1H
60132
1904-BGE
Berecz Gabor
2005/10/19 (SzA)

Solvent: CDCl3
Temp. 25.0 C / 298.1 K
INOVA-500 "i500"

PULSE SEQUENCE
Relax. delay 3.000 sec
Pulse 6.4 usec
Acq. time 5.000 sec
Width 8000.0 Hz
16 repetitions
OBSERVE H1, 499.5244649 MHz
DATA PROCESSING
FT size 131072
Total time 2 minutes

INDEX	FREQUENCY	PPM	HEIGHT
1	3964.966	7.937	21.1
2	3962.891	7.933	20.8
3	3714.844	7.437	8.0
4	3712.769	7.433	7.1
5	3706.543	7.420	23.8
6	3704.468	7.416	24.5
7	3698.486	7.404	26.0
8	3690.186	7.387	8.2
9	3631.958	7.271	10.2
10	3465.942	6.938	53.3
11	3419.556	6.846	42.3
12	2423.462	4.852	14.9
13	2110.474	4.225	147.1
14	1835.205	3.674	23.9
15	1830.566	3.665	32.1
16	1825.928	3.655	23.6
17	1694.824	3.393	159.7
18	1294.800	2.592	10.5
19	1292.969	2.588	10.9
20	1289.063	2.581	7.1
21	1286.987	2.576	7.3
22	1245.239	2.493	7.6
23	1239.014	2.480	15.7
24	1233.032	2.468	15.0
25	1226.807	2.456	7.7
26	1179.199	2.361	16.7
27	1174.927	2.352	22.2
28	1170.654	2.344	15.9
29	997.681	1.997	11.4
30	0.000	0.000	71.2

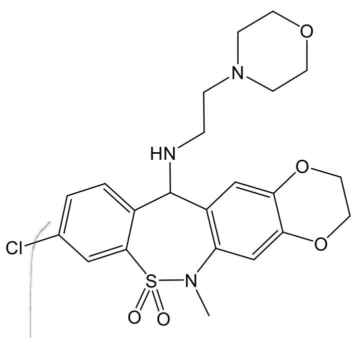

21d

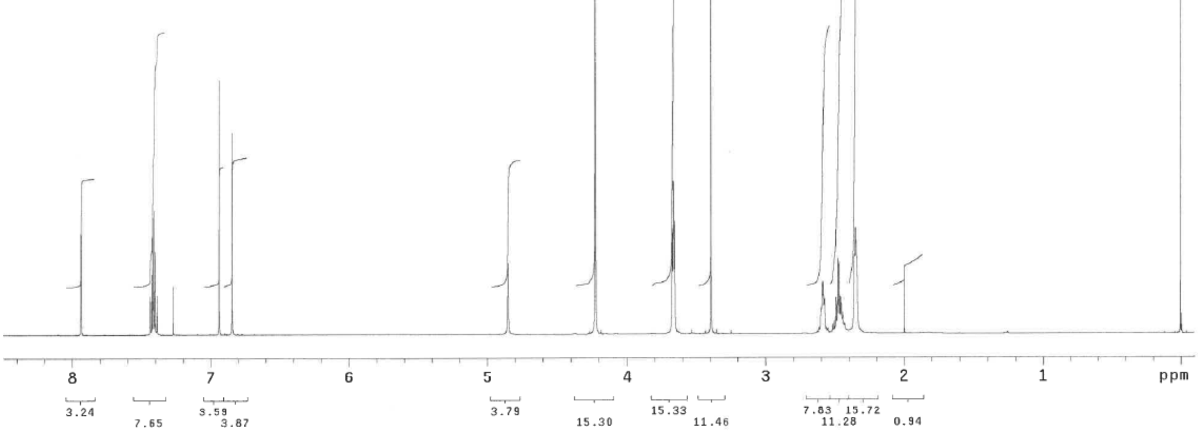

STANDARD 13C
60132
1904-BGE
Berecz Gabor
2005/10/19 (SzA)

Solvent: CDCl3
Temp. 25.0 C / 298.1 K
User: 1-14-87
INOVA-500 "i500"

PULSE SEQUENCE
Relax. delay 3.000 sec
Pulse 6.0 usec
Acq. time 1.300 sec
Width 32000.0 Hz
672 repetitions
OBSERVE C13, 125.6055347 MHz
DECOUPLE H1, 499.5269647 MHz
Power 32 dB
continuously on
WALTZ-16 modulated
DATA PROCESSING
Line broadening 0.5 Hz
FT size 131072
Total time 48 minutes

INDEX	FREQUENCY	PPM	HEIGHT
1	18043.953	143.645	55.8
2	18004.890	143.334	68.0
3	17705.086	140.947	37.8
4	17232.430	137.184	28.6
5	16847.664	134.121	54.9
6	16714.851	133.064	18.9
7	16558.113	131.816	97.9
8	16451.668	130.969	40.2
9	16397.469	130.537	18.8
10	16116.219	128.298	98.5
11	14721.687	117.197	23.0
12	14691.414	116.956	90.7
13	(11896.492	94.706	6.2)
14	9704.109	77.253	136.0
15	9672.371	77.000	134.9
16	9640.144	76.743	136.6
17	8397.469	66.851	180.8
18	8159.676	64.958	10.1
19	8075.203	64.285	84.7
20	8064.461	64.200	84.6
21	7290.047	58.035	90.5
22	6736.824	53.631	150.5
23	5548.836	44.173	82.8
24	4824.715	38.409	49.9
25	(-7.317	-0.058	20.8)

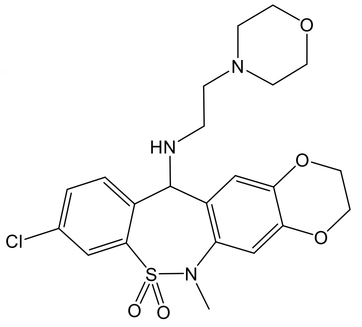

21d

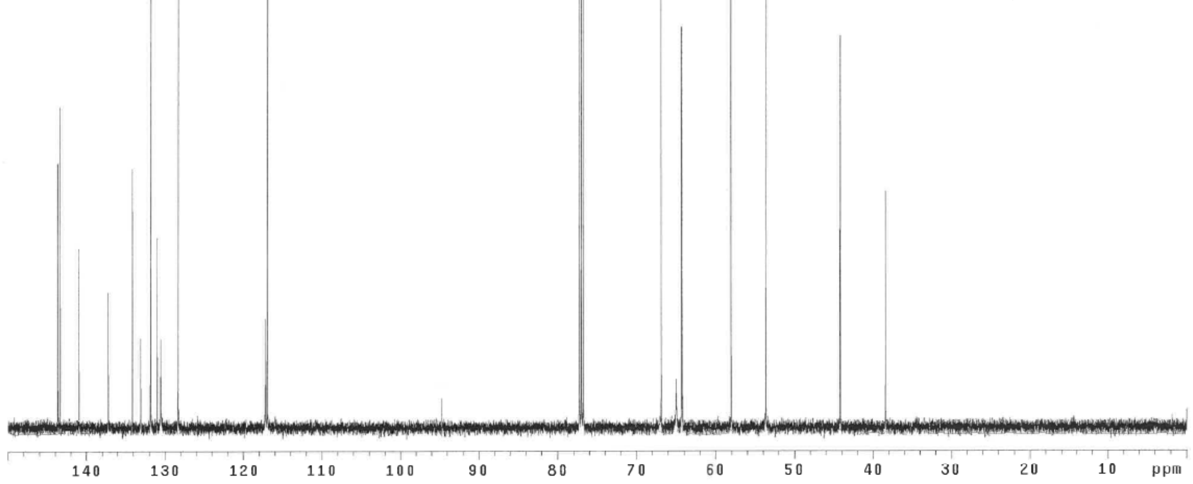

STANDARD 1H
60177
1905-BGE
Berecz Gabor
2005/10/21 (SzA)
Solvent: CDCl3
Temp. 25.0 C / 298.1 K
INOVA-500 "i500"
PULSE SEQUENCE
Relax. delay 3.000 sec
Pulse 6.4 usec
Acq. time 5.000 sec
Width 8000.0 Hz
16 repetitions
OBSERVE H1, 499.5244651 MHz
DATA PROCESSING
FT size 131072
Total time 2 minutes

INDEX	FREQUENCY	PPM	HEIGHT
1	3963.501	7.935	20.5
2	3962.769	7.933	25.6
3	3710.693	7.428	66.4
4	3632.080	7.271	10.7
5	3459.229	6.925	55.0
6	3423.950	6.854	47.2
7	2459.106	4.923	22.6
8	2111.938	4.228	162.0
9	1834.229	3.672	28.4
10	1829.590	3.663	44.5
11	1825.073	3.654	27.7
12	1676.147	3.355	159.0
13	1291.748	2.586	7.3
14	1288.818	2.580	8.7
15	1285.278	2.573	14.9
16	1282.349	2.567	15.3
17	1278.687	2.560	9.0
18	1276.001	2.554	8.1
19	1208.252	2.419	24.1
20	1203.735	2.410	33.6
21	1196.655	2.396	42.7
22	1189.697	2.382	21.5
23	998.657	1.999	5.7
24	852.051	1.706	8.6
25	848.389	1.698	8.9
26	845.337	1.692	12.2
27	841.675	1.685	12.2
28	838.623	1.679	8.5
29	834.839	1.671	8.3
30	-0.000	-0.000	14.4

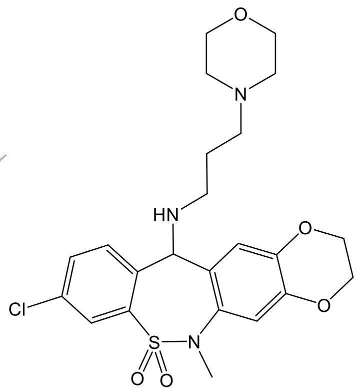

21e

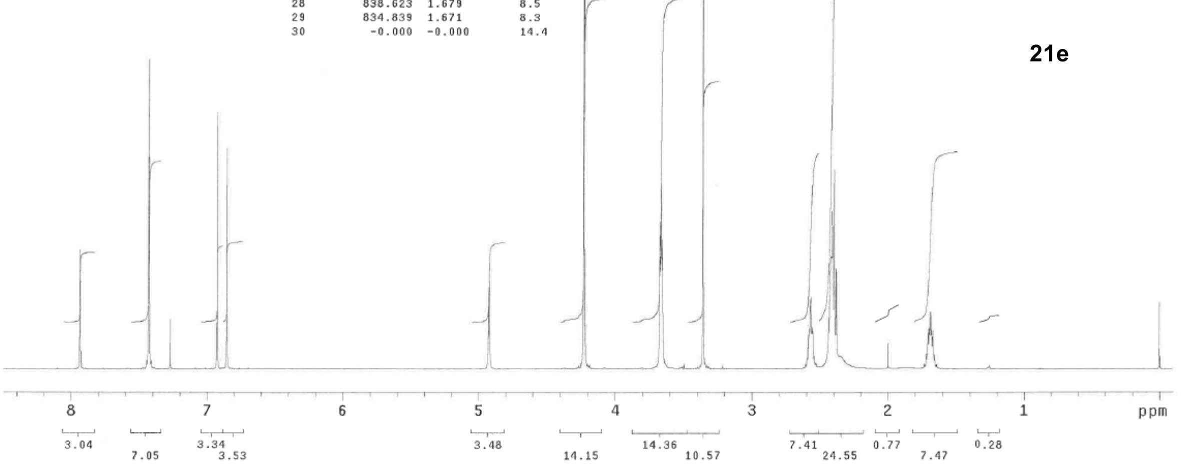

STANDARD 13C
60177
1905-BGE
Berecz Gabor
2005/10/21 (SzA)
Solvent: CDCl3
Temp. 25.0 C / 298.1 K
User: 1-14-87
INOVA-500 "i500"
PULSE SEQUENCE
Relax. delay 3.000 sec
Pulse 6.0 usec
Acq. time 1.300 sec
Width 32000.0 Hz
528 repetitions
OBSERVE C13, 125.6055337 MHz
DECOUPLE H1, 499.5269647 MHz
Power 32 dB
continuously on
WALTZ-16 modulated
DATA PROCESSING
Line broadening 0.5 Hz
FT size 131072
Total time 37 minutes

INDEX	FREQUENCY	PPM	HEIGHT
1	18031.746	143.547	42.5
2	18006.844	143.349	48.4
3	17671.883	140.683	30.6
4	17265.633	137.449	20.6
5	16832.527	134.001	39.7
6	16722.664	133.126	11.5
7	16564.949	131.871	67.2
8	16451.179	130.965	26.7
9	16334.480	130.036	13.7
10	16124.519	128.364	71.9
11	14687.996	116.928	74.1
12	9704.109	77.253	114.3
13	9672.371	77.000	121.1
14	9640.144	76.743	114.6
15	8401.863	66.886	146.8
16	8077.156	64.301	69.0
17	8065.437	64.207	63.9
18	7198.738	57.308	68.1
19	6750.496	53.739	140.6
20	5891.609	46.902	56.9
21	4822.761	38.393	34.1
22	3312.996	26.374	66.6

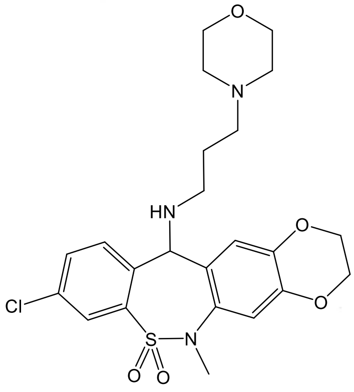

21e

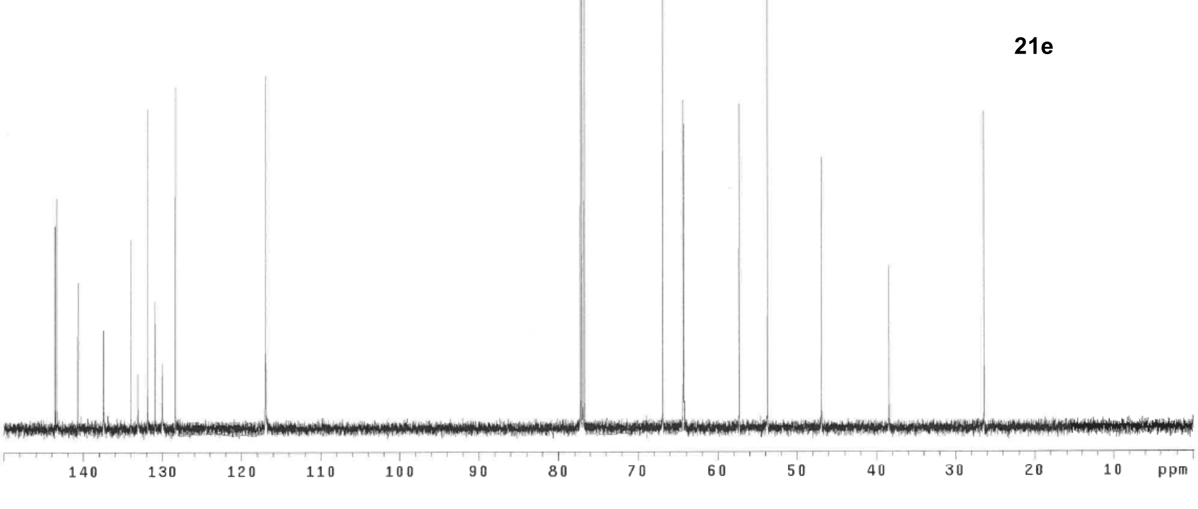

STANDARD 1H
63626
1962-BGE
Berecz Gabor
2006.05.26. (BT)
Solvent: CDCl3
Temp. 25.0 C / 298.1 K
INOVA-500 "i500"
PULSE SEQUENCE
Relax. delay 3.000 sec
Pulse 6.4 usec
Acq. time 5.000 sec
Width 8000.0 Hz
16 repetitions
OBSERVE H1, 499.5244573 MHz
DATA PROCESSING
FT size 131072
Total time 2 minutes

INDEX	FREQUENCY	PPM	HEIGHT
1	3961.426	7.930	27.2
2	3959.473	7.926	29.5
3	3713.257	7.434	8.9
4	3711.182	7.429	9.0
5	3704.956	7.417	24.5
6	3702.881	7.413	26.8
7	3695.801	7.399	33.1
8	3687.500	7.382	11.8
9	3639.404	7.286	4.7
10	3453.369	6.913	54.5
11	3415.527	6.838	49.6
12	2429.077	4.863	26.0
13	2109.009	4.222	150.5
14	1842.896	3.689	40.1
15	1838.379	3.680	61.8
16	1833.862	3.671	43.1
17	1669.189	3.342	151.2
18	1247.681	2.498	18.2
19	1243.896	2.490	17.9
20	1194.702	2.392	39.0
21	1152.100	2.306	12.7
22	1145.142	2.292	26.5
23	1138.550	2.279	13.4
24	966.919	1.936	3.6
25	752.686	1.507	35.2
26	749.268	1.500	48.2
27	0.000	0.000	5.2

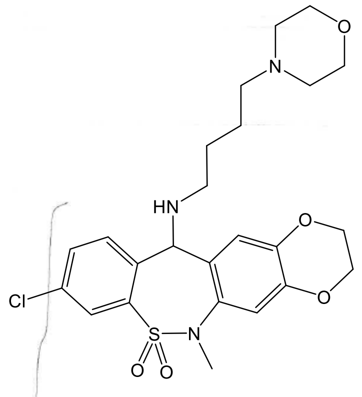

21f

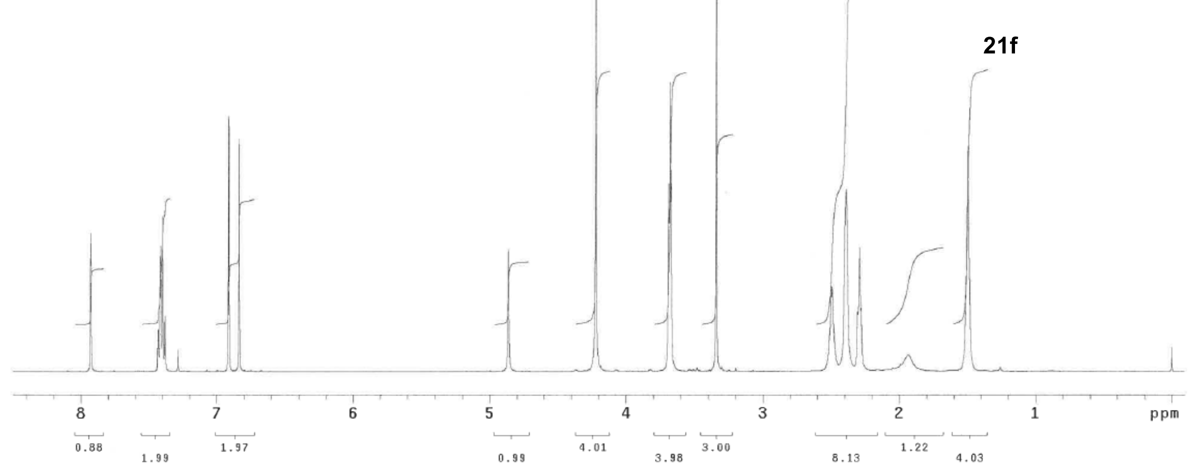

STANDARD 13C
63626
1962-BGE
Berecz Gabor
2006.05.26. (BT)
Solvent: CDCl3
Temp. 25.0 C / 298.1 K
User: 1-14-87
INOVA-500 "i500"
PULSE SEQUENCE
Relax. delay 3.000 sec
Pulse 6.0 usec
Acq. time 1.300 sec
Width 32000.0 Hz
144 repetitions
OBSERVE C13, 125.6055406 MHz
DECOUPLE H1, 499.5269647 MHz
Power 32 dB
continuously on
WALTZ-16 modulated
DATA PROCESSING
Line broadening 0.5 Hz
FT size 131072
Total time 10 minutes

INDEX	FREQUENCY	PPM	HEIGHT
1	18033.700	143.563	41.0
2	17989.266	143.209	42.7
3	17675.789	140.714	24.2
4	17216.805	137.060	20.0
5	16830.575	133.985	42.4
6	16646.004	132.516	10.8
7	16561.532	131.843	64.4
8	16460.946	131.043	25.0
9	16412.117	130.654	12.8
10	16112.801	128.271	58.1
11	14759.285	117.496	16.6
12	14682.137	116.882	61.5
13	9704.110	77.253	68.5
14	9672.371	77.000	71.8
15	9640.145	76.743	68.8
16	8395.028	66.831	148.7
17	8182.137	65.137	6.9
18	8067.391	64.223	54.1
19	8058.114	64.149	53.3
20	7367.684	58.653	86.7
21	6733.895	53.607	160.7
22	6016.121	47.893	59.9
23	4851.082	38.619	37.3
24	3497.078	27.840	72.1
25	3037.606	24.182	82.6

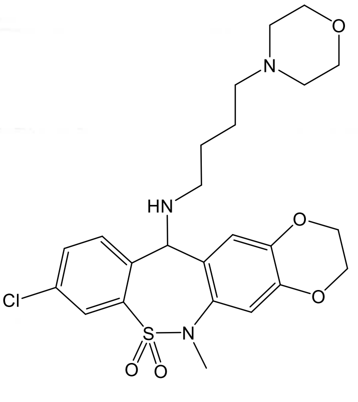

21f

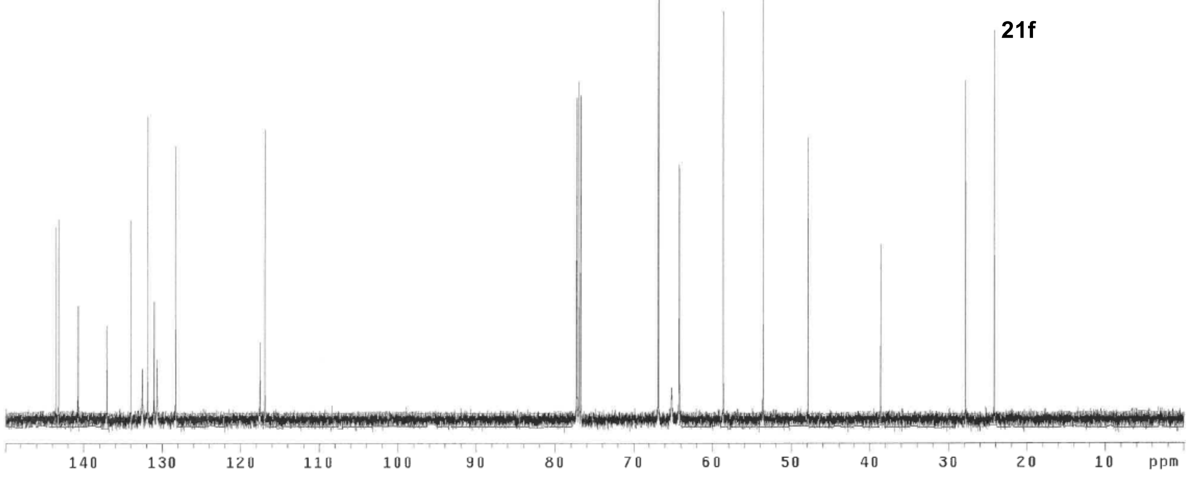

STANDARD PROTON PARAMETERS
54081
1735-BGE
Berecz Gabor
2004.06.10. (DA)

Pulse Sequence: s2pul

Solvent: CDCl3
Temp. 25.0 C / 298.1 K
INOVA-500 "i500"

Pulse 90.0 degrees
Acq. time 5.000 sec
Width 8000.0 Hz
16 repetitions
OBSERVE H1, 499.5244692 MHz
DATA PROCESSING
FT size 131072
Total time 1 min, 20 sec

INDEX	FREQUENCY	PPM	HEIGHT
1	3963.013	7.934	44.5
2	3960.937	7.929	43.5
3	3678.345	7.364	27.0
4	3676.147	7.359	25.4
5	3670.044	7.347	46.3
6	3667.847	7.343	44.9
7	3646.240	7.299	71.6
8	3637.939	7.283	43.6
9	3627.808	7.263	23.8
10	3480.469	6.968	104.9
11	3393.799	6.794	127.5
12	2121.094	4.246	15.3
13	2115.356	4.235	95.6
14	2113.037	4.230	125.8
15	2111.694	4.227	114.6
16	2109.741	4.223	88.7
17	2103.516	4.211	17.0
18	2097.534	4.199	14.0
19	2011.719	4.027	57.6
20	1730.835	3.465	438.7
21	1129.028	2.260	24.8
22	1126.709	2.256	24.5
23	1120.117	2.242	33.4
24	1086.792	2.176	33.1
25	1080.444	2.163	24.3
26	1077.881	2.158	24.1
27	847.412	1.696	43.6
28	843.750	1.689	64.5
29	840.942	1.683	125.3
30	838.135	1.678	61.9
31	834.595	1.671	36.5
32	-0.000	-0.000	37.3

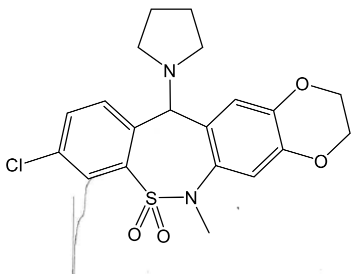

21g

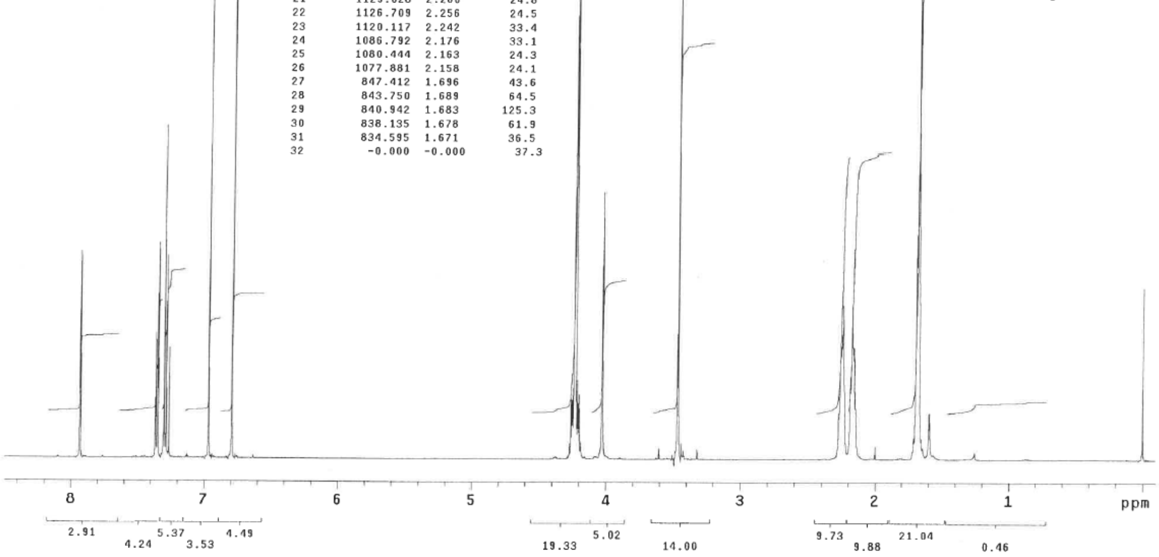

STANDARD 13C PARAMETERS
54081
1735-BGE
Berecz Gabor
2004.06.10. (DA)

Pulse Sequence: s2pul

Solvent: CDCl3
Temp. 25.0 C / 298.1 K
User: 1-14-87
INOVA-500 "i500"

Relax. delay 1.200 sec
Pulse 45.0 degrees
Acq. time 1.300 sec
Width 32000.0 Hz
1600 repetitions
OBSERVE C13, 125.6055311 MHz
DECOUPLE H1, 499.5269647 MHz
Power 34 dB
continuously on
WALTZ-16 modulated
DATA PROCESSING
Line broadening 0.5 Hz
FT size 131072
Total time 2 hr, 51 min, 18 sec

INDEX	FREQUENCY	PPM	HEIGHT
1	18067.622	143.844	38.6
2	18016.352	143.436	42.3
3	17972.895	143.090	9.7
4	17055.415	135.786	12.0
5	16931.392	134.798	33.9
6	16911.372	134.639	33.1
7	16670.649	132.722	25.4
8	16456.782	131.020	94.7
9	16409.907	130.646	33.1
10	16118.403	128.326	100.8
11	14993.892	119.373	55.1
12	14854.243	118.261	50.4
13	9703.852	77.257	110.9
14	9671.626	77.000	114.1
15	9639.888	76.747	114.3
16	9500.727	75.639	13.3
17	8078.364	64.315	98.9
18	8059.809	64.168	98.2
19	6741.938	53.675	200.0
20	5014.888	39.926	28.9
21	2912.349	23.186	190.3
22	-4.644	-0.037	6.6

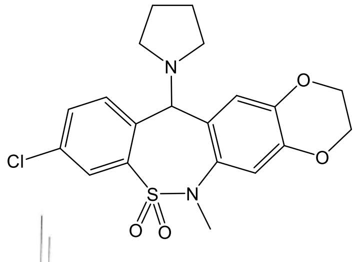

21g

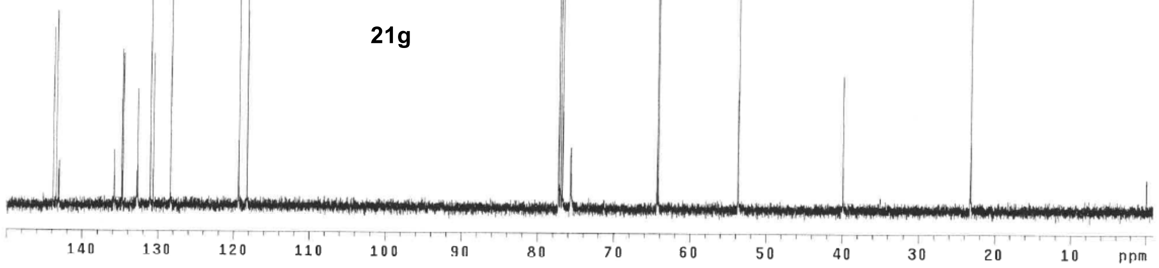

STANDARD PROTON PARAMETERS
56102
1798-BGE
Berecz Gabor
2004.12.15. (BT)
Solvent: DMSO
Temp. 25.0 C / 298.1 K
INOVA-500 "i500"
PULSE SEQUENCE
Relax. delay 3.000 sec
Pulse 90.0 degrees
Acq. time 5.000 sec
Width 8000.0 Hz
16 repetitions
OBSERVE H1, 499.5268343 MHz
DATA PROCESSING
FT size 131072
Total time 2 minutes

INDEX	FREQUENCY	PPM	HEIGHT
1	3893.555	7.794	29.9
2	3885.010	7.777	41.2
3	3860.840	7.729	44.0
4	3858.521	7.724	54.4
5	3845.337	7.698	36.9
6	3843.018	7.693	28.8
7	3836.792	7.681	26.5
8	3834.595	7.676	22.3
9	3523.560	7.054	81.1
10	3506.348	7.019	111.3
11	2732.300	5.470	52.2
12	2113.525	4.231	206.4
13	1658.691	3.321	306.7
14	1282.471	2.567	43.6
15	1251.465	2.505	10.4
16	1249.634	2.502	13.9
17	1247.803	2.498	9.9
18	-0.000	-0.000	9.9

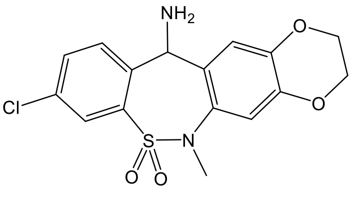

21h

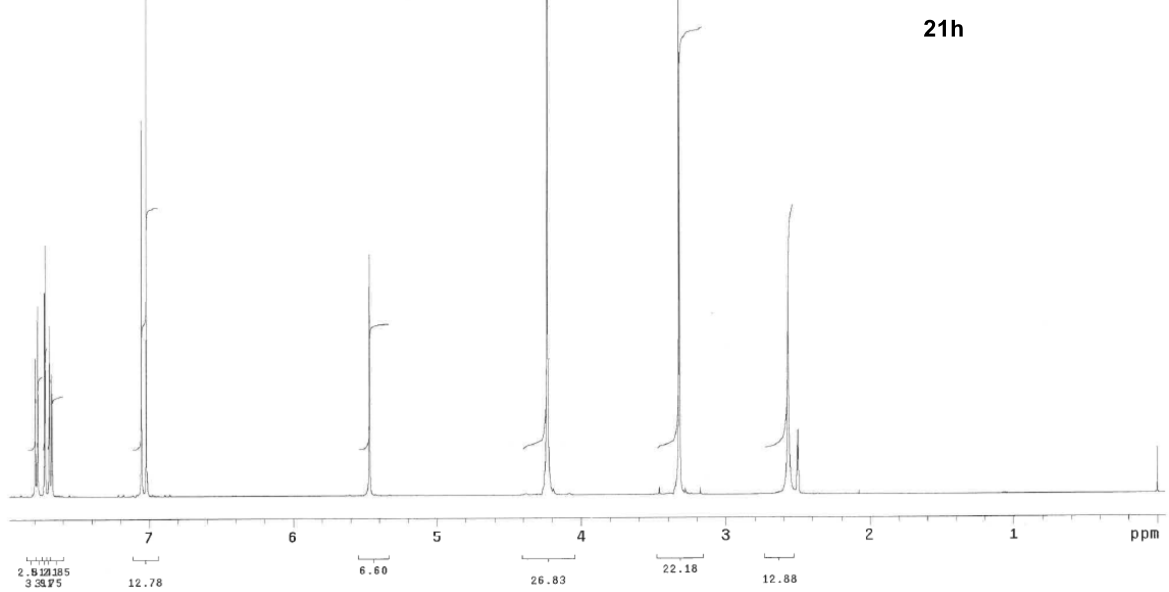

STANDARD CARBON PARAMETERS
56102
1798-BGE
Berecz Gabor
2004.12.15. (BT)
Solvent: DMSO
Temp. 25.0 C / 298.1 K
User: 1-14-87
INOVA-500 "i500"
PULSE SEQUENCE
Relax. delay 3.000 sec
Pulse 45.0 degrees
Acq. time 1.300 sec
Width 32000.0 Hz
256 repetitions
OBSERVE C13, 125.6061602 MHz
DECOUPLE H1, 499.5293374 MHz
Power 32 dB
continuously on
WALTZ-16 modulated
DATA PROCESSING
Line broadening 1.0 Hz
FT size 131072
Total time 18 minutes

INDEX	FREQUENCY	PPM	HEIGHT
1	18014.106	143.412	72.2
2	17950.141	142.902	61.6
3	17692.329	140.850	64.2
4	17662.056	140.609	61.4
5	17236.763	137.223	66.7
6	16614.692	132.271	61.2
7	16594.673	132.111	83.5
8	16274.848	129.565	65.3
9	16247.016	129.344	79.3
10	15918.891	126.732	86.5
11	14640.571	116.555	89.6
12	14449.653	115.035	84.0
13	8079.536	64.322	70.9
14	8066.841	64.221	72.3
15	6679.145	53.173	85.8
16	5049.751	40.201	44.2
17	5028.755	40.034	140.4
18	5007.759	39.867	281.8
19	4986.763	39.700	333.7
20	4965.766	39.533	282.3
21	4944.770	39.366	142.9
22	4923.774	39.199	45.7
23	4721.626	37.589	106.1

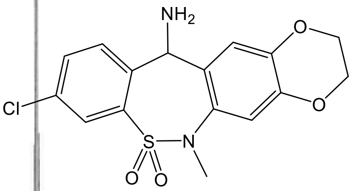

21h

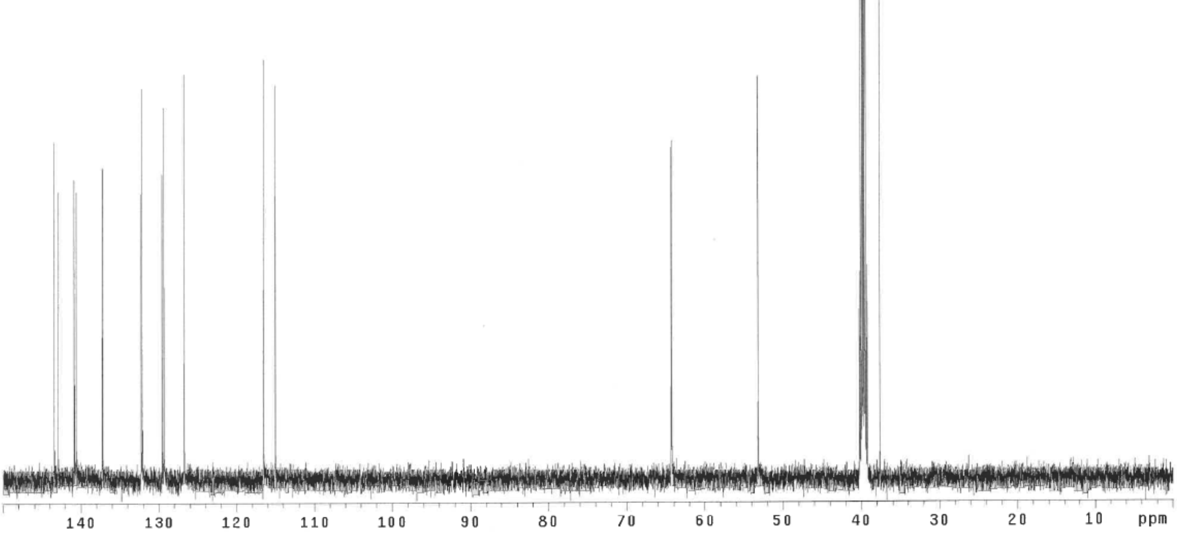

STANDARD 1H
79981
2264-BGE
Berecz Gabor
2009.05.15. (BB)
Solvent: CDCl3
Temp. 30.0 C / 303.1 K
INOVA-500 "i500"
PULSE SEQUENCE
Relax. delay 1.000 sec
Pulse 90.0 degrees
Acq. time 5.000 sec
Width 11999.4 Hz
16 repetitions
OBSERVE H1, 499.5244598 MHz
DATA PROCESSING
FT size 131072
Total time 1 minutes

INDEX	FREQUENCY	PPM	HEIGHT
1	3958.908	7.925	19.0
2	3956.894	7.921	18.5
3	3714.475	7.436	8.2
4	3712.461	7.432	7.2
5	3706.236	7.420	23.9
6	3705.137	7.417	10.0
7	3704.222	7.415	23.2
8	3697.630	7.402	28.5
9	3689.208	7.385	9.7
10	3636.659	7.280	7.1
11	3453.013	6.913	49.2
12	3412.732	6.832	43.9
13	2422.914	4.850	23.5
14	2111.100	4.226	130.2
15	2104.692	4.213	3.7
16	1673.134	3.349	155.3
17	1490.587	2.984	137.9
18	1461.658	2.926	110.4
19	1240.661	2.484	9.3
20	1239.379	2.481	8.8
21	1232.971	2.468	19.4
22	1226.379	2.455	10.3
23	1145.817	2.294	16.7
24	1138.493	2.279	25.7
25	1130.803	2.264	18.4
26	995.861	1.994	9.0
27	931.960	1.866	9.9
28	808.920	1.619	8.3
29	801.596	1.605	11.3
30	794.272	1.590	7.7
31	748.498	1.498	6.6
32	741.906	1.485	10.3
33	734.949	1.471	7.9
34	660.428	1.322	23.4
35	656.766	1.315	27.8
36	653.105	1.307	19.9
37	-0.000	-0.000	10.2

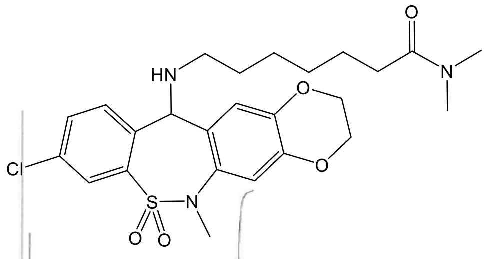

21i

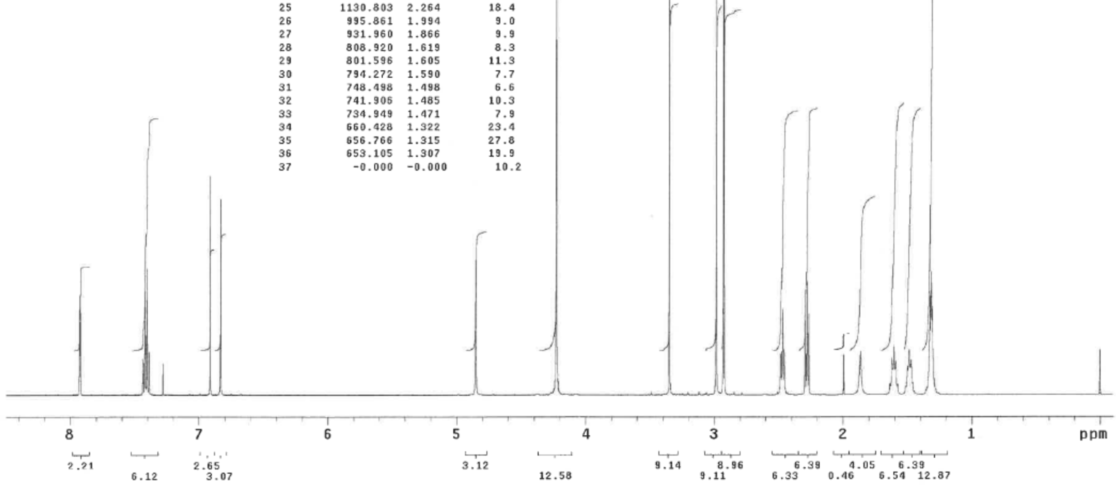

STANDARD 13C
79981
2264-BGE
Berecz Gabor
2009.05.15. (BB)
Solvent: CDCl3
Temp. 30.0 C / 303.1 K
User: 1-14-87
INOVA-500 "i500"
PULSE SEQUENCE
Relax. delay 3.000 sec
Pulse 90.0 degrees
Acq. time 1.300 sec
Width 32000.0 Hz
352 repetitions
OBSERVE C13, 125.6055352 MHz
DECOUPLE H1, 499.5269647 MHz
Power 34 dB
continuously on
WALTZ-16 modulated
DATA PROCESSING
Line broadening 0.5 Hz
FT size 131072
Total time 25 minutes

INDEX	FREQUENCY	PPM	HEIGHT
1	21721.687	172.922	33.7
2	18037.605	143.594	50.1
3	17997.078	143.271	49.6
4	17693.367	140.854	33.2
5	17234.383	137.200	31.8
6	16833.504	134.008	57.7
7	16677.254	132.765	18.2
8	16564.461	131.867	116.8
9	16465.340	131.078	34.3
10	16418.953	130.708	28.2
11	16112.801	128.271	113.4
12	14760.750	117.508	31.1
13	14693.855	116.975	108.1
14	9704.109	77.253	62.1
15	9672.371	77.000	66.3
16	9640.144	76.743	64.3
17	8196.297	65.249	15.4
18	8072.273	64.262	96.4
19	8063.484	64.192	101.4
20	6036.629	48.057	111.6
21	4852.058	38.626	67.5
22	4667.976	37.161	64.4
23	4427.254	35.245	67.7
24	4162.117	33.134	156.0
25	3749.031	29.845	124.9
26	3671.394	29.227	164.2
27	3396.492	27.039	154.0
28	3132.332	24.936	152.3

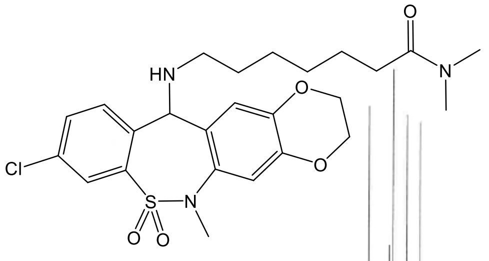

21i

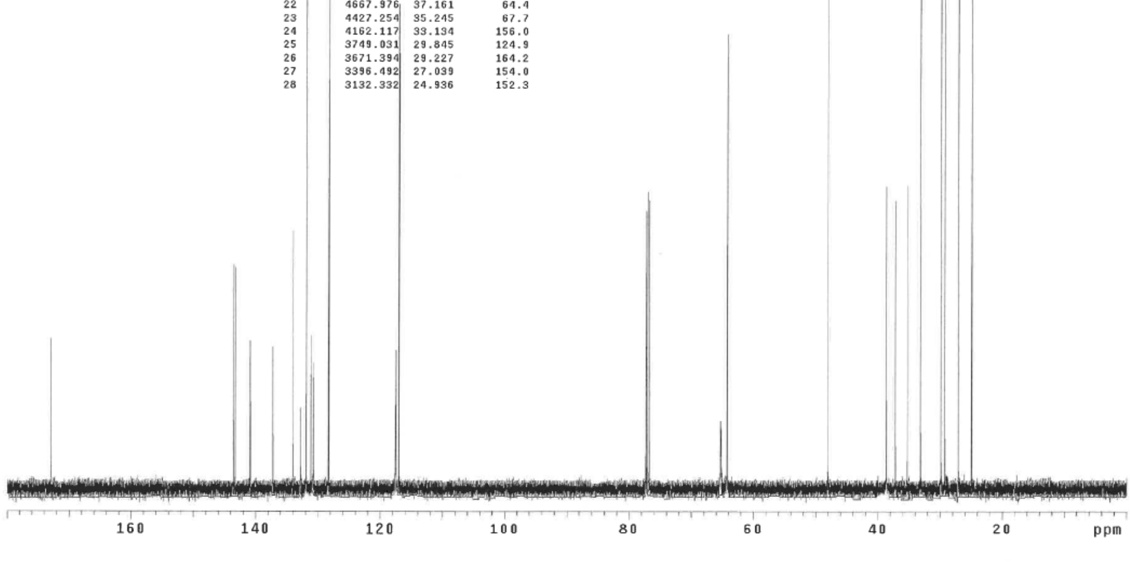

STANDARD 1H
62641
1946-BGE
Berecz Gabor
2006.03.22. (CzB)

Solvent: CDCl3
Temp. 25.0 C / 298.1 K
INOVA-500 "i500"

PULSE SEQUENCE
Relax. delay 1.000 sec
Pulse 6.4 usec
Acq. time 5.000 sec
Width 8000.0 Hz
16 repetitions
OBSERVE H1, 499.5244647 MHz
DATA PROCESSING
FT size 131072
Total time 1 minutes

INDEX	FREQUENCY	PPM	HEIGHT
1	3958.618	7.925	20.9
2	3702.148	7.411	53.5
3	3701.172	7.409	48.4
4	3632.324	7.272	4.1
5	3465.942	6.938	39.4
6	3419.556	6.846	38.6
7	2425.903	4.856	16.1
8	2107.300	4.219	130.3
9	1698.364	3.400	122.4
10	1304.321	2.611	6.4
11	1298.828	2.600	16.6
12	1296.387	2.595	21.5
13	1292.480	2.587	18.6
14	1290.405	2.583	23.4
15	1286.987	2.576	19.3
16	1281.616	2.566	6.7
17	1278.076	2.559	3.2
18	1210.205	2.423	15.1
19	1203.857	2.410	34.5
20	995.605	1.993	4.4
21	866.333	1.734	18.5
22	861.938	1.726	37.8
23	-0.000	-0.000	3.8

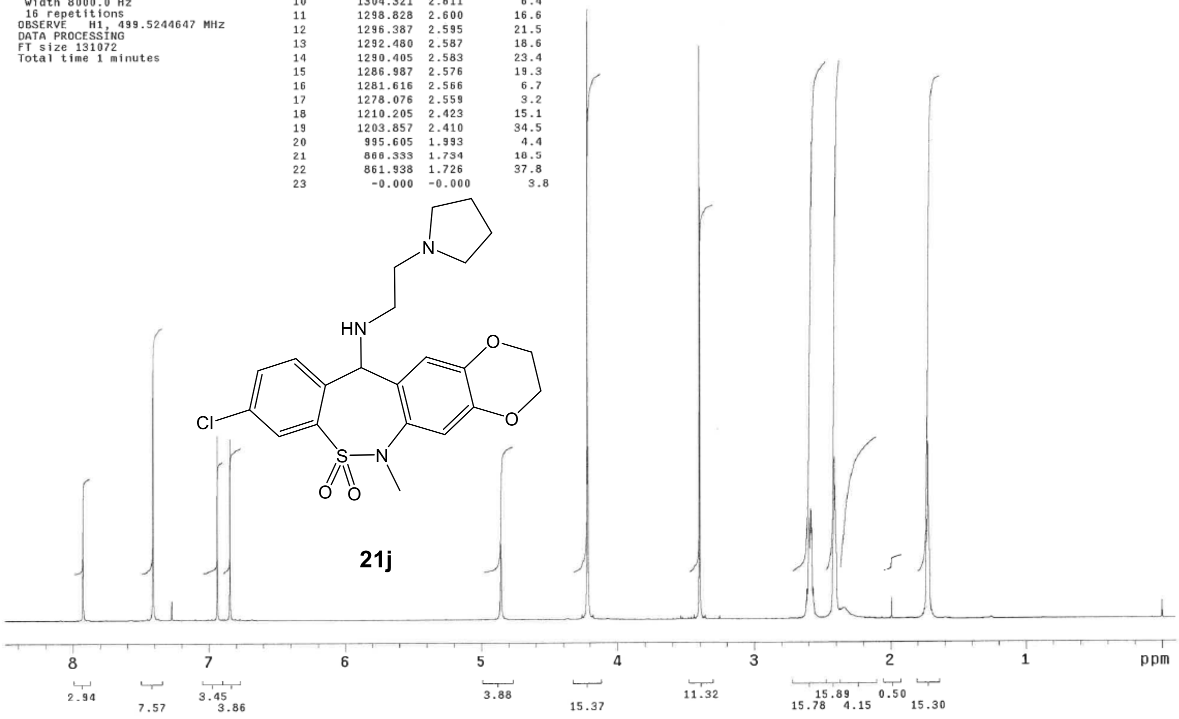

STANDARD 13C
62641
1946-BGE
Berecz Gabor
2006.03.22. (CzB)

Solvent: CDCl3
Temp. 25.0 C / 298.1 K
User: 1-14-87
INOVA-500 "i500"

PULSE SEQUENCE
Relax. delay 3.000 sec
Pulse 6.0 usec
Acq. time 1.300 sec
Width 32000.0 Hz
720 repetitions
OBSERVE C13, 125.6055357 MHz
DECOUPLE H1, 499.5269647 MHz
Power 32 dB
continuously on
WALTZ-16 modulated
DATA PROCESSING
Line broadening 0.5 Hz
FT size 131072
Total time 51 minutes

INDEX	FREQUENCY	PPM	HEIGHT
1	18037.117	143.590	35.5
2	18001.961	143.310	38.0
3	17729.012	141.137	20.6
4	17245.613	137.289	17.6
5	16837.898	134.043	36.3
6	16747.566	133.324	11.7
7	16555.672	131.797	59.0
8	16441.902	130.891	22.3
9	16416.023	130.685	12.3
10	16096.199	128.139	60.2
11	14735.848	117.309	15.7
12	14700.203	117.026	58.0
13	9704.598	77.257	65.0
14	9672.371	77.000	66.3
15	9640.633	76.747	65.3
16	8180.184	65.121	6.6
17	8072.762	64.266	55.7
18	8062.508	64.184	52.0
19	6979.500	55.563	59.8
20	6792.976	54.078	144.3
21	5869.637	46.727	57.0
22	4813.484	38.319	31.7
23	2937.996	23.389	143.5

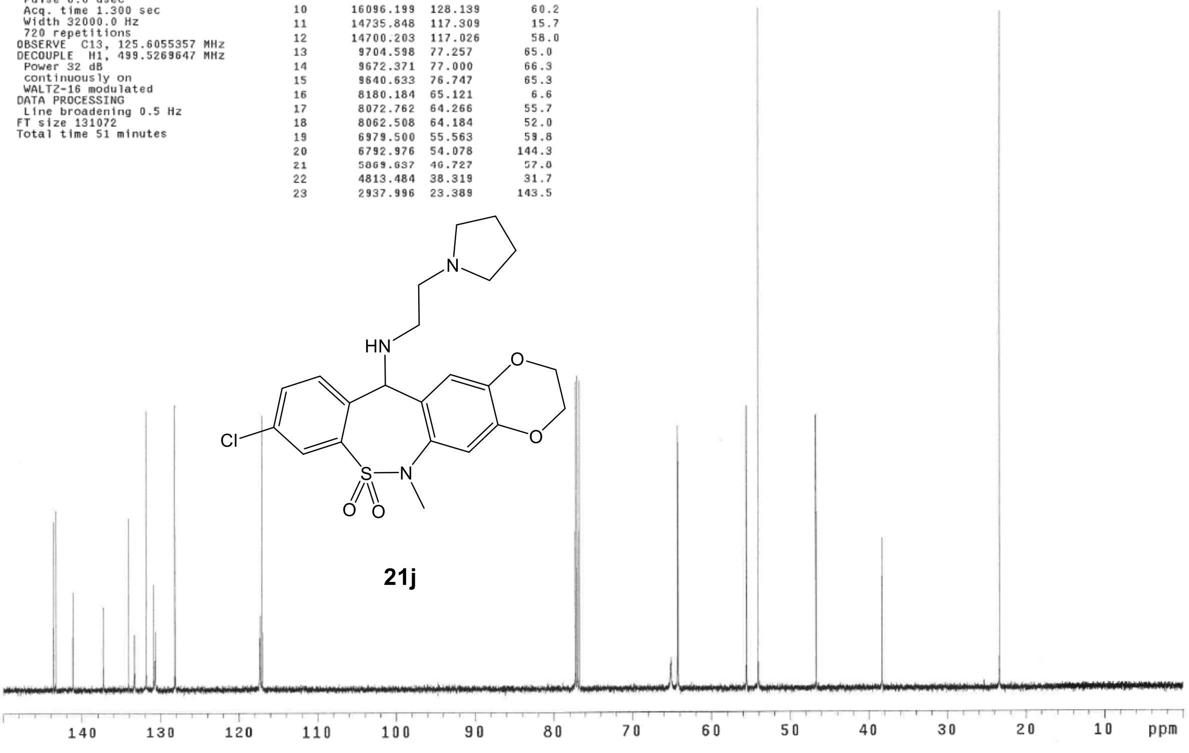

STANDARD 1H
62615
1945-BGE
Berecz Gabor
2006.03.21. (BT)
Solvent: CDCl3
Temp. 25.0 C / 298.1 K
INOVA-500 "i500"
PULSE SEQUENCE
Relax. delay 3.000 sec
Pulse 6.4 usec
Acq. time 5.000 sec
Width 8000.0 Hz
16 repetitions
OBSERVE H1, 499.5244668 MHz
DATA PROCESSING
FT size 131072
Total time 2 minutes

INDEX	FREQUENCY	PPM	HEIGHT
1	3962.158	7.932	19.6
2	3960.937	7.929	25.3
3	3702.637	7.412	46.3
4	3701.294	7.410	66.9
5	3630.737	7.268	8.1
6	3467.896	6.942	60.8
7	3416.504	6.840	52.7
8	2428.223	4.861	8.0
9	2424.438	4.853	8.1
10	2108.032	4.220	177.2
11	1705.811	3.415	177.7
12	1290.527	2.584	12.1
13	1285.522	2.573	13.5
14	1211.914	2.426	22.1
15	1205.811	2.414	33.9
16	1199.829	2.402	15.4
17	1185.913	2.374	5.1
18	1180.420	2.363	4.9
19	1141.602	2.285	16.8
20	996.338	1.995	3.6
21	772.339	1.546	7.4
22	766.602	1.535	21.5
23	760.986	1.523	32.5
24	755.493	1.512	26.3
25	750.000	1.501	9.4
26	704.956	1.411	9.0
27	700.073	1.401	9.8
28	-0.000	-0.000	9.0

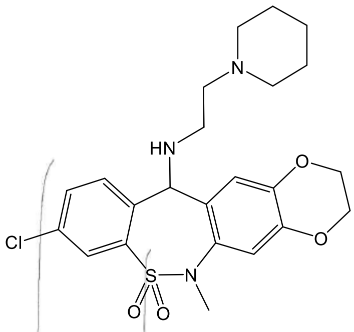

21k

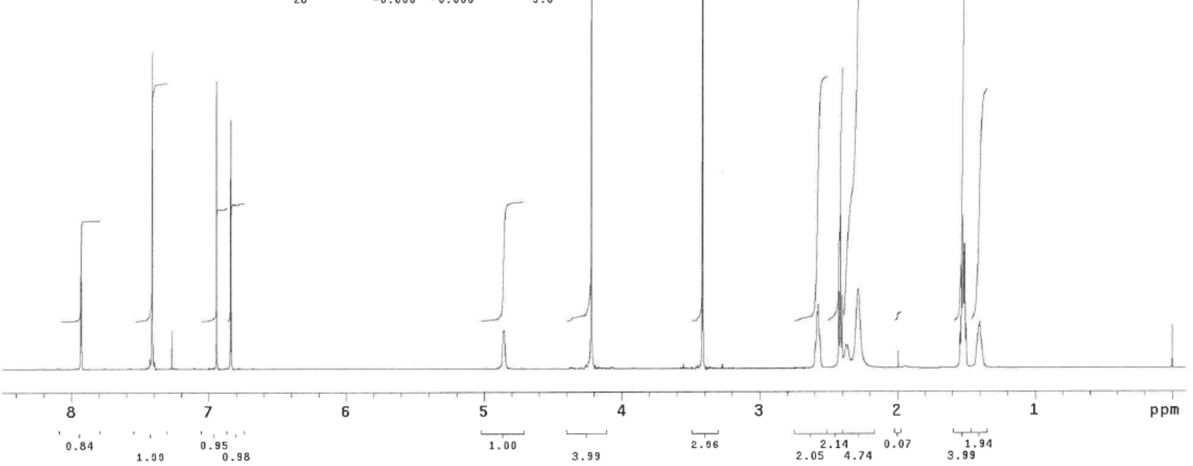

STANDARD 13C
62615
1945-BGE
Berecz Gabor
2006.03.21. (BT)
Solvent: CDCl3
Temp. 25.0 C / 298.1 K
User: 1-14-87
INOVA-500 "i500"
PULSE SEQUENCE
Relax. delay 3.000 sec
Pulse 6.0 usec
Acq. time 1.300 sec
Width 32000.0 Hz
256 repetitions
OBSERVE C13, 125.6055342 MHz
DECOUPLE H1, 499.5269647 MHz
Power 32 dB
continuously on
WALTZ-16 modulated
DATA PROCESSING
Line broadening 0.5 Hz
FT size 131072
Total time 18 minutes

INDEX	FREQUENCY	PPM	HEIGHT
1	18036.140	143.582	51.7
2	18008.797	143.365	50.9
3	17729.012	141.137	27.0
4	17257.332	137.383	22.6
5	16836.922	134.036	47.1
6	16774.422	133.538	14.1
7	16550.789	131.758	71.5
8	16440.437	130.879	29.2
9	16390.633	130.483	16.1
10	16103.523	128.197	77.6
11	14713.387	117.131	20.4
12	14707.039	117.080	76.1
13	9704.597	77.257	91.1
14	9672.371	77.000	93.5
15	9640.633	76.747	92.7
16	8145.515	64.845	7.9
17	8074.715	64.281	62.5
18	8063.484	64.192	66.5
19	7328.621	58.342	74.4
20	6870.613	54.696	136.1
21	5634.773	44.857	72.0
22	4825.203	38.413	39.9
23	3259.773	25.950	170.3
24	3059.090	24.353	86.7

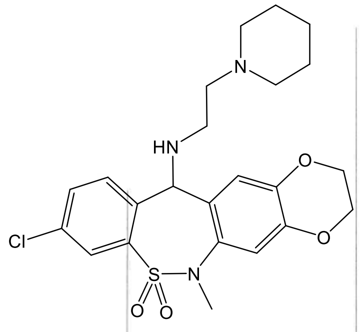

21k

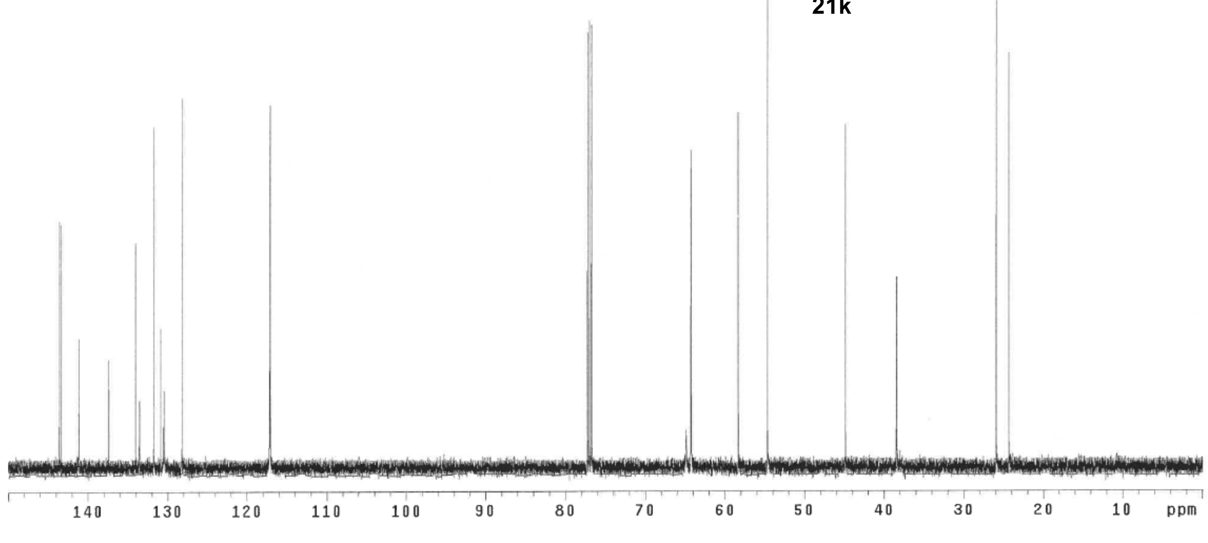

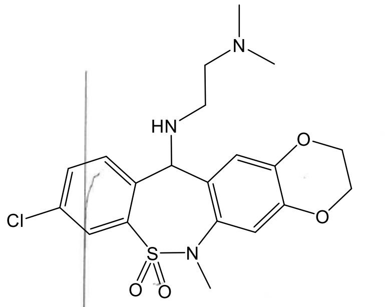

21l

STANDARD 1H
62658
1947-BGE
Berecz Gabor
2006.03.23. (CzB)
Solvent: CDCl3
Temp. 25.0 C / 298.1 K
INOVA-500 "i500"
PULSE SEQUENCE
Relax. delay 1.000 sec
Pulse 6.4 usec
Acq. time 5.000 sec
Width 8000.0 Hz
16 repetitions
OBSERVE H1, 499.5244637 MHz
DATA PROCESSING
FT size 131072
Total time 1 minutes

INDEX	FREQUENCY	PPM	HEIGHT
1	3957.764	7.923	16.5
2	3712.769	7.433	2.7
3	3704.224	7.415	37.4
4	3695.190	7.397	2.1
5	3694.092	7.395	2.1
6	3632.812	7.273	2.4
7	3468.384	6.943	26.2
8	3420.410	6.847	27.2
9	2434.570	4.874	15.5
10	2106.079	4.216	96.6
11	1703.003	3.409	82.0
12	1285.034	2.573	9.9
13	1279.297	2.561	20.4
14	1273.560	2.550	12.7
15	1206.787	2.416	1.6
16	1199.707	2.402	12.9
17	1193.848	2.390	18.6
18	1188.477	2.379	9.4
19	1181.152	2.365	1.8
20	1155.396	2.313	3.5
21	1140.015	2.282	2.5
22	1094.727	2.192	1.7
23	1074.341	2.151	151.5
24	1047.119	2.096	2.0
25	-0.000	-0.000	2.7

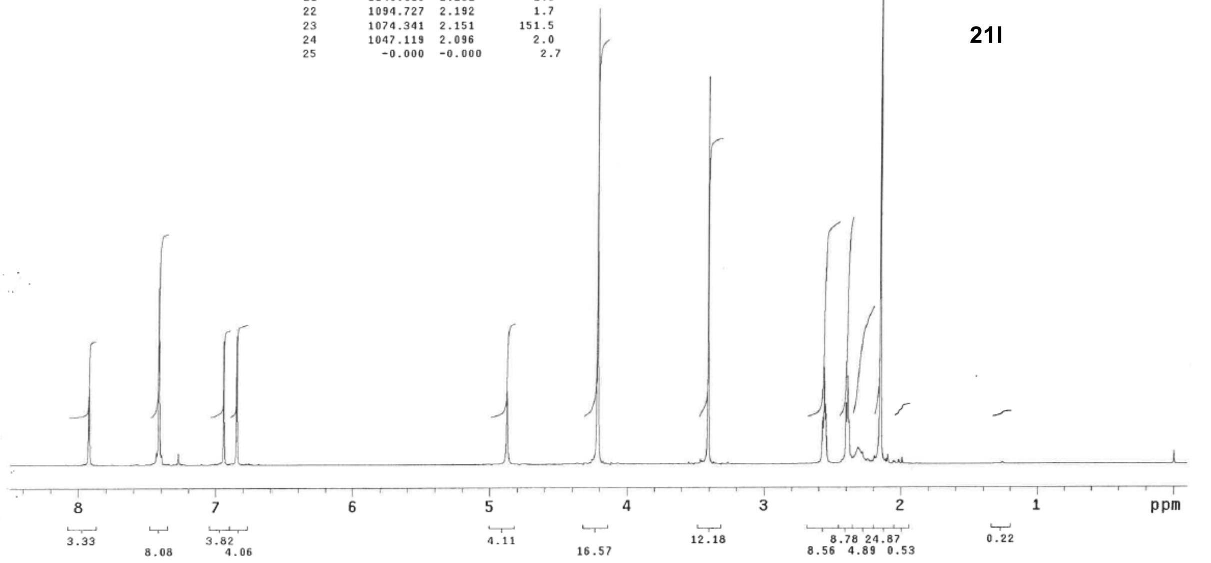

STANDARD 13C
62658
1947-BGE
Berecz Gabor
2006.03.23. (CzB)
Solvent: CDCl3
Temp. 25.0 C / 298.1 K
User: 1-14-87
INOVA-500 "i500"
PULSE SEQUENCE
Relax. delay 3.000 sec
Pulse 6.0 usec
Acq. time 1.300 sec
Width 32000.0 Hz
256 repetitions
OBSERVE C13, 125.6055362 MHz
DECOUPLE H1, 499.5269647 MHz
Power 32 dB
continuously on
WALTZ-16 modulated
DATA PROCESSING
Line broadening 0.5 Hz
FT size 131072
Total time 18 minutes

INDEX	FREQUENCY	PPM	HEIGHT
1	18034.676	143.571	41.9
2	18002.937	143.318	47.3
3	17729.500	141.141	26.9
4	17252.449	137.344	23.8
5	16835.457	134.024	42.0
6	16764.656	133.460	16.7
7	16557.137	131.808	84.8
8	16435.555	130.840	29.8
9	16391.609	130.491	17.5
10	16093.758	128.120	79.8
11	14714.363	117.138	24.6
12	14706.551	117.076	82.3
13	9704.598	77.257	89.3
14	9672.371	77.000	93.8
15	9640.633	76.747	89.4
16	8146.980	64.857	9.4
17	8072.273	64.262	76.3
18	8060.555	64.169	73.6
19	7385.750	58.797	79.4
20	5717.781	45.518	79.9
21	5704.109	45.409	200.0
22	4809.090	38.284	43.8

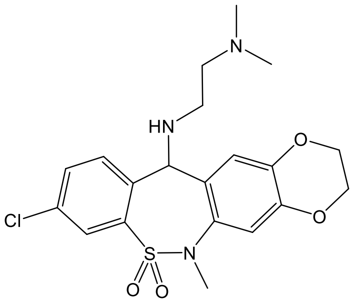

21l

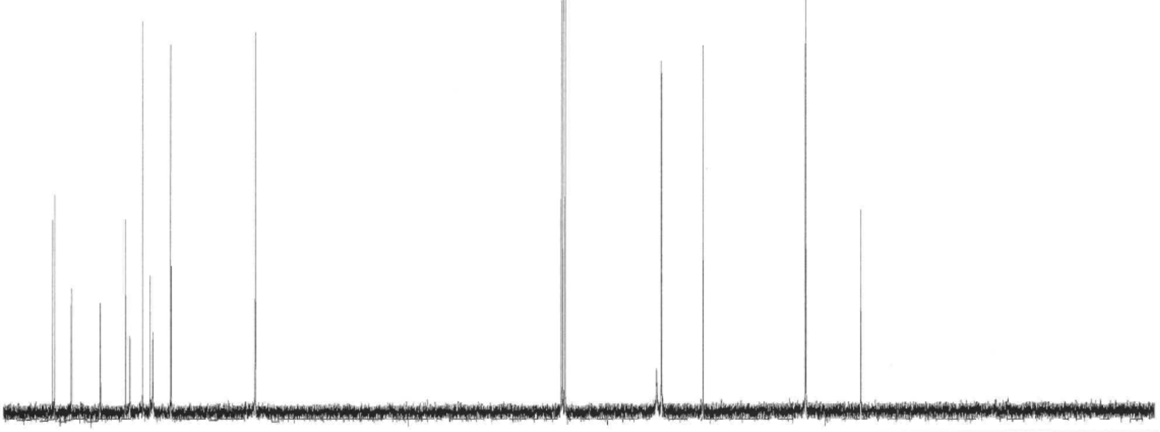

STANDARD 1H
62678
1948-BGE
Berecz Gabor
2006/03/24 (SzA)
 Solvent: CDCl3
 Temp. 25.0 C / 298.1 K
INOVA-500 "i500"
PULSE SEQUENCE
 Relax. delay 3.000 sec
 Pulse 6.4 usec
 Acq. time 5.000 sec
 Width 8000.0 Hz
 16 repetitions
OBSERVE H1, 499.5244625 MHz
DATA PROCESSING
 Line broadening 0.5 Hz
FT size 131072
Total time 2 minutes

INDEX	FREQUENCY	PPM	HEIGHT
1	3958.374	7.924	16.8
2	3705.811	7.419	40.2
3	3704.834	7.417	33.6
4	3458.496	6.924	30.8
5	3417.114	6.841	28.1
6	2446.777	4.898	12.2
7	2108.032	4.220	91.7
8	1679.932	3.363	90.0
9	1280.029	2.562	9.4
10	1273.560	2.550	9.8
11	1155.273	2.313	11.0
12	1148.193	2.299	23.1
13	1141.235	2.285	12.1
14	1113.647	2.229	9.5
15	1097.412	2.197	161.8
16	995.728	1.993	7.4
17	836.670	1.675	7.1
18	835.083	1.672	6.6
19	829.956	1.661	9.6
20	828.125	1.658	9.1
21	822.998	1.648	6.2
22	821.167	1.644	6.1

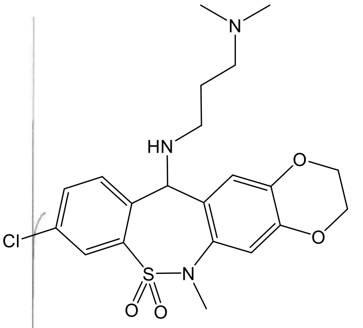

21m

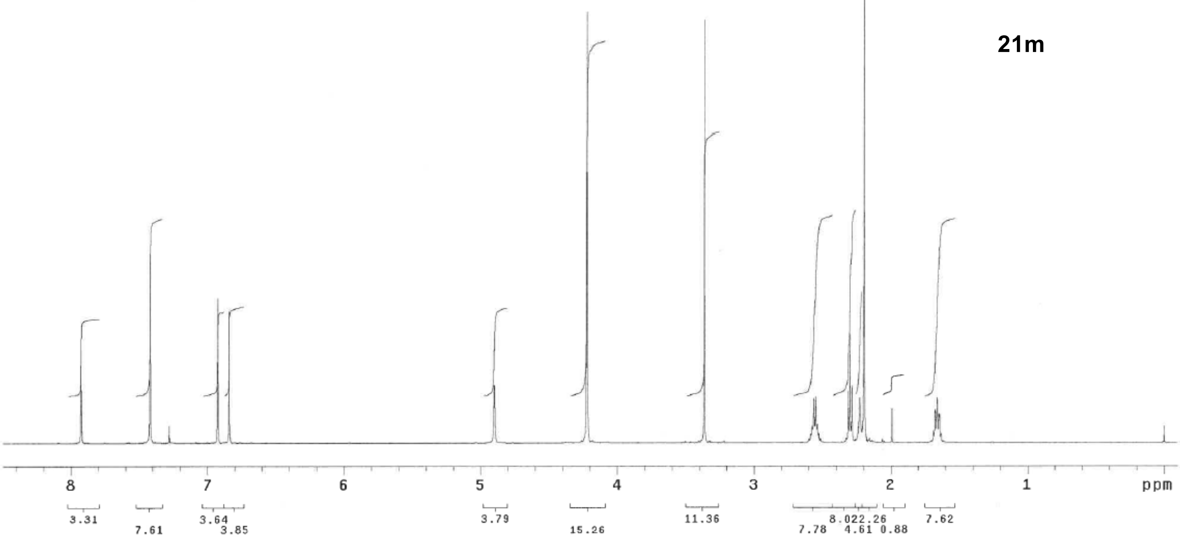

STANDARD 13C
62678
1948-BGE
Berecz Gabor
2006/03/24 (SzA)
 Solvent: CDCl3
 Temp. 25.0 C / 298.1 K
User: 1-14-87
INOVA-500 "i500"
PULSE SEQUENCE
 Relax. delay 3.000 sec
 Pulse 6.0 usec
 Acq. time 1.300 sec
 Width 32000.0 Hz
 528 repetitions
OBSERVE C13, 125.6055367 MHz
DECOUPLE H1, 499.5269647 MHz
 Power 32 dB
 continuously on
 WALTZ-16 modulated
DATA PROCESSING
 Line broadening 0.5 Hz
FT size 131072
Total time 37 minutes

INDEX	FREQUENCY	PPM	HEIGHT
1	18028.816	143.524	45.2
2	18004.402	143.330	47.5
3	17682.137	140.764	25.0
4	17261.238	137.414	18.9
5	16824.715	133.939	42.7
6	16729.012	133.177	10.6
7	16562.996	131.855	68.1
8	16442.391	130.895	24.7
9	16357.918	130.222	12.8
10	16111.824	128.263	69.0
11	14707.527	117.084	14.7
12	14686.531	116.917	70.5
13	9704.109	77.253	75.7
14	9672.371	77.000	75.4
15	9640.145	76.743	76.7
16	8071.785	64.258	62.4
17	8061.043	64.173	62.3
18	7290.535	58.039	88.1
19	5885.750	46.855	63.3
20	5713.875	45.487	162.0
21	4818.855	38.362	33.5
22	3493.172	27.809	80.6

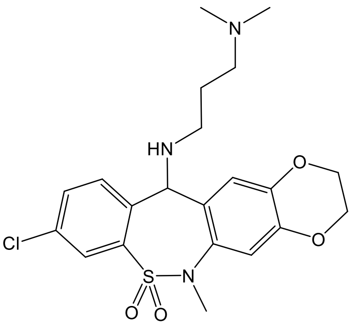

21m

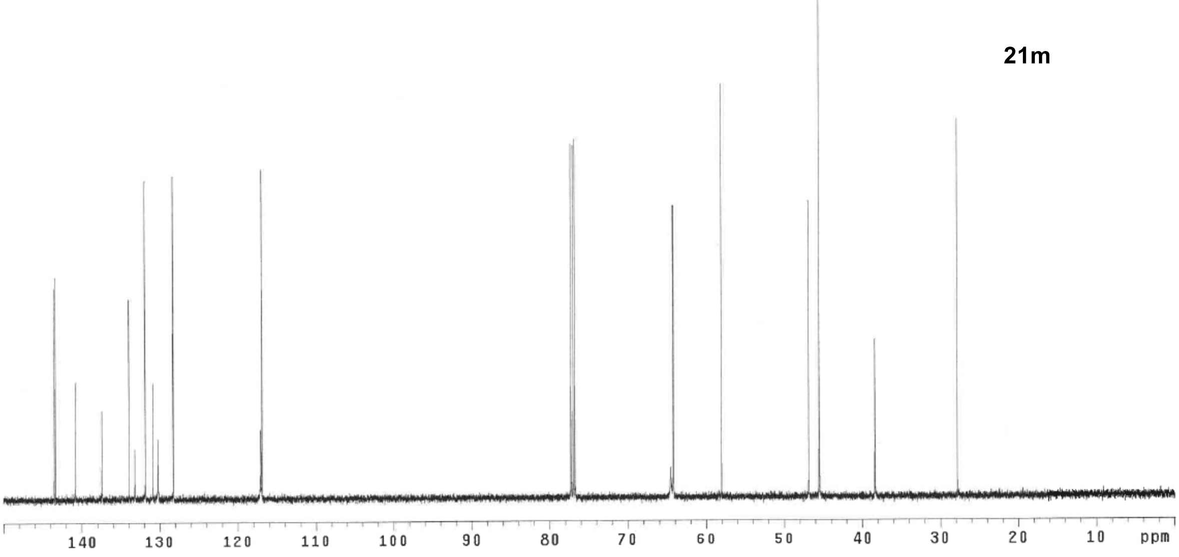

STANDARD 1H
65651
2014-BGE
Berecz Gabor
2006/10/05 (SzA)

Solvent: CDCl3
Temp. 25.0 C / 298.1 K
INOVA-500 "i500"

PULSE SEQUENCE
Relax. delay 3.000 sec
Pulse 6.4 usec
Acq. time 5.000 sec
Width 8000.0 Hz
16 repetitions
OBSERVE H1, 499.5244663 MHz
DATA PROCESSING
FT size 131072
Total time 2 minutes

INDEX	FREQUENCY	PPM	HEIGHT	INDEX	FREQUENCY	PPM	HEIGHT
1	3965.698	7.939	29.4	40	946.411	1.895	7.2
2	3963.501	7.935	28.5	41	935.059	1.872	7.8
3	3675.537	7.358	18.4	42	925.171	1.852	3.3
4	3673.218	7.353	17.6	43	923.706	1.849	3.3
5	3667.236	7.341	24.0	44	912.109	1.826	7.1
6	3664.917	7.337	22.9	45	910.645	1.823	7.0
7	3630.615	7.268	17.9	46	908.569	1.819	6.7
8	3612.183	7.231	26.2	47	903.320	1.808	5.0
9	3603.882	7.215	20.2	48	898.804	1.799	9.5
10	3486.450	6.980	63.2	49	897.339	1.796	9.3
11	3368.652	6.744	57.7	50	835.693	1.673	3.2
12	2127.197	4.258	7.3	51	832.642	1.667	3.5
13	2124.268	4.253	5.0	52	820.801	1.643	22.5
14	2122.192	4.248	7.3	53	811.279	1.624	6.4
15	2120.972	4.246	6.2	54	619.629	1.240	57.0
16	2115.601	4.235	24.3	55	612.549	1.226	122.3
17	2114.014	4.232	12.5	56	605.347	1.212	54.1
18	2112.305	4.229	22.8	57	1.099	0.002	3.3
19	2110.962	4.226	30.4	58	0.000	0.000	22.3
20	2108.765	4.222	18.8				
21	2106.445	4.217	22.4				
22	2105.347	4.215	17.4				
23	2099.976	4.204	8.6				
24	2097.290	4.199	5.0				
25	2094.604	4.193	7.0				
26	2062.012	4.128	14.8				
27	2054.932	4.114	47.5				
28	2047.729	4.099	48.6				
29	2040.649	4.085	15.2				
30	1973.145	3.950	22.7				
31	1744.507	3.492	197.3				
32	1326.660	2.656	3.2				
33	1315.796	2.634	3.4				
34	1238.892	2.480	3.7				
35	1227.783	2.458	4.0				
36	1135.986	2.274	3.4				
37	1131.958	2.266	5.8				
38	1127.930	2.258	3.5				
39	999.146	2.000	7.1				

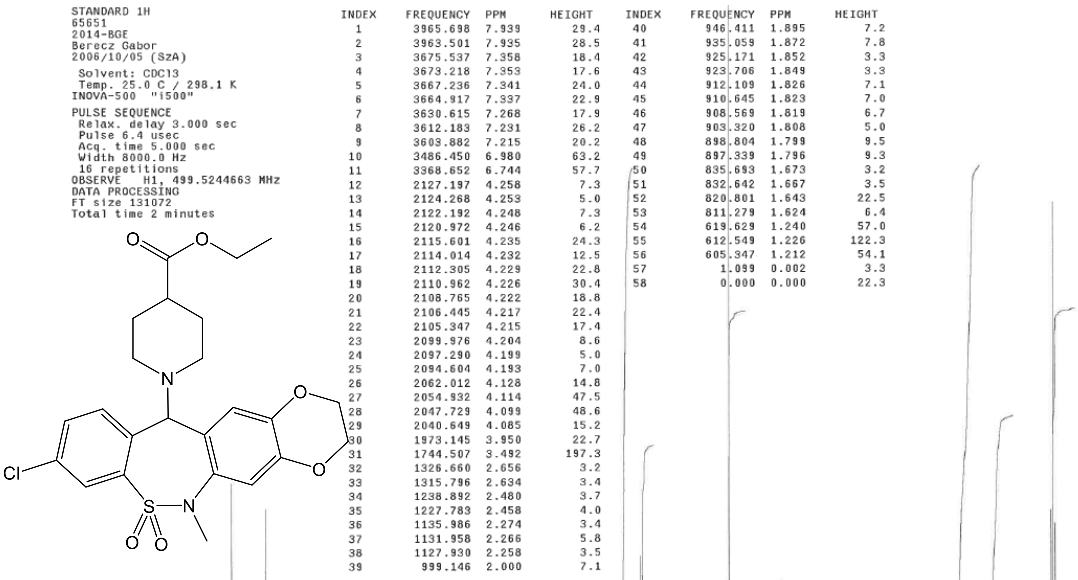

21n

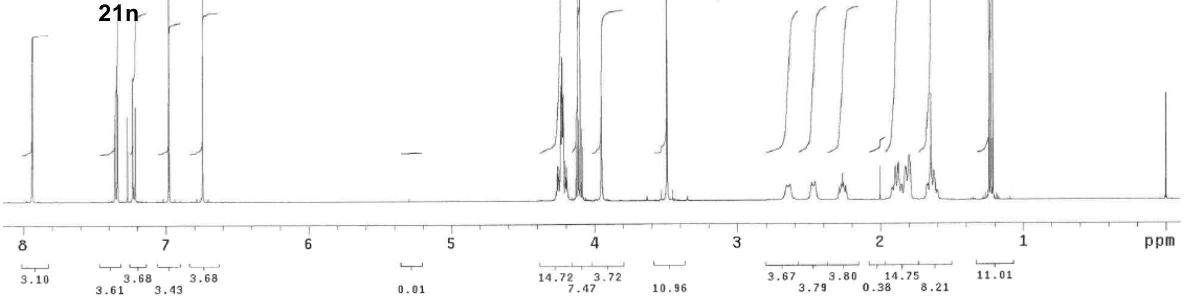

STANDARD 13C
65651
2014-BGE
Berecz Gabor
2006/10/05 (SzA)

Solvent: CDCl3
Temp. 25.0 C / 298.1 K
User: 1-14-87
INOVA-500 "i500"

PULSE SEQUENCE
Relax. delay 3.000 sec
Pulse 6.0 usec
Acq. time 1.300 sec
Width 32000.0 Hz
666 repetitions
OBSERVE C13, 125.6055323 MHz
DECOUPLE H1, 499.5269647 MHz
Power 32 dB
continuously on
WALTZ-16 modulated
DATA PROCESSING
Line broadening 0.5 Hz
FT size 131072
Total time 47 minutes

INDEX	FREQUENCY	PPM	HEIGHT
1	21956.062	174.788	43.2
2	18088.875	144.002	42.1
3	18021.980	143.470	49.7
4	17983.406	143.163	13.8
5	16969.734	135.093	34.9
6	16883.308	134.405	15.2
7	16783.699	133.612	47.7
8	16765.632	133.468	21.9
9	16457.527	131.015	59.7
10	16366.707	130.292	31.9
11	16169.929	128.726	64.6
12	15029.793	119.649	43.4
13	14948.738	119.004	34.0
14	9704.597	77.257	115.6
15	9672.371	77.000	119.0
16	9640.632	76.747	119.2
17	8076.179	64.293	56.4
18	8057.625	64.145	55.7
19	7580.574	60.348	55.8
20	6560.554	52.227	19.7
21	6533.211	52.010	29.6
22	5116.707	40.733	9.9
23	5076.179	40.411	26.9
24	3560.554	28.345	32.4
25	3509.773	27.941	49.0
26	1777.351	14.149	72.2

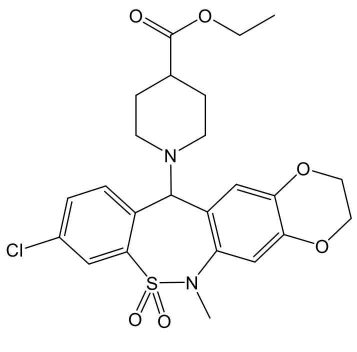

21n

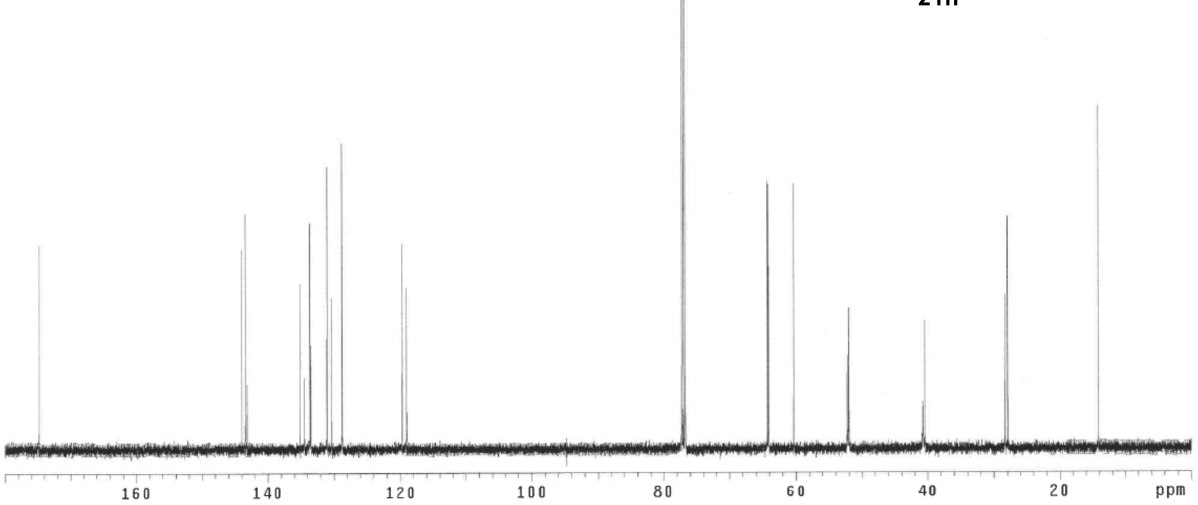

STANDARD 1H
65479
2009-BGE
Berecz Gabor
2006.09.25. (BT)

Solvent: DMSO
Temp. 25.0 C / 298.1 K
INOVA-500 "i500"

PULSE SEQUENCE
Relax. delay 3.000 sec
Pulse 6.4 usec
Acq. time 5.000 sec
Width 8000.0 Hz
16 repetitions
OBSERVE H1, 499.5268306 MHz
DATA PROCESSING
FT size 131072
Total time 2 minutes

INDEX	FREQUENCY	PPM	HEIGHT	INDEX	FREQUENCY	PPM	HEIGHT
1	3882.568	7.772	34.1	40	898.804	1.799	5.3
2	3880.249	7.768	35.0	41	896.851	1.795	4.4
3	3821.045	7.649	19.3	42	809.937	1.621	9.8
4	3818.848	7.645	17.4	43	798.584	1.599	12.8
5	3812.744	7.633	26.1	44	735.474	1.472	5.1
6	3810.425	7.628	25.2	45	733.032	1.467	5.6
7	3767.456	7.542	32.5	46	723.267	1.448	6.6
8	3758.911	7.525	23.8	47	710.205	1.422	6.6
9	3600.586	7.208	14.6	48	700.562	1.402	4.8
10	3513.550	7.034	75.3	49	697.876	1.397	4.4
11	3474.976	6.957	65.7				
12	3362.427	6.731	14.3				
13	2141.479	4.287	7.9				
14	2138.306	4.281	6.1				
15	2135.376	4.275	8.3				
16	2129.395	4.263	34.6				
17	2124.756	4.254	44.5				
18	2122.681	4.249	53.8				
19	2116.333	4.237	7.5				
20	2113.525	4.231	5.8				
21	2111.450	4.227	5.9				
22	2108.521	4.221	7.3				
23	1710.937	3.425	165.7				
24	1670.776	3.345	82.0				
25	1275.513	2.553	6.3				
26	1263.916	2.530	6.7				
27	1255.127	2.513	6.3				
28	1253.296	2.509	8.2				
29	1251.465	2.505	5.6				
30	1160.034	2.322	6.3				
31	1148.926	2.300	6.9				
32	1024.658	2.051	4.8				
33	1020.874	2.044	7.9				
34	1017.090	2.036	4.7				
35	1009.277	2.020	4.3				
36	927.124	1.856	5.1				
37	917.725	1.837	9.9				
38	910.400	1.823	9.6				
39	907.959	1.818	10.4				

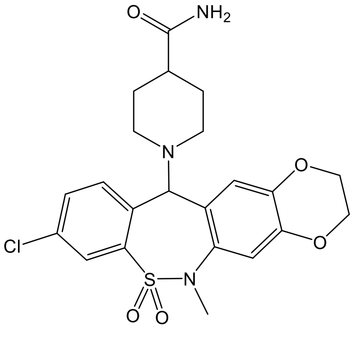

21o

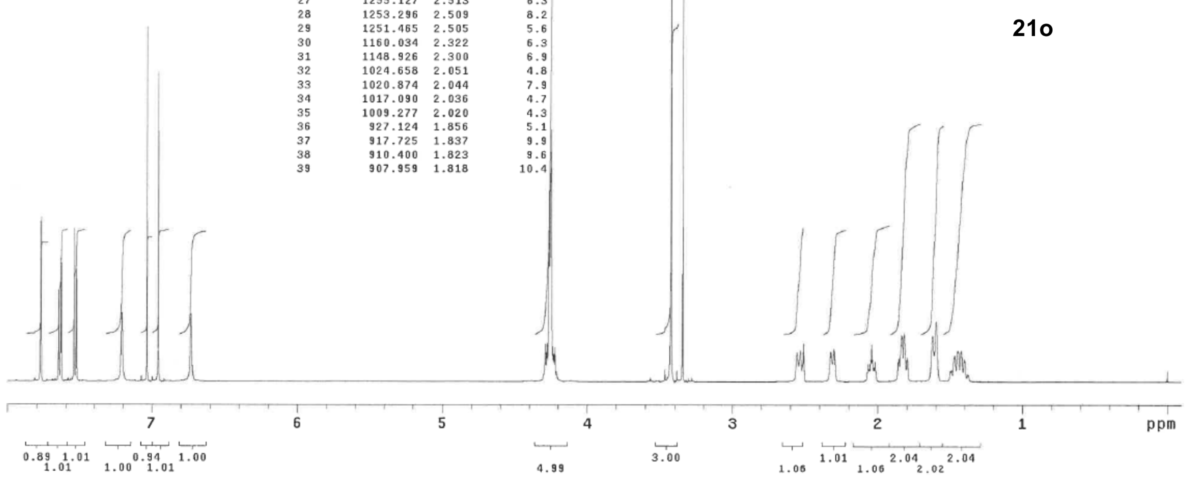

STANDARD 13C
65479
2009-BGE
Berecz Gabor
2006.09.25. (BT)

Solvent: DMSO
Temp. 25.0 C / 298.1 K
User: 1-14-87
INOVA-500 "i500"

PULSE SEQUENCE
Relax. delay 3.000 sec
Pulse 6.0 usec
Acq. time 1.300 sec
Width 32000.0 Hz
512 repetitions
OBSERVE C13, 125.6061592 MHz
DECOUPLE H1, 499.5293374 MHz
Power 32 dB
continuously on
WALTZ-16 modulated
DATA PROCESSING
Line broadening 0.5 Hz
FT size 131072
Total time 36 minutes

INDEX	FREQUENCY	PPM	HEIGHT
1	22175.239	176.539	97.7
2	18057.563	143.758	78.3
3	18011.177	143.388	77.2
4	18008.735	143.369	60.6
5	16972.602	135.120	55.6
6	16956.001	134.988	44.7
7	16814.399	133.861	64.6
8	16789.009	133.659	71.6
9	16509.223	131.431	56.8
10	16326.118	129.973	79.1
11	16005.805	127.423	57.3
12	15000.434	119.420	60.3
13	14967.720	119.159	54.0
14	9372.016	74.611	45.1
15	8075.630	64.291	46.9
16	8067.329	64.225	48.0
17	6541.450	52.077	39.8
18	6503.852	51.778	42.1
19	5222.602	41.578	33.4
20	5059.516	40.279	75.8
21	5050.239	40.205	51.1
22	5029.243	40.038	161.5
23	5007.759	39.867	323.7
24	4987.251	39.704	382.3
25	4965.766	39.533	327.1
26	4944.770	39.366	163.7
27	4923.774	39.199	53.1
28	3617.134	28.796	43.3
29	3558.540	28.330	46.4

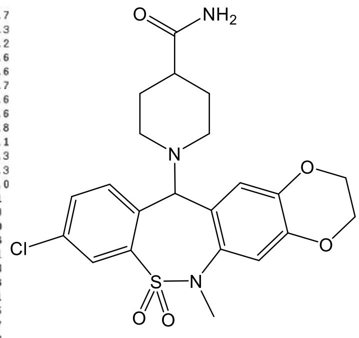

21o

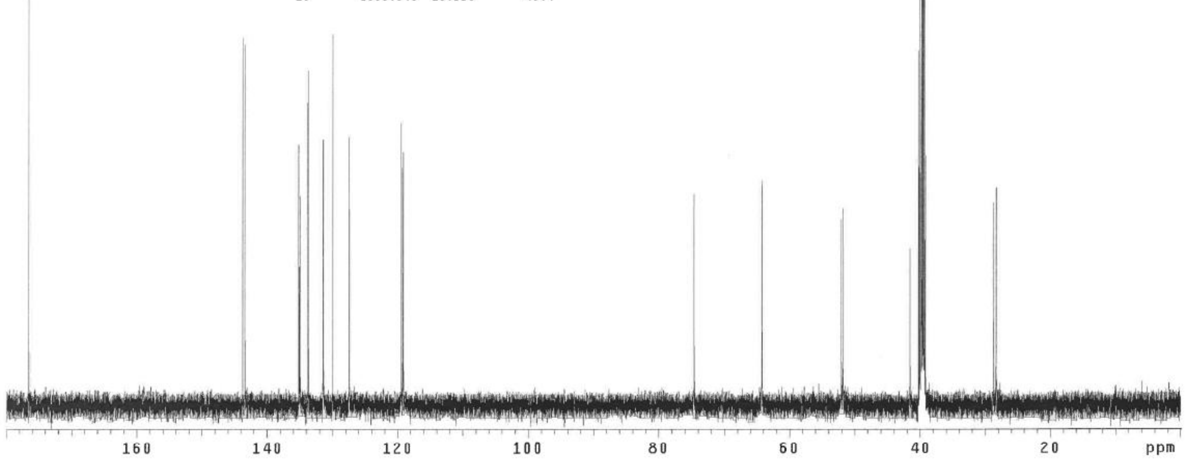

STANDARD 1H
67546
2044-BGE
Berecz Gabor
2007.02.07. (BB)
Solvent: CDCl3
Temp. 25.0 C / 298.1 K
INOVA-500 "i500"
PULSE SEQUENCE
Relax. delay 1.000 sec
Pulse 6.4 usec
Acq. time 5.000 sec
Width 8000.0 Hz
16 repetitions
OBSERVE H1, 499.5244648 MHz
DATA PROCESSING
FT size 131072
Total time 1 minutes

INDEX	FREQUENCY	PPM	HEIGHT
1	3966.675	7.941	25.3
2	3964.478	7.937	24.9
3	3686.768	7.381	14.5
4	3684.570	7.376	14.1
5	3678.467	7.364	18.6
6	3676.270	7.360	17.9
7	3632.080	7.271	6.0
8	3619.995	7.247	26.2
9	3611.694	7.230	20.7
10	3487.793	6.982	53.8
11	3372.681	6.752	53.2
12	2131.104	4.266	1.9
13	2125.732	4.256	7.6
14	2122.559	4.249	6.4
15	2120.728	4.245	9.2
16	2113.892	4.232	29.3
17	2109.863	4.224	37.6
18	2105.469	4.215	28.5
19	2099.121	4.202	8.9
20	2096.558	4.197	5.6
21	2093.628	4.191	7.4
22	2003.906	4.012	27.4
23	1736.328	3.476	3.0
24	1729.370	3.462	167.9
25	1324.951	2.652	3.8
26	1233.398	2.469	3.4
27	1185.181	2.373	4.9
28	1116.455	2.235	2.2
29	1065.063	2.132	3.1
30	910.767	1.823	10.1
31	904.663	1.811	10.3
32	897.461	1.797	10.5
33	893.677	1.789	10.3
34	890.137	1.782	9.9
35	886.597	1.775	9.7
36	880.005	1.762	5.9
37	876.709	1.755	4.8
38	873.169	1.748	3.6
39	832.886	1.667	10.7
40	-0.000	-0.000	6.4

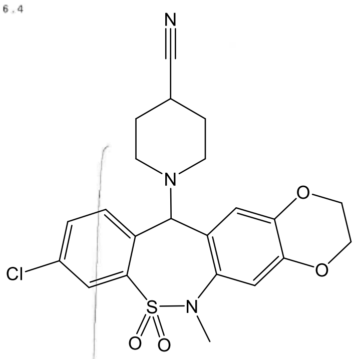

21p

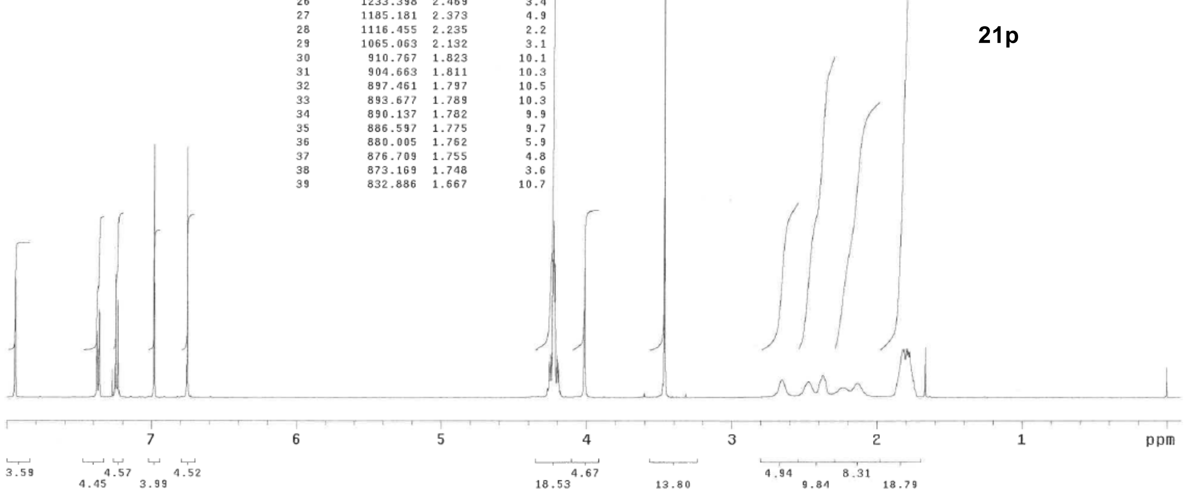

STANDARD 13C
67546
2044-BGE
Berecz Gabor
2007.02.07. (BB)
Solvent: CDCl3
Temp. 25.0 C / 298.1 K
User: 1-14-87
INOVA-500 "i500"
PULSE SEQUENCE
Relax. delay 3.000 sec
Pulse 6.0 usec
Acq. time 1.300 sec
Width 32000.0 Hz
400 repetitions
OBSERVE C13, 125.6055381 MHz
DECOUPLE H1, 499.5269647 MHz
Power 32 dB
continuously on
WALTZ-16 modulated
DATA PROCESSING
Line broadening 0.5 Hz
FT size 131072
Total time 28 minutes

INDEX	FREQUENCY	PPM	HEIGHT
1	18106.942	144.146	73.3
2	18030.281	143.536	82.2
3	17972.664	143.077	32.6
4	16994.149	135.287	61.4
5	16809.090	133.814	34.4
6	16770.516	133.507	46.1
7	16689.949	132.866	63.3
8	16477.059	131.171	108.4
9	16346.688	130.133	51.8
10	16169.930	128.726	112.5
11	15235.848	121.290	36.5
12	15012.215	119.510	90.6
13	14956.063	119.063	67.9
14	9704.598	77.257	131.3
15	9672.371	77.000	132.9
16	9640.633	76.747	133.3
17	9588.875	76.335	28.7
18	8070.320	64.246	96.0
19	8054.695	64.122	97.2
20	6318.367	50.299	18.1
21	5036.629	40.096	57.9
22	3594.246	28.613	61.0
23	3242.684	25.814	25.3

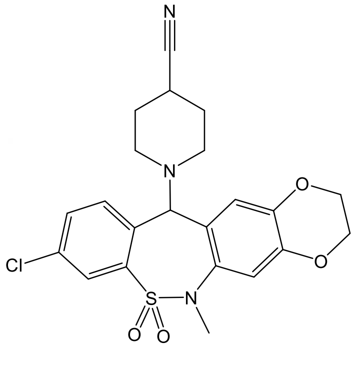

21p

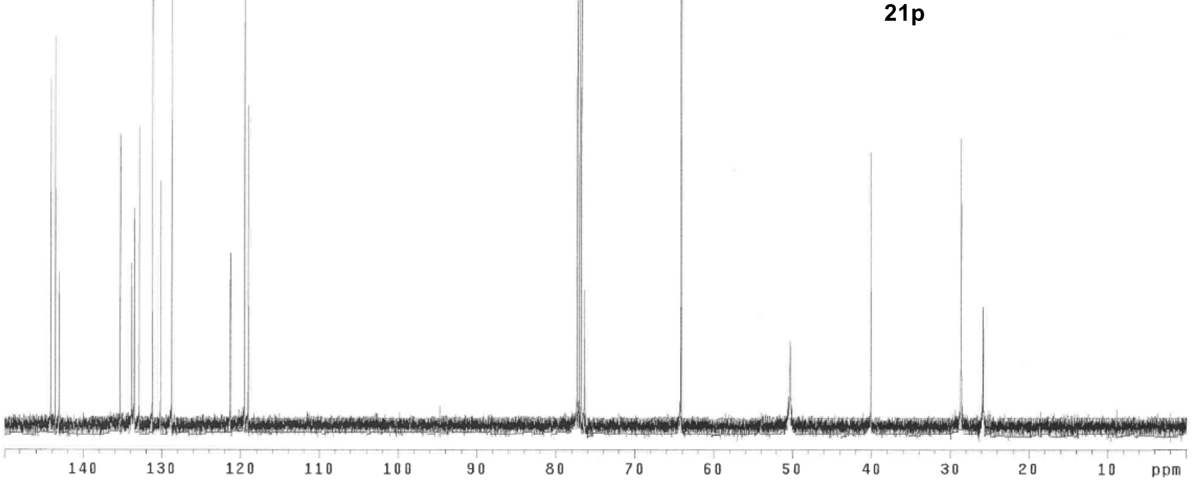

STANDARD 1H
68717
2068-BGE
Berecz Gabor
2007.04.10. (BB)
Solvent: CDCl3
Temp. 25.0 C / 298.1 K
INOVA-500 "i500"
PULSE SEQUENCE
Relax. delay 1.000 sec
Pulse 6.4 usec
Acq. time 5.000 sec
Width 8000.0 Hz
16 repetitions
OBSERVE H1, 499.5244691 MHz
DATA PROCESSING
FT size 131072
Total time 1 minutes

INDEX	FREQUENCY	PPM	HEIGHT
1	3950.317	7.908	16.9
2	3948.242	7.904	16.9
3	3787.598	7.582	17.6
4	3779.175	7.566	22.0
5	3720.581	7.448	14.2
6	3718.384	7.444	13.8
7	3712.036	7.431	11.0
8	3709.961	7.427	10.8
9	3626.953	7.261	4.8
10	3491.333	6.989	41.3
11	3450.195	6.907	39.7
12	2743.774	5.493	31.3
13	2119.507	4.243	4.9
14	2113.159	4.230	135.2
15	2106.812	4.218	4.9
16	1726.074	3.455	152.1
17	1700.439	3.404	153.4
18	997.070	1.996	1.7
19	797.729	1.597	3.2
20	0.000	0.000	4.5

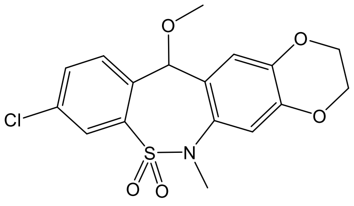

23a

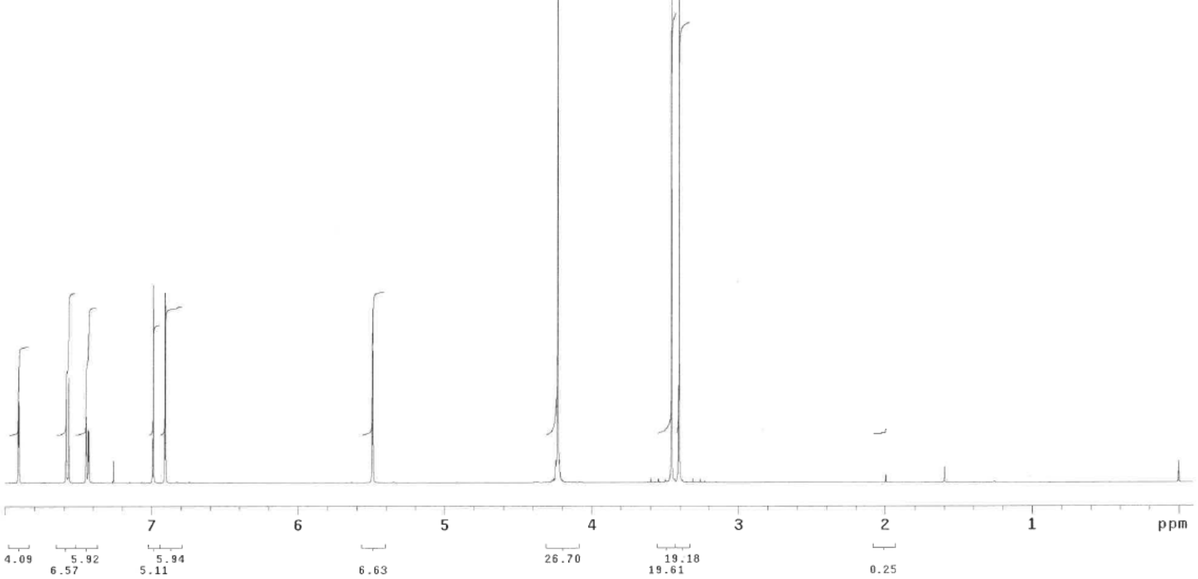

STANDARD 13C
68717
2068-BGE
Berecz Gabor
2007.04.10. (BB)
Solvent: DMSO
Temp. 25.0 C / 298.1 K
User: 1-14-87
INOVA-500 "i500"
PULSE SEQUENCE
Relax. delay 3.000 sec
Pulse 6.0 usec
Acq. time 1.300 sec
Width 32000.0 Hz
318 repetitions
OBSERVE C13, 125.6055317 MHz
DECOUPLE H1, 499.5293374 MHz
Power 32 dB
continuously on
WALTZ-16 modulated
DATA PROCESSING
Line broadening 1.0 Hz
FT size 131072
Total time 22 minutes

INDEX	FREQUENCY	PPM	HEIGHT
1	18079.597	143.928	46.8
2	18055.183	143.734	51.9
3	17638.679	140.418	38.1
4	17063.972	135.843	43.1
5	16903.816	134.568	47.5
6	16691.414	132.877	50.6
7	16564.949	131.871	107.0
8	16313.972	129.873	42.7
9	16145.027	128.528	62.5
10	16079.109	128.003	118.0
11	14763.191	117.527	96.0
12	14491.218	115.362	70.9
13	10113.777	80.514	50.2
14	9704.109	77.253	139.9
15	9672.371	77.000	139.8
16	9640.144	76.743	139.3
17	8078.132	64.309	101.5
18	8063.484	64.192	99.4
19	7265.632	57.840	51.0
20	7267.097	57.852	52.4
21	4773.445	38.001	43.1

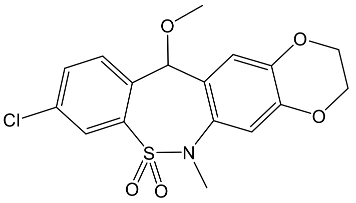

23a

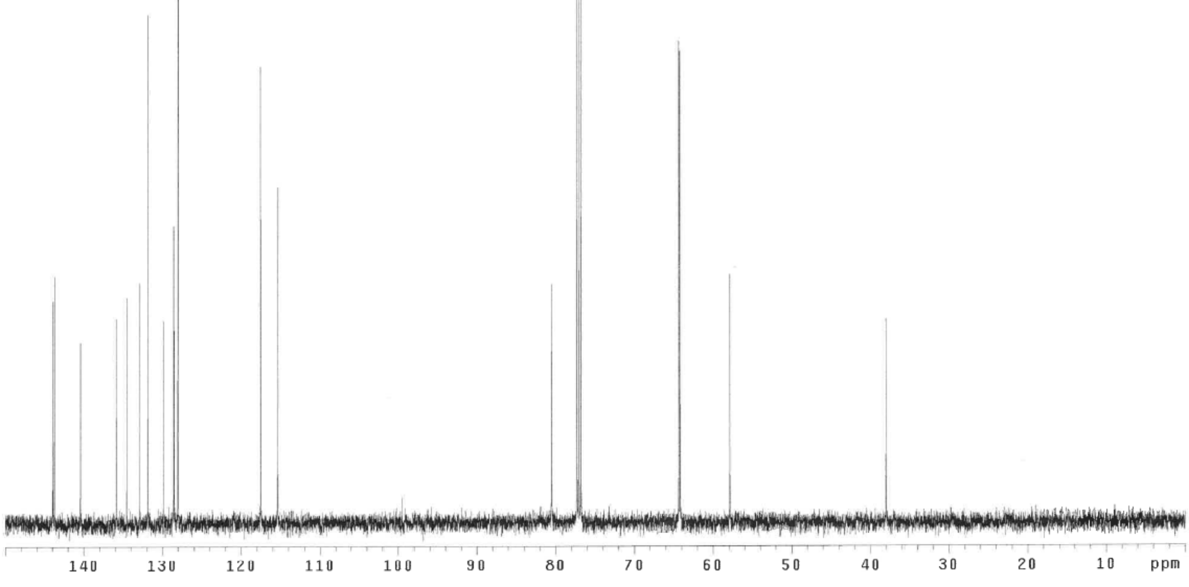

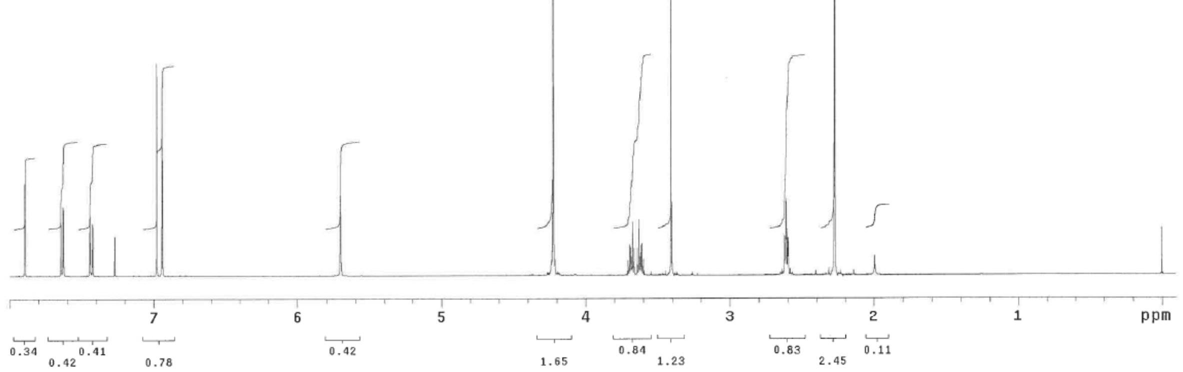

* STANDARD 1H
62848
1950-BGE
Berecz Gabor
2006/04/04 (SzA)

Solvent: CDCl3
Temp. 25.0 C / 298.1 K
INOVA-500 "i500"

PULSE SEQUENCE
Relax. delay 3.000 sec
Pulse 6.4 usec
Acq. time 5.000 sec
Width 8000.0 Hz
16 repetitions
OBSERVE H1, 499.5244667 MHz
DATA PROCESSING
FT size 131072
Total time 2 minutes

INDEX	FREQUENCY	PPM	HEIGHT
1	3944.336	7.896	19.7
2	3942.139	7.892	19.8
3	3818.237	7.644	12.7
4	3809.814	7.627	14.7
5	3717.285	7.442	13.1
6	3715.088	7.437	12.7
7	3708.862	7.425	11.2
8	3706.665	7.420	11.0
9	3630.615	7.268	8.4
10	3485.718	6.978	45.6
11	3467.163	6.941	32.9
12	2848.633	5.703	18.6
13	2110.474	4.225	89.5
14	1846.069	3.696	6.5
15	1842.041	3.688	6.1
16	1840.210	3.684	3.9
17	1836.182	3.676	11.4
18	1830.322	3.664	5.7
19	1819.336	3.642	5.8
20	1813.354	3.630	11.9
21	1809.448	3.622	4.0
22	1807.373	3.618	6.4
23	1803.589	3.611	6.8
24	1701.538	3.406	144.2
25	1311.401	2.625	8.5
26	1309.204	2.621	8.3
27	1305.420	2.613	15.8
28	1303.345	2.609	16.3
29	1299.561	2.602	8.0
30	1297.363	2.597	8.0
31	1136.719	2.276	244.7
32	997.070	1.996	4.3
33	0.000	0.000	10.0

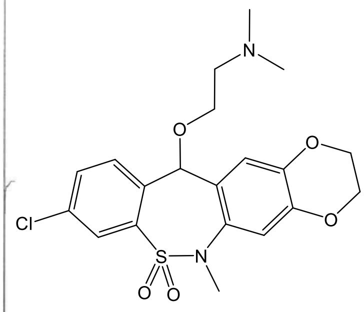

23b

STANDARD 13C
62848
1950-BGE
Berecz Gabor
2006/04/04 (SzA)

Solvent: CDCl3
Temp. 25.0 C / 298.1 K
User: 1-14-87
INOVA-500 "i500"

PULSE SEQUENCE
Relax. delay 3.000 sec
Pulse 6.0 usec
Acq. time 1.300 sec
Width 32000.0 Hz
704 repetitions
OBSERVE C13, 125.6055328 MHz
DECOUPLE H1, 499.5269647 MHz
Power 32 dB
continuously on
WALTZ-16 modulated
DATA PROCESSING
Line broadening 0.5 Hz
FT size 131072
Total time 50 minutes

INDEX	FREQUENCY	PPM	HEIGHT
1	18067.390	143.831	40.8
2	18054.207	143.726	43.0
3	17622.078	140.286	22.1
4	17110.847	136.216	21.0
5	16884.285	134.413	33.8
6	16752.937	133.367	31.7
7	16564.461	131.867	62.6
8	16290.535	129.686	25.1
9	16119.636	128.326	19.8
10	16070.808	127.937	64.8
11	14738.289	117.329	52.0
12	14464.363	115.148	27.1
13	9881.843	78.668	13.9
14	9704.597	77.257	103.2
15	9672.371	77.000	106.9
16	9640.633	76.747	103.6
17	8617.683	68.604	57.8
18	8075.691	64.289	61.7
19	8061.531	64.176	56.6
20	7402.840	58.933	78.4
21	5781.258	46.024	177.9
22	4756.355	37.864	40.8

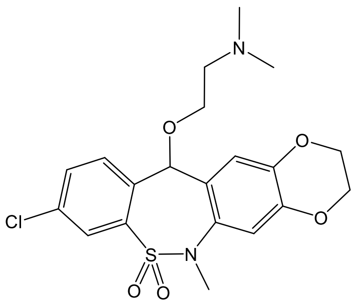

23b

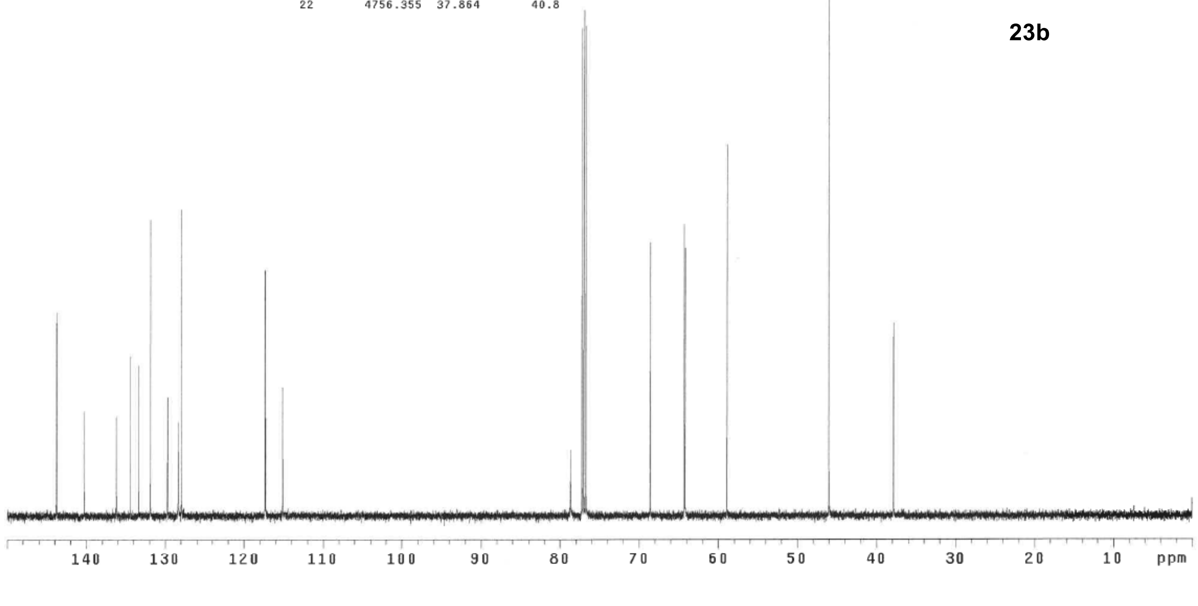

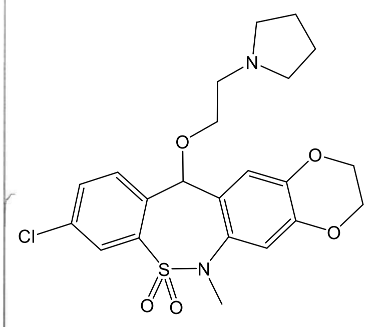

23c

* STANDARD 1H
62848
1950-BGE
Berecz Gabor
2006/04/04 (SzA)

Solvent: CDCl3
Temp. 25.0 C / 298.1 K
INOVA-500 "i500"

PULSE SEQUENCE
Relax. delay 3.000 sec
Pulse 6.4 usec
Acq. time 5.000 sec
Width 8000.0 Hz
16 repetitions
OBSERVE H1, 499.5244667 MHz
DATA PROCESSING
FT size 131072
Total time 2 minutes

INDEX	FREQUENCY	PPM	HEIGHT
1	3944.336	7.896	19.7
2	3942.139	7.892	19.8
3	3818.237	7.644	12.7
4	3809.814	7.627	14.7
5	3717.285	7.442	13.1
6	3715.088	7.437	12.7
7	3708.862	7.425	11.2
8	3706.665	7.420	11.0
9	3630.615	7.268	8.4
10	3485.718	6.978	45.6
11	3467.163	6.941	32.9
12	2848.633	5.703	18.6
13	2110.474	4.225	89.5
14	1846.069	3.696	6.5
15	1842.041	3.688	6.1
16	1840.210	3.684	3.9
17	1836.182	3.676	11.4
18	1830.322	3.664	5.7
19	1819.336	3.642	5.8
20	1813.354	3.630	11.9
21	1809.448	3.622	4.0
22	1807.373	3.618	6.4
23	1803.589	3.611	6.8
24	1701.538	3.406	144.2
25	1311.401	2.625	8.5
26	1309.204	2.621	8.3
27	1305.420	2.613	15.8
28	1303.345	2.609	16.3
29	1299.561	2.602	8.0
30	1297.363	2.597	8.0
31	1136.719	2.276	244.7
32	997.070	1.996	4.3
33	0.000	0.000	10.0

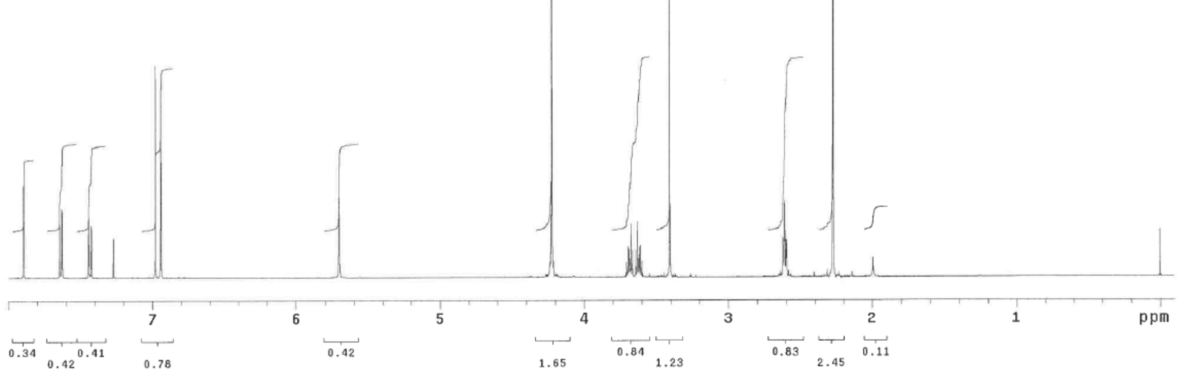

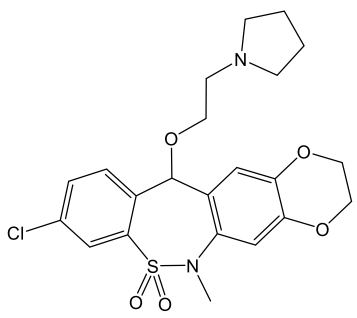

23c

STANDARD 13C
62848
1950-BGE
Berecz Gabor
2006/04/04 (SzA)

Solvent: CDCl3
Temp. 25.0 C / 298.1 K
User: 1-14-87
INOVA-500 "i500"

PULSE SEQUENCE
Relax. delay 3.000 sec
Pulse 6.0 usec
Acq. time 1.300 sec
Width 32000.0 Hz
704 repetitions
OBSERVE C13, 125.6055328 MHz
DECOUPLE H1, 499.5269647 MHz
Power 32 dB
continuously on
WALTZ-16 modulated
DATA PROCESSING
Line broadening 0.5 Hz
FT size 131072
Total time 50 minutes

INDEX	FREQUENCY	PPM	HEIGHT
1	18067.390	143.831	40.8
2	18054.207	143.726	43.0
3	17622.078	140.286	22.1
4	17110.847	136.216	21.0
5	16884.285	134.413	33.8
6	16752.937	133.367	31.7
7	16564.461	131.867	62.6
8	16290.535	129.686	25.1
9	16119.636	128.326	19.8
10	16070.808	127.937	64.8
11	14738.289	117.329	52.0
12	14464.363	115.148	27.1
13	9881.843	78.668	13.9
14	9704.597	77.257	103.2
15	9672.371	77.000	106.9
16	9640.633	76.747	103.6
17	8617.683	68.604	57.8
18	8075.691	64.289	61.7
19	8061.531	64.176	56.6
20	7402.840	58.933	78.4
21	5781.258	46.024	177.9
22	4756.355	37.864	40.8

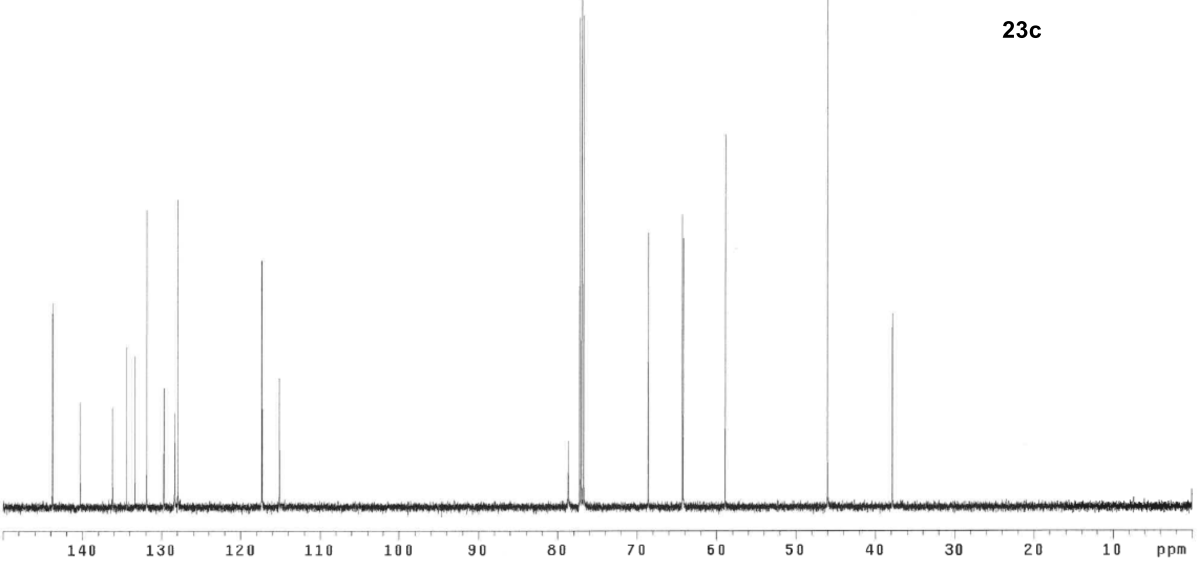

STANDARD 1H
63184
1957-BGE
Berecz Gabor
2006.04.26. (BT)

Solvent: CDCl3
Temp. 25.0 C / 298.1 K
INOVA-500 "i500"

PULSE SEQUENCE
Relax. delay 3.000 sec
Pulse 6.4 usec
Acq. time 5.000 sec
Width 8000.0 Hz
16 repetitions
OBSERVE H1, 499.5244656 MHz
DATA PROCESSING
FT size 131072
Total time 2 minutes

INDEX	FREQUENCY	PPM	HEIGHT
1	3943.237	7.894	29.7
2	3941.040	7.890	27.3
3	3831.177	7.670	20.6
4	3822.632	7.653	23.5
5	3717.285	7.442	18.9
6	3715.210	7.437	17.2
7	3708.862	7.425	16.5
8	3706.787	7.421	14.8
9	3631.470	7.270	5.9
10	3481.812	6.970	57.7
11	3474.609	6.956	48.7
12	2843.384	5.692	33.2
13	2108.276	4.221	140.2
14	2102.295	4.209	7.9
15	1857.544	3.719	4.4
16	1851.563	3.707	9.6
17	1847.778	3.699	9.7
18	1845.581	3.695	7.1
19	1841.797	3.687	16.6
20	1835.815	3.675	8.2
21	1827.881	3.659	8.3
22	1821.655	3.647	16.8
23	1818.115	3.640	7.0
24	1815.552	3.635	9.5
25	1812.012	3.627	9.3
26	1805.786	3.615	4.5
27	1698.242	3.400	172.8
28	1330.566	2.664	4.9
29	1323.608	2.650	12.3
30	1317.383	2.637	30.6
31	1311.401	2.625	30.1
32	1305.298	2.613	11.4
33	1298.218	2.599	4.8
34	1209.229	2.421	29.9
35	1019.409	2.041	3.5
36	793.945	1.589	11.7
37	788.330	1.578	30.5
38	782.715	1.567	44.2
39	777.100	1.556	34.9
40	771.606	1.545	13.3
41	717.163	1.436	14.4
42	711.914	1.425	15.9
43	0.000	0.000	5.8

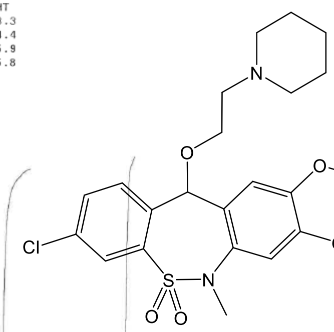

23d

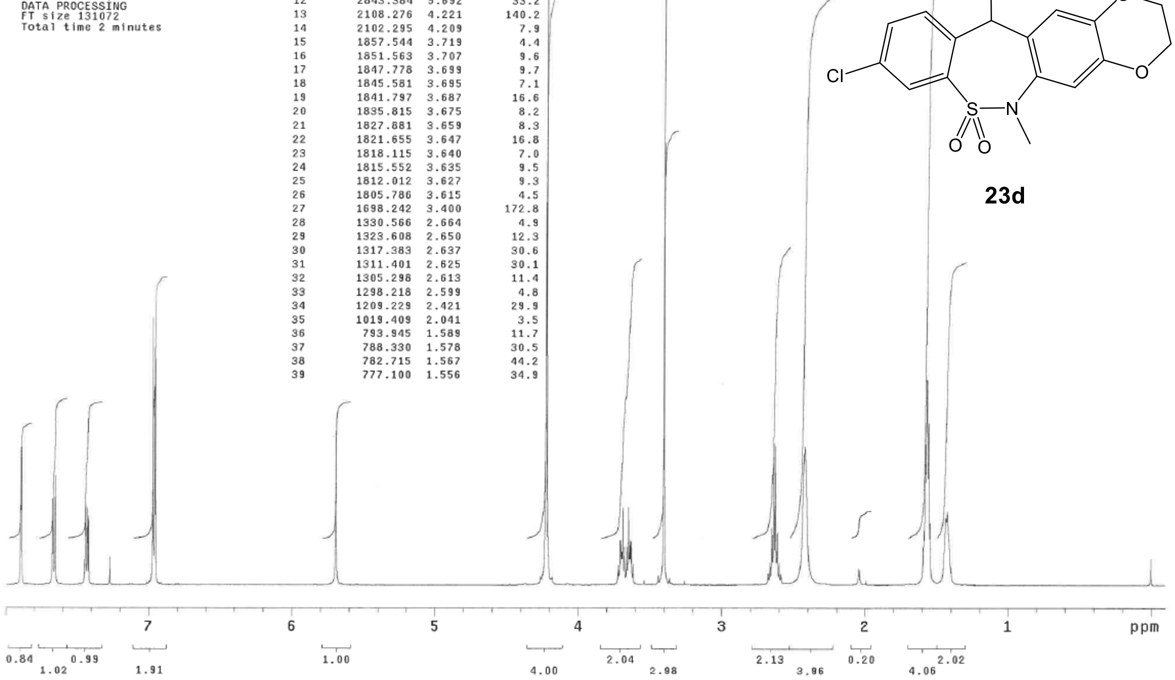

STANDARD 13C
63184
1957-BGE
Berecz Gabor
2006.04.26. (BT)

Solvent: CDCl3
Temp. 25.0 C / 298.1 K
User: 1-14-87
INOVA-500 "i500"

PULSE SEQUENCE
Relax. delay 3.000 sec
Pulse 6.0 usec
Acq. time 1.300 sec
Width 32000.0 Hz
160 repetitions
OBSERVE C13, 125.6055357 MHz
DECOUPLE H1, 499.5269647 MHz
Power 32 dB
continuously on
WALTZ-16 modulated
DATA PROCESSING
Line broadening 0.5 Hz
FT size 131072
Total time 11 minutes

INDEX	FREQUENCY	PPM	HEIGHT
1	18062.508	143.792	33.9
2	18046.883	143.668	36.5
3	17623.055	140.294	21.8
4	17114.754	136.247	22.2
5	16875.984	134.347	31.0
6	16742.195	133.282	28.6
7	16558.113	131.816	59.9
8	16296.883	129.737	24.3
9	16145.027	128.528	22.9
10	16061.043	127.859	61.1
11	14731.453	117.274	48.4
12	14482.430	115.292	29.7
13	9885.262	78.695	15.1
14	9704.109	77.253	71.4
15	9672.371	77.000	74.0
16	9640.144	76.743	72.8
17	8562.508	68.165	49.2
18	8071.785	64.258	56.5
19	8059.090	64.157	54.9
20	7351.082	58.521	62.3
21	6919.441	55.084	155.1
22	4769.051	37.966	38.5
23	3260.262	25.954	159.1
24	3034.187	24.155	72.7

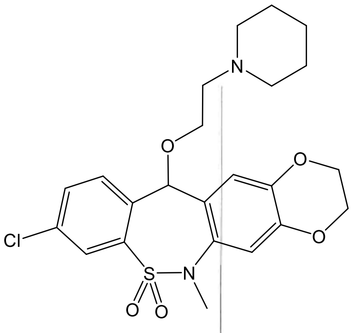

23d

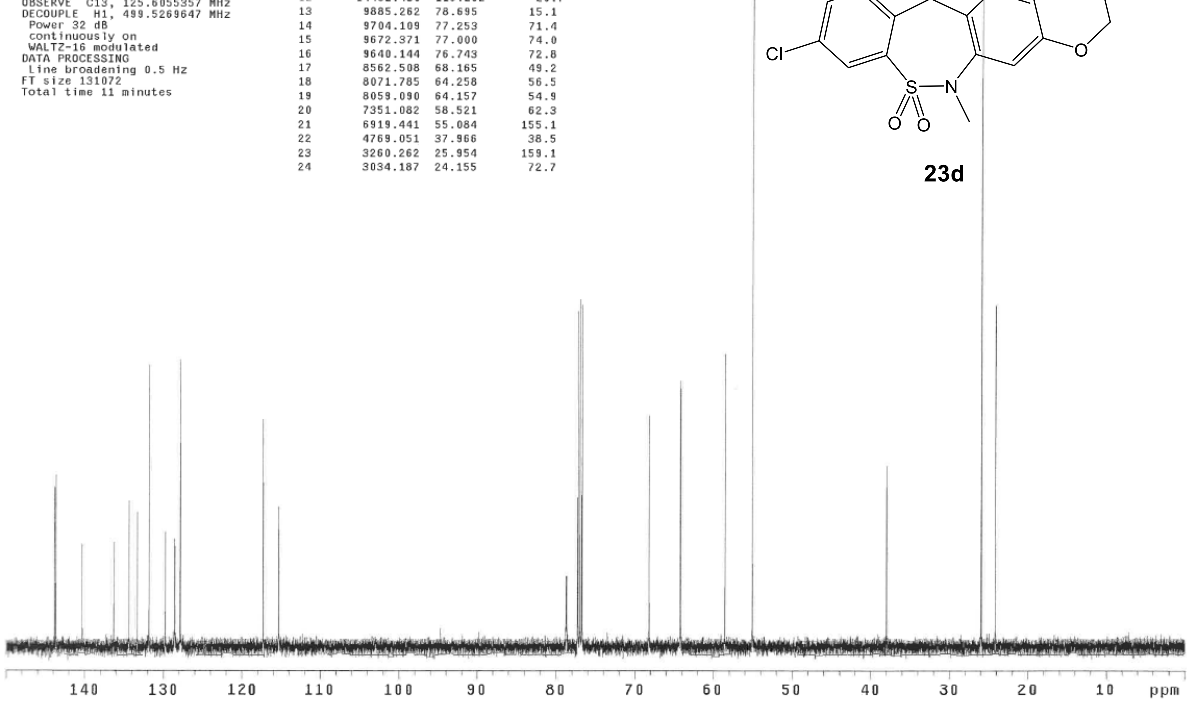

STANDARD 1H
62763
1949-BGE
Berecz Gabor
2006.03.29. (CzB)

Solvent: CDCl3
Temp. 25.0 C / 298.1 K
INOVA-500 "i500"

PULSE SEQUENCE
Relax. delay 1.000 sec
Pulse 6.4 usec
Acq. time 5.000 sec
Width 8000.0 Hz
16 repetitions
OBSERVE H1, 499.5244634 MHz
DATA PROCESSING
FT size 131072
Total time 1 minutes

INDEX	FREQUENCY	PPM	HEIGHT
1	3944.824	7.897	21.2
2	3942.749	7.893	22.1
3	3827.515	7.662	16.2
4	3819.092	7.645	18.7
5	3720.337	7.448	14.2
6	3718.262	7.444	14.2
7	3711.914	7.431	12.3
8	3709.717	7.426	12.3
9	3633.301	7.274	5.7
10	3487.793	6.982	39.0
11	3482.910	6.972	48.7
12	2848.633	5.703	25.5
13	2116.089	4.236	5.1
14	2109.863	4.224	112.5
15	2103.760	4.212	5.0
16	1865.723	3.735	3.8
17	1859.985	3.724	9.5
18	1856.201	3.716	39.4
19	1851.440	3.706	47.2
20	1847.046	3.698	35.6
21	1835.083	3.674	6.7
22	1829.346	3.662	13.4
23	1825.073	3.654	5.5
24	1823.608	3.651	7.6
25	1819.458	3.642	7.3
26	1813.721	3.631	3.6
27	1696.777	3.397	151.5
28	1342.041	2.687	19.2
29	1336.304	2.675	37.5
30	1330.566	2.664	17.7
31	1253.662	2.510	24.8
32	1249.268	2.501	32.9
33	1244.873	2.492	23.6
34	995.605	1.993	9.1
35	946.411	1.895	2.9
36	-0.000	-0.000	6.4

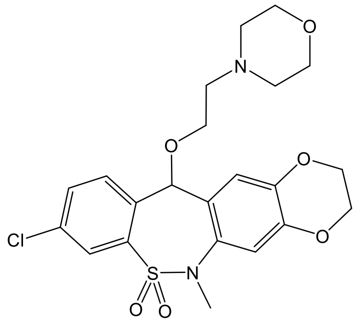

23e

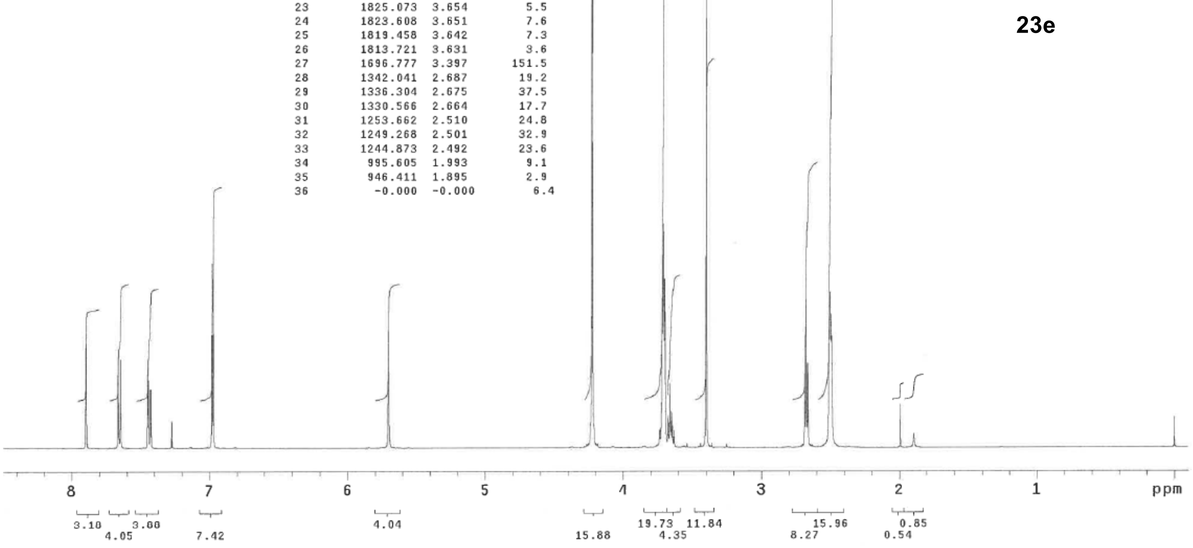

STANDARD 13C
62763
1949-BGE
Berecz Gabor
2006.03.29. (CzB)

Solvent: CDCl3
Temp. 25.0 C / 298.1 K
User: 1-14-87
INOVA-500 "i500"

PULSE SEQUENCE
Relax. delay 3.000 sec
Pulse 6.0 usec
Acq. time 1.300 sec
Width 32000.0 Hz
368 repetitions
OBSERVE C13, 125.6055367 MHz
DECOUPLE H1, 499.5269647 MHz
Power 32 dB
continuously on
WALTZ-16 modulated
DATA PROCESSING
Line broadening 0.5 Hz
FT size 131072
Total time 26 minutes

INDEX	FREQUENCY	PPM	HEIGHT
1	18065.926	143.820	40.1
2	18045.418	143.656	48.6
3	17608.406	140.177	23.8
4	17102.059	136.146	23.9
5	16880.867	134.386	39.6
6	16712.898	133.048	37.9
7	16562.020	131.847	70.5
8	16296.395	129.733	27.8
9	16110.359	128.252	23.0
10	16065.926	127.898	68.4
11	14721.199	117.193	52.7
12	14459.969	115.113	31.8
13	9866.707	78.547	15.7
14	9704.598	77.257	74.9
15	9672.371	77.000	79.1
16	9640.633	76.747	75.7
17	8509.285	67.741	54.7
18	8398.445	66.859	145.4
19	8071.297	64.254	59.4
20	8058.602	64.153	60.5
21	7308.113	58.179	73.3
22	6791.023	54.062	168.9
23	4761.727	37.907	44.3

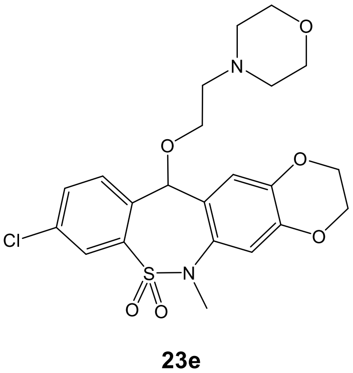

23e

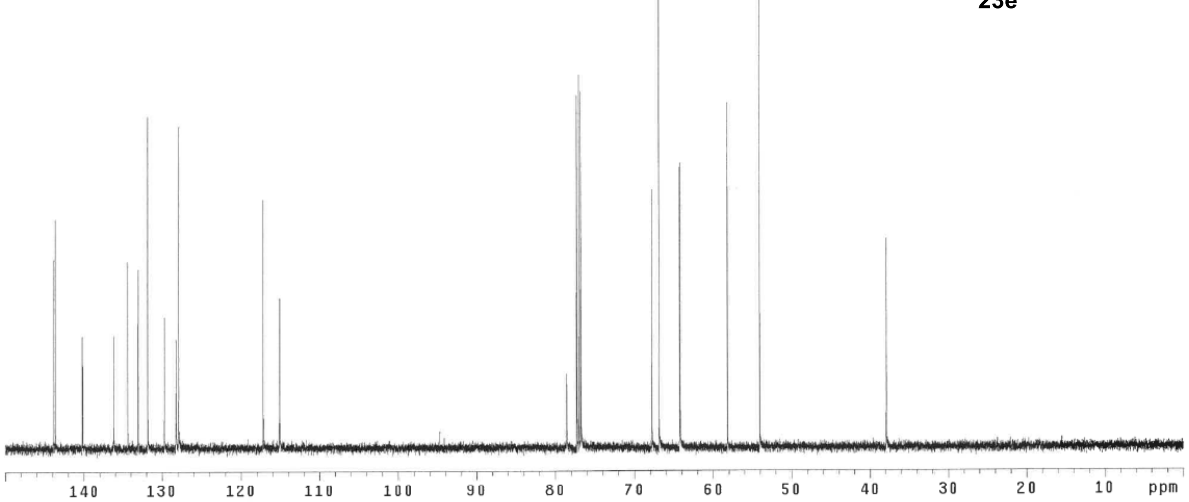

STANDARD 1H
63256
1960-BGE
Berecz Gabor
2006.05.03. (CzB)

Solvent: CDCl3
Temp. 25.0 C / 298.1 K
INOVA-500 "i500"

PULSE SEQUENCE
Relax. delay 1.000 sec
Pulse 6.4 usec
Acq. time 5.000 sec
Width 8000.0 Hz
16 repetitions
OBSERVE H1, 499.5244696 MHz
DATA PROCESSING
FT size 131072
Total time 1 minutes

INDEX	FREQUENCY	PPM	HEIGHT	INDEX	FREQUENCY	PPM	HEIGHT
1	3964.844	7.937	20.8	40	1230.957	2.464	5.9
2	3962.646	7.933	20.6	41	1225.952	2.454	7.1
3	3703.979	7.415	12.7	42	1223.145	2.449	8.5
4	3701.782	7.411	12.3	43	1220.947	2.444	6.7
5	3695.679	7.398	18.2	44	1217.896	2.438	7.5
6	3693.481	7.394	18.0	45	1211.792	2.426	6.6
7	3655.273	7.318	25.3	46	1206.543	2.415	13.3
8	3646.973	7.301	18.2	47	1202.759	2.408	24.7
9	3628.174	7.263	23.5	48	1198.120	2.399	24.6
10	3492.554	6.992	48.4	49	1194.092	2.390	11.4
11	3373.169	6.753	48.7	50	1187.012	2.376	3.1
12	2470.215	4.945	30.9	51	1000.488	2.003	2.1
13	2133.911	4.272	3.4	52	816.895	1.635	4.5
14	2130.371	4.265	7.2	53	-0.000	-0.000	23.7
15	2128.418	4.261	10.8				
16	2126.587	4.257	26.0				
17	2124.512	4.253	88.4				
18	2119.263	4.243	8.4				
19	2115.112	4.234	3.5				
20	2112.915	4.230	1.9				
21	1845.703	3.695	32.9				
22	1841.064	3.686	52.1				
23	1836.304	3.676	32.4				
24	1753.540	3.510	168.5				
25	1312.378	2.627	3.3				
26	1307.495	2.617	6.5				
27	1301.270	2.605	6.3				
28	1298.950	2.600	8.2				
29	1296.875	2.596	6.3				
30	1292.358	2.587	8.3				
31	1284.546	2.572	5.7				
32	1264.404	2.531	3.9				
33	1259.033	2.520	4.3				
34	1256.470	2.515	8.2				
35	1250.977	2.504	18.7				
36	1245.972	2.494	6.6				
37	1243.530	2.489	17.3				
38	1238.892	2.480	11.5				
39	1235.718	2.474	15.0				

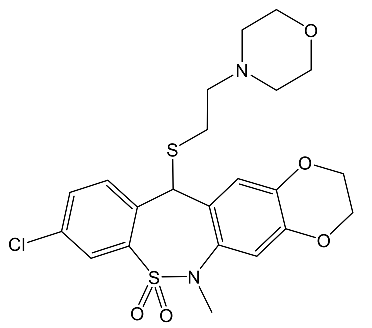

24

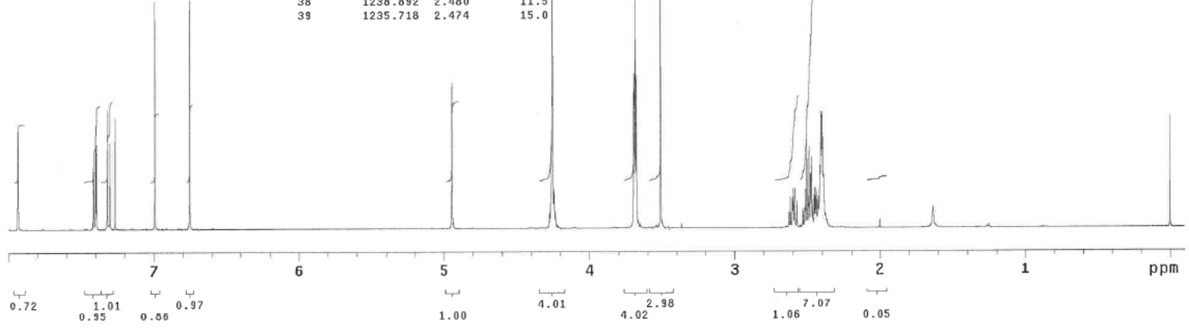

STANDARD 13C
63256
1960-BGE
Berecz Gabor
2006.05.03. (CzB)

Solvent: CDCl3
Temp. 25.0 C / 298.1 K
User: 1-14-87
INOVA-500 "i500"

PULSE SEQUENCE
Relax. delay 3.000 sec
Pulse 6.0 usec
Acq. time 1.300 sec
Width 32000.0 Hz
14336 repetitions
OBSERVE C13, 125.6055293 MHz
DECOUPLE H1, 499.5269647 MHz
Power 32 dB
continuously on
WALTZ-16 modulated
DATA PROCESSING
Line broadening 0.5 Hz
FT size 131072
Total time 17.1 hours

INDEX	FREQUENCY	PPM	HEIGHT
1	18128.425	144.317	29.4
2	17968.757	143.046	25.2
3	17875.984	142.307	21.2
4	16911.628	134.630	26.1
5	16901.863	134.553	23.6
6	16644.050	132.500	29.6
7	16566.902	131.886	22.7
8	16546.394	131.723	43.0
9	16511.238	131.443	21.3
10	16052.742	127.793	40.6
11	14835.456	118.102	40.4
12	14810.554	117.904	44.2
13	9704.597	77.257	171.4
14	9672.371	77.000	178.2
15	9640.632	76.747	174.4
16	8393.074	66.816	90.0
17	8080.574	64.328	37.7
18	8071.785	64.258	41.5
19	7313.484	58.221	56.6
20	6825.691	54.338	20.8
21	6725.593	53.541	113.2
22	4942.390	39.345	37.0
23	3803.230	30.277	46.5

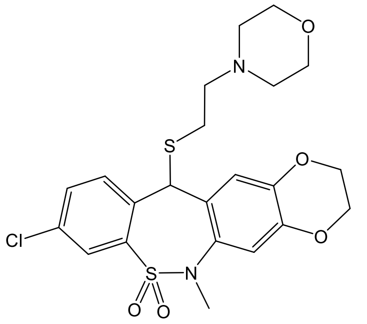

24

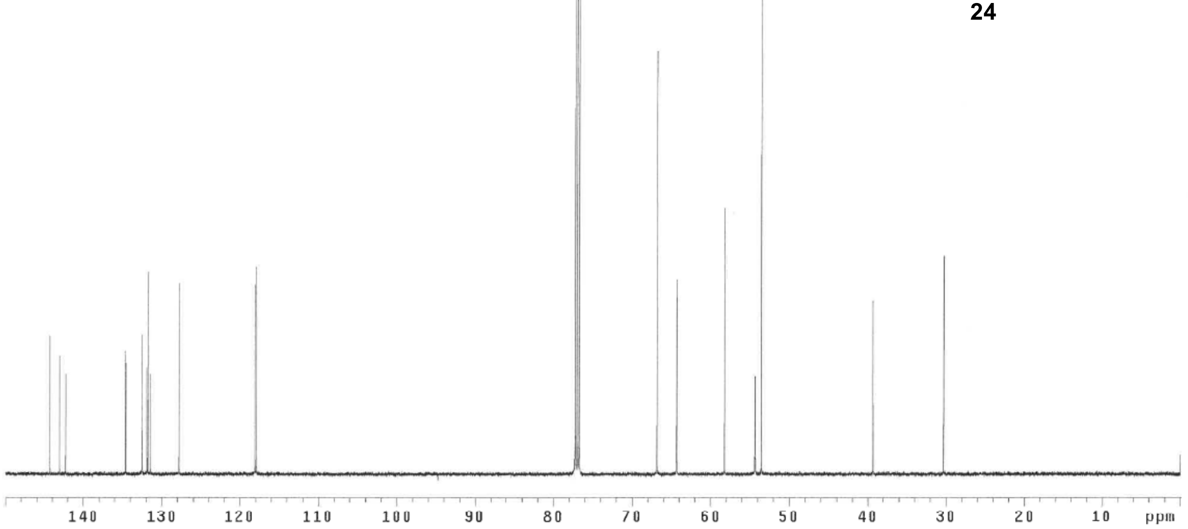

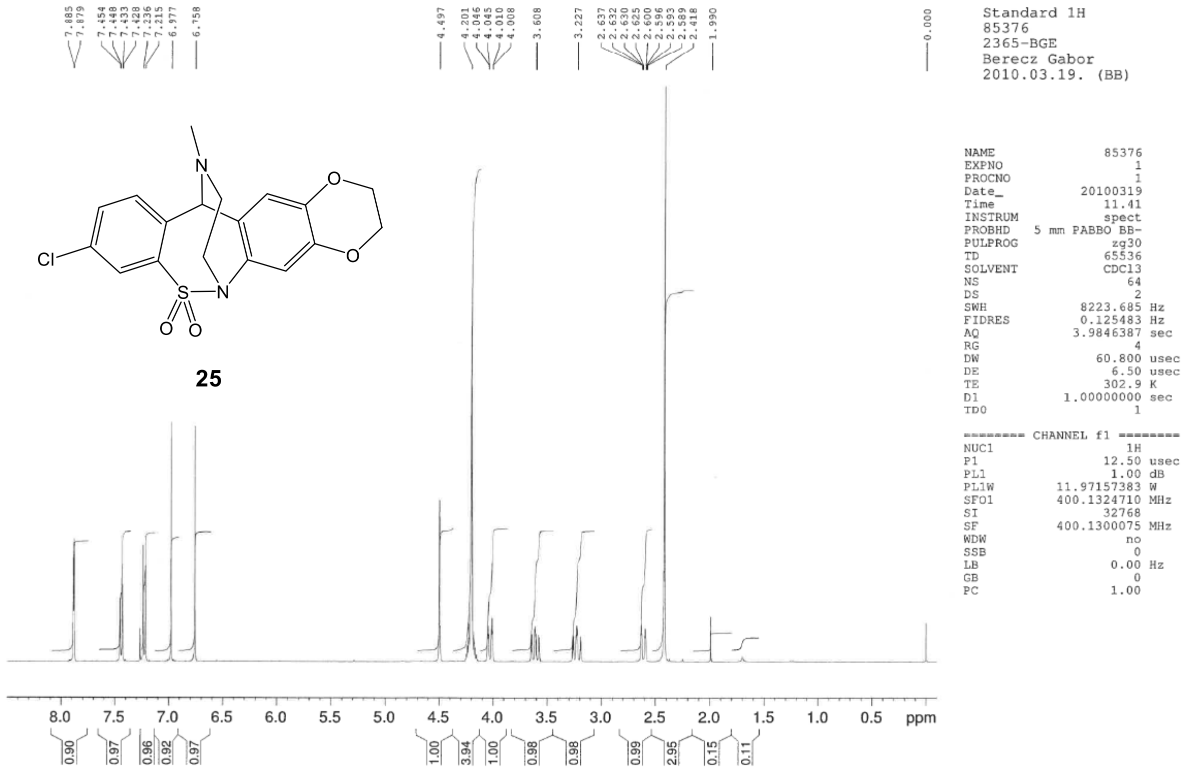
7.885
7.879
7.454
7.448
7.433
7.428
7.236
7.215
6.977
6.758
4.497
4.201
4.046
4.045
4.010
4.008
3.608
3.227
2.637
2.632
2.630
2.625
2.600
2.596
2.593
2.589
2.418
1.990
0.000
Standard 1H
85376
2365-BGE
Berecz Gabor
2010.03.19. (BB)
NAME 85376
EXPNO 1
PROCNO 1
Date_ 20100319
Time 11.41
INSTRUM spect
PROBHD 5 mm PABBO BB-
PULPROG zg30
TD 65536
SOLVENT CDCl3
NS 64
DS 2
SWH 8223.685 Hz
FIDRES 0.125483 Hz
AQ 3.9846387 sec
RG 4
DW 60.800 usec
DE 6.50 usec
TE 302.9 K
D1 1.00000000 sec
TD0 1
======== CHANNEL f1 ========
NUC1 1H
P1 12.50 usec
PL1 1.00 dB
PL1W 11.97157383 W
SFO1 400.1324710 MHz
SI 32768
SF 400.1300075 MHz
WDW no
SSB 0
LB 0.00 Hz
GB 0
PC 1.00
Cl
N
O
O
S
N
O
O
25
0.90
0.97
0.96
0.92
0.97
1.00
3.94
1.00
0.98
0.98
0.99
2.95
0.15
0.11
8.0 7.5 7.0 6.5 6.0 5.5 5.0 4.5 4.0 3.5 3.0 2.5 2.0 1.5 1.0 0.5 ppm

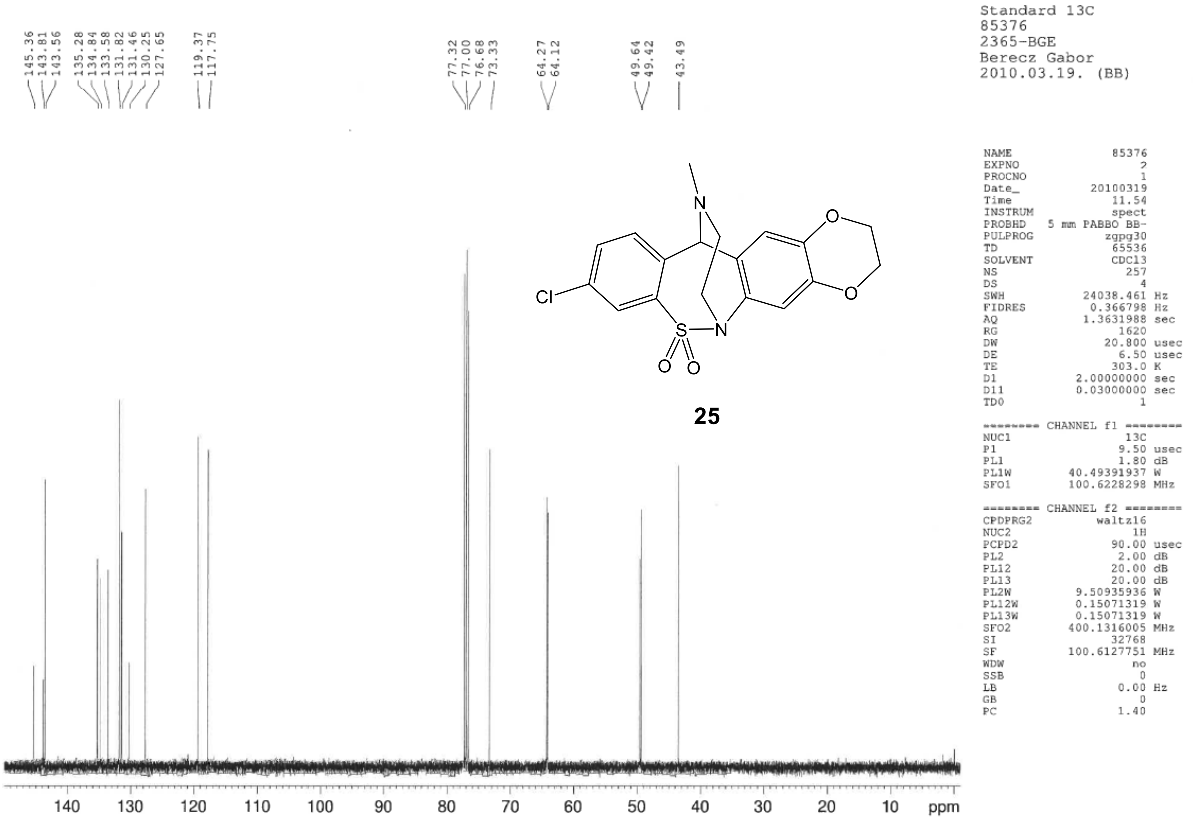
145.36
143.81
143.56
135.28
134.84
133.58
131.82
131.46
130.25
127.65
119.37
117.75
77.32
77.00
76.68
73.33
64.27
64.12
49.64
49.42
43.49
Standard 13C
85376
2365-BGE
Berecz Gabor
2010.03.19. (BB)
NAME 85376
EXPNO 2
PROCNO 1
Date_ 20100319
Time 11.54
INSTRUM spect
PROBHD 5 mm PABBO BB-
PULPROG zgpg30
TD 65536
SOLVENT CDCl3
NS 257
DS 4
SWH 24038.461 Hz
FIDRES 0.366798 Hz
AQ 1.3631988 sec
RG 1620
DW 20.800 usec
DE 6.50 usec
TE 303.0 K
D1 2.00000000 sec
D11 0.03000000 sec
TD0 1
======== CHANNEL f1 ========
NUC1 13C
P1 9.50 usec
PL1 1.80 dB
PL1W 40.49391937 W
SFO1 100.6228298 MHz
======== CHANNEL f2 ========
CPDPRG2 waltz16
NUC2 1H
PCPD2 90.00 usec
PL2 2.00 dB
PL12 20.00 dB
PL13 20.00 dB
PL2W 9.50935936 W
PL12W 0.15071319 W
PL13W 0.15071319 W
SFO2 400.1316005 MHz
SI 32768
SF 100.6127751 MHz
WDW no
SSB 0
LB 0.00 Hz
GB 0
PC 1.40
Cl
N
O
O
S
N
O
O
25
140 130 120 110 100 90 80 70 60 50 40 30 20 10 ppm

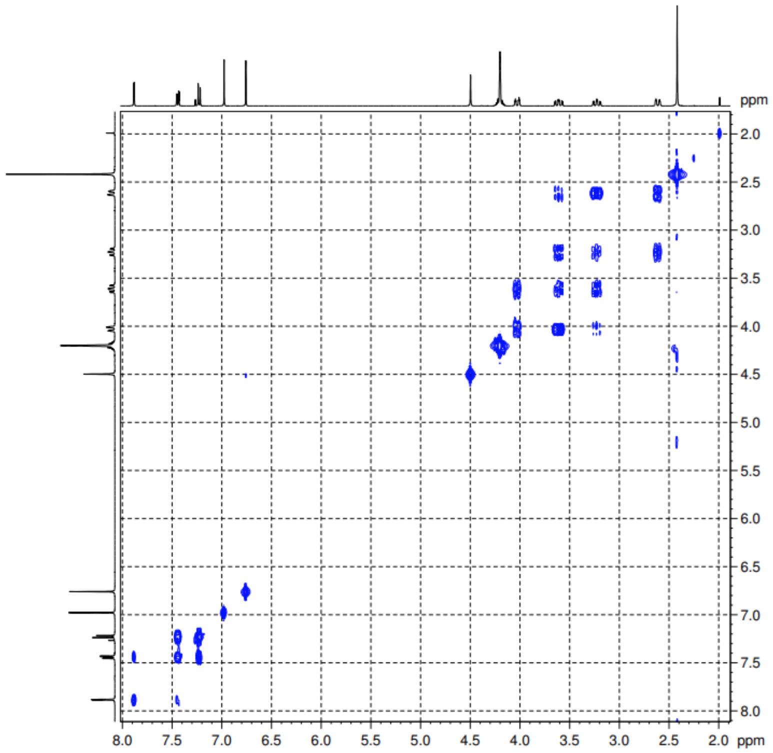

COSY
85376
2365-BGE
Berecz Gabor
2010.03.19. (BB)

25

```
NAME              85376
EXPNO                 3
PROCNO                1
Date_          20100319
Time              12.00
INSTRUM           spect
PROBHD   5 mm PABBO BB-
PULPROG        cosygpqf
TD                 2048
SOLVENT           CDCl3
NS                    1
DS                    8
SWH            3401.361 Hz
FIDRES         1.660821 Hz
AQ            0.3011060 sec
RG                   36
DW              147.000 usec
DE                 6.50 usec
TE                303.0 K
D0           0.00000300 sec
D1           1.00000000 sec
D13          0.00000400 sec
D16          0.00020000 sec
IN0          0.00029400 sec

======== CHANNEL f1 ========
NUC1                 1H
P0                12.50 usec
P1                12.50 usec
PL1                1.00 dB
PL1W        11.97157383 W
SFO1        400.1317006 MHz

====== GRADIENT CHANNEL =====
GPNAM1         SINE.100
GPZ1              10.00 %
P16             1000.00 usec
ND0                   1
TD                  128
SFO1           400.1317 MHz
FIDRES        26.571245 Hz
SW                8.500 ppm
FnMODE               QF
SI                 1024
SF          400.1300059 MHz
WDW                SINE
SSB                   0
LB                 0.00 Hz
GB                    0
PC                 1.40
SI                 1024
MC2                  QF
SF          400.1300051 MHz
WDW               QSINE
SSB                   0
LB                 0.00 Hz
GB                    0
```

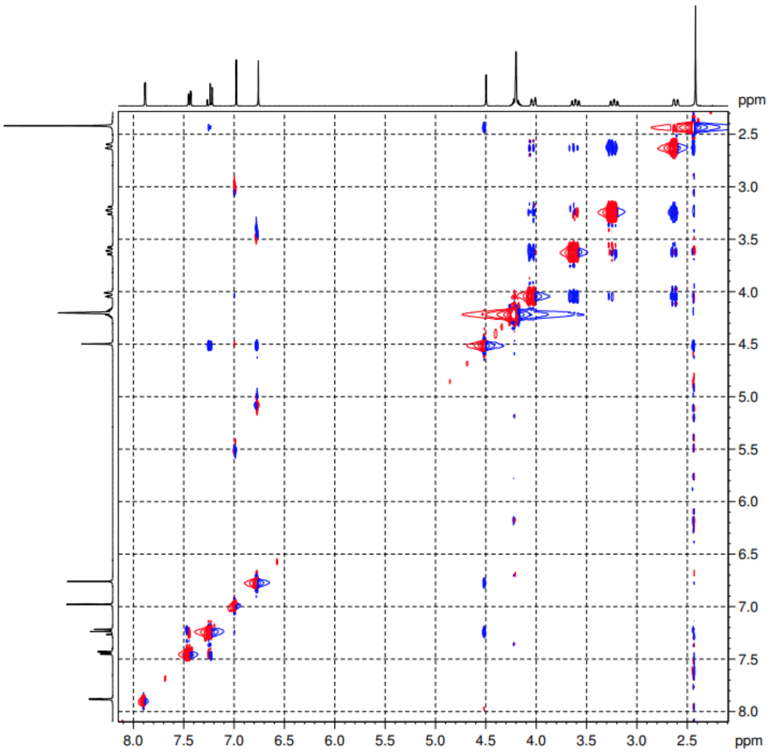

NOESY
85376
2365-BGE
Berecz Gabor
2010.03.19. (BB)

25

```
NAME              85376
EXPNO                 6
PROCNO                1
Date_          20100319
Time              13.43
INSTRUM           spect
PROBHD   5 mm PABBO BB-
PULPROG         noesyph
TD                 2048
SOLVENT           CDCl3
NS                    4
DS                    4
SWH            3401.361 Hz
FIDRES         1.660821 Hz
AQ            0.3011060 sec
RG                   64
DW              147.000 usec
DE                 6.50 usec
TE                303.0 K
D0           0.00013110 sec
D1           1.00000000 sec
D8           0.30000001 sec
IN0          0.00029400 sec

-------- CHANNEL f1 --------
NUC1                 1H
P1                12.50 usec
PL1                1.00 dB
PL1W        11.97157383 W
SFO1        400.1317006 MHz
ND0                   1
TD                  142
SFO1           400.1317 MHz
FIDRES        23.951546 Hz
SW                8.500 ppm
FnMODE      States-TPPI
SI                 1024
SF          400.1300000 MHz
WDW               QSINE
SSB                   2
LB                 0.00 Hz
GB                    0
PC                 1.00
SI                 1024
MC2         States-TPPI
SF          400.1300000 MHz
WDW               QSINE
SSB                   2
LB                 0.00 Hz
GB                    0
```

25

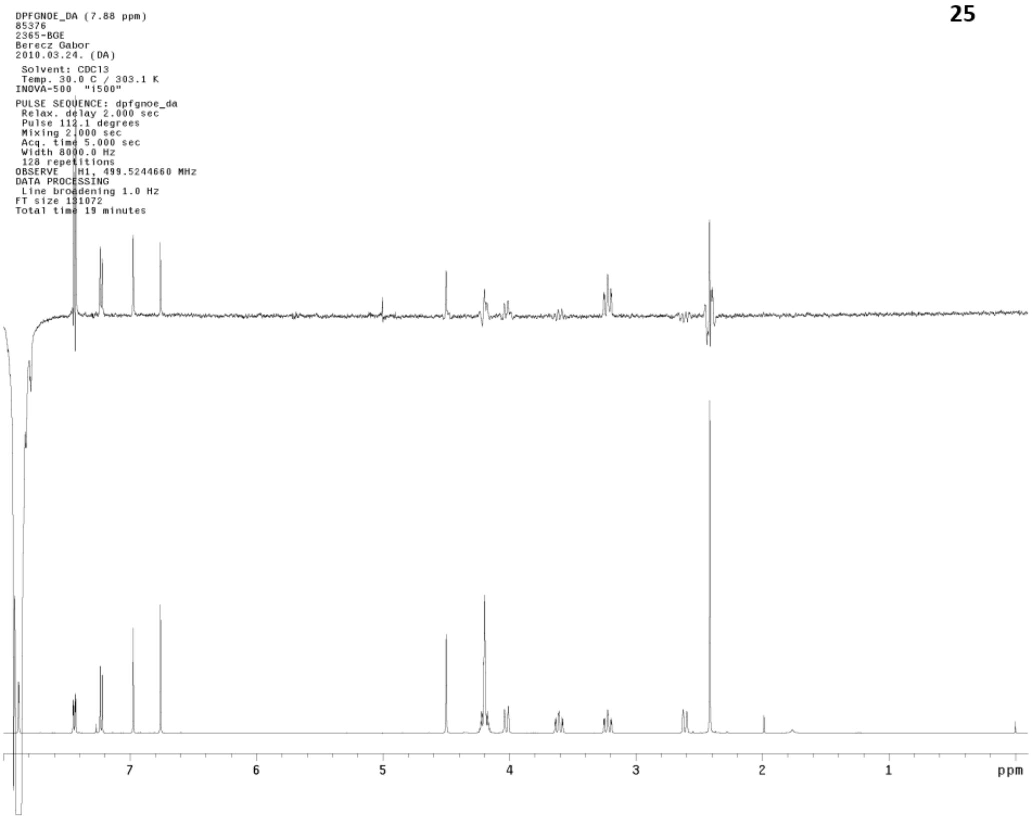
DPFGNOE_DA (7.88 ppm)
85376
2365-BGE
Berecz Gabor
2010.03.24. (DA)
Solvent: CDCl3
Temp. 30.0 C / 303.1 K
INOVA-500 "i500"
PULSE SEQUENCE: dpfgnoe_da
Relax. delay 2.000 sec
Pulse 112.1 degrees
Mixing 2.000 sec
Acq. time 5.000 sec
Width 8000.0 Hz
128 repetitions
OBSERVE H1, 499.5244660 MHz
DATA PROCESSING
Line broadening 1.0 Hz
FT size 131072
Total time 19 minutes
7
6
5
4
3
2
1
ppm

25

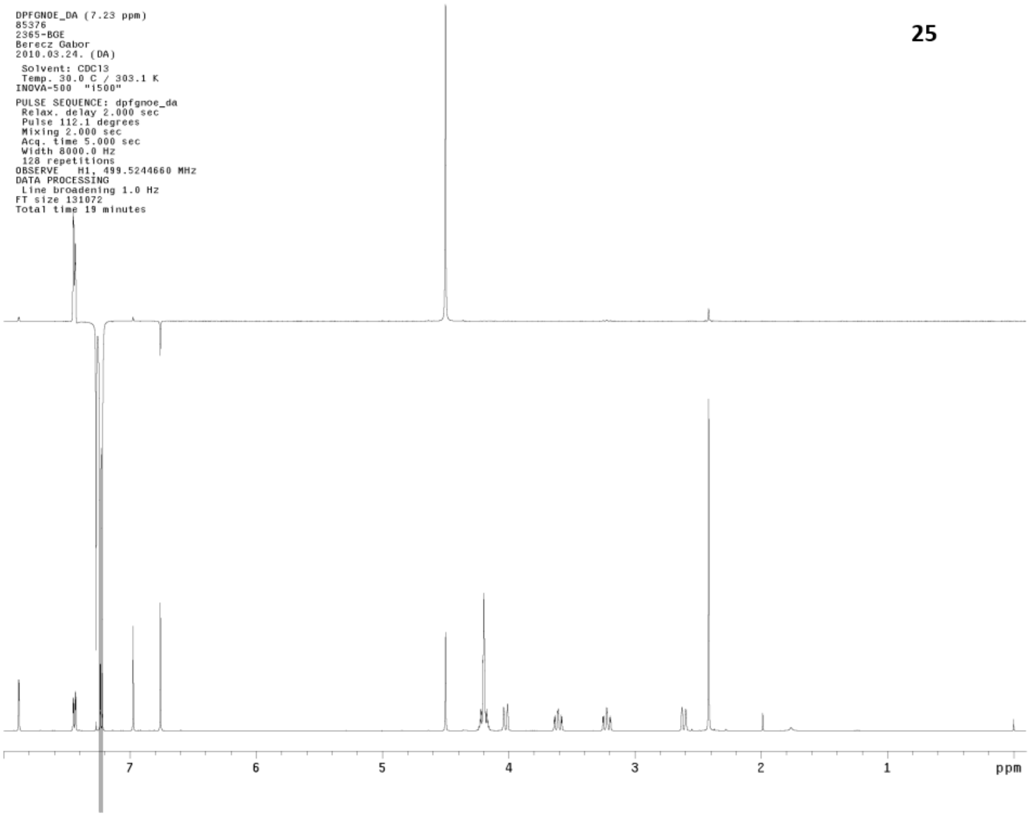
DPFGNOE_DA (7.23 ppm)
85376
2365-BGE
Berecz Gabor
2010.03.24. (DA)
Solvent: CDCl3
Temp. 30.0 C / 303.1 K
INOVA-500 "i500"
PULSE SEQUENCE: dpfgnoe_da
Relax. delay 2.000 sec
Pulse 112.1 degrees
Mixing 2.000 sec
Acq. time 5.000 sec
Width 8000.0 Hz
128 repetitions
OBSERVE H1, 499.5244660 MHz
DATA PROCESSING
Line broadening 1.0 Hz
FT size 131072
Total time 19 minutes
7
6
5
4
3
2
1
ppm

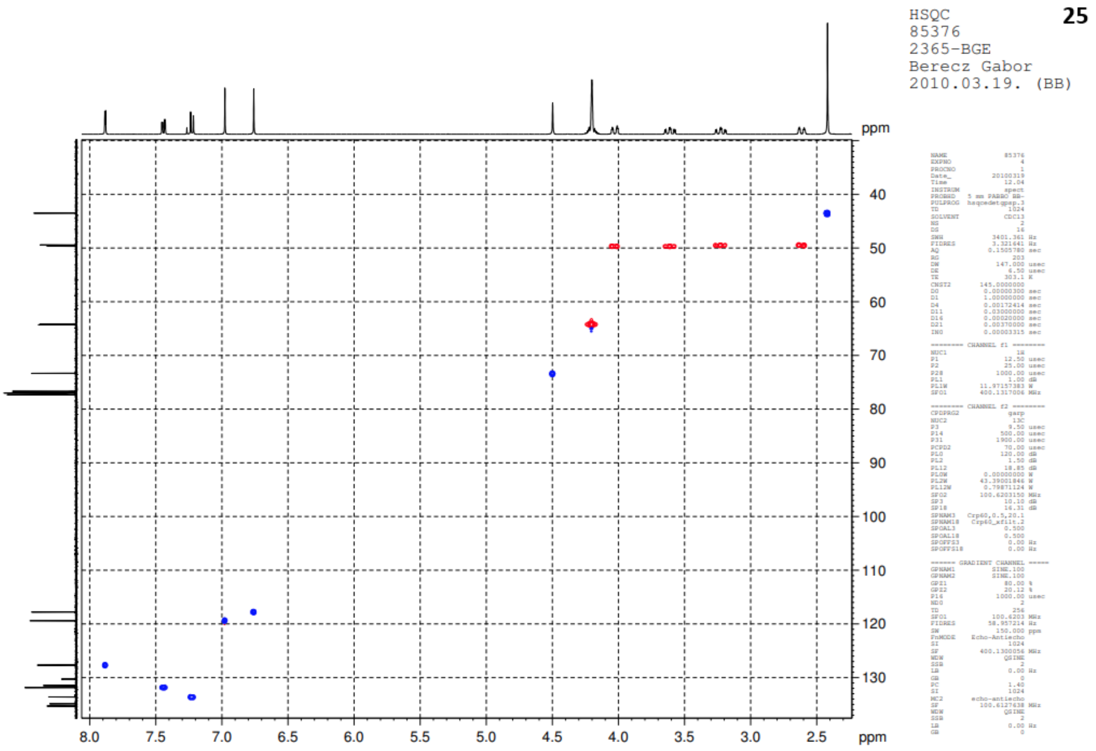
HSQC
85376
2365-BGE
Berecz Gabor
2010.03.19. (BB)
25
ppm

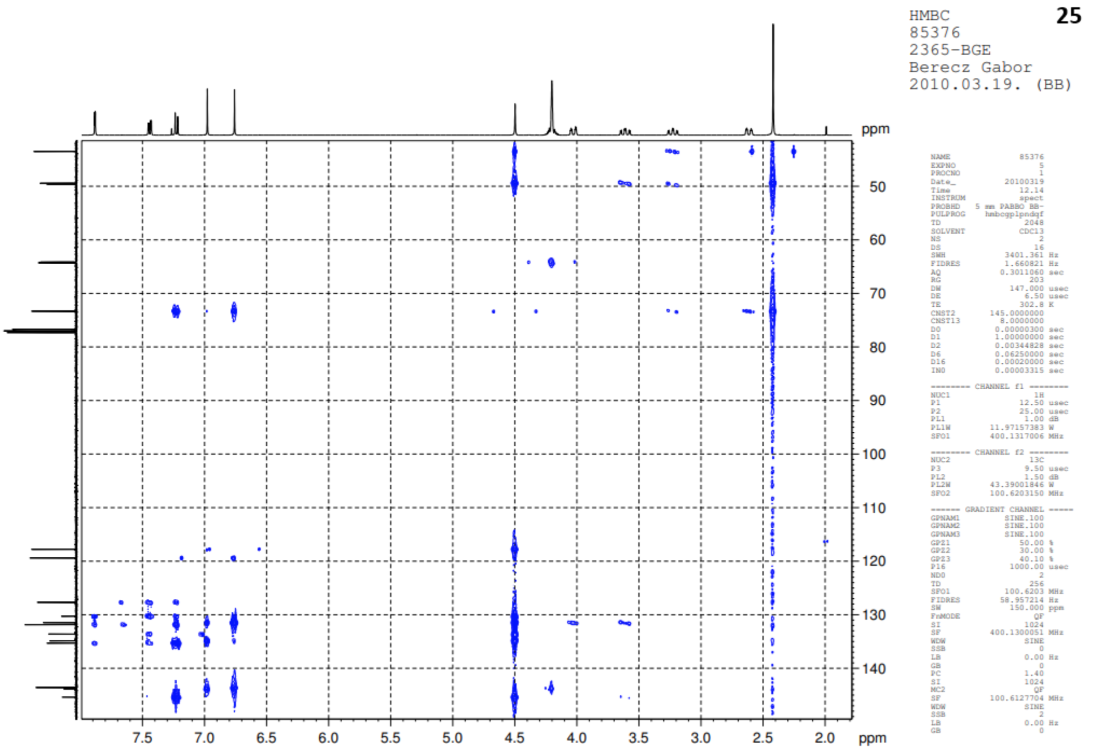
HMBC
85376
2365-BGE
Berecz Gabor
2010.03.19. (BB)
25
ppm

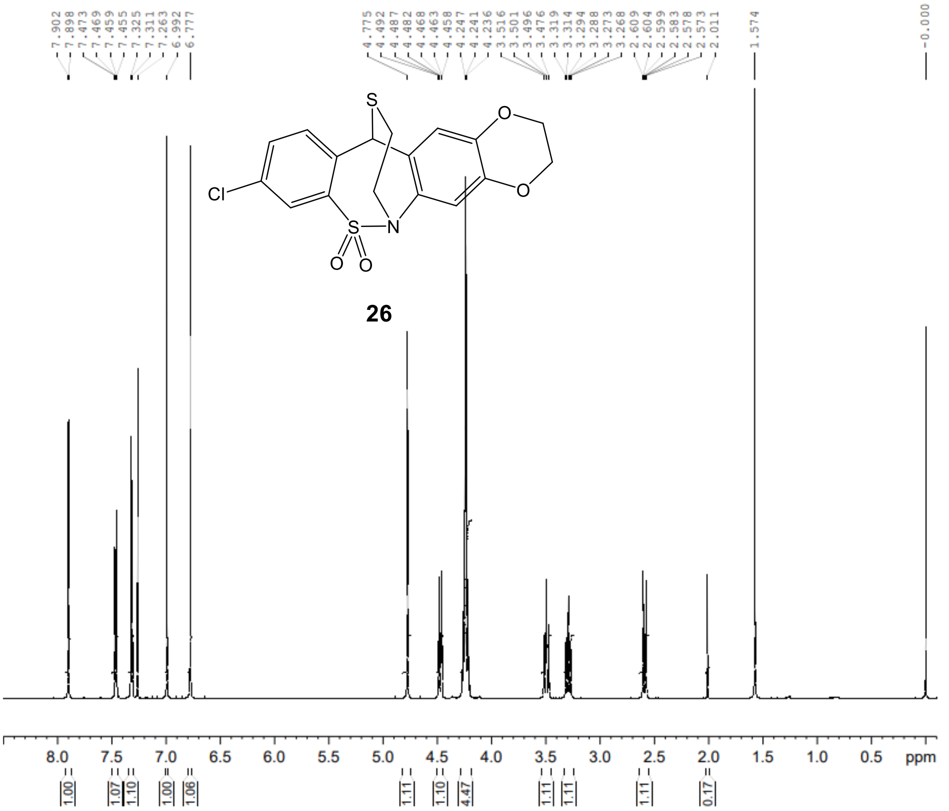

Standard 1H
143465
BEG0701_1B
Berecz Gabor
2024.09.06. (KP)

Current Data Parameters
NAME 143465
EXPNO 11
PROCNO 1

F2 - Acquisition Parameters
Date_ 20240906
Time 15.41 h
INSTRUM spect
PROBHD Z145856_0002 (
PULPROG zg30
TD 65536
SOLVENT CDCl3
NS 16
DS 2
SWH 12019.230 Hz
FIDRES 0.366798 Hz
AQ 2.7262976 sec
RG 196.07
DW 41.600 usec
DE 25.00 usec
TE 295.0 K
D1 1.00000000 sec
TD0 1
SFO1 600.0037050 MHz
NUC1 1H
P1 11.50 usec
PLW1 28.00000000 W

F2 - Processing parameters
SI 65536
SF 600.0000132 MHz
WDW EM
SSB 0
LB 0.30 Hz
GB 0
PC 1.00

BRUKER

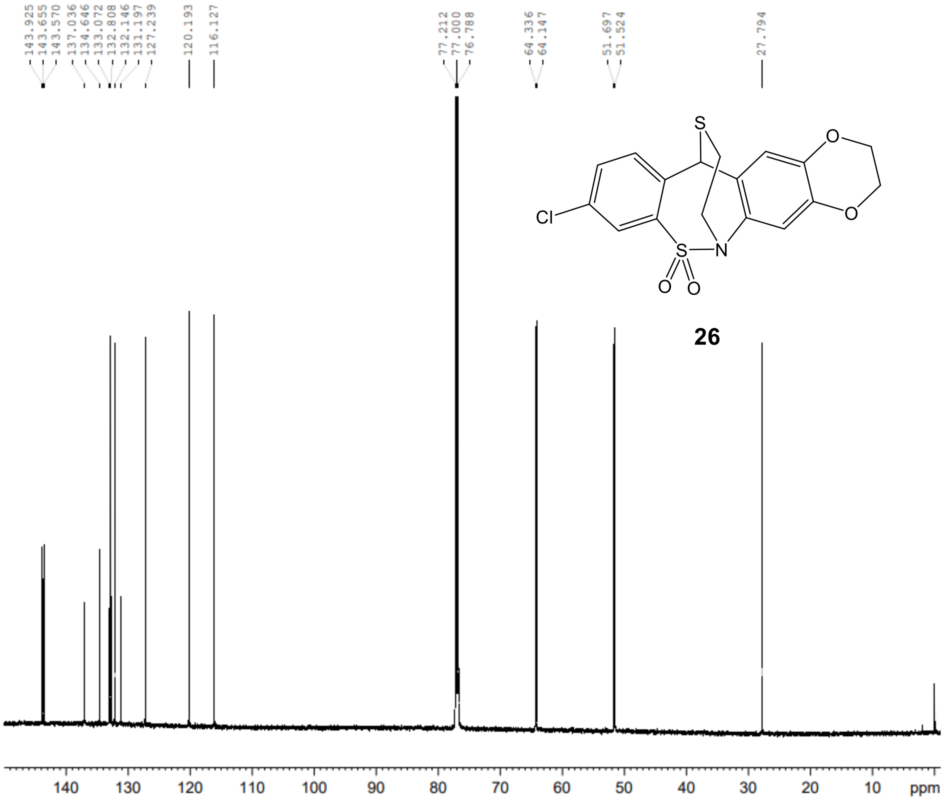

Standard 13C
143465
BEG0701_1B
Berecz Gabor
2024.09.06. (KP)

Current Data Parameters
NAME 143465
EXPNO 12
PROCNO 1

F2 - Acquisition Parameters
Date_ 20240906
Time 16.50 h
INSTRUM spect
PROBHD Z145856_0002 (
PULPROG zgpg30
TD 65536
SOLVENT CDCl3
NS 2048
DS 4
SWH 36231.883 Hz
FIDRES 1.105709 Hz
AQ 0.9043968 sec
RG 196.07
DW 13.800 usec
DE 18.00 usec
TE 295.0 K
D1 1.00000000 sec
D11 0.03000000 sec
TD0 1
SFO1 150.8852070 MHz
NUC1 13C
P1 9.90 usec
PLW1 71.00000000 W
SFO2 600.0024000 MHz
NUC2 1H
CPDPRG[2 waltz16
PCPD2 80.00 usec
PLW2 32.90000153 W
PLW12 0.70370001 W
PLW13 0.35339001 W

F2 - Processing parameters
SI 131072
SF 150.8701274 MHz
WDW EM
SSB 0
LB 1.00 Hz
GB 0
PC 1.40

BRUKER

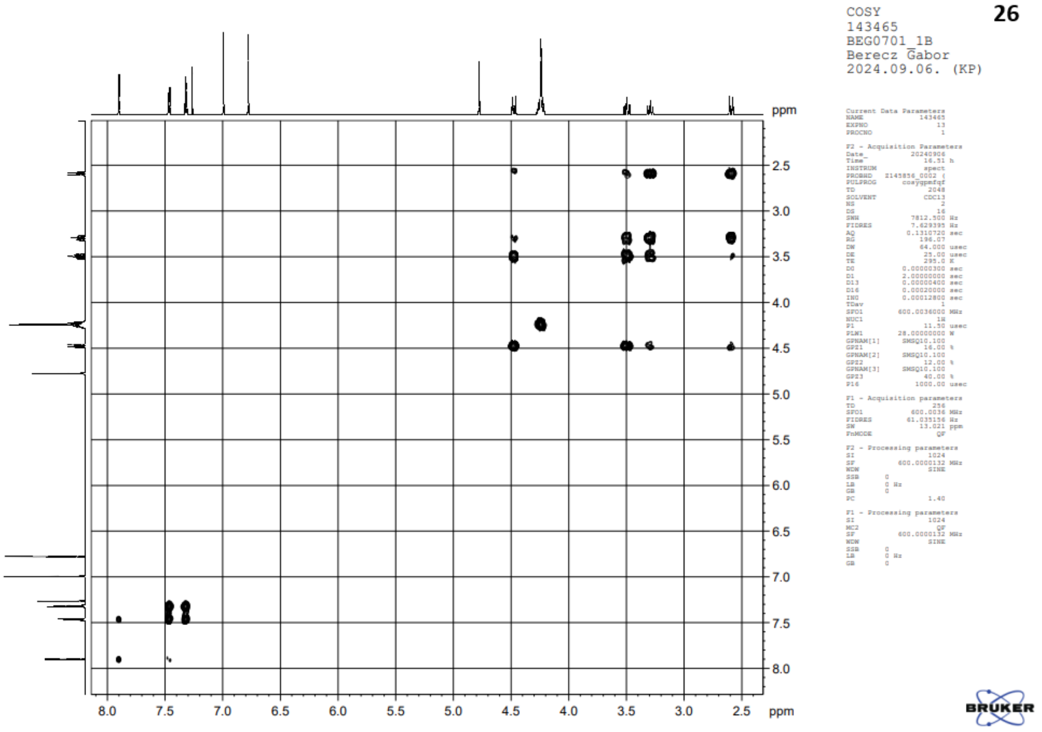
COSY
143465
BEG0701_1B
Berecz Gabor
2024.09.06. (KP)
ppm
BRUKER

26

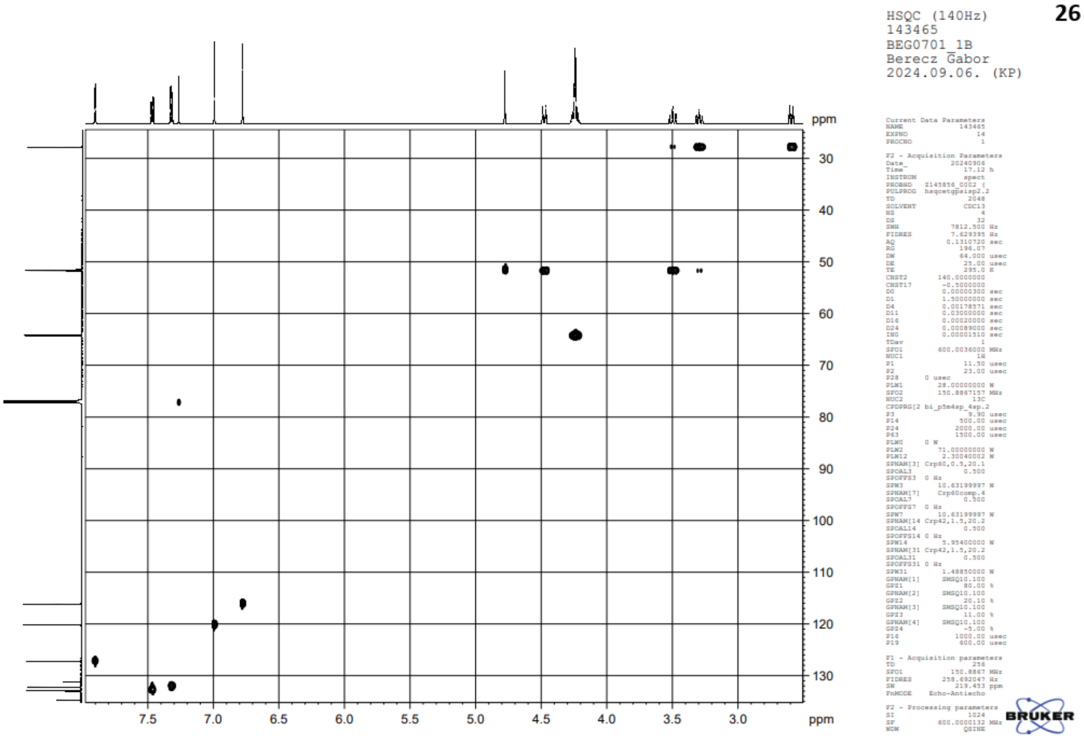
HSQC (140Hz)
143465
BEG0701_1B
Berecz Gabor
2024.09.06. (KP)
ppm
BRUKER

26

26

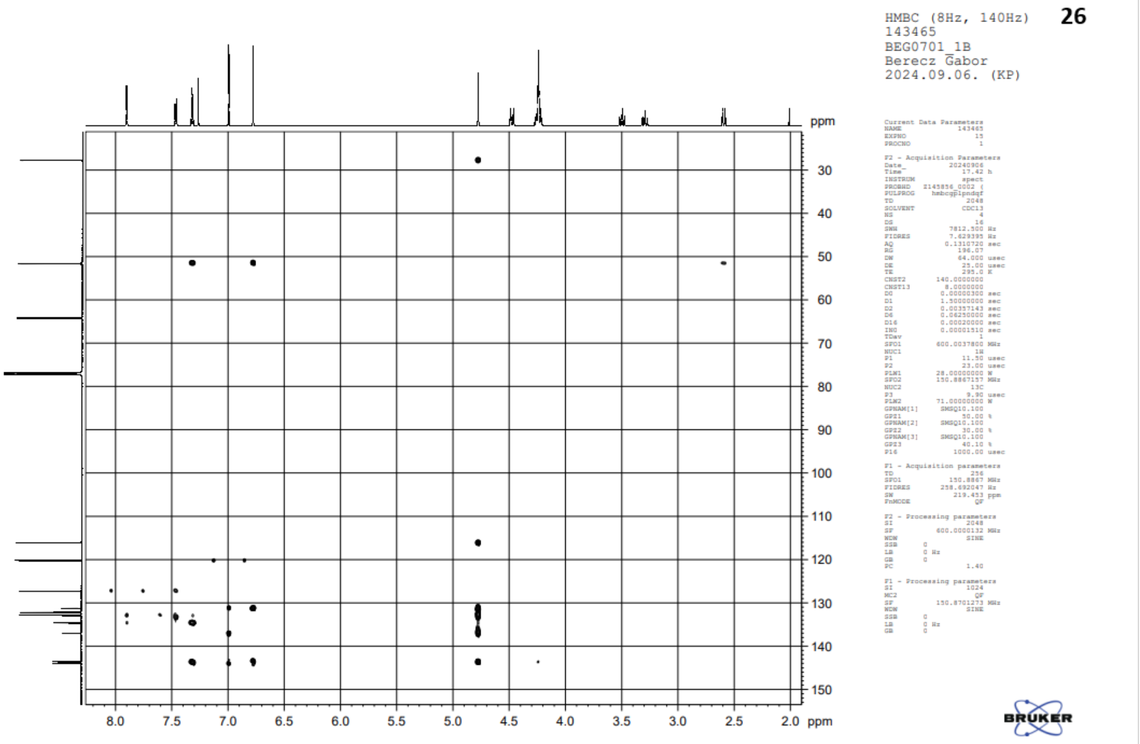

HMBC (8Hz, 140Hz)
143465
BEG0701_1B
Berecz Gabor
2024.09.06. (KP)
ppm
30
40
50
60
70
80
90
100
110
120
130
140
150
8.0
7.5
7.0
6.5
6.0
5.5
5.0
4.5
4.0
3.5
3.0
2.5
2.0 ppm

STANDARD 1H
74867
2180-BGE
Berecz Gabor
2008.06.24. (BB)

Solvent: CDCl3
Temp. 30.0 C / 303.1 K
INOVA-500 "i500"

PULSE SEQUENCE
Relax. delay 1.000 sec
Pulse 90.0 degrees
Acq. time 5.000 sec
Width 8000.0 Hz
16 repetitions
OBSERVE H1, 499.5244696 MHz
DATA PROCESSING
FT size 131072
Total time 1 minutes

INDEX	FREQUENCY	PPM	HEIGHT	INDEX	FREQUENCY	PPM	HEIGHT
1	3939.697	7.887	53.3	40	1869.141	3.742	18.6
2	3937.622	7.883	51.2	41	1862.427	3.728	16.0
3	3735.474	7.478	37.8	42	1858.887	3.721	32.4
4	3733.276	7.474	36.1	43	1855.347	3.714	15.3
5	3727.173	7.461	49.0	44	1789.795	3.583	17.1
6	3724.976	7.457	46.6	45	1786.011	3.575	17.7
7	3664.673	7.336	77.2	46	1779.541	3.562	17.7
8	3660.645	7.328	2.1	47	1775.757	3.555	24.1
9	3656.494	7.320	62.1	48	1771.484	3.546	16.1
10	3626.709	7.260	18.6	49	1764.893	3.533	16.7
11	3512.085	7.031	112.5	50	1761.353	3.526	13.4
12	3402.710	6.812	145.9	51	995.605	1.993	2.9
13	2738.647	5.483	110.8	52	799.683	1.601	38.8
14	2129.028	4.262	8.2	53	-0.000	-0.000	21.8
15	2123.901	4.252	18.1				
16	2121.582	4.247	15.6				
17	2119.263	4.243	23.8				
18	2113.525	4.231	65.2				
19	2111.328	4.227	116.4				
20	2109.497	4.223	120.9				
21	2107.178	4.218	108.4				
22	2104.980	4.214	55.8				
23	2099.121	4.202	28.5				
24	2096.802	4.198	25.8				
25	2094.604	4.193	29.2				
26	2093.506	4.191	30.9				
27	2090.210	4.184	19.6				
28	2081.909	4.168	15.4				
29	2078.857	4.162	31.0				
30	2075.684	4.155	17.8				
31	2060.669	4.125	15.2				
32	2057.739	4.119	11.6				
33	2050.537	4.105	16.8				
34	2047.241	4.098	26.0				
35	2043.945	4.092	14.7				
36	2036.743	4.077	19.3				
37	2033.813	4.071	15.4				
38	1876.099	3.756	17.3				
39	1872.559	3.749	37.3				

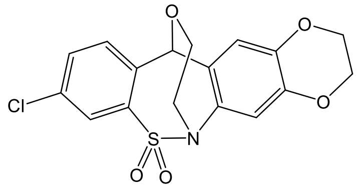

27

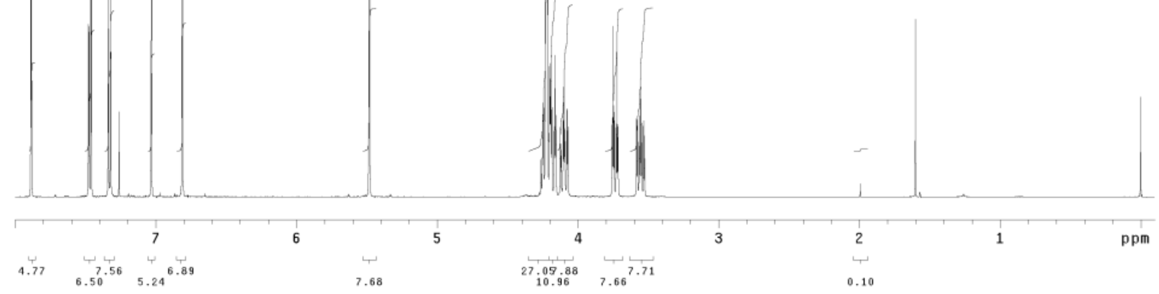

STANDARD 13C
74867
2180-BGE
Berecz Gabor
2008.06.24. (BB)

Solvent: CDCl3
Temp. 30.0 C / 303.1 K
User: 1-14-87
INOVA-500 "i500"

PULSE SEQUENCE
Relax. delay 1.200 sec
Pulse 90.0 degrees
Acq. time 1.300 sec
Width 32000.0 Hz
2019 repetitions
OBSERVE C13, 125.6055303 MHz
DECOUPLE H1, 499.5269647 MHz
Power 34 dB
continuously on
WALTZ-16 modulated
DATA PROCESSING
Line broadening 0.5 Hz
FT size 131072
Total time 84 minutes

INDEX	FREQUENCY	PPM	HEIGHT
1	18178.230	144.714	7.8
2	18111.335	144.181	26.0
3	18092.781	144.033	28.9
4	17126.960	136.345	28.1
5	16871.589	134.312	10.6
6	16715.339	133.068	68.9
7	16639.656	132.465	126.4
8	16510.261	131.435	6.0
9	16384.285	130.432	7.7
10	16070.808	127.937	120.6
11	15036.628	119.704	142.6
12	14732.429	117.282	155.8
13	10448.250	83.177	128.3
14	9704.109	77.253	43.3
15	9672.371	77.000	45.6
16	9640.144	76.743	44.0
17	8075.203	64.285	149.9
18	8061.042	64.173	154.2
19	7772.468	61.875	157.0
20	6609.382	52.616	101.6

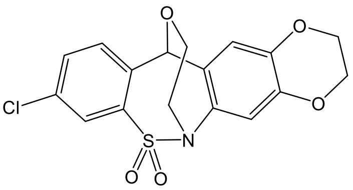

27

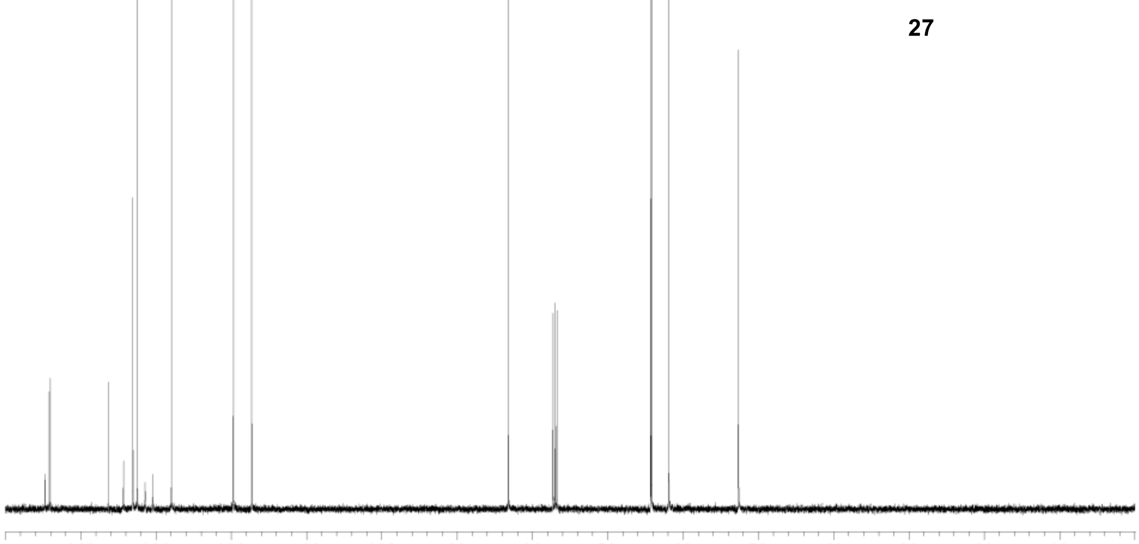

GMQFCOPS_DA
74867
2180-BGE
Berecz Gabor
2008.07.02. (DA)
Solvent: CDCl3
Temp. 30.0 C / 303.1 K
INOVA-500 "i500"
SEQUENCE: gmqfcops_da
Relax. delay 1.500 sec
Acq. time 0.197 sec
Width 5204.6 Hz
2D Width 5204.6 Hz
Single scan
2 x 256 increments
OBSERVE H1, 499.5244693 MHz
DATA PROCESSING
Sq. sine bell 0.197 sec
Shifted by -0.197 sec
F1 DATA PROCESSING
Sine bell 0.049 sec
Shifted by -0.049 sec
FT size 4096 x 512
Total time 14 minutes

27

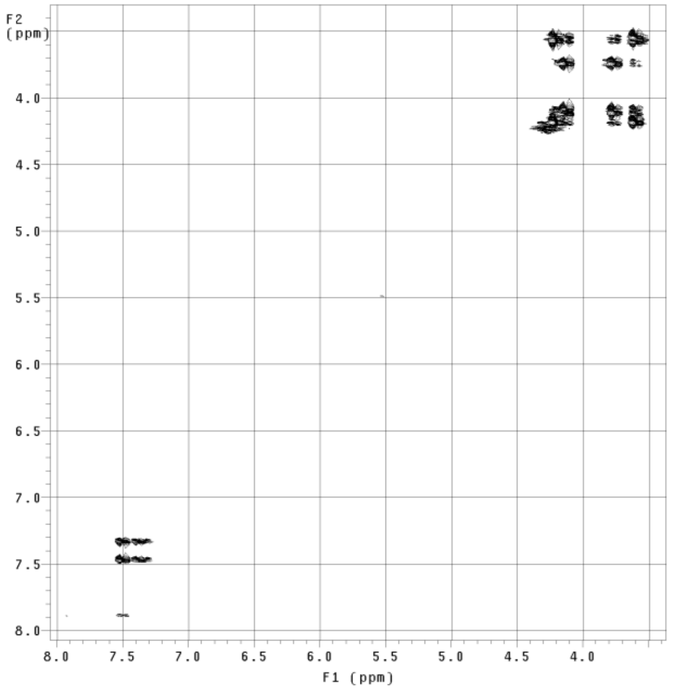

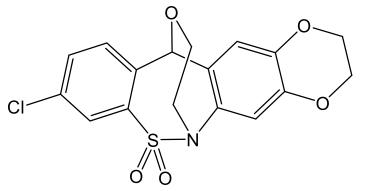

27

DPFGNOE_DA (3.74 ppm)
74867
2180-BGE
Berecz Gabor
2008.07.02. (DA)
Solvent: CDCl3
Temp. 30.0 C / 303.1 K
INOVA-500 "i500"
PULSE SEQUENCE: dpfgnoe_da
Relax. delay 2.000 sec
Pulse 90.0 degrees
Mixing 2.000 sec
Acq. time 5.000 sec
Width 8000.0 Hz
128 repetitions
OBSERVE H1, 499.5244696 MHz
DATA PROCESSING
Line broadening 1.0 Hz
FT size 131072
Total time 19 minutes

27

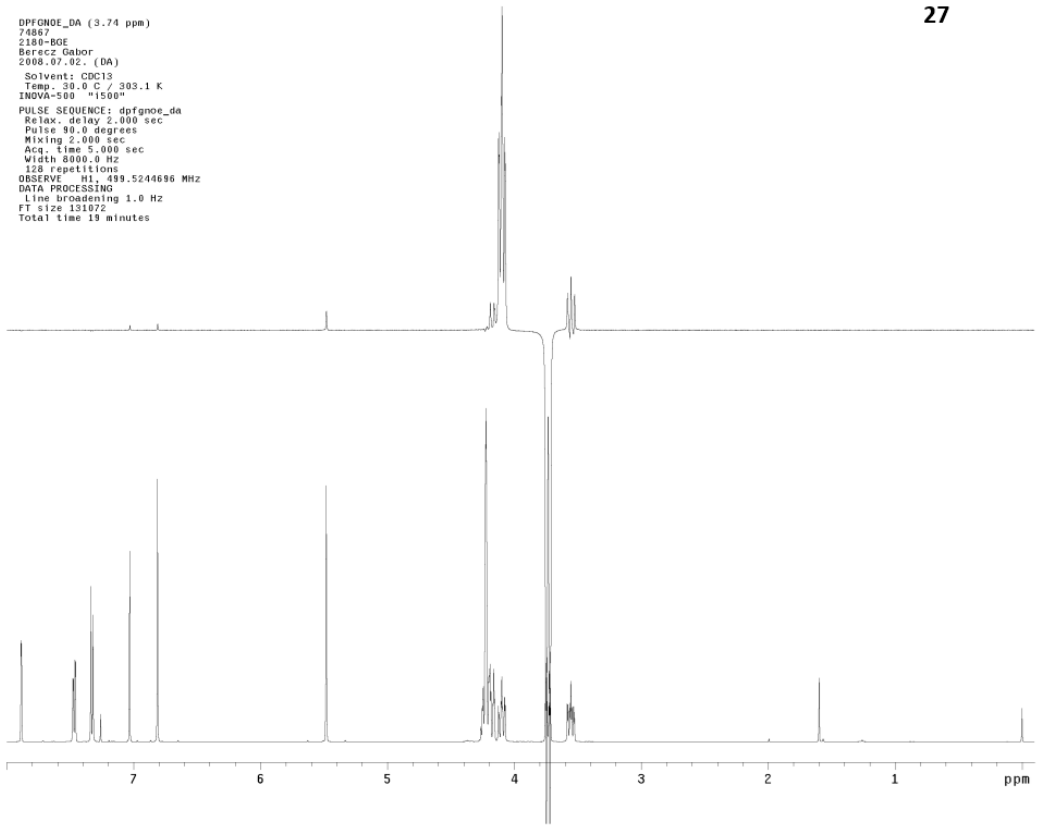

```
GHSQC_DA (140 Hz)
74867
2180-BGE
Berecz Gabor
2008.07.02. (DA)

 Solvent: CDCl3
 Temp. 30.0 C / 303.1 K
INOVA-500  "i500"

PULSE SEQUENCE: ghsqc_da
 Relax. delay 1.500 sec
 Acq. time 0.197 sec
 Width    5204.6 Hz
 2D Width 20576.1 Hz
 2 repetitions
 2 x 256 increments
OBSERVE   H1, 499.5244709 MHz
DECOUPLE C13, 125.6148077 MHz
 Power 44 dB
 on during acquisition
 off during delay
 GARP-1 modulated
DATA PROCESSING
 Sq. sine bell 0.203 sec
  Shifted by -0.203 sec
F1 DATA PROCESSING
 Sq. sine bell 0.006 sec
  Shifted by -0.006 sec
FT size 4096 x 512
Total time 29 minutes
```

27

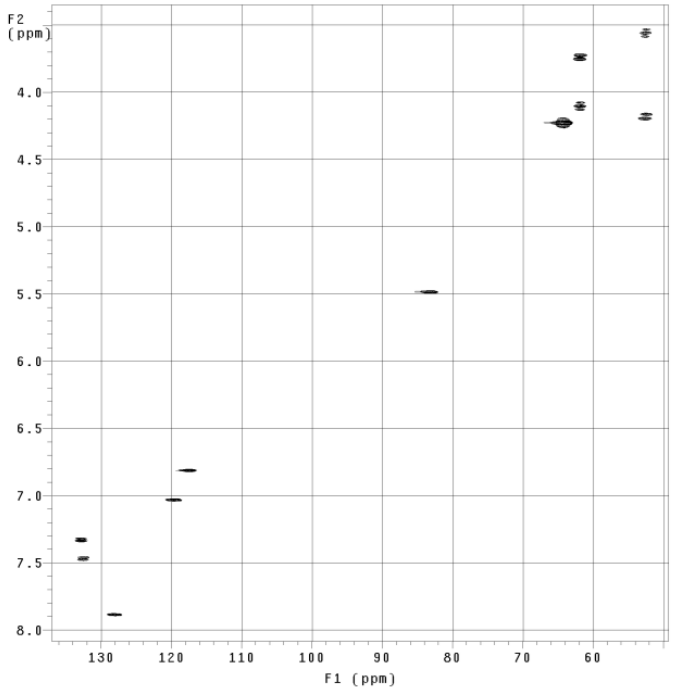

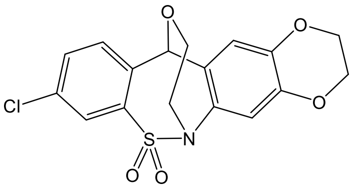

27

```
GHMQC_DA (140 Hz, 8 Hz)
74867
2180-BGE
Berecz Gabor
2008.07.02. (DA)

 Solvent: CDCl3
 Temp. 30.0 C / 303.1 K
INOVA-500  "i500"

PULSE SEQUENCE: ghmqc_da
 Relax. delay 1.500 sec
 Acq. time 0.197 sec
 Width    5204.6 Hz
 2D Width 20576.1 Hz
 4 repetitions
 256 increments
OBSERVE   H1, 499.5244711 MHz
DATA PROCESSING
 Sine bell 0.098 sec
F1 DATA PROCESSING
 Sine bell 0.003 sec
FT size 4096 x 512
Total time 29 minutes
```

27

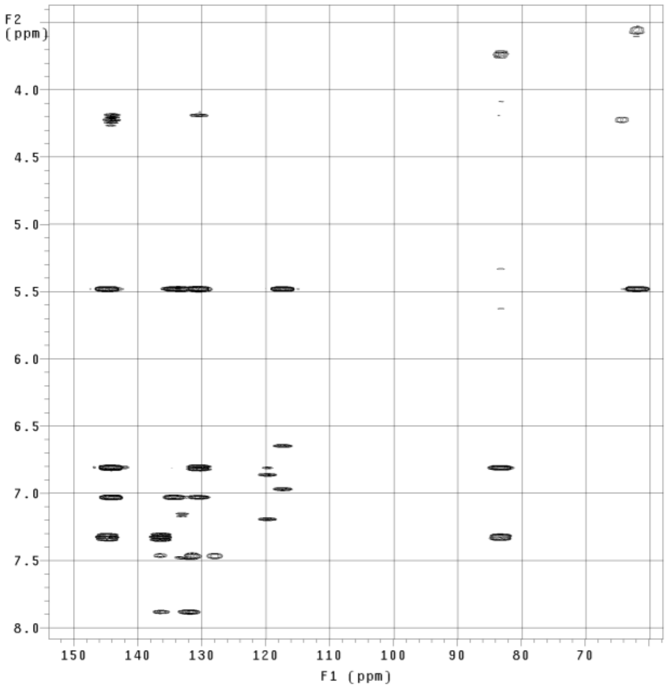

STANDARD 1H
64576
1986-BGE
Berecz Gabor
2006.07.25. (BT)

Solvent: DMSO
Temp. 25.0 C / 298.1 K
INOVA-500 "i500"

PULSE SEQUENCE
Relax. delay 3.000 sec
Pulse 6.4 usec
Acq. time 5.000 sec
Width 8000.0 Hz
16 repetitions
OBSERVE H1, 499.5268278 MHz
DATA PROCESSING
FT size 131072
Total time 2 minutes

INDEX	FREQUENCY	PPM	HEIGHT
1	5856.567	11.724	4.5
2	3950.317	7.908	27.7
3	3948.242	7.904	36.9
4	3942.139	7.892	33.7
5	3940.063	7.888	73.9
6	3937.500	7.882	63.4
7	3937.134	7.882	69.1
8	3935.425	7.878	42.0
9	3917.114	7.842	67.1
10	3916.626	7.841	58.7
11	3908.936	7.825	43.3
12	3908.447	7.824	38.0
13	3751.343	7.510	121.7
14	3344.116	6.695	121.8
15	2181.763	4.368	21.2
16	2178.955	4.362	31.4
17	2175.903	4.356	30.2
18	2173.828	4.352	33.8
19	2145.386	4.295	35.7
20	2143.311	4.291	28.7
21	2140.625	4.285	31.6
22	2137.451	4.279	22.1
23	1260.132	2.523	4.7
24	1258.179	2.519	10.8
25	1256.348	2.515	15.2
26	1254.517	2.511	10.7
27	1252.686	2.508	4.7
28	1042.236	2.086	13.4
29	-0.000	-0.000	27.5

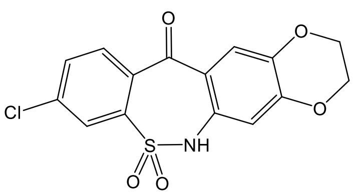

28

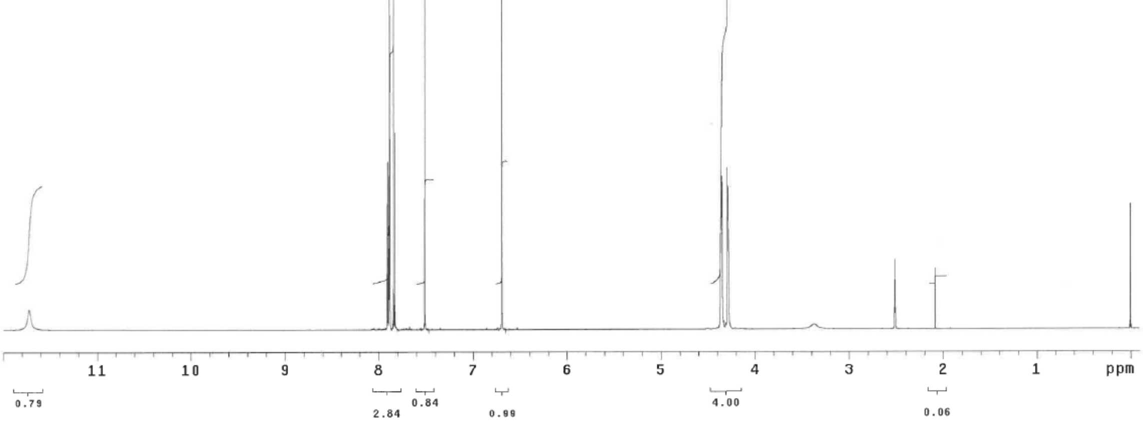

STANDARD 13C
64576
1986-BGE
Berecz Gabor
2006.07.25. (BT)

Solvent: DMSO
Temp. 25.0 C / 298.1 K
User: 1-14-87
INOVA-500 "i500"

PULSE SEQUENCE
Relax. delay 3.000 sec
Pulse 6.0 usec
Acq. time 1.300 sec
Width 28208.7 Hz
128 repetitions
OBSERVE C13, 125.6061583 MHz
DECOUPLE H1, 499.5293374 MHz
Power 32 dB
continuously on
WALTZ-16 modulated
DATA PROCESSING
Line broadening 0.5 Hz
FT size 131072
Total time 9 minutes

INDEX	FREQUENCY	PPM	HEIGHT
1	23766.049	189.203	47.1
2	18782.946	149.532	63.0
3	17652.633	140.534	58.0
4	17541.582	139.650	62.3
5	17226.076	137.138	59.1
6	16889.909	134.462	51.5
7	16770.680	133.513	59.4
8	16702.241	132.968	61.7
9	16631.220	132.402	59.0
10	15325.722	122.009	62.8
11	15119.976	120.371	59.7
12	14867.313	118.360	67.3
13	13660.383	108.751	66.7
14	8187.449	65.181	52.4
15	8047.990	64.071	51.8
16	5050.036	40.204	30.0
17	5028.945	40.036	95.7
18	5007.854	39.868	186.4
19	4986.762	39.700	214.3
20	4966.102	39.536	186.0
21	4945.011	39.368	94.5
22	4923.919	39.200	30.3

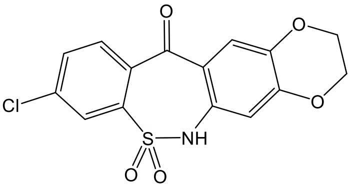

28

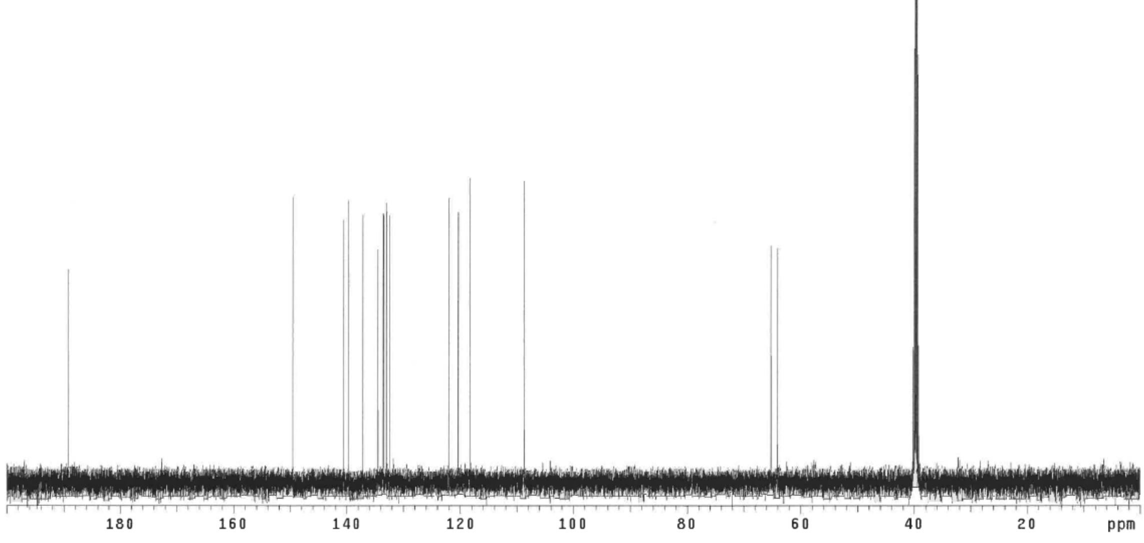

```
STANDARD 1H
73018
2155-BGE
Berecz Gabor
2008.02.27. (BT)

 Solvent: CDCl3
 Temp. 25.0 C / 298.1 K
INOVA-500  "i500"

PULSE SEQUENCE
 Relax. delay 1.000 sec
 Pulse 6.4 usec
 Acq. time 5.000 sec
 Width 8000.0 Hz
 32 repetitions
OBSERVE   H1, 499.5244681 MHz
DATA PROCESSING
FT size 131072
Total time 3 minutes
```

INDEX	FREQUENCY	PPM	HEIGHT
1	4041.992	8.092	57.0
2	4033.569	8.075	61.5
3	3985.840	7.979	51.2
4	3983.765	7.975	53.5
5	3921.143	7.850	103.4
6	3835.205	7.678	40.3
7	3833.008	7.673	39.2
8	3826.782	7.661	37.3
9	3824.585	7.656	36.6
10	3628.540	7.264	21.6
11	3429.932	6.866	114.6
12	2178.955	4.362	24.7
13	2175.903	4.356	39.5
14	2172.607	4.349	37.2
15	2170.654	4.345	40.8
16	2169.678	4.343	33.4
17	2154.175	4.312	38.4
18	2153.198	4.310	37.8
19	2151.245	4.307	37.6
20	2149.170	4.302	35.1
21	2148.560	4.301	36.0
22	2147.949	4.300	34.0
23	2145.508	4.295	24.9
24	2005.005	4.014	24.7
25	1997.803	3.999	49.1
26	1990.723	3.985	25.9
27	1690.308	3.384	16.6
28	1683.350	3.370	29.5
29	1676.270	3.356	14.7
30	1000.000	2.002	2.7
31	792.358	1.586	18.6
32	0.000	0.000	22.1

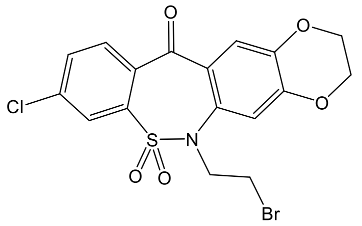

29

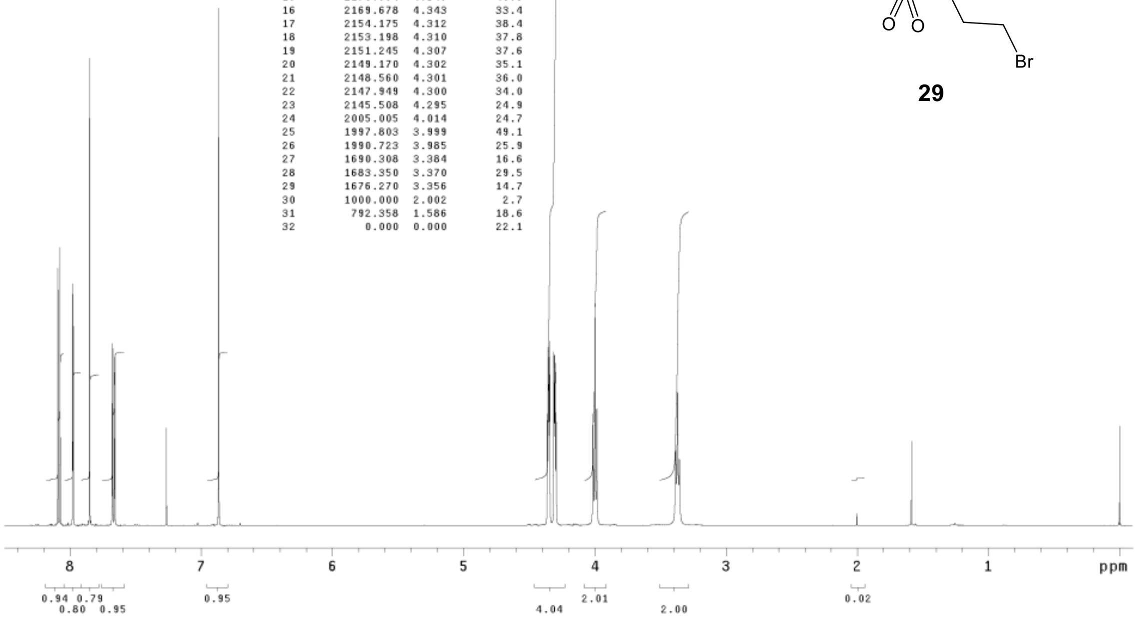


```
STANDARD 13C
73018
2155-BGE
Berecz Gabor
2008.02.27. (BT)

 Solvent: CDCl3
 Temp. 25.0 C / 298.1 K
User:  1-14-87
INOVA-500  "i500"

PULSE SEQUENCE
 Relax. delay 3.000 sec
 Pulse 6.0 usec
 Acq. time 1.300 sec
 Width 32000.0 Hz
 512 repetitions
OBSERVE  C13, 125.6055313 MHz
DECOUPLE  H1, 499.5269647 MHz
 Power 32 dB
 continuously on
 WALTZ-16 modulated
DATA PROCESSING
 Line broadening 0.5 Hz
FT size 131072
Total time 36 minutes
```

INDEX	FREQUENCY	PPM	HEIGHT
1	23416.511	186.415	38.4
2	18733.406	149.133	48.8
3	17943.367	142.844	48.0
4	17544.441	139.668	37.2
5	17454.597	138.953	41.8
6	16862.800	134.242	86.1
7	16770.515	133.507	47.4
8	16729.500	133.181	89.0
9	16699.226	132.940	46.1
10	15929.207	126.810	31.7
11	15802.742	125.803	99.0
12	15209.968	121.084	96.0
13	14301.277	113.850	86.7
14	9704.109	77.253	138.0
15	9672.371	77.000	142.0
16	9640.144	76.743	139.1
17	8158.211	64.946	89.4
18	8045.906	64.052	87.6
19	6678.718	53.168	69.2
20	3504.402	27.898	73.6

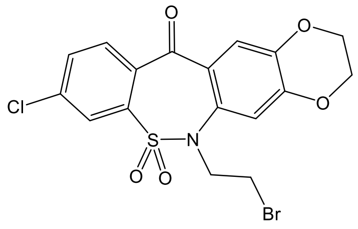

29

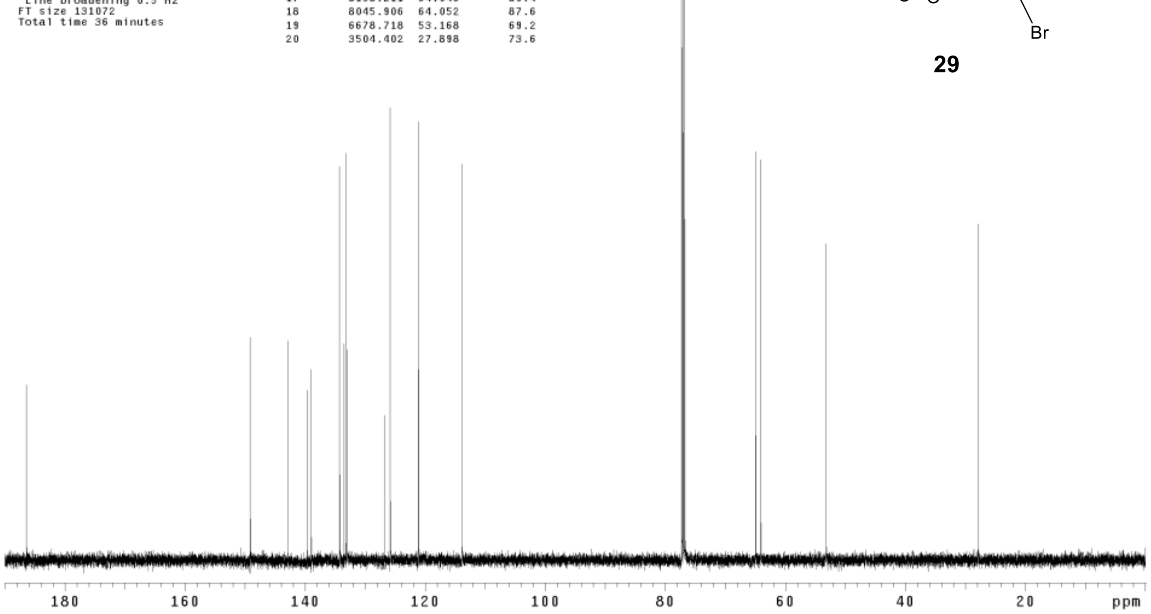

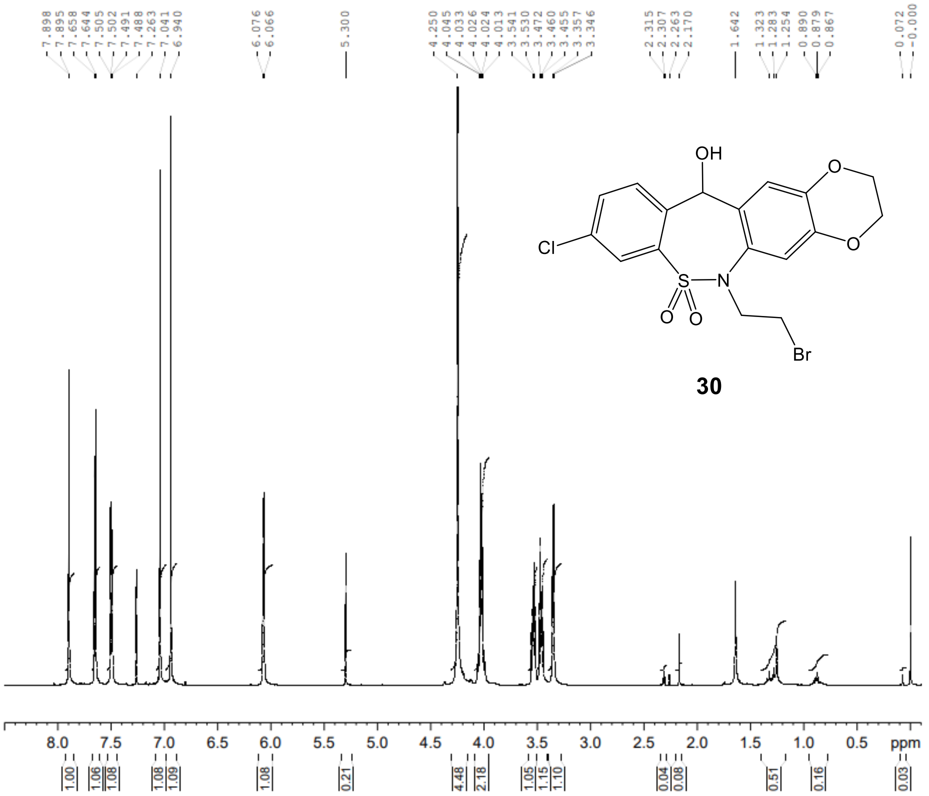

Standard 1H
144447
bge2363-2363-BGE_1
Berecz Gabor
2025.03.28. (KP)

Current Data Parameters

NAME	144447
EXPNO	11
PROCNO	1

F2 - Acquisition Parameters

Date_	20250328
Time	10.34 h
INSTRUM	spect
PROBHD	Z145856_0002 (
PULPROG	zg30
TD	65536
SOLVENT	CDCl3
NS	16
DS	2
SWH	12019.230 Hz
FIDRES	0.366798 Hz
AQ	2.7262976 sec
RG	196.07
DW	41.600 usec
DE	25.00 usec
TE	295.0 K
D1	1.00000000 sec
TD0	1
SFO1	600.0037050 MHz
NUC1	1H
P1	11.50 usec
PLW1	28.00000000 W

F2 - Processing parameters

SI	65536
SF	600.0000120 MHz
WDW	EM
SSB	0
LB	0.30 Hz
GB	0
PC	1.00

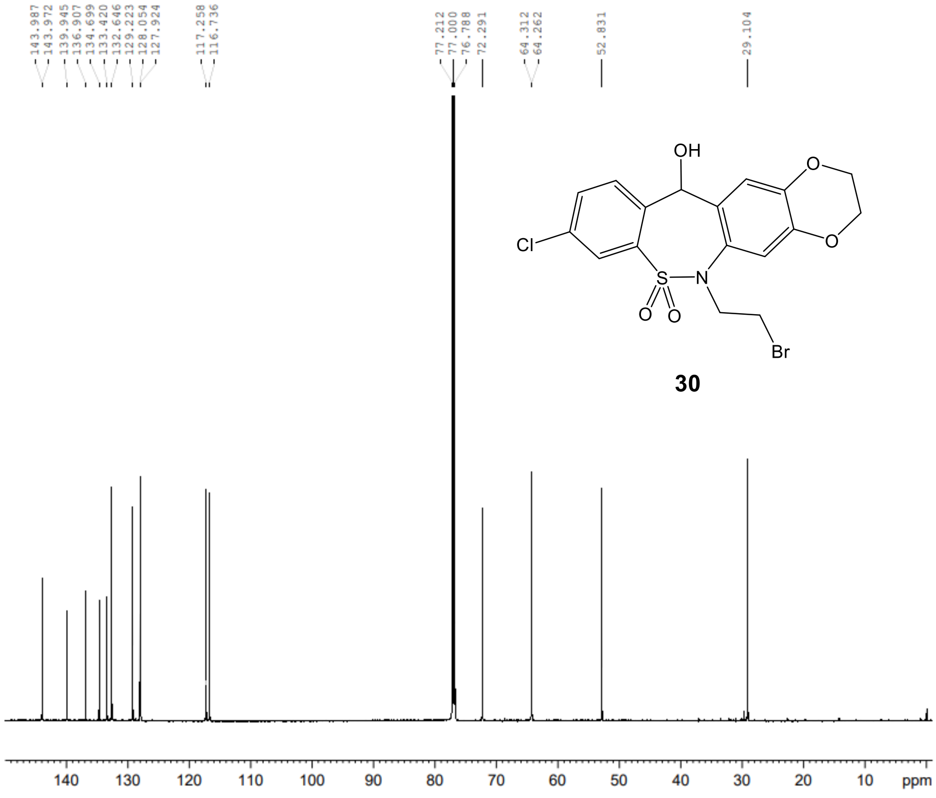

Standard 13C
144447
bge2363-2363-BGE_1
Berecz Gabor
2025.03.28. (KP)

Current Data Parameters

NAME	144447
EXPNO	21
PROCNO	1

F2 - Acquisition Parameters

Date_	20250329
Time	12.15 h
INSTRUM	spect
PROBHD	Z145856_0002 (
PULPROG	zgpg30
TD	65536
SOLVENT	CDCl3
NS	2048
DS	4
SWH	36231.883 Hz
FIDRES	1.105709 Hz
AQ	0.9043968 sec
RG	196.07
DW	13.800 usec
DE	18.00 usec
TE	295.0 K
D1	1.00000000 sec
D11	0.03000000 sec
TD0	1
SFO1	150.8852070 MHz
NUC1	13C
P1	9.80 usec
PLW1	72.69999695 W
SFO2	600.0024000 MHz
NUC2	1H
CPDPRG[2	waltz16
PCPD2	80.00 usec
PLW2	32.50000000 W
PLW12	0.70708001 W
PLW13	0.35508999 W

F2 - Processing parameters

SI	131072
SF	150.8701299 MHz
WDW	EM
SSB	0
LB	1.00 Hz
GB	0
PC	1.40

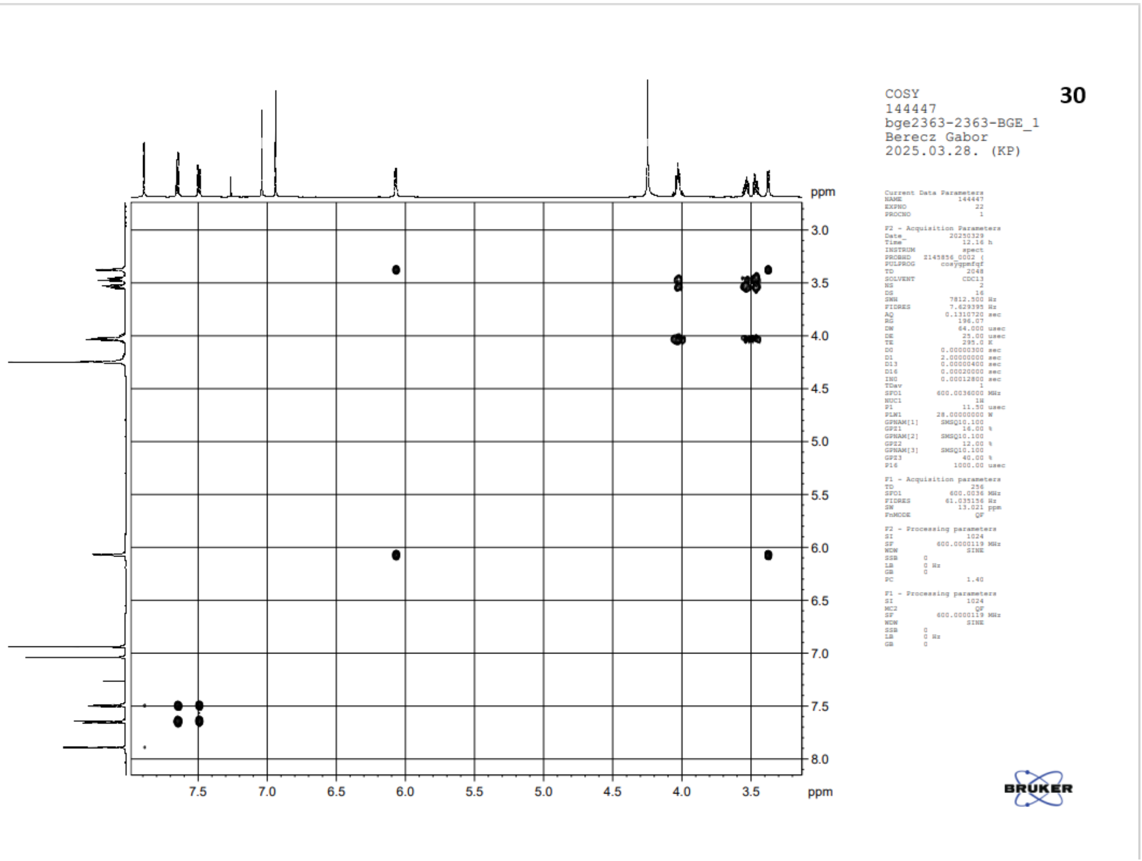
COSY
144447
bge2363-2363-BGE_1
Berecz Gabor
2025.03.28. (KP)
30
ppm
BRUKER

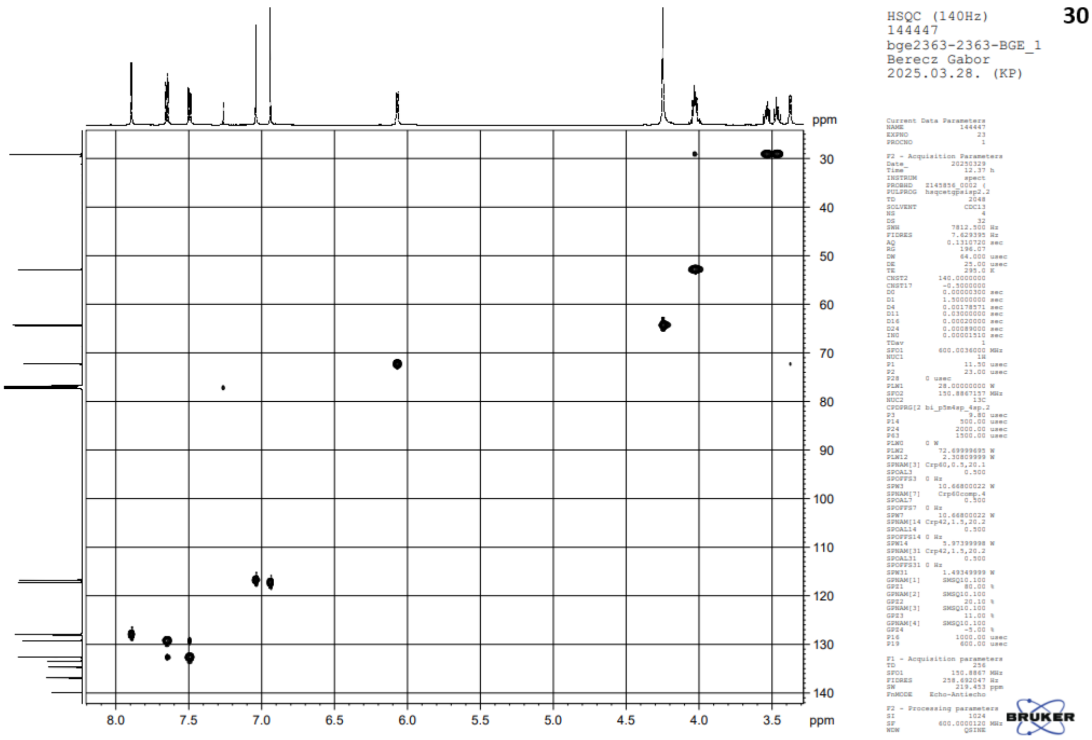
HSQC (140Hz)
144447
bge2363-2363-BGE_1
Berecz Gabor
2025.03.28. (KP)
30
ppm
BRUKER

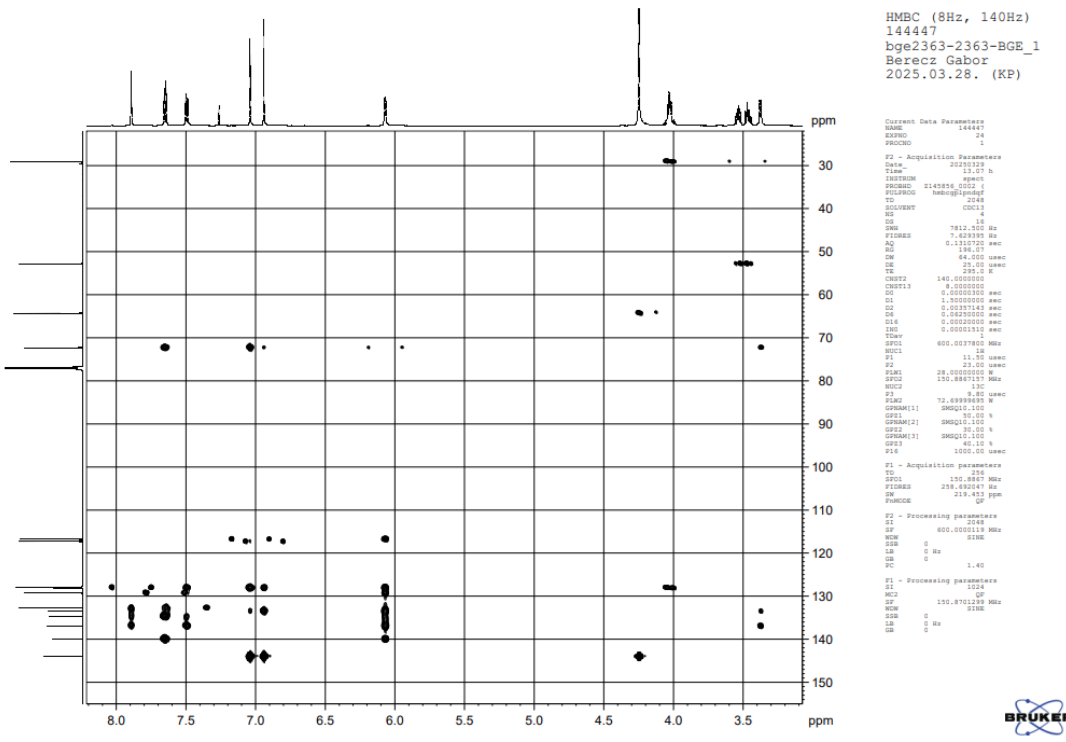

HMBC (8Hz, 140Hz)
144447
bge2363-2363-BGE_1
Berecz Gabor
2025.03.28. (KP)
Current Data Parameters
NAME 144447
EXPNO 24
PROCNO 1
F2 - Acquisition Parameters
Date_ 20250329
Time 13.07 h
INSTRUM spect
PROBHD Z145856_0002 (
PULPROG hmbcgplpndqf
TD 2048
SOLVENT CDCl3
NS 4
DS 16
SWH 7812.500 Hz
FIDRES 7.629395 Hz
AQ 0.1310720 sec
RG 196.07
DW 64.000 usec
DE 25.00 usec
TE 295.0 K
CNST2 140.0000000
CNST13 8.0000000
D0 0.00000300 sec
D1 1.50000000 sec
D2 0.00357143 sec
D6 0.06250000 sec
D16 0.00020000 sec
IN0 0.00001510 sec
TDav 1
SFO1 600.0037800 MHz
NUC1 1H
P1 11.50 usec
P2 23.00 usec
PLW1 28.00000000 W
SFO2 150.8867157 MHz
NUC2 13C
P3 9.80 usec
PLW2 72.69999695 W
GPNAM[1] SMSQ10.100
GPZ1 50.00 %
GPNAM[2] SMSQ10.100
GPZ2 30.00 %
GPNAM[3] SMSQ10.100
GPZ3 40.10 %
P16 1000.00 usec
F1 - Acquisition parameters
TD 256
SFO1 150.8867 MHz
FIDRES 258.692047 Hz
SW 219.453 ppm
FnMODE QF
F2 - Processing parameters
SI 2048
SF 600.0000119 MHz
WDW SINE
SSB 0
LB 0 Hz
GB 0
PC 1.40
F1 - Processing parameters
SI 1024
MC2 QF
SF 150.8701299 MHz
WDW SINE
SSB 0
LB 0 Hz
GB 0
BRUKER
ppm
30
40
50
60
70
80
90
100
110
120
130
140
150
8.0
7.5
7.0
6.5
6.0
5.5
5.0
4.5
4.0
3.5
ppm

30

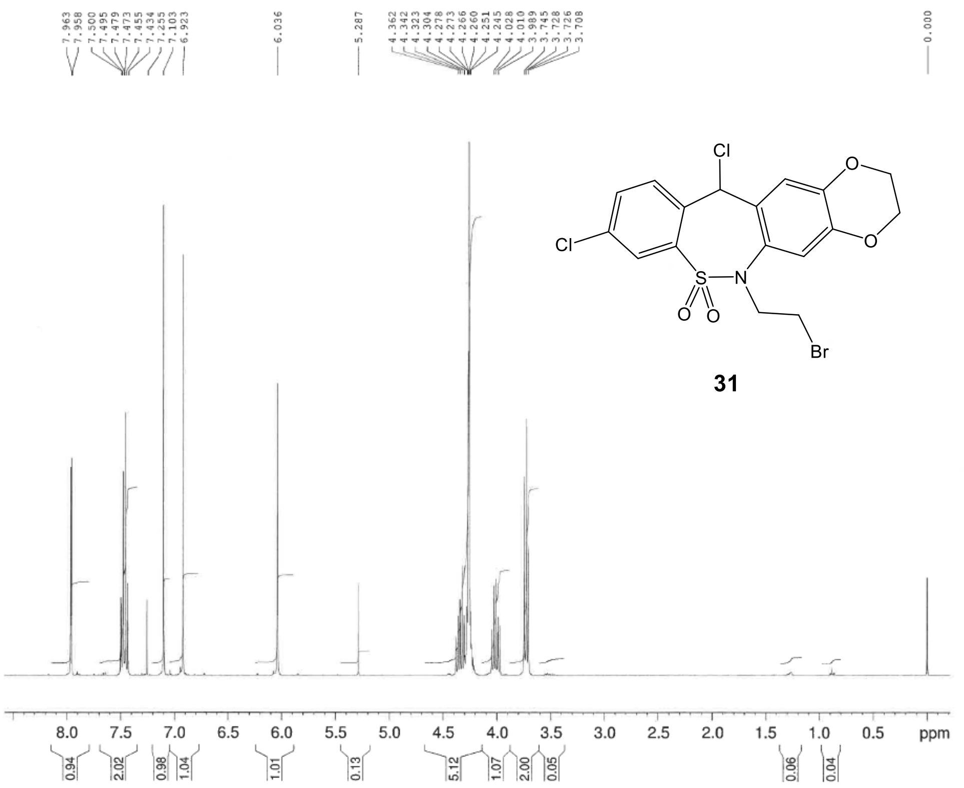

Standard 1H
85354
2364-BGE
Berecz Gabor
2010.03.18. (BB)

```
NAME              85354
EXPNO                 1
PROCNO                1
Date_          20100318
Time              14.34
INSTRUM           spect
PROBHD   5 mm PABBO BB-
PULPROG            zg30
TD                65536
SOLVENT           CDCl3
NS                   64
DS                    2
SWH            8223.685 Hz
FIDRES         0.125483 Hz
AQ            3.9846387 sec
RG                 71.8
DW               60.800 usec
DE                 6.50 usec
TE                303.0 K
D1           1.00000000 sec
TD0                   1

======== CHANNEL f1 ========
NUC1                 1H
P1                12.50 usec
PL1                1.00 dB
PL1W        11.97157383 W
SFO1        400.1324710 MHz
SI                32768
SF          400.1300114 MHz
WDW                  no
SSB                   0
LB                 0.00 Hz
GB                    0
PC                 1.00
```

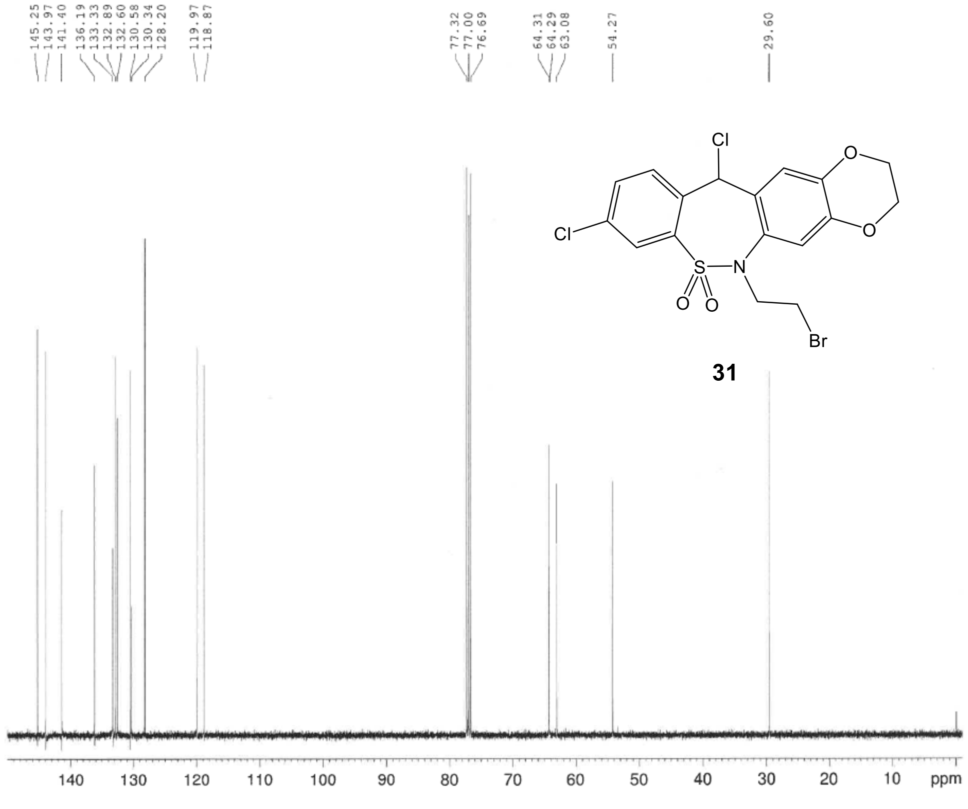

Standard 13C
85354
2364-BGE
Berecz Gabor
2010.03.18. (BB)

```
NAME              85354
EXPNO                 2
PROCNO                1
Date_          20100318
Time              15.28
INSTRUM           spect
PROBHD   5 mm PABBO BB-
PULPROG          zgpg30
TD                65536
SOLVENT           CDCl3
NS                 1024
DS                    4
SWH           24038.461 Hz
FIDRES         0.366798 Hz
AQ            1.3631988 sec
RG                 2050
DW               20.800 usec
DE                 6.50 usec
TE                303.0 K
D1           2.00000000 sec
D11          0.03000000 sec
TD0                   1

======== CHANNEL f1 ========
NUC1                13C
P1                 9.50 usec
PL1                1.80 dB
PL1W        40.49391937 W
SFO1        100.6228298 MHz

======== CHANNEL f2 ========
CPDPRG2         waltz16
NUC2                 1H
PCPD2             90.00 usec
PL2                2.00 dB
PL12              20.00 dB
PL13              20.00 dB
PL2W         9.50935936 W
PL12W        0.15071319 W
PL13W        0.15071319 W
SFO2        400.1316005 MHz
SI                32768
SF          100.6127744 MHz
WDW                  no
SSB                   0
LB                 0.00 Hz
GB                    0
PC                 1.40
```

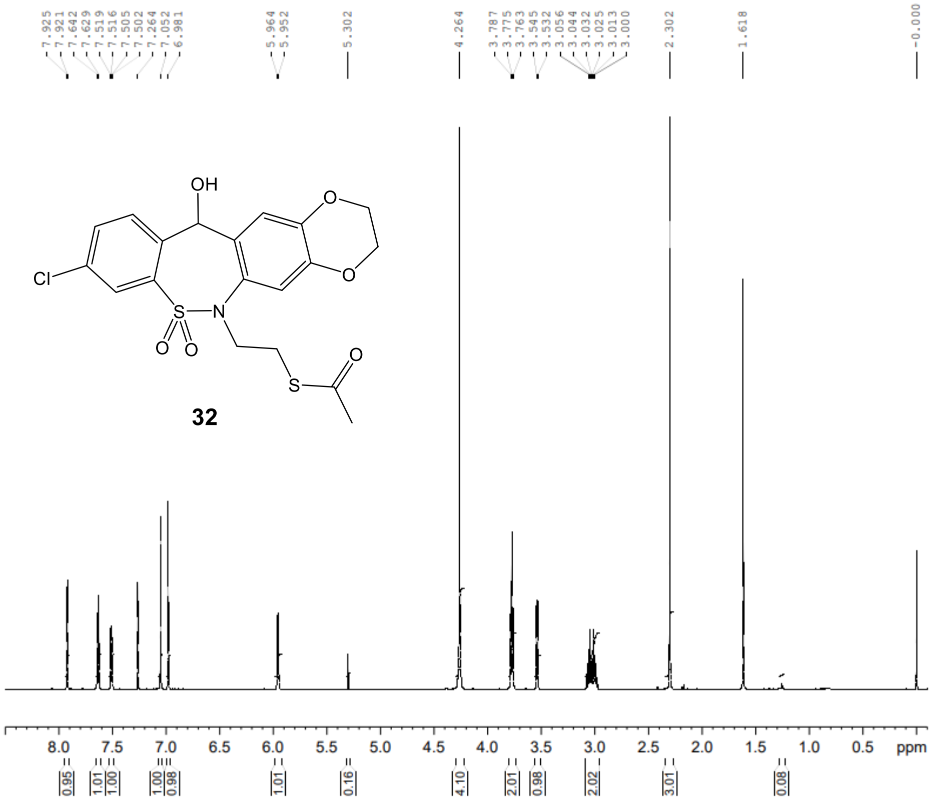


```
Standard 1H
143408
BEG0700_1A
Berecz Gabor
2024.09.02. (KP)

Current Data Parameters
NAME              143408
EXPNO                 11
PROCNO                 1

F2 - Acquisition Parameters
Date_           20240902
Time               17.48 h
INSTRUM            spect
PROBHD   Z145856_0002 (
PULPROG             zg30
TD                 65536
SOLVENT            CDCl3
NS                    16
DS                     2
SWH            12019.230 Hz
FIDRES          0.366798 Hz
AQ             2.7262976 sec
RG                196.07
DW                41.600 usec
DE                 25.00 usec
TE                 295.0 K
D1            1.00000000 sec
TD0                    1
SFO1         600.0037050 MHz
NUC1                  1H
P1                 11.50 usec
PLW1         28.00000000 W

F2 - Processing parameters
SI                 65536
SF           600.0000127 MHz
WDW                   EM
SSB      0
LB                  0.30 Hz
GB       0
PC                  1.00
```

BRUKER

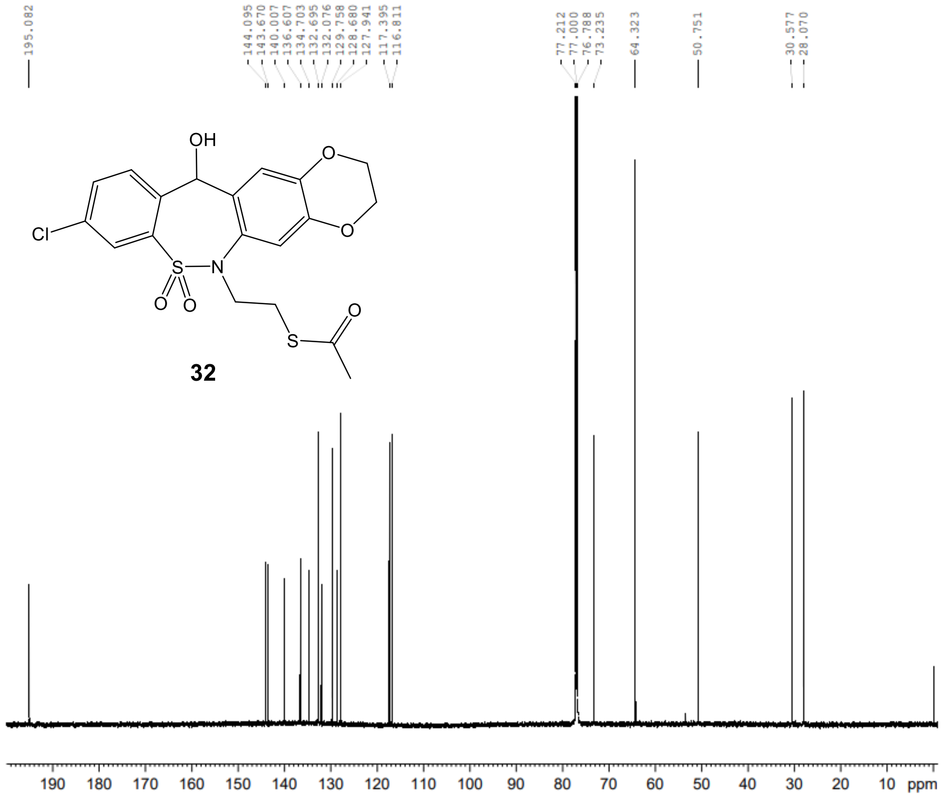


```
Standard 13C
143408
BEG0700_1A
Berecz Gabor
2024.09.02. (KP)

Current Data Parameters
NAME              143408
EXPNO                 12
PROCNO                 1

F2 - Acquisition Parameters
Date_           20240902
Time               18.57 h
INSTRUM            spect
PROBHD   Z145856_0002 (
PULPROG          zgpg30
TD                 65536
SOLVENT            CDCl3
NS                  2048
DS                     4
SWH            36231.883 Hz
FIDRES          1.105709 Hz
AQ             0.9043968 sec
RG                196.07
DW                13.800 usec
DE                 18.00 usec
TE                 295.0 K
D1            1.00000000 sec
D11           0.03000000 sec
TD0                    1
SFO1         150.8852070 MHz
NUC1                 13C
P1                  9.90 usec
PLW1         71.00000000 W
SFO2         600.0024000 MHz
NUC2                  1H
CPDPRG[2         waltz16
PCPD2              80.00 usec
PLW2         32.90000153 W
PLW12         0.70370001 W
PLW13         0.35339001 W

F2 - Processing parameters
SI                131072
SF           150.8701271 MHz
WDW                   EM
SSB      0
LB                  1.00 Hz
GB       0
PC                  1.40
```

BRUKER

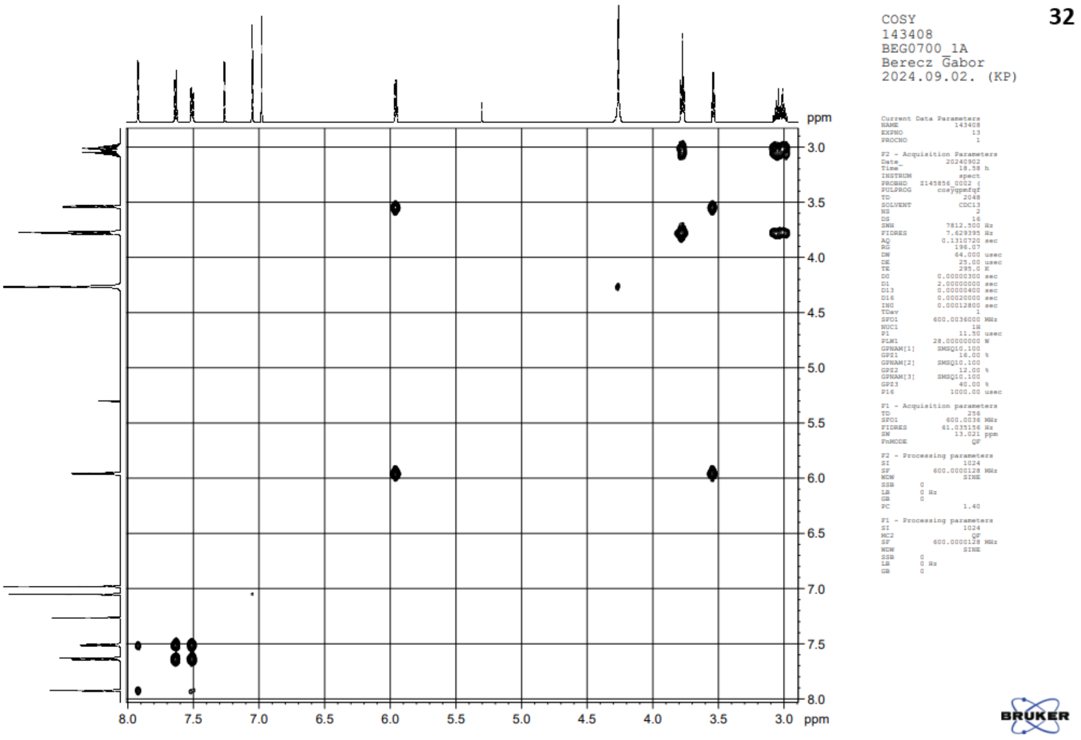
COSY
143408
BEG0700_1A
Berecz Gabor
2024.09.02. (KP)
32
ppm
BRUKER

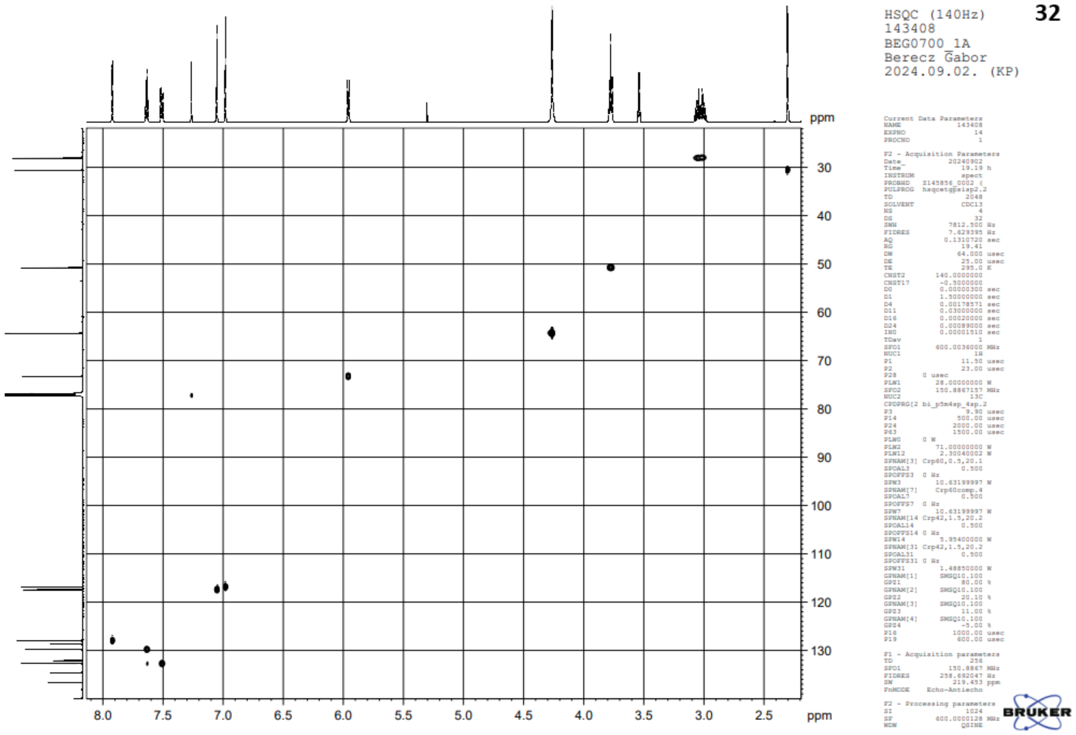
HSQC (140Hz)
143408
BEG0700_1A
Berecz Gabor
2024.09.02. (KP)
32
ppm
BRUKER

32

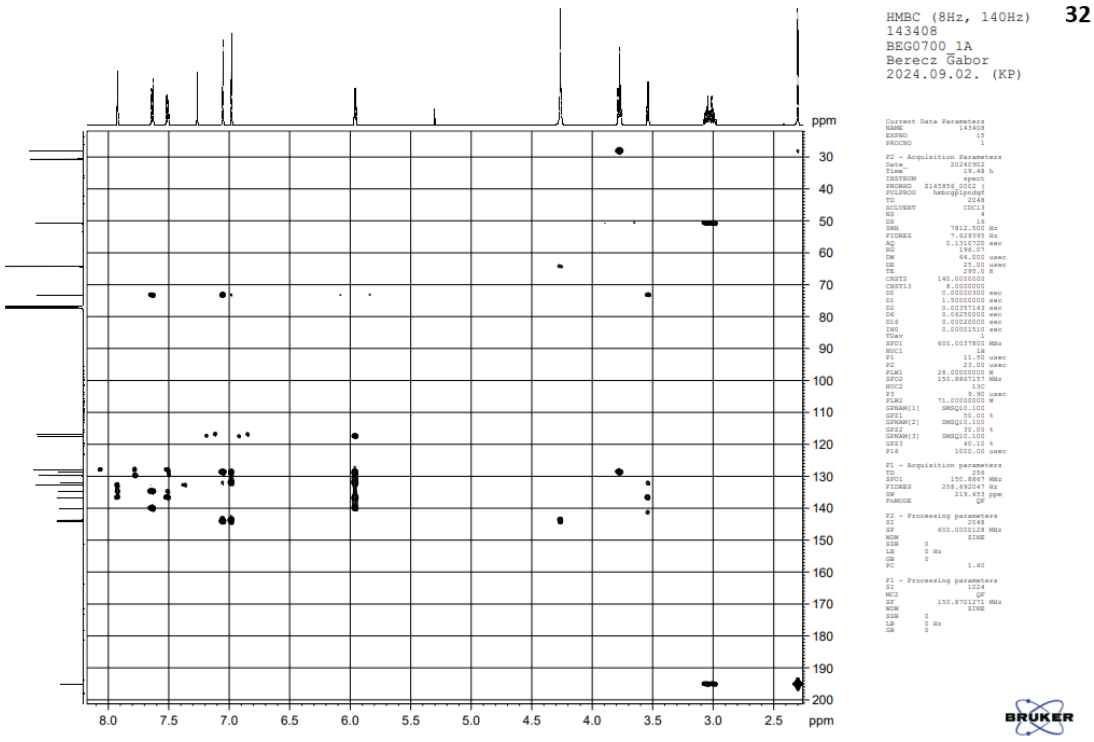

HMBC (8Hz, 140Hz)
143408
BEG0700_1A
Berecz Gabor
2024.09.02. (KP)
ppm
BRUKER

STANDARD 1H
65409
2007-BGE
Berecz Gabor
2006.09.20. (BT)

Solvent: DMSO
Temp. 25.0 C / 298.1 K
INOVA-500 "i500"

PULSE SEQUENCE
Relax. delay 3.000 sec
Pulse 6.4 usec
Acq. time 5.000 sec
Width 8000.0 Hz
16 repetitions
OBSERVE H1, 499.5268312 MHz
DATA PROCESSING
FT size 131072
Total time 2 minutes

INDEX	FREQUENCY	PPM	HEIGHT
1	5182.129	10.374	2.9
2	3937.378	7.882	32.4
3	3934.448	7.876	9.5
4	3931.152	7.870	8.6
5	3928.223	7.864	40.0
6	3855.469	7.718	23.5
7	3853.271	7.714	41.8
8	3848.999	7.705	57.1
9	3846.802	7.701	98.1
10	3844.116	7.696	10.1
11	3544.922	7.097	87.3
12	3350.342	6.707	122.4
13	3331.787	6.670	2.6
14	3330.200	6.667	3.1
15	3324.219	6.655	3.1
16	3315.674	6.638	4.0
17	3243.774	6.494	51.4
18	2117.432	4.239	5.1
19	2110.596	4.225	13.9
20	2103.271	4.211	140.5
21	2095.703	4.195	14.0
22	2088.989	4.182	4.6
23	2084.473	4.173	2.9
24	1696.899	3.397	5.4
25	1689.819	3.383	16.5
26	1682.861	3.369	20.1
27	1675.903	3.355	6.6
28	1256.226	2.515	3.7
29	1254.639	2.512	7.6
30	1252.808	2.508	10.1
31	1250.977	2.504	7.1
32	1249.268	2.501	3.2
33	1038.696	2.079	4.7
34	551.880	1.105	10.9
35	544.922	1.091	21.5
36	537.842	1.077	10.5
37	-0.000	-0.000	4.5

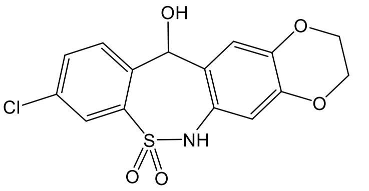

33

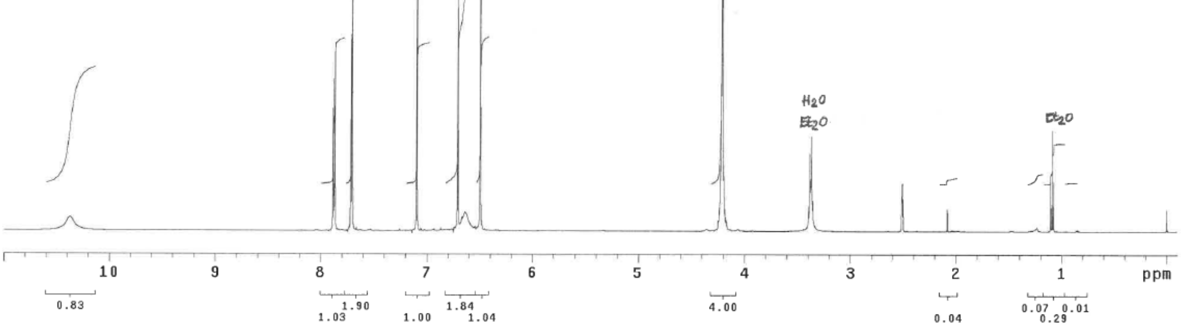

STANDARD 13C
65409
2007-BGE
Berecz Gabor
2006.09.20. (BT)

Solvent: DMSO
Temp. 25.0 C / 298.1 K
User: 1-14-87
INOVA-500 "i500"

PULSE SEQUENCE
Relax. delay 3.000 sec
Pulse 6.0 usec
Acq. time 1.300 sec
Width 32000.0 Hz
784 repetitions
OBSERVE C13, 125.6061553 MHz
DECOUPLE H1, 499.5293374 MHz
Power 32 dB
continuously on
WALTZ-16 modulated
DATA PROCESSING
Line broadening 0.5 Hz
FT size 131072
Total time 56 minutes

INDEX	FREQUENCY	PPM	HEIGHT
1	17926.704	142.716	78.1
2	17869.087	142.257	72.7
3	17548.774	139.707	78.1
4	17511.176	139.408	66.7
5	16657.172	132.609	72.1
6	16633.247	132.419	72.3
7	16589.790	132.073	69.8
8	16007.758	127.439	70.3
9	15773.872	125.577	72.6
10	15735.297	125.270	77.3
11	14452.583	115.058	71.2
12	14274.360	113.639	74.1
13	8264.594	65.795	74.7
14	8179.145	65.115	11.2
15	8079.536	64.322	73.1
16	8077.583	64.306	73.9
17	5050.239	40.205	27.4
18	5028.755	40.034	84.8
19	5019.477	39.960	7.8
20	5007.758	39.867	169.8
21	4986.762	39.700	200.0
22	4965.766	39.533	170.2
23	4944.770	39.366	85.5
24	4924.262	39.202	27.8
25	1929.145	15.358	11.8

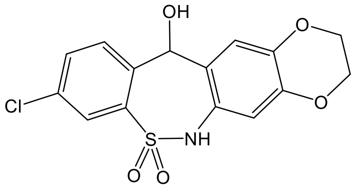

33

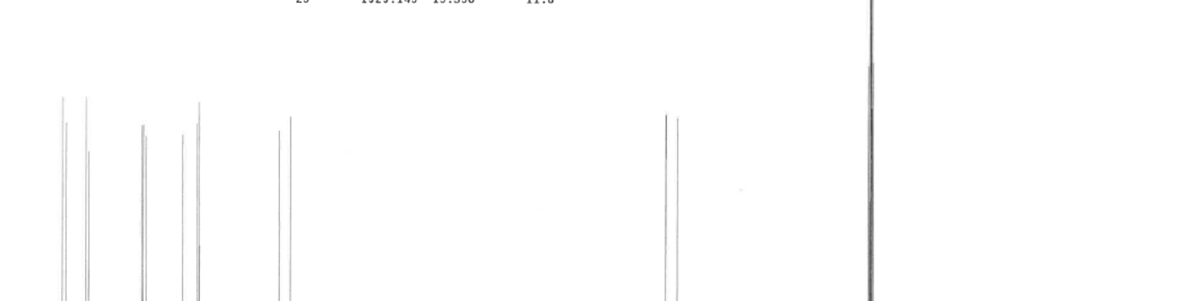

STANDARD 1H
67203
2039-BGE
Berecz Gabor
2007.01.16. (BB)
Solvent: CDCl3
Temp. 25.0 C / 298.1 K
INOVA-500 "i500"
PULSE SEQUENCE
Relax. delay 1.000 sec
Pulse 6.4 usec
Acq. time 5.000 sec
Width 8000.0 Hz
16 repetitions
OBSERVE H1, 499.5244692 MHz
DATA PROCESSING
FT size 131072
Total time 1 minutes

INDEX	FREQUENCY	PPM	HEIGHT
1	3954.712	7.917	24.4
2	3952.515	7.913	24.8
3	3731.934	7.471	13.1
4	3729.736	7.467	12.7
5	3723.633	7.454	18.4
6	3721.436	7.450	17.8
7	3682.251	7.372	25.1
8	3673.828	7.355	18.0
9	3626.831	7.261	12.8
10	3612.793	7.232	15.6
11	3534.058	7.075	42.1
12	3435.791	6.878	49.3
13	2927.246	5.860	32.8
14	2645.386	5.296	111.9
15	2126.587	4.257	27.1
16	2123.535	4.251	42.7
17	2119.751	4.244	24.2
18	2102.173	4.208	7.0
19	-0.000	-0.000	15.2

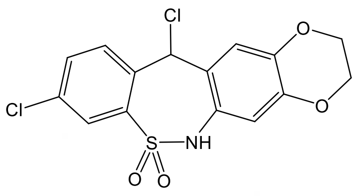

34

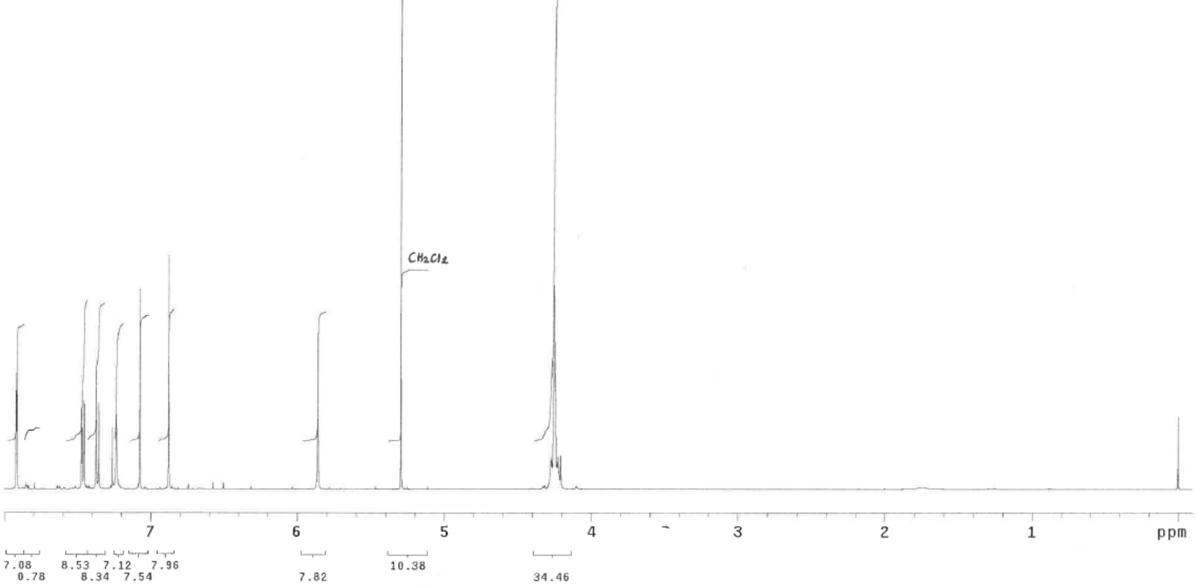

STANDARD 13C
67203
2039-BGE
Berecz Gabor
2007.01.16. (BB)
Solvent: CDCl3
Temp. 25.0 C / 298.1 K
user: 1-14-87
INOVA-500 "i500"
PULSE SEQUENCE
Relax. delay 3.000 sec
Pulse 6.0 usec
Acq. time 1.300 sec
Width 32000.0 Hz
132 repetitions
OBSERVE C13, 125.6055308 MHz
DECOUPLE H1, 499.5269647 MHz
Power 32 dB
continuously on
WALTZ-16 modulated
DATA PROCESSING
Line broadening 0.5 Hz
FT size 131072
Total time 9 minutes

INDEX	FREQUENCY	PPM	HEIGHT
1	18230.964	145.133	22.9
2	18063.484	143.800	33.4
3	17827.644	141.923	25.1
4	17187.019	136.823	27.2
5	16753.914	133.375	29.9
6	16643.074	132.493	72.3
7	16555.183	131.793	59.1
8	16425.789	130.763	33.6
9	16212.410	129.064	75.4
10	15853.523	126.207	27.2
11	15236.824	121.298	61.7
12	14778.816	117.651	70.7
13	9704.597	77.257	158.1
14	9672.371	77.000	161.9
15	9640.632	76.747	158.0
16	8076.668	64.297	76.3
17	8069.832	64.242	69.7
18	7947.761	63.271	45.4
19	6709.480	53.413	18.4

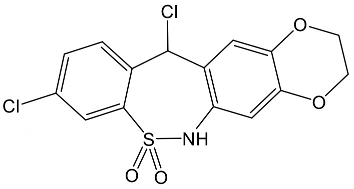

34

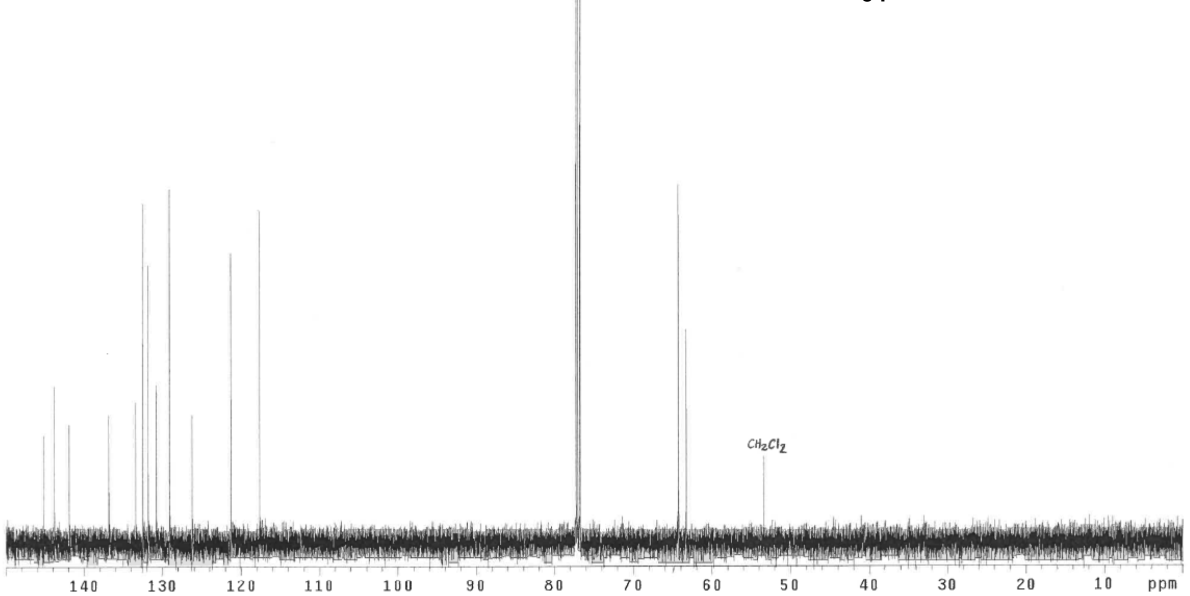

STANDARD 1H
74813
2177-BGE
Berecz Gabor
2008.06.19. (BB)
Solvent: CDCl3
Temp. 30.0 C / 303.1 K
INOVA-500 "i500"
PULSE SEQUENCE
Relax. delay 1.000 sec
Pulse 90.0 degrees
Acq. time 5.000 sec
Width 8000.0 Hz
16 repetitions
OBSERVE H1, 499.5244712 MHz
DATA PROCESSING
FT size 131072
Total time 1 minutes

INDEX	FREQUENCY	PPM	HEIGHT	INDEX	FREQUENCY	PPM	HEIGHT
1	3942.139	7.892	45.2	40	1755.127	3.514	29.8
2	3939.941	7.887	42.9	41	1750.732	3.505	47.4
3	3854.736	7.717	10.2	42	1748.657	3.501	34.6
4	3719.238	7.446	27.4	43	1745.850	3.495	45.1
5	3717.041	7.441	25.2	44	1744.019	3.491	30.1
6	3710.938	7.429	46.7	45	1740.356	3.484	29.2
7	3708.862	7.425	43.8	46	1734.619	3.473	7.1
8	3687.500	7.382	28.9	47	1729.614	3.463	3.4
9	3679.199	7.365	17.7	48	802.612	1.607	5.1
10	3626.343	7.260	16.7	49	600.342	1.202	4.4
11	3500.610	7.008	47.8	50	0.000	0.000	16.3
12	3412.354	6.831	60.4				
13	2640.015	5.285	11.9				
14	2127.197	4.258	3.5				
15	2121.826	4.248	9.1				
16	2119.019	4.242	8.3				
17	2116.333	4.237	16.9				
18	2108.887	4.222	156.5				
19	2107.056	4.218	149.5				
20	2099.609	4.203	17.0				
21	2096.680	4.197	9.2				
22	2093.506	4.191	8.0				
23	2088.745	4.181	3.8				
24	1929.810	3.863	8.7				
25	1924.927	3.854	14.4				
26	1919.312	3.842	28.2				
27	1914.307	3.832	28.0				
28	1913.696	3.831	27.3				
29	1908.813	3.821	20.3				
30	1901.001	3.806	13.2				
31	1895.996	3.796	19.7				
32	1894.531	3.793	17.2				
33	1889.648	3.783	18.6				
34	1885.254	3.774	10.1				
35	1884.033	3.772	9.0				
36	1878.906	3.761	7.5				
37	1765.991	3.535	5.4				
38	1761.230	3.526	6.3				
39	1759.399	3.522	4.5				

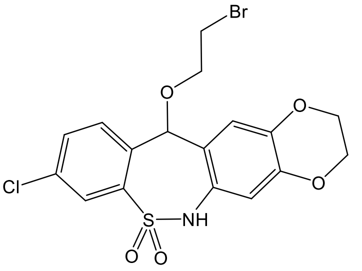

35

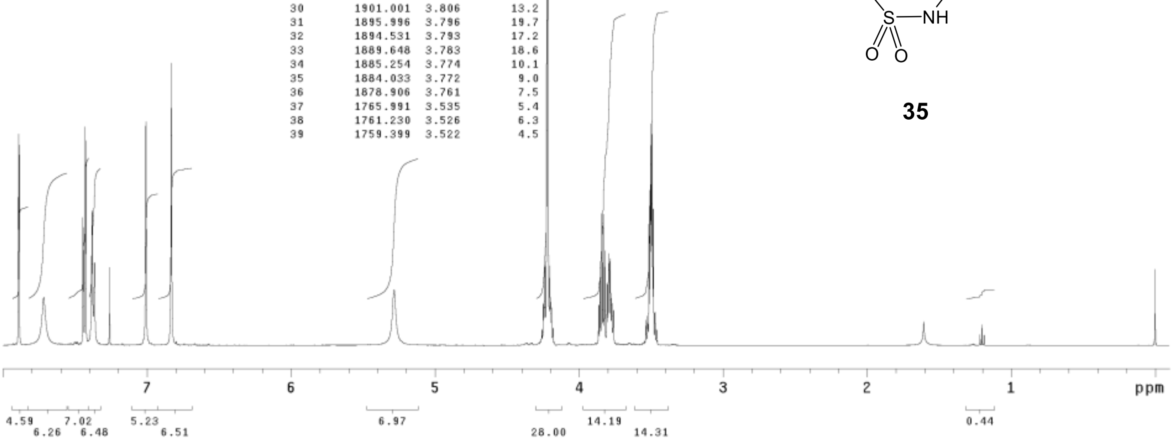

STANDARD 13C
74813
2177-BGE
Berecz Gabor
2008.06.19. (BB)
Solvent: CDCl3
Temp. 30.0 C / 303.1 K
User: 1-14-87
INOVA-500 "i500"
PULSE SEQUENCE
Relax. delay 3.000 sec
Pulse 90.0 degrees
Acq. time 1.300 sec
Width 32000.0 Hz
1658 repetitions
OBSERVE C13, 125.6055303 MHz
DECOUPLE H1, 499.5269647 MHz
Power 34 dB
continuously on
WALTZ-16 modulated
DATA PROCESSING
Line broadening 0.5 Hz
FT size 131072
Total time 118 minutes

INDEX	FREQUENCY	PPM	HEIGHT
1	18119.148	144.243	34.9
2	18031.746	143.547	39.2
3	17853.523	142.129	4.7
4	17114.265	136.244	14.3
5	16803.718	133.771	12.9
6	16583.503	132.018	115.2
7	16470.710	131.120	10.8
8	16404.304	130.592	12.0
9	16208.503	129.033	33.3
10	15807.136	125.838	29.1
11	15080.574	120.054	9.8
12	14684.578	116.901	23.6
13	11900.886	94.741	-4.3
14	10510.261	83.670	4.5
15	9704.597	77.257	86.6
16	9672.371	77.000	91.2
17	9640.632	76.747	89.4
18	8701.667	69.272	78.2
19	8078.132	64.309	156.3
20	8062.996	64.188	146.9
21	3832.527	30.510	115.8

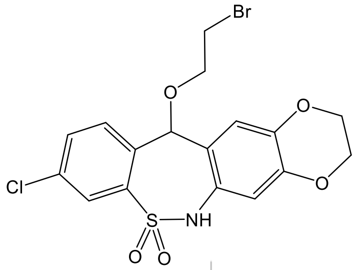

35

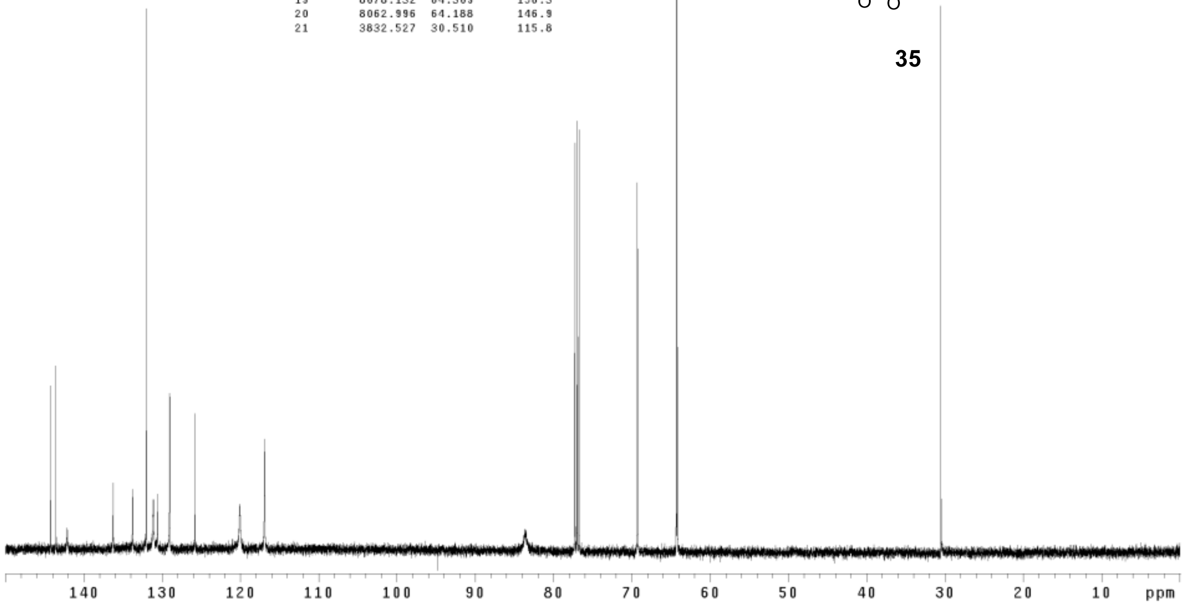

STANDARD 1H
65227
2000-BGE
Berecz Gabor
2006.09.06. (SzA)

Solvent: DMSO
Temp. 25.0 C / 298.1 K
INOVA-500 "i500"

PULSE SEQUENCE
Relax. delay 3.000 sec
Pulse 6.4 usec
Acq. time 5.000 sec
Width 8000.0 Hz
16 repetitions
OBSERVE H1, 499.5268346 MHz
DATA PROCESSING
FT size 131072
Total time 2 minutes

INDEX	FREQUENCY	PPM	HEIGHT
1	3883.667	7.775	27.7
2	3881.470	7.770	30.4
3	3841.187	7.690	17.8
4	3838.867	7.685	15.7
5	3832.886	7.673	27.7
6	3830.566	7.668	25.9
7	3804.688	7.617	30.6
8	3796.265	7.600	19.4
9	3496.460	7.000	27.5
10	3487.793	6.982	42.8
11	3458.862	6.924	62.0
12	3450.195	6.907	41.5
13	3106.201	6.218	25.0
14	3101.929	6.210	30.2
15	3071.655	6.149	21.6
16	3067.383	6.141	17.4
17	2171.387	4.347	6.5
18	2168.579	4.341	7.6
19	2166.870	4.338	10.2
20	2164.063	4.332	11.4
21	2161.377	4.327	8.6
22	2159.302	4.323	5.5
23	2158.203	4.320	7.6
24	2155.884	4.316	9.3
25	2147.461	4.299	6.0
26	2144.043	4.292	6.4
27	2135.498	4.275	10.3
28	2133.667	4.271	6.3
29	2132.568	4.269	8.4
30	2130.859	4.266	10.5
31	2129.395	4.263	10.5
32	2128.540	4.261	10.3
33	2125.977	4.256	9.3
34	2124.023	4.252	7.0
35	2120.483	4.245	5.8
36	1701.294	3.406	184.7
37	1662.354	3.328	81.2
38	1250.732	2.504	7.6
39	1248.901	2.500	10.6
40	1247.070	2.497	7.3
41	1035.278	2.073	5.6
42	519.653	1.040	7.4
43	513.672	1.028	7.1
44	0.000	0.000	14.2

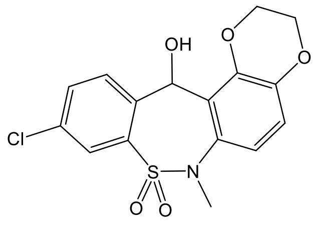

36

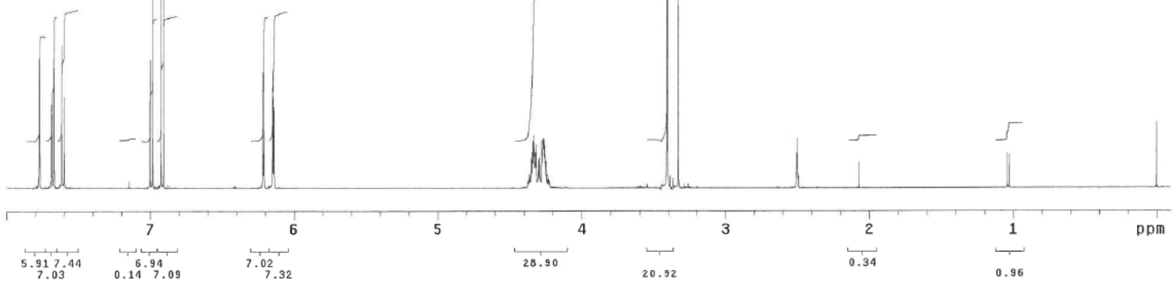

STANDARD 13C
65227
2000-BGE
Berecz Gabor
2006.09.06. (SzA)

Solvent: DMSO
Temp. 25.0 C / 298.1 K
User: 1-14-87
INOVA-500 "i500"

PULSE SEQUENCE
Relax. delay 1.200 sec
Pulse 6.0 usec
Acq. time 1.300 sec
Width 32000.0 Hz
368 repetitions
OBSERVE C13, 125.6061592 MHz
DECOUPLE H1, 499.5293374 MHz
Power 32 dB
continuously on
WALTZ-16 modulated
DATA PROCESSING
Line broadening 1.0 Hz
FT size 131072
Total time 15 minutes

INDEX	FREQUENCY	PPM	HEIGHT
1	18025.337	143.501	27.6
2	17947.212	142.879	23.0
3	17682.563	140.772	28.2
4	17168.891	136.683	30.9
5	16875.923	134.351	33.5
6	16818.794	133.896	26.1
7	16600.532	132.158	40.1
8	16518.012	131.501	28.8
9	16351.997	130.180	22.9
10	15943.305	126.926	41.5
11	15401.313	122.611	41.1
12	14773.872	117.616	41.1
13	8389.106	66.786	45.3
14	8103.462	64.512	36.9
15	8025.337	63.890	34.2
16	5115.180	40.722	44.9
17	5050.239	40.205	23.5
18	5029.243	40.038	68.9
19	5007.759	39.867	137.5
20	4986.762	39.700	162.0
21	4965.766	39.533	137.6
22	4944.770	39.366	69.2
23	4923.774	39.199	23.1

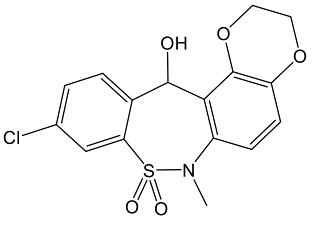

36

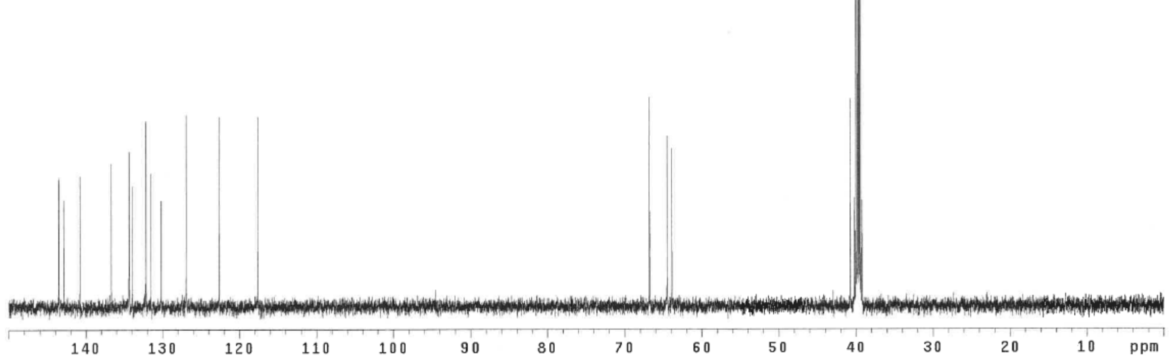

STANDARD 1H
62897
1951-BGE
Berecz Gabor
2006.04.06. (BT)
 Solvent: CDCl3
 Temp. 25.0 C / 298.1 K
INOVA-500 "i500"
PULSE SEQUENCE
 Relax. delay 3.000 sec
 Pulse 6.4 usec
 Acq. time 5.000 sec
 Width 8000.0 Hz
 16 repetitions
OBSERVE H1, 499.5244718 MHz
DATA PROCESSING
FT size 131072
Total time 2 minutes

INDEX	FREQUENCY	PPM	HEIGHT
1	3981.079	7.970	13.3
2	3980.835	7.969	14.5
3	3979.980	7.968	23.8
4	3978.638	7.965	13.7
5	3734.131	7.475	77.8
6	3732.788	7.473	62.0
7	3625.122	7.257	12.0
8	3503.296	7.013	18.6
9	3494.629	6.996	41.9
10	3481.812	6.970	61.4
11	3473.145	6.953	28.2
12	3354.980	6.716	46.9
13	2642.212	5.289	73.4
14	2186.035	4.376	5.9
15	2183.105	4.370	8.0
16	2180.664	4.365	9.1
17	2177.368	4.359	13.0
18	2172.607	4.349	6.8
19	2168.335	4.341	6.7
20	2165.894	4.336	8.7
21	2162.964	4.330	10.6
22	2160.645	4.325	12.4
23	2152.222	4.309	23.2
24	2143.677	4.291	12.3
25	2141.235	4.287	9.9
26	2138.550	4.281	8.5
27	2135.986	4.276	6.1
28	2126.953	4.258	6.2
29	2122.314	4.249	13.3
30	2118.896	4.242	8.6
31	2116.455	4.237	7.5
32	2113.403	4.231	6.2
33	2107.422	4.219	4.7
34	1769.287	3.542	151.2
35	-0.000	-0.000	13.5

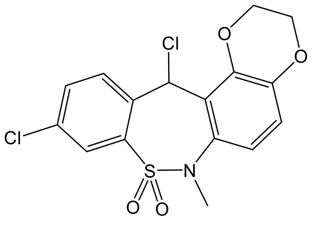

37

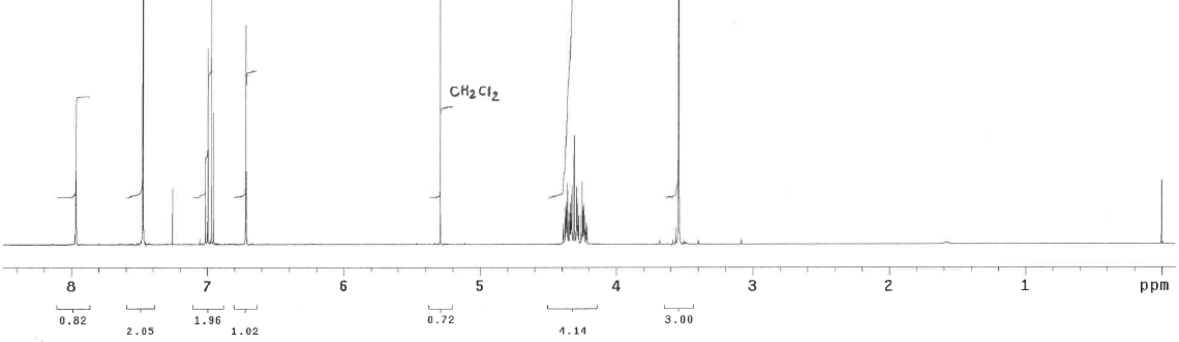

STANDARD 13C
62897
1951-BGE
Berecz Gabor
2006.04.06. (BT)
 Solvent: CDCl3
 Temp. 25.0 C / 298.1 K
User: 1-14-87
INOVA-500 "i500"
PULSE SEQUENCE
 Relax. delay 3.000 sec
 Pulse 6.0 usec
 Acq. time 1.300 sec
 Width 32000.0 Hz
 272 repetitions
OBSERVE C13, 125.6055332 MHz
DECOUPLE H1, 499.5269647 MHz
 Power 32 dB
 continuously on
 WALTZ-16 modulated
DATA PROCESSING
 Line broadening 1.0 Hz
FT size 131072
Total time 19 minutes

INDEX	FREQUENCY	PPM	HEIGHT
1	18020.027	143.454	35.5
2	17863.289	142.206	25.8
3	17649.422	140.504	26.9
4	17099.617	136.127	38.9
5	16760.750	133.429	78.3
6	16731.453	133.196	37.3
7	16672.371	132.726	29.7
8	16612.312	132.248	81.8
9	16062.019	127.867	80.5
10	15843.758	126.129	15.4
11	15264.656	121.519	85.7
12	14991.218	119.342	89.0
13	9704.109	77.253	104.8
14	9672.371	77.000	109.1
15	9640.144	76.743	105.2
16	8119.636	64.639	81.5
17	8013.191	63.792	82.8
18	6731.941	53.592	59.8
19	6708.504	53.405	9.6 ← DCM
20	4954.109	39.439	61.8

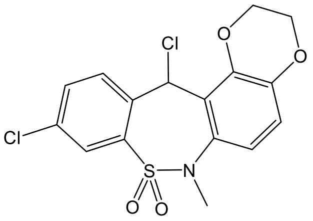

37

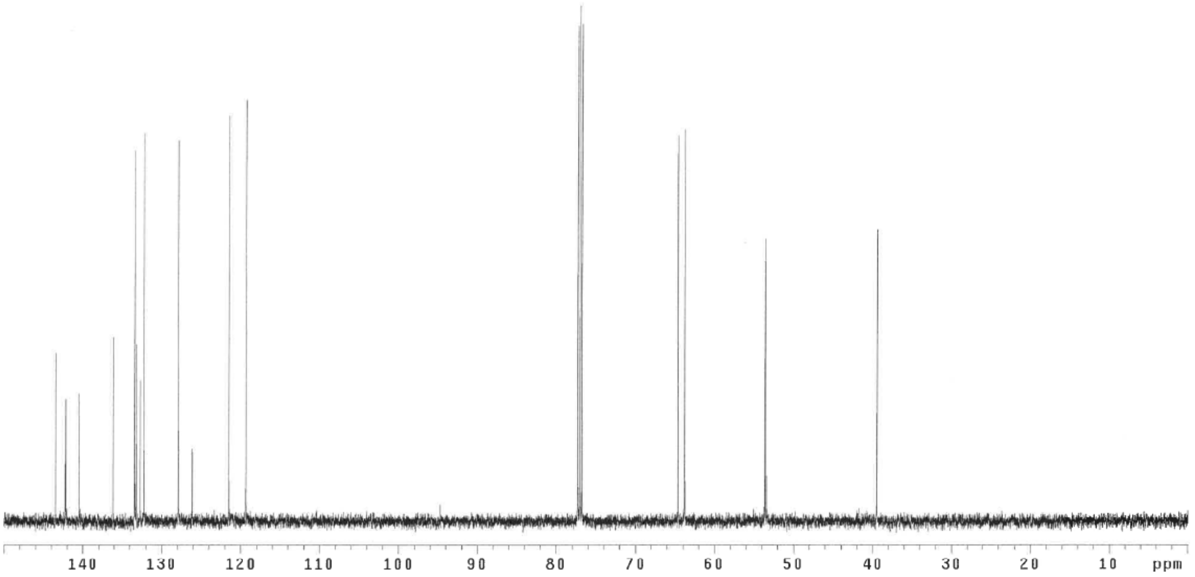

STANDARD 1H
62898
1952-BGE
Berecz Gabor
2006.04.06. (BT)
 Solvent: CDCl3
 Temp. 25.0 C / 298.1 K
INOVA-500 "i500"
PULSE SEQUENCE
 Relax. delay 3.000 sec
 Pulse 6.4 usec
 Acq. time 5.000 sec
 Width 8000.0 Hz
 16 repetitions
OBSERVE H1, 499.5244699 MHz
DATA PROCESSING
FT size 131072
Total time 2 minutes

INDEX	FREQUENCY	PPM	HEIGHT
1	3958.374	7.924	23.9
2	3675.049	7.357	64.9
3	3673.950	7.355	56.9
4	3627.197	7.261	8.4
5	3626.831	7.261	8.0
6	3473.999	6.955	24.4
7	3465.454	6.938	32.8
8	3421.997	6.851	34.5
9	3413.330	6.833	25.0
10	2434.082	4.873	38.9
11	2133.179	4.270	7.7
12	2130.615	4.265	15.7
13	2126.221	4.256	42.2
14	2124.878	4.254	40.6
15	2119.751	4.244	2.7
16	2115.967	4.236	20.1
17	2113.525	4.231	10.5
18	2112.061	4.228	7.5
19	2108.887	4.222	8.2
20	2104.370	4.213	8.0
21	2102.539	4.209	11.1
22	2098.877	4.202	6.9
23	2097.046	4.198	6.9
24	2094.116	4.192	4.9
25	2089.722	4.183	2.9
26	2087.036	4.178	3.0
27	1740.845	3.485	163.7
28	1730.225	3.464	3.0
29	1143.555	2.289	9.5
30	1141.479	2.285	9.9
31	1139.404	2.281	9.0
32	1136.719	2.276	11.1
33	1134.766	2.272	14.2
34	1130.005	2.262	6.0
35	1107.544	2.217	6.1
36	1102.783	2.208	14.4
37	1100.952	2.204	11.3
38	1098.145	2.198	9.3
39	1096.313	2.195	10.0
40	1093.994	2.190	9.3
41	841.553	1.685	16.8
42	835.083	1.672	45.8
43	828.857	1.659	14.1
44	801.514	1.605	5.9
45	567.505	1.136	2.8
46	561.401	1.124	2.8
47	-0.000	-0.000	8.7
48	-0.244	-0.000	8.1

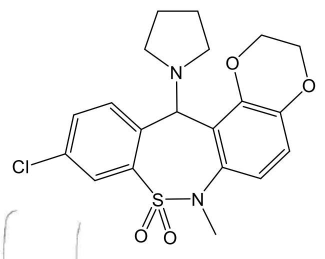

38a

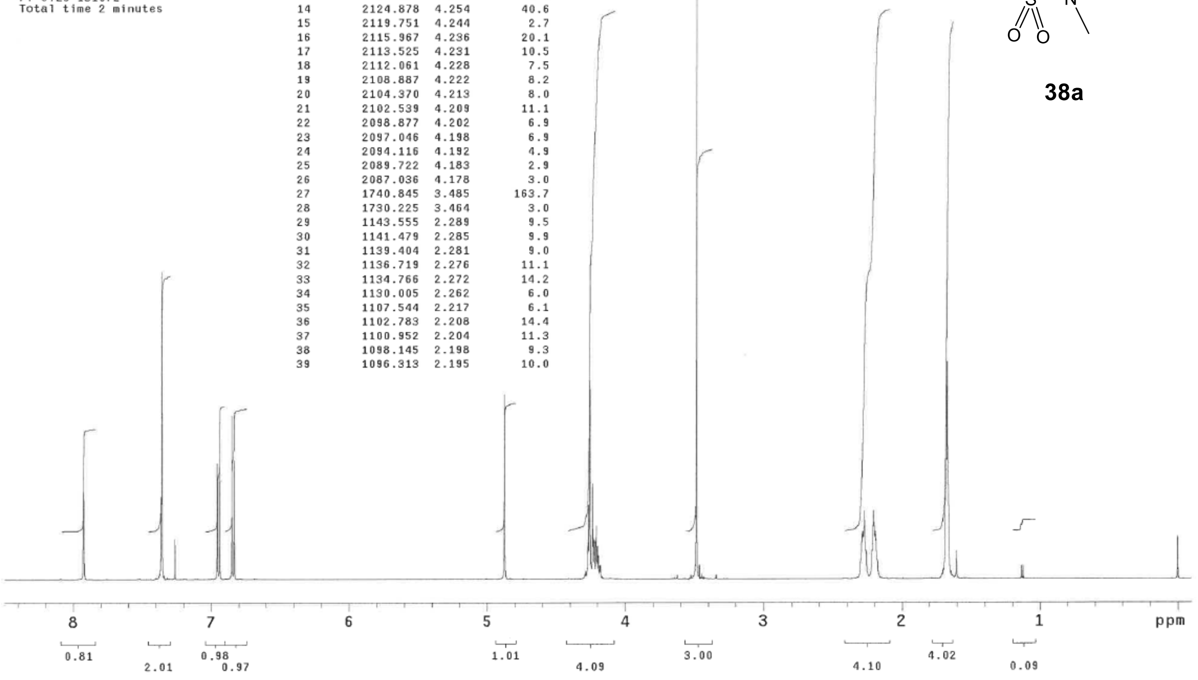

STANDARD 13C
62898
1952-BGE
Berecz Gabor
2006.04.06. (BT)
 Solvent: CDCl3
 Temp. 25.0 C / 298.1 K
User: 1-14-87
INOVA-500 "i500"
PULSE SEQUENCE
 Relax. delay 3.000 sec
 Pulse 6.0 usec
 Acq. time 1.300 sec
 Width 32000.0 Hz
 256 repetitions
OBSERVE C13, 125.6055323 MHz
DECOUPLE H1, 499.5269647 MHz
 Power 32 dB
 continuously on
 WALTZ-16 modulated
DATA PROCESSING
 Line broadening 1.0 Hz
FT size 131072
Total time 18 minutes

INDEX	FREQUENCY	PPM	HEIGHT
1	18063.484	143.800	21.2
2	18033.211	143.559	26.7
3	17748.054	141.289	27.2
4	16979.011	135.167	32.6
5	16941.414	134.868	29.4
6	16785.164	133.624	56.8
7	16506.355	131.404	22.8
8	16444.832	130.914	59.0
9	16322.273	129.939	26.6
10	16094.246	128.123	59.5
11	15443.367	122.942	65.6
12	14783.699	117.690	64.9
13	9704.597	77.257	96.2
14	9672.371	77.000	99.2
15	9640.632	76.747	96.3
16	8132.820	64.744	57.7
17	8064.461	64.200	60.6
18	8028.328	63.912	59.9
19	6687.996	53.242	116.7
20	5033.211	40.068	43.6
21	2910.652	23.171	123.4

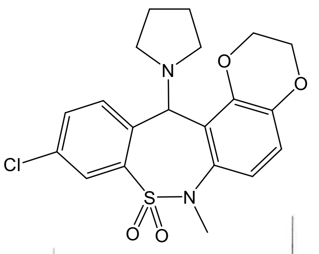

38a

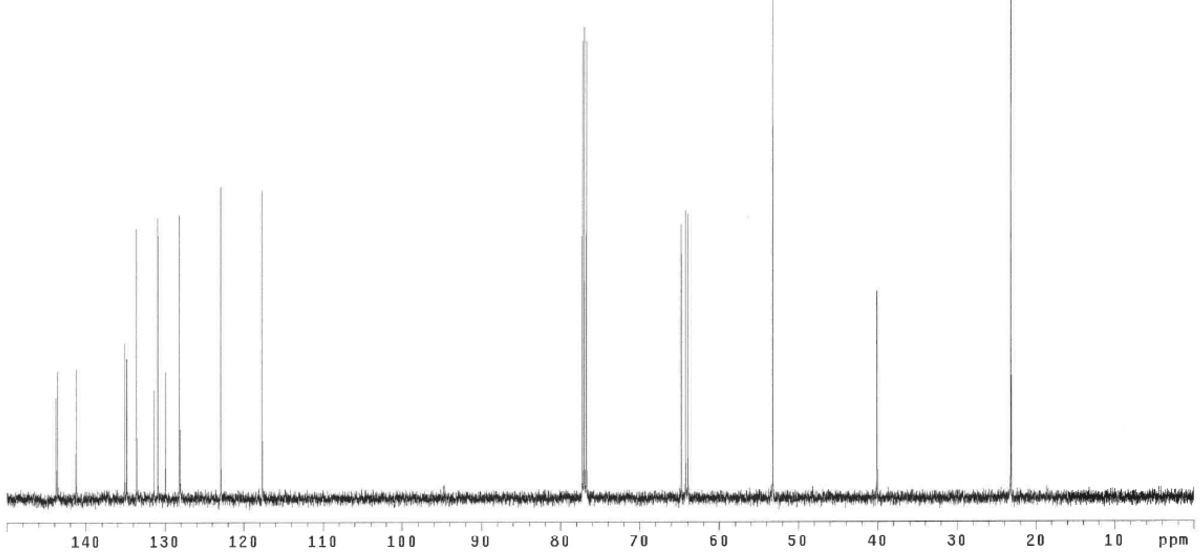

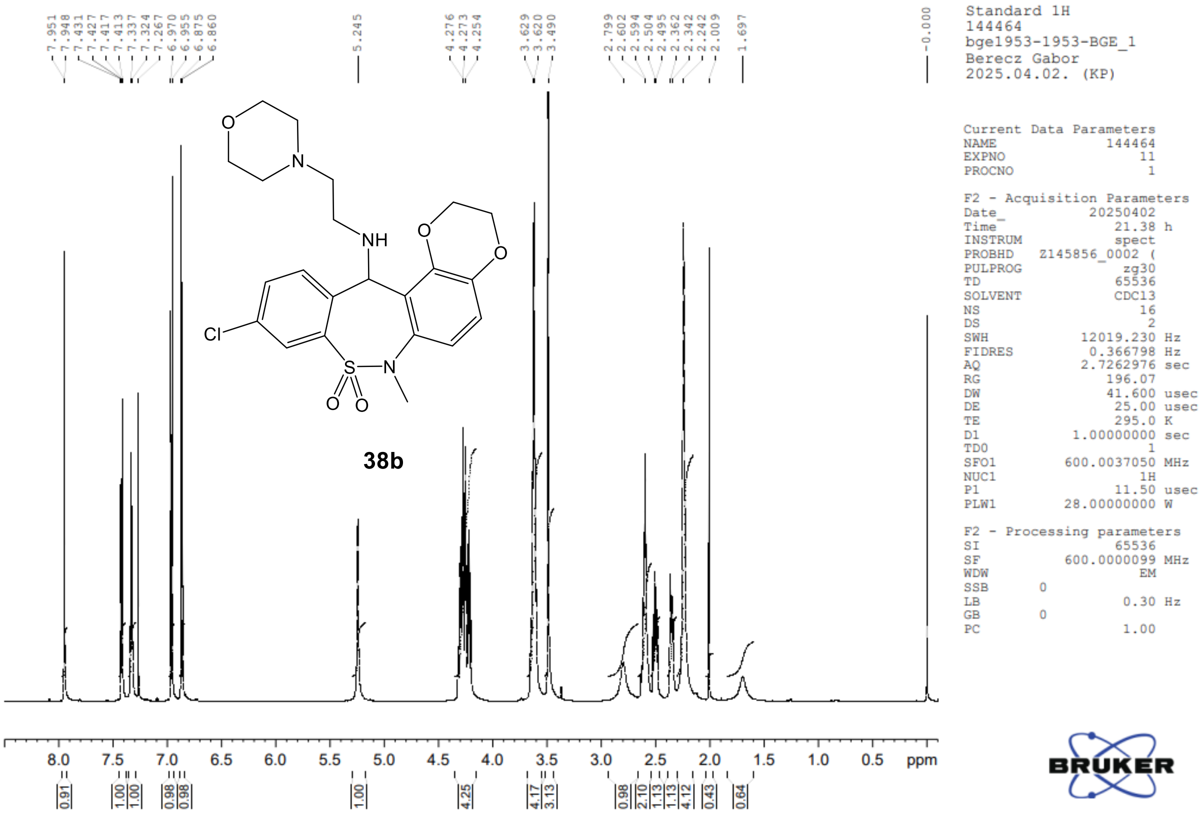
38b
Standard 1H
144464
bge1953-1953-BGE_1
Berecz Gabor
2025.04.02. (KP)
Current Data Parameters
NAME 144464
EXPNO 11
PROCNO 1
F2 - Acquisition Parameters
Date_ 20250402
Time 21.38 h
INSTRUM spect
PROBHD Z145856_0002 (
PULPROG zg30
TD 65536
SOLVENT CDCl3
NS 16
DS 2
SWH 12019.230 Hz
FIDRES 0.366798 Hz
AQ 2.7262976 sec
RG 196.07
DW 41.600 usec
DE 25.00 usec
TE 295.0 K
D1 1.00000000 sec
TD0 1
SFO1 600.0037050 MHz
NUC1 1H
P1 11.50 usec
PLW1 28.00000000 W
F2 - Processing parameters
SI 65536
SF 600.0000099 MHz
WDW EM
SSB 0
LB 0.30 Hz
GB 0
PC 1.00
ppm

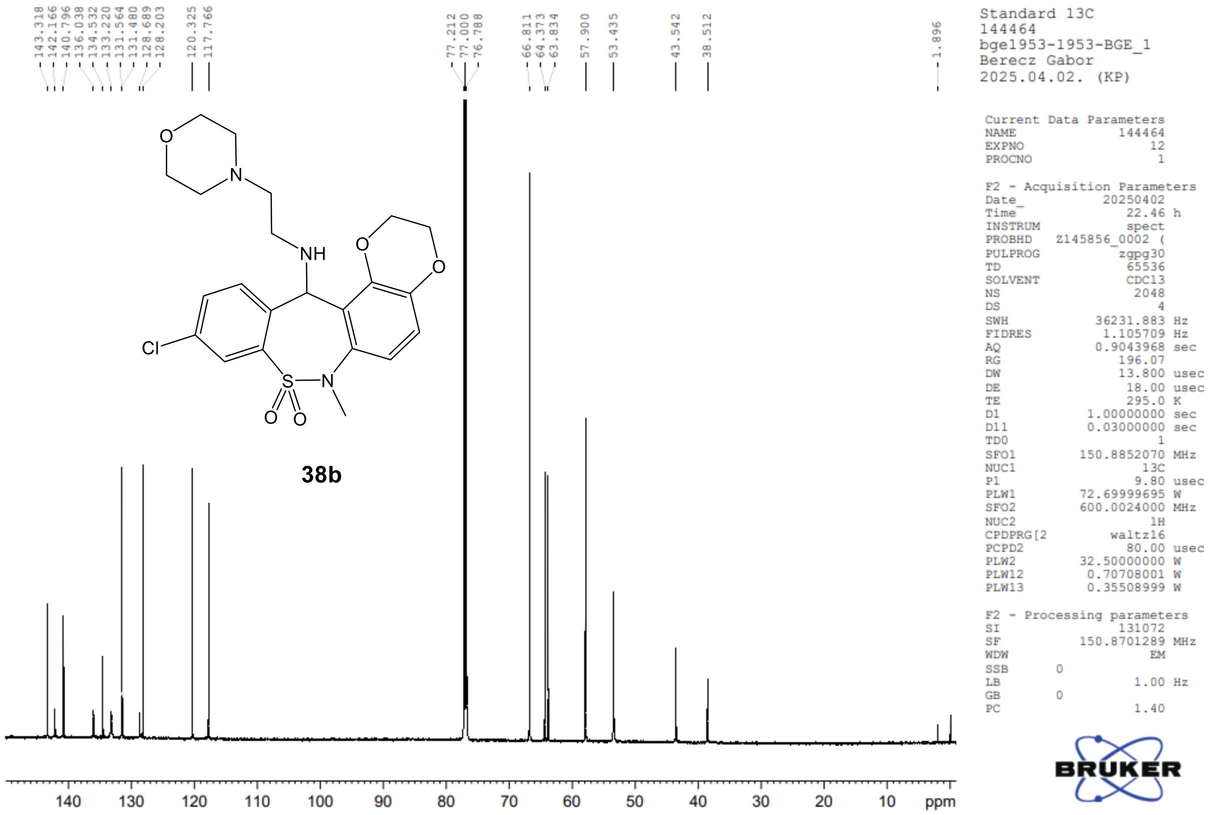
38b
Standard 13C
144464
bge1953-1953-BGE_1
Berecz Gabor
2025.04.02. (KP)
Current Data Parameters
NAME 144464
EXPNO 12
PROCNO 1
F2 - Acquisition Parameters
Date_ 20250402
Time 22.46 h
INSTRUM spect
PROBHD Z145856_0002 (
PULPROG zgpg30
TD 65536
SOLVENT CDCl3
NS 2048
DS 4
SWH 36231.883 Hz
FIDRES 1.105709 Hz
AQ 0.9043968 sec
RG 196.07
DW 13.800 usec
DE 18.00 usec
TE 295.0 K
D1 1.00000000 sec
D11 0.03000000 sec
TD0 1
SFO1 150.8852070 MHz
NUC1 13C
P1 9.80 usec
PLW1 72.69999695 W
SFO2 600.0024000 MHz
NUC2 1H
CPDPRG[2 waltz16
PCPD2 80.00 usec
PLW2 32.50000000 W
PLW12 0.70708001 W
PLW13 0.35508999 W
F2 - Processing parameters
SI 131072
SF 150.8701289 MHz
WDW EM
SSB 0
LB 1.00 Hz
GB 0
PC 1.40
ppm

38b

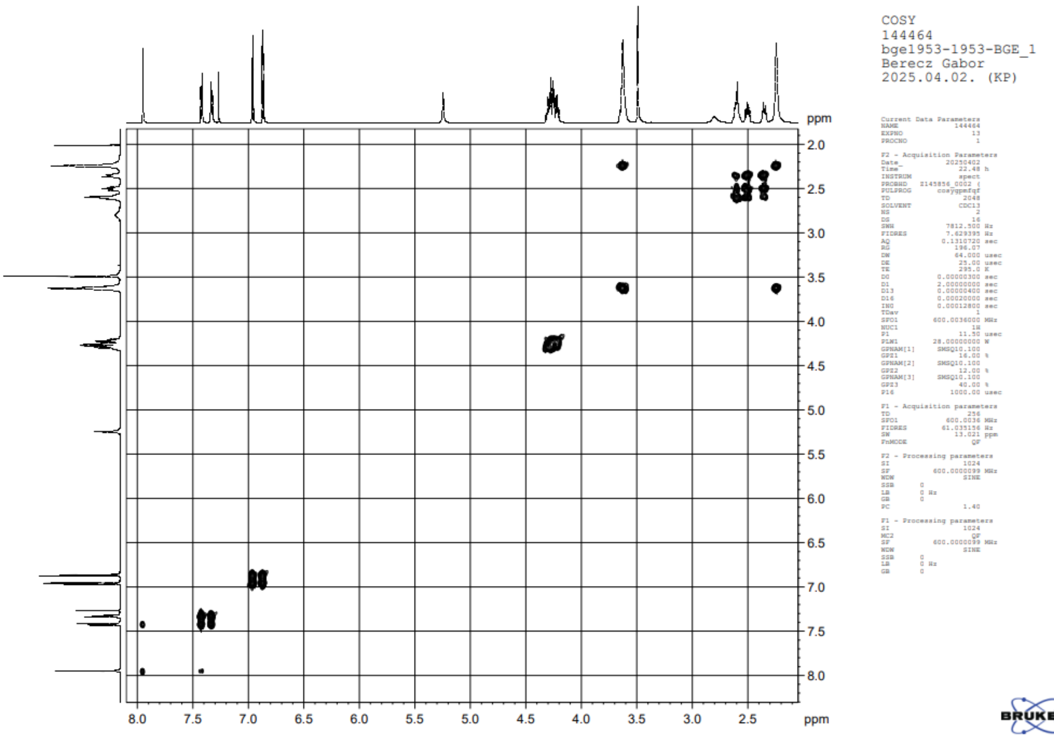
COSY
144464
bge1953-1953-BGE_1
Berecz Gabor
2025.04.02. (KP)
ppm
2.0
2.5
3.0
3.5
4.0
4.5
5.0
5.5
6.0
6.5
7.0
7.5
8.0
8.0 7.5 7.0 6.5 6.0 5.5 5.0 4.5 4.0 3.5 3.0 2.5 ppm
BRUKER

38b

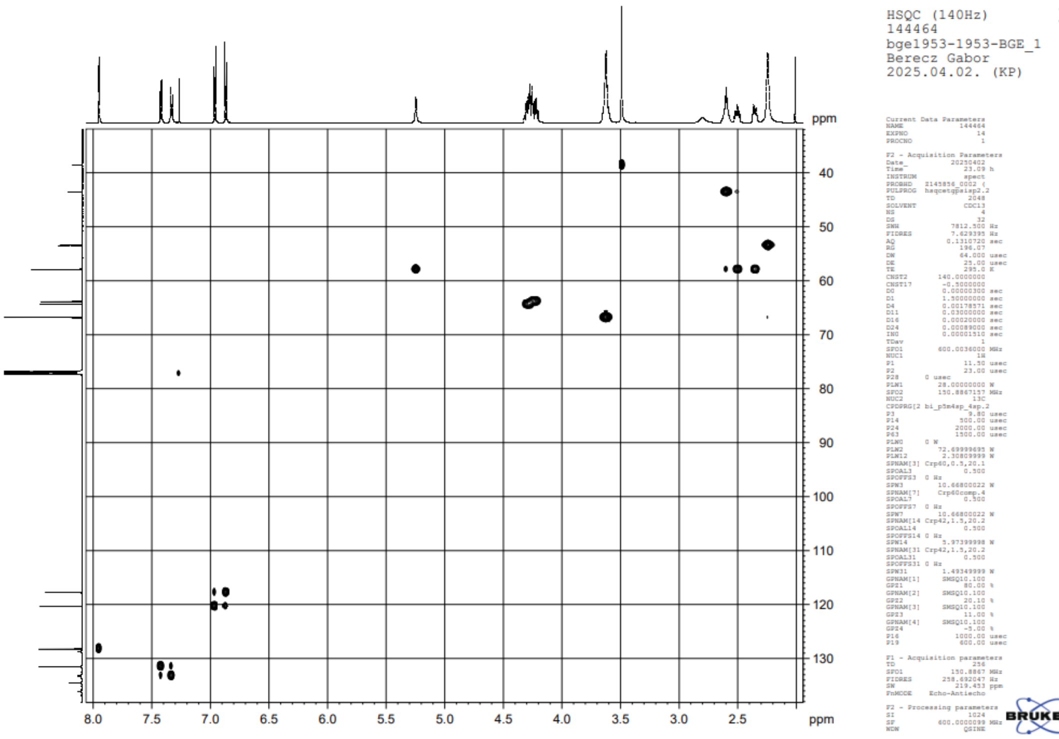
HSQC (140Hz)
144464
bge1953-1953-BGE_1
Berecz Gabor
2025.04.02. (KP)
ppm
40
50
60
70
80
90
100
110
120
130
8.0 7.5 7.0 6.5 6.0 5.5 5.0 4.5 4.0 3.5 3.0 2.5 ppm
BRUKER

38b

HMBC (8Hz, 140Hz)
144464
bge1953-1953-BGE_1
Berecz Gabor
2025.04.02. (KP)

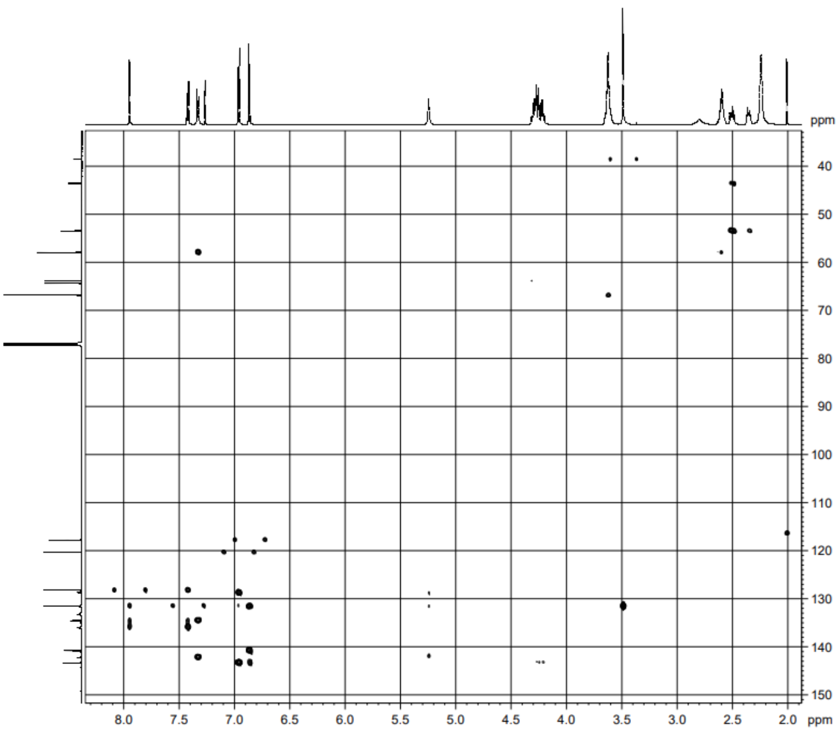


```
Current Data Parameters
NAME             144464
EXPNO                15
PROCNO                1

F2 - Acquisition Parameters
Date_          20250402
Time              23.38 h
INSTRUM           spect
PROBHD   Z145856_0002 (
PULPROG    hmbcgplpndqf
TD                 2048
SOLVENT           CDCl3
NS                    4
DS                   16
SWH            7812.500 Hz
FIDRES         7.629395 Hz
AQ            0.1310720 sec
RG               196.07
DW               64.000 usec
DE                25.00 usec
TE                295.0 K
CNST2       140.0000000
CNST13        8.0000000
D0           0.00000300 sec
D1           1.50000000 sec
D2           0.00357143 sec
D6           0.06250000 sec
D16          0.00020000 sec
IN0          0.00001510 sec
TDav                  1
SFO1        600.0037800 MHz
NUC1                 1H
P1                11.50 usec
P2                23.00 usec
PLW1        28.00000000 W
SFO2        150.8867157 MHz
NUC2                13C
P3                 9.80 usec
PLW2        72.69999695 W
GPNAM[1]     SMSQ10.100
GPZ1              50.00 %
GPNAM[2]     SMSQ10.100
GPZ2              30.00 %
GPNAM[3]     SMSQ10.100
GPZ3              40.10 %
P16             1000.00 usec

F1 - Acquisition parameters
TD                  256
SFO1           150.8867 MHz
FIDRES       258.692047 Hz
SW              219.453 ppm
FnMODE               QF

F2 - Processing parameters
SI                 2048
SF          600.0000099 MHz
WDW                SINE
SSB      0
LB       0 Hz
GB       0
PC                 1.40

F1 - Processing parameters
SI                 1024
MC2                  QF
SF          150.8701289 MHz
WDW                SINE
SSB      0
LB       0 Hz
GB       0
```

STANDARD 1H
63034
1954-BGE
Berecz Gabor
2006.04.13. (CzB)

Solvent: CDCl3
Temp. 25.0 C / 298.1 K
INOVA-500 "i500"

PULSE SEQUENCE
Relax. delay 1.000 sec
Pulse 6.4 usec
Acq. time 5.000 sec
Width 8000.0 Hz
16 repetitions
OBSERVE H1, 499.5244656 MHz
DATA PROCESSING
FT size 131072
Total time 1 minutes

INDEX	FREQUENCY	PPM	HEIGHT	INDEX	FREQUENCY	PPM	HEIGHT	INDEX	FREQUENCY	PPM	HEIGHT
1	3966.797	7.941	21.6	40	2095.459	4.195	2.5	79	645.874	1.293	2.9
2	3964.600	7.937	21.5	41	2090.454	4.185	0.6	80	640.991	1.283	16.9
3	3714.478	7.436	14.1	42	2074.707	4.153	0.6	81	638.672	1.279	17.5
4	3712.280	7.432	13.4	43	2067.627	4.139	0.6	82	637.085	1.275	20.8
5	3706.177	7.419	22.2	44	2061.768	4.127	15.4	83	633.911	1.269	35.7
6	3703.979	7.415	22.1	45	2054.565	4.113	47.6	84	630.493	1.262	19.3
7	3683.960	7.375	0.9	46	2047.485	4.099	48.2	85	625.854	1.253	66.6
8	3678.345	7.364	15.4	47	2040.283	4.084	15.8	86	618.652	1.238	114.8
9	3670.044	7.347	10.0	48	2034.546	4.073	0.6	87	611.572	1.224	56.4
10	3631.226	7.269	11.1	49	1767.456	3.538	0.7	88	608.276	1.218	1.3
11	3467.041	6.941	21.5	50	1718.018	3.439	1.6	89	605.835	1.213	1.1
12	3463.379	6.933	0.6	51	1697.876	3.399	151.7	90	602.051	1.205	0.9
13	3458.374	6.923	34.4	52	1677.856	3.359	1.4	91	598.633	1.198	1.4
14	3451.782	6.910	0.7	53	1625.854	3.255	0.8	92	591.553	1.184	0.7
15	3431.641	6.870	44.4	54	1247.437	2.497	0.7	93	554.443	1.110	0.7
16	3428.223	6.863	0.8	55	1235.962	2.474	7.9	94	0.000	0.000	11.1
17	3426.392	6.859	0.7	56	1228.882	2.460	15.6				
18	3422.974	6.852	27.6	57	1221.680	2.446	8.9				
19	2648.804	5.303	13.3	58	1209.106	2.421	0.8				
20	2645.264	5.296	4.6	59	1133.301	2.269	22.5				
21	2644.287	5.294	6.5	60	1125.977	2.254	40.1				
22	2157.349	4.319	2.9	61	1118.286	2.239	25.5				
23	2154.785	4.314	0.9	62	1013.062	2.028	1.4				
24	2151.733	4.308	4.0	63	803.467	1.608	2.4				
25	2148.682	4.301	6.7	64	796.021	1.594	8.6				
26	2143.311	4.291	9.9	65	793.701	1.589	6.3				
27	2139.038	4.282	15.4	66	788.696	1.579	12.3				
28	2134.644	4.273	24.0	67	783.691	1.569	6.9				
29	2130.737	4.266	2.1	68	781.372	1.564	8.6				
30	2129.395	4.263	1.9	69	773.926	1.549	2.7				
31	2125.732	4.256	27.0	70	741.699	1.485	0.8				
32	2121.094	4.246	6.1	71	728.516	1.458	4.6				
33	2119.751	4.244	5.9	72	721.924	1.445	7.9				
34	2115.723	4.235	6.1	73	715.942	1.433	8.0				
35	2111.084	4.226	13.7	74	709.717	1.421	5.0				
36	2106.323	4.217	7.6	75	703.247	1.408	2.4				
37	2105.469	4.215	7.4	76	696.289	1.394	1.1				
38	2102.295	4.209	6.3	77	688.477	1.378	0.7				
39	2097.046	4.198	4.5	78	681.274	1.364	0.8				

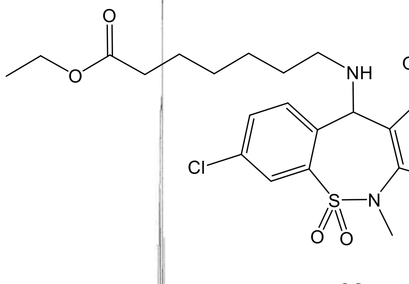

38c

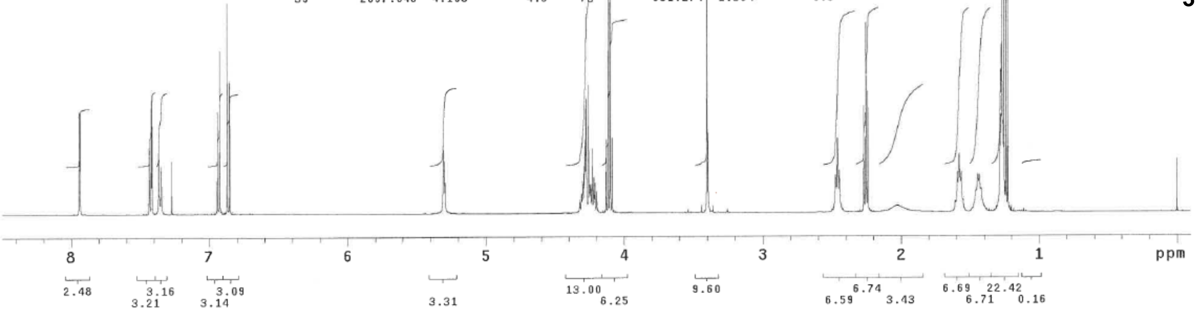

STANDARD 13C
63034
1954-BGE
Berecz Gabor
2006.04.13. (CzB)

Solvent: CDCl3
Temp. 25.0 C / 298.1 K
User: 1-14-87
INOVA-500 "i500"

PULSE SEQUENCE
Relax. delay 3.000 sec
Pulse 6.0 usec
Acq. time 1.300 sec
Width 32000.0 Hz
368 repetitions
OBSERVE C13, 125.6055337 MHz
DECOUPLE H1, 499.5269647 MHz
Power 32 dB
continuously on
WALTZ-16 modulated
DATA PROCESSING
Line broadening 0.5 Hz
Sq. sine bell 1.092 sec
Shifted by -0.884 sec
FT size 131072
Total time 26 minutes

INDEX	FREQUENCY	PPM	HEIGHT
1	21812.508	173.645	40.0
2	17985.847	143.182	43.7
3	17825.691	141.907	7.2
4	17741.219	141.235	44.2
5	17100.105	136.131	4.9
6	16883.308	134.405	24.6
7	16728.035	133.169	4.6
8	16553.719	131.781	9.1
9	16539.558	131.668	82.6
10	16094.734	128.127	77.8
11	15128.426	120.435	73.5
12	14781.258	117.671	70.0
13	9704.597	77.257	125.9
14	9672.371	77.000	131.3
15	9640.633	76.747	130.0
16	8080.086	64.324	73.9
17	8018.074	63.830	70.1
18	7550.789	60.110	73.2
19	7312.508	58.214	56.8
20	6002.937	47.788	43.8
21	4863.289	38.716	10.7
22	4292.000	34.168	102.8
23	3737.312	29.752	77.6
24	3628.914	28.889	98.3
25	3375.984	26.876	92.1
26	3110.847	24.765	113.4
27	1782.722	14.192	93.2

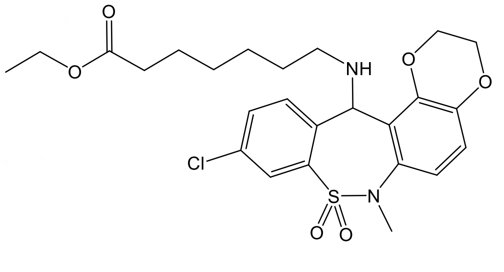

38c

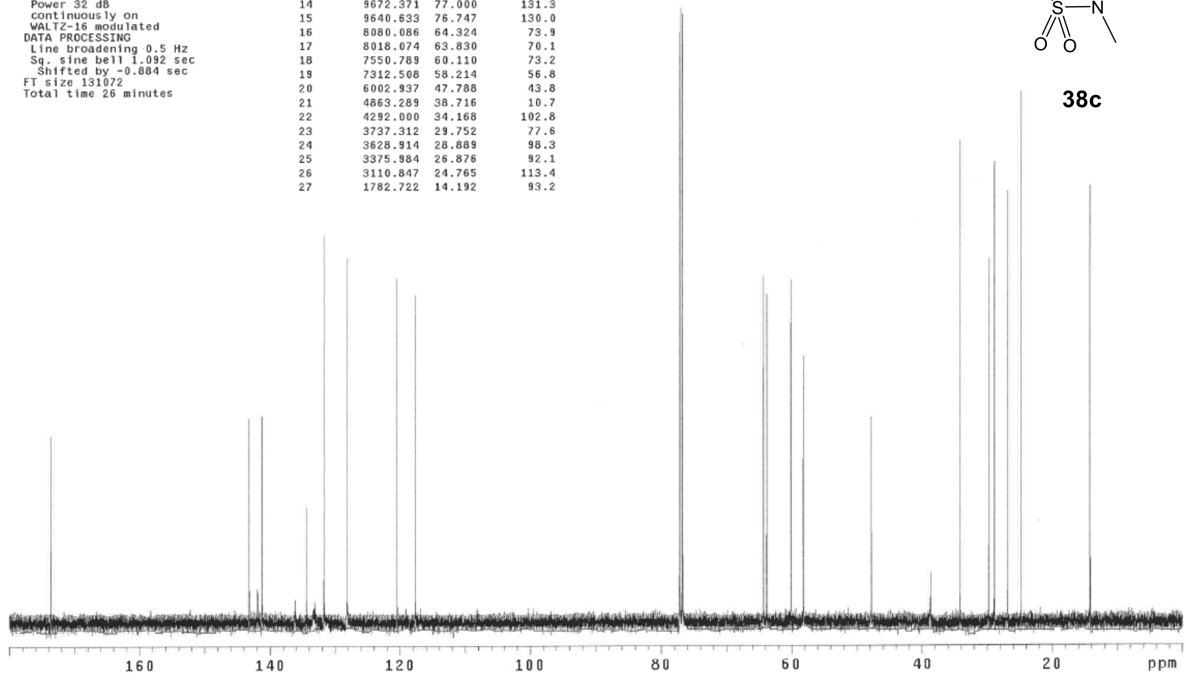

STANDARD 1H
63158
1956-BGE
Berecz Gabor
2006/04/25 (SzA)

Solvent: CDCl3
Temp. 25.0 C / 298.1 K
INOVA-500 "i500"

PULSE SEQUENCE
Relax. delay 3.000 sec
Pulse 6.4 usec
Acq. time 5.000 sec
Width 8000.0 Hz
16 repetitions
OBSERVE H1, 499.5244671 MHz
DATA PROCESSING
FT size 131072
Total time 2 minutes

INDEX	FREQUENCY	PPM	HEIGHT
1	3964.111	7.936	35.4
2	3962.646	7.933	35.1
3	3711.548	7.430	39.4
4	3709.717	7.426	42.2
5	3706.665	7.420	40.2
6	3467.651	6.942	22.6
7	3458.862	6.924	43.5
8	3442.383	6.891	47.7
9	3433.716	6.874	24.8
10	3376.587	6.760	16.2
11	2712.036	5.429	21.2
12	2145.630	4.295	15.4
13	2141.235	4.287	20.5
14	2138.184	4.280	28.0
15	2133.911	4.272	26.3
16	2129.761	4.264	15.7
17	2125.244	4.255	14.6
18	2121.216	4.246	21.8
19	2119.019	4.242	17.9
20	2117.065	4.238	15.9
21	2114.136	4.232	12.6
22	2111.328	4.227	14.4
23	2107.178	4.218	14.8
24	2103.638	4.211	14.6
25	1689.209	3.382	162.0
26	1251.953	2.506	18.4
27	1244.141	2.491	17.5
28	1094.971	2.192	22.8
29	1087.646	2.177	44.9
30	1080.322	2.163	24.4
31	765.503	1.532	16.3
32	758.789	1.519	23.2
33	751.953	1.505	17.6
34	726.196	1.454	20.3
35	619.751	1.241	56.4

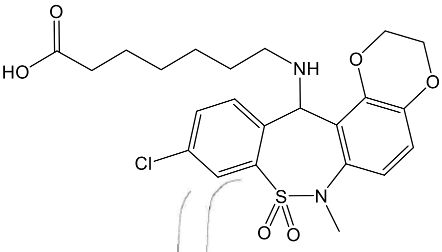

38d

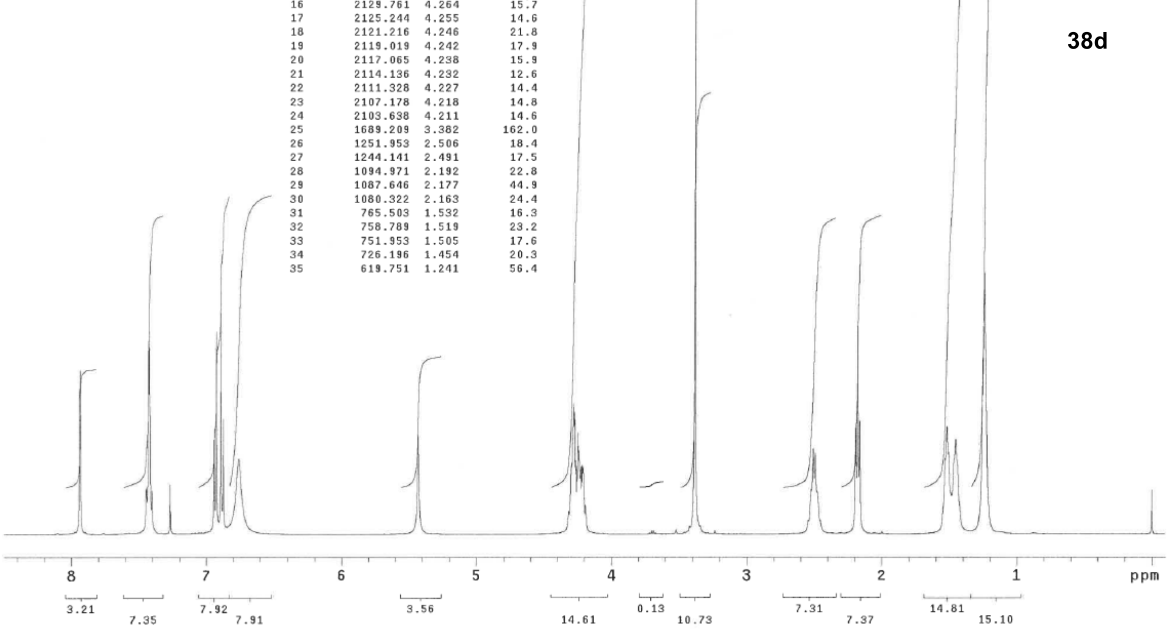

STANDARD 13C
63158
1956-BGE
Berecz Gabor
2006/04/25 (SzA)

Solvent: CDCl3
Temp. 25.0 C / 298.1 K
User: 1-14-87
INOVA-500 "i500"

PULSE SEQUENCE
Relax. delay 3.000 sec
Pulse 6.0 usec
Acq. time 1.300 sec
Width 32000.0 Hz
800 repetitions
OBSERVE C13, 125.6055337 MHz
DECOUPLE H1, 499.5269647 MHz
Power 32 dB
continuously on
WALTZ-16 modulated
DATA PROCESSING
Line broadening 2.0 Hz
FT size 131072
Total time 57 minutes

INDEX	FREQUENCY	PPM	HEIGHT
1	22338.875	177.836	31.6
2	17980.965	143.143	38.8
3	17809.090	141.775	13.2
4	17750.496	141.308	37.4
5	16949.715	134.934	23.3
6	16910.164	134.619	8.7
7	16793.465	133.690	7.7
8	16567.879	131.894	65.0
9	16091.316	128.100	62.3
10	15845.222	126.141	7.1
11	15035.164	119.692	40.0
12	14833.015	118.083	51.0
13	9704.109	77.253	169.8
14	9672.371	77.000	171.1
15	9640.144	76.743	171.0
16	8088.875	64.394	59.7
17	8020.027	63.846	60.7
18	7201.179	57.327	61.0
19	5921.883	47.143	35.7
20	4783.211	38.078	22.8
21	4326.668	34.444	44.1
22	3634.285	28.932	54.5
23	3622.566	28.839	75.5
24	3369.636	26.825	69.7
25	3119.148	24.831	69.7

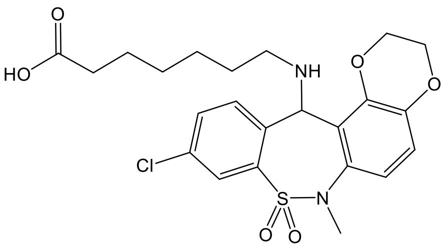

38d

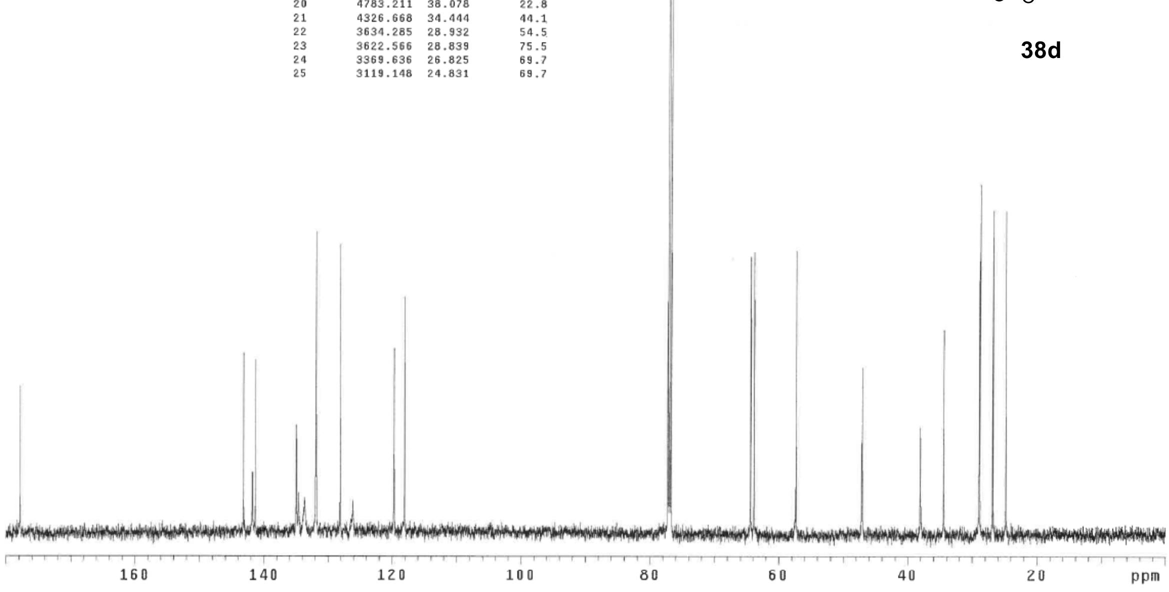

STANDARD 1H
63992
1975-BGE
Berecz Gabor
2006.06.20. (CzB)

Solvent: CDCl3
Temp. 25.0 C / 298.1 K
INOVA-500 "i500"

PULSE SEQUENCE
Relax. delay 1.000 sec
Pulse 6.4 usec
Acq. time 5.000 sec
Width 8000.0 Hz
16 repetitions
OBSERVE H1, 499.5244685 MHz
DATA PROCESSING
FT size 131072
Total time 1 minutes

INDEX	FREQUENCY	PPM	HEIGHT
1	4145.752	8.299	12.5
2	3954.224	7.916	22.4
3	3952.026	7.912	22.3
4	3907.715	7.823	24.1
5	3899.414	7.806	27.4
6	3773.315	7.554	15.9
7	3771.118	7.549	15.4
8	3765.015	7.537	14.3
9	3762.939	7.533	13.5
10	3628.784	7.264	4.2
11	3574.097	7.155	13.9
12	3572.632	7.152	13.3
13	3565.918	7.139	15.6
14	3564.453	7.136	14.2
15	3400.146	6.807	13.4
16	3391.846	6.790	27.4
17	3383.667	6.774	15.5
18	3328.491	6.663	16.7
19	3327.026	6.660	15.9
20	3320.190	6.647	13.5
21	3318.726	6.644	12.0
22	2053.955	4.112	18.2
23	2051.392	4.107	16.6
24	2049.927	4.104	21.0
25	2047.852	4.100	16.7
26	2045.654	4.095	25.0
27	2025.635	4.055	1.7
28	2025.146	4.054	1.6
29	2005.127	4.014	159.1
30	2000.000	4.004	25.9
31	1997.803	3.999	16.9
32	1995.850	3.995	21.8
33	1994.263	3.992	15.4
34	1991.821	3.987	18.0
35	1985.107	3.974	1.4
36	827.637	1.657	1.5
37	0.000	0.000	5.1

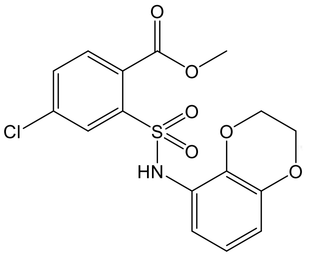

39

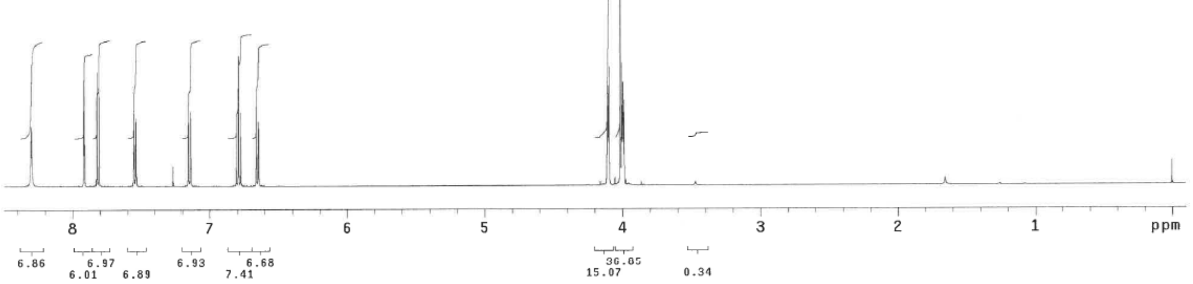

STANDARD 13C
63992
1975-BGE
Berecz Gabor
2006.06.20. (CzB)

Solvent: CDCl3
Temp. 25.0 C / 298.1 K
User: 1-14-87
INOVA-500 "i500"

PULSE SEQUENCE
Relax. delay 3.000 sec
Pulse 6.0 usec
Acq. time 1.300 sec
Width 32000.0 Hz
183 repetitions
OBSERVE C13, 125.6055357 MHz
DECOUPLE H1, 499.5269647 MHz
Power 32 dB
continuously on
WALTZ-16 modulated
DATA PROCESSING
Line broadening 0.5 Hz
FT size 131072
Total time 13 minutes

INDEX	FREQUENCY	PPM	HEIGHT
1	20898.445	166.369	25.7
2	18025.398	143.497	23.3
3	17708.016	140.970	19.8
4	17319.344	137.876	27.7
5	17022.957	135.517	21.4
6	16615.730	132.275	58.8
7	16593.758	132.100	55.5
8	16315.926	129.888	60.8
9	16145.516	128.532	19.4
10	15734.383	125.259	28.6
11	15176.766	120.819	64.8
12	14521.492	115.603	62.1
13	14357.918	114.301	60.0
14	9704.109	77.253	48.0
15	9672.371	77.000	50.1
16	9640.144	76.743	47.9
17	8074.226	64.277	50.4
18	8026.375	63.897	54.8
19	6707.039	53.394	41.3

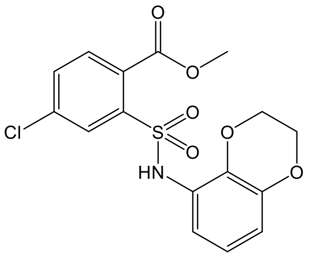

39

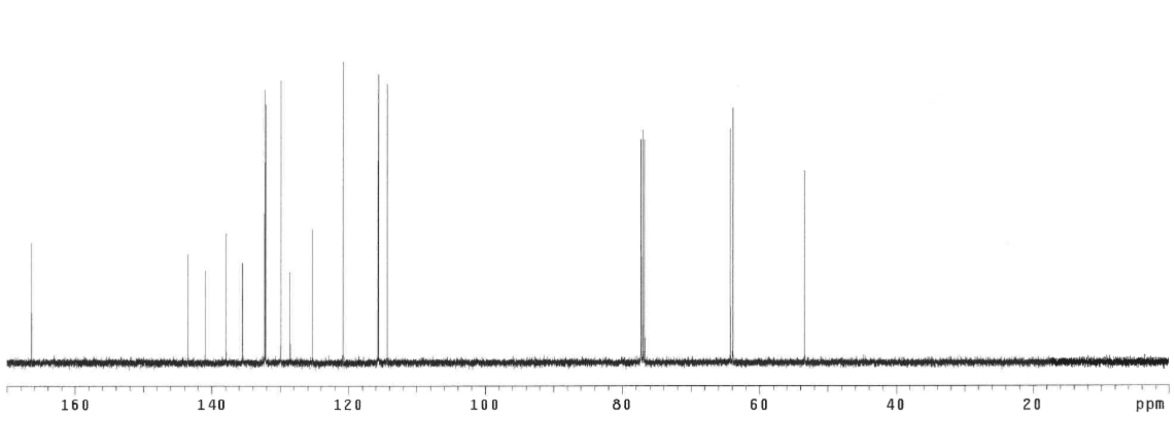

STANDARD 1H
64032
1976-BGE
Berecz Gabor
2006.06.22. (CzB)

Solvent: CDCl3
Temp. 25.0 C / 298.1 K
INOVA-500 "i500"

PULSE SEQUENCE
Relax. delay 1.000 sec
Pulse 6.4 usec
Acq. time 5.000 sec
Width 8000.0 Hz
16 repetitions
OBSERVE H1, 499.5244646 MHz
DATA PROCESSING
FT size 131072
Total time 1 minutes

INDEX	FREQUENCY	PPM	HEIGHT
1	3862.061	7.731	17.7
2	3860.107	7.728	18.1
3	3768.921	7.545	10.5
4	3766.846	7.541	9.9
5	3760.742	7.529	13.2
6	3758.667	7.524	12.9
7	3705.933	7.419	22.5
8	3697.754	7.403	17.5
9	3632.935	7.273	2.8
10	3453.613	6.914	8.8
11	3451.904	6.910	10.1
12	3445.923	6.898	11.5
13	3444.214	6.895	15.0
14	3441.650	6.890	8.7
15	3439.941	6.886	8.6
16	3433.472	6.873	17.3
17	3431.763	6.870	11.2
18	3411.743	6.830	16.9
19	3403.809	6.814	17.2
20	3395.752	6.798	7.7
21	2070.313	4.145	15.6
22	2066.650	4.137	15.9
23	2062.134	4.128	17.9
24	1962.036	3.928	11.5
25	1959.473	3.923	10.5
26	1957.886	3.919	12.4
27	1953.979	3.912	9.6
28	1883.911	3.771	123.3
29	1645.020	3.293	124.3
30	845.337	1.692	3.6
31	0.000	0.000	3.3

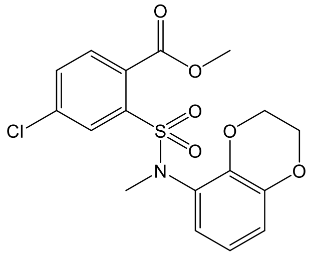

40

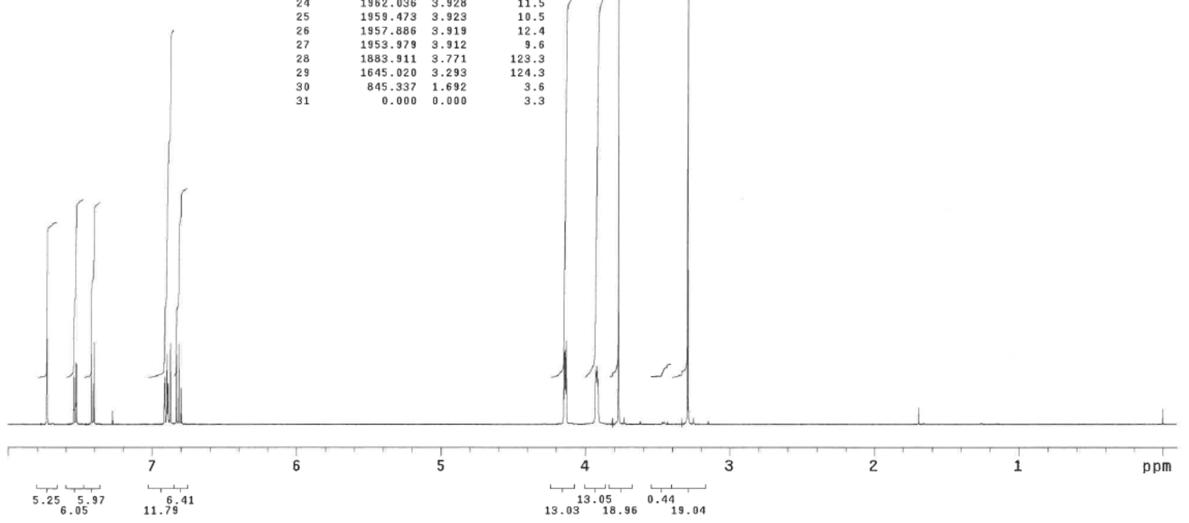

STANDARD 13C
64032
1976-BGE
Berecz Gabor
2006.06.22. (CzB)

Solvent: CDCl3
Temp. 25.0 C / 298.1 K
User: 1-14-87
INOVA-500 "i500"

PULSE SEQUENCE
Relax. delay 3.000 sec
Pulse 6.0 usec
Acq. time 1.300 sec
Width 32000.0 Hz
400 repetitions
OBSERVE C13, 125.6055371 MHz
DECOUPLE H1, 499.5269647 MHz
Power 32 dB
continuously on
WALTZ-16 modulated
DATA PROCESSING
Line broadening 0.5 Hz
FT size 131072
Total time 28 minutes

INDEX	FREQUENCY	PPM	HEIGHT
1	21038.094	167.480	33.9
2	18134.773	144.368	35.3
3	17721.199	141.075	30.0
4	17474.617	139.112	24.2
5	17037.117	135.629	44.0
6	16556.160	131.801	89.8
7	16519.051	131.505	34.0
8	16246.590	129.336	85.8
9	16223.641	129.153	91.4
10	16118.660	128.318	28.8
11	15540.047	123.712	83.5
12	15128.914	120.439	98.5
13	14778.816	117.651	100.0
14	9704.109	77.253	52.1
15	9672.371	77.000	54.7
16	9640.145	76.743	52.4
17	8045.418	64.048	74.7
18	8015.633	63.811	83.6
19	6665.047	53.059	52.1
20	4799.324	38.207	75.9

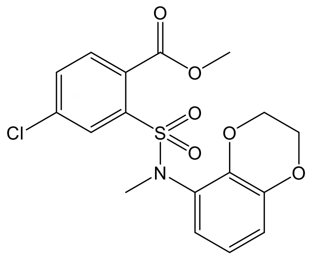

40

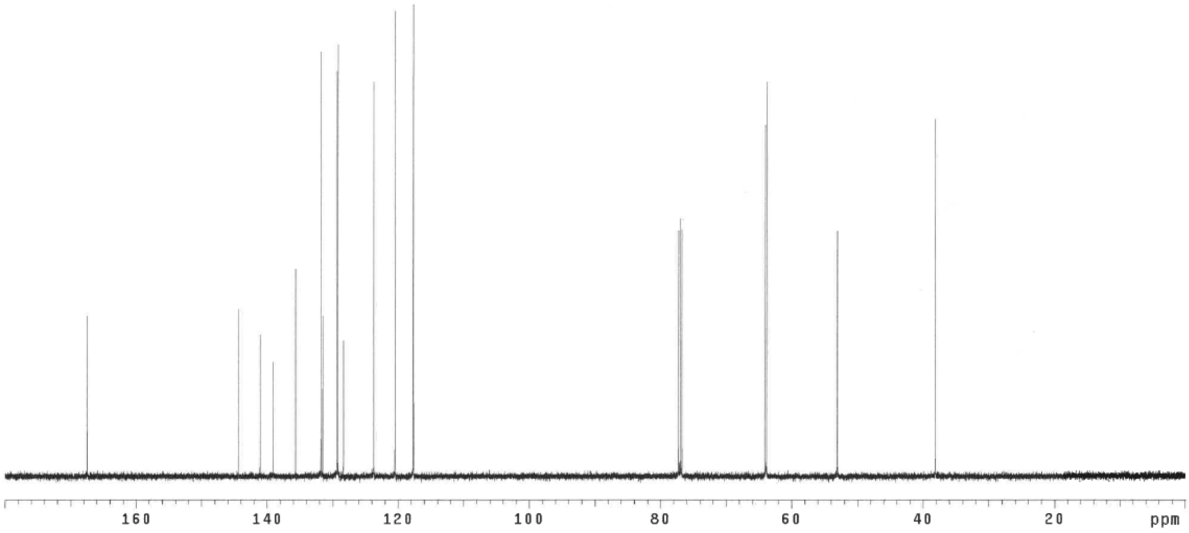

STANDARD 1H
64048
1977-BGE
Berecz Gabor
2006.06.23. (CzB)
Solvent: DMSO
Temp. 25.0 C / 298.1 K
INOVA-500 "i500"
PULSE SEQUENCE
Relax. delay 1.000 sec
Pulse 6.4 usec
Acq. time 5.000 sec
Width 11999.4 Hz
16 repetitions
OBSERVE H1, 499.5268312 MHz
DATA PROCESSING
FT size 131072
Total time 1 minutes

INDEX	FREQUENCY	PPM	HEIGHT
1	6779.690	13.572	0.9
2	3900.318	7.808	16.8
3	3898.121	7.804	17.0
4	3892.078	7.792	20.2
5	3889.881	7.787	20.8
6	3818.840	7.645	35.8
7	3810.600	7.628	29.8
8	3754.939	7.517	31.8
9	3752.925	7.513	30.1
10	3452.647	6.912	13.6
11	3450.816	6.908	14.2
12	3444.591	6.896	21.5
13	3442.760	6.892	20.9
14	3423.352	6.853	0.8
15	3418.957	6.844	14.0
16	3410.901	6.828	32.1
17	3403.028	6.813	21.5
18	3396.986	6.800	24.8
19	3394.972	6.796	24.5
20	3388.930	6.784	11.9
21	3387.099	6.781	9.3
22	2076.312	4.157	19.5
23	2072.467	4.149	26.9
24	2068.256	4.140	21.8
25	2056.721	4.117	0.5
26	1962.609	3.929	19.3
27	1700.232	3.404	1.2
28	1693.275	3.390	2.3
29	1686.317	3.376	2.4
30	1679.359	3.362	1.5
31	1668.740	3.341	1.6
32	1626.628	3.256	0.6
33	1619.304	3.242	1.8
34	1611.797	3.227	0.8
35	1598.980	3.201	164.1
36	1588.727	3.180	1.0
37	1585.065	3.173	0.7
38	1579.206	3.161	1.7
39	1527.023	3.057	0.9
40	1254.027	2.510	6.5
41	1252.379	2.507	8.5
42	1250.548	2.503	6.0
43	552.402	1.106	1.7
44	545.261	1.092	3.5
45	538.303	1.078	1.7
46	-0.000	-0.000	11.3

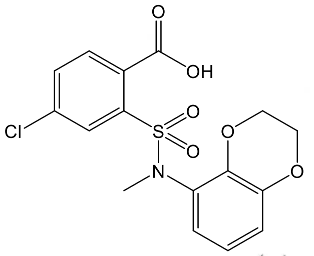

41

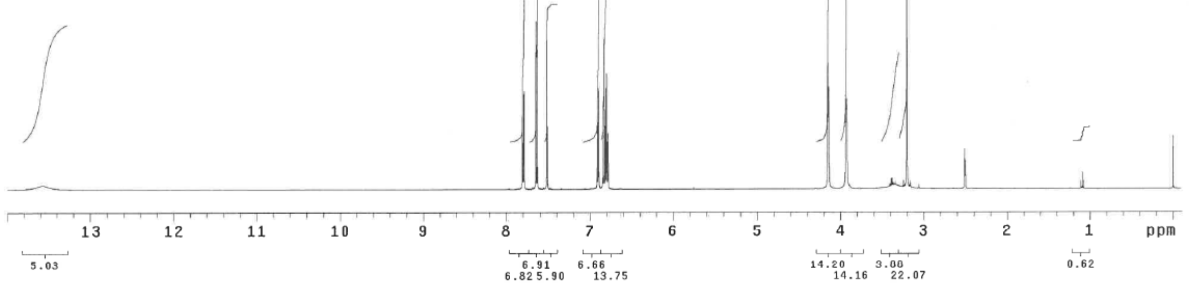

STANDARD 13C
64048
1977-BGE
Berecz Gabor
2006.06.23. (CzB)
Solvent: DMSO
Temp. 25.0 C / 298.1 K
User: 1-14-87
INOVA-500 "i500"
PULSE SEQUENCE
Relax. delay 3.000 sec
Pulse 6.0 usec
Acq. time 1.300 sec
Width 32000.0 Hz
631 repetitions
OBSERVE C13, 125.6061572 MHz
DECOUPLE H1, 499.5293374 MHz
Power 32 dB
continuously on
WALTZ-16 modulated
DATA PROCESSING
Line broadening 0.5 Hz
FT size 131072
Total time 45 minutes

INDEX	FREQUENCY	PPM	HEIGHT
1	21128.364	168.205	35.2
2	18150.825	144.500	36.6
3	17737.739	141.212	36.2
4	17303.169	137.752	40.1
5	16827.583	133.966	31.5
6	16761.665	133.441	17.4
7	16677.680	132.772	38.4
8	16318.794	129.915	37.9
9	16140.083	128.492	37.6
10	16122.505	128.353	34.9
11	15479.926	123.237	44.9
12	15122.505	120.391	42.8
13	14771.919	117.600	42.1
14	8067.329	64.225	35.7
15	8017.524	63.828	37.1
16	5049.751	40.201	23.7
17	5028.755	40.034	70.9
18	5018.989	39.957	7.3
19	5007.759	39.867	141.0
20	4986.762	39.700	165.6
21	4965.766	39.533	139.7
22	4944.770	39.366	70.9
23	4923.774	39.199	22.9
24	4793.891	38.165	60.6

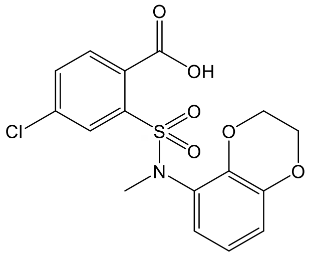

41

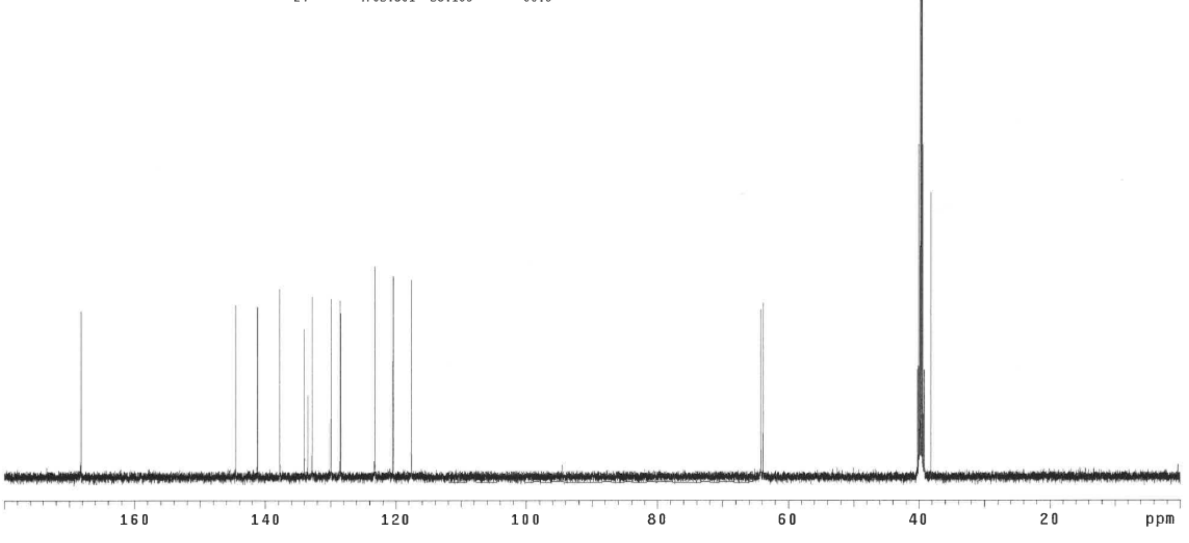

STANDARD 1H
64124
1978-BGE
Berecz Gabor
2006.06.29. (BT)
Solvent: CDCl3 + CD_3OD
Temp. 25.0 C / 298.1 K
INOVA-500 "i500"
PULSE SEQUENCE
Relax. delay 3.000 sec
Pulse 6.4 usec
Acq. time 5.000 sec
Width 8000.0 Hz
16 repetitions
OBSERVE H1, 499.5244200 MHz
DATA PROCESSING
FT size 131072
Total time 2 minutes

INDEX	FREQUENCY	PPM	HEIGHT
1	4007.935	8.024	26.3
2	3999.512	8.007	29.2
3	3985.352	7.978	23.0
4	3983.276	7.974	22.2
5	3946.777	7.901	29.4
6	3937.744	7.883	30.2
7	3851.196	7.710	17.4
8	3849.121	7.706	16.3
9	3842.896	7.693	15.6
10	3840.698	7.689	14.5
11	3677.124	7.361	8.8
12	3488.770	6.984	30.9
13	3479.736	6.966	29.6
14	2206.055	4.416	22.8
15	2202.026	4.408	34.6
16	2195.679	4.396	28.9
17	2191.650	4.387	19.7
18	1816.040	3.636	22.6
19	1550.415	3.104	178.9
20	-0.000	-0.000	7.6

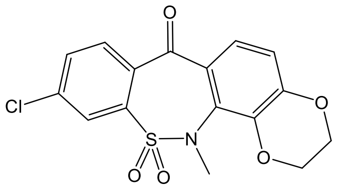

42

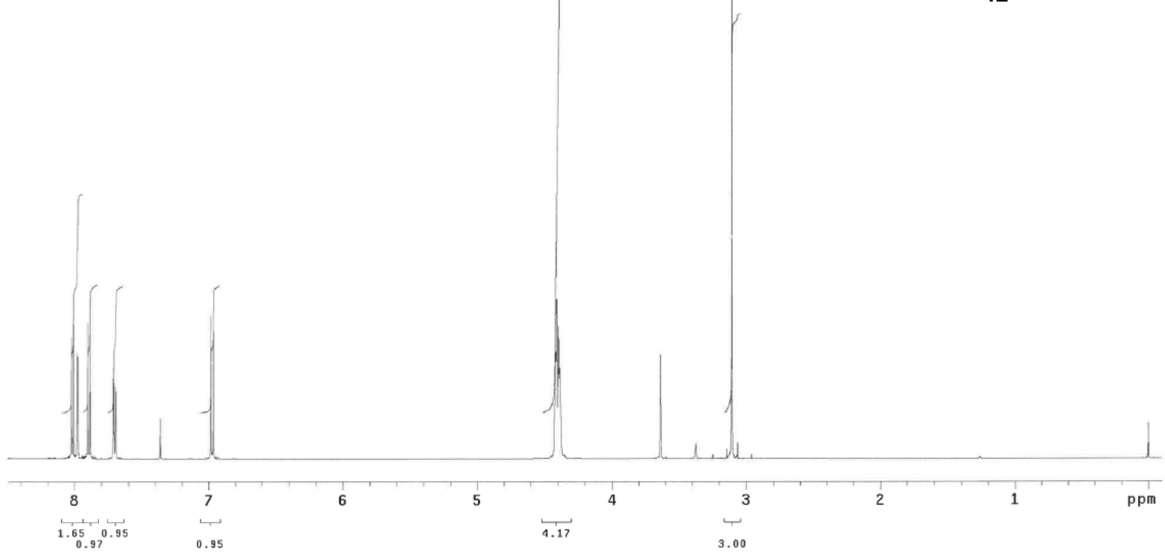

STANDARD 13C
64124
1978-BGE
Berecz Gabor
2006.06.29. (BT)
Solvent: CDCl3 + CD_3OD
Temp. 25.0 C / 298.1 K
User: 1-14-87
INOVA-500 "i500"
PULSE SEQUENCE
Relax. delay 1.200 sec
Pulse 6.0 usec
Acq. time 1.300 sec
Width 32000.0 Hz
288 repetitions
OBSERVE C13, 125.6055381 MHz
DECOUPLE H1, 499.5269647 MHz
Power 32 dB
continuously on
WALTZ-16 modulated
DATA PROCESSING
Line broadening 0.5 Hz
FT size 131072
Total time 12 minutes

INDEX	FREQUENCY	PPM	HEIGHT
1	23565.926	187.604	23.9
2	18758.797	149.335	35.0
3	17411.141	138.607	24.5
4	17397.469	138.498	33.7
5	17279.793	137.561	23.7
6	16842.781	134.082	32.5
7	16812.996	133.845	98.7
8	16720.223	133.107	85.4
9	16393.074	130.502	17.0
10	15881.844	126.432	100.0
11	15820.320	125.943	24.2
12	15622.078	124.365	87.6
13	14605.477	116.272	85.7
14	9704.598	77.257	81.4
15	9672.371	77.000	90.2
16	9640.145	76.743	87.6
17	8080.574	64.328	85.4
18	8051.766	64.099	90.5
19	6175.301	49.160	21.0
20	6153.817	48.989	45.5
21	6132.332	48.818	58.1
22	6110.848	48.647	52.3
23	6089.363	48.476	25.0
24	4740.242	37.736	71.2

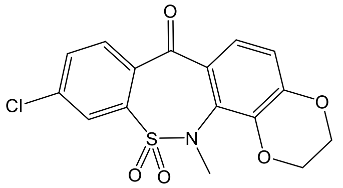

42

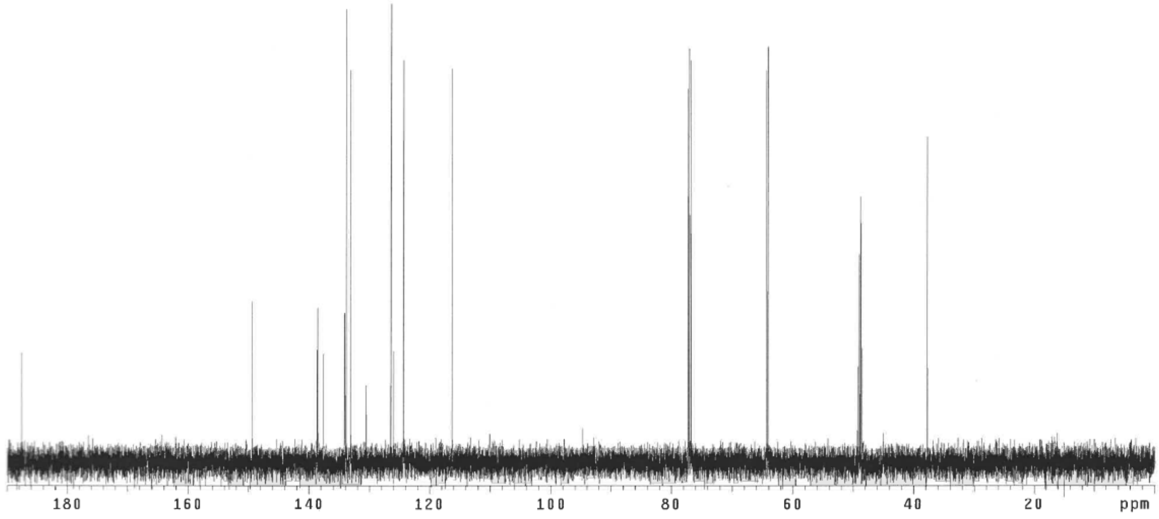

GHSQC_DA (140 Hz)
64124
1978-BGE
Berecz Gabor
2006.06.29. (BT)

Solvent: CDCl3
Temp. 25.0 C / 298.1 K
INOVA-500 "i500"

PULSE SEQUENCE: ghsqc_da
Relax. delay 1.500 sec
Acq. time 0.205 sec
Width 5002.2 Hz
2D Width 32000.0 Hz
2 repetitions
2 x 256 increments
OBSERVE H1, 499.5244235 MHz
DECOUPLE C13, 125.6174312 MHz
Power 42 dB
on during acquisition
off during delay
GARP-1 modulated
DATA PROCESSING
Gauss apodization 0.095 sec
F1 DATA PROCESSING
Gauss apodization 0.015 sec
FT size 2048 x 2048
Total time 30 minutes

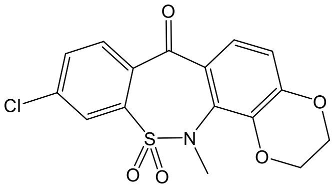

42

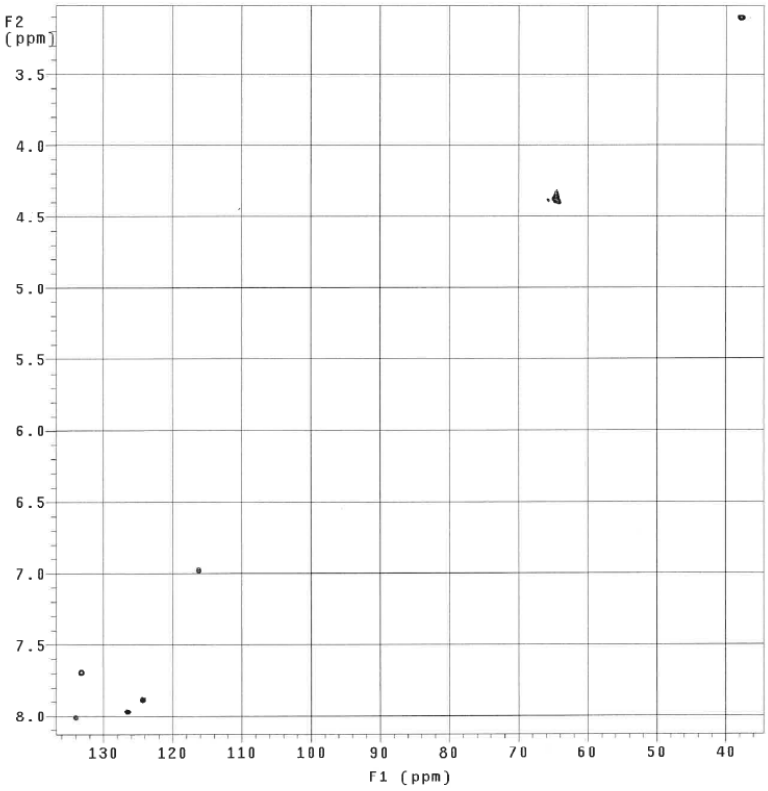

GHMQC_DA (140Hz, 8Hz)
64124
1978-BGE
Berecz Gabor
2006.06.29. (BT)

Solvent: CDCl3
Temp. 25.0 C / 298.1 K
INOVA-500 "i500"

PULSE SEQUENCE: ghmqc_da
Relax. delay 1.500 sec
Acq. time 0.205 sec
Width 5002.2 Hz
2D Width 32000.0 Hz
4 repetitions
256 increments
OBSERVE H1, 499.5244235 MHz
DATA PROCESSING
Sine bell 0.102 sec
F1 DATA PROCESSING
Sine bell 0.002 sec
FT size 4096 x 512
Total time 29 minutes

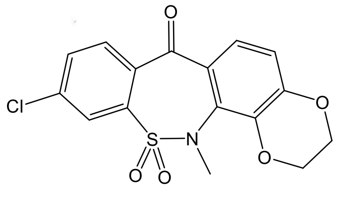

42

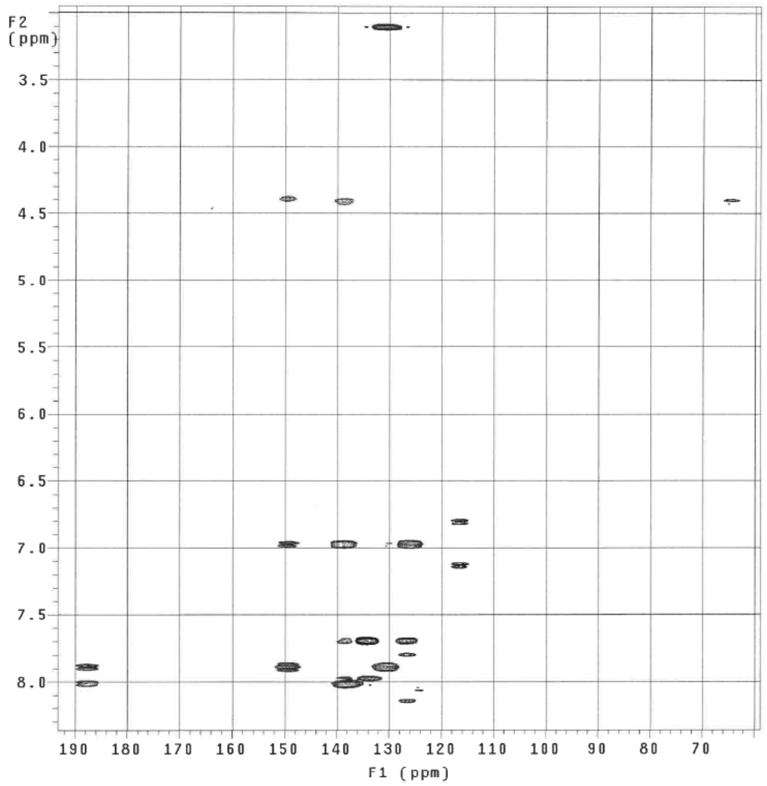

STANDARD 1H
64397
1979-BGE
Berecz Gabor
2007.09.20. (BT)

Solvent: DMSO
Temp. 25.0 C / 298.1 K
INOVA-500 "i500"

PULSE SEQUENCE
Relax. delay 1.000 sec
Pulse 6.4 usec
Acq. time 5.000 sec
Width 11999.4 Hz
32 repetitions
OBSERVE H1, 499.5268339 MHz
DATA PROCESSING
FT size 131072
Total time 3 minutes

INDEX	FREQUENCY	PPM	HEIGHT
1	3960.556	7.929	17.4
2	3952.317	7.912	19.7
3	3879.445	7.766	15.3
4	3858.938	7.725	36.3
5	3842.642	7.693	40.3
6	3507.759	7.022	37.8
7	3499.337	7.005	48.7
8	3460.337	6.927	24.0
9	3451.732	6.910	24.5
10	3281.086	6.568	20.8
11	3116.482	6.239	13.6
12	3088.469	6.183	22.4
13	2859.781	5.725	13.8
14	2175.917	4.356	43.1
15	2146.621	4.297	29.0
16	2137.833	4.280	54.7
17	2123.185	4.250	18.8
18	1677.345	3.358	99.3
19	1664.895	3.333	205.6
20	1646.951	3.297	5.9
21	1627.726	3.259	49.9
22	1251.646	2.506	56.2
23	1249.815	2.502	73.6
24	1248.168	2.499	54.0
25	1036.325	2.075	4.1
26	0.000	0.000	15.9

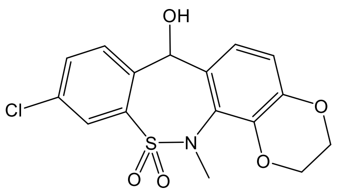

43

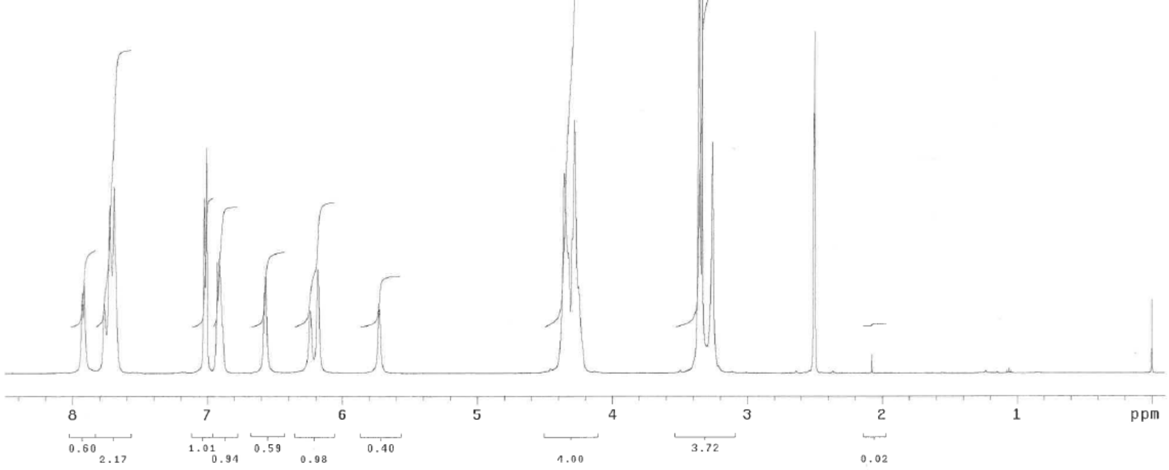

STANDARD 13C
64397
1979-BGE
Berecz Gabor
2007.09.20. (BT)

Solvent: DMSO
Temp. 25.0 C / 298.1 K
User: 1-14-87
INOVA-500 "i500"

PULSE SEQUENCE
Relax. delay 3.000 sec
Pulse 6.0 usec
Acq. time 1.300 sec
Width 32000.0 Hz
15712 repetitions
OBSERVE C13, 125.6061592 MHz
DECOUPLE H1, 499.5293374 MHz
Power 32 dB
continuously on
WALTZ-16 modulated
DATA PROCESSING
Line broadening 1.0 Hz
FT size 131072
Total time 18.8 hours

INDEX	FREQUENCY	PPM	HEIGHT
1	18148.872	144.485	9.3
2	18024.360	143.493	18.2
3	17789.497	141.624	107.8
4	17689.887	140.831	16.8
5	17585.884	140.003	19.8
6	17351.997	138.141	19.2
7	17261.665	137.421	9.7
8	16839.790	134.063	11.2
9	16810.981	133.834	16.6
10	16762.641	133.449	10.4
11	16646.919	132.527	19.4
12	16616.645	132.286	17.1
13	16577.583	131.975	30.9
14	15960.395	127.062	31.7
15	15908.149	126.646	57.7
16	15395.942	122.568	15.9
17	15218.696	121.157	17.2
18	14764.595	117.542	32.0
19	14655.708	116.675	17.5
20	14563.423	115.941	31.5
21	9472.602	75.412	18.1
22	8286.567	65.970	34.3
23	8111.762	64.578	50.7
24	8049.262	64.081	127.6
25	5061.470	40.295	15.9
26	5049.751	40.201	120.2
27	5040.473	40.128	37.1
28	5028.755	40.034	362.7
29	5019.477	39.960	64.1
30	5007.759	39.867	719.2
31	4998.481	39.793	65.4
32	4986.762	39.700	843.9
33	4965.766	39.533	717.6
34	4944.770	39.366	359.3
35	4923.774	39.199	118.3
36	4886.177	38.899	17.9
37	4523.872	36.015	31.3

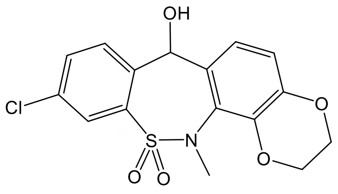

43

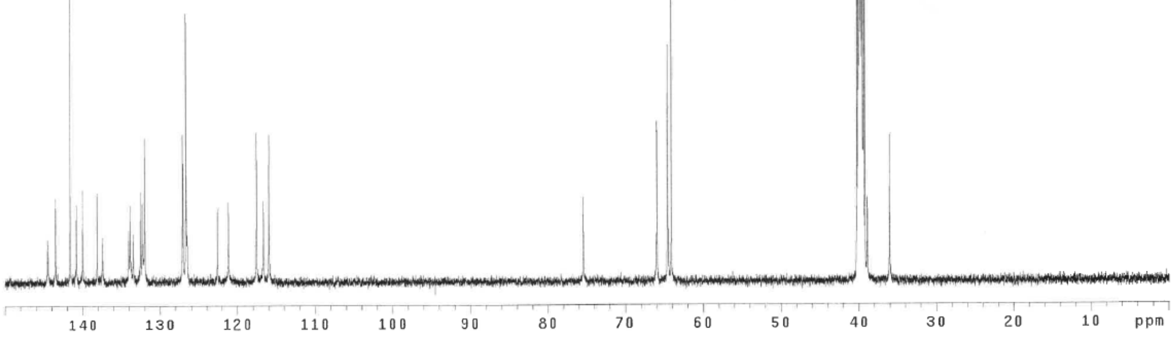

STANDARD 1H
71204
2122-BGE
Berecz Gabor
2007.10.08. (BT)

Solvent: DMSO
Temp. 25.0 C / 298.1 K
INOVA-500 "i500"

PULSE SEQUENCE
Relax. delay 1.000 sec
Pulse 6.4 usec
Acq. time 5.000 sec
Width 8000.0 Hz
16 repetitions
OBSERVE H1, 499.5268326 MHz
DATA PROCESSING
FT size 131072
Total time 1 minutes

INDEX	FREQUENCY	PPM	HEIGHT
1	3874.268	7.756	47.1
2	3865.601	7.739	6.4
3	3857.056	7.721	16.1
4	3847.168	7.702	12.5
5	3838.379	7.684	5.8
6	3833.008	7.673	14.3
7	3831.055	7.669	14.1
8	3824.707	7.657	19.7
9	3822.754	7.653	19.9
10	3791.016	7.589	30.1
11	3782.593	7.572	20.3
12	3454.468	6.915	48.1
13	3441.895	6.890	52.8
14	3439.453	6.885	55.2
15	3431.030	6.869	7.5
16	2562.256	5.129	7.5
17	2553.833	5.113	7.8
18	2334.473	4.673	37.3
19	2198.120	4.400	6.1
20	2186.890	4.378	13.6
21	2184.326	4.373	13.4
22	2180.176	4.364	11.3
23	2170.898	4.346	14.1
24	2168.335	4.341	13.7
25	2165.771	4.336	15.5
26	2163.330	4.331	18.0
27	2155.396	4.315	21.5
28	2145.142	4.294	25.1
29	2143.311	4.291	26.3
30	2138.306	4.281	18.1
31	2135.620	4.275	21.4
32	2133.179	4.270	19.5
33	2125.000	4.254	12.9
34	1682.251	3.368	148.6
35	1670.898	3.345	19.1
36	1665.894	3.335	77.0
37	1619.141	3.241	61.0
38	1502.563	3.008	4.2
39	1252.930	2.508	19.2
40	1251.099	2.505	26.5
41	1249.390	2.501	20.3
42	1164.795	2.332	27.6
43	1160.400	2.323	28.4
44	1139.526	2.281	12.5
45	1045.532	2.093	95.6
46	1038.574	2.079	7.6
47	1037.842	2.078	8.8
48	1037.598	2.077	8.8
49	0.977	0.002	3.2
50	0.244	0.000	5.9
51	-0.000	-0.000	6.1

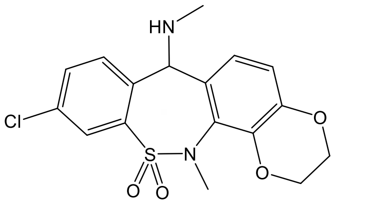

45a

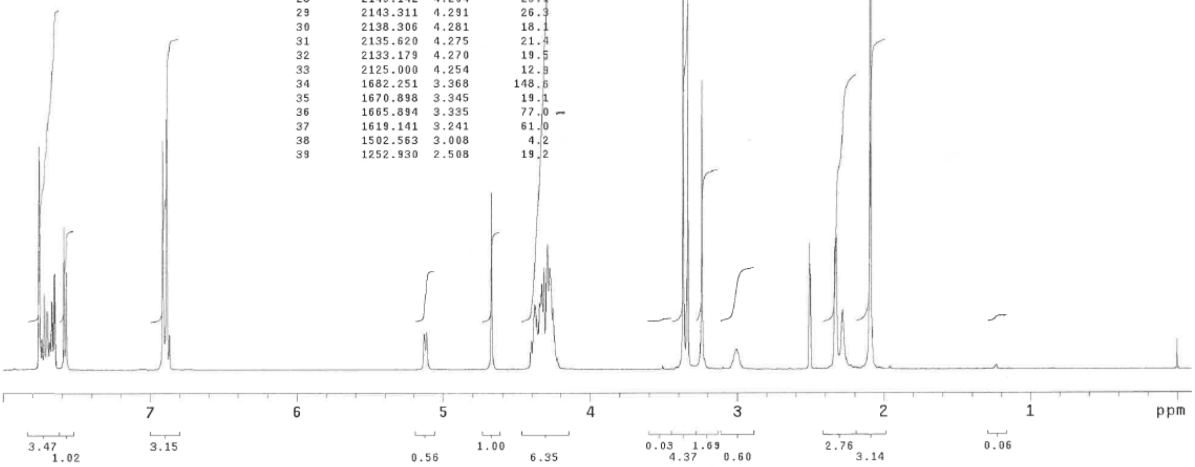

STANDARD 13C
71204
2122-BGE
Berecz Gabor
2007.10.08. (BT)

Solvent: DMSO
Temp. 25.0 C / 298.1 K
User: 1-14-87
INOVA-500 "i500"

PULSE SEQUENCE
Relax. delay 3.000 sec
Pulse 6.0 usec
Acq. time 1.300 sec
Width 32000.0 Hz
1024 repetitions
OBSERVE C13, 125.6061597 MHz
DECOUPLE H1, 499.5293374 MHz
Power 32 dB
continuously on
WALTZ-16 modulated
DATA PROCESSING
Line broadening 0.5 Hz
FT size 131072
Total time 73 minutes

INDEX	FREQUENCY	PPM	HEIGHT
1	18140.571	144.418	49.1
2	17997.505	143.280	22.1
3	17987.739	143.202	47.7
4	17840.766	142.032	40.0
5	17762.153	141.406	15.1
6	17634.223	140.387	15.9
7	17431.587	138.774	16.5
8	17191.841	136.866	47.8
9	16914.009	134.654	65.6
10	16770.942	133.515	49.9
11	16745.552	133.313	48.3
12	16614.204	132.267	20.1
13	16594.673	132.111	28.0
14	16544.868	131.715	58.2
15	16179.634	128.807	12.7
16	15997.993	127.361	26.1
17	15890.571	126.506	63.5
18	15843.208	126.129	43.6
19	15702.095	125.006	10.9
20	15396.919	122.576	63.6
21	14797.798	117.806	12.1
22	14725.532	117.231	27.6
23	14669.868	116.788	66.5
24	8819.770	70.215	70.6
25	8102.485	64.505	61.8
26	8048.286	64.073	56.6
27	8042.427	64.026	28.9
28	7586.372	60.396	11.4
29	5060.493	40.287	7.4
30	5049.751	40.201	62.3
31	5040.473	40.128	14.2
32	5028.755	40.034	187.9
33	5018.989	39.957	22.4
34	5007.759	39.867	374.0
35	4997.993	39.789	20.7
36	4986.763	39.700	441.5
37	4965.766	39.533	374.8
38	4944.770	39.366	185.9
39	4923.774	39.199	59.3
40	4825.141	38.413	76.6
41	4583.442	36.489	30.6
42	4383.735	34.899	94.2
43	4345.161	34.592	39.1

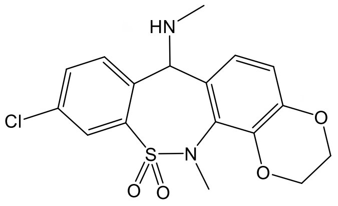

45a

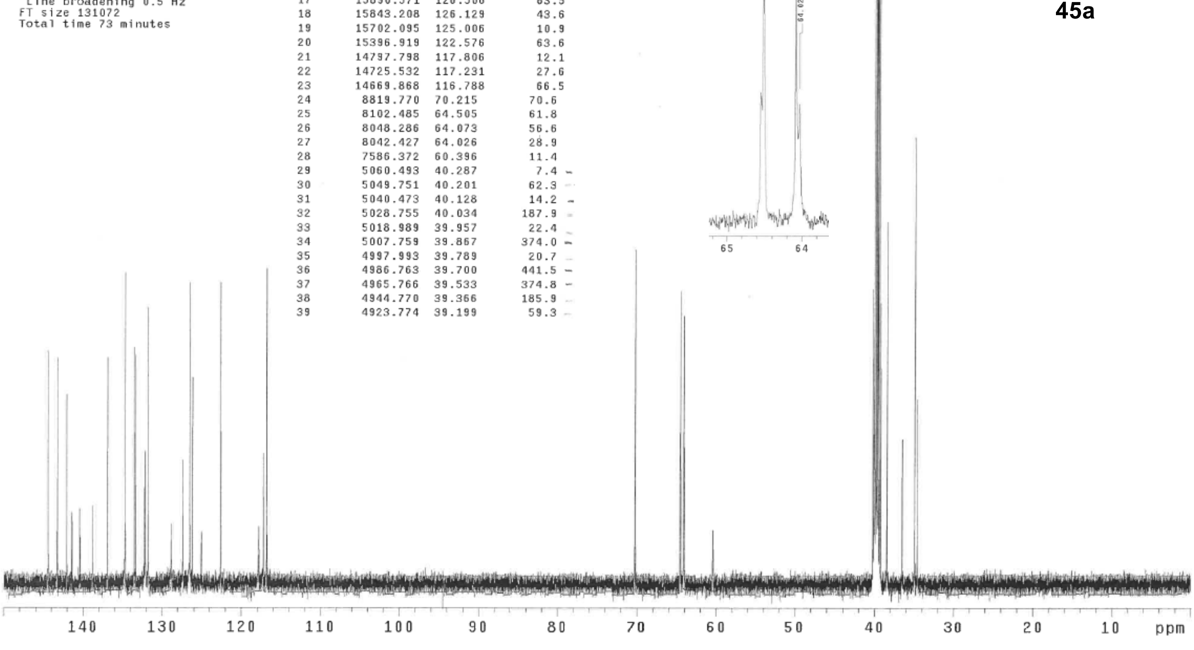

STANDARD 1H
64558
1982-BGE
Berecz Gabor
2006.07.24. (BB)
Solvent: CDCl3
Temp. 25.0 C / 298.1 K
INOVA-500 "i500"
PULSE SEQUENCE
Relax. delay 1.000 sec
Pulse 6.4 usec
Acq. time 5.000 sec
Width 8000.0 Hz
16 repetitions
OBSERVE H1, 499.5244689 MHz
DATA PROCESSING
FT size 131072
Total time 1 minutes

INDEX	FREQUENCY	PPM	HEIGHT	INDEX	FREQUENCY	PPM	HEIGHT
1	3966.309	7.940	19.0	40	1110.962	2.224	8.2
2	3964.111	7.936	18.8	41	1108.765	2.220	7.6
3	3676.514	7.360	11.1	42	1106.567	2.215	9.1
4	3674.316	7.356	10.5	43	1104.248	2.211	12.3
5	3668.213	7.343	18.7	44	1099.976	2.202	5.1
6	3666.016	7.339	18.4	45	1077.393	2.157	4.7
7	3644.043	7.295	26.4	46	1075.928	2.154	5.0
8	3635.742	7.278	16.0	47	1071.045	2.144	12.4
9	3629.395	7.266	2.1	48	1069.092	2.140	9.3
10	3628.418	7.264	7.1	49	1064.697	2.131	8.2
11	3395.508	6.797	20.7	50	1062.134	2.126	8.2
12	3387.085	6.781	37.2	51	1057.617	2.117	3.5
13	3367.676	6.742	30.8	52	848.633	1.699	1.7
14	3359.131	6.725	17.0	53	844.116	1.690	2.9
15	2199.219	4.403	2.3	54	840.576	1.683	14.2
16	2195.435	4.395	3.8	55	836.792	1.675	20.8
17	2194.458	4.393	3.9	56	834.106	1.670	41.1
18	2190.796	4.386	3.4	57	831.543	1.665	19.7
19	2187.622	4.379	6.9	58	827.759	1.657	12.0
20	2184.204	4.373	9.2	59	819.946	1.641	1.6
21	2182.739	4.370	10.9	60	814.331	1.630	6.4
22	2179.565	4.363	9.0	61	0.977	0.002	2.3
23	2176.636	4.357	6.5	62	-0.000	-0.000	8.1
24	2172.852	4.350	14.1				
25	2168.579	4.341	9.9				
26	2165.039	4.334	2.2				
27	2161.133	4.326	5.3				
28	2157.227	4.319	4.4				
29	2151.001	4.306	1.9				
30	2149.414	4.303	1.9				
31	2142.822	4.290	20.7				
32	2138.794	4.282	27.2				
33	2135.132	4.274	11.6				
34	2017.700	4.039	32.0				
35	1730.591	3.464	1.6				
36	1717.773	3.439	1.6				
37	1710.327	3.424	163.4				
38	1119.263	2.241	3.2				
39	1113.281	2.229	8.2				

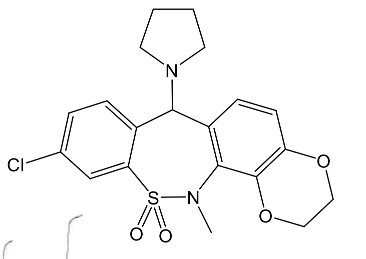

45b

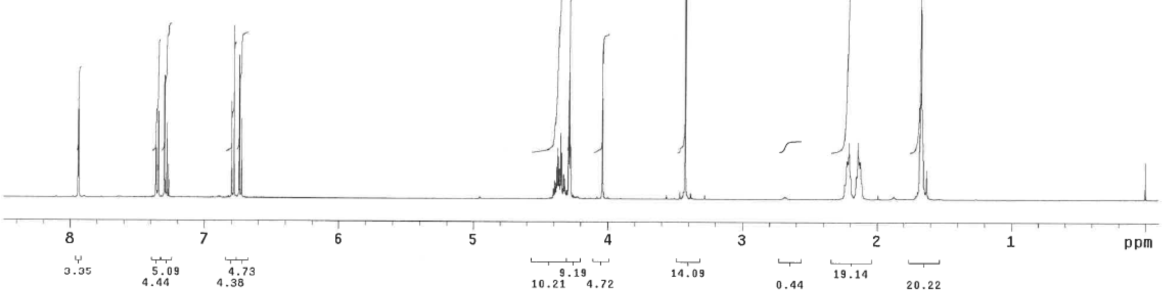

STANDARD 13C
64558
1982-BGE
Berecz Gabor
2006.07.24. (BB)
Solvent: CDCl3
Temp. 25.0 C / 298.1 K
User: 1-14-87
INOVA-500 "i500"
PULSE SEQUENCE
Relax. delay 3.000 sec
Pulse 6.0 usec
Acq. time 1.300 sec
Width 27220.1 Hz
102 repetitions
OBSERVE C13, 125.6055333 MHz
DECOUPLE H1, 499.5269647 MHz
Power 32 dB
continuously on
WALTZ-16 modulated
DATA PROCESSING
Line broadening 1.0 Hz
FT size 131072
Total time 7 minutes

INDEX	FREQUENCY	PPM	HEIGHT
1	18160.806	144.575	19.1
2	18019.588	143.451	17.6
3	17881.277	142.350	15.2
4	17038.955	135.644	23.0
5	16935.118	134.817	24.6
6	16928.473	134.765	22.0
7	16713.323	133.052	46.4
8	16440.441	130.879	43.5
9	16090.304	128.092	46.4
10	15785.024	125.662	15.5
11	15235.521	121.287	45.0
12	14712.600	117.124	46.6
13	9704.353	77.255	64.8
14	9672.371	77.000	66.0
15	9640.389	76.745	65.0
16	9613.807	76.534	43.2
17	8099.039	64.475	44.2
18	8053.350	64.111	43.0
19	6739.194	53.650	86.2
20	4787.482	38.112	33.9
21	2909.700	23.164	89.6

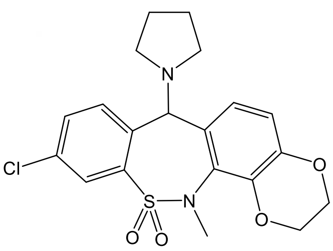

45b

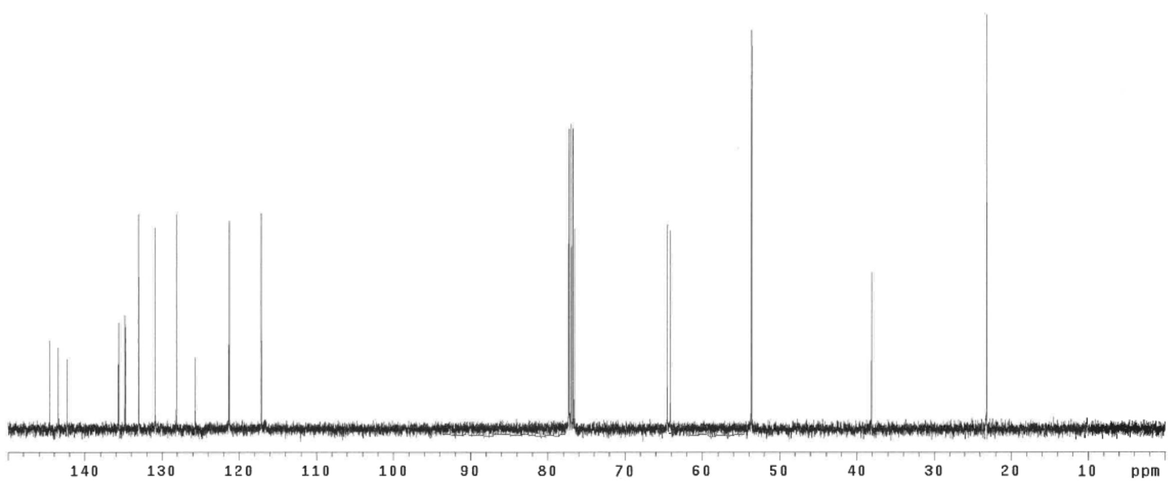

STANDARD 1H
66309
2025-BGE
Berecz Gabor
2006.11.14. (BB)

Solvent: DMSO
Temp. 25.0 C / 298.1 K
INOVA-500 "i500"

PULSE SEQUENCE
Relax. delay 1.000 sec
Pulse 6.4 usec
Acq. time 5.000 sec
Width 8000.0 Hz
16 repetitions
OBSERVE H1, 499.5268317 MHz
DATA PROCESSING
FT size 131072
Total time 1 minutes

INDEX	FREQUENCY	PPM	HEIGHT	INDEX	FREQUENCY	PPM	HEIGHT
1	3881.470	7.770	24.0	40	1017.822	2.038	5.6
2	3879.272	7.766	24.7	41	1014.160	2.030	3.4
3	3820.923	7.649	11.8	42	1006.226	2.014	3.1
4	3818.726	7.645	10.5	43	929.932	1.862	3.3
5	3812.622	7.632	18.1	44	920.166	1.842	6.1
6	3810.303	7.628	17.5	45	918.457	1.839	6.0
7	3783.813	7.575	23.6	46	913.696	1.829	3.6
8	3775.391	7.558	15.1	47	911.011	1.824	4.4
9	3596.191	7.199	10.1	48	909.180	1.820	4.5
10	3441.284	6.889	11.0	49	901.855	1.805	6.3
11	3432.861	6.872	47.2	50	899.658	1.801	6.1
12	3428.589	6.864	37.6	51	890.137	1.782	3.6
13	3420.166	6.847	8.6	52	804.565	1.611	5.8
14	3357.300	6.721	10.0	53	800.659	1.603	5.4
15	2203.369	4.411	3.4	54	793.945	1.589	7.0
16	2198.853	4.402	4.4	55	791.260	1.584	7.6
17	2197.510	4.399	4.9	56	730.835	1.463	3.6
18	2194.214	4.393	4.8	57	727.539	1.456	4.0
19	2188.354	4.381	8.3	58	717.529	1.436	4.5
20	2158.569	4.321	14.6	59	704.712	1.411	4.6
21	2150.757	4.306	15.2	60	695.190	1.392	3.5
22	2149.414	4.303	11.9	61	692.261	1.386	3.1
23	2142.578	4.289	3.1				
24	2127.808	4.260	34.8				
25	2122.314	4.249	5.3				
26	2118.896	4.242	5.1				
27	2113.281	4.231	3.3				
28	1684.448	3.372	123.5				
29	1665.405	3.334	71.2				
30	1255.615	2.514	7.1				
31	1253.784	2.510	9.7				
32	1251.953	2.506	11.1				
33	1250.122	2.503	8.1				
34	1248.291	2.499	5.3				
35	1244.263	2.491	4.8				
36	1145.142	2.292	4.5				
37	1134.399	2.271	5.0				
38	1038.086	2.078	4.9				
39	1021.606	2.045	3.3				

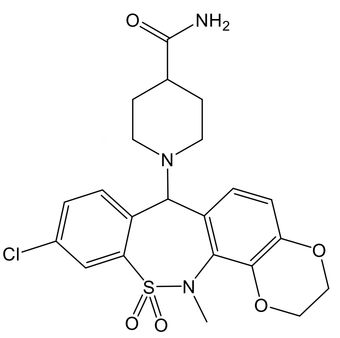

45c

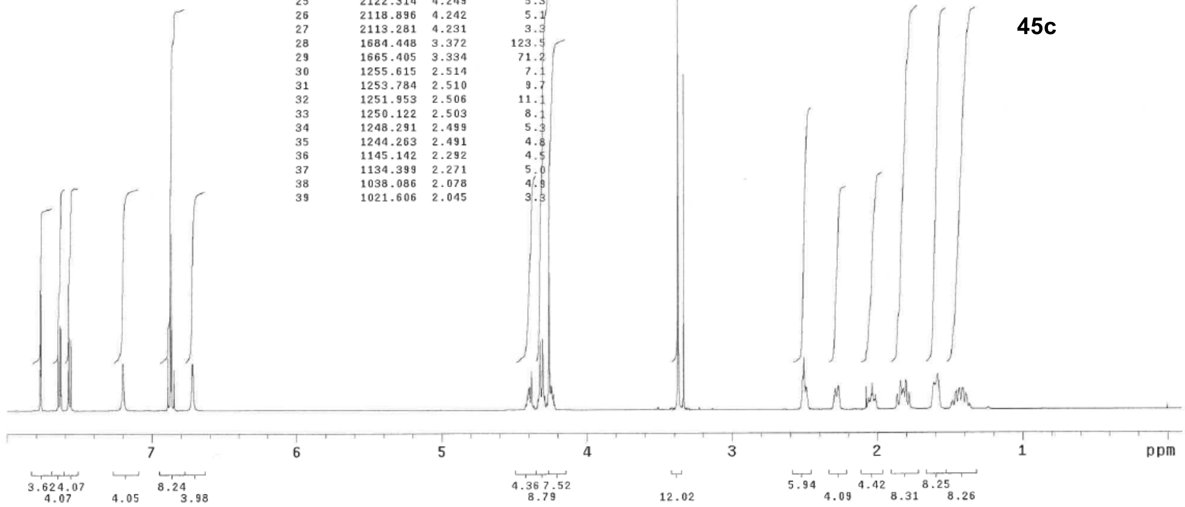

STANDARD 13C
66309
2025-BGE
Berecz Gabor
2006.11.14. (BB)

Solvent: DMSO
Temp. 25.0 C / 298.1 K
User: 1-14-87
INOVA-500 "i500"

PULSE SEQUENCE
Relax. delay 3.000 sec
Pulse 6.0 usec
Acq. time 1.300 sec
Width 32000.0 Hz
1024 repetitions
OBSERVE C13, 125.6061597 MHz
DECOUPLE H1, 499.5293374 MHz
Power 32 dB
continuously on
WALTZ-16 modulated
DATA PROCESSING
Line broadening 0.5 Hz
FT size 131072
Total time 73 minutes

INDEX	FREQUENCY	PPM	HEIGHT
1	22172.798	176.519	84.8
2	18155.220	144.535	77.0
3	18017.036	143.435	67.9
4	17904.731	142.541	63.0
5	16970.649	135.105	55.0
6	16962.837	135.042	65.4
7	16826.118	133.954	69.6
8	16817.329	133.884	69.8
9	16508.247	131.423	55.9
10	15991.645	127.311	57.3
11	15706.001	125.037	74.9
12	15359.321	122.277	59.4
13	14710.395	117.111	62.4
14	9464.302	75.346	70.7
15	8104.438	64.520	48.6
16	8048.286	64.073	46.2
17	6549.751	52.143	36.5
18	6508.735	51.817	43.2
19	5221.626	41.570	28.9
20	5049.751	40.201	71.1
21	5028.755	40.034	227.2
22	5018.989	39.957	16.1
23	5007.759	39.867	453.5
24	4986.763	39.700	537.0
25	4965.766	39.533	455.6
26	4944.770	39.366	228.6
27	4923.774	39.199	74.2
28	4860.786	38.697	90.8
29	3623.970	28.851	41.5
30	3565.376	28.384	47.2

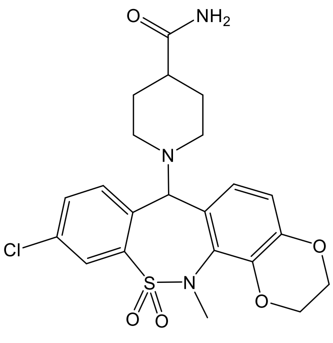

45c

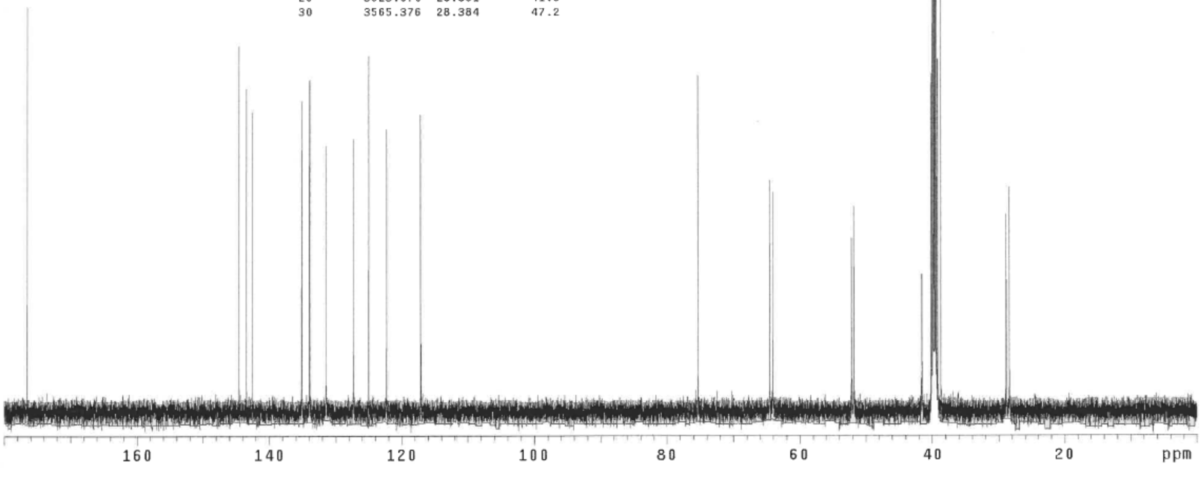

STANDARD 1H
64557
1981-BGE
Berecz Gabor
2006.07.24. (BB)
Solvent: CDCl3
Temp. 25.0 C / 298.1 K
INOVA-500 "i500"
PULSE SEQUENCE
Relax. delay 1.000 sec
Pulse 6.4 usec
Acq. time 5.000 sec
Width 8000.0 Hz
16 repetitions
OBSERVE H1, 499.5244693 MHz
DATA PROCESSING
FT size 131072
Total time 1 minutes

2 konformer

INDEX	FREQUENCY	PPM	HEIGHT
1	3978.760	7.965	18.8
2	3931.763	7.871	12.5
3	3932.007	7.871	12.5
4	3857.300	7.722	9.6
5	3849.365	7.706	10.7
6	3723.389	7.454	13.3
7	3716.553	7.440	22.0
8	3707.031	7.421	16.4
9	3692.383	7.392	19.9
10	3684.082	7.375	12.5
11	3627.319	7.262	23.6
12	3429.443	6.865	56.3
13	3421.021	6.849	53.6
14	2873.901	5.753	17.4
15	2641.846	5.289	4.4
16	2498.657	5.002	26.8
17	2174.927	4.354	51.7
18	2143.677	4.291	42.3
19	2137.329	4.279	42.6
20	1790.039	3.583	52.1
21	1746.460	3.496	11.8
22	1739.380	3.482	50.2
23	1736.572	3.476	53.0
24	1732.422	3.468	43.6
25	1725.464	3.454	15.2
26	1685.547	3.374	81.7
27	1610.474	3.224	81.7
28	821.899	1.645	35.0
29	608.521	1.218	16.5
30	601.562	1.204	32.5
31	594.482	1.190	16.0
32	-0.000	-0.000	25.9

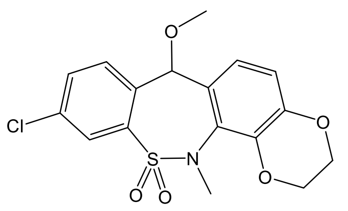

46

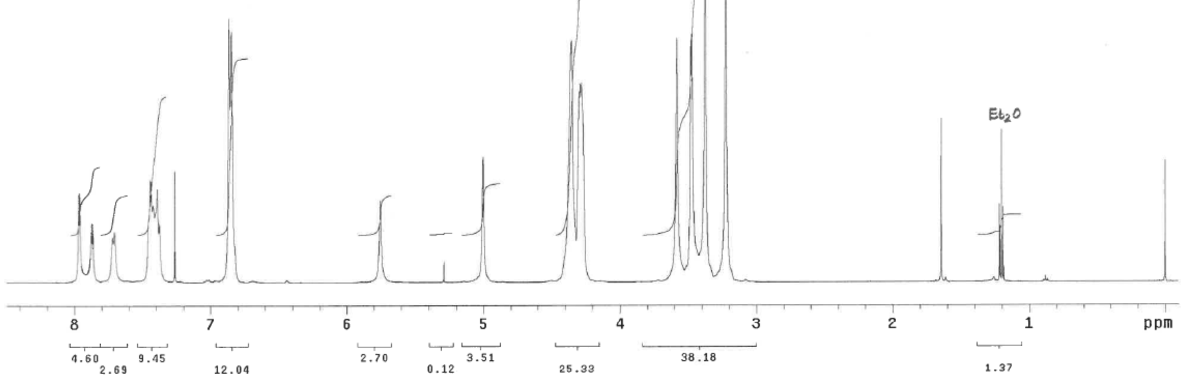

STANDARD 13C
64557
1981-BGE
Berecz Gabor
2006.07.24. (BB)
Solvent: CDCl3
Temp. 25.0 C / 298.1 K
User: 1-14-87
INOVA-500 "i500"
PULSE SEQUENCE
Relax. delay 3.000 sec
Pulse 6.0 usec
Acq. time 1.300 sec
Width 27220.1 Hz
823 repetitions
OBSERVE C13, 125.6055354 MHz
DECOUPLE H1, 499.5269647 MHz
Power 32 dB
continuously on
WALTZ-16 modulated
DATA PROCESSING
Line broadening 4.0 Hz
FT size 131072
Total time 58 minutes

INDEX	FREQUENCY	PPM	HEIGHT
1	18252.597	145.306	18.0
2	18071.922	143.867	11.5
3	17911.598	142.591	16.9
4	17875.047	142.300	18.9
5	17578.905	139.942	10.6
6	17269.888	137.482	13.8
7	17022.341	135.512	21.7
8	16994.098	135.287	15.2
9	16815.083	133.862	15.6
10	16754.858	133.382	50.6
11	16531.817	131.607	58.5
12	16470.761	131.121	22.0
13	16078.259	127.996	59.3
14	15853.141	126.204	14.7
15	15755.119	125.424	33.8
16	15460.639	123.079	11.0
17	15341.019	122.127	48.3
18	14822.251	117.997	33.8
19	14695.986	116.992	50.5
20	14496.620	115.405	32.6
21	10968.667	87.320	47.5
22	9704.353	77.255	255.8
23	9672.371	77.000	259.9
24	9640.389	76.745	288.6
25	8261.439	65.768	9.4
26	8098.208	64.468	90.8
27	8052.520	64.105	167.0
28	7342.693	58.454	30.1
29	7134.604	56.797	45.3
30	4812.818	38.314	37.5
31	4483.033	35.689	26.6
32	1911.208	15.215	8.6

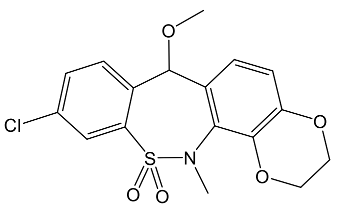

46

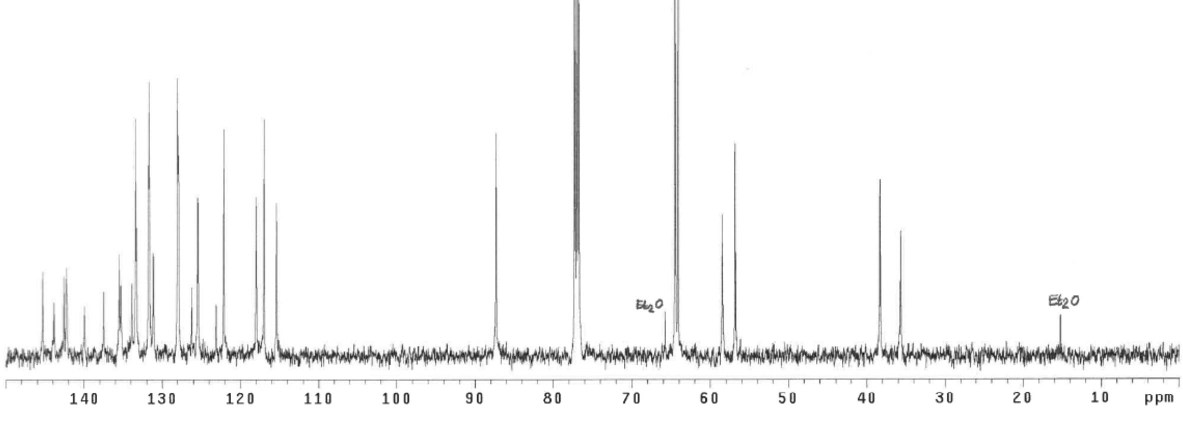

STANDARD PROTON PARAMETERS
57140
1825-BGE
Berecz Gabor
2005.03.11. (BT)
Solvent: CDCl3
Temp. 25.0 C / 298.1 K
INOVA-500 "i500"
PULSE SEQUENCE
Relax. delay 3.000 sec
Pulse 90.0 degrees
Acq. time 5.000 sec
Width 8000.0 Hz
16 repetitions
OBSERVE H1, 499.5244670 MHz
DATA PROCESSING
FT size 131072
Total time 2 minutes

INDEX	FREQUENCY	PPM	HEIGHT	INDEX	FREQUENCY	PPM	HEIGHT
1	5302.612	10.615	21.5	40	3419.678	6.846	57.4
2	3960.938	7.929	23.2	41	2643.921	5.293	9.2
3	3960.571	7.929	20.5	42	2130.859	4.266	13.7
4	3959.229	7.926	25.0	43	2129.639	4.263	16.2
5	3958.862	7.925	20.3	44	2127.563	4.259	41.0
6	3952.881	7.913	24.6	45	2125.977	4.256	24.0
7	3952.515	7.913	22.2	46	2124.146	4.252	41.5
8	3951.294	7.910	25.1	47	2122.559	4.249	51.3
9	3950.928	7.909	20.4	48	2121.948	4.248	49.7
10	3833.130	7.674	21.9	49	2115.479	4.235	51.6
11	3832.764	7.673	21.3	50	2114.868	4.234	47.0
12	3832.031	7.671	23.6	51	2113.281	4.231	41.9
13	3824.707	7.657	26.2	52	2111.450	4.227	24.7
14	3824.341	7.656	25.6	53	2109.863	4.224	41.8
15	3823.608	7.654	27.1	54	2107.788	4.220	16.4
16	3727.783	7.463	12.8	55	2106.567	4.217	13.2
17	3726.196	7.459	13.3	56	1939.087	3.882	406.2
18	3720.459	7.448	14.7	57	824.463	1.650	9.8
19	3720.215	7.448	14.0	58	0.000	0.000	18.2
20	3718.994	7.445	20.9				
21	3717.773	7.443	12.5				
22	3712.036	7.431	12.1				
23	3710.449	7.428	12.2				
24	3687.134	7.381	47.2				
25	3684.937	7.377	64.1				
26	3672.974	7.353	42.3				
27	3670.654	7.348	31.4				
28	3664.429	7.336	42.9				
29	3662.109	7.331	36.1				
30	3629.761	7.266	15.2				
31	3517.334	7.041	18.9				
32	3516.235	7.039	20.0				
33	3510.010	7.027	22.6				
34	3509.399	7.025	26.3				
35	3508.911	7.025	24.0				
36	3508.301	7.023	20.6				
37	3502.075	7.011	18.6				
38	3500.977	7.009	17.4				
39	3428.223	6.863	61.0				

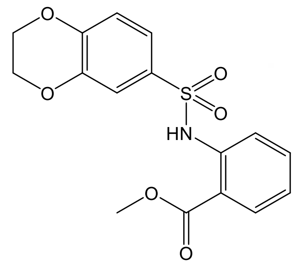

48

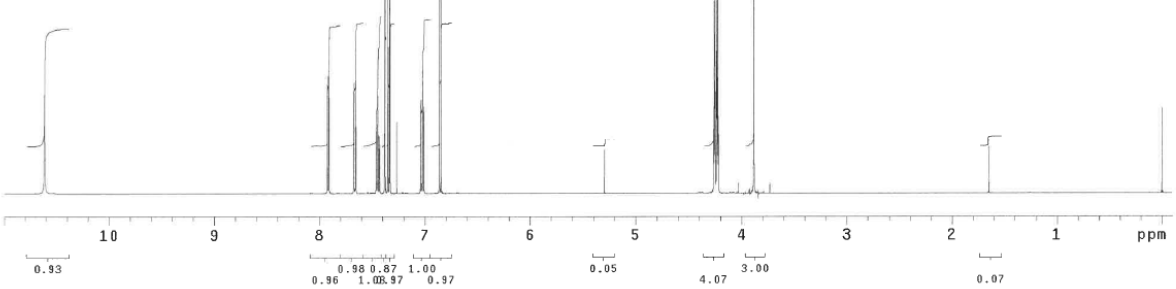

STANDARD CARBON PARAMETERS
57140
1825-BGE
Berecz Gabor
2005.03.11. (BT)
Solvent: CDCl3
Temp. 25.0 C / 298.1 K
User: 1-14-87
INOVA-500 "i500"
PULSE SEQUENCE
Relax. delay 3.000 sec
Pulse 45.0 degrees
Acq. time 1.300 sec
Width 32000.0 Hz
240 repetitions
OBSERVE C13, 125.6055337 MHz
DECOUPLE H1, 499.5269647 MHz
Power 32 dB
continuously on
WALTZ-16 modulated
DATA PROCESSING
Line broadening 0.5 Hz
FT size 131072
Total time 17 minutes

INDEX	FREQUENCY	PPM	HEIGHT
1	21134.285	168.246	50.8
2	18543.953	147.625	60.8
3	18013.191	143.400	65.5
4	17646.004	140.477	57.7
5	16888.679	134.448	143.0
6	16545.418	131.715	37.6
7	16467.293	131.093	146.4
8	15406.746	122.650	152.1
9	15194.344	120.959	162.5
10	14911.140	118.705	96.2
11	14772.469	117.601	166.7
12	14679.695	116.862	147.8
13	14529.304	115.665	39.0
14	9704.109	77.253	94.8
15	9672.371	77.000	98.9
16	9640.144	76.743	95.4
17	8092.781	64.425	148.6
18	8043.465	64.033	148.8
19	6583.504	52.410	105.5

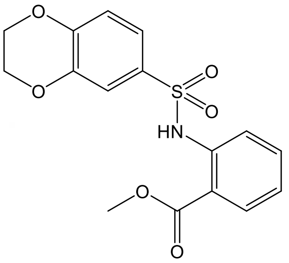

48

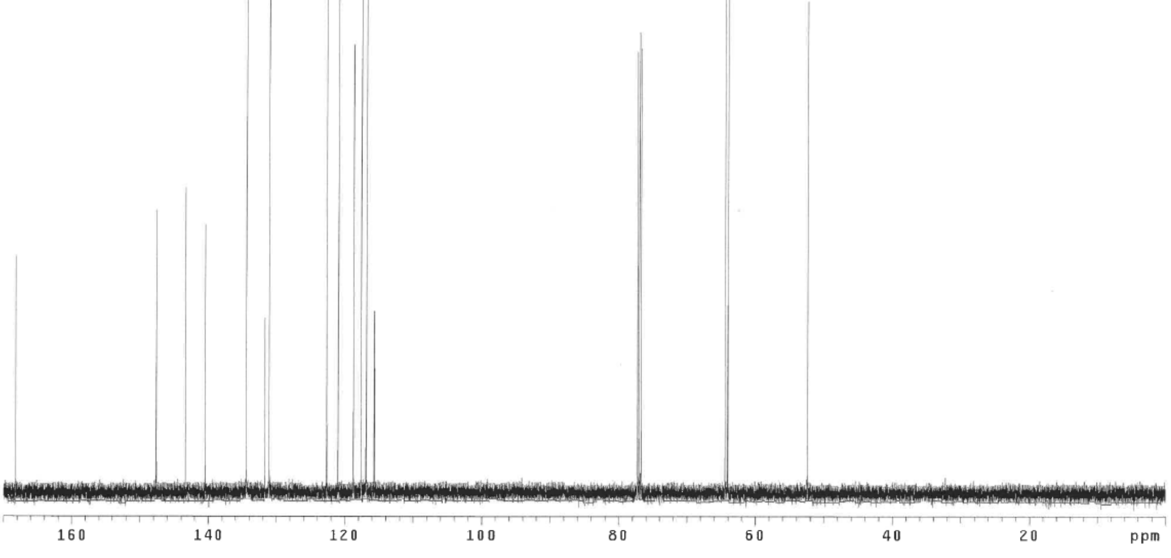

STANDARD PROTON PARAMETERS
57243
1830-BGE
Berecz Gabor
2005.03.23. (CzB)

Solvent: CDCl3
Temp. 25.0 C / 298.1 K
INOVA-500 "i500"

PULSE SEQUENCE
Relax. delay 3.000 sec
Pulse 90.0 degrees
Acq. time 5.000 sec
Width 8000.0 Hz
16 repetitions
OBSERVE H1, 499.5244681 MHz
DATA PROCESSING
FT size 131072
Total time 2 minutes

INDEX	FREQUENCY	PPM	HEIGHT
1	3916.504	7.840	22.7
2	3914.673	7.837	22.4
3	3908.813	7.825	26.4
4	3906.982	7.821	24.8
5	3718.384	7.444	11.1
6	3716.553	7.440	11.7
7	3710.815	7.429	25.7
8	3709.106	7.425	23.9
9	3703.125	7.413	22.2
10	3701.294	7.410	20.1
11	3691.040	7.389	20.4
12	3689.697	7.386	22.2
13	3683.472	7.374	28.4
14	3682.129	7.371	29.4
15	3675.903	7.359	12.3
16	3674.683	7.356	11.2
17	3628.418	7.264	51.4
18	3599.731	7.206	54.2
19	3597.534	7.202	59.6
20	3557.373	7.122	31.7
21	3555.176	7.117	28.2
22	3548.828	7.104	36.6
23	3546.631	7.100	33.8
24	3491.333	6.989	25.0
25	3490.112	6.987	26.3
26	3483.643	6.974	22.2
27	3482.300	6.971	23.0
28	3448.486	6.904	64.0
29	3439.941	6.886	55.8
30	2161.133	4.326	17.1
31	2160.278	4.325	17.8
32	2157.959	4.320	35.6
33	2156.494	4.317	25.0
34	2154.541	4.313	36.6
35	2152.832	4.310	44.9
36	2152.344	4.309	42.4
37	2141.846	4.288	44.7
38	2141.235	4.287	42.5
39	2139.526	4.283	37.2
40	2137.578	4.279	26.3
41	2136.108	4.276	34.5
42	2133.789	4.272	18.5
43	2132.935	4.270	16.1
44	1936.768	3.877	371.8
45	1625.000	3.253	371.1
46	801.147	1.604	22.1
47	-0.122	-0.000	61.7

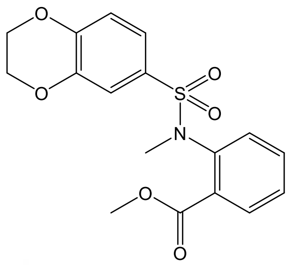

49

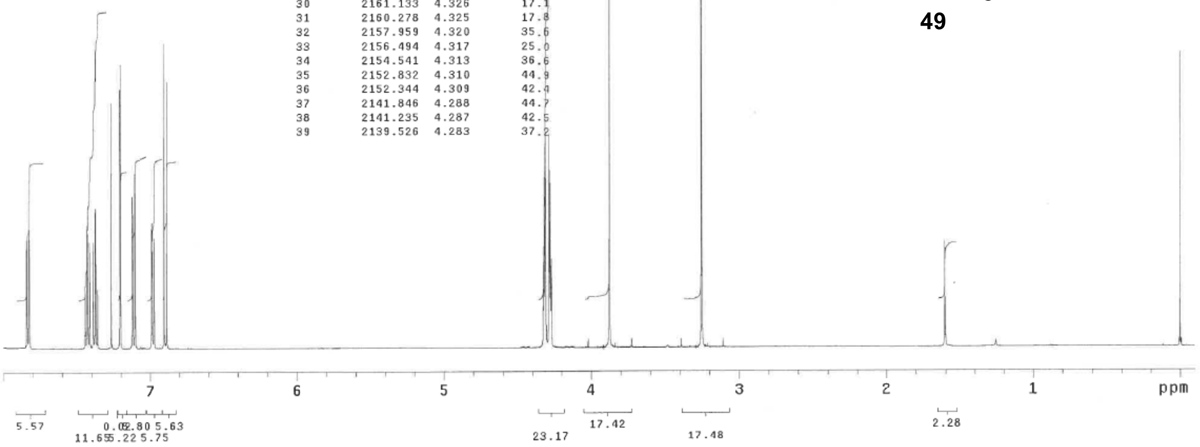

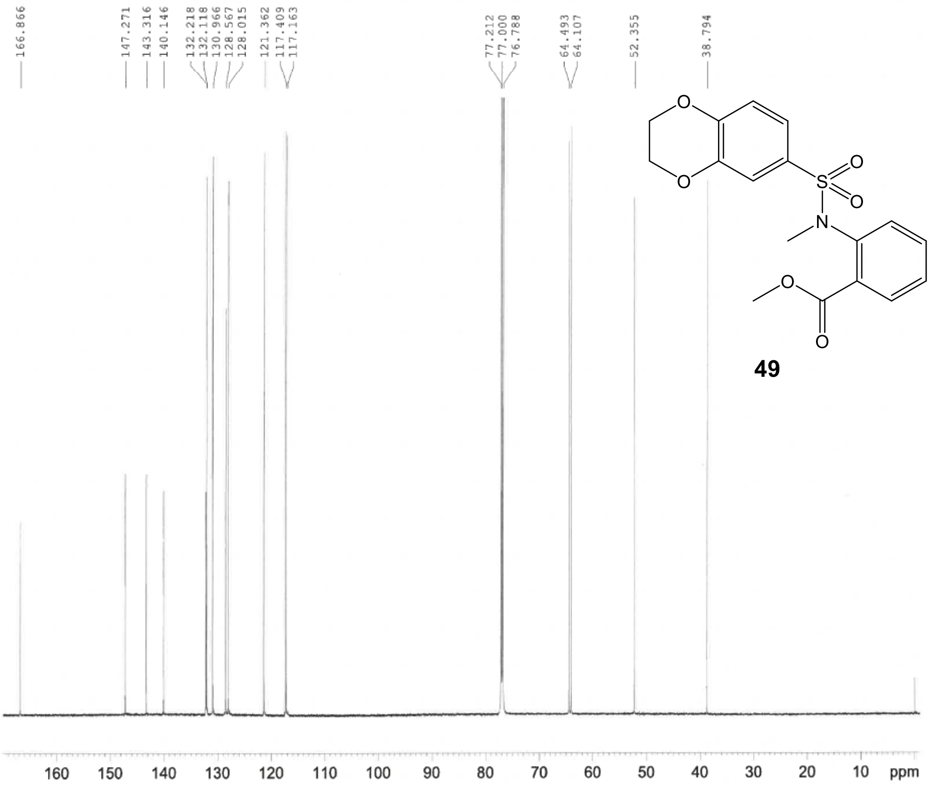

49

Standard 13C
143708
bge1830-1830-BGE_1
Berecz Gabor
2024.10.11. (KP)

Current Data Parameters
NAME 143708
EXPNO 12
PROCNO 1

F2 - Acquisition Parameters
Date_ 20241012
Time 4.08 h
INSTRUM spect
PROBHD Z145856_0002 (
PULPROG zgpg30
TD 65536
SOLVENT CDCl3
NS 2048
DS 4
SWH 36231.883 Hz
FIDRES 1.105709 Hz
AQ 0.9043968 sec
RG 196.07
DW 13.800 usec
DE 18.00 usec
TE 295.0 K
D1 1.00000000 sec
D11 0.03000000 sec
TD0 1
SFO1 150.8852070 MHz
NUC1 13C
P1 9.90 usec
PLW1 71.00000000 W
SFO2 600.0024000 MHz
NUC2 1H
CPDPRG[2 waltz16
PCPD2 80.00 usec
PLW2 32.90000153 W
PLW12 0.70370001 W
PLW13 0.35339001 W

F2 - Processing parameters
SI 131072
SF 150.8701291 MHz
WDW EM
SSB 0
LB 1.00 Hz
GB 0
PC 1.40

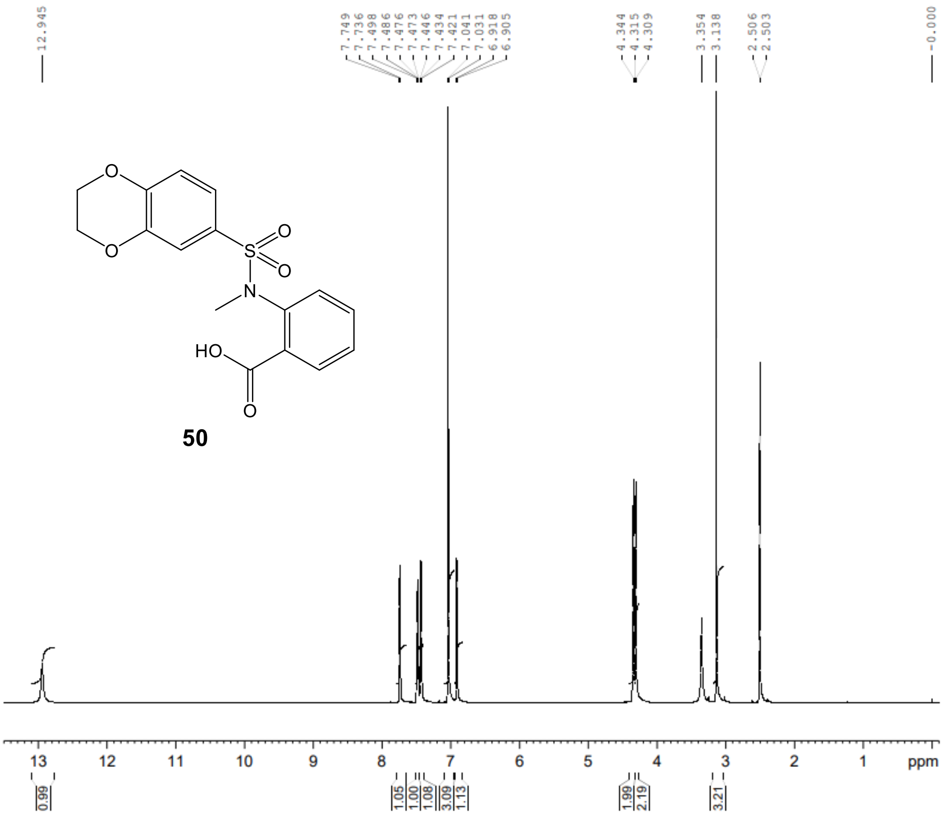

Standard 1H
144479
bge1827-1827-BGE_1
Berecz Gabor
2025.04.04. (KP)

Current Data Parameters
NAME 144479
EXPNO 11
PROCNO 1

F2 - Acquisition Parameters
Date_ 20250404
Time 19.11 h
INSTRUM spect
PROBHD Z145856_0002 (
PULPROG zg30
TD 65536
SOLVENT DMSO
NS 16
DS 2
SWH 12019.230 Hz
FIDRES 0.366798 Hz
AQ 2.7262976 sec
RG 196.07
DW 41.600 usec
DE 25.00 usec
TE 295.0 K
D1 1.00000000 sec
TD0 1
SFO1 600.0037050 MHz
NUC1 1H
P1 11.50 usec
PLW1 28.00000000 W

F2 - Processing parameters
SI 65536
SF 600.0000004 MHz
WDW EM
SSB 0
LB 0.30 Hz
GB 0
PC 1.00

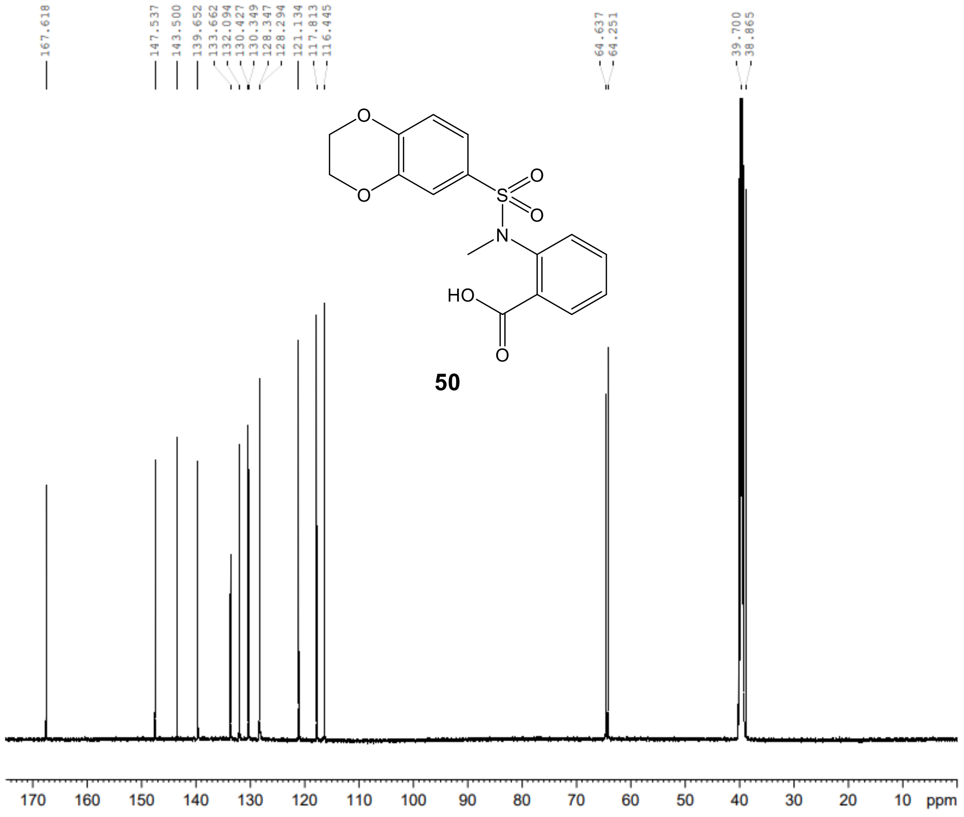

Standard 13C
144479
bge1827-1827-BGE_1
Berecz Gabor
2025.04.04. (KP)

Current Data Parameters
NAME 144479
EXPNO 12
PROCNO 1

F2 - Acquisition Parameters
Date_ 20250404
Time 20.19 h
INSTRUM spect
PROBHD Z145856_0002 (
PULPROG zgpg30
TD 65536
SOLVENT DMSO
NS 2048
DS 4
SWH 36231.883 Hz
FIDRES 1.105709 Hz
AQ 0.9043968 sec
RG 196.07
DW 13.800 usec
DE 18.00 usec
TE 295.0 K
D1 1.00000000 sec
D11 0.03000000 sec
TD0 1
SFO1 150.8852070 MHz
NUC1 13C
P1 9.80 usec
PLW1 72.69999695 W
SFO2 600.0024000 MHz
NUC2 1H
CPDPRG[2 waltz16
PCPD2 80.00 usec
PLW2 32.50000000 W
PLW12 0.70708001 W
PLW13 0.35508999 W

F2 - Processing parameters
SI 131072
SF 150.8701581 MHz
WDW EM
SSB 0
LB 1.00 Hz
GB 0
PC 1.40

50

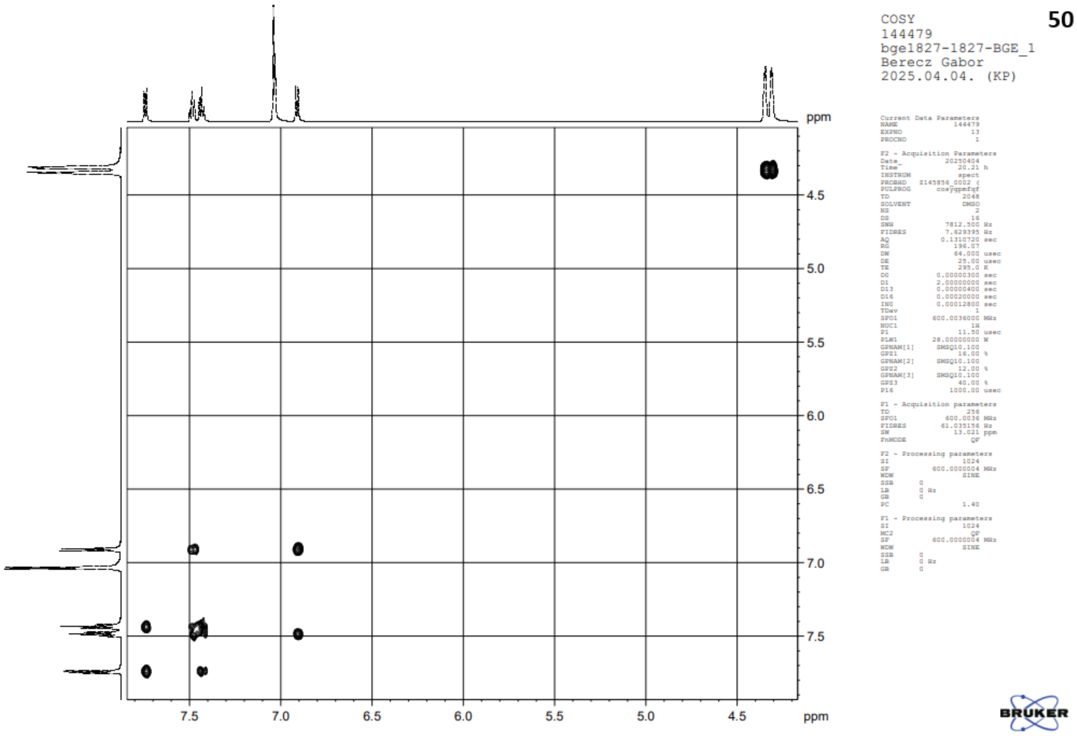
COSY
144479
bge1827-1827-BGE_1
Berecz Gabor
2025.04.04. (KP)
ppm
BRUKER

50

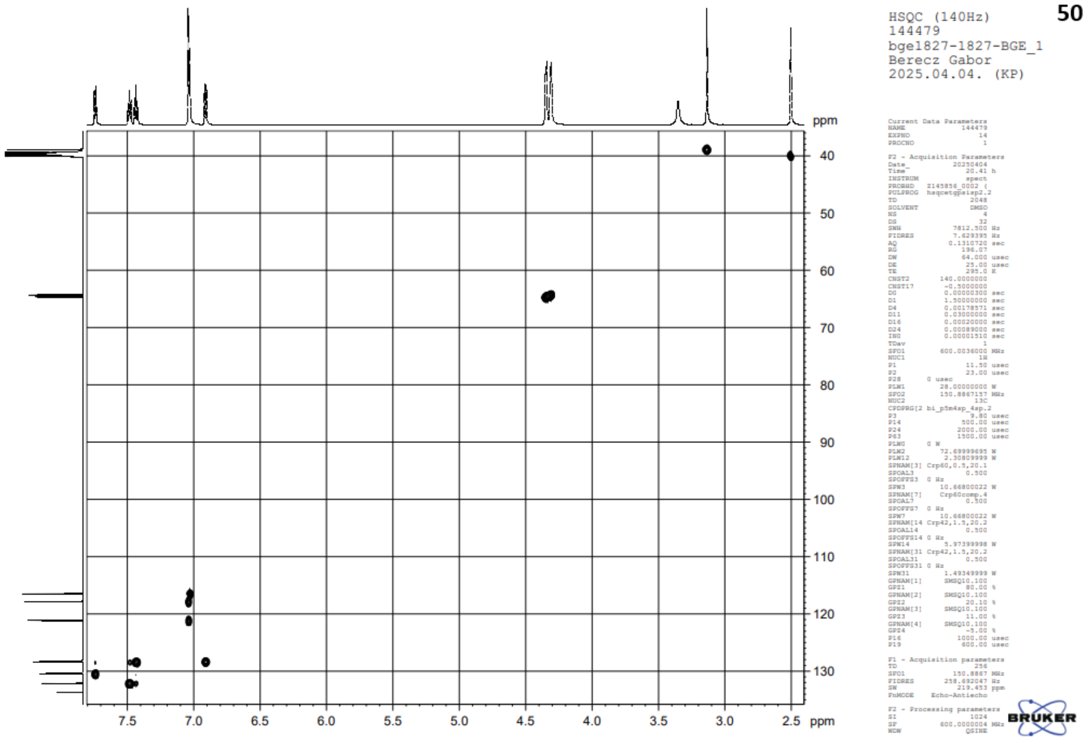
HSQC (140Hz)
144479
bge1827-1827-BGE_1
Berecz Gabor
2025.04.04. (KP)
ppm
BRUKER

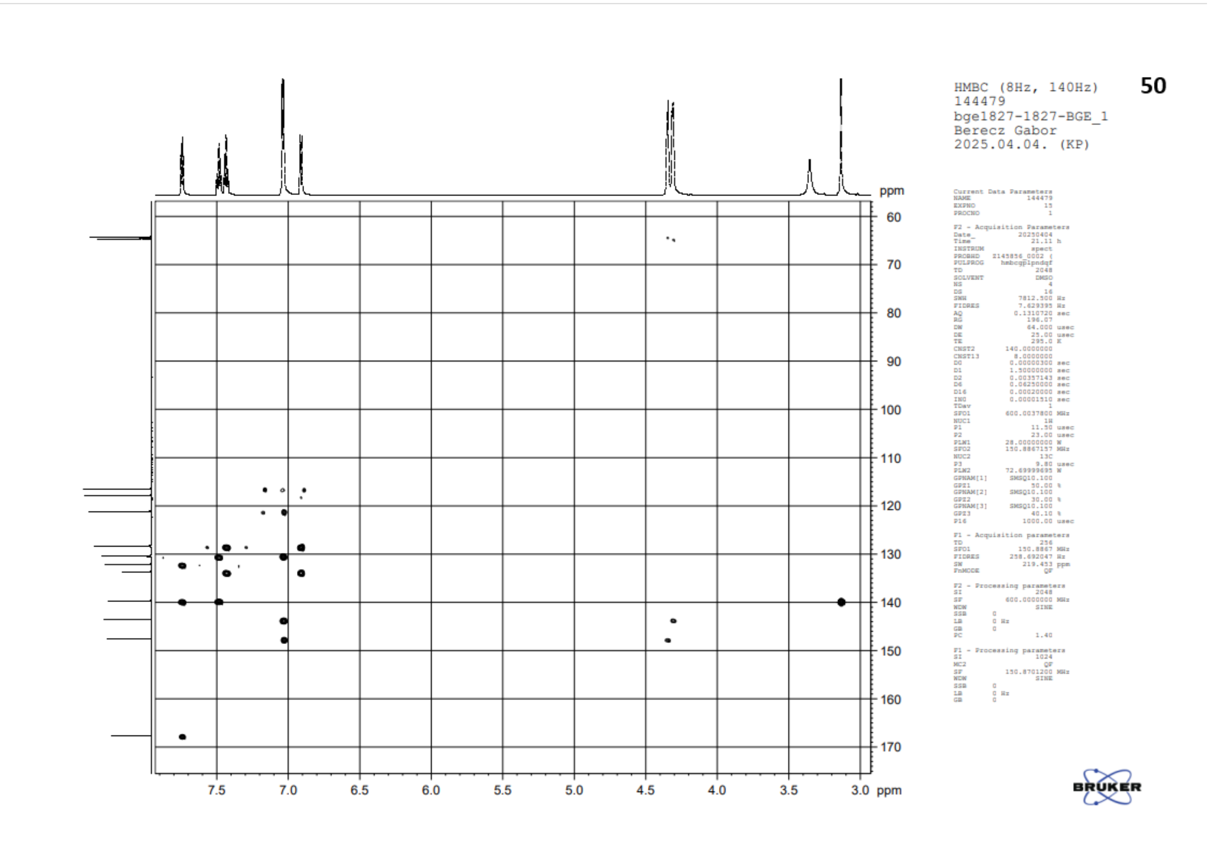

HMBC (8Hz, 140Hz)
144479
bge1827-1827-BGE_1
Berecz Gabor
2025.04.04. (KP)
ppm
60
70
80
90
100
110
120
130
140
150
160
170
7.5
7.0
6.5
6.0
5.5
5.0
4.5
4.0
3.5
3.0
ppm
BRUKER

50

STANDARD PROTON PARAMETERS
57266
1831-BGE
Berecz Gabor
2005.03.24. (DA)

Solvent: CDCl3
Temp. 25.0 C / 298.1 K
INOVA-500 "i500"

PULSE SEQUENCE
Pulse 90.0 degrees
Acq. time 5.000 sec
Width 8000.0 Hz
16 repetitions
OBSERVE H1, 499.5244689 MHz
DATA PROCESSING
FT size 131072
Total time 1 minute

INDEX	FREQUENCY	PPM	HEIGHT
1	4145.996	8.300	21.6
2	4144.531	8.297	21.7
3	4137.939	8.284	22.5
4	4136.475	8.281	21.8
5	3811.035	7.629	11.9
6	3809.448	7.626	11.9
7	3802.979	7.613	22.2
8	3802.246	7.612	21.9
9	3795.776	7.599	15.1
10	3794.189	7.596	14.2
11	3772.949	7.553	63.6
12	3734.253	7.476	65.5
13	3685.791	7.379	15.5
14	3684.814	7.377	16.5
15	3677.612	7.362	28.1
16	3667.847	7.343	32.8
17	3659.790	7.327	26.9
18	3628.540	7.264	10.8
19	2181.396	4.367	35.1
20	2179.321	4.363	35.5
21	2176.147	4.356	76.0
22	2171.631	4.347	76.4
23	2168.457	4.341	33.7
24	2166.382	4.337	34.0
25	1649.536	3.302	268.0
26	-0.000	-0.000	14.1

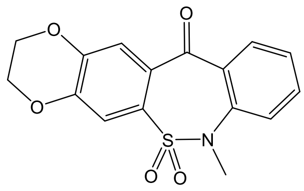

51

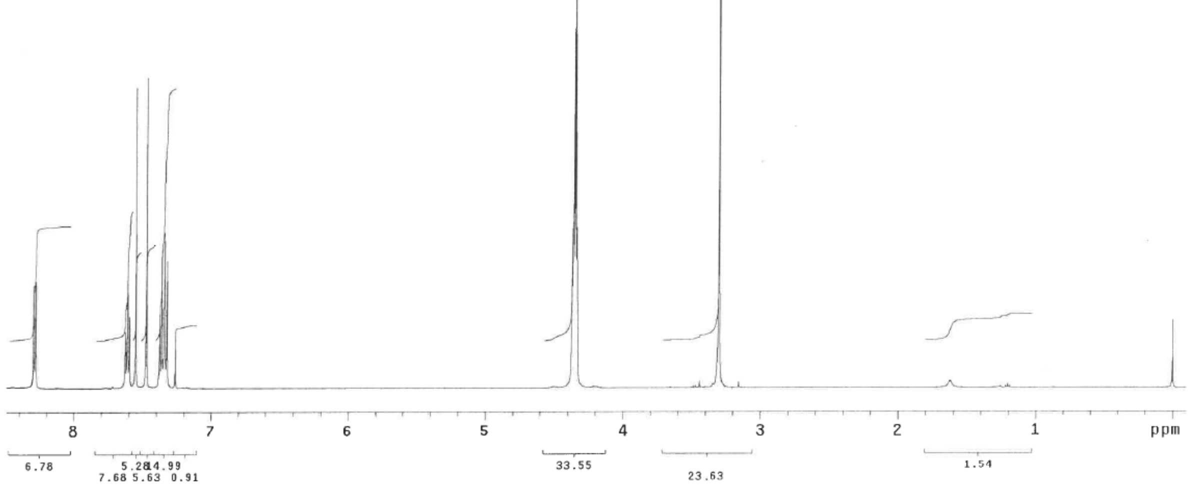

STANDARD 13C PARAMETERS
57266
1831-BGE
Berecz Gabor
2005.03.24. (DA)

Solvent: CDCl3
Temp. 25.0 C / 298.1 K
User: 1-14-87
INOVA-500 "i500"

PULSE SEQUENCE
Relax. delay 1.200 sec
Pulse 45.0 degrees
Acq. time 1.300 sec
Width 32000.0 Hz
720 repetitions
OBSERVE C13, 125.6055323 MHz
DECOUPLE H1, 499.5269647 MHz
Power 32 dB
continuously on
WALTZ-16 modulated
DATA PROCESSING
Line broadening 0.5 Hz
FT size 131072
Total time 30 minutes

INDEX	FREQUENCY	PPM	HEIGHT
1	23716.804	188.805	31.9
2	18476.570	147.089	46.0
3	18389.168	146.393	42.2
4	17751.472	141.316	49.4
5	16893.562	134.487	133.3
6	16597.664	132.131	152.1
7	16542.976	131.696	36.4
8	16382.332	130.417	34.0
9	16280.281	129.604	39.2
10	15849.617	126.176	147.8
11	15741.707	125.317	152.2
12	15239.265	121.317	155.2
13	14506.355	115.482	145.5
14	9704.109	77.253	110.3
15	9672.371	77.000	112.9
16	9640.144	76.743	116.1
17	8118.172	64.627	163.5
18	8084.480	64.359	159.7
19	4922.371	39.186	96.4

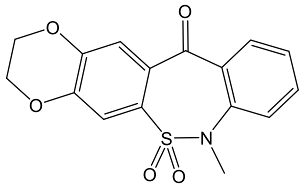

51

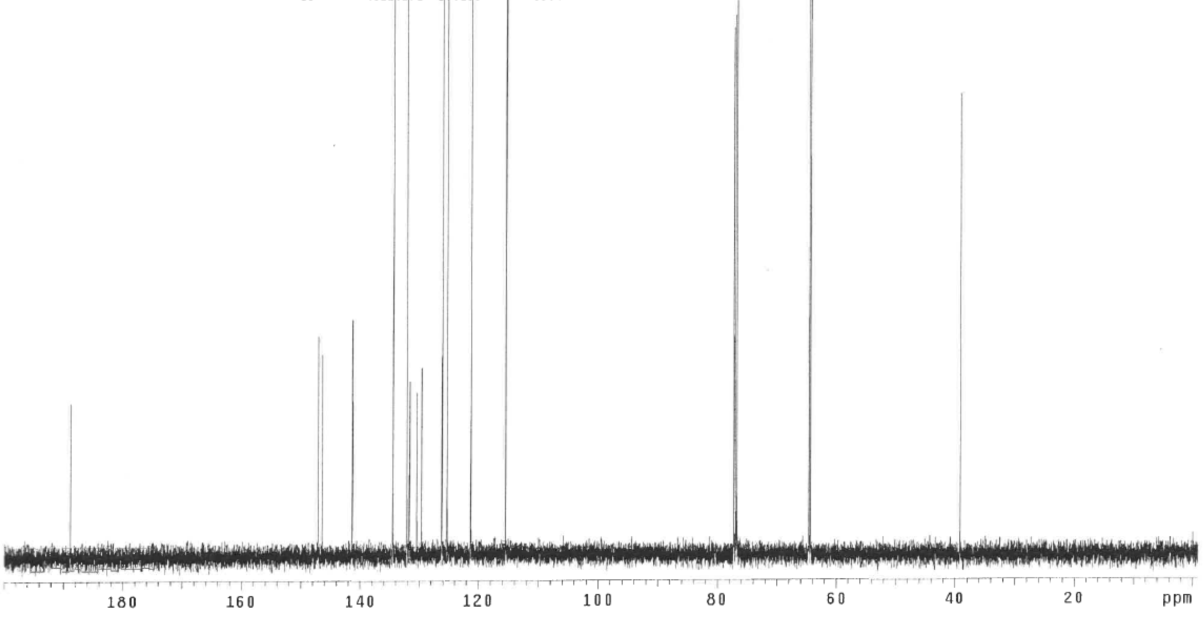

GMQFCOPS_DA
57266
1831-BGE
Berecz Gabor
2005.03.24. (DA)

Solvent: CDCl3
Temp. 25.0 C / 298.1 K
INOVA-500 "i500"

SEQUENCE: gmqfcops_da
Relax. delay 1.500 sec
Acq. time 0.128 sec
Width 8000.0 Hz
2D Width 8000.0 Hz
Single scan
2 x 256 increments
OBSERVE H1, 499.5244665 MHz
DATA PROCESSING
Sq. sine bell 0.128 sec
Shifted by -0.128 sec
F1 DATA PROCESSING
Sine bell 0.042 sec
Shifted by -0.042 sec
FT size 4096 x 512
Total time 14 minutes

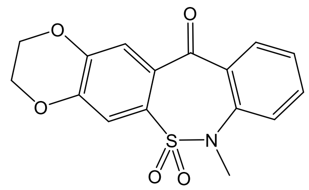

51

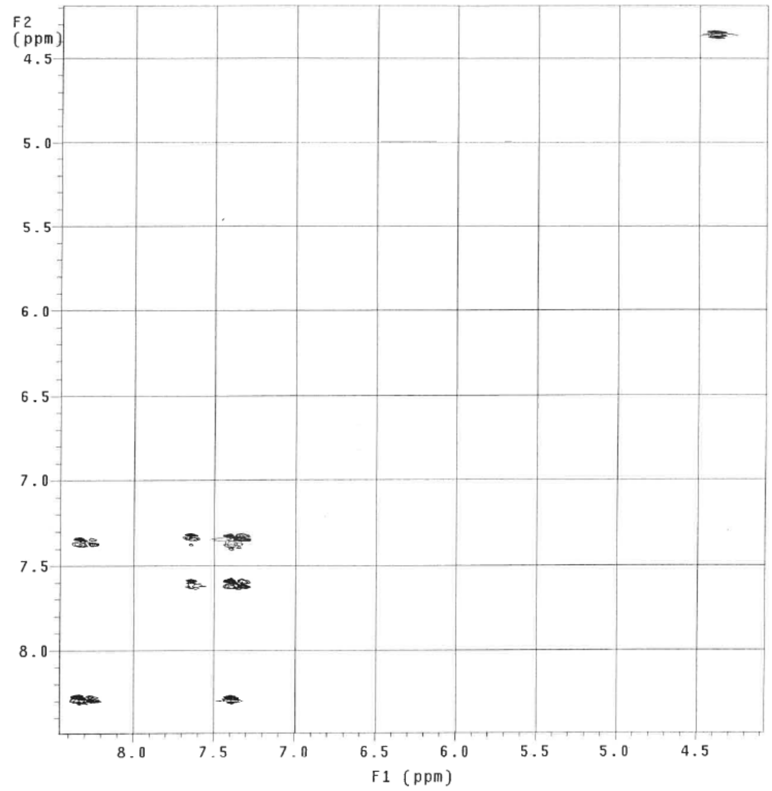

GHSQC_DA (140 Hz)
57266
1831-BGE
Berecz Gabor
2005.03.24. (DA)

Solvent: CDCl3
Temp. 25.0 C / 298.1 K
INOVA-500 "i500"

PULSE SEQUENCE: ghsqc_da
Relax. delay 1.500 sec
Acq. time 0.128 sec
Width 8000.0 Hz
2D Width 26507.6 Hz
2 repetitions
2 x 256 increments
OBSERVE H1, 499.5244697 MHz
DECOUPLE C13, 125.6176221 MHz
Power 42 dB
on during acquisition
off during delay
GARP-1 modulated
DATA PROCESSING
Sq. sine bell 0.200 sec
Shifted by -0.200 sec
F1 DATA PROCESSING
Sq. sine bell 0.005 sec
Shifted by -0.005 sec
FT size 4096 x 512
Total time 28 minutes

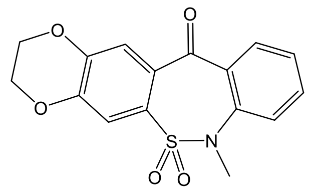

51

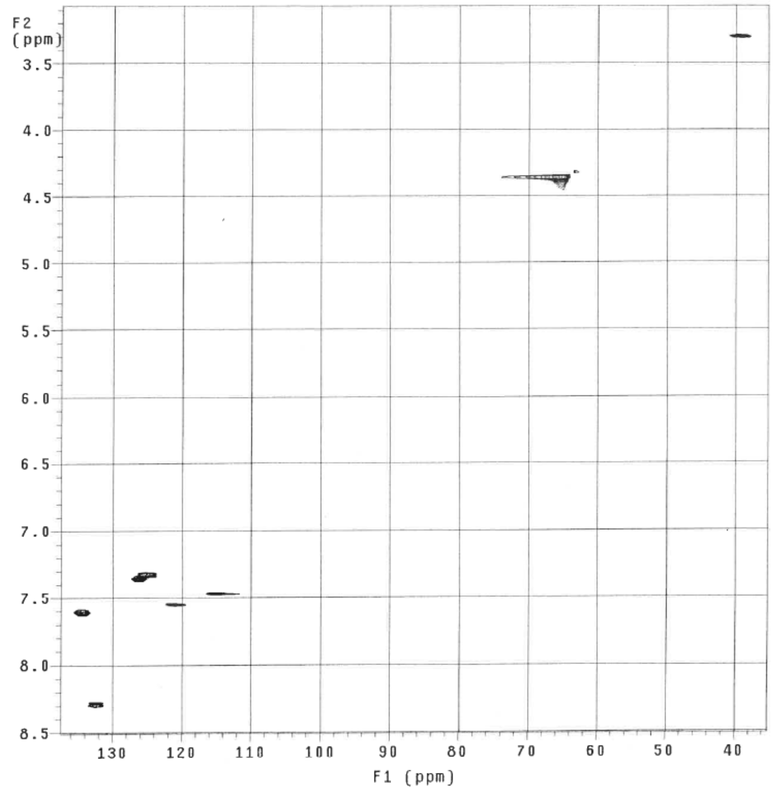

GHMQC_DA (140 Hz, 8 Hz)
57266
1831-BGE
Berecz Gabor
2005.03.24. (DA)
Solvent: CDCl3
Temp. 25.0 C / 298.1 K
INOVA-500 "i500"
PULSE SEQUENCE: ghmqc_da
Relax. delay 1.500 sec
Acq. time 0.128 sec
Width 8000.0 Hz
2D Width 26507.6 Hz
4 repetitions
256 increments
OBSERVE H1, 499.5244689 MHz
DATA PROCESSING
Sine bell 0.128 sec
F1 DATA PROCESSING
Sine bell 0.002 sec
FT size 4096 x 512
Total time 28 minutes

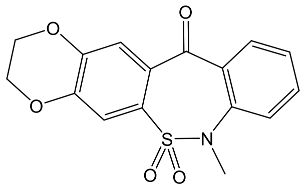

51

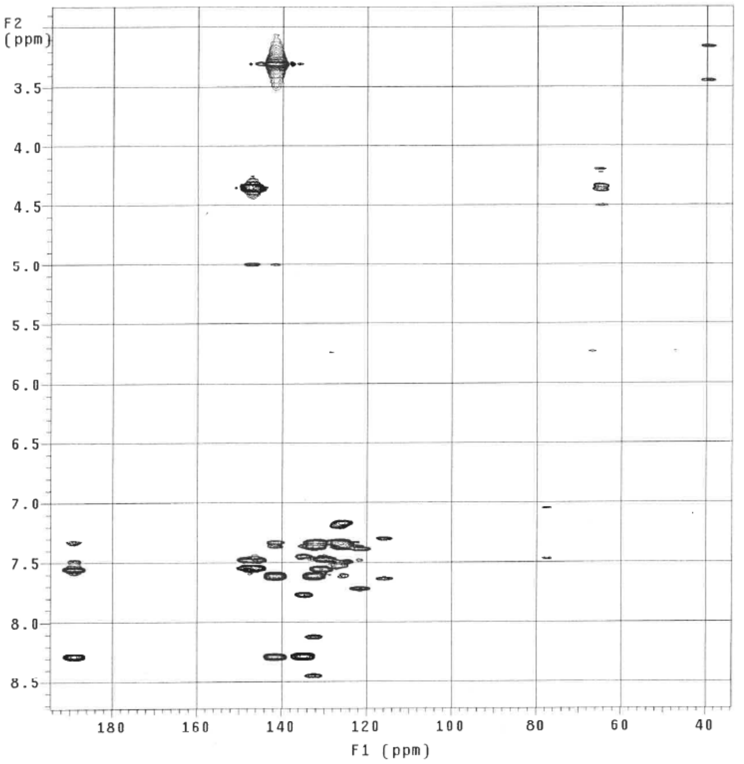

DPFGNOE_DA (3.30 ppm)
57266
1831-BGE
Berecz Gabor
2005.03.24. (DA)
Solvent: CDCl3
Temp. 25.0 C / 298.1 K
INOVA-500 "i500"
PULSE SEQUENCE: dpfgnoe_da
Relax. delay 2.000 sec
Pulse 102.9 degrees
Mixing 2.000 sec
Acq. time 5.000 sec
Width 8000.0 Hz
204 repetitions
OBSERVE H1, 499.5244689 MHz
DATA PROCESSING
Line broadening 1.0 Hz
FT size 131072
Total time 30 minutes

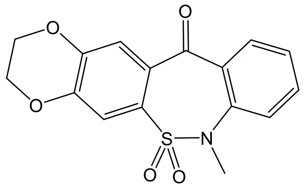

51

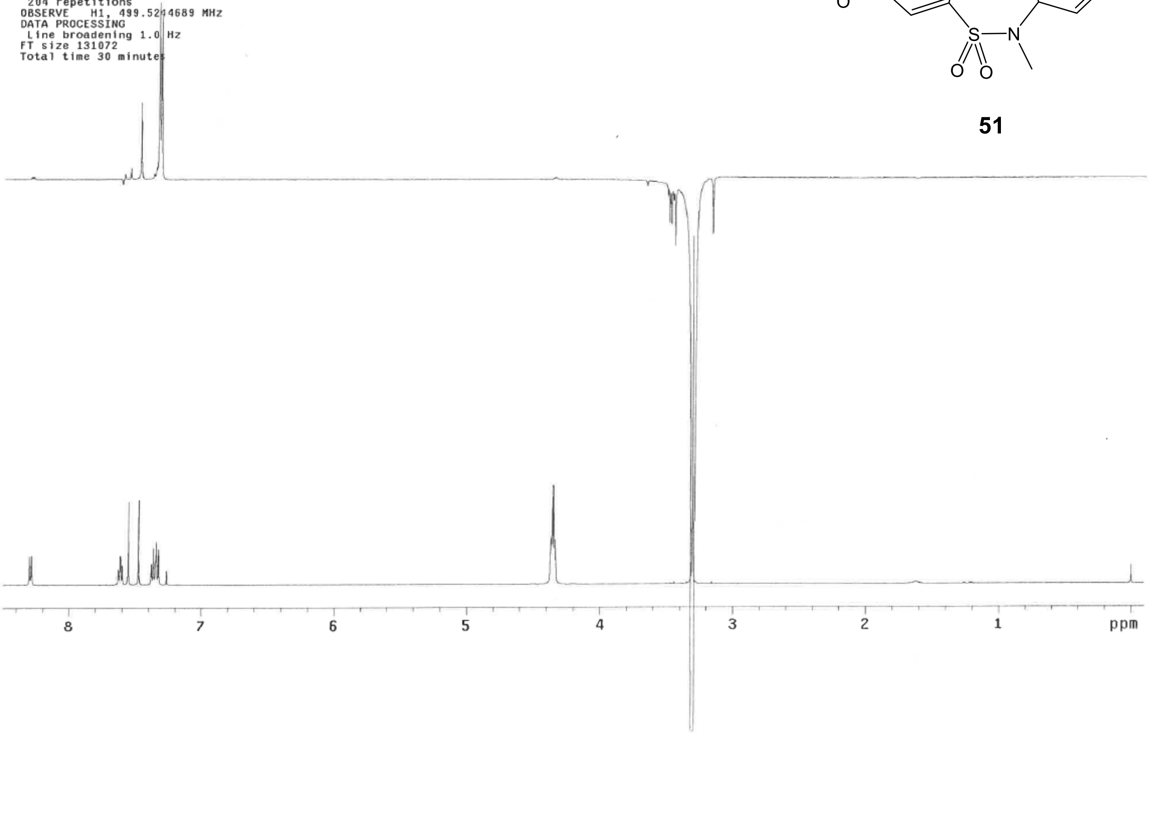

STANDARD 1H
58946
1868-BGE
Berecz Gabor
2005/07/12 (SzA)

Solvent: DMSO
Temp. 25.0 C / 298.1 K
Sample #1
INOVA-500 "i500"

PULSE SEQUENCE
Relax. delay 3.000 sec
Pulse 6.4 usec
Acq. time 5.000 sec
Width 8000.0 Hz
16 repetitions
OBSERVE H1, 499.5268368 MHz
DATA PROCESSING
FT size 131072
Total time 2 minutes

INDEX	FREQUENCY	PPM	HEIGHT
1	3788.818	7.585	8.3
2	3787.598	7.582	6.7
3	3786.743	7.581	8.8
4	3781.860	7.571	10.0
5	3781.250	7.570	10.2
6	3779.541	7.566	10.0
7	3733.643	7.474	10.5
8	3731.934	7.471	13.8
9	3726.440	7.460	9.7
10	3725.830	7.459	11.1
11	3724.487	7.456	15.1
12	3684.570	7.376	5.0
13	3679.077	7.365	12.3
14	3677.124	7.361	11.8
15	3671.753	7.350	21.1
16	3669.556	7.346	21.7
17	3664.062	7.335	13.2
18	3662.476	7.332	12.1
19	3656.738	7.320	4.3
20	3637.817	7.283	34.1
21	3636.963	7.281	32.9
22	3592.163	7.191	56.8
23	3219.971	6.446	29.9
24	3214.844	6.436	31.0
25	3087.891	6.182	13.8
26	3082.764	6.171	13.3
27	2157.959	4.320	6.1
28	2154.419	4.313	12.0
29	2152.710	4.309	6.8
30	2150.024	4.304	17.0
31	2149.170	4.302	14.8
32	2147.217	4.299	13.7
33	2134.888	4.274	16.7
34	2133.911	4.272	15.4
35	2132.446	4.269	13.6
36	2130.737	4.266	11.9
37	2127.808	4.260	13.7
38	2124.756	4.254	6.1
39	1670.288	3.344	71.4 ← w
40	1656.982	3.317	162.0
41	1247.681	2.498	4.9
42	1033.447	2.069	7.1
43	519.165	1.039	7.3
44	513.062	1.027	7.1

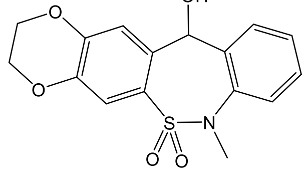

52

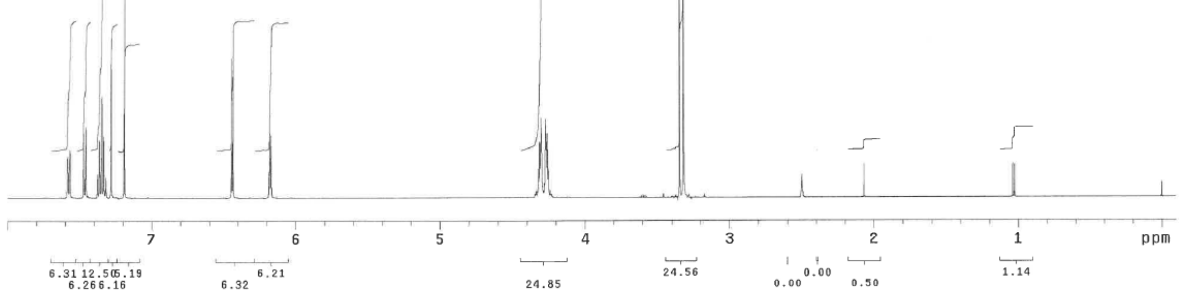

STANDARD 13C
58946
1868-BGE
Berecz Gabor
2005/07/12 (SzA)

Solvent: DMSO
Temp. 25.0 C / 298.1 K
Sample #1, user 1-14-87
INOVA-500 "i500"

PULSE SEQUENCE
Relax. delay 3.000 sec
Pulse 6.0 usec
Acq. time 1.300 sec
Width 32000.0 Hz
192 repetitions
OBSERVE C13, 125.6061582 MHz
DECOUPLE H1, 499.5293374 MHz
Power 32 dB
continuously on
WALTZ-16 modulated
DATA PROCESSING
Line broadening 1.0 Hz
FT size 131072
Total time 13 minutes

INDEX	FREQUENCY	PPM	HEIGHT
1	18407.173	146.541	60.8
2	17989.204	143.213	51.6
3	17920.356	142.665	58.0
4	17199.165	136.924	55.4
5	16945.259	134.903	60.1
6	16294.868	129.725	49.8
7	16167.427	128.710	68.3
8	16083.442	128.042	68.3
9	16021.430	127.548	70.7
10	15779.731	125.624	66.8
11	14586.860	116.127	72.8
12	14366.645	114.374	75.4
13	8503.852	67.700	78.1
14	8132.759	64.746	67.1
15	8083.930	64.357	65.6
16	5049.751	40.201	33.0
17	5028.755	40.034	100.5
18	5008.247	39.871	199.2
19	4986.762	39.700	238.6
20	4965.766	39.533	200.8
21	4944.770	39.366	101.1
22	4924.262	39.202	33.6
23	4656.196	37.068	96.7

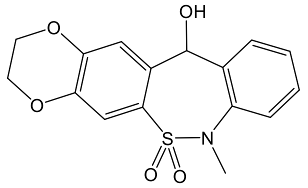

52

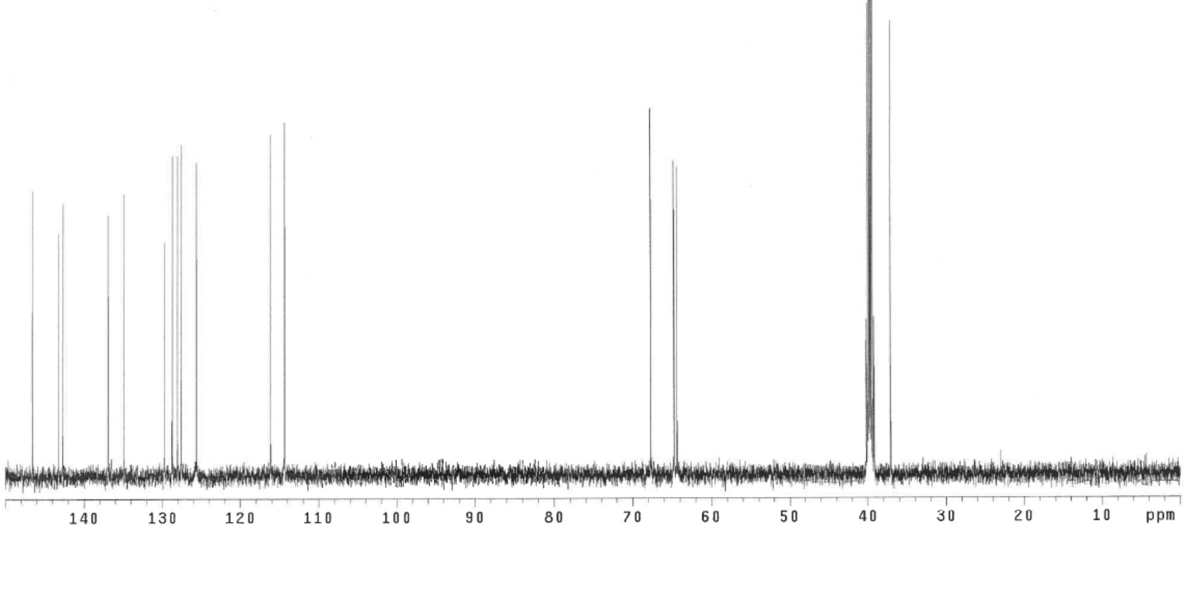

STANDARD 1H
59214
1879-BGE
Berecz Gabor
2005.08.04. (CzB)
Solvent: CDCl3
Temp. 25.0 C / 298.1 K
INOVA-500 "i500"
PULSE SEQUENCE
Relax. delay 3.000 sec
Pulse 6.4 usec
Acq. time 5.000 sec
Width 8000.0 Hz
16 repetitions
OBSERVE H1, 499.5244724 MHz
DATA PROCESSING
FT size 131072
Total time 2 minutes

INDEX	FREQUENCY	PPM	HEIGHT
1	3752.930	7.513	6.0
2	3749.390	7.506	41.7
3	3744.385	7.496	15.4
4	3742.798	7.493	14.8
5	3739.380	7.486	9.8
6	3737.915	7.483	10.3
7	3732.422	7.472	10.4
8	3730.957	7.469	11.5
9	3724.487	7.456	4.4
10	3723.022	7.453	5.1
11	3699.219	7.405	8.0
12	3697.876	7.403	8.2
13	3691.650	7.390	13.0
14	3690.186	7.387	11.3
15	3664.062	7.335	8.7
16	3662.476	7.332	8.6
17	3656.982	7.321	8.8
18	3656.494	7.320	9.3
19	3655.396	7.318	9.5
20	3654.907	7.317	9.1
21	3649.292	7.306	5.3
22	3647.705	7.302	5.5
23	3625.610	7.258	17.2
24	3567.261	7.141	3.1
25	3512.939	7.033	39.4
26	3024.902	6.056	33.0
27	2865.479	5.736	2.0
28	2160.645	4.325	2.6
29	2158.813	4.322	2.2
30	2157.227	4.319	3.4
31	2154.419	4.313	4.9
32	2152.832	4.310	4.1
33	2149.170	4.302	11.8
34	2146.973	4.298	6.2
35	2144.531	4.293	18.0
36	2142.578	4.289	25.0
37	2141.357	4.287	18.8
38	2138.428	4.281	39.1
39	2135.864	4.276	11.4
40	2134.155	4.272	8.3
41	2130.859	4.266	3.1
42	2129.761	4.264	2.2
43	2126.587	4.257	1.8
44	1769.165	3.542	162.0
45	1555.176	3.113	13.5
46	3.174	0.006	2.2
47	-0.000	-0.000	84.4
48	-3.418	-0.007	2.3

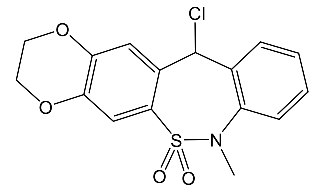

53

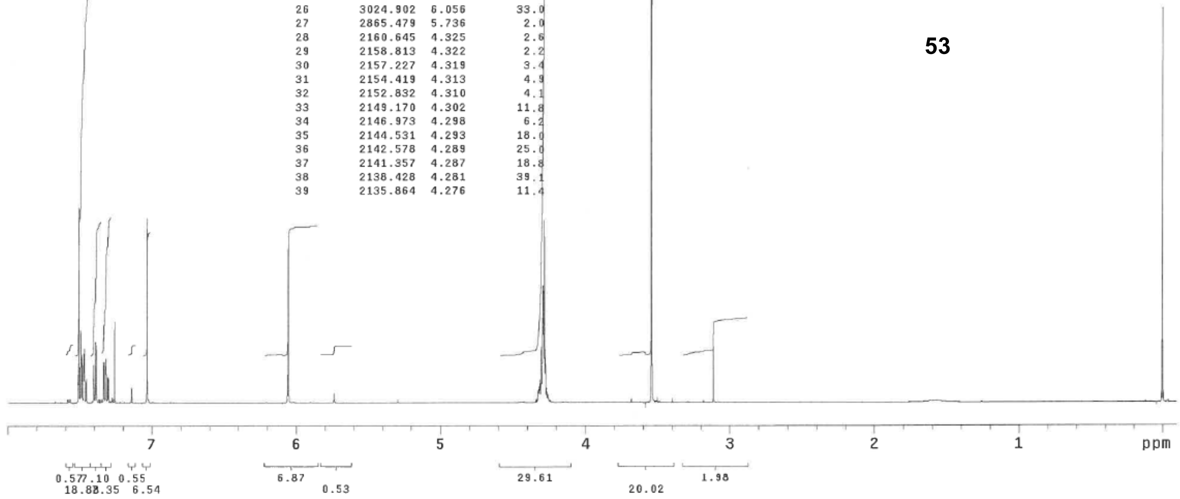

STANDARD 13C
59214
1879-BGE
Berecz Gabor
2005.08.04. (CzB)
Solvent: CDCl3
Temp. 25.0 C / 298.1 K
User: 1-14-87
INOVA-500 "i500"
PULSE SEQUENCE
Relax. delay 3.000 sec
Pulse 6.0 usec
Acq. time 1.300 sec
Width 32000.0 Hz
864 repetitions
OBSERVE C13, 125.6055298 MHz
DECOUPLE H1, 499.5259647 MHz
Power 32 dB
continuously on
WALTZ-16 modulated
DATA PROCESSING
Line broadening 1.0 Hz
FT size 131072
Total time 61 minutes

INDEX	FREQUENCY	PPM	HEIGHT
1	18367.195	146.218	64.2
2	18151.374	144.500	57.2
3	17507.820	139.377	52.4
4	17320.808	137.888	55.9
5	16710.945	133.033	46.7
6	16621.589	(132.321 imp	8.3)
7	16475.105	131.155	144.0
8	16297.859	129.744	137.3
9	16230.964	129.212	156.0
10	16150.886	128.574	64.8
11	16140.144	128.489	151.7
12	15987.312	(127.272 imp	13.2)
13	15939.949	(126.895 imp	14.8)
14	15045.906	119.778	153.2
15	14848.152	(118.203 imp	11.4)
16	14728.523	117.251	149.8
17	9704.109	77.253	417.3
18	9672.371	77.000	431.8
19	9640.632	76.747	421.6
20	8109.382	64.557	155.0
21	8084.480	64.359	158.1
22	8075.203	(64.285 imp	15.5)
23	8057.624	64.145	129.4
24	4975.105	(39.606 imp	7.7)
25	4886.238	38.898	113.5
26	-2.922	-0.023	45.0

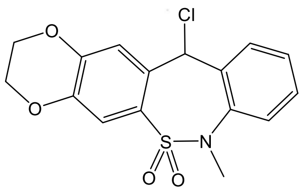

53

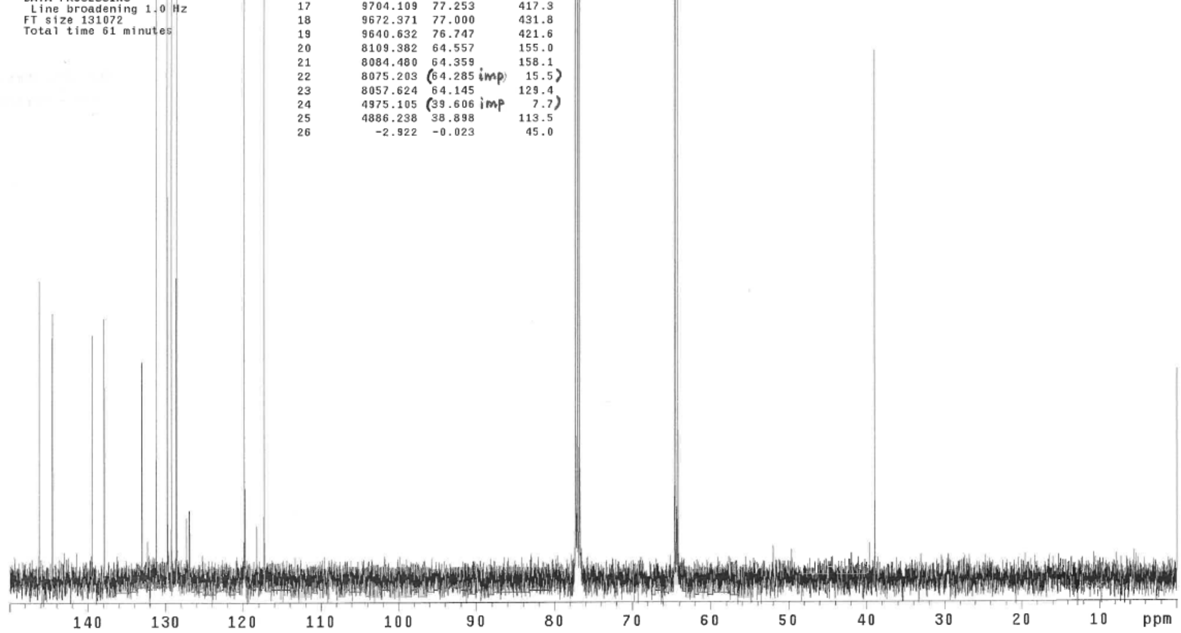

STANDARD 1H
59373
1883-BGE
Berecz Gabor
2005.08.24. (CzB)

Solvent: CDCl3
Temp. 25.0 C / 298.1 K
INOVA-500 "i500"

PULSE SEQUENCE
Pulse 6.4 usec
Acq. time 5.000 sec
Width 8000.0 Hz
16 repetitions
OBSERVE H1, 499.5244696 MHz
DATA PROCESSING
FT size 131072
Total time 1 minute

INDEX	FREQUENCY	PPM	HEIGHT
1	3752.930	7.513	38.1
2	3694.214	7.395	11.7
3	3687.012	7.381	13.0
4	3686.523	7.380	12.9
5	3661.377	7.330	27.9
6	3660.522	7.328	23.9
7	3658.569	7.324	20.1
8	3655.518	7.318	15.6
9	3654.175	7.315	12.2
10	3633.911	7.275	9.3
11	3631.104	7.269	7.2
12	3628.052	7.263	15.4
13	3626.343	7.260	8.8
14	3625.244	7.257	6.7
15	3623.779	7.254	6.1
16	3620.239	7.247	5.0
17	3617.676	7.242	4.4
18	3475.830	6.958	41.3
19	2388.428	4.781	26.5
20	2150.391	4.305	6.6
21	2146.973	4.298	17.0
22	2144.531	4.293	14.8
23	2143.066	4.290	19.1
24	2141.113	4.286	31.7
25	2134.399	4.273	17.9
26	2133.057	4.270	24.7
27	2132.080	4.268	25.7
28	2129.883	4.264	14.9
29	2128.418	4.261	15.3
30	2126.221	4.256	9.2
31	2125.122	4.254	7.1
32	1634.155	3.271	151.2
33	1173.584	2.349	131.4
34	-0.000	-0.000	12.9

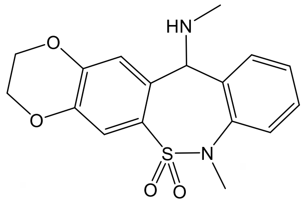

54a

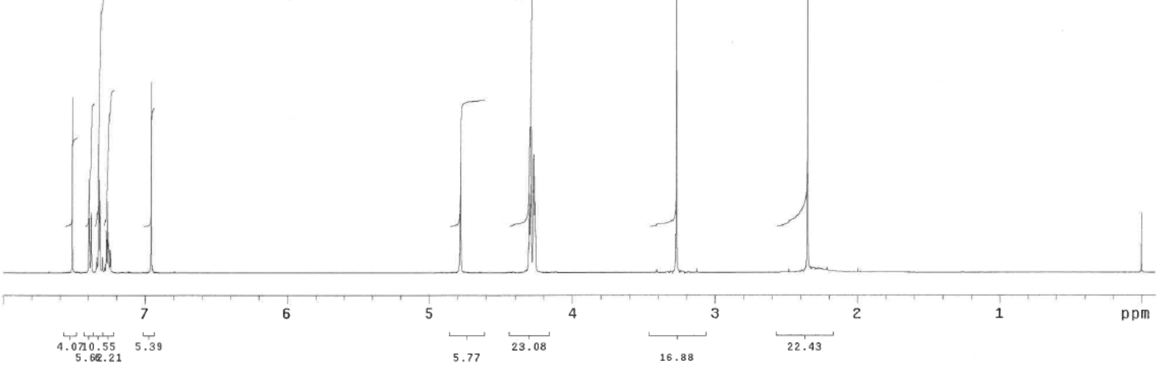

STANDARD 13C
59373
1883-BGE
Berecz Gabor
2005.08.24. (CzB)

Solvent: CDCl3
Temp. 25.0 C / 298.1 K
User: 1-14-87
INOVA-500 "i500"

PULSE SEQUENCE
Relax. delay 3.000 sec
Pulse 12.0 usec
Acq. time 1.300 sec
Width 32000.0 Hz
736 repetitions
OBSERVE C13, 125.6055318 MHz
DECOUPLE H1, 499.5269647 MHz
Power 32 dB
continuously on
WALTZ-16 modulated
DATA PROCESSING
Line broadening 0.5 Hz
FT size 131072
Total time 52 minutes

INDEX	FREQUENCY	PPM	HEIGHT
1	18377.449	146.300	33.0
2	17918.953	142.650	30.7
3	17484.871	139.194	26.6
4	17344.246	138.074	12.8
5	16558.113	131.816	17.2
6	16389.168	130.471	20.2
7	16381.355	130.409	27.2
8	16190.437	128.889	94.6
9	16015.632	127.498	116.7
10	16013.679	127.482	112.0
11	14913.093	118.720	32.5
12	14859.871	118.297	110.0
13	9704.109	77.253	64.4
14	9672.371	77.000	66.1
15	9640.144	76.743	65.8
16	8537.605	67.966	17.7
17	8115.730	64.608	109.9
18	8063.972	64.196	107.1
19	4842.781	38.553	43.5
20	4371.101	34.798	104.6

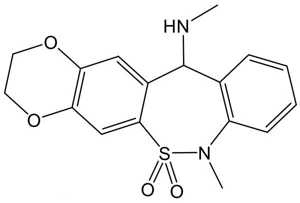

54a

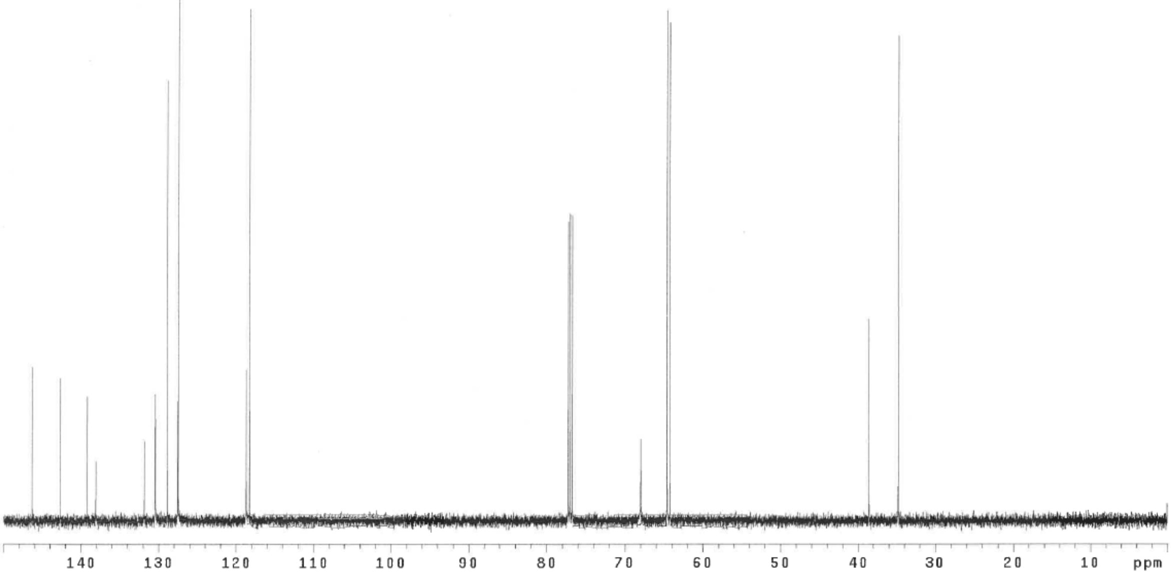

STANDARD 1H
59234
1880-BGE
Berecz Gabor
2005.08.09. (CzB)
Solvent: CDCl3
Temp. 25.0 C / 298.1 K
File: 59234ih
INOVA-500 "i500"
PULSE SEQUENCE
Pulse 6.4 usec
Acq. time 5.000 sec
Width 8000.0 Hz
16 repetitions
OBSERVE H1, 499.5244702 MHz
DATA PROCESSING
FT size 131072
Total time 1 minute

INDEX	FREQUENCY	PPM	HEIGHT	INDEX	FREQUENCY	PPM	HEIGHT
1	3729.126	7.465	31.3	40	1121.216	2.245	22.4
2	3716.675	7.440	8.0	41	850.586	1.703	11.8
3	3715.454	7.438	7.6	42	848.511	1.699	14.6
4	3708.862	7.425	11.7	43	845.947	1.694	18.2
5	3707.764	7.423	10.6	44	843.994	1.690	32.8
6	3676.758	7.361	5.8	45	841.919	1.685	16.8
7	3674.927	7.357	6.2	46	839.722	1.681	13.5
8	3669.434	7.346	9.2	47	837.524	1.677	10.1
9	3667.725	7.342	10.1	48	798.340	1.598	3.3
10	3661.743	7.330	5.4	49	0.000	0.000	14.8
11	3660.034	7.327	6.1				
12	3648.682	7.304	6.4				
13	3646.973	7.301	6.9				
14	3641.113	7.289	13.6				
15	3639.404	7.286	10.4				
16	3626.953	7.261	20.6				
17	3625.610	7.258	9.4				
18	3619.507	7.246	10.5				
19	3618.286	7.243	9.7				
20	3612.061	7.231	4.2				
21	3610.718	7.228	3.8				
22	3440.674	6.888	33.1				
23	2142.822	4.290	4.1				
24	2139.771	4.284	4.8				
25	2136.719	4.278	5.8				
26	2131.714	4.267	10.4				
27	2129.883	4.264	4.3				
28	2127.686	4.259	7.9				
29	2124.512	4.253	3.5				
30	2122.070	4.248	6.0				
31	2120.361	4.245	10.0				
32	2119.263	4.243	12.5				
33	2115.601	4.235	21.5				
34	2113.647	4.231	32.8				
35	2111.328	4.227	16.9				
36	2108.154	4.220	4.7				
37	2106.689	4.217	4.0				
38	2051.514	4.107	13.9				
39	1747.925	3.499	143.3				

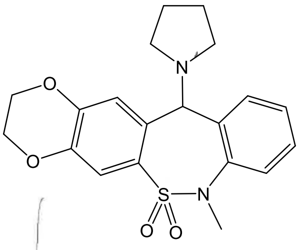

54b

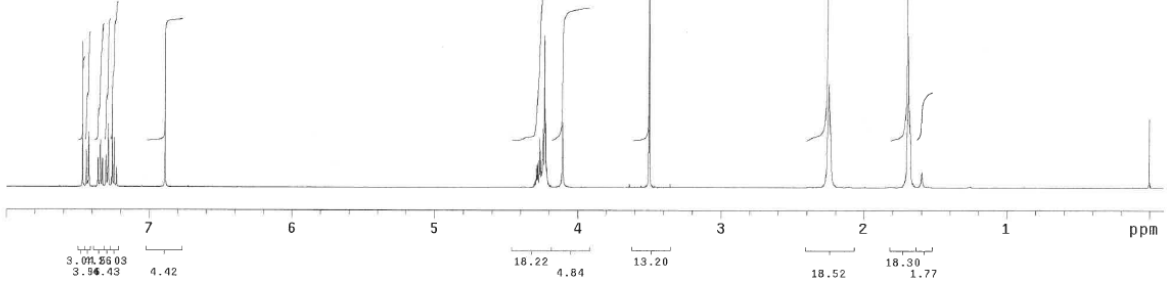

STANDARD 13C
59234
1880-BGE
Berecz Gabor
2005.08.09. (CzB)
Solvent: CDCl3
Temp. 25.0 C / 298.1 K
User: 1-14-87
INOVA-500 "i500"
PULSE SEQUENCE
Relax. delay 3.000 sec
Pulse 6.0 usec
Acq. time 1.300 sec
Width 32000.0 Hz
10240 repetitions
OBSERVE C13, 125.6055308 MHz
DECOUPLE H1, 499.5269647 MHz
Power 32 dB
continuously on
WALTZ-16 modulated
DATA PROCESSING
Line broadening 1.0 Hz
FT size 131072
Total time 12.2 hours

INDEX	FREQUENCY	PPM	HEIGHT
1	18259.285	145.359	29.7
2	18019.539	143.450	31.7
3	17859.382	142.175	33.3
4	17367.683	138.261	31.1
5	16798.835	133.733	6.6
6	16525.886	131.560	10.1
7	16377.937	130.382	27.8
8	16296.882	129.737	38.6
9	16268.562	129.511	58.2
10	16127.937	128.392	68.4
11	14982.429	119.272	26.8
12	14760.261	117.504	70.8
13	9704.109	77.253	196.6
14	9672.371	77.000	203.0
15	9640.144	76.743	196.9
16	9521.980	75.803	11.1
17	8113.289	64.588	78.2
18	8065.437	64.207	77.4
19	6742.195	53.673	157.3
20	4954.597	39.443	23.7
21	2921.394	23.257	166.2
22	-3.899	-0.031	4.5

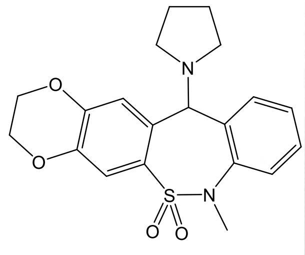

54b

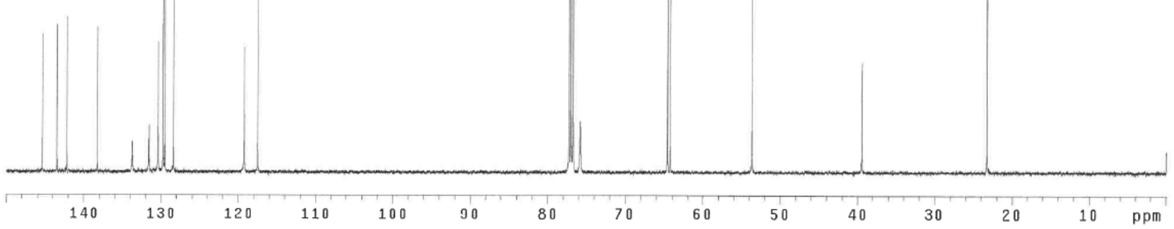

STANDARD 1H
62179
1939-BGE
Berecz Gabor
2006.02.24. (CzB)
Solvent: CDCl3
Temp. 25.0 C / 298.1 K
INOVA-500 "i500"
PULSE SEQUENCE
Relax. delay 3.000 sec
Pulse 6.4 usec
Acq. time 5.000 sec
Width 8000.0 Hz
16 repetitions
OBSERVE H1, 499.5244668 MHz
DATA PROCESSING
FT size 131072
Total time 2 minutes

INDEX	FREQUENCY	PPM	HEIGHT	INDEX	FREQUENCY	PPM	HEIGHT
1	3742.798	7.493	58.6	40	1326.172	2.655	2.9
2	3686.035	7.379	11.4	41	1324.341	2.651	3.0
3	3684.448	7.376	13.9	42	1320.068	2.643	5.4
4	3682.007	7.371	11.7	43	1314.575	2.632	7.0
5	3678.467	7.364	14.8	44	1312.866	2.628	7.4
6	3676.880	7.361	17.4	45	1307.495	2.617	9.2
7	3675.537	7.358	22.0	46	1302.979	2.608	8.4
8	3674.194	7.355	20.4	47	1297.485	2.597	6.1
9	3662.964	7.333	9.2	48	1291.504	2.585	3.2
10	3661.377	7.330	9.2	49	1271.484	2.545	4.2
11	3655.640	7.318	15.3	50	1266.357	2.535	4.1
12	3654.175	7.315	13.0	51	1264.038	2.530	3.1
13	3647.705	7.302	7.9	52	1259.155	2.521	8.8
14	3646.240	7.299	6.6	53	1254.028	2.510	8.0
15	3630.981	7.269	11.6	54	1251.831	2.506	6.2
16	3628.540	7.264	11.4	55	1246.582	2.496	4.8
17	3627.075	7.261	11.0	56	1236.816	2.476	6.5
18	3621.094	7.249	15.1	57	1231.323	2.465	12.2
19	3619.629	7.246	14.5	58	1225.708	2.454	6.3
20	3613.770	7.234	6.1	59	1218.994	2.440	6.0
21	3612.305	7.231	5.7	60	1213.623	2.430	2.5
22	3479.248	6.965	46.0	61	1194.580	2.391	2.9
23	2459.229	4.923	15.0	62	1190.308	2.383	4.7
24	2144.897	4.294	7.2	63	1183.350	2.369	11.4
25	2141.479	4.287	17.9	64	1178.711	2.360	18.3
26	2138.550	4.281	17.9	65	1174.072	2.350	17.0
27	2137.329	4.279	20.5	66	1160.645	2.323	4.0
28	2135.132	4.274	25.4	67	993.896	1.990	4.9
29	2125.610	4.255	35.0	68	-0.000	-0.000	12.2
30	2122.192	4.248	21.1				
31	2118.530	4.241	10.1				
32	1846.313	3.696	2.4				
33	1839.111	3.682	22.6				
34	1838.135	3.680	23.0				
35	1834.717	3.673	36.2				
36	1833.496	3.670	35.4				
37	1829.956	3.663	23.7				
38	1677.612	3.358	171.9				
39	1362.061	2.727	2.5				

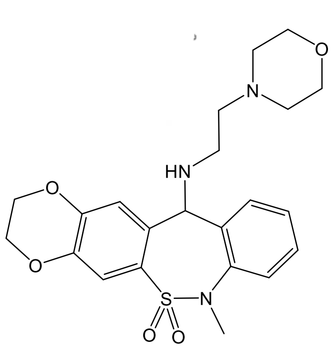

54c

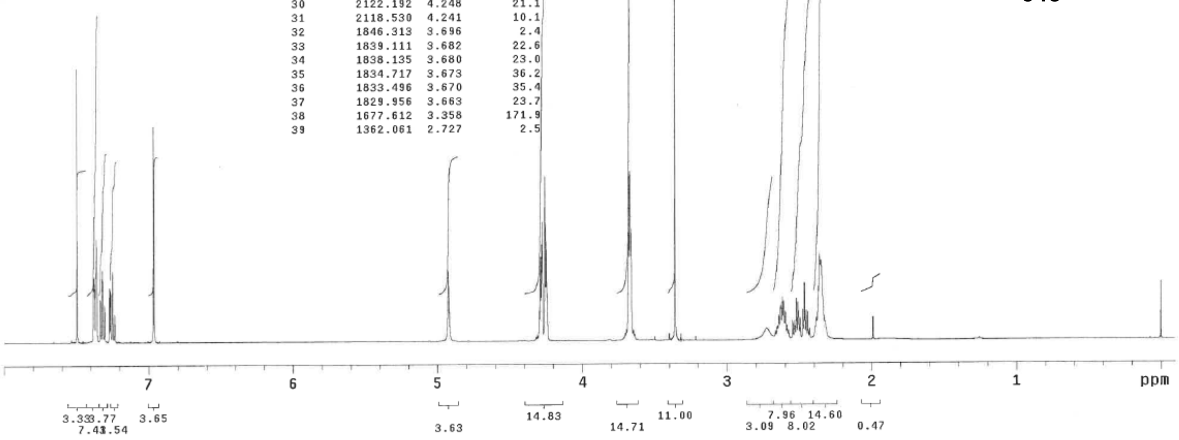

STANDARD 13C
62179
1939-BGE
Berecz Gabor
2006.02.24. (CzB)
Solvent: CDCl3
Temp. 25.0 C / 298.1 K
User: 1-14-87
INOVA-500 "i500"
PULSE SEQUENCE
Relax. delay 3.000 sec
Pulse 6.0 usec
Acq. time 1.300 sec
Width 32000.0 Hz
416 repetitions
OBSERVE C13, 125.6055347 MHz
DECOUPLE H1, 499.5269647 MHz
Power 32 dB
continuously on
WALTZ-16 modulated
DATA PROCESSING
Line broadening 0.5 Hz
FT size 131072
Total time 29 minutes

INDEX	FREQUENCY	PPM	HEIGHT
1	18366.707	146.214	53.6
2	17927.742	142.720	52.4
3	17523.933	139.505	11.4
4	17440.437	138.840	38.3
5	16660.652	132.632	16.6
6	16426.765	130.771	27.3
7	16246.101	129.332	14.0
8	16184.578	128.843	76.8
9	16034.676	127.649	79.2
10	16003.914	127.404	68.3
11	14829.598	118.056	83.7
12	14804.695	117.858	16.3
13	9704.598	77.257	131.7
14	9672.371	77.000	139.6
15	9640.633	76.747	132.3
16	8399.910	66.870	158.2
17	8213.875	65.389	8.0
18	8114.265	64.596	78.2
19	8059.090	64.157	77.3
20	7309.090	58.186	75.5
21	6736.336	53.627	107.8
22	5540.535	44.107	65.5
23	4767.098	37.950	36.4

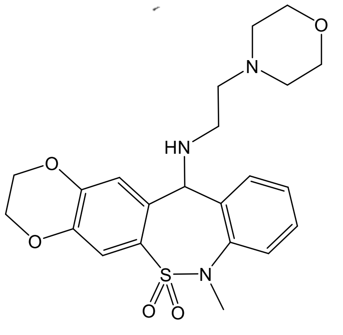

54c

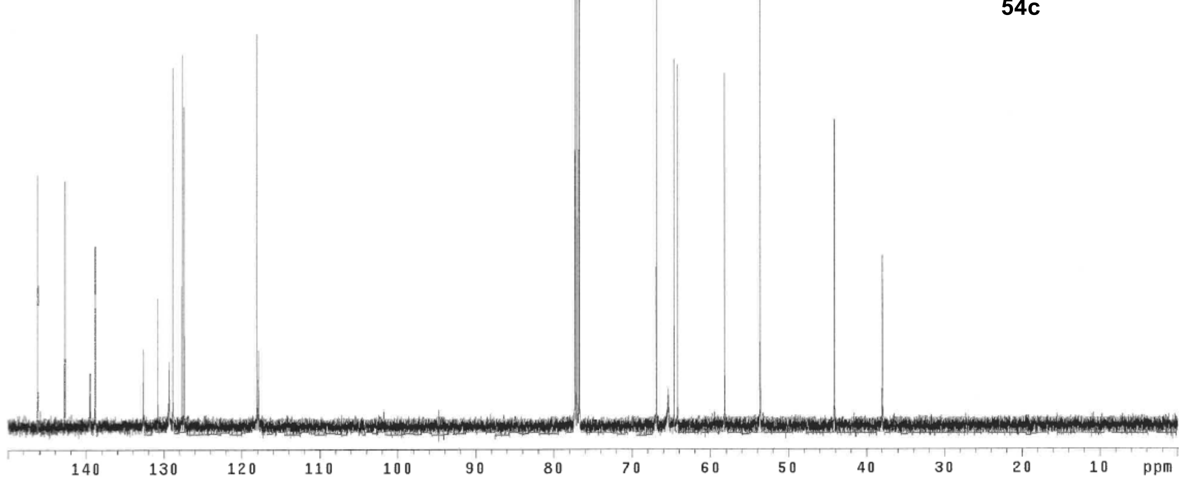

STANDARD 1H
62209
1940-BGE
Berecz Gabor
2006.02.24. (DA)
Solvent: CDCl3
Temp. 25.0 C / 298.1 K
File: 62209ih
INOVA-500 "i500"
PULSE SEQUENCE
Pulse 6.4 usec
Acq. time 5.000 sec
Width 8000.0 Hz
16 repetitions
OBSERVE H1, 499.5244654 MHz
DATA PROCESSING
FT size 131072
Total time 1 minute

INDEX	FREQUENCY	PPM	HEIGHT
1	3743.408	7.494	51.7
2	3685.303	7.378	10.6
3	3683.838	7.375	10.7
4	3677.734	7.362	13.8
5	3676.270	7.360	14.1
6	3674.194	7.355	9.3
7	3672.729	7.352	9.6
8	3666.260	7.339	19.3
9	3664.917	7.337	18.6
10	3657.227	7.321	9.3
11	3655.640	7.318	9.1
12	3650.024	7.307	15.0
13	3648.437	7.304	12.4
14	3642.212	7.291	7.9
15	3632.812	7.273	8.2
16	3628.174	7.263	11.6
17	3626.587	7.260	10.8
18	3620.728	7.248	14.0
19	3619.263	7.245	12.9
20	3494.629	6.996	42.2
21	2494.385	4.994	14.3
22	2140.991	4.286	9.8
23	2140.259	4.285	10.2
24	2137.329	4.279	18.4
25	2132.935	4.270	29.4
26	2122.681	4.249	27.0
27	2120.972	4.246	16.9
28	2119.263	4.243	14.5
29	2117.554	4.239	18.0
30	2115.845	4.236	9.4
31	2114.136	4.232	7.2
32	1839.478	3.682	28.5
33	1834.839	3.673	42.5
34	1830.200	3.664	27.1
35	1660.034	3.323	162.0
36	1289.551	2.582	14.2
37	1210.693	2.424	22.7
38	1206.787	2.416	33.8
39	1199.707	2.402	38.6
40	1192.627	2.388	20.1
41	991.943	1.986	7.9
42	854.736	1.711	9.4
43	848.022	1.698	13.0
44	841.309	1.684	8.5
45	-0.000	-0.000	10.4

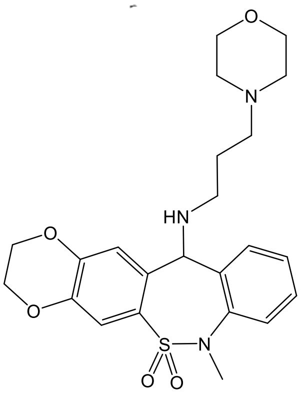

54d

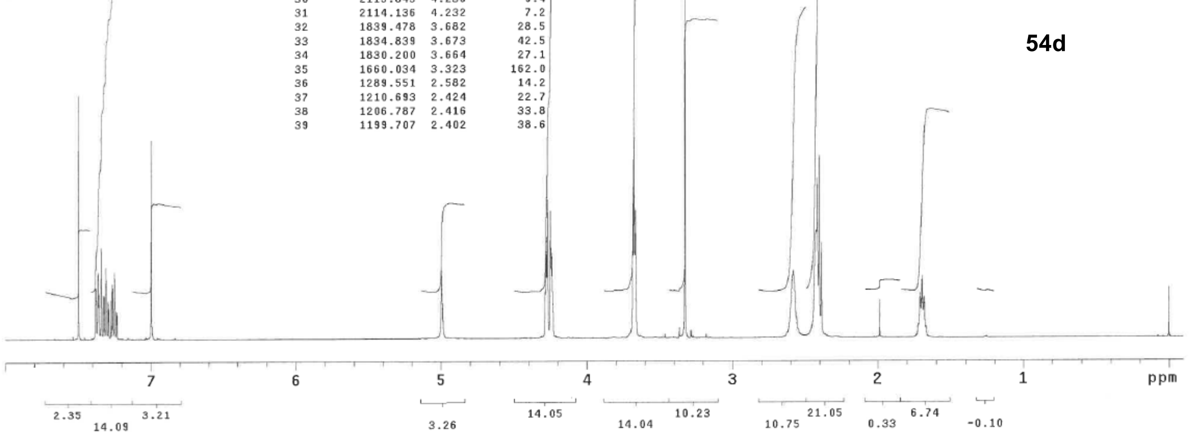

STANDARD 13C
62209
1940-BGE
Berecz Gabor
2006.02.24. (DA)
Solvent: CDCl3
Temp. 25.0 C / 298.1 K
Sample #3, user 1-14-87
INOVA-500 "i500"
PULSE SEQUENCE
Relax. delay 1.200 sec
Pulse 6.0 usec
Acq. time 1.300 sec
Width 32000.0 Hz
8192 repetitions
OBSERVE C13, 125.6055371 MHz
DECOUPLE H1, 499.5269647 MHz
Power 32 dB
continuously on
WALTZ-16 modulated
DATA PROCESSING
Line broadening 0.5 Hz
FT size 131072
Total time 5.7 hours

INDEX	FREQUENCY	PPM	HEIGHT
1	18379.891	146.319	41.1
2	17909.676	142.576	40.9
3	17529.305	139.548	6.4
4	17438.484	138.825	25.0
5	16696.785	132.920	10.2
6	16378.914	130.390	19.0
7	16213.387	129.072	9.0
8	16163.094	128.671	59.8
9	16019.539	127.529	66.9
10	15996.590	127.346	54.9
11	14838.387	118.126	67.2
12	14745.613	117.387	11.0
13	9704.598	77.257	49.2
14	9672.371	77.000	51.7
15	9640.633	76.747	49.6
16	8401.375	66.882	146.6
17	8112.801	64.585	68.4
18	8056.160	64.134	59.1
19	7190.438	57.242	70.5
20	6747.078	53.712	154.1
21	5854.500	46.607	50.0
22	4762.703	37.915	22.6
23	3324.715	26.467	59.3

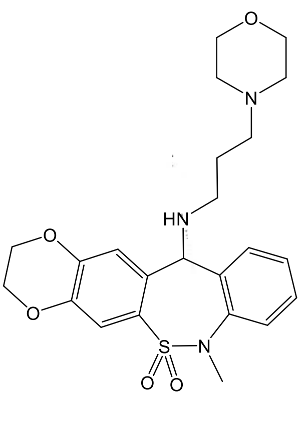

54d

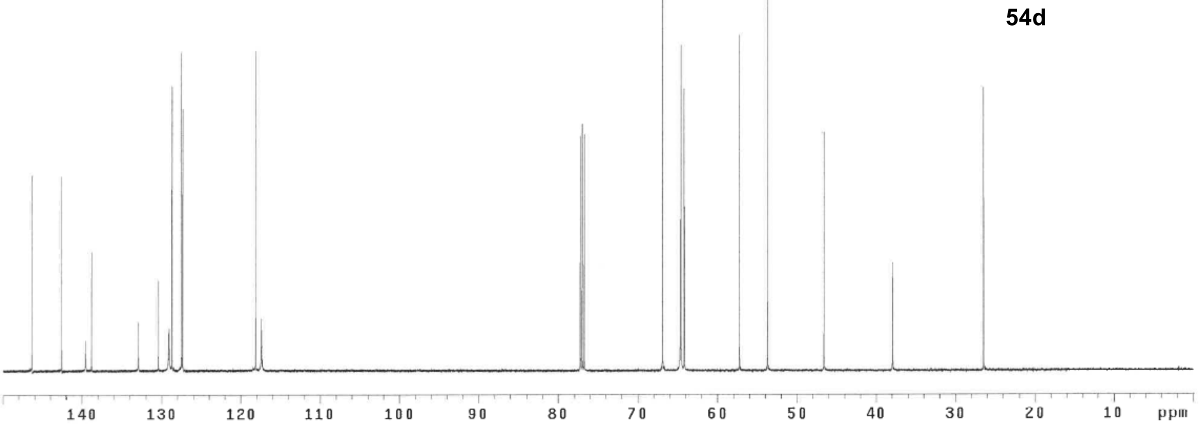

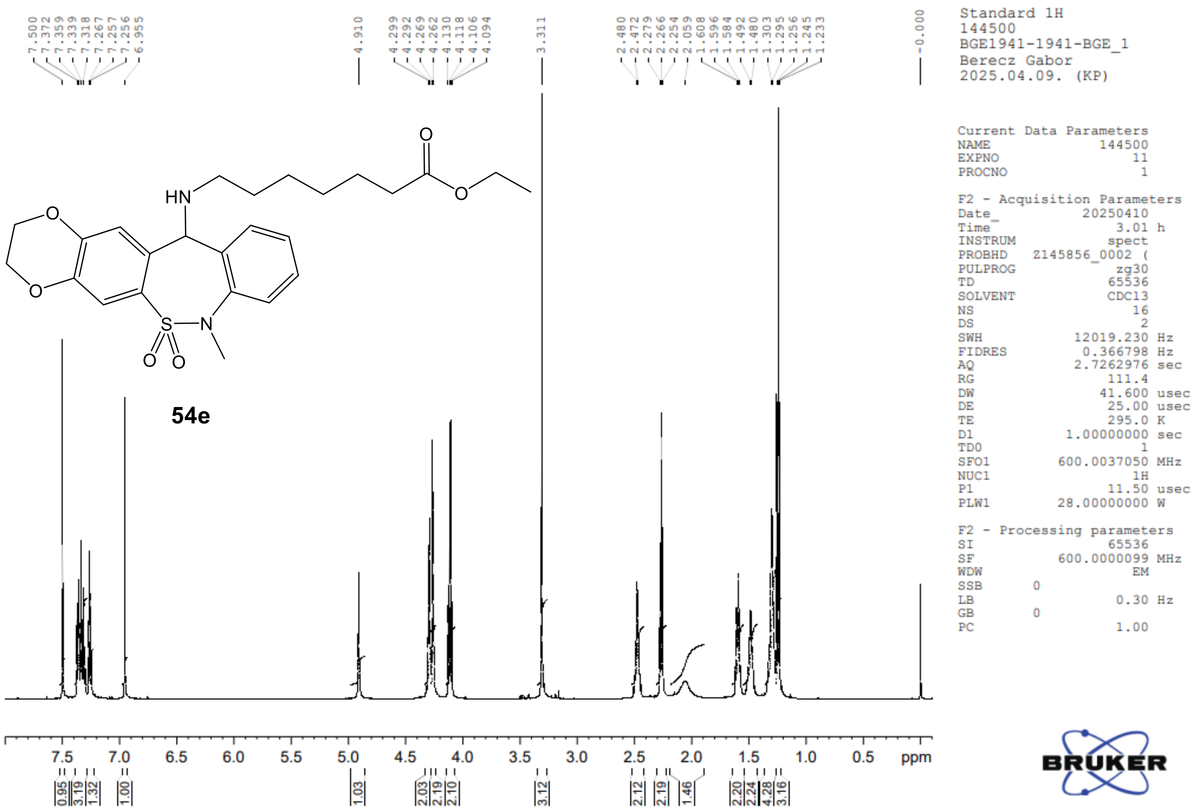

54e
Standard 1H
144500
BGE1941-1941-BGE_1
Berecz Gabor
2025.04.09. (KP)
Current Data Parameters
NAME 144500
EXPNO 11
PROCNO 1
F2 - Acquisition Parameters
Date_ 20250410
Time 3.01 h
INSTRUM spect
PROBHD Z145856_0002 (
PULPROG zg30
TD 65536
SOLVENT CDCl3
NS 16
DS 2
SWH 12019.230 Hz
FIDRES 0.366798 Hz
AQ 2.7262976 sec
RG 111.4
DW 41.600 usec
DE 25.00 usec
TE 295.0 K
D1 1.00000000 sec
TD0 1
SFO1 600.0037050 MHz
NUC1 1H
P1 11.50 usec
PLW1 28.00000000 W
F2 - Processing parameters
SI 65536
SF 600.0000099 MHz
WDW EM
SSB 0
LB 0.30 Hz
GB 0
PC 1.00
ppm

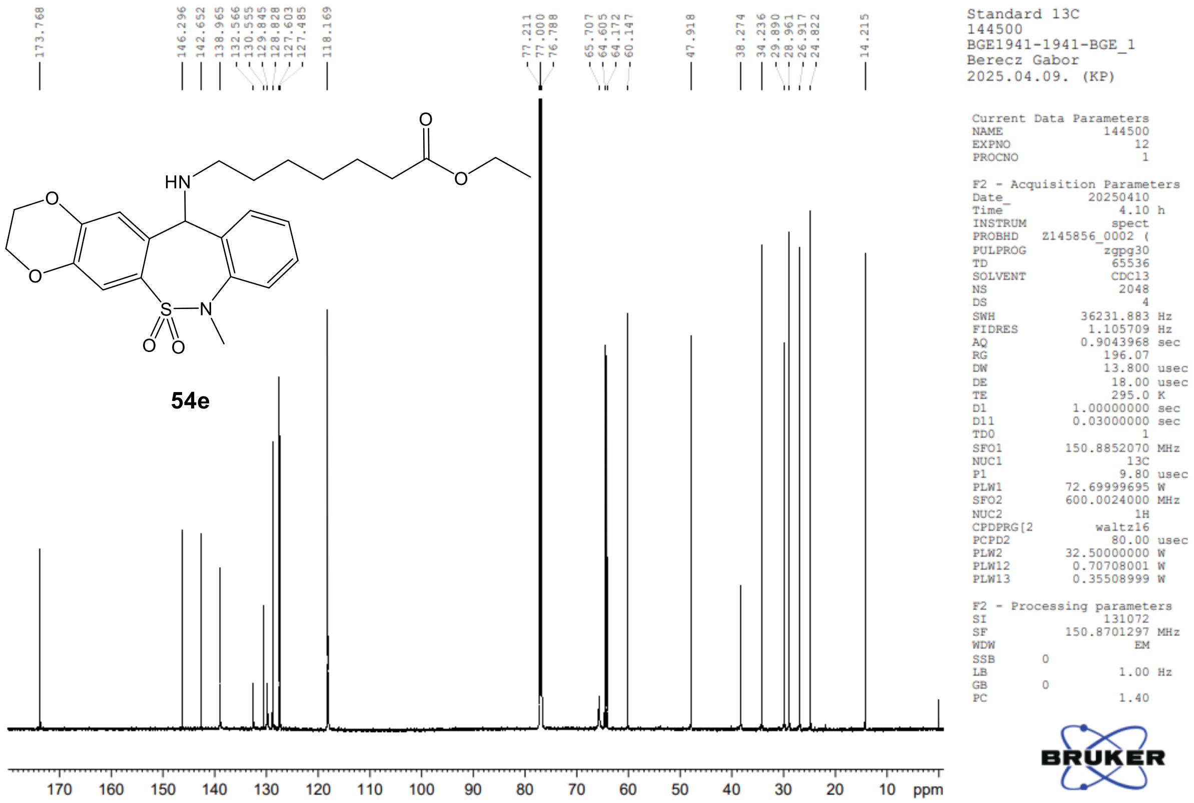

54e
Standard 13C
144500
BGE1941-1941-BGE_1
Berecz Gabor
2025.04.09. (KP)
Current Data Parameters
NAME 144500
EXPNO 12
PROCNO 1
F2 - Acquisition Parameters
Date_ 20250410
Time 4.10 h
INSTRUM spect
PROBHD Z145856_0002 (
PULPROG zgpg30
TD 65536
SOLVENT CDCl3
NS 2048
DS 4
SWH 36231.883 Hz
FIDRES 1.105709 Hz
AQ 0.9043968 sec
RG 196.07
DW 13.800 usec
DE 18.00 usec
TE 295.0 K
D1 1.00000000 sec
D11 0.03000000 sec
TD0 1
SFO1 150.8852070 MHz
NUC1 13C
P1 9.80 usec
PLW1 72.69999695 W
SFO2 600.0024000 MHz
NUC2 1H
CPDPRG[2 waltz16
PCPD2 80.00 usec
PLW2 32.50000000 W
PLW12 0.70708001 W
PLW13 0.35508999 W
F2 - Processing parameters
SI 131072
SF 150.8701297 MHz
WDW EM
SSB 0
LB 1.00 Hz
GB 0
PC 1.40
ppm

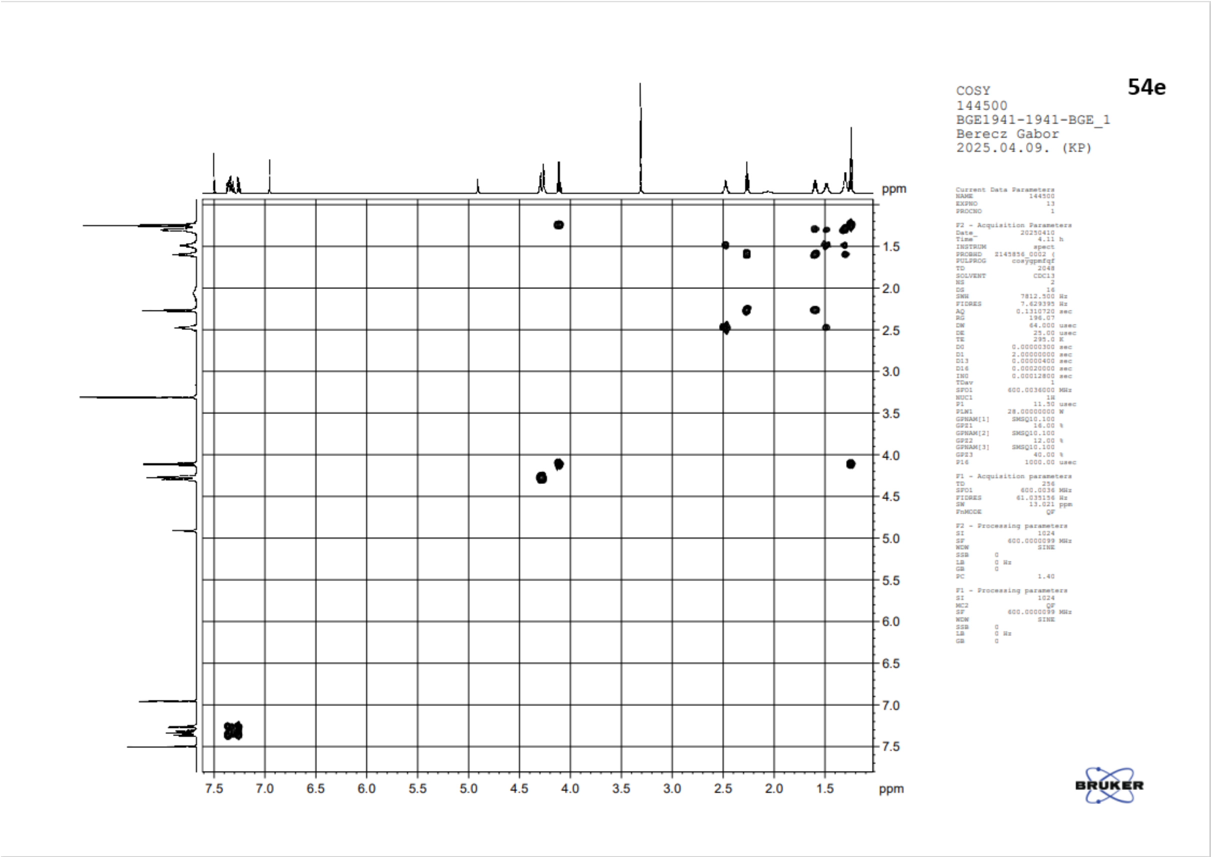
COSY
144500
BGE1941-1941-BGE_1
Berecz Gabor
2025.04.09. (KP)
54e
ppm
BRUKER

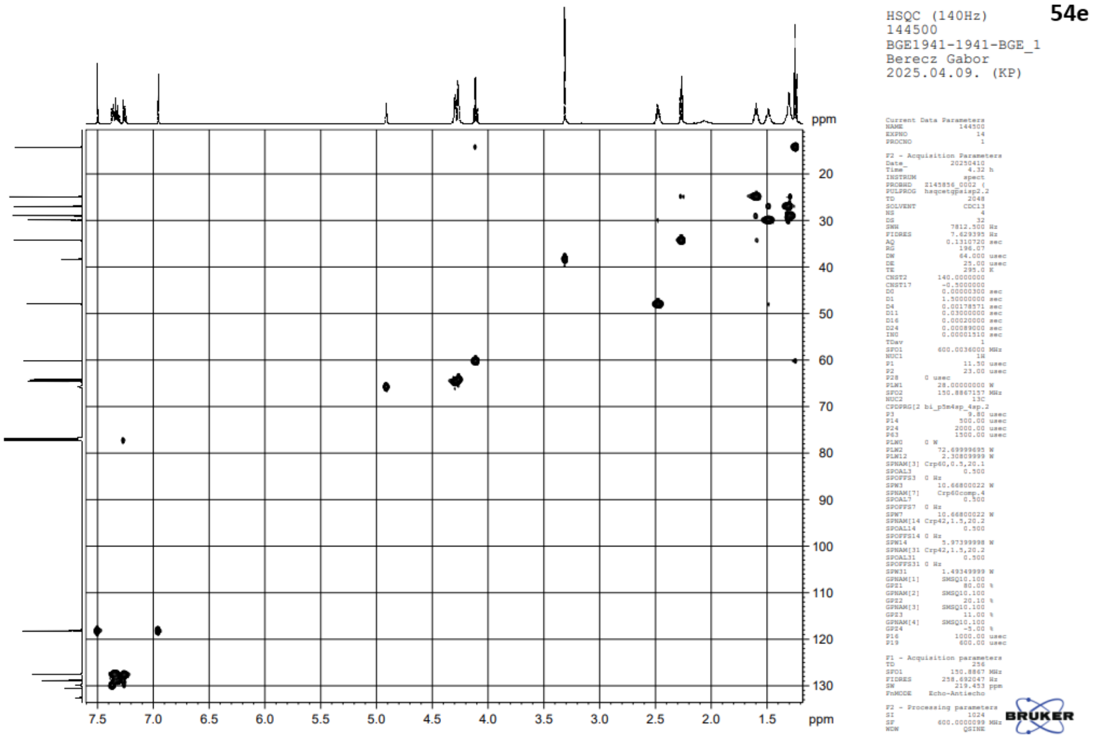
HSQC (140Hz)
144500
BGE1941-1941-BGE_1
Berecz Gabor
2025.04.09. (KP)
54e
ppm
BRUKER

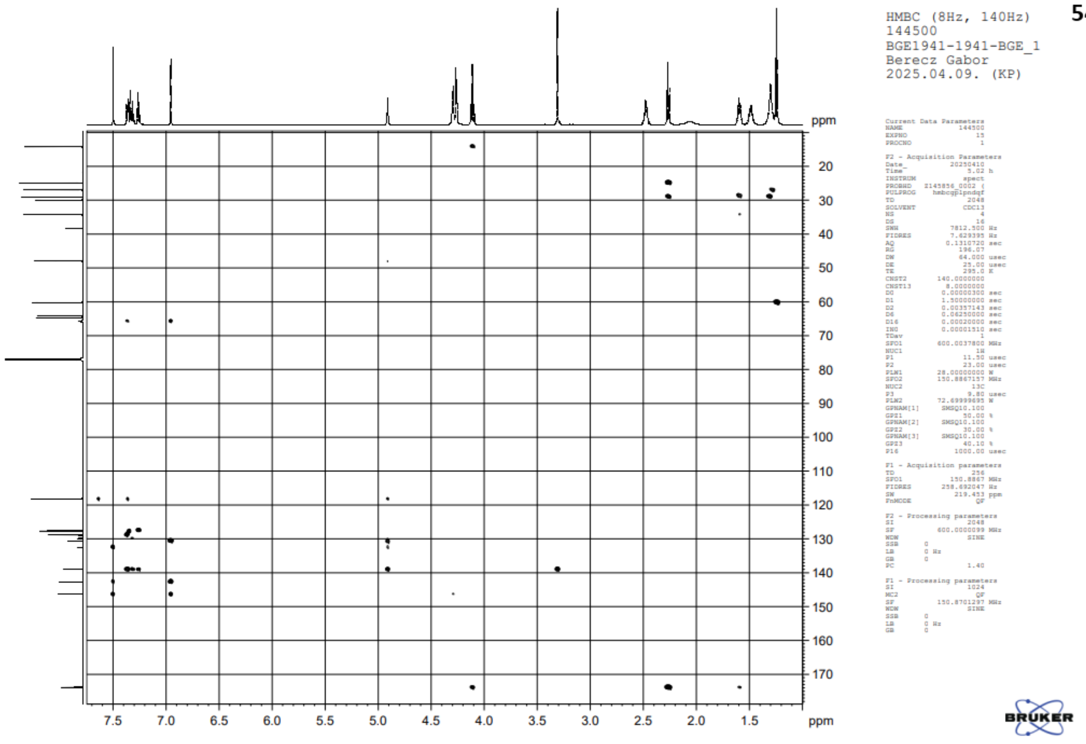
HMBC (8Hz, 140Hz)
144500
BGE1941-1941-BGE_1
Berecz Gabor
2025.04.09. (KP)
ppm
ppm

54e

STANDARD 1H
62547
1942-BGE
Berecz Gabor
2006.03.16. (CzB)
Solvent: CDCl3
Temp. 25.0 C / 298.1 K
INOVA-500 "i500"
PULSE SEQUENCE
Relax. delay 1.000 sec
Pulse 6.4 usec
Acq. time 5.000 sec
Width 8000.0 Hz
16 repetitions
OBSERVE H1, 499.5244638 MHz
DATA PROCESSING
FT size 131072
Total time 1 minutes

INDEX	FREQUENCY	PPM	HEIGHT	INDEX	FREQUENCY	PPM	HEIGHT
1	4047.607	8.103	10.5	40	762.817	1.527	16.5
2	3745.239	7.498	67.1	41	755.005	1.511	9.4
3	3734.985	7.477	21.8	42	744.507	1.490	9.6
4	3677.368	7.362	6.9	43	738.525	1.478	11.1
5	3676.270	7.360	6.4	44	731.812	1.465	9.9
6	3669.434	7.346	19.5	45	724.487	1.450	7.1
7	3662.354	7.332	19.0	46	626.343	1.254	41.5
8	3652.344	7.312	29.2	47	0.000	0.000	7.7
9	3651.489	7.310	29.1				
10	3644.531	7.296	14.8				
11	3633.911	7.275	6.6				
12	3625.244	7.257	13.7				
13	3617.676	7.242	21.0				
14	3610.474	7.228	9.8				
15	3531.128	7.069	57.9				
16	2610.107	5.225	40.6				
17	2154.053	4.312	5.7				
18	2151.001	4.306	7.5				
19	2148.193	4.300	8.7				
20	2142.578	4.289	21.1				
21	2138.550	4.281	25.9				
22	2135.864	4.276	35.0				
23	2130.127	4.264	48.4				
24	1605.713	3.214	160.8				
25	1276.733	2.556	7.7				
26	1274.536	2.551	8.2				
27	1270.752	2.544	8.2				
28	1268.677	2.540	8.4				
29	1265.991	2.534	8.2				
30	1260.132	2.523	5.8				
31	1217.041	2.436	5.5				
32	1210.327	2.423	9.1				
33	1201.904	2.406	8.3				
34	1199.463	2.401	7.9				
35	1108.521	2.219	20.6				
36	1101.196	2.204	42.1				
37	1093.872	2.190	22.2				
38	776.367	1.554	13.7				
39	769.653	1.541	20.1				

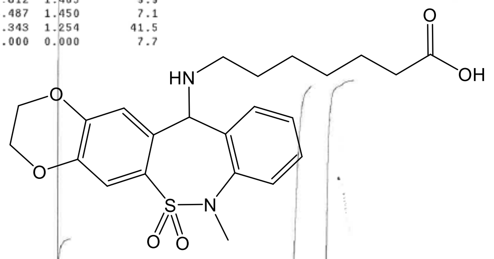

54f

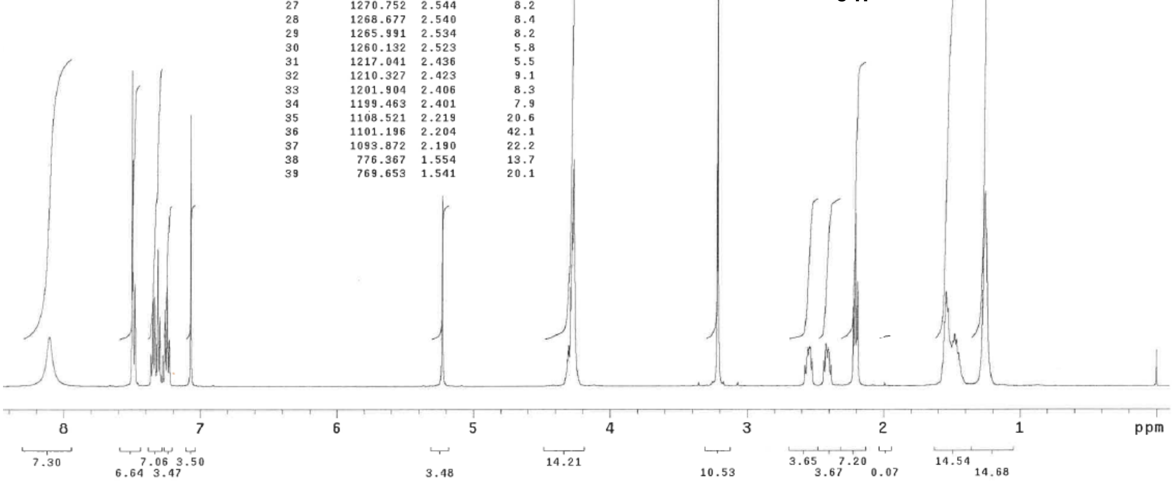

STANDARD 13C
62547
1942-BGE
Berecz Gabor
2006.03.16. (CzB)
Solvent: CDCl3
Temp. 25.0 C / 298.1 K
User: 1-14-87
INOVA-500 "i500"
PULSE SEQUENCE
Relax. delay 3.000 sec
Pulse 6.0 usec
Acq. time 1.300 sec
Width 32000.0 Hz
688 repetitions
OBSERVE C13, 125.6055371 MHz
DECOUPLE H1, 499.5269647 MHz
Power 32 dB
continuously on
WALTZ-16 modulated
DATA PROCESSING
Line broadening 0.5 Hz
FT size 131072
Total time 49 minutes

INDEX	FREQUENCY	PPM	HEIGHT
1	22379.891	178.162	61.0
2	18417.000	146.614	79.6
3	17986.824	143.190	76.8
4	17558.602	139.781	50.3
5	16852.547	134.160	7.7
6	16542.000	131.688	13.7
7	16379.402	130.393	49.1
8	16284.676	129.639	54.0
9	16181.160	128.815	11.3
10	16002.938	127.396	62.7
11	15970.711	127.140	64.1
12	15117.684	120.349	15.9
13	14838.875	118.130	70.5
14	9704.109	77.253	151.4
15	9672.371	77.000	162.0
16	9640.145	76.743	154.1
17	8237.313	65.576	15.2
18	8103.523	64.511	60.5
19	8064.949	64.204	58.3
20	5881.356	46.820	52.2
21	4877.938	38.832	47.7
22	4416.512	35.159	56.2
23	3634.285	28.932	93.8
24	3590.340	28.582	63.5
25	3364.754	26.786	86.8
26	3155.770	25.123	85.9

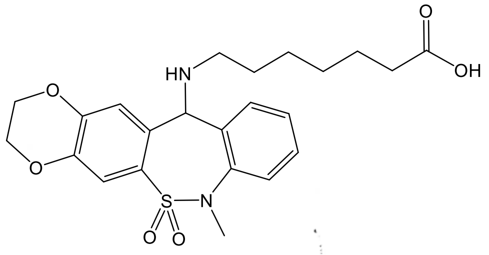

54f

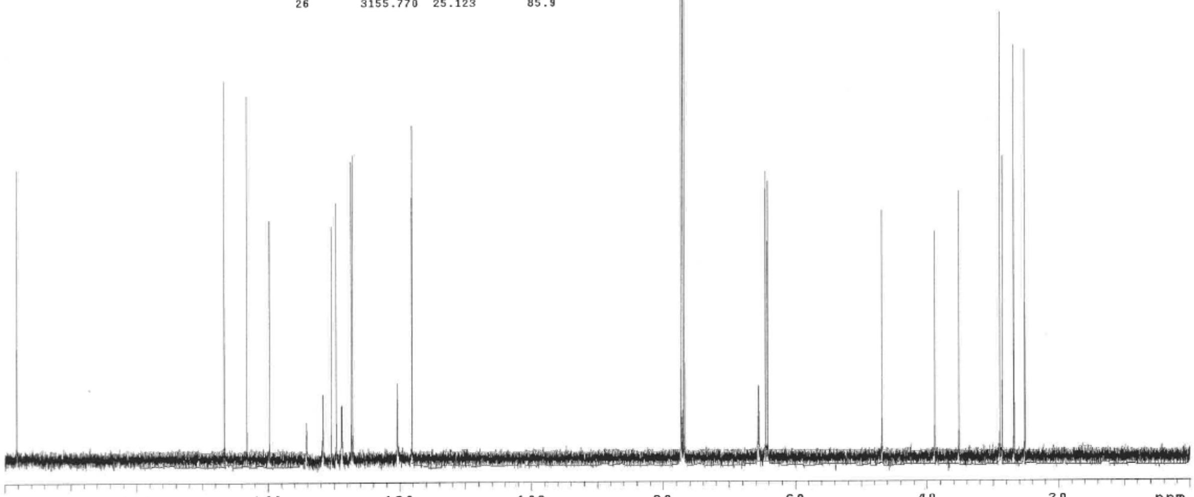

Supplement: File 1 — Experimental procedures and characterization of compounds 6, 10–19, 20a–e, 21c–p, 23a–e, 24, 26–28, 30–37, 38a–d, 39–44, 45a–c, 46–53, 54a–f. 1H and 13C NMR spectra of all new compounds. 2D NMR spectra of compounds 6–8, 12, 21a, 21b, 25–27, 30, 32, 38b, 42, 50, 51, 54e. [file Beilstein_J_Org_Chem-21-2645-s001.pdf]
