# Supplementary material for: Synthesis of new tetra- and pentacyclic, methylenedioxy- and ethylenedioxy-substituted derivatives of the dibenzo[c,f][1,2]thiazepine ring system
Source: Beilstein J Org Chem. 2025 Dec 9;21:2645–56. doi: 10.3762/bjoc.21.205 (PMC12706374; doi:10.3762/bjoc.21.205)

checkCIF/PLATON report

You have not supplied any structure factors. As a result the full set of tests cannot be run.

THIS REPORT IS FOR GUIDANCE ONLY. IF USED AS PART OF A REVIEW PROCEDURE FOR PUBLICATION, IT SHOULD NOT REPLACE THE EXPERTISE OF AN EXPERIENCED CRYSTALLOGRAPHIC REFEREE.

No syntax errors found.   CIF dictionary   Interpreting this report

Datablock: _144830_1

```
Bond precision:   C-C = 0.0035 A          Wavelength=1.54187

Cell:             a=6.6806(5)     b=21.7251(14)      c=12.1261(8)
                  alpha=90        beta=92.707(4)     gamma=90
Temperature:      293 K
```

	Calculated	Reported
Volume	1758.0(2)	1758.0(2)
Space group	P 21/a	P 1 21/a 1
Hall group	-P 2yab	-P 2yab
Moiety formula	2(C17 H14 Cl N O5 S), C4 H6 N	C19 H17 Cl N2 O5 S
Sum formula	C38 H34 Cl2 N3 O10 S2	C19 H17 Cl N2 O5 S
Mr	827.70	420.87
Dx,g cm-3	1.564	1.590
Z	2	4
Mu (mm-1)	3.347	3.368
F000	858.0	872.0
F000'	863.25	
h,k,lmax	8,26,14	8,26,14
Nref	3441	3324
Tmin,Tmax	0.730,0.845	0.549,0.845
Tmin'	0.359	

```
Correction method= # Reported T Limits: Tmin=0.549 Tmax=0.845
AbsCorr = MULTI-SCAN

Data completeness= 0.966          Theta(max)= 71.700

R(reflections)= 0.0726( 13258)               wR2(reflections)=
                                             0.1078( 13358)
S = 1.036                 Npar= 279
```

The following ALERTS were generated. Each ALERT has the format
test-name_ALERT_alert-type_alert-level.
Click on the hyperlinks for more details of the test.

Alert level A

```
DIFMN02_ALERT_2_A  The minimum difference density is < -0.1*ZMAX*2.00
            _refine_diff_density_min given =     -7.570
            Test value =     -3.400
```

Author Response: Altough the residual density is out of border but there is no doubt about the chemical structure taking into account other evidences, e.g. NMR. The error maybe due to crystal imperfections.

```
PLAT097_ALERT_2_A Large Reported Max.  (Positive) Residual Density       6.13 eA-3
```

Author Response: Altough the residual density is out of border but there is no doubt about the chemical structure taking into account other evidences, e.g. NMR. The error maybe due to crystal imperfections.

```
PLAT098_ALERT_2_A Large Reported Min.  (Negative) Residual Density      -7.57 eA-3
```

Author Response: Altough the residual density is out of border but there is no doubt about the chemical structure taking into account other evidences, e.g. NMR. The error maybe due to crystal imperfections.

```
PLAT703_ALERT_1_A Torsion Calc -169.84(16), Rep   -169.2(2), Dev..       4.00 Sigma
              O(6)-S(1)-N(1)-C(21  1_555  1_555  1_555  1_555      #     6 Check
```

Author Response: The structure is affected by some strain. Crystal imperfections can not be excluded either.

```
PLAT703_ALERT_1_A Torsion Calc    -63.8(2), Rep    -64.9(3), Dev..       5.50 Sigma
              S(1)-N(1)-C(21-C(19  1_555  1_555  1_555  1_555      #    24 Check
```

Author Response: The structure is affected by some strain. Crystal imperfections can not be excluded either.

Alert level B

```
REFLT02_ALERT_1_B  The number of reflections greater than the sigma threshold
            cannot exceed the number of symmetry-independent reflections
            Number of symmetry-independent reflections =   3324
            Number of reflections greater than sigma threshold =  13258
PLAT043_ALERT_1_B Calculated and Reported Mol. Weight Differ by ..      14.04 Check
PLAT230_ALERT_2_B Hirshfeld Test Diff for    S1       --O6       .        7.8 s.u.
```

```
PLAT230_ALERT_2_B Hirshfeld Test Diff for    S1       --N1       .        7.5 s.u.
PLAT230_ALERT_2_B Hirshfeld Test Diff for    N1       --C21      .        9.5 s.u.
PLAT241_ALERT_2_B High   'MainMol' Ueq as Compared to Neighbors of        C26 Check
PLAT364_ALERT_2_B Short  C(sp3)-C(sp)  Bond  C2       - C28      .       1.22 Ang.
PLAT413_ALERT_2_B Short Inter XH3 .. XHn     H8       ..H17      .       1.97 Ang.
                                                -1+x,y,z  =      1_455 Check
PLAT703_ALERT_1_B Torsion Calc  -40.54(19), Rep    -40.0(2), Dev..       2.84 Sigma
              O(5)-S(1)-N(1)-C(21  1_555  1_555  1_555  1_555      #    2 Check
```

Author Response: The structure is affected by some strain. Crystal imperfections can not be excluded either.

```
PLAT703_ALERT_1_B Torsion Calc   74.99(18), Rep     75.5(2), Dev..       2.83 Sigma
              C(15-S(1)-N(1)-C(21  1_555  1_555  1_555  1_555      #   12 Check
```

Author Response: The structure is affected by some strain. Crystal imperfections can not be excluded either.

```
PLAT703_ALERT_1_B Torsion Calc   -172.4(2), Rep   -173.0(3), Dev..       3.00 Sigma
              C(26-O(7)-C(14-C(16  1_555  1_555  1_555  1_555      #   21 Check
```

Author Response: The structure is affected by some strain. Crystal imperfections can not be excluded either.

```
PLAT703_ALERT_1_B Torsion Calc     79.0(2), Rep     78.5(4), Dev..       2.50 Sigma
              C(11-N(1)-C(21-C(19  1_555  1_555  1_555  1_555      #   25 Check
```

Author Response: The structure is affected by some strain. Crystal imperfections can not be excluded either.

```
PLAT703_ALERT_1_B Torsion Calc    118.8(2), Rep    118.2(3), Dev..       3.00 Sigma
              C(21-N(1)-C(11-C(16  1_555  1_555  1_555  1_555      #   27 Check
```

Author Response: The structure is affected by some strain. Crystal imperfections can not be excluded either.

```
PLAT703_ALERT_1_B Torsion Calc   -176.9(2), Rep   -177.5(3), Dev..       3.00 Sigma
              C(13-C(1)-C(12-C(18  1_555  1_555  1_555  1_555      #   31 Check
```

Author Response: The structure is affected by some strain. Crystal imperfections can not be excluded either.

```
PLAT703_ALERT_1_B Torsion Calc    176.0(2), Rep    176.5(3), Dev..       2.50 Sigma
              C(16-C(11-C(12-C(18  1_555  1_555  1_555  1_555      #   47 Check
```

Author Response: The structure is affected by some strain. Crystal imperfections can not be excluded either.

```
PLAT703_ALERT_1_B Torsion Calc  178.64(16), Rep    179.0(2), Dev..       2.25 Sigma
              C(15-C(17-C(23-CL(2  1_555  1_555  1_555  1_555      #   60 Check
```

Author Response: The structure is affected by some strain. Crystal imperfections can not be excluded either.

```
PLAT703_ALERT_1_B Torsion Calc    -59.9(3), Rep    -59.0(4), Dev..       3.00 Sigma
              O(3)-C(19-C(21-N(1)  1_555  1_555  1_555  1_555      #   62 Check
```

Author Response: The structure is affected by some strain. Crystal imperfections can not be excluded either.

```
PLAT703_ALERT_1_B Torsion Calc -179.45(16), Rep -179.93(19), Dev..       3.00 Sigma
              C(20-C(22-C(23-CL(2  1_555  1_555  1_555  1_555      #   64 Check
```

Author Response: The structure is affected by some strain. Crystal imperfections can not be excluded either.

Alert level C

```
DIFMN03_ALERT_1_C  The minimum difference density is < -0.1*ZMAX*0.75
            The relevant atom site should be identified.
DIFMX02_ALERT_1_C  The maximum difference density is > 0.1*ZMAX*0.75
            The relevant atom site should be identified.
PLAT029_ALERT_3_C _diffrn_measured_fraction_theta_full value Low .      0.966 Why?
PLAT041_ALERT_1_C Calc. and Reported SumFormula    Strings  Differ     Please Check
              Calc: C38 H34 Cl2 N3 O10 S2
              Rep.: C19 H17 Cl N2 O5 S
PLAT042_ALERT_1_C Calc. and Reported MoietyFormula Strings  Differ     Please Check
              Calc: 2(C17 H14 Cl N O5 S), C4 H6 N
              Rep.: C19 H17 Cl N2 O5 S
PLAT068_ALERT_1_C Reported F000 Differs from Calcd (or Missing)...     Please Check
PLAT166_ALERT_4_C S.U's Given on Coordinates for Calc-flagged ....         H1 Note
PLAT166_ALERT_4_C S.U's Given on Coordinates for Calc-flagged ....         H2 Note
PLAT166_ALERT_4_C S.U's Given on Coordinates for Calc-flagged ....         H3 Note
PLAT166_ALERT_4_C S.U's Given on Coordinates for Calc-flagged ....         H4 Note
PLAT166_ALERT_4_C S.U's Given on Coordinates for Calc-flagged ....         H5 Note
PLAT166_ALERT_4_C S.U's Given on Coordinates for Calc-flagged ....         H6 Note
PLAT166_ALERT_4_C S.U's Given on Coordinates for Calc-flagged ....         H7 Note
PLAT166_ALERT_4_C S.U's Given on Coordinates for Calc-flagged ....         H8 Note
PLAT166_ALERT_4_C S.U's Given on Coordinates for Calc-flagged ....         H9 Note
PLAT166_ALERT_4_C S.U's Given on Coordinates for Calc-flagged ....        H10 Note
PLAT202_ALERT_3_C Isotropic non-H Atoms in Anion/Solvent ........          3 Check
              N2      C2      C28
PLAT213_ALERT_2_C Atom C26             has ADP max/min Ratio .....        3.2 prolat
PLAT220_ALERT_2_C NonSolvent   Resd 1  C   Ueq(max)/Ueq(min) Range        4.6 Ratio
PLAT222_ALERT_3_C NonSolvent Resd 1  H   Uiso(max)/Uiso(min) Range       10.0 Ratio
```

```
PLAT230_ALERT_2_C Hirshfeld Test Diff for    C12      --C23      .        5.3 s.u.
PLAT230_ALERT_2_C Hirshfeld Test Diff for    C9       --C20      .        6.5 s.u.
PLAT242_ALERT_2_C Low    'MainMol' Ueq as Compared to Neighbors of         O4 Check
PLAT242_ALERT_2_C Low    'MainMol' Ueq as Compared to Neighbors of         O7 Check
PLAT243_ALERT_4_C High   'Solvent' Ueq as Compared to Neighbors of        C28 Check
PLAT245_ALERT_2_C U(iso) H3        Smaller than U(eq) C17      by      0.026 Ang**2
PLAT245_ALERT_2_C U(iso) H4        Smaller than U(eq) C18      by      0.026 Ang**2
PLAT245_ALERT_2_C U(iso) H5        Smaller than U(eq) C1       by      0.015 Ang**2
PLAT245_ALERT_2_C U(iso) H7        Smaller than U(eq) C19      by      0.022 Ang**2
PLAT245_ALERT_2_C U(iso) H10       Smaller than U(eq) C21      by      0.011 Ang**2
PLAT360_ALERT_2_C Short  C(sp3)-C(sp3) Bond  C25      - C26      .       1.34 Ang.
PLAT413_ALERT_2_C Short Inter XH3 .. XHn     H13      ..H16      .       2.06 Ang.
                                                x,y,1+z  =      1_556 Check
PLAT703_ALERT_1_C Torsion Calc  175.82(15), Rep    175.6(2), Dev..       1.47 Sigma
              O(5)-S(1)-N(1)-C(11  1_555  1_555  1_555  1_555      #    1 Check
```

Author Response: The structure is affected by some strain. Crystal imperfections can not be excluded either.

```
PLAT703_ALERT_1_C Torsion Calc -104.95(19), Rep   -105.3(2), Dev..       1.84 Sigma
              O(6)-S(1)-C(15-C(9)  1_555  1_555  1_555  1_555      #    7 Check
```

Author Response: The structure is affected by some strain. Crystal imperfections can not be excluded either.

```
PLAT703_ALERT_1_C Torsion Calc   75.09(17), Rep     74.9(2), Dev..       1.12 Sigma
              O(6)-S(1)-C(15-C(17  1_555  1_555  1_555  1_555      #    8 Check
```

Author Response: The structure is affected by some strain. Crystal imperfections can not be excluded either.

```
PLAT703_ALERT_1_C Torsion Calc  -68.65(17), Rep    -68.9(2), Dev..       1.47 Sigma
              C(15-S(1)-N(1)-C(11  1_555  1_555  1_555  1_555      #   11 Check
```

Author Response: The structure is affected by some strain. Crystal imperfections can not be excluded either.

```
PLAT703_ALERT_1_C Torsion Calc     64.9(3), Rep     64.5(4), Dev..       1.33 Sigma
              C(18-O(3)-C(19-C(21  1_555  1_555  1_555  1_555      #   13 Check
```

Author Response: The structure is affected by some strain. Crystal imperfections can not be excluded either.

```
PLAT703_ALERT_1_C Torsion Calc   -169.0(2), Rep   -168.6(3), Dev..       2.00 Sigma
              C(25-O(4)-C(13-C(1)  1_555  1_555  1_555  1_555      #   17 Check
```

Author Response: The structure is affected by some strain. Crystal imperfections can not be excluded either.

```
PLAT703_ALERT_1_C Torsion Calc       9.9(3), Rep     10.3(4), Dev..       1.33 Sigma
              C(25-O(4)-C(13-C(14  1_555  1_555  1_555  1_555      #   18 Check
```

Author Response: The structure is affected by some strain. Crystal imperfections can not be excluded either.

```
PLAT703_ALERT_1_C Torsion Calc      81.1(2), Rep     81.4(3), Dev..       1.50 Sigma
              S(1)-N(1)-C(11-C(12  1_555  1_555  1_555  1_555      #   22 Check
```

Author Response: The structure is affected by some strain. Crystal imperfections can not be excluded either.

```
PLAT703_ALERT_1_C Torsion Calc       2.6(3), Rep      2.0(5), Dev..       2.00 Sigma
              C(13-C(1)-C(12-C(11  1_555  1_555  1_555  1_555      #   30 Check
```

Author Response: The structure is affected by some strain. Crystal imperfections can not be excluded either.

```
PLAT703_ALERT_1_C Torsion Calc      48.6(3), Rep     49.0(4), Dev..       1.33 Sigma
              C(15-C(9)-C(18-C(12  1_555  1_555  1_555  1_555      #   33 Check
```

Author Response: The structure is affected by some strain. Crystal imperfections can not be excluded either.

```
PLAT703_ALERT_1_C Torsion Calc    -134.3(2), Rep   -133.9(3), Dev..       2.00 Sigma
              C(20-C(9)-C(18-C(12  1_555  1_555  1_555  1_555      #   41 Check
```

Author Response: The structure is affected by some strain. Crystal imperfections can not be excluded either.

```
PLAT703_ALERT_1_C Torsion Calc     179.3(2), Rep    179.6(3), Dev..       1.50 Sigma
              N(1)-C(11-C(16-C(14  1_555  1_555  1_555  1_555      #   44 Check
```

Author Response: The structure is affected by some strain. Crystal imperfections can not be excluded either.

```
PLAT703_ALERT_1_C Torsion Calc      -3.5(3), Rep     -3.0(5), Dev..       1.67 Sigma
              C(16-C(11-C(12-C(1)  1_555  1_555  1_555  1_555      #   46 Check
```

Author Response: The structure is affected by some strain. Crystal imperfections can not be excluded either.

```
PLAT703_ALERT_1_C Torsion Calc     114.6(2), Rep    114.2(3), Dev..       2.00 Sigma
              C(1)-C(12-C(18-C(9)  1_555  1_555  1_555  1_555      #   49 Check
```

Author Response: The structure is affected by some strain. Crystal imperfections can not be excluded either.

```
PLAT703_ALERT_1_C Torsion Calc    -64.9(3), Rep    -65.3(4), Dev..       1.33 Sigma
              C(11-C(12-C(18-C(9)  1_555  1_555  1_555  1_555      #   51 Check
```

Author Response: The structure is affected by some strain. Crystal imperfections can not be excluded either.

```
PLAT703_ALERT_1_C Torsion Calc    176.4(2), Rep    176.0(3), Dev..       2.00 Sigma
              C(1)-C(13-C(14-O(7)  1_555  1_555  1_555  1_555      #   54 Check
```

Author Response: The structure is affected by some strain. Crystal imperfections can not be excluded either.

```
PLAT703_ALERT_1_C Torsion Calc   -177.4(2), Rep   -177.0(3), Dev..       2.00 Sigma
              O(7)-C(14-C(16-C(11  1_555  1_555  1_555  1_555      #   56 Check
```

Author Response: The structure is affected by some strain. Crystal imperfections can not be excluded either.

Alert level G

```
FORMU01_ALERT_2_G  There is a discrepancy between the atom counts in the
            _chemical_formula_sum and the formula from the _atom_site* data.
            Atom count from _chemical_formula_sum:C19 H17 Cl1 N2 O5 S1
            Atom count from the _atom_site data:  C19 H17 Cl1 N1.5 O5 S1
CELLZ01_ALERT_1_G Difference between formula and atom_site contents detected.
CELLZ01_ALERT_1_G ALERT: Large difference may be due to a
            symmetry error - see SYMMG tests
           From the CIF: _cell_formula_units_Z    4
           From the CIF: _chemical_formula_sum  C19 H17 Cl N2 O5 S
           TEST: Compare cell contents of formula and atom_site data

           atom    Z*formula  cif sites diff
           C         76.00     76.00    0.00
           H         68.00     68.00    0.00
           Cl         4.00      4.00    0.00
           N          8.00      6.00    2.00
           O         20.00     20.00    0.00
           S          4.00      4.00    0.00
PLAT005_ALERT_5_G No Embedded Refinement Details Found  in the CIF     Please Do !
PLAT045_ALERT_1_G Calculated and Reported Z Differ by a Factor ...      0.500 Check
PLAT128_ALERT_4_G Alternate Setting for Input Space Group  P21/a        P21/c Note
PLAT199_ALERT_1_G Reported _cell_measurement_temperature ..... (K)        293 Check
PLAT200_ALERT_1_G Reported   _diffrn_ambient_temperature ..... (K)        293 Check
PLAT793_ALERT_4_G Model has Chirality at C18         (Centro SpGr)          S Verify
PLAT808_ALERT_5_G No Parseable SHELXL Style Weighting Scheme Found     Please Check
PLAT882_ALERT_1_G No Datum for _diffrn_reflns_av_unetI/netI ......     Please Do !
PLAT883_ALERT_1_G Absent Datum for _atom_sites_solution_primary ..     Please Do !
```

```
  5 ALERT level A = Most likely a serious problem - resolve or explain
 18 ALERT level B = A potentially serious problem, consider carefully
 49 ALERT level C = Check. Ensure it is not caused by an omission or oversight
 12 ALERT level G = General information/check it is not something unexpected

 43 ALERT type 1 CIF construction/syntax error, inconsistent or missing data
 23 ALERT type 2 Indicator that the structure model may be wrong or deficient
  3 ALERT type 3 Indicator that the structure quality may be low
 13 ALERT type 4 Improvement, methodology, query or suggestion
  2 ALERT type 5 Informative message, check
```

It is advisable to attempt to resolve as many as possible of the alerts in all categories. Often the minor alerts point to easily fixed oversights, errors and omissions in your CIF or refinement strategy, so attention to these fine details can be worthwhile. In order to resolve some of the more serious problems it may be necessary to carry out additional measurements or structure refinements. However, the purpose of your study may justify the reported deviations and the more serious of these should normally be commented upon in the discussion or experimental section of a paper or in the "special_details" fields of the CIF. checkCIF was carefully designed to identify outliers and unusual parameters, but every test has its limitations and alerts that are not important in a particular case may appear. Conversely, the absence of alerts does not guarantee there are no aspects of the results needing attention. It is up to the individual to critically assess their own results and, if necessary, seek expert advice.

Publication of your CIF in IUCr journals

A basic structural check has been run on your CIF. These basic checks will be run on all CIFs submitted for publication in IUCr journals (*Acta Crystallographica*, *Journal of Applied Crystallography*, *Journal of Synchrotron Radiation*); however, if you intend to submit to *Acta Crystallographica Section C* or *E* or *IUCrData*, you should make sure that full publication checks are run on the final version of your CIF prior to submission.

Publication of your CIF in other journals

Please refer to the *Notes for Authors* of the relevant journal for any special instructions relating to CIF submission.

Validation response form

Please find below a validation response form (VRF) that can be filled in and pasted into your CIF.

```
# start Validation Reply Form
_vrf_REFLT02__144830_1
;
PROBLEM: The number of reflections greater than the sigma threshold
RESPONSE: ...
;
_vrf_DIFMN03__144830_1
;
PROBLEM: The minimum difference density is < -0.1*ZMAX*0.75
RESPONSE: ...
```

```
;
_vrf_DIFMX02__144830_1
;
PROBLEM: The maximum difference density is > 0.1*ZMAX*0.75
RESPONSE: ...
;
_vrf_PLAT043__144830_1
;
PROBLEM: Calculated and Reported Mol. Weight Differ by ..      14.04 Check
RESPONSE: ...
;
_vrf_PLAT230__144830_1
;
PROBLEM: Hirshfeld Test Diff for    S1       --O6       .        7.8 s.u.
RESPONSE: ...
;
_vrf_PLAT241__144830_1
;
PROBLEM: High   'MainMol' Ueq as Compared to Neighbors of        C26 Check
RESPONSE: ...
;
_vrf_PLAT364__144830_1
;
PROBLEM: Short  C(sp3)-C(sp)  Bond  C2       - C28      .       1.22 Ang.
RESPONSE: ...
;
_vrf_PLAT413__144830_1
;
PROBLEM: Short Inter XH3 .. XHn     H8       ..H17      .       1.97 Ang.
RESPONSE: ...
;
_vrf_PLAT029__144830_1
;
PROBLEM: _diffrn_measured_fraction_theta_full value Low .      0.966 Why?
RESPONSE: ...
;
_vrf_PLAT041__144830_1
;
PROBLEM: Calc. and Reported SumFormula    Strings  Differ     Please Check
RESPONSE: ...
;
_vrf_PLAT042__144830_1
;
PROBLEM: Calc. and Reported MoietyFormula Strings  Differ     Please Check
RESPONSE: ...
;
_vrf_PLAT068__144830_1
;
PROBLEM: Reported F000 Differs from Calcd (or Missing)...     Please Check
RESPONSE: ...
;
_vrf_PLAT166__144830_1
;
PROBLEM: S.U's Given on Coordinates for Calc-flagged ....         H1 Note
RESPONSE: ...
;
_vrf_PLAT202__144830_1
```

```
;
PROBLEM: Isotropic non-H Atoms in Anion/Solvent .........          3 Check
RESPONSE: ...
;
_vrf_PLAT213__144830_1
;
PROBLEM: Atom C26             has ADP max/min Ratio .....        3.2 prolat
RESPONSE: ...
;
_vrf_PLAT220__144830_1
;
PROBLEM: NonSolvent   Resd 1  C   Ueq(max)/Ueq(min) Range        4.6 Ratio
RESPONSE: ...
;
_vrf_PLAT222__144830_1
;
PROBLEM: NonSolvent Resd 1  H   Uiso(max)/Uiso(min) Range       10.0 Ratio
RESPONSE: ...
;
_vrf_PLAT242__144830_1
;
PROBLEM: Low    'MainMol' Ueq as Compared to Neighbors of         O4 Check
RESPONSE: ...
;
_vrf_PLAT243__144830_1
;
PROBLEM: High   'Solvent' Ueq as Compared to Neighbors of        C28 Check
RESPONSE: ...
;
_vrf_PLAT245__144830_1
;
PROBLEM: U(iso) H3        Smaller than U(eq) C17       by      0.026 Ang**2
RESPONSE: ...
;
_vrf_PLAT360__144830_1
;
PROBLEM: Short  C(sp3)-C(sp3) Bond  C25      - C26      .       1.34 Ang.
RESPONSE: ...
;
# end Validation Reply Form
```

PLATON version of 04/06/2025; check.def file version of 30/05/2025

Datablock _144830_1 - ellipsoid plot

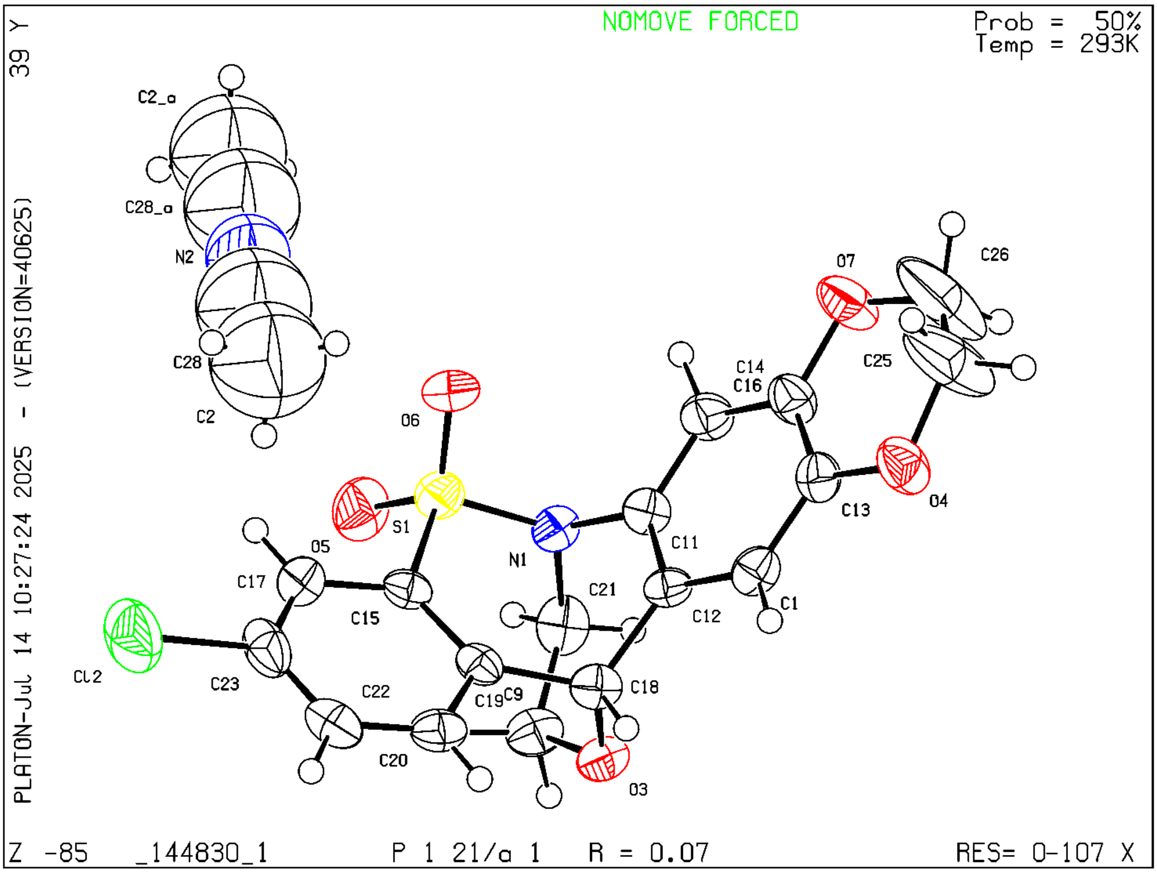

Supplement: File 2 — Crystallographic information files, checkcif and structure report files for compounds 20e, 21g, 23a, 25–27. [file compound 27 checkcif.pdf › compound 27 checkcif.pdf]
