# Supplementary material for: Synthesis of new tetra- and pentacyclic, methylenedioxy- and ethylenedioxy-substituted derivatives of the dibenzo[c,f][1,2]thiazepine ring system
Source: Beilstein J Org Chem. 2025 Dec 9;21:2645–56. doi: 10.3762/bjoc.21.205 (PMC12706374; doi:10.3762/bjoc.21.205)

checkCIF/PLATON report

You have not supplied any structure factors. As a result the full set of tests cannot be run.

THIS REPORT IS FOR GUIDANCE ONLY. IF USED AS PART OF A REVIEW PROCEDURE FOR PUBLICATION, IT SHOULD NOT REPLACE THE EXPERTISE OF AN EXPERIENCED CRYSTALLOGRAPHIC REFEREE.

No syntax errors found. CIF dictionary Interpreting this report

Datablock: _143750_1

```
Bond precision:   C-C = 0.0042 A          Wavelength=1.54187

Cell:             a=10.1431(5)     b=37.2421(15)       c=10.4416(5)
                  alpha=90         beta=105.318(2)     gamma=90
Temperature:      293 K
```

	Calculated	Reported
Volume	3804.2(3)	3804.2(3)
Space group	P 21/a	P 1 21/a 1
Hall group	-P 2yab	-P 2yab
Moiety formula	C19 H19 Cl N2 O4 S	C19 H19 Cl N2 O4 S
Sum formula	C19 H19 Cl N2 O4 S	C19 H19 Cl N2 O4 S
Mr	406.87	406.88
Dx,g cm-3	1.421	1.421
Z	8	8
Mu (mm-1)	3.048	3.048
F000	1696.0	1696.0
F000'	1706.21	
h,k,lmax	12,45,12	11,45,12
Nref	7454	7156
Tmin,Tmax	0.702,0.833	0.643,0.833
Tmin'	0.445	

```
Correction method= # Reported T Limits: Tmin=0.643 Tmax=0.833
AbsCorr = MULTI-SCAN

Data completeness= 0.960          Theta(max)= 71.750

R(reflections)= 0.0424( 15512)                wR2(reflections)=
                                              0.0569( 15714)
S = 1.015                 Npar= 525
```

The following ALERTS were generated. Each ALERT has the format
test-name_ALERT_alert-type_alert-level.
Click on the hyperlinks for more details of the test.

Alert level A

```
DIFMN02_ALERT_2_A  The minimum difference density is < -0.1*ZMAX*2.00
            _refine_diff_density_min given =     -8.850
            Test value =     -3.400
```

Author Response: Altough the residual density is out of border but there is no doubt about the chemical structure taking into account other evidences, e.g. NMR. The error maybe due to crystal imperfections.

```
PLAT097_ALERT_2_A Large Reported Max.  (Positive) Residual Density       4.69 eA-3
```

Author Response: Altough the residual density is out of border but there is no doubt about the chemical structure taking into account other evidences, e.g. NMR. The error maybe due to crystal imperfections.

```
PLAT098_ALERT_2_A Large Reported Min.  (Negative) Residual Density      -8.85 eA-3
```

Author Response: Altough the residual density is out of border but there is no doubt about the chemical structure taking into account other evidences, e.g. NMR. The error maybe due to crystal imperfections.

```
PLAT703_ALERT_1_A Torsion Calc   -170.1(2), Rep   -169.4(3), Dev..       3.50 Sigma
              N(1)-S(1)-C(1)-C(31  1_555  1_555  1_555  1_555      #   10 Check
```

Author Response: The structure is affected by some strain. Crystal imperfections can not be excluded either.

```
PLAT703_ALERT_1_A Torsion Calc    -44.7(2), Rep    -43.7(3), Dev..       5.00 Sigma
              C(50-N(2)-C(17-C(48  1_555  1_555  1_555  1_555      #   45 Check
```

Author Response: The structure is affected by some strain. Crystal imperfections can not be excluded either.

```
PLAT703_ALERT_1_A Torsion Calc -174.02(19), Rep   -175.0(3), Dev..       5.16 Sigma
              C(50-N(2)-C(35-C(29  1_555  1_555  1_555  1_555      #   47 Check
```

Author Response: The structure is affected by some strain. Crystal imperfections can not be excluded either.

```
PLAT703_ALERT_1_A Torsion Calc     56.2(2), Rep     55.4(4), Dev..       4.00 Sigma
              C(50-N(2)-C(35-C(44  1_555  1_555  1_555  1_555      #   48 Check
```

Author Response: The structure is affected by some strain. Crystal imperfections can not be excluded either.

```
PLAT703_ALERT_1_A Torsion Calc     55.5(2), Rep     54.6(5), Dev..       4.50 Sigma
              C(45-N(3)-C(28-C(30  1_555  1_555  1_555  1_555      #   50 Check
```

Author Response: The structure is affected by some strain. Crystal imperfections can not be excluded either.

```
PLAT703_ALERT_1_A Torsion Calc -176.33(18), Rep   -177.1(3), Dev..       4.28 Sigma
              C(45-N(3)-C(28-C(42  1_555  1_555  1_555  1_555      #   51 Check
```

Author Response: The structure is affected by some strain. Crystal imperfections can not be excluded either.

```
PLAT703_ALERT_1_A Torsion Calc    -24.9(3), Rep    -23.9(5), Dev..       3.33 Sigma
              N(3)-C(45-C(51-C(52  1_555  1_555  1_555  1_555      #  136 Check
```

Author Response: The structure is affected by some strain. Crystal imperfections can not be excluded either.

```
PLAT703_ALERT_1_A Torsion Calc     -0.8(3), Rep     -2.1(5), Dev..       4.33 Sigma
              C(45-C(51-C(52-C(46  1_555  1_555  1_555  1_555      #  140 Check
```

Author Response: The structure is affected by some strain. Crystal imperfections can not be excluded either.

Alert level B

```
REFLT02_ALERT_1_B  The number of reflections greater than the sigma threshold
            cannot exceed the number of symmetry-independent reflections
            Number of symmetry-independent reflections =   7156
            Number of reflections greater than sigma threshold =  15512
PLAT230_ALERT_2_B Hirshfeld Test Diff for    S2       --O9       .        7.9 s.u.
PLAT230_ALERT_2_B Hirshfeld Test Diff for    S2       --O12      .        7.9 s.u.
PLAT230_ALERT_2_B Hirshfeld Test Diff for    O6       --C37      .        7.7 s.u.
PLAT230_ALERT_2_B Hirshfeld Test Diff for    N3       --C46      .        7.3 s.u.
PLAT230_ALERT_2_B Hirshfeld Test Diff for    S1       --O8       .       10.5 s.u.
PLAT230_ALERT_2_B Hirshfeld Test Diff for    S1       --N1       .        8.3 s.u.
PLAT230_ALERT_2_B Hirshfeld Test Diff for    O2       --C41      .        8.0 s.u.
PLAT230_ALERT_2_B Hirshfeld Test Diff for    O2       --C54      .        7.5 s.u.
PLAT230_ALERT_2_B Hirshfeld Test Diff for    N2       --C17      .        7.7 s.u.
PLAT230_ALERT_2_B Hirshfeld Test Diff for    C1       --C31      .        7.3 s.u.
PLAT703_ALERT_1_B Torsion Calc   -176.4(3), Rep   -175.7(6), Dev..       2.33 Sigma
              C(47-O(3)-C(36-C(25  1_555  1_555  1_555  1_555      #   29 Check
```

Author Response: The structure is affected by some strain. Crystal imperfections can not be excluded either.

```
PLAT703_ALERT_1_B Torsion Calc   -164.6(2), Rep   -165.1(4), Dev..       2.50 Sigma
             C(28-N(3)-C(46-C(52  1_555  1_555  1_555  1_555      #   52 Check
```

Author Response: The structure is affected by some strain. Crystal imperfections can not be excluded either.

```
PLAT703_ALERT_1_B Torsion Calc    172.1(2), Rep    171.6(4), Dev..       2.50 Sigma
             C(46-N(3)-C(28-C(30  1_555  1_555  1_555  1_555      #   53 Check
```

Author Response: The structure is affected by some strain. Crystal imperfections can not be excluded either.

```
PLAT703_ALERT_1_B Torsion Calc    174.6(2), Rep    175.2(4), Dev..       3.00 Sigma
             S(1)-C(1)-C(29-C(40  1_555  1_555  1_555  1_555      #   62 Check
```

Author Response: The structure is affected by some strain. Crystal imperfections can not be excluded either.

```
PLAT703_ALERT_1_B Torsion Calc    179.8(2), Rep   -179.7(4), Dev..       2.50 Sigma
             N(1)-C(2)-C(39-C(26  1_555  1_555  1_555  1_555      #   67 Check
```

Author Response: The structure is affected by some strain. Crystal imperfections can not be excluded either.

```
PLAT703_ALERT_1_B Torsion Calc   -132.7(2), Rep   -133.2(4), Dev..       2.50 Sigma
             C(40-C(29-C(35-C(44  1_555  1_555  1_555  1_555      #  113 Check
```

Author Response: The structure is affected by some strain. Crystal imperfections can not be excluded either.

```
PLAT703_ALERT_1_B Torsion Calc     26.4(3), Rep     27.3(5), Dev..       3.00 Sigma
             N(3)-C(46-C(52-C(51  1_555  1_555  1_555  1_555      #  137 Check
```

Author Response: The structure is affected by some strain. Crystal imperfections can not be excluded either.

```
PLAT703_ALERT_1_B Torsion Calc    -29.2(2), Rep    -28.7(4), Dev..       2.50 Sigma
             N(2)-C(50-C(53-C(48  1_555  1_555  1_555  1_555      #  139 Check
```

Author Response: The structure is affected by some strain. Crystal imperfections can not be excluded either.

Alert level C

```
DIFMN03_ALERT_1_C  The minimum difference density is < -0.1*ZMAX*0.75
           The relevant atom site should be identified.
DIFMX02_ALERT_1_C  The maximum difference density is > 0.1*ZMAX*0.75
           The relevant atom site should be identified.
PLAT029_ALERT_3_C _diffrn_measured_fraction_theta_full value Low .      0.960 Why?
PLAT230_ALERT_2_C Hirshfeld Test Diff for    O6        --C47       .        7.0 s.u.
PLAT230_ALERT_2_C Hirshfeld Test Diff for    C1        --C29       .        5.7 s.u.
PLAT230_ALERT_2_C Hirshfeld Test Diff for    C26       --C41       .        7.0 s.u.
PLAT241_ALERT_2_C High   'MainMol' Ueq as Compared to Neighbors of        C54 Check
PLAT242_ALERT_2_C Low    'MainMol' Ueq as Compared to Neighbors of        C49 Check
PLAT340_ALERT_3_C Low Bond Precision on  C-C Bonds ..............    0.00418 Ang.
PLAT601_ALERT_2_C Unit Cell Contains Solvent Accessible VOIDS   <=         32 Ang**3
PLAT703_ALERT_1_C Torsion Calc   -101.5(3), Rep   -101.9(5), Dev..       1.33 Sigma
              O(1)-S(1)-C(1)-C(29  1_555  1_555  1_555  1_555      #    3 Check
```

Author Response: The structure is affected by some strain. Crystal imperfections can not be excluded either.

```
PLAT703_ALERT_1_C Torsion Calc     74.0(2), Rep     74.4(4), Dev..       2.00 Sigma
              O(1)-S(1)-C(1)-C(31  1_555  1_555  1_555  1_555      #    4 Check
```

Author Response: The structure is affected by some strain. Crystal imperfections can not be excluded either.

```
PLAT703_ALERT_1_C Torsion Calc    131.0(3), Rep    130.5(4), Dev..       1.67 Sigma
              O(8)-S(1)-C(1)-C(29  1_555  1_555  1_555  1_555      #    7 Check
```

Author Response: The structure is affected by some strain. Crystal imperfections can not be excluded either.

```
PLAT703_ALERT_1_C Torsion Calc    -53.5(2), Rep    -53.2(4), Dev..       1.50 Sigma
              O(8)-S(1)-C(1)-C(31  1_555  1_555  1_555  1_555      #    8 Check
```

Author Response: The structure is affected by some strain. Crystal imperfections can not be excluded either.

```
PLAT703_ALERT_1_C Torsion Calc     -6.0(2), Rep     -6.3(3), Dev..       1.50 Sigma
              O(12-S(2)-N(4)-C(21  1_555  1_555  1_555  1_555      #   17 Check
```

Author Response: The structure is affected by some strain. Crystal imperfections can not be excluded either.

```
PLAT703_ALERT_1_C Torsion Calc -177.03(17), Rep   -176.7(2), Dev..       1.94 Sigma
              N(4)-S(2)-C(32-C(23  1_555  1_555  1_555  1_555      #   21 Check
```

Author Response: The structure is affected by some strain. Crystal imperfections can not be excluded either.

```
PLAT703_ALERT_1_C Torsion Calc      8.5(3), Rep      9.0(6), Dev..       1.67 Sigma
              C(37-O(6)-C(47-O(3)  1_555  1_555  1_555  1_555      #   31 Check
```

Author Response: The structure is affected by some strain. Crystal imperfections can not be excluded either.

```
PLAT703_ALERT_1_C Torsion Calc     -5.8(4), Rep     -6.4(6), Dev..       1.50 Sigma
              C(47-O(6)-C(37-C(36  1_555  1_555  1_555  1_555      #   33 Check
```

Author Response: The structure is affected by some strain. Crystal imperfections can not be excluded either.

```
PLAT703_ALERT_1_C Torsion Calc     80.9(3), Rep     80.5(5), Dev..       1.33 Sigma
              C(27-N(1)-C(2)-C(39  1_555  1_555  1_555  1_555      #   39 Check
```

Author Response: The structure is affected by some strain. Crystal imperfections can not be excluded either.

```
PLAT703_ALERT_1_C Torsion Calc  163.97(19), Rep    164.3(3), Dev..       1.74 Sigma
              C(28-N(3)-C(45-C(51  1_555  1_555  1_555  1_555      #   49 Check
```

Author Response: The structure is affected by some strain. Crystal imperfections can not be excluded either.

```
PLAT703_ALERT_1_C Torsion Calc   -102.8(3), Rep   -103.4(4), Dev..       2.00 Sigma
              S(2)-N(4)-C(24-C(19  1_555  1_555  1_555  1_555      #   57 Check
```

Author Response: The structure is affected by some strain. Crystal imperfections can not be excluded either.

```
PLAT703_ALERT_1_C Torsion Calc     82.7(3), Rep     82.2(5), Dev..       1.67 Sigma
              C(21-N(4)-C(24-C(19  1_555  1_555  1_555  1_555      #   59 Check
```

Author Response: The structure is affected by some strain. Crystal imperfections can not be excluded either.

```
PLAT703_ALERT_1_C Torsion Calc     -8.8(4), Rep     -8.1(8), Dev..       1.75 Sigma
              S(1)-C(1)-C(29-C(35  1_555  1_555  1_555  1_555      #   61 Check
```

Author Response: The structure is affected by some strain. Crystal imperfections can not be excluded either.

```
PLAT703_ALERT_1_C Torsion Calc      0.3(4), Rep      0.8(8), Dev..       1.25 Sigma
              C(29-C(1)-C(31-C(33  1_555  1_555  1_555  1_555      #   64 Check
```

Author Response: The structure is affected by some strain. Crystal imperfections can not be excluded either.

```
PLAT703_ALERT_1_C Torsion Calc      4.1(4), Rep      3.6(6), Dev..       1.25 Sigma
              N(1)-C(2)-C(44-C(35  1_555  1_555  1_555  1_555      #   69 Check
```

Author Response: The structure is affected by some strain. Crystal imperfections can not be excluded either.

```
PLAT703_ALERT_1_C Torsion Calc   -176.1(2), Rep   -176.4(4), Dev..       1.50 Sigma
              C(39-C(2)-C(44-C(35  1_555  1_555  1_555  1_555      #   71 Check
```

Author Response: The structure is affected by some strain. Crystal imperfections can not be excluded either.

```
PLAT703_ALERT_1_C Torsion Calc    176.6(2), Rep    177.0(4), Dev..       2.00 Sigma
              C(41-C(3)-C(44-C(35  1_555  1_555  1_555  1_555      #   74 Check
```

Author Response: The structure is affected by some strain. Crystal imperfections can not be excluded either.

```
PLAT703_ALERT_1_C Torsion Calc   -179.2(3), Rep   -178.8(4), Dev..       1.33 Sigma
              C(44-C(3)-C(41-O(2)  1_555  1_555  1_555  1_555      #   75 Check
```

Author Response: The structure is affected by some strain. Crystal imperfections can not be excluded either.

```
PLAT703_ALERT_1_C Torsion Calc     25.7(2), Rep     25.4(3), Dev..       1.50 Sigma
              N(2)-C(17-C(48-C(53  1_555  1_555  1_555  1_555      #   77 Check
```

Author Response: The structure is affected by some strain. Crystal imperfections can not be excluded either.

```
PLAT703_ALERT_1_C Torsion Calc     -0.8(5), Rep      0.0(8), Dev..       1.60 Sigma
              C(24-C(19-C(37-C(36  1_555  1_555  1_555  1_555      #   79 Check
```

Author Response: The structure is affected by some strain. Crystal imperfections can not be excluded either.

```
PLAT703_ALERT_1_C Torsion Calc     -0.4(4), Rep     -0.9(7), Dev..       1.25 Sigma
              C(37-C(19-C(24-C(30  1_555  1_555  1_555  1_555      #   81 Check
```

Author Response: The structure is affected by some strain. Crystal imperfections can not be excluded either.

```
PLAT703_ALERT_1_C Torsion Calc  178.16(19), Rep    177.8(3), Dev..       1.89 Sigma
            C(32-C(23-C(49-CL(1  1_555  1_555  1_555  1_555      #   82 Check
```

Author Response: The structure is affected by some strain. Crystal imperfections can not be excluded either.

```
PLAT703_ALERT_1_C Torsion Calc     -0.4(4), Rep      0.2(5), Dev..       1.50 Sigma
            C(32-C(23-C(49-C(38  1_555  1_555  1_555  1_555      #   83 Check
```

Author Response: The structure is affected by some strain. Crystal imperfections can not be excluded either.

```
PLAT703_ALERT_1_C Torsion Calc -176.48(19), Rep   -176.8(3), Dev..       1.68 Sigma
            C(49-C(23-C(32-S(2)  1_555  1_555  1_555  1_555      #   84 Check
```

Author Response: The structure is affected by some strain. Crystal imperfections can not be excluded either.

```
PLAT703_ALERT_1_C Torsion Calc      2.5(4), Rep      2.0(7), Dev..       1.25 Sigma
            N(4)-C(24-C(30-C(28  1_555  1_555  1_555  1_555      #   87 Check
```

Author Response: The structure is affected by some strain. Crystal imperfections can not be excluded either.

```
PLAT703_ALERT_1_C Torsion Calc      0.7(4), Rep      1.2(7), Dev..       1.25 Sigma
            C(19-C(24-C(30-C(25  1_555  1_555  1_555  1_555      #   88 Check
```

Author Response: The structure is affected by some strain. Crystal imperfections can not be excluded either.

```
PLAT703_ALERT_1_C Torsion Calc     -1.1(5), Rep     -0.3(7), Dev..       1.60 Sigma
            C(30-C(25-C(36-C(37  1_555  1_555  1_555  1_555      #   91 Check
```

Author Response: The structure is affected by some strain. Crystal imperfections can not be excluded either.

```
PLAT703_ALERT_1_C Torsion Calc      0.0(4), Rep     -0.6(7), Dev..       1.50 Sigma
            C(36-C(25-C(30-C(24  1_555  1_555  1_555  1_555      #   92 Check
```

Author Response: The structure is affected by some strain. Crystal imperfections can not be excluded either.

```
PLAT703_ALERT_1_C Torsion Calc     -0.4(4), Rep     -1.0(6), Dev..       1.50 Sigma
             O(7)-C(26-C(41-O(2)  1_555  1_555  1_555  1_555      #   95 Check
```

Author Response: The structure is affected by some strain. Crystal imperfections can not be excluded either.

```
PLAT703_ALERT_1_C Torsion Calc      1.7(5), Rep      2.6(9), Dev..       1.80 Sigma
             C(39-C(26-C(41-C(3)  1_555  1_555  1_555  1_555      #   98 Check
```

Author Response: The structure is affected by some strain. Crystal imperfections can not be excluded either.

```
PLAT703_ALERT_1_C Torsion Calc     -1.2(4), Rep     -2.0(8), Dev..       2.00 Sigma
             C(41-C(26-C(39-C(2)  1_555  1_555  1_555  1_555      #   99 Check
```

Author Response: The structure is affected by some strain. Crystal imperfections can not be excluded either.

```
PLAT703_ALERT_1_C Torsion Calc     50.7(4), Rep     50.1(7), Dev..       1.50 Sigma
             C(1)-C(29-C(35-C(44  1_555  1_555  1_555  1_555      #  109 Check
```

Author Response: The structure is affected by some strain. Crystal imperfections can not be excluded either.

```
PLAT703_ALERT_1_C Torsion Calc     -0.8(5), Rep     -1.4(8), Dev..       1.20 Sigma
             C(1)-C(31-C(33-C(43  1_555  1_555  1_555  1_555      #  115 Check
```

Author Response: The structure is affected by some strain. Crystal imperfections can not be excluded either.

```
PLAT703_ALERT_1_C Torsion Calc  176.38(19), Rep    176.6(3), Dev..       1.16 Sigma
             S(2)-C(32-C(42-C(34  1_555  1_555  1_555  1_555      #  117 Check
```

Author Response: The structure is affected by some strain. Crystal imperfections can not be excluded either.

```
PLAT703_ALERT_1_C Torsion Calc    179.4(2), Rep    179.1(3), Dev..       1.50 Sigma
             C(38-C(34-C(42-C(28  1_555  1_555  1_555  1_555      #  122 Check
```

Author Response: The structure is affected by some strain. Crystal imperfections can not be excluded either.

```
PLAT703_ALERT_1_C Torsion Calc     -0.4(4), Rep      0.3(6), Dev..       1.75 Sigma
             C(42-C(34-C(38-C(49  1_555  1_555  1_555  1_555      #  124 Check
```

Author Response: The structure is affected by some strain. Crystal imperfections can not be excluded either.

```
PLAT703_ALERT_1_C Torsion Calc    -67.3(3), Rep    -66.7(5), Dev..       2.00 Sigma
              C(29-C(35-C(44-C(2)  1_555  1_555  1_555  1_555      #  127 Check
```

Author Response: The structure is affected by some strain. Crystal imperfections can not be excluded either.

```
PLAT703_ALERT_1_C Torsion Calc      1.1(4), Rep      1.7(7), Dev..       1.50 Sigma
              O(3)-C(36-C(37-O(6)  1_555  1_555  1_555  1_555      #  129 Check
```

Author Response: The structure is affected by some strain. Crystal imperfections can not be excluded either.

```
PLAT703_ALERT_1_C Torsion Calc      1.5(6), Rep      0.6(9), Dev..       1.50 Sigma
              C(25-C(36-C(37-C(19  1_555  1_555  1_555  1_555      #  132 Check
```

Author Response: The structure is affected by some strain. Crystal imperfections can not be excluded either.

```
PLAT703_ALERT_1_C Torsion Calc      1.1(4), Rep      0.3(6), Dev..       2.00 Sigma
              C(34-C(38-C(49-C(23  1_555  1_555  1_555  1_555      #  134 Check
```

Author Response: The structure is affected by some strain. Crystal imperfections can not be excluded either.

```
PLAT790_ALERT_4_C Centre of Gravity not Within Unit Cell: Resd.  #          1 Note
              C19 H19 Cl N2 O4 S
```

Alert level G

```
PLAT005_ALERT_5_G No Embedded Refinement Details Found  in the CIF     Please Do !
PLAT128_ALERT_4_G Alternate Setting for Input Space Group  P21/a        P21/c Note
PLAT199_ALERT_1_G Reported _cell_measurement_temperature ..... (K)        293 Check
PLAT200_ALERT_1_G Reported   _diffrn_ambient_temperature ..... (K)        293 Check
PLAT398_ALERT_2_G Deviating  C-O-C   Angle From 120 for O3       .      104.6 Degree
PLAT398_ALERT_2_G Deviating  C-O-C   Angle From 120 for O6       .      104.5 Degree
PLAT398_ALERT_2_G Deviating  C-O-C   Angle From 120 for O2       .      103.5 Degree
PLAT398_ALERT_2_G Deviating  C-O-C   Angle From 120 for O7       .      105.2 Degree
PLAT793_ALERT_4_G Model has Chirality at C28         (Centro SpGr)          R Verify
PLAT793_ALERT_4_G Model has Chirality at C35         (Centro SpGr)          S Verify
PLAT808_ALERT_5_G No Parseable SHELXL Style Weighting Scheme Found     Please Check
PLAT882_ALERT_1_G No Datum for _diffrn_reflns_av_unetI/netI ......     Please Do !
PLAT883_ALERT_1_G Absent Datum for _atom_sites_solution_primary ..     Please Do !
```

---

11 **ALERT level A** = Most likely a serious problem - resolve or explain
19 **ALERT level B** = A potentially serious problem, consider carefully

```
51 ALERT level C = Check. Ensure it is not caused by an omission or oversight
13 ALERT level G = General information/check it is not something unexpected

63 ALERT type 1 CIF construction/syntax error, inconsistent or missing data
23 ALERT type 2 Indicator that the structure model may be wrong or deficient
 2 ALERT type 3 Indicator that the structure quality may be low
 4 ALERT type 4 Improvement, methodology, query or suggestion
 2 ALERT type 5 Informative message, check
```

It is advisable to attempt to resolve as many as possible of the alerts in all categories. Often the minor alerts point to easily fixed oversights, errors and omissions in your CIF or refinement strategy, so attention to these fine details can be worthwhile. In order to resolve some of the more serious problems it may be necessary to carry out additional measurements or structure refinements. However, the purpose of your study may justify the reported deviations and the more serious of these should normally be commented upon in the discussion or experimental section of a paper or in the "special_details" fields of the CIF. checkCIF was carefully designed to identify outliers and unusual parameters, but every test has its limitations and alerts that are not important in a particular case may appear. Conversely, the absence of alerts does not guarantee there are no aspects of the results needing attention. It is up to the individual to critically assess their own results and, if necessary, seek expert advice.

Publication of your CIF in IUCr journals

A basic structural check has been run on your CIF. These basic checks will be run on all CIFs submitted for publication in IUCr journals (*Acta Crystallographica*, *Journal of Applied Crystallography*, *Journal of Synchrotron Radiation*); however, if you intend to submit to *Acta Crystallographica Section C* or *E* or *IUCrData*, you should make sure that full publication checks are run on the final version of your CIF prior to submission.

Publication of your CIF in other journals

Please refer to the *Notes for Authors* of the relevant journal for any special instructions relating to CIF submission.

Validation response form

Please find below a validation response form (VRF) that can be filled in and pasted into your CIF.

```
# start Validation Reply Form
_vrf_REFLT02__143750_1
;
PROBLEM: The number of reflections greater than the sigma threshold
RESPONSE: ...
;
_vrf_DIFMN03__143750_1
;
PROBLEM: The minimum difference density is < -0.1*ZMAX*0.75
RESPONSE: ...
;
_vrf_DIFMX02__143750_1
```

```
;
PROBLEM: The maximum difference density is > 0.1*ZMAX*0.75
RESPONSE: ...
;
_vrf_PLAT230__143750_1
;
PROBLEM: Hirshfeld Test Diff for    S2       --O9       .        7.9 s.u.
RESPONSE: ...
;
_vrf_PLAT029__143750_1
;
PROBLEM: _diffrn_measured_fraction_theta_full value Low .      0.960 Why?
RESPONSE: ...
;
_vrf_PLAT241__143750_1
;
PROBLEM: High   'MainMol' Ueq as Compared to Neighbors of        C54 Check
RESPONSE: ...
;
_vrf_PLAT242__143750_1
;
PROBLEM: Low    'MainMol' Ueq as Compared to Neighbors of        C49 Check
RESPONSE: ...
;
_vrf_PLAT340__143750_1
;
PROBLEM: Low Bond Precision on  C-C Bonds ...............    0.00418 Ang.
RESPONSE: ...
;
_vrf_PLAT601__143750_1
;
PROBLEM: Unit Cell Contains Solvent Accessible VOIDS   <=         32 Ang**3
RESPONSE: ...
;
_vrf_PLAT790__143750_1
;
PROBLEM: Centre of Gravity not Within Unit Cell: Resd.  #          1 Note
RESPONSE: ...
;
# end Validation Reply Form
```

PLATON version of 04/06/2025; check.def file version of 30/05/2025

Datablock _143750_1 - ellipsoid plot

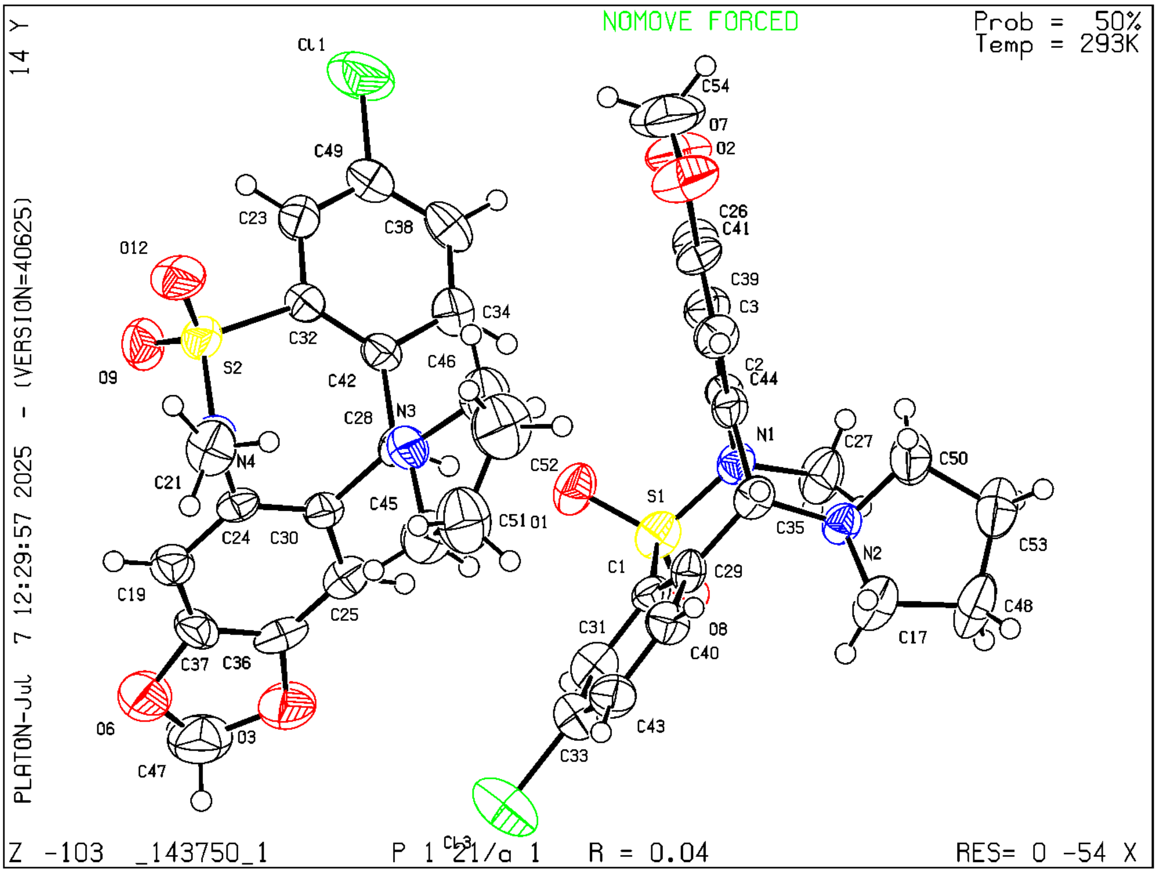

Supplement: File 2 — Crystallographic information files, checkcif and structure report files for compounds 20e, 21g, 23a, 25–27. [file compound 20e checkcif.pdf › compound 20e checkcif.pdf]
