# Supplementary material for: Synthesis of new tetra- and pentacyclic, methylenedioxy- and ethylenedioxy-substituted derivatives of the dibenzo[c,f][1,2]thiazepine ring system
Source: Beilstein J Org Chem. 2025 Dec 9;21:2645–56. doi: 10.3762/bjoc.21.205 (PMC12706374; doi:10.3762/bjoc.21.205)

checkCIF/PLATON report

You have not supplied any structure factors. As a result the full set of tests cannot be run.

THIS REPORT IS FOR GUIDANCE ONLY. IF USED AS PART OF A REVIEW PROCEDURE FOR PUBLICATION, IT SHOULD NOT REPLACE THE EXPERTISE OF AN EXPERIENCED CRYSTALLOGRAPHIC REFEREE.

No syntax errors found. CIF dictionary Interpreting this report

Datablock: _143734_1

```
Bond precision:   C-C = 0.0059 A          Wavelength=1.54187

Cell:             a=10.3705(8)     b=37.377(3)          c=10.6257(8)
                  alpha=90         beta=104.647(3)      gamma=90
Temperature:      293 K

                 Calculated                    Reported
Volume           3984.9(5)                     3984.8(5)
Space group      P 21/c                        P 1 21/c 1
Hall group       -P 2ybc                       -P 2ybc
Moiety formula   C20 H21 Cl N2 O4 S            C20 H21 Cl N2 O4 S
Sum formula      C20 H21 Cl N2 O4 S            C20 H21 Cl N2 O4 S
Mr               420.90                        420.91
Dx,g cm-3        1.403                         1.403
Z                8                             8
Mu (mm-1)        2.928                         2.929
F000             1760.0                        1760.0
F000'            1770.35
h,k,lmax         12,46,13                      12,46,12
Nref             7797                          7615
Tmin,Tmax        0.869,0.889                   0.706,0.884
Tmin'            0.348

Correction method= # Reported T Limits: Tmin=0.706 Tmax=0.884
AbsCorr = MULTI-SCAN

Data completeness= 0.977          Theta(max)= 71.770

R(reflections)= 0.0767( 20535)                       wR2(reflections)=
                                                     0.1030( 20815)
S = 1.074                  Npar= 547
```

The following ALERTS were generated. Each ALERT has the format
test-name_ALERT_alert-type_alert-level.
Click on the hyperlinks for more details of the test.

Alert level A

```
DIFMN02_ALERT_2_A  The minimum difference density is < -0.1*ZMAX*2.00
            _refine_diff_density_min given =    -12.600
            Test value =     -3.400
```

Author Response: Altough the residual density is out of border but there is no doubt about the chemical structure taking into account other evidences, e.g. NMR. The error maybe due to crystal imperfections.

```
PLAT097_ALERT_2_A Large Reported Max.  (Positive) Residual Density       5.03 eA-3
```

Author Response: Altough the residual density is out of border but there is no doubt about the chemical structure taking into account other evidences, e.g. NMR. The error maybe due to crystal imperfections.

```
PLAT098_ALERT_2_A Large Reported Min.  (Negative) Residual Density     -12.60 eA-3
```

Author Response: Altough the residual density is out of border but there is no doubt about the chemical structure taking into account other evidences, e.g. NMR. The error maybe due to crystal imperfections.

```
PLAT703_ALERT_1_A Torsion Calc     60.6(3), Rep     59.5(5), Dev..       3.67 Sigma
              C(39-N(2)-C(45-C(3)  1_555  1_555  1_555  1_555      #   46 Check
```

Author Response: The structure is affected by some strain. Crystal imperfections can not be excluded either.

Alert level B

```
REFLT02_ALERT_1_B  The number of reflections greater than the sigma threshold
            cannot exceed the number of symmetry-independent reflections
            Number of symmetry-independent reflections =   7615
            Number of reflections greater than sigma threshold =  20535
PLAT230_ALERT_2_B Hirshfeld Test Diff for    Cl1      --C29      .        8.3 s.u.
PLAT230_ALERT_2_B Hirshfeld Test Diff for    N13      --C28      .        8.3 s.u.
PLAT230_ALERT_2_B Hirshfeld Test Diff for    S1       --O6       .        7.5 s.u.
PLAT230_ALERT_2_B Hirshfeld Test Diff for    O9       --C41      .        7.2 s.u.
PLAT230_ALERT_2_B Hirshfeld Test Diff for    O9       --C54      .        7.8 s.u.
PLAT230_ALERT_2_B Hirshfeld Test Diff for    C22      --C33      .        7.7 s.u.
PLAT241_ALERT_2_B High   'MainMol' Ueq as Compared to Neighbors of       C49 Check
PLAT241_ALERT_2_B High   'MainMol' Ueq as Compared to Neighbors of       C55 Check
PLAT360_ALERT_2_B Short  C(sp3)-C(sp3) Bond  C49      - C56      .       1.30 Ang.
PLAT360_ALERT_2_B Short  C(sp3)-C(sp3) Bond  C54      - C55      .       1.30 Ang.
```

```
PLAT410_ALERT_2_B Short Intra H...H Contact  H23       ..H36      .       1.87 Ang.
                                                       x,y,z  =     1_555 Check
PLAT410_ALERT_2_B Short Intra H...H Contact  H24       ..H35      .       1.87 Ang.
                                                       x,y,z  =     1_555 Check
PLAT410_ALERT_2_B Short Intra H...H Contact  H31       ..H34      .       1.88 Ang.
                                                       x,y,z  =     1_555 Check
PLAT410_ALERT_2_B Short Intra H...H Contact  H32       ..H33      .       1.89 Ang.
                                                       x,y,z  =     1_555 Check
PLAT703_ALERT_1_B Torsion Calc    178.5(2), Rep    177.9(3), Dev..       3.00 Sigma
              O(6)-S(1)-N(1)-C(24  1_555  1_555  1_555  1_555      #    1 Check
```

Author Response: The structure is affected by some strain. Crystal imperfections can not be excluded either.

```
PLAT703_ALERT_1_B Torsion Calc    178.8(4), Rep    179.8(5), Dev..       2.50 Sigma
              C(49-O(12-C(43-C(30  1_555  1_555  1_555  1_555      #   35 Check
```

Author Response: The structure is affected by some strain. Crystal imperfections can not be excluded either.

```
PLAT703_ALERT_1_B Torsion Calc    -54.1(3), Rep    -53.2(5), Dev..       3.00 Sigma
              C(34-N(2)-C(45-C(20  1_555  1_555  1_555  1_555      #   44 Check
```

Author Response: The structure is affected by some strain. Crystal imperfections can not be excluded either.

```
PLAT703_ALERT_1_B Torsion Calc   -179.1(3), Rep   -178.4(5), Dev..       2.33 Sigma
              C(32-C(2)-C(24-N(1)  1_555  1_555  1_555  1_555      #   73 Check
```

Author Response: The structure is affected by some strain. Crystal imperfections can not be excluded either.

```
PLAT703_ALERT_1_B Torsion Calc    -99.9(4), Rep    -99.0(5), Dev..       2.25 Sigma
              C(26-C(3)-C(45-N(2)  1_555  1_555  1_555  1_555      #   78 Check
```

Author Response: The structure is affected by some strain. Crystal imperfections can not be excluded either.

```
PLAT703_ALERT_1_B Torsion Calc    -49.6(5), Rep    -50.7(7), Dev..       2.20 Sigma
              C(38-C(3)-C(45-C(20  1_555  1_555  1_555  1_555      #   82 Check
```

Author Response: The structure is affected by some strain. Crystal imperfections can not be excluded either.

Alert level C

```
DIFMN03_ALERT_1_C  The minimum difference density is < -0.1*ZMAX*0.75
            The relevant atom site should be identified.
DIFMX02_ALERT_1_C  The maximum difference density is > 0.1*ZMAX*0.75
            The relevant atom site should be identified.
PLAT029_ALERT_3_C _diffrn_measured_fraction_theta_full value Low .      0.977 Why?
PLAT213_ALERT_2_C Atom C49             has ADP max/min Ratio .....        3.9 prolat
PLAT213_ALERT_2_C Atom C55             has ADP max/min Ratio .....        3.5 prolat
PLAT220_ALERT_2_C NonSolvent   Resd 1  C   Ueq(max)/Ueq(min) Range        5.4 Ratio
PLAT220_ALERT_2_C NonSolvent   Resd 2  C   Ueq(max)/Ueq(min) Range        4.2 Ratio
PLAT222_ALERT_3_C NonSolvent Resd 1  H   Uiso(max)/Uiso(min) Range        4.6 Ratio
PLAT230_ALERT_2_C Hirshfeld Test Diff for    S2       --O7       .        5.7 s.u.
PLAT230_ALERT_2_C Hirshfeld Test Diff for    S2       --N13      .        7.0 s.u.
PLAT230_ALERT_2_C Hirshfeld Test Diff for    S2       --C38      .        5.5 s.u.
PLAT230_ALERT_2_C Hirshfeld Test Diff for    N2       --C34      .        6.0 s.u.
PLAT230_ALERT_2_C Hirshfeld Test Diff for    C20      --C28      .        5.5 s.u.
PLAT230_ALERT_2_C Hirshfeld Test Diff for    C20      --C45      .        7.0 s.u.
PLAT230_ALERT_2_C Hirshfeld Test Diff for    C21      --C38      .        6.0 s.u.
PLAT230_ALERT_2_C Hirshfeld Test Diff for    C28      --C37      .        6.2 s.u.
PLAT230_ALERT_2_C Hirshfeld Test Diff for    C30      --C43      .        5.5 s.u.
PLAT230_ALERT_2_C Hirshfeld Test Diff for    C34      --C46      .        5.2 s.u.
PLAT230_ALERT_2_C Hirshfeld Test Diff for    C42      --C43      .        5.8 s.u.
PLAT230_ALERT_2_C Hirshfeld Test Diff for    O8       --C55      .        5.1 s.u.
PLAT230_ALERT_2_C Hirshfeld Test Diff for    N1       --C24      .        6.7 s.u.
PLAT230_ALERT_2_C Hirshfeld Test Diff for    N3       --C35      .        5.7 s.u.
PLAT230_ALERT_2_C Hirshfeld Test Diff for    C32      --C41      .        5.2 s.u.
PLAT234_ALERT_4_C Large Hirshfeld Difference O1       --C56      .       0.16 Ang.
PLAT234_ALERT_4_C Large Hirshfeld Difference C49      --C56      .       0.18 Ang.
PLAT234_ALERT_4_C Large Hirshfeld Difference C54      --C55      .       0.16 Ang.
PLAT241_ALERT_2_C High   'MainMol' Ueq as Compared to Neighbors of        C44 Check
PLAT241_ALERT_2_C High   'MainMol' Ueq as Compared to Neighbors of        C53 Check
PLAT242_ALERT_2_C Low    'MainMol' Ueq as Compared to Neighbors of         O1 Check
PLAT242_ALERT_2_C Low    'MainMol' Ueq as Compared to Neighbors of        O12 Check
PLAT242_ALERT_2_C Low    'MainMol' Ueq as Compared to Neighbors of         N2 Check
PLAT242_ALERT_2_C Low    'MainMol' Ueq as Compared to Neighbors of         O8 Check
PLAT242_ALERT_2_C Low    'MainMol' Ueq as Compared to Neighbors of         N3 Check
PLAT340_ALERT_3_C Low Bond Precision on  C-C Bonds ...............    0.00594 Ang.
PLAT601_ALERT_2_C Unit Cell Contains Solvent Accessible VOIDS   <=         51 Ang**3
PLAT703_ALERT_1_C Torsion Calc    -51.8(3), Rep    -52.4(4), Dev..       2.00 Sigma
              O(10-S(1)-N(1)-C(24  1_555  1_555  1_555  1_555      #    5 Check
```

Author Response: The structure is affected by some strain. Crystal imperfections can not be excluded either.

```
PLAT703_ALERT_1_C Torsion Calc     61.3(3), Rep     60.7(4), Dev..       2.00 Sigma
              C(25-S(1)-N(1)-C(24  1_555  1_555  1_555  1_555      #   11 Check
```

Author Response: The structure is affected by some strain. Crystal imperfections can not be excluded either.

```
PLAT703_ALERT_1_C Torsion Calc    142.9(2), Rep    142.5(3), Dev..       2.00 Sigma
              O(5)-S(2)-N(13-C(31  1_555  1_555  1_555  1_555      #   14 Check
```

Author Response: The structure is affected by some strain. Crystal imperfections can not be excluded either.

```
PLAT703_ALERT_1_C Torsion Calc     98.4(3), Rep     97.9(5), Dev..       1.67 Sigma
              O(5)-S(2)-C(38-C(3)  1_555  1_555  1_555  1_555      #   15 Check
```

Author Response: The structure is affected by some strain. Crystal imperfections can not be excluded either.

```
PLAT703_ALERT_1_C Torsion Calc    -76.2(3), Rep    -75.6(4), Dev..       2.00 Sigma
              O(5)-S(2)-C(38-C(21  1_555  1_555  1_555  1_555      #   16 Check
```

Author Response: The structure is affected by some strain. Crystal imperfections can not be excluded either.

```
PLAT703_ALERT_1_C Torsion Calc   -133.9(3), Rep   -134.5(5), Dev..       2.00 Sigma
              O(7)-S(2)-C(38-C(3)  1_555  1_555  1_555  1_555      #   19 Check
```

Author Response: The structure is affected by some strain. Crystal imperfections can not be excluded either.

```
PLAT703_ALERT_1_C Torsion Calc     51.5(3), Rep     52.0(5), Dev..       1.67 Sigma
              O(7)-S(2)-C(38-C(21  1_555  1_555  1_555  1_555      #   20 Check
```

Author Response: The structure is affected by some strain. Crystal imperfections can not be excluded either.

```
PLAT703_ALERT_1_C Torsion Calc    -17.8(4), Rep    -18.5(6), Dev..       1.75 Sigma
              N(13-S(2)-C(38-C(3)  1_555  1_555  1_555  1_555      #   21 Check
```

Author Response: The structure is affected by some strain. Crystal imperfections can not be excluded either.

```
PLAT703_ALERT_1_C Torsion Calc    167.6(2), Rep    168.0(4), Dev..       2.00 Sigma
              N(13-S(2)-C(38-C(21  1_555  1_555  1_555  1_555      #   22 Check
```

Author Response: The structure is affected by some strain. Crystal imperfections can not be excluded either.

```
PLAT703_ALERT_1_C Torsion Calc     67.2(3), Rep     67.6(4), Dev..       1.33 Sigma
              C(38-S(2)-N(13-C(28  1_555  1_555  1_555  1_555      #   23 Check
```

Author Response: The structure is affected by some strain. Crystal imperfections can not be excluded either.

```
PLAT703_ALERT_1_C Torsion Calc     12.0(8), Rep    11.1(12), Dev..       1.12 Sigma
            C(32-O(8)-C(55-C(54  1_555  1_555  1_555  1_555      #   28 Check
```

Author Response: The structure is affected by some strain. Crystal imperfections can not be excluded either.

```
PLAT703_ALERT_1_C Torsion Calc    172.7(4), Rep    173.4(6), Dev..       1.75 Sigma
            C(54-O(9)-C(41-C(36  1_555  1_555  1_555  1_555      #   33 Check
```

Author Response: The structure is affected by some strain. Crystal imperfections can not be excluded either.

```
PLAT703_ALERT_1_C Torsion Calc    -75.3(4), Rep    -74.7(6), Dev..       1.50 Sigma
            S(1)-N(1)-C(24-C(22  1_555  1_555  1_555  1_555      #   38 Check
```

Author Response: The structure is affected by some strain. Crystal imperfections can not be excluded either.

```
PLAT703_ALERT_1_C Torsion Calc    -83.8(4), Rep    -84.3(6), Dev..       1.25 Sigma
            C(51-N(1)-C(24-C(2)  1_555  1_555  1_555  1_555      #   39 Check
```

Author Response: The structure is affected by some strain. Crystal imperfections can not be excluded either.

```
PLAT703_ALERT_1_C Torsion Calc    -44.5(3), Rep    -44.1(5), Dev..       1.33 Sigma
            C(39-N(2)-C(34-C(46  1_555  1_555  1_555  1_555      #   42 Check
```

Author Response: The structure is affected by some strain. Crystal imperfections can not be excluded either.

```
PLAT703_ALERT_1_C Torsion Calc   -166.9(3), Rep   -167.4(4), Dev..       1.67 Sigma
            C(45-N(2)-C(34-C(46  1_555  1_555  1_555  1_555      #   45 Check
```

Author Response: The structure is affected by some strain. Crystal imperfections can not be excluded either.

```
PLAT703_ALERT_1_C Torsion Calc    174.9(3), Rep    174.5(4), Dev..       1.33 Sigma
            C(35-N(3)-C(33-C(1)  1_555  1_555  1_555  1_555      #   50 Check
```

Author Response: The structure is affected by some strain. Crystal imperfections can not be excluded either.

```
PLAT703_ALERT_1_C Torsion Calc    -56.8(4), Rep    -57.3(6), Dev..       1.25 Sigma
              C(35-N(3)-C(33-C(22  1_555  1_555  1_555  1_555      #   51 Check
```

Author Response: The structure is affected by some strain. Crystal imperfections can not be excluded either.

```
PLAT703_ALERT_1_C Torsion Calc   -174.8(3), Rep   -175.2(5), Dev..       1.33 Sigma
              C(52-N(3)-C(33-C(22  1_555  1_555  1_555  1_555      #   54 Check
```

Author Response: The structure is affected by some strain. Crystal imperfections can not be excluded either.

```
PLAT703_ALERT_1_C Torsion Calc   -179.8(3), Rep   -179.4(4), Dev..       1.33 Sigma
              C(33-C(1)-C(25-C(27  1_555  1_555  1_555  1_555      #   64 Check
```

Author Response: The structure is affected by some strain. Crystal imperfections can not be excluded either.

```
PLAT703_ALERT_1_C Torsion Calc   -174.0(3), Rep   -173.5(4), Dev..       1.67 Sigma
              C(26-C(3)-C(38-S(2)  1_555  1_555  1_555  1_555      #   75 Check
```

Author Response: The structure is affected by some strain. Crystal imperfections can not be excluded either.

```
PLAT703_ALERT_1_C Torsion Calc      0.1(5), Rep     -0.6(8), Dev..       1.40 Sigma
              C(26-C(3)-C(38-C(21  1_555  1_555  1_555  1_555      #   76 Check
```

Author Response: The structure is affected by some strain. Crystal imperfections can not be excluded either.

```
PLAT703_ALERT_1_C Torsion Calc    134.8(3), Rep    134.4(5), Dev..       1.33 Sigma
              C(26-C(3)-C(45-C(20  1_555  1_555  1_555  1_555      #   79 Check
```

Author Response: The structure is affected by some strain. Crystal imperfections can not be excluded either.

```
PLAT703_ALERT_1_C Torsion Calc    175.5(3), Rep    175.0(5), Dev..       1.67 Sigma
              C(45-C(3)-C(26-C(17  1_555  1_555  1_555  1_555      #   80 Check
```

Author Response: The structure is affected by some strain. Crystal imperfections can not be excluded either.

```
PLAT703_ALERT_1_C Torsion Calc     10.4(5), Rep     11.4(9), Dev..       2.00 Sigma
              C(45-C(3)-C(38-S(2)  1_555  1_555  1_555  1_555      #   83 Check
```

Author Response: The structure is affected by some strain. Crystal imperfections can not be excluded either.

```
PLAT703_ALERT_1_C Torsion Calc      1.0(5), Rep      0.4(7), Dev..       1.20 Sigma
            C(28-C(20-C(30-C(43  1_555  1_555  1_555  1_555      #   88 Check
```

Author Response: The structure is affected by some strain. Crystal imperfections can not be excluded either.

```
PLAT703_ALERT_1_C Torsion Calc    -58.3(4), Rep    -58.8(5), Dev..       1.25 Sigma
            C(28-C(20-C(45-N(2)  1_555  1_555  1_555  1_555      #   91 Check
```

Author Response: The structure is affected by some strain. Crystal imperfections can not be excluded either.

```
PLAT703_ALERT_1_C Torsion Calc     66.7(5), Rep     67.3(6), Dev..       1.20 Sigma
            C(28-C(20-C(45-C(3)  1_555  1_555  1_555  1_555      #   92 Check
```

Author Response: The structure is affected by some strain. Crystal imperfections can not be excluded either.

```
PLAT703_ALERT_1_C Torsion Calc    118.5(4), Rep    117.8(5), Dev..       1.75 Sigma
            C(30-C(20-C(45-N(2)  1_555  1_555  1_555  1_555      #   95 Check
```

Author Response: The structure is affected by some strain. Crystal imperfections can not be excluded either.

```
PLAT703_ALERT_1_C Torsion Calc   -116.6(4), Rep   -116.1(5), Dev..       1.25 Sigma
            C(30-C(20-C(45-C(3)  1_555  1_555  1_555  1_555      #   96 Check
```

Author Response: The structure is affected by some strain. Crystal imperfections can not be excluded either.

```
PLAT703_ALERT_1_C Torsion Calc   -175.9(4), Rep   -176.4(5), Dev..       1.25 Sigma
            C(45-C(20-C(30-C(43  1_555  1_555  1_555  1_555      #   97 Check
```

Author Response: The structure is affected by some strain. Crystal imperfections can not be excluded either.

```
PLAT703_ALERT_1_C Torsion Calc      1.7(5), Rep      2.5(9), Dev..       1.60 Sigma
            C(29-C(21-C(38-C(3)  1_555  1_555  1_555  1_555      #   99 Check
```

Author Response: The structure is affected by some strain. Crystal imperfections can not be excluded either.

```
PLAT703_ALERT_1_C Torsion Calc    179.2(3), Rep    178.8(4), Dev..       1.33 Sigma
              C(38-C(21-C(29-CL(1  1_555  1_555  1_555  1_555      #  100 Check
```

Author Response: The structure is affected by some strain. Crystal imperfections can not be excluded either.

```
PLAT703_ALERT_1_C Torsion Calc      0.0(6), Rep     -0.7(8), Dev..       1.17 Sigma
              C(33-C(22-C(24-N(1)  1_555  1_555  1_555  1_555      #  104 Check
```

Author Response: The structure is affected by some strain. Crystal imperfections can not be excluded either.

```
PLAT703_ALERT_1_C Torsion Calc      1.6(6), Rep      0.6(8), Dev..       1.67 Sigma
              C(24-C(22-C(36-C(41  1_555  1_555  1_555  1_555      #  106 Check
```

Author Response: The structure is affected by some strain. Crystal imperfections can not be excluded either.

```
PLAT703_ALERT_1_C Torsion Calc    121.5(4), Rep    121.0(5), Dev..       1.25 Sigma
              C(36-C(22-C(33-N(3)  1_555  1_555  1_555  1_555      #  110 Check
```

Author Response: The structure is affected by some strain. Crystal imperfections can not be excluded either.

```
PLAT703_ALERT_1_C Torsion Calc    174.8(3), Rep    174.4(4), Dev..       1.33 Sigma
              S(1)-C(25-C(27-C(40  1_555  1_555  1_555  1_555      #  112 Check
```

Author Response: The structure is affected by some strain. Crystal imperfections can not be excluded either.

```
PLAT703_ALERT_1_C Torsion Calc     -0.3(6), Rep     -1.3(7), Dev..       1.67 Sigma
              C(1)-C(25-C(27-C(40  1_555  1_555  1_555  1_555      #  113 Check
```

Author Response: The structure is affected by some strain. Crystal imperfections can not be excluded either.

```
PLAT703_ALERT_1_C Torsion Calc   -179.0(3), Rep   -178.5(4), Dev..       1.67 Sigma
              C(25-C(27-C(40-CL(3  1_555  1_555  1_555  1_555      #  114 Check
```

Author Response: The structure is affected by some strain. Crystal imperfections can not be excluded either.

```
PLAT703_ALERT_1_C Torsion Calc      1.9(6), Rep      3.0(9), Dev..       1.83 Sigma
              C(20-C(30-C(43-C(42  1_555  1_555  1_555  1_555      #  119 Check
```

Author Response: The structure is affected by some strain. Crystal imperfections can not be excluded either.

```
PLAT703_ALERT_1_C Torsion Calc   -179.7(4), Rep    179.5(5), Dev..      2.00 Sigma
              O(8)-C(32-C(41-C(36  1_555  1_555  1_555  1_555      #  121 Check
```

Author Response: The structure is affected by some strain. Crystal imperfections can not be excluded either.

```
PLAT703_ALERT_1_C Torsion Calc    178.0(4), Rep    178.8(5), Dev..      2.00 Sigma
              C(2)-C(32-C(41-O(9)  1_555  1_555  1_555  1_555      #  122 Check
```

Author Response: The structure is affected by some strain. Crystal imperfections can not be excluded either.

```
PLAT703_ALERT_1_C Torsion Calc     -0.5(6), Rep      0.4(8), Dev..      1.50 Sigma
              C(22-C(36-C(41-C(32  1_555  1_555  1_555  1_555      #  127 Check
```

Author Response: The structure is affected by some strain. Crystal imperfections can not be excluded either.

```
PLAT703_ALERT_1_C Torsion Calc    178.7(4), Rep    178.2(5), Dev..      1.25 Sigma
              O(1)-C(42-C(43-C(30  1_555  1_555  1_555  1_555      #  134 Check
```

Author Response: The structure is affected by some strain. Crystal imperfections can not be excluded either.

```
PLAT703_ALERT_1_C Torsion Calc     -4.0(7), Rep     -5.1(9), Dev..      1.57 Sigma
              C(37-C(42-C(43-C(30  1_555  1_555  1_555  1_555      #  136 Check
```

Author Response: The structure is affected by some strain. Crystal imperfections can not be excluded either.

```
PLAT703_ALERT_1_C Torsion Calc      6.6(4), Rep      5.9(6), Dev..      1.75 Sigma
              C(35-C(50-C(53-C(52  1_555  1_555  1_555  1_555      #  140 Check
```

Author Response: The structure is affected by some strain. Crystal imperfections can not be excluded either.

```
PLAT703_ALERT_1_C Torsion Calc    -30.0(4), Rep    -29.5(6), Dev..      1.25 Sigma
              N(3)-C(52-C(53-C(50  1_555  1_555  1_555  1_555      #  141 Check
```

Author Response: The structure is affected by some strain. Crystal imperfections can not be excluded either.

```
PLAT751_ALERT_4_C Bond    Calc     0.95000, Rep    0.950(5) ......  Senseless s.u.
              C(2)     -H(1)           1_555   1_555 ........     #   34 Check
PLAT751_ALERT_4_C Bond    Calc     0.95000, Rep    0.950(6) ......  Senseless s.u.
              C(17)    -H(2)           1_555   1_555 ........     #   40 Check
PLAT751_ALERT_4_C Bond    Calc     0.95000, Rep    0.950(5) ......  Senseless s.u.
              C(21)    -H(3)           1_555   1_555 ........     #   46 Check
PLAT751_ALERT_4_C Bond    Calc     0.95000, Rep    0.950(4) ......  Senseless s.u.
              C(26)    -H(4)           1_555   1_555 ........     #   51 Check
PLAT751_ALERT_4_C Bond    Calc     0.95000, Rep    0.950(5) ......  Senseless s.u.
              C(27)    -H(5)           1_555   1_555 ........     #   53 Check
PLAT751_ALERT_4_C Bond    Calc     0.95000, Rep    0.950(5) ......  Senseless s.u.
              C(36)    -H(6)           1_555   1_555 ........     #   69 Check
PLAT751_ALERT_4_C Bond    Calc     0.95000, Rep    0.950(5) ......  Senseless s.u.
              C(37)    -H(7)           1_555   1_555 ........     #   71 Check
PLAT751_ALERT_4_C Bond    Calc     0.95000, Rep    0.950(6) ......  Senseless s.u.
              C(47)    -H(8)           1_555   1_555 ........     #   84 Check
PLAT751_ALERT_4_C Bond    Calc     0.95000, Rep    0.950(6) ......  Senseless s.u.
              C(48)    -H(9)           1_555   1_555 ........     #   85 Check
PLAT752_ALERT_4_C Angle   Calc      119.00, Rep    119.1(4) ......  Senseless s.u.
              C(24)   -C(2)    -H(1)       1_555  1_555  1_555     #   33 Check
PLAT752_ALERT_4_C Angle   Calc      120.00, Rep    119.5(4) ......  Senseless s.u.
              C(32)   -C(2)    -H(1)       1_555  1_555  1_555     #   34 Check
PLAT752_ALERT_4_C Angle   Calc      121.00, Rep    121.2(4) ......  Senseless s.u.
              C(26)   -C(17)   -H(2)       1_555  1_555  1_555     #   39 Check
PLAT752_ALERT_4_C Angle   Calc      120.00, Rep    120.1(4) ......  Senseless s.u.
              C(29)   -C(17)   -H(2)       1_555  1_555  1_555     #   40 Check
PLAT752_ALERT_4_C Angle   Calc      121.00, Rep    120.5(4) ......  Senseless s.u.
              C(29)   -C(21)   -H(3)       1_555  1_555  1_555     #   45 Check
PLAT752_ALERT_4_C Angle   Calc      120.00, Rep    119.9(4) ......  Senseless s.u.
              C(38)   -C(21)   -H(3)       1_555  1_555  1_555     #   46 Check
PLAT752_ALERT_4_C Angle   Calc      118.00, Rep    117.6(4) ......  Senseless s.u.
              C(3)    -C(26)   -H(4)       1_555  1_555  1_555     #   57 Check
PLAT752_ALERT_4_C Angle   Calc      120.00, Rep    119.8(4) ......  Senseless s.u.
              C(17)   -C(26)   -H(4)       1_555  1_555  1_555     #   58 Check
PLAT752_ALERT_4_C Angle   Calc      119.00, Rep    119.1(4) ......  Senseless s.u.
              C(25)   -C(27)   -H(5)       1_555  1_555  1_555     #   60 Check
PLAT752_ALERT_4_C Angle   Calc      119.00, Rep    118.9(5) ......  Senseless s.u.
              C(40)   -C(27)   -H(5)       1_555  1_555  1_555     #   61 Check
PLAT752_ALERT_4_C Angle   Calc      119.00, Rep    119.3(4) ......  Senseless s.u.
              C(22)   -C(36)   -H(6)       1_555  1_555  1_555     #   99 Check
PLAT752_ALERT_4_C Angle   Calc      119.00, Rep    119.5(5) ......  Senseless s.u.
              C(41)   -C(36)   -H(6)       1_555  1_555  1_555     #  100 Check
PLAT752_ALERT_4_C Angle   Calc      120.00, Rep    120.0(5) ......  Senseless s.u.
              C(28)   -C(37)   -H(7)       1_555  1_555  1_555     #  102 Check
PLAT752_ALERT_4_C Angle   Calc      120.00, Rep    120.2(6) ......  Senseless s.u.
              C(42)   -C(37)   -H(7)       1_555  1_555  1_555     #  103 Check
PLAT752_ALERT_4_C Angle   Calc      117.00, Rep    117.2(5) ......  Senseless s.u.
              C(1)    -C(47)   -H(8)       1_555  1_555  1_555     #  144 Check
PLAT752_ALERT_4_C Angle   Calc      119.00, Rep    118.9(5) ......  Senseless s.u.
              C(48)   -C(47)   -H(8)       1_555  1_555  1_555     #  145 Check
PLAT752_ALERT_4_C Angle   Calc      120.00, Rep    119.9(5) ......  Senseless s.u.
              C(40)   -C(48)   -H(9)       1_555  1_555  1_555     #  147 Check
PLAT752_ALERT_4_C Angle   Calc      121.00, Rep    121.4(6) ......  Senseless s.u.
              C(47)   -C(48)   -H(9)       1_555  1_555  1_555     #  148 Check
PLAT754_ALERT_4_C Contact Calc     3.15000, Rep    3.150(3) ......  Senseless s.u.
              CL(3)    -H(3)           1_555   1_556 ........     #  106 Check
PLAT754_ALERT_4_C Contact Calc     3.31000, Rep    3.306(3) ......  Senseless s.u.
```

```
              CL(3)    -H(7)          1_555   3_657 ........     #  106 Check
PLAT754_ALERT_4_C Contact Calc     2.92000, Rep    2.922(5) ......  Senseless s.u.
              O(1)     -H(9)          1_555   1_555 ........     #  106 Check
PLAT754_ALERT_4_C Contact Calc     2.89000, Rep    2.887(5) ......  Senseless s.u.
              O(1)     -H(9)          1_555   3_657 ........     #  106 Check
PLAT754_ALERT_4_C Contact Calc     3.15000, Rep    3.147(4) ......  Senseless s.u.
              O(5)     -H(6)          1_555   1_555 ........     #  106 Check
PLAT754_ALERT_4_C Contact Calc     2.68000, Rep    2.683(5) ......  Senseless s.u.
              O(5)     -H(8)          1_555   1_555 ........     #  106 Check
PLAT754_ALERT_4_C Contact Calc     3.15000, Rep    3.154(4) ......  Senseless s.u.
              O(6)     -H(1)          1_555   4_555 ........     #  106 Check
PLAT754_ALERT_4_C Contact Calc     2.77000, Rep    2.772(4) ......  Senseless s.u.
              O(7)     -H(7)          1_555   3_656 ........     #  106 Check
PLAT754_ALERT_4_C Contact Calc     2.67000, Rep    2.672(6) ......  Senseless s.u.
              O(8)     -H(2)          1_555   1_455 ........     #  106 Check
PLAT754_ALERT_4_C Contact Calc     3.49000, Rep    3.492(5) ......  Senseless s.u.
              O(8)     -H(4)          1_555   1_455 ........     #  106 Check
PLAT754_ALERT_4_C Contact Calc     2.82000, Rep    2.822(5) ......  Senseless s.u.
              O(9)     -H(5)          1_555   1_554 ........     #  106 Check
PLAT754_ALERT_4_C Contact Calc     2.85000, Rep    2.850(4) ......  Senseless s.u.
              O(10)    -H(4)          1_555   1_455 ........     #  106 Check
PLAT754_ALERT_4_C Contact Calc     2.99000, Rep    2.988(5) ......  Senseless s.u.
              C(2)     -H(4)          1_555   1_455 ........     #  106 Check
PLAT754_ALERT_4_C Contact Calc     3.20000, Rep    3.201(6) ......  Senseless s.u.
              C(21)    -H(6)          1_555   1_555 ........     #  106 Check
PLAT754_ALERT_4_C Contact Calc     3.52000, Rep    3.521(5) ......  Senseless s.u.
              C(24)    -H(4)          1_555   1_455 ........     #  106 Check
PLAT754_ALERT_4_C Contact Calc     3.33000, Rep    3.330(6) ......  Senseless s.u.
              C(28)    -H(8)          1_555   1_555 ........     #  106 Check
PLAT754_ALERT_4_C Contact Calc     3.36000, Rep    3.357(6) ......  Senseless s.u.
              C(32)    -H(2)          1_555   1_455 ........     #  106 Check
PLAT754_ALERT_4_C Contact Calc     3.19000, Rep    3.190(6) ......  Senseless s.u.
              C(32)    -H(4)          1_555   1_455 ........     #  106 Check
PLAT754_ALERT_4_C Contact Calc     3.19000, Rep    3.186(5) ......  Senseless s.u.
              C(37)    -H(8)          1_555   1_555 ........     #  106 Check
PLAT754_ALERT_4_C Contact Calc     3.54000, Rep    3.544(6) ......  Senseless s.u.
              C(37)    -H(9)          1_555   1_555 ........     #  106 Check
PLAT754_ALERT_4_C Contact Calc     3.58000, Rep    3.578(5) ......  Senseless s.u.
              C(39)    -H(6)          1_555   1_655 ........     #  106 Check
PLAT754_ALERT_4_C Contact Calc     3.40000, Rep    3.405(5) ......  Senseless s.u.
              C(40)    -H(3)          1_555   1_556 ........     #  106 Check
PLAT754_ALERT_4_C Contact Calc     3.31000, Rep    3.309(6) ......  Senseless s.u.
              C(42)    -H(8)          1_555   1_555 ........     #  106 Check
PLAT754_ALERT_4_C Contact Calc     3.19000, Rep    3.193(6) ......  Senseless s.u.
              C(42)    -H(9)          1_555   1_555 ........     #  106 Check
PLAT754_ALERT_4_C Contact Calc     3.51000, Rep    3.508(5) ......  Senseless s.u.
              C(43)    -H(8)          1_555   1_555 ........     #  106 Check
PLAT754_ALERT_4_C Contact Calc     3.43000, Rep    3.430(5) ......  Senseless s.u.
              C(44)    -H(6)          1_555   1_655 ........     #  106 Check
PLAT754_ALERT_4_C Contact Calc     3.46000, Rep    3.466(5) ......  Senseless s.u.
              C(50)    -H(2)          1_555   4_555 ........     #  106 Check
PLAT754_ALERT_4_C Contact Calc     3.04000, Rep    3.042(8) ......  Senseless s.u.
              C(54)    -H(5)          1_555   1_554 ........     #  106 Check
PLAT754_ALERT_4_C Contact Calc     3.40000, Rep    3.397(8) ......  Senseless s.u.
              C(55)    -H(1)          1_555   4_554 ........     #  106 Check
PLAT754_ALERT_4_C Contact Calc     3.34000, Rep    3.336(8) ......  Senseless s.u.
              C(55)    -H(2)          1_555   1_455 ........     #  106 Check
```

```
PLAT754_ALERT_4_C Contact Calc     3.52000, Rep    3.515(8) ......  Senseless s.u.
              C(56)    -H(7)            1_555   3_657 ........     #  106 Check
PLAT754_ALERT_4_C Contact Calc     3.45000, Rep    3.452(7) ......  Senseless s.u.
              C(56)    -H(9)            1_555   1_555 ........     #  106 Check
PLAT754_ALERT_4_C Contact Calc     3.15000, Rep    3.154(4) ......  Senseless s.u.
              H(1)     -O(6)            1_555   4_554 ........     #  106 Check
PLAT754_ALERT_4_C Contact Calc     3.40000, Rep    3.397(8) ......  Senseless s.u.
              H(1)     -C(55)           1_555   4_555 ........     #  106 Check
PLAT754_ALERT_4_C Contact Calc     3.44000, Rep    3.436(5) ......  Senseless s.u.
              H(1)     -H(2)            1_555   1_455 ........     #  106 Check
PLAT754_ALERT_4_C Contact Calc     3.04000, Rep    3.038(5) ......  Senseless s.u.
              H(1)     -H(4)            1_555   1_455 ........     #  106 Check
PLAT754_ALERT_4_C Contact Calc     2.67000, Rep    2.672(6) ......  Senseless s.u.
              H(2)     -O(8)            1_555   1_655 ........     #  106 Check
PLAT754_ALERT_4_C Contact Calc     3.36000, Rep    3.357(6) ......  Senseless s.u.
              H(2)     -C(32)           1_555   1_655 ........     #  106 Check
PLAT754_ALERT_4_C Contact Calc     3.46000, Rep    3.466(5) ......  Senseless s.u.
              H(2)     -C(50)           1_555   4_554 ........     #  106 Check
PLAT754_ALERT_4_C Contact Calc     3.34000, Rep    3.336(8) ......  Senseless s.u.
              H(2)     -C(55)           1_555   1_655 ........     #  106 Check
PLAT754_ALERT_4_C Contact Calc     3.44000, Rep    3.436(5) ......  Senseless s.u.
              H(2)     -H(1)            1_555   1_655 ........     #  106 Check
PLAT754_ALERT_4_C Contact Calc     3.15000, Rep    3.150(3) ......  Senseless s.u.
              H(3)     -CL(3)           1_555   1_554 ........     #  106 Check
PLAT754_ALERT_4_C Contact Calc     3.40000, Rep    3.405(5) ......  Senseless s.u.
              H(3)     -C(40)           1_555   1_554 ........     #  106 Check
PLAT754_ALERT_4_C Contact Calc     2.93000, Rep    2.932(6) ......  Senseless s.u.
              H(3)     -H(6)            1_555   1_555 ........     #  106 Check
PLAT754_ALERT_4_C Contact Calc     3.49000, Rep    3.492(5) ......  Senseless s.u.
              H(4)     -O(8)            1_555   1_655 ........     #  106 Check
PLAT754_ALERT_4_C Contact Calc     2.85000, Rep    2.850(4) ......  Senseless s.u.
              H(4)     -O(10)           1_555   1_655 ........     #  106 Check
PLAT754_ALERT_4_C Contact Calc     2.99000, Rep    2.988(5) ......  Senseless s.u.
              H(4)     -C(2)            1_555   1_655 ........     #  106 Check
PLAT754_ALERT_4_C Contact Calc     3.52000, Rep    3.521(5) ......  Senseless s.u.
              H(4)     -C(24)           1_555   1_655 ........     #  106 Check
PLAT754_ALERT_4_C Contact Calc     3.19000, Rep    3.190(6) ......  Senseless s.u.
              H(4)     -C(32)           1_555   1_655 ........     #  106 Check
PLAT754_ALERT_4_C Contact Calc     3.04000, Rep    3.038(5) ......  Senseless s.u.
              H(4)     -H(1)            1_555   1_655 ........     #  106 Check
PLAT754_ALERT_4_C Contact Calc     2.82000, Rep    2.822(5) ......  Senseless s.u.
              H(5)     -O(9)            1_555   1_556 ........     #  106 Check
PLAT754_ALERT_4_C Contact Calc     3.04000, Rep    3.042(8) ......  Senseless s.u.
              H(5)     -C(54)           1_555   1_556 ........     #  106 Check
PLAT754_ALERT_4_C Contact Calc     3.15000, Rep    3.147(4) ......  Senseless s.u.
              H(6)     -O(5)            1_555   1_555 ........     #  106 Check
PLAT754_ALERT_4_C Contact Calc     3.20000, Rep    3.201(6) ......  Senseless s.u.
              H(6)     -C(21)           1_555   1_555 ........     #  106 Check
PLAT754_ALERT_4_C Contact Calc     3.58000, Rep    3.578(5) ......  Senseless s.u.
              H(6)     -C(39)           1_555   1_455 ........     #  106 Check
PLAT754_ALERT_4_C Contact Calc     3.43000, Rep    3.430(5) ......  Senseless s.u.
              H(6)     -C(44)           1_555   1_455 ........     #  106 Check
PLAT754_ALERT_4_C Contact Calc     2.93000, Rep    2.932(6) ......  Senseless s.u.
              H(6)     -H(3)            1_555   1_555 ........     #  106 Check
PLAT754_ALERT_4_C Contact Calc     3.31000, Rep    3.306(3) ......  Senseless s.u.
              H(7)     -CL(3)           1_555   3_657 ........     #  106 Check
PLAT754_ALERT_4_C Contact Calc     2.77000, Rep    2.772(4) ......  Senseless s.u.
```

```
              H(7)     -O(7)            1_555   3_656 ........     #  106 Check
PLAT754_ALERT_4_C Contact Calc     3.52000, Rep    3.515(8) ......  Senseless s.u.
              H(7)     -C(56)           1_555   3_657 ........     #  106 Check
PLAT754_ALERT_4_C Contact Calc     3.54000, Rep    3.536(6) ......  Senseless s.u.
              H(7)     -H(8)            1_555   1_555 ........     #  106 Check
PLAT754_ALERT_4_C Contact Calc     3.56000, Rep    3.562(6) ......  Senseless s.u.
              H(7)     -H(9)            1_555   1_555 ........     #  106 Check
PLAT754_ALERT_4_C Contact Calc     3.58000, Rep    3.584(6) ......  Senseless s.u.
              H(7)     -H(9)            1_555   3_657 ........     #  106 Check
PLAT754_ALERT_4_C Contact Calc     2.68000, Rep    2.683(5) ......  Senseless s.u.
              H(8)     -O(5)            1_555   1_555 ........     #  106 Check
PLAT754_ALERT_4_C Contact Calc     3.33000, Rep    3.330(6) ......  Senseless s.u.
              H(8)     -C(28)           1_555   1_555 ........     #  106 Check
PLAT754_ALERT_4_C Contact Calc     3.19000, Rep    3.186(5) ......  Senseless s.u.
              H(8)     -C(37)           1_555   1_555 ........     #  106 Check
PLAT754_ALERT_4_C Contact Calc     3.31000, Rep    3.309(6) ......  Senseless s.u.
              H(8)     -C(42)           1_555   1_555 ........     #  106 Check
PLAT754_ALERT_4_C Contact Calc     3.51000, Rep    3.508(5) ......  Senseless s.u.
              H(8)     -C(43)           1_555   1_555 ........     #  106 Check
PLAT754_ALERT_4_C Contact Calc     3.54000, Rep    3.536(6) ......  Senseless s.u.
              H(8)     -H(7)            1_555   1_555 ........     #  106 Check
PLAT754_ALERT_4_C Contact Calc     2.92000, Rep    2.922(5) ......  Senseless s.u.
              H(9)     -O(1)            1_555   1_555 ........     #  106 Check
PLAT754_ALERT_4_C Contact Calc     2.89000, Rep    2.887(5) ......  Senseless s.u.
              H(9)     -O(1)            1_555   3_657 ........     #  106 Check
PLAT754_ALERT_4_C Contact Calc     3.54000, Rep    3.544(6) ......  Senseless s.u.
              H(9)     -C(37)           1_555   1_555 ........     #  106 Check
PLAT754_ALERT_4_C Contact Calc     3.19000, Rep    3.193(6) ......  Senseless s.u.
              H(9)     -C(42)           1_555   1_555 ........     #  106 Check
PLAT754_ALERT_4_C Contact Calc     3.45000, Rep    3.452(7) ......  Senseless s.u.
              H(9)     -C(56)           1_555   1_555 ........     #  106 Check
PLAT754_ALERT_4_C Contact Calc     3.56000, Rep    3.562(6) ......  Senseless s.u.
              H(9)     -H(7)            1_555   1_555 ........     #  106 Check
PLAT754_ALERT_4_C Contact Calc     3.58000, Rep    3.584(6) ......  Senseless s.u.
              H(9)     -H(7)            1_555   3_657 ........     #  106 Check
```

Alert level G

```
PLAT005_ALERT_5_G No Embedded Refinement Details Found  in the CIF     Please Do !
PLAT199_ALERT_1_G Reported _cell_measurement_temperature ..... (K)        293 Check
PLAT200_ALERT_1_G Reported   _diffrn_ambient_temperature ..... (K)        293 Check
PLAT343_ALERT_2_G Unusual sp3      Angle Range in Main Residue for        C49 Check
PLAT793_ALERT_4_G Model has Chirality at C33         (Centro SpGr)          R Verify
PLAT793_ALERT_4_G Model has Chirality at C45         (Centro SpGr)          R Verify
PLAT808_ALERT_5_G No Parseable SHELXL Style Weighting Scheme Found     Please Check
PLAT882_ALERT_1_G No Datum for _diffrn_reflns_av_unetI/netI ......     Please Do !
PLAT883_ALERT_1_G Absent Datum for _atom_sites_solution_primary ..     Please Do !
```

---

4 **ALERT level A** = Most likely a serious problem - resolve or explain
21 **ALERT level B** = A potentially serious problem, consider carefully
185 **ALERT level C** = Check. Ensure it is not caused by an omission or oversight
9 **ALERT level G** = General information/check it is not something unexpected

61 ALERT type 1 CIF construction/syntax error, inconsistent or missing data

```
 45 ALERT type 2 Indicator that the structure model may be wrong or deficient
  3 ALERT type 3 Indicator that the structure quality may be low
108 ALERT type 4 Improvement, methodology, query or suggestion
  2 ALERT type 5 Informative message, check
```

It is advisable to attempt to resolve as many as possible of the alerts in all categories. Often the minor alerts point to easily fixed oversights, errors and omissions in your CIF or refinement strategy, so attention to these fine details can be worthwhile. In order to resolve some of the more serious problems it may be necessary to carry out additional measurements or structure refinements. However, the purpose of your study may justify the reported deviations and the more serious of these should normally be commented upon in the discussion or experimental section of a paper or in the "special_details" fields of the CIF. checkCIF was carefully designed to identify outliers and unusual parameters, but every test has its limitations and alerts that are not important in a particular case may appear. Conversely, the absence of alerts does not guarantee there are no aspects of the results needing attention. It is up to the individual to critically assess their own results and, if necessary, seek expert advice.

Publication of your CIF in IUCr journals

A basic structural check has been run on your CIF. These basic checks will be run on all CIFs submitted for publication in IUCr journals (*Acta Crystallographica*, *Journal of Applied Crystallography*, *Journal of Synchrotron Radiation*); however, if you intend to submit to *Acta Crystallographica Section C* or *E* or *IUCrData*, you should make sure that full publication checks are run on the final version of your CIF prior to submission.

Publication of your CIF in other journals

Please refer to the *Notes for Authors* of the relevant journal for any special instructions relating to CIF submission.

Validation response form

Please find below a validation response form (VRF) that can be filled in and pasted into your CIF.

```
# start Validation Reply Form
_vrf_REFLT02__143734_1
;
PROBLEM: The number of reflections greater than the sigma threshold
RESPONSE: ...
;
_vrf_DIFMN03__143734_1
;
PROBLEM: The minimum difference density is < -0.1*ZMAX*0.75
RESPONSE: ...
;
_vrf_DIFMX02__143734_1
;
PROBLEM: The maximum difference density is > 0.1*ZMAX*0.75
RESPONSE: ...
;
```

```
_vrf_PLAT230__143734_1
;
PROBLEM: Hirshfeld Test Diff for    Cl1      --C29      .        8.3 s.u.
RESPONSE: ...
;
_vrf_PLAT241__143734_1
;
PROBLEM: High   'MainMol' Ueq as Compared to Neighbors of        C49 Check
RESPONSE: ...
;
_vrf_PLAT360__143734_1
;
PROBLEM: Short  C(sp3)-C(sp3) Bond  C49      - C56      .       1.30 Ang.
RESPONSE: ...
;
_vrf_PLAT410__143734_1
;
PROBLEM: Short Intra H...H Contact  H23      ..H36      .       1.87 Ang.
RESPONSE: ...
;
_vrf_PLAT029__143734_1
;
PROBLEM: _diffrn_measured_fraction_theta_full value Low .      0.977 Why?
RESPONSE: ...
;
_vrf_PLAT213__143734_1
;
PROBLEM: Atom C49             has ADP max/min Ratio .....        3.9 prolat
RESPONSE: ...
;
_vrf_PLAT220__143734_1
;
PROBLEM: NonSolvent   Resd 1  C   Ueq(max)/Ueq(min) Range        5.4 Ratio
RESPONSE: ...
;
_vrf_PLAT222__143734_1
;
PROBLEM: NonSolvent Resd 1  H   Uiso(max)/Uiso(min) Range        4.6 Ratio
RESPONSE: ...
;
_vrf_PLAT234__143734_1
;
PROBLEM: Large Hirshfeld Difference O1       --C56      .       0.16 Ang.
RESPONSE: ...
;
_vrf_PLAT242__143734_1
;
PROBLEM: Low    'MainMol' Ueq as Compared to Neighbors of         O1 Check
RESPONSE: ...
;
_vrf_PLAT340__143734_1
;
PROBLEM: Low Bond Precision on  C-C Bonds ..............    0.00594 Ang.
RESPONSE: ...
;
_vrf_PLAT601__143734_1
;
```

```
PROBLEM: Unit Cell Contains Solvent Accessible VOIDS   <=         51 Ang**3
RESPONSE: ...
;
_vrf_PLAT751__143734_1
;
PROBLEM: Bond    Calc     0.95000, Rep    0.950(5) ......  Senseless s.u.
RESPONSE: ...
;
_vrf_PLAT752__143734_1
;
PROBLEM: Angle   Calc      119.00, Rep    119.1(4) ......  Senseless s.u.
RESPONSE: ...
;
_vrf_PLAT754__143734_1
;
PROBLEM: Contact Calc     3.15000, Rep    3.150(3) ......  Senseless s.u.
RESPONSE: ...
;
# end Validation Reply Form
```

PLATON version of 04/06/2025; check.def file version of 30/05/2025

Datablock _143734_1 - ellipsoid plot

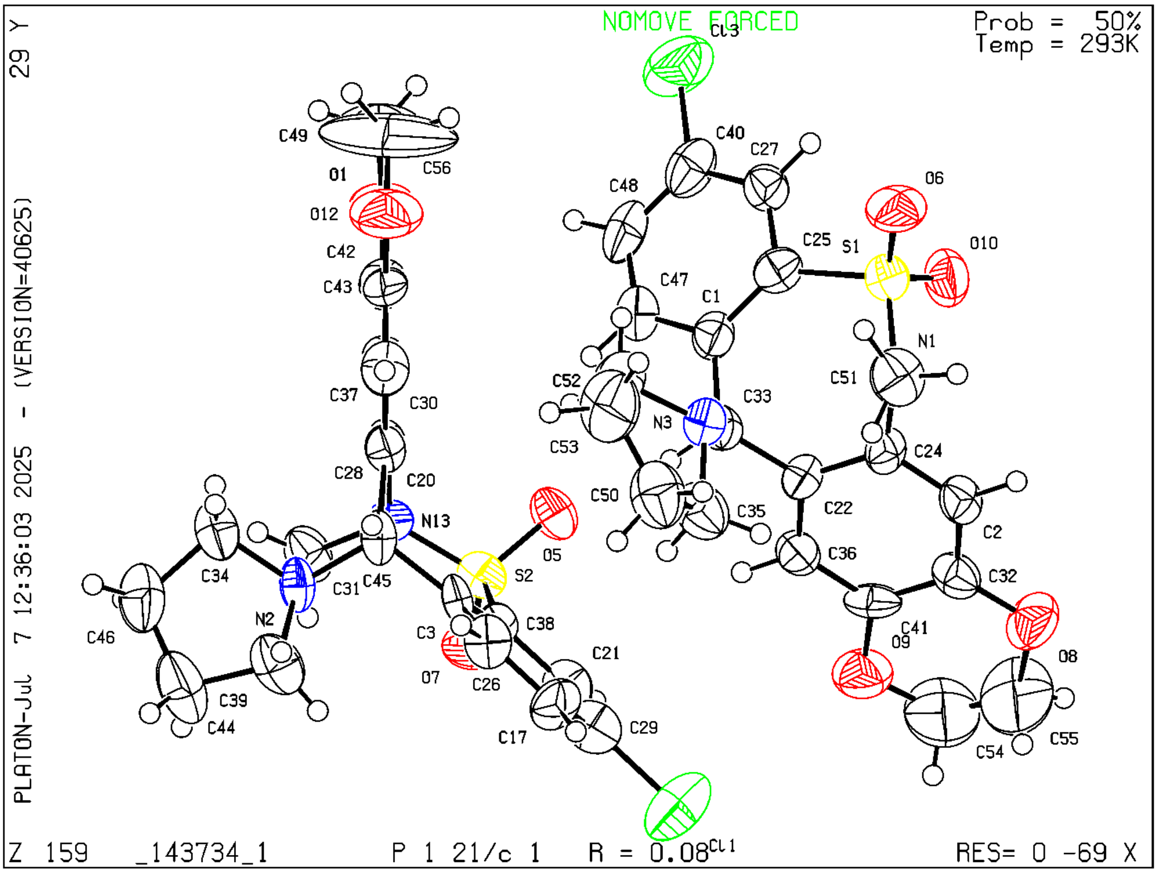

Supplement: File 2 — Crystallographic information files, checkcif and structure report files for compounds 20e, 21g, 23a, 25–27. [file compound 21g checkcif.pdf › compound 21g checkcif.pdf]
