# Supplementary material for: Synthesis of new tetra- and pentacyclic, methylenedioxy- and ethylenedioxy-substituted derivatives of the dibenzo[c,f][1,2]thiazepine ring system
Source: Beilstein J Org Chem. 2025 Dec 9;21:2645–56. doi: 10.3762/bjoc.21.205 (PMC12706374; doi:10.3762/bjoc.21.205)

checkCIF/PLATON report

You have not supplied any structure factors. As a result the full set of tests cannot be run.

THIS REPORT IS FOR GUIDANCE ONLY. IF USED AS PART OF A REVIEW PROCEDURE FOR PUBLICATION, IT SHOULD NOT REPLACE THE EXPERTISE OF AN EXPERIENCED CRYSTALLOGRAPHIC REFEREE.

No syntax errors found. CIF dictionary Interpreting this report

Datablock: _143758_1

```
Bond precision:   C-C = 0.0068 A          Wavelength=1.54187

Cell:              a=8.1686(14)      b=11.9644(16)      c=17.018(2)
                   alpha=90          beta=90            gamma=90
Temperature:       293 K

                Calculated                  Reported
Volume          1663.2(4)                   1663.2(4)
Space group     P 21 21 21                  P 21 21 21
Hall group      P 2ac 2ab                   P 2ac 2ab
Moiety formula  C17 H16 Cl N O5 S           C17 H16 Cl N O5 S
Sum formula     C17 H16 Cl N O5 S           C17 H16 Cl N O5 S
Mr              381.82                      381.83
Dx,g cm-3       1.525                       1.525
Z               4                           4
Mu (mm-1)       3.473                       3.474
F000            792.0                       792.0
F000'           797.06
h,k,lmax        10,14,20                    10,14,20
Nref            3252[ 1876]                 3162
Tmin,Tmax       0.316,0.707                 0.342,0.709
Tmin'           0.176

Correction method= # Reported T Limits: Tmin=0.342 Tmax=0.709
AbsCorr = MULTI-SCAN

Data completeness= 1.69/0.97       Theta(max)= 71.790

                                                  wR2(reflections)=
R(reflections)= 0.0826( 9804)                     0.1158( 9893)

S = 1.202                   Npar= 242
```

The following ALERTS were generated. Each ALERT has the format
test-name_ALERT_alert-type_alert-level.
Click on the hyperlinks for more details of the test.

Alert level A

```
DIFMN02_ALERT_2_A  The minimum difference density is > 0.0
```

Author Response: Altough the residual density is out of border but there is no doubt about the chemical structure taking into account other evidences, e.g. NMR. The error maybe due to crystal imperfections.

```
PLAT099_ALERT_1_A Minimum (Negative) Residual Density .GE. 0 !!...       1.83 eA-3
```

Author Response: Altough the residual density is out of border but there is no doubt about the chemical structure taking into account other evidences, e.g. NMR. The error maybe due to crystal imperfections.

Alert level B

```
REFLT02_ALERT_1_B  The number of reflections greater than the sigma threshold
            cannot exceed the number of symmetry-independent reflections
            Number of symmetry-independent reflections =   3162
            Number of reflections greater than sigma threshold =   9804
PLAT035_ALERT_1_B _chemical_absolute_configuration Info  Not Given     Please Do !
PLAT097_ALERT_2_B Large Reported Max.  (Positive) Residual Density       1.83 eA-3
PLAT230_ALERT_2_B Hirshfeld Test Diff for    S1        --O4        .     12.5 s.u.
PLAT703_ALERT_1_B Torsion Calc     83.0(3), Rep     82.1(6), Dev..       3.00 Sigma
              O(4)-S(1)-C(22-C(12  1_555  1_555  1_555  1_555      #     4 Check
PLAT703_ALERT_1_B Torsion Calc   -162.2(3), Rep   -163.0(5), Dev..       2.67 Sigma
              N(1)-S(1)-C(22-C(12  1_555  1_555  1_555  1_555      #    10 Check
PLAT703_ALERT_1_B Torsion Calc    -73.3(3), Rep    -74.0(6), Dev..       2.33 Sigma
              C(22-S(1)-N(1)-C(10  1_555  1_555  1_555  1_555      #    11 Check
PLAT703_ALERT_1_B Torsion Calc    172.0(4), Rep    173.0(7), Dev..       2.50 Sigma
              C(14-C(11-C(22-C(12  1_555  1_555  1_555  1_555      #    44 Check
PLAT703_ALERT_1_B Torsion Calc    179.4(4), Rep   -179.6(6), Dev..       2.50 Sigma
              C(22-C(12-C(18-CL(2  1_555  1_555  1_555  1_555      #    49 Check
```

Alert level C

```
DIFMX02_ALERT_1_C  The maximum difference density is > 0.1*ZMAX*0.75
            The relevant atom site should be identified.
RINTA01_ALERT_3_C  The value of Rint is greater than 0.12
            Rint given   0.171
PLAT020_ALERT_3_C The Value of Rint is Greater Than 0.12 .........      0.171 Report
PLAT034_ALERT_1_C No Flack Parameter Given. Z > Si, NonCentro ....     Please Do !
PLAT234_ALERT_4_C Large Hirshfeld Difference C20      --C25       .      0.16 Ang.
PLAT241_ALERT_2_C High   'MainMol' Ueq as Compared to Neighbors of        C25 Check
PLAT340_ALERT_3_C Low Bond Precision on  C-C Bonds ...............     0.0068 Ang.
PLAT360_ALERT_2_C Short  C(sp3)-C(sp3) Bond  C20       - C25       .      1.37 Ang.
PLAT703_ALERT_1_C Torsion Calc    -88.8(4), Rep    -88.3(7), Dev..       1.25 Sigma
```

```
              O(4)-S(1)-C(22-C(11  1_555  1_555  1_555  1_555      #    3 Check
PLAT703_ALERT_1_C Torsion Calc    169.6(3), Rep    169.1(5), Dev..      1.67 Sigma
              O(5)-S(1)-N(1)-C(10  1_555  1_555  1_555  1_555      #    5 Check
PLAT703_ALERT_1_C Torsion Calc    142.9(4), Rep    143.6(7), Dev..      1.75 Sigma
              O(5)-S(1)-C(22-C(11  1_555  1_555  1_555  1_555      #    7 Check
PLAT703_ALERT_1_C Torsion Calc    -45.4(4), Rep    -46.1(6), Dev..      1.75 Sigma
              O(5)-S(1)-C(22-C(12  1_555  1_555  1_555  1_555      #    8 Check
PLAT703_ALERT_1_C Torsion Calc     26.1(4), Rep     26.7(8), Dev..      1.50 Sigma
              N(1)-S(1)-C(22-C(11  1_555  1_555  1_555  1_555      #    9 Check
PLAT703_ALERT_1_C Torsion Calc   -170.3(3), Rep   -169.8(6), Dev..      1.67 Sigma
              C(23-O(3)-C(14-C(11  1_555  1_555  1_555  1_555      #   14 Check
PLAT703_ALERT_1_C Torsion Calc    -94.1(5), Rep    -94.7(8), Dev..      1.20 Sigma
              C(24-N(1)-C(10-C(1)  1_555  1_555  1_555  1_555      #   23 Check
PLAT703_ALERT_1_C Torsion Calc   -174.3(4), Rep   -174.9(7), Dev..      1.50 Sigma
              C(14-C(11-C(13-C(16  1_555  1_555  1_555  1_555      #   39 Check
PLAT703_ALERT_1_C Torsion Calc    169.7(3), Rep    169.3(6), Dev..      1.33 Sigma
              C(13-C(11-C(22-S(1)  1_555  1_555  1_555  1_555      #   40 Check
PLAT703_ALERT_1_C Torsion Calc     -1.5(6), Rep    -0.3(10), Dev..      2.00 Sigma
              C(13-C(11-C(22-C(12  1_555  1_555  1_555  1_555      #   41 Check
PLAT703_ALERT_1_C Torsion Calc     -0.2(7), Rep    -1.0(12), Dev..      1.14 Sigma
              C(22-C(11-C(13-C(16  1_555  1_555  1_555  1_555      #   42 Check
PLAT703_ALERT_1_C Torsion Calc      1.9(7), Rep     0.5(11), Dev..      2.00 Sigma
              C(18-C(12-C(22-C(11  1_555  1_555  1_555  1_555      #   48 Check
PLAT703_ALERT_1_C Torsion Calc     -0.6(8), Rep     0.7(12), Dev..      1.62 Sigma
              C(22-C(12-C(18-C(16  1_555  1_555  1_555  1_555      #   50 Check
PLAT703_ALERT_1_C Torsion Calc    178.9(4), Rep    178.3(7), Dev..      1.50 Sigma
              C(13-C(16-C(18-CL(2  1_555  1_555  1_555  1_555      #   54 Check
PLAT703_ALERT_1_C Torsion Calc     -1.1(8), Rep    -2.0(14), Dev..      1.12 Sigma
              C(13-C(16-C(18-C(12  1_555  1_555  1_555  1_555      #   55 Check
```

Alert level G

```
PLAT005_ALERT_5_G No Embedded Refinement Details Found  in the CIF     Please Do !
PLAT199_ALERT_1_G Reported _cell_measurement_temperature ..... (K)        293 Check
PLAT200_ALERT_1_G Reported   _diffrn_ambient_temperature ..... (K)        293 Check
PLAT791_ALERT_4_G Model has Chirality at C14        (Sohncke SpGr)          S Verify
PLAT808_ALERT_5_G No Parseable SHELXL Style Weighting Scheme Found     Please Check
PLAT882_ALERT_1_G No Datum for _diffrn_reflns_av_unetI/netI ......     Please Do !
PLAT883_ALERT_1_G Absent Datum for _atom_sites_solution_primary ..     Please Do !
```

---

2 **ALERT level A** = Most likely a serious problem - resolve or explain
9 **ALERT level B** = A potentially serious problem, consider carefully
23 **ALERT level C** = Check. Ensure it is not caused by an omission or oversight
7 **ALERT level G** = General information/check it is not something unexpected

29 ALERT type 1 CIF construction/syntax error, inconsistent or missing data
5 ALERT type 2 Indicator that the structure model may be wrong or deficient
3 ALERT type 3 Indicator that the structure quality may be low
2 ALERT type 4 Improvement, methodology, query or suggestion
2 ALERT type 5 Informative message, check

It is advisable to attempt to resolve as many as possible of the alerts in all categories. Often the minor alerts point to easily fixed oversights, errors and omissions in your CIF or refinement strategy, so attention to these fine details can be worthwhile. In order to resolve some of the more serious problems it may be necessary to carry out additional measurements or structure refinements. However, the purpose of your study may justify the reported deviations and the more serious of these should normally be commented upon in the discussion or experimental section of a paper or in the "special_details" fields of the CIF. checkCIF was carefully designed to identify outliers and unusual parameters, but every test has its limitations and alerts that are not important in a particular case may appear. Conversely, the absence of alerts does not guarantee there are no aspects of the results needing attention. It is up to the individual to critically assess their own results and, if necessary, seek expert advice.

Publication of your CIF in IUCr journals

A basic structural check has been run on your CIF. These basic checks will be run on all CIFs submitted for publication in IUCr journals (*Acta Crystallographica*, *Journal of Applied Crystallography*, *Journal of Synchrotron Radiation*); however, if you intend to submit to *Acta Crystallographica Section C* or *E* or *IUCrData*, you should make sure that full publication checks are run on the final version of your CIF prior to submission.

Publication of your CIF in other journals

Please refer to the *Notes for Authors* of the relevant journal for any special instructions relating to CIF submission.

Validation response form

Please find below a validation response form (VRF) that can be filled in and pasted into your CIF.

```
# start Validation Reply Form
_vrf_REFLT02__143758_1
;
PROBLEM: The number of reflections greater than the sigma threshold
RESPONSE: ...
;
_vrf_DIFMX02__143758_1
;
PROBLEM: The maximum difference density is > 0.1*ZMAX*0.75
RESPONSE: ...
;
_vrf_RINTA01__143758_1
;
PROBLEM: The value of Rint is greater than 0.12
RESPONSE: ...
;
_vrf_PLAT035__143758_1
;
PROBLEM: _chemical_absolute_configuration Info  Not Given     Please Do !
RESPONSE: ...
;
_vrf_PLAT097__143758_1
```

```
;
PROBLEM: Large Reported Max.  (Positive) Residual Density       1.83 eA-3
RESPONSE: ...
;
_vrf_PLAT230__143758_1
;
PROBLEM: Hirshfeld Test Diff for    S1       --O4       .       12.5 s.u.
RESPONSE: ...
;
_vrf_PLAT703__143758_1
;
PROBLEM: Torsion Calc     83.0(3), Rep     82.1(6), Dev..       3.00 Sigma
RESPONSE: ...
;
_vrf_PLAT020__143758_1
;
PROBLEM: The Value of Rint is Greater Than 0.12 .........      0.171 Report
RESPONSE: ...
;
_vrf_PLAT034__143758_1
;
PROBLEM: No Flack Parameter Given. Z > Si, NonCentro ....     Please Do !
RESPONSE: ...
;
_vrf_PLAT234__143758_1
;
PROBLEM: Large Hirshfeld Difference C20      --C25      .       0.16 Ang.
RESPONSE: ...
;
_vrf_PLAT241__143758_1
;
PROBLEM: High   'MainMol' Ueq as Compared to Neighbors of        C25 Check
RESPONSE: ...
;
_vrf_PLAT340__143758_1
;
PROBLEM: Low Bond Precision on  C-C Bonds ..............     0.0068 Ang.
RESPONSE: ...
;
_vrf_PLAT360__143758_1
;
PROBLEM: Short  C(sp3)-C(sp3) Bond  C20      - C25      .       1.37 Ang.
RESPONSE: ...
;
# end Validation Reply Form
```

PLATON version of 04/06/2025; check.def file version of 30/05/2025

Datablock _143758_1 - ellipsoid plot

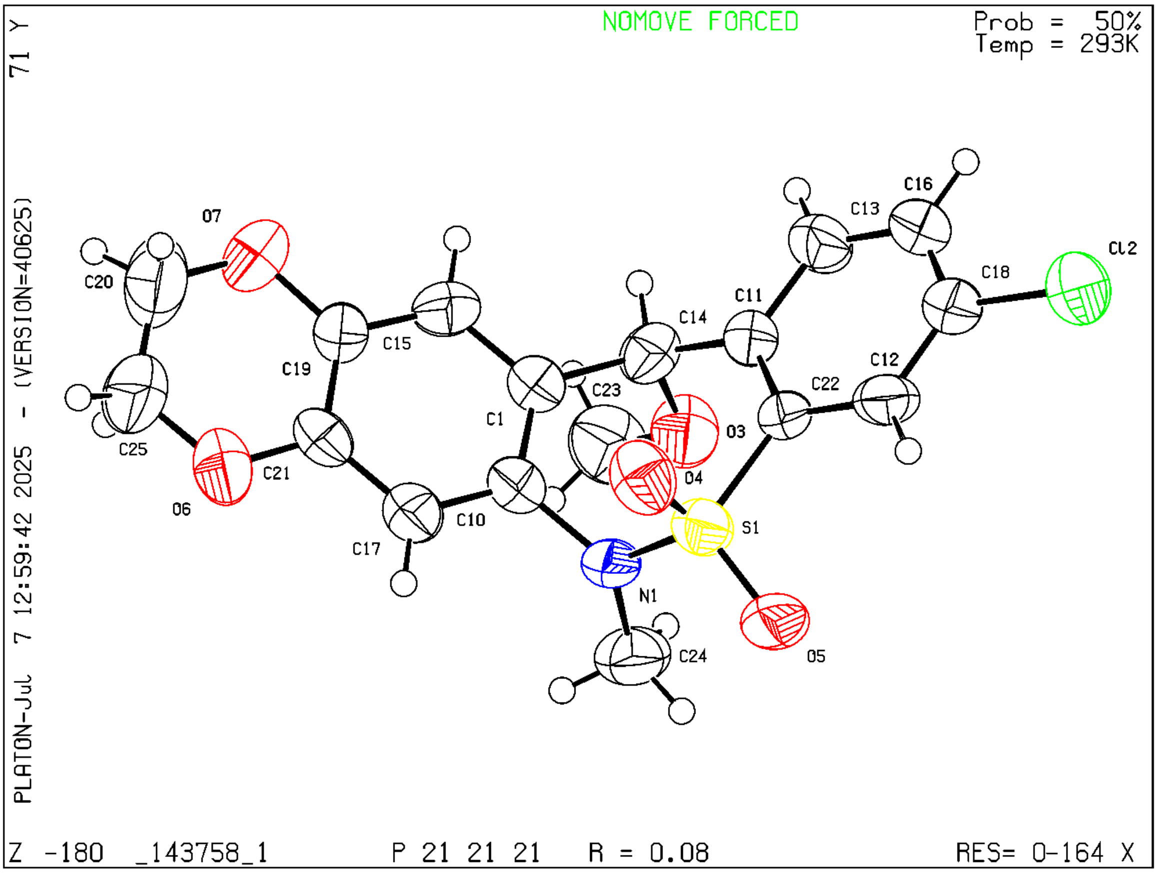

Supplement: File 2 — Crystallographic information files, checkcif and structure report files for compounds 20e, 21g, 23a, 25–27. [file compound 23a checkcif.pdf › compound 23a checkcif.pdf]
