# Supplementary material for: Synthesis of new tetra- and pentacyclic, methylenedioxy- and ethylenedioxy-substituted derivatives of the dibenzo[c,f][1,2]thiazepine ring system
Source: Beilstein J Org Chem. 2025 Dec 9;21:2645–56. doi: 10.3762/bjoc.21.205 (PMC12706374; doi:10.3762/bjoc.21.205)

checkCIF/PLATON report

You have not supplied any structure factors. As a result the full set of tests cannot be run.

THIS REPORT IS FOR GUIDANCE ONLY. IF USED AS PART OF A REVIEW PROCEDURE FOR PUBLICATION, IT SHOULD NOT REPLACE THE EXPERTISE OF AN EXPERIENCED CRYSTALLOGRAPHIC REFEREE.

No syntax errors found. CIF dictionary Interpreting this report

Datablock: _85623_1

Bond precision: C-C = 0.0023 A Wavelength=0.71075

Cell:	a=26.820(2)	b=10.064(1)	c=16.4457(15)
	alpha=90	beta=127.6638(16)	gamma=90
Temperature:	293 K		

	Calculated	Reported
Volume	3513.9(5)	3514.0(6)
Space group	C 2/c	C 1 2/c 1
Hall group	-C 2yc	-C 2yc
Moiety formula	C18 H17 Cl N2 O4 S	C18 H17 Cl N2 O4 S
Sum formula	C18 H17 Cl N2 O4 S	C18 H17 Cl N2 O4 S
Mr	392.85	392.86
Dx,g cm-3	1.485	1.485
Z	8	8
Mu (mm-1)	0.364	0.363
F000	1632.0	1632.0
F000’	1634.87	
h,k,lmax	34,13,21	34,13,21
Nref	4027	4020
Tmin,Tmax	0.957,0.986	
Tmin’	0.874	

Correction method= Not given

Data completeness= 0.998 Theta(max)= 27.470

R(reflections)= 0.0579(26276) wR2(reflections)= 0.0857(26624)

S = 0.613 Npar= 297

The following ALERTS were generated. Each ALERT has the format
test-name_ALERT_alert-type_alert-level.
Click on the hyperlinks for more details of the test.

Alert level A

```
DIFMN02_ALERT_2_A  The minimum difference density is < -0.1*ZMAX*2.00
            _refine_diff_density_min given =    -20.600
            Test value =     -3.400
```

Author Response: Altough the residual density is out of border but there is no doubt about the chemical structure taking into account other evidences, e.g. NMR. The error maybe due to crystal imperfections.

```
PLAT097_ALERT_2_A Large Reported Max.  (Positive) Residual Density      15.90 eA-3
```

Author Response: Altough the residual density is out of border but there is no doubt about the chemical structure taking into account other evidences, e.g. NMR. The error maybe due to crystal imperfections.

```
PLAT098_ALERT_2_A Large Reported Min.  (Negative) Residual Density     -20.60 eA-3
```

Author Response: Altough the residual density is out of border but there is no doubt about the chemical structure taking into account other evidences, e.g. NMR. The error maybe due to crystal imperfections.

```
PLAT703_ALERT_1_A Torsion Calc   175.52(9), Rep  175.85(15), Dev..       3.67 Sigma
              O(3)-S(1)-N(1)-C(9)  1_555  1_555  1_555  1_555      #    1 Check
```

Author Response: The structure is affected by some strain. Crystal imperfections can not be excluded either.

```
PLAT703_ALERT_1_A Torsion Calc  -39.95(13), Rep    -40.6(2), Dev..       5.00 Sigma
              O(3)-S(1)-N(1)-C(15  1_555  1_555  1_555  1_555      #    2 Check
```

Author Response: The structure is affected by some strain. Crystal imperfections can not be excluded either.

```
PLAT703_ALERT_1_A Torsion Calc   45.67(12), Rep     46.1(2), Dev..       3.58 Sigma
              O(4)-S(1)-N(1)-C(9)  1_555  1_555  1_555  1_555      #    5 Check
```

Author Response: The structure is affected by some strain. Crystal imperfections can not be excluded either.

```
PLAT703_ALERT_1_A Torsion Calc -169.80(12), Rep   -170.4(2), Dev..       5.00 Sigma
           O(4)-S(1)-N(1)-C(15  1_555  1_555  1_555  1_555      #    6 Check
```

Author Response: The structure is affected by some strain. Crystal imperfections can not be excluded either.

```
PLAT703_ALERT_1_A Torsion Calc   76.06(14), Rep     75.2(2), Dev..       6.14 Sigma
           C(13-S(1)-N(1)-C(15  1_555  1_555  1_555  1_555      #   12 Check
```

Author Response: The structure is affected by some strain. Crystal imperfections can not be excluded either.

```
PLAT703_ALERT_1_A Torsion Calc  -16.79(19), Rep    -16.0(3), Dev..       4.16 Sigma
           C(26-O(1)-C(17-C(21  1_555  1_555  1_555  1_555      #   15 Check
```

Author Response: The structure is affected by some strain. Crystal imperfections can not be excluded either.

```
PLAT703_ALERT_1_A Torsion Calc     44.1(2), Rep     41.6(4), Dev..      12.50 Sigma
           C(21-O(5)-C(25-C(26  1_555  1_555  1_555  1_555      #   16 Check
```

Author Response: The structure is affected by some strain. Crystal imperfections can not be excluded either.

```
PLAT703_ALERT_1_A Torsion Calc  167.72(14), Rep    169.5(2), Dev..      12.71 Sigma
           C(25-O(5)-C(21-C(16  1_555  1_555  1_555  1_555      #   17 Check
```

Author Response: The structure is affected by some strain. Crystal imperfections can not be excluded either.

```
PLAT703_ALERT_1_A Torsion Calc    -13.5(2), Rep    -10.8(3), Dev..      13.50 Sigma
           C(25-O(5)-C(21-C(17  1_555  1_555  1_555  1_555      #   18 Check
```

Author Response: The structure is affected by some strain. Crystal imperfections can not be excluded either.

```
PLAT703_ALERT_1_A Torsion Calc   79.12(14), Rep     78.6(2), Dev..       3.71 Sigma
           S(1)-N(1)-C(9)-C(14  1_555  1_555  1_555  1_555      #   20 Check
```

Author Response: The structure is affected by some strain. Crystal imperfections can not be excluded either.

```
PLAT703_ALERT_1_A Torsion Calc    -65.2(2), Rep    -64.0(3), Dev..       6.00 Sigma
           S(1)-N(1)-C(15-C(19  1_555  1_555  1_555  1_555      #   21 Check
```

Author Response: The structure is affected by some strain. Crystal imperfections can not be excluded either.

```
PLAT703_ALERT_1_A Torsion Calc  114.41(15), Rep    115.3(2), Dev..       5.93 Sigma
             C(15-N(1)-C(9)-C(10  1_555  1_555  1_555  1_555      #   23 Check
```

Author Response: The structure is affected by some strain. Crystal imperfections can not be excluded either.

```
PLAT703_ALERT_1_A Torsion Calc   49.77(16), Rep     49.2(2), Dev..       3.56 Sigma
             C(19-N(8)-C(18-C(12  1_555  1_555  1_555  1_555      #   26 Check
```

Author Response: The structure is affected by some strain. Crystal imperfections can not be excluded either.

```
PLAT703_ALERT_1_A Torsion Calc  -77.85(18), Rep    -77.1(2), Dev..       4.17 Sigma
             C(23-N(8)-C(18-C(12  1_555  1_555  1_555  1_555      #   28 Check
```

Author Response: The structure is affected by some strain. Crystal imperfections can not be excluded either.

```
PLAT703_ALERT_1_A Torsion Calc  149.36(16), Rep    150.1(2), Dev..       4.63 Sigma
             C(23-N(8)-C(18-C(14  1_555  1_555  1_555  1_555      #   29 Check
```

Author Response: The structure is affected by some strain. Crystal imperfections can not be excluded either.

```
PLAT703_ALERT_1_A Torsion Calc  179.81(13), Rep    179.4(2), Dev..       3.15 Sigma
             C(10-C(9)-C(14-C(18  1_555  1_555  1_555  1_555      #   35 Check
```

Author Response: The structure is affected by some strain. Crystal imperfections can not be excluded either.

```
PLAT703_ALERT_1_A Torsion Calc      0.5(2), Rep      1.4(4), Dev..       4.50 Sigma
             C(14-C(9)-C(10-C(17  1_555  1_555  1_555  1_555      #   36 Check
```

Author Response: The structure is affected by some strain. Crystal imperfections can not be excluded either.

```
PLAT703_ALERT_1_A Torsion Calc      1.1(2), Rep      0.2(3), Dev..       4.50 Sigma
             C(9)-C(10-C(17-C(21  1_555  1_555  1_555  1_555      #   38 Check
```

Author Response: The structure is affected by some strain. Crystal imperfections can not be excluded either.

```
PLAT703_ALERT_1_A Torsion Calc     -0.3(3), Rep      1.2(4), Dev..       5.00 Sigma
              C(13-C(12-C(22-C(24  1_555  1_555  1_555  1_555      #   47 Check
```

Author Response: The structure is affected by some strain. Crystal imperfections can not be excluded either.

```
PLAT703_ALERT_1_A Torsion Calc  175.70(14), Rep    175.2(2), Dev..       3.57 Sigma
              C(22-C(12-C(13-S(1)  1_555  1_555  1_555  1_555      #   48 Check
```

Author Response: The structure is affected by some strain. Crystal imperfections can not be excluded either.

```
PLAT703_ALERT_1_A Torsion Calc -176.28(19), Rep   -175.6(3), Dev..       3.58 Sigma
              C(18-C(12-C(22-C(24  1_555  1_555  1_555  1_555      #   50 Check
```

Author Response: The structure is affected by some strain. Crystal imperfections can not be excluded either.

```
PLAT703_ALERT_1_A Torsion Calc  -57.28(19), Rep    -58.1(2), Dev..       4.32 Sigma
              N(1)-C(15-C(19-N(8)  1_555  1_555  1_555  1_555      #   59 Check
```

Author Response: The structure is affected by some strain. Crystal imperfections can not be excluded either.

```
PLAT703_ALERT_1_A Torsion Calc  179.25(14), Rep    179.9(2), Dev..       4.64 Sigma
              C(14-C(16-C(21-O(5)  1_555  1_555  1_555  1_555      #   60 Check
```

Author Response: The structure is affected by some strain. Crystal imperfections can not be excluded either.

```
PLAT703_ALERT_1_A Torsion Calc     -1.1(2), Rep     -2.2(4), Dev..       5.50 Sigma
              O(1)-C(17-C(21-O(5)  1_555  1_555  1_555  1_555      #   62 Check
```

Author Response: The structure is affected by some strain. Crystal imperfections can not be excluded either.

```
PLAT703_ALERT_1_A Torsion Calc     -0.8(3), Rep      0.7(5), Dev..       5.00 Sigma
              C(11-C(20-C(24-C(22  1_555  1_555  1_555  1_555      #   67 Check
```

Author Response: The structure is affected by some strain. Crystal imperfections can not be excluded either.

```
PLAT703_ALERT_1_A Torsion Calc      1.1(3), Rep     -0.8(5), Dev..       6.33 Sigma
              C(12-C(22-C(24-C(20  1_555  1_555  1_555  1_555      #   68 Check
```

Author Response: The structure is affected by some strain. Crystal imperfections can not be excluded either.

```
PLAT703_ALERT_1_A Torsion Calc    -62.8(2), Rep    -61.5(4), Dev..       6.50 Sigma
           O(5)-C(25-C(26-O(1)  1_555  1_555  1_555  1_555      #   69 Check
```

Author Response: The structure is affected by some strain. Crystal imperfections can not be excluded either.

Alert level B

```
REFLT02_ALERT_1_B  The number of reflections greater than the sigma threshold
            cannot exceed the number of symmetry-independent reflections
            Number of symmetry-independent reflections =   4020
            Number of reflections greater than sigma threshold =  26276
PLAT230_ALERT_2_B Hirshfeld Test Diff for    S1       --O4       .        8.2 s.u.
PLAT230_ALERT_2_B Hirshfeld Test Diff for    N8       --C23      .        7.3 s.u.
PLAT703_ALERT_1_B Torsion Calc  129.12(15), Rep    128.7(2), Dev..       2.80 Sigma
           O(3)-S(1)-C(13-C(12  1_555  1_555  1_555  1_555      #    4 Check
```

Author Response: The structure is affected by some strain. Crystal imperfections can not be excluded either.

```
PLAT703_ALERT_1_B Torsion Calc -102.25(16), Rep   -102.6(2), Dev..       2.19 Sigma
           O(4)-S(1)-C(13-C(12  1_555  1_555  1_555  1_555      #    8 Check
```

Author Response: The structure is affected by some strain. Crystal imperfections can not be excluded either.

```
PLAT703_ALERT_1_B Torsion Calc   47.33(18), Rep     47.7(3), Dev..       2.06 Sigma
           C(17-O(1)-C(26-C(25  1_555  1_555  1_555  1_555      #   13 Check
```

Author Response: The structure is affected by some strain. Crystal imperfections can not be excluded either.

```
PLAT703_ALERT_1_B Torsion Calc   62.70(17), Rep     63.2(2), Dev..       2.94 Sigma
           C(18-N(8)-C(19-C(15  1_555  1_555  1_555  1_555      #   25 Check
```

Author Response: The structure is affected by some strain. Crystal imperfections can not be excluded either.

```
PLAT703_ALERT_1_B Torsion Calc  -83.02(16), Rep    -83.5(2), Dev..       3.00 Sigma
           C(19-N(8)-C(18-C(14  1_555  1_555  1_555  1_555      #   27 Check
```

Author Response: The structure is affected by some strain. Crystal imperfections can not be excluded either.

```
PLAT703_ALERT_1_B Torsion Calc     -1.6(2), Rep     -2.1(4), Dev..       2.50 Sigma
            C(10-C(9)-C(14-C(16  1_555  1_555  1_555  1_555      #   34 Check
```

Author Response: The structure is affected by some strain. Crystal imperfections can not be excluded either.

```
PLAT703_ALERT_1_B Torsion Calc     -0.2(3), Rep     -1.1(4), Dev..       3.00 Sigma
            C(13-C(11-C(20-C(24  1_555  1_555  1_555  1_555      #   40 Check
```

Author Response: The structure is affected by some strain. Crystal imperfections can not be excluded either.

```
PLAT703_ALERT_1_B Torsion Calc -175.84(14), Rep   -175.5(2), Dev..       2.43 Sigma
            C(20-C(11-C(13-S(1)  1_555  1_555  1_555  1_555      #   41 Check
```

Author Response: The structure is affected by some strain. Crystal imperfections can not be excluded either.

```
PLAT703_ALERT_1_B Torsion Calc     -0.8(3), Rep     -1.6(4), Dev..       2.67 Sigma
            C(22-C(12-C(13-C(11  1_555  1_555  1_555  1_555      #   49 Check
```

Author Response: The structure is affected by some strain. Crystal imperfections can not be excluded either.

```
PLAT703_ALERT_1_B Torsion Calc   97.53(19), Rep     98.1(3), Dev..       3.00 Sigma
            C(22-C(12-C(18-N(8)  1_555  1_555  1_555  1_555      #   51 Check
```

Author Response: The structure is affected by some strain. Crystal imperfections can not be excluded either.

```
PLAT703_ALERT_1_B Torsion Calc -131.14(18), Rep   -130.6(3), Dev..       3.00 Sigma
            C(22-C(12-C(18-C(14  1_555  1_555  1_555  1_555      #   52 Check
```

Author Response: The structure is affected by some strain. Crystal imperfections can not be excluded either.

```
PLAT703_ALERT_1_B Torsion Calc     -1.5(2), Rep     -1.0(4), Dev..       2.50 Sigma
            C(10-C(17-C(21-C(16  1_555  1_555  1_555  1_555      #   65 Check
```

Author Response: The structure is affected by some strain. Crystal imperfections can not be excluded either.

```
PLAT703_ALERT_1_B Torsion Calc -179.77(16), Rep   -179.3(2), Dev..       2.94 Sigma
              CL(1-C(20-C(24-C(22  1_555  1_555  1_555  1_555      #   66 Check
```

Author Response: The structure is affected by some strain. Crystal imperfections can not be excluded either.

Alert level C

```
DIFMN03_ALERT_1_C  The minimum difference density is < -0.1*ZMAX*0.75
            The relevant atom site should be identified.
DIFMX02_ALERT_1_C  The maximum difference density is > 0.1*ZMAX*0.75
            The relevant atom site should be identified.
GOODF01_ALERT_2_C  The least squares goodness of fit parameter lies
            outside the range 0.80 <> 2.00
            Goodness of fit given =      0.613
PLAT166_ALERT_4_C S.U's Given on Coordinates for Calc-flagged ....          H1 Note
PLAT166_ALERT_4_C S.U's Given on Coordinates for Calc-flagged ....          H2 Note
PLAT166_ALERT_4_C S.U's Given on Coordinates for Calc-flagged ....          H3 Note
PLAT166_ALERT_4_C S.U's Given on Coordinates for Calc-flagged ....          H4 Note
PLAT166_ALERT_4_C S.U's Given on Coordinates for Calc-flagged ....          H5 Note
PLAT166_ALERT_4_C S.U's Given on Coordinates for Calc-flagged ....          H6 Note
PLAT166_ALERT_4_C S.U's Given on Coordinates for Calc-flagged ....          H7 Note
PLAT166_ALERT_4_C S.U's Given on Coordinates for Calc-flagged ....          H8 Note
PLAT166_ALERT_4_C S.U's Given on Coordinates for Calc-flagged ....          H9 Note
PLAT166_ALERT_4_C S.U's Given on Coordinates for Calc-flagged ....         H10 Note
PLAT166_ALERT_4_C S.U's Given on Coordinates for Calc-flagged ....         H11 Note
PLAT166_ALERT_4_C S.U's Given on Coordinates for Calc-flagged ....         H12 Note
PLAT166_ALERT_4_C S.U's Given on Coordinates for Calc-flagged ....         H13 Note
PLAT166_ALERT_4_C S.U's Given on Coordinates for Calc-flagged ....         H14 Note
PLAT166_ALERT_4_C S.U's Given on Coordinates for Calc-flagged ....         H15 Note
PLAT230_ALERT_2_C Hirshfeld Test Diff for    S1       --N1       .         6.3 s.u.
PLAT230_ALERT_2_C Hirshfeld Test Diff for    O1       --C26      .         5.3 s.u.
PLAT230_ALERT_2_C Hirshfeld Test Diff for    O5       --C25      .         6.1 s.u.
PLAT230_ALERT_2_C Hirshfeld Test Diff for    C15      --C19      .         5.1 s.u.
PLAT245_ALERT_2_C U(iso) H6        Smaller than U(eq) C23       by      0.017 Ang**2
PLAT245_ALERT_2_C U(iso) H9        Smaller than U(eq) C19       by      0.012 Ang**2
PLAT245_ALERT_2_C U(iso) H10       Smaller than U(eq) C15       by      0.016 Ang**2
PLAT245_ALERT_2_C U(iso) H12       Smaller than U(eq) C16       by      0.014 Ang**2
PLAT245_ALERT_2_C U(iso) H13       Smaller than U(eq) C10       by      0.013 Ang**2
PLAT351_ALERT_3_C Long   C-H (X0.96,N1.08A)  C23      - H5       .        1.13 Ang.
PLAT703_ALERT_1_C Torsion Calc  -54.08(14), Rep    -54.3(2), Dev..       1.57 Sigma
              O(3)-S(1)-C(13-C(11  1_555  1_555  1_555  1_555      #    3 Check
```

Author Response: The structure is affected by some strain. Crystal imperfections can not be excluded either.

```
PLAT703_ALERT_1_C Torsion Calc -170.04(13), Rep   -169.9(2), Dev..       1.08 Sigma
              N(1)-S(1)-C(13-C(11  1_555  1_555  1_555  1_555      #    9 Check
```

Author Response: The structure is affected by some strain. Crystal imperfections can not be excluded either.

```
PLAT703_ALERT_1_C Torsion Calc  -68.47(11), Rep  -68.32(18), Dev..       1.36 Sigma
              C(13-S(1)-N(1)-C(9)  1_555  1_555  1_555  1_555      #   11 Check
```

Author Response: The structure is affected by some strain. Crystal imperfections can not be excluded either.

```
PLAT703_ALERT_1_C Torsion Calc   78.81(18), Rep     79.1(2), Dev..       1.61 Sigma
              C(9)-N(1)-C(15-C(19  1_555  1_555  1_555  1_555      #   22 Check
```

Author Response: The structure is affected by some strain. Crystal imperfections can not be excluded either.

```
PLAT703_ALERT_1_C Torsion Calc  -65.78(16), Rep    -65.6(3), Dev..       1.13 Sigma
              C(15-N(1)-C(9)-C(14  1_555  1_555  1_555  1_555      #   24 Check
```

Author Response: The structure is affected by some strain. Crystal imperfections can not be excluded either.

```
PLAT703_ALERT_1_C Torsion Calc -169.78(14), Rep   -169.5(2), Dev..       2.00 Sigma
              C(23-N(8)-C(19-C(15  1_555  1_555  1_555  1_555      #   30 Check
```

Author Response: The structure is affected by some strain. Crystal imperfections can not be excluded either.

```
PLAT703_ALERT_1_C Torsion Calc -179.74(13), Rep   -179.5(2), Dev..       1.85 Sigma
              N(1)-C(9)-C(10-C(17  1_555  1_555  1_555  1_555      #   31 Check
```

Author Response: The structure is affected by some strain. Crystal imperfections can not be excluded either.

```
PLAT703_ALERT_1_C Torsion Calc  178.60(13), Rep    178.8(2), Dev..       1.54 Sigma
              N(1)-C(9)-C(14-C(16  1_555  1_555  1_555  1_555      #   32 Check
```

Author Response: The structure is affected by some strain. Crystal imperfections can not be excluded either.

```
PLAT703_ALERT_1_C Torsion Calc    0.00(19), Rep      0.3(3), Dev..       1.58 Sigma
              N(1)-C(9)-C(14-C(18  1_555  1_555  1_555  1_555      #   33 Check
```

Author Response: The structure is affected by some strain. Crystal imperfections can not be excluded either.

```
PLAT703_ALERT_1_C Torsion Calc -178.14(13), Rep   -178.4(2), Dev..       2.00 Sigma
              C(9)-C(10-C(17-O(1)  1_555  1_555  1_555  1_555      #   37 Check
```

Author Response: The structure is affected by some strain. Crystal imperfections can not be excluded either.

```
PLAT703_ALERT_1_C Torsion Calc  178.70(14), Rep    178.9(2), Dev..       1.43 Sigma
              C(13-C(11-C(20-CL(1  1_555  1_555  1_555  1_555      #   39 Check
```

Author Response: The structure is affected by some strain. Crystal imperfections can not be excluded either.

```
PLAT703_ALERT_1_C Torsion Calc      1.1(3), Rep      1.6(4), Dev..       1.67 Sigma
              C(20-C(11-C(13-C(12  1_555  1_555  1_555  1_555      #   42 Check
```

Author Response: The structure is affected by some strain. Crystal imperfections can not be excluded either.

```
PLAT703_ALERT_1_C Torsion Calc  -78.02(19), Rep    -78.3(3), Dev..       1.47 Sigma
              C(13-C(12-C(18-N(8)  1_555  1_555  1_555  1_555      #   43 Check
```

Author Response: The structure is affected by some strain. Crystal imperfections can not be excluded either.

```
PLAT703_ALERT_1_C Torsion Calc     53.3(2), Rep     53.0(4), Dev..       1.50 Sigma
              C(13-C(12-C(18-C(14  1_555  1_555  1_555  1_555      #   44 Check
```

Author Response: The structure is affected by some strain. Crystal imperfections can not be excluded either.

```
PLAT703_ALERT_1_C Torsion Calc  177.70(14), Rep    177.5(2), Dev..       1.43 Sigma
              O(1)-C(17-C(21-C(16  1_555  1_555  1_555  1_555      #   63 Check
```

Author Response: The structure is affected by some strain. Crystal imperfections can not be excluded either.

```
PLAT703_ALERT_1_C Torsion Calc  179.65(14), Rep    179.4(2), Dev..       1.79 Sigma
              C(10-C(17-C(21-O(5)  1_555  1_555  1_555  1_555      #   64 Check
```

Author Response: The structure is affected by some strain. Crystal imperfections can not be excluded either.

Alert level G

```
PLAT005_ALERT_5_G No Embedded Refinement Details Found  in the CIF     Please Do !
PLAT128_ALERT_4_G Alternate Setting for Input Space Group     C2/c       I2/a Note
PLAT199_ALERT_1_G Reported _cell_measurement_temperature ..... (K)        293 Check
PLAT200_ALERT_1_G Reported   _diffrn_ambient_temperature ..... (K)        293 Check
```

```
PLAT793_ALERT_4_G Model has Chirality at C18         (Centro SpGr)          S Verify
PLAT808_ALERT_5_G No Parseable SHELXL Style Weighting Scheme Found     Please Check
PLAT882_ALERT_1_G No Datum for _diffrn_reflns_av_unetI/netI ......     Please Do !
PLAT883_ALERT_1_G Absent Datum for _atom_sites_solution_primary ..     Please Do !
```

```
  30 ALERT level A = Most likely a serious problem - resolve or explain
  16 ALERT level B = A potentially serious problem, consider carefully
  44 ALERT level C = Check. Ensure it is not caused by an omission or oversight
   8 ALERT level G = General information/check it is not something unexpected

  63 ALERT type 1 CIF construction/syntax error, inconsistent or missing data
  15 ALERT type 2 Indicator that the structure model may be wrong or deficient
   1 ALERT type 3 Indicator that the structure quality may be low
  17 ALERT type 4 Improvement, methodology, query or suggestion
   2 ALERT type 5 Informative message, check
```

It is advisable to attempt to resolve as many as possible of the alerts in all categories. Often the minor alerts point to easily fixed oversights, errors and omissions in your CIF or refinement strategy, so attention to these fine details can be worthwhile. In order to resolve some of the more serious problems it may be necessary to carry out additional measurements or structure refinements. However, the purpose of your study may justify the reported deviations and the more serious of these should normally be commented upon in the discussion or experimental section of a paper or in the "special_details" fields of the CIF. checkCIF was carefully designed to identify outliers and unusual parameters, but every test has its limitations and alerts that are not important in a particular case may appear. Conversely, the absence of alerts does not guarantee there are no aspects of the results needing attention. It is up to the individual to critically assess their own results and, if necessary, seek expert advice.

Publication of your CIF in IUCr journals

A basic structural check has been run on your CIF. These basic checks will be run on all CIFs submitted for publication in IUCr journals (*Acta Crystallographica*, *Journal of Applied Crystallography*, *Journal of Synchrotron Radiation*); however, if you intend to submit to *Acta Crystallographica Section C* or *E* or *IUCrData*, you should make sure that full publication checks are run on the final version of your CIF prior to submission.

Publication of your CIF in other journals

Please refer to the *Notes for Authors* of the relevant journal for any special instructions relating to CIF submission.

Validation response form

Please find below a validation response form (VRF) that can be filled in and pasted into your CIF.

```
# start Validation Reply Form
_vrf_REFLT02__85623_1
;
PROBLEM: The number of reflections greater than the sigma threshold
```

```
RESPONSE: ...
;
_vrf_DIFMN03__85623_1
;
PROBLEM: The minimum difference density is < -0.1*ZMAX*0.75
RESPONSE: ...
;
_vrf_DIFMX02__85623_1
;
PROBLEM: The maximum difference density is > 0.1*ZMAX*0.75
RESPONSE: ...
;
_vrf_GOODF01__85623_1
;
PROBLEM: The least squares goodness of fit parameter lies
RESPONSE: ...
;
_vrf_PLAT230__85623_1
;
PROBLEM: Hirshfeld Test Diff for    S1       --O4       .        8.2 s.u.
RESPONSE: ...
;
_vrf_PLAT166__85623_1
;
PROBLEM: S.U's Given on Coordinates for Calc-flagged ....         H1 Note
RESPONSE: ...
;
_vrf_PLAT245__85623_1
;
PROBLEM: U(iso) H6        Smaller than U(eq) C23       by      0.017 Ang**2
RESPONSE: ...
;
_vrf_PLAT351__85623_1
;
PROBLEM: Long   C-H (X0.96,N1.08A)  C23      - H5       .       1.13 Ang.
RESPONSE: ...
;
# end Validation Reply Form
```

PLATON version of 04/06/2025; check.def file version of 30/05/2025

Datablock _85623_1 - ellipsoid plot

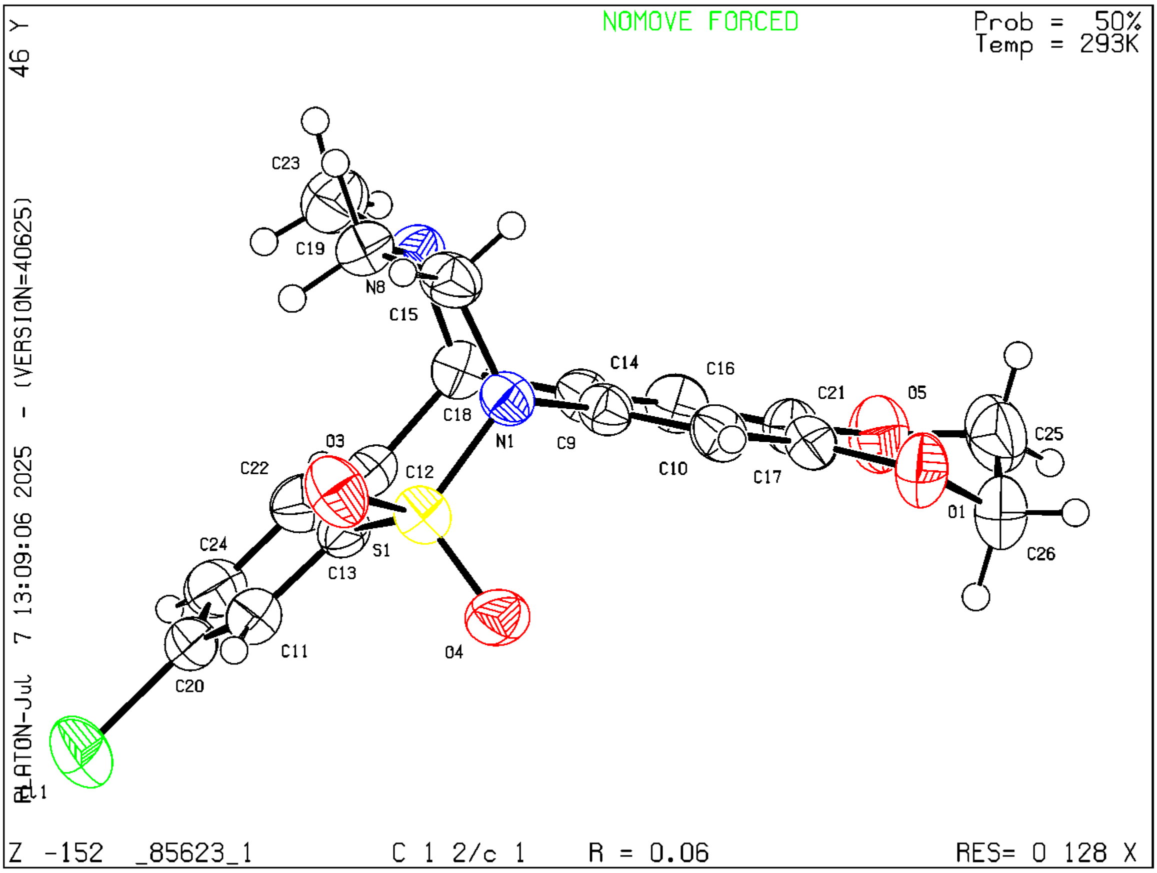

Supplement: File 2 — Crystallographic information files, checkcif and structure report files for compounds 20e, 21g, 23a, 25–27. [file compound 25 checkcif.pdf › compound 25 checkcif.pdf]
