# Supplementary material for: Synthesis of new tetra- and pentacyclic, methylenedioxy- and ethylenedioxy-substituted derivatives of the dibenzo[c,f][1,2]thiazepine ring system
Source: Beilstein J Org Chem. 2025 Dec 9;21:2645–56. doi: 10.3762/bjoc.21.205 (PMC12706374; doi:10.3762/bjoc.21.205)

checkCIF/PLATON report

You have not supplied any structure factors. As a result the full set of tests cannot be run.

THIS REPORT IS FOR GUIDANCE ONLY. IF USED AS PART OF A REVIEW PROCEDURE FOR PUBLICATION, IT SHOULD NOT REPLACE THE EXPERTISE OF AN EXPERIENCED CRYSTALLOGRAPHIC REFEREE.

No syntax errors found. CIF dictionary Interpreting this report

Datablock: _143527_1

```
Bond precision:   C-C = 0.0029 A          Wavelength=1.54187

Cell:              a=8.8017(4)     b=15.2880(6)       c=12.7533(5)
                   alpha=90        beta=97.430(2)     gamma=90
Temperature:       293 K
```

	Calculated	Reported
Volume	1701.68(12)	1701.68(12)
Space group	P 21/a	P 1 21/a 1
Hall group	-P 2yab	-P 2yab
Moiety formula	C17 H14 Cl N O4 S2	C17 H14 Cl N O4 S2
Sum formula	C17 H14 Cl N O4 S2	C17 H14 Cl N O4 S2
Mr	395.86	395.87
Dx,g cm-3	1.545	1.545
Z	4	4
Mu (mm-1)	4.491	4.491
F000	816.0	816.0
F000’	822.22	
h,k,lmax	10,18,15	10,18,15
Nref	3328	3204
Tmin,Tmax	0.521,0.638	0.353,0.638
Tmin’	0.151	

```
Correction method= # Reported T Limits: Tmin=0.353 Tmax=0.638
AbsCorr = MULTI-SCAN

Data completeness= 0.963          Theta(max)= 71.700

R(reflections)= 0.0427( 11055)          wR2(reflections)= 0.0642( 11129)

S = 1.162                  Npar= 267
```

The following ALERTS were generated. Each ALERT has the format
test-name_ALERT_alert-type_alert-level.
Click on the hyperlinks for more details of the test.

Alert level A

```
PLAT097_ALERT_2_A Large Reported Max.  (Positive) Residual Density       4.89 eA-3
```

Author Response: Altough the residual density is out of border but there is no doubt about the chemical structure taking into account other evidences, e.g. NMR. The error maybe due to crystal imperfections.

Alert level B

```
DIFMN02_ALERT_2_B  The minimum difference density is < -0.1*ZMAX*1.00
            _refine_diff_density_min given =     -3.400
            Test value =     -1.700
REFLT02_ALERT_1_B  The number of reflections greater than the sigma threshold
            cannot exceed the number of symmetry-independent reflections
            Number of symmetry-independent reflections =   3204
            Number of reflections greater than sigma threshold =  11055
PLAT098_ALERT_2_B Large Reported Min.  (Negative) Residual Density      -3.40 eA-3
PLAT213_ALERT_2_B Atom C22              has ADP max/min Ratio .....       4.6 prolat
PLAT230_ALERT_2_B Hirshfeld Test Diff for    S1       --O1       .        8.8 s.u.
PLAT230_ALERT_2_B Hirshfeld Test Diff for    S1       --N1       .       10.5 s.u.
PLAT241_ALERT_2_B High   'MainMol' Ueq as Compared to Neighbors of        C22 Check
PLAT242_ALERT_2_B Low    'MainMol' Ueq as Compared to Neighbors of         O5 Check
PLAT360_ALERT_2_B Short  C(sp3)-C(sp3) Bond  C22       - C25       .       1.32 Ang.
PLAT703_ALERT_1_B Torsion Calc  -50.01(14), Rep  -50.40(19), Dev..       2.79 Sigma
              O(7)-S(1)-C(15-C(9)  1_555  1_555  1_555  1_555      #    7 Check
PLAT703_ALERT_1_B Torsion Calc   55.83(15), Rep     56.2(2), Dev..       2.47 Sigma
              C(24-S(3)-C(14-C(19  1_555  1_555  1_555  1_555      #   15 Check
PLAT703_ALERT_1_B Torsion Calc     85.4(2), Rep     86.0(3), Dev..       3.00 Sigma
              C(12-N(1)-C(23-C(24  1_555  1_555  1_555  1_555      #   25 Check
PLAT703_ALERT_1_B Torsion Calc    100.4(2), Rep     99.9(2), Dev..       2.50 Sigma
              C(23-N(1)-C(12-C(11  1_555  1_555  1_555  1_555      #   26 Check
PLAT703_ALERT_1_B Torsion Calc  176.68(17), Rep    177.1(2), Dev..       2.47 Sigma
              C(13-C(10-C(16-C(14  1_555  1_555  1_555  1_555      #   33 Check
PLAT703_ALERT_1_B Torsion Calc -179.45(16), Rep   -179.8(2), Dev..       2.19 Sigma
              C(12-C(11-C(20-O(4)  1_555  1_555  1_555  1_555      #   36 Check
PLAT703_ALERT_1_B Torsion Calc       5.1(3), Rep      4.4(3), Dev..       2.33 Sigma
              N(1)-C(12-C(16-C(14  1_555  1_555  1_555  1_555      #   41 Check
PLAT703_ALERT_1_B Torsion Calc -174.66(17), Rep   -175.1(2), Dev..       2.59 Sigma
              C(11-C(12-C(16-C(14  1_555  1_555  1_555  1_555      #   43 Check
PLAT703_ALERT_1_B Torsion Calc     66.3(2), Rep     66.9(2), Dev..       3.00 Sigma
              S(3)-C(14-C(16-C(12  1_555  1_555  1_555  1_555      #   49 Check
PLAT703_ALERT_1_B Torsion Calc  118.49(19), Rep    118.1(2), Dev..       2.05 Sigma
              C(19-C(14-C(16-C(10  1_555  1_555  1_555  1_555      #   54 Check
PLAT703_ALERT_1_B Torsion Calc    -65.3(2), Rep    -64.8(3), Dev..       2.50 Sigma
              C(19-C(14-C(16-C(12  1_555  1_555  1_555  1_555      #   55 Check
```

Alert level C

```
DIFMN03_ALERT_1_C  The minimum difference density is < -0.1*ZMAX*0.75
            The relevant atom site should be identified.
DIFMX02_ALERT_1_C  The maximum difference density is > 0.1*ZMAX*0.75
            The relevant atom site should be identified.
PLAT029_ALERT_3_C _diffrn_measured_fraction_theta_full value Low .      0.962 Why?
PLAT166_ALERT_4_C S.U's Given on Coordinates for Calc-flagged ....         H1 Note
PLAT166_ALERT_4_C S.U's Given on Coordinates for Calc-flagged ....         H2 Note
PLAT166_ALERT_4_C S.U's Given on Coordinates for Calc-flagged ....         H3 Note
PLAT166_ALERT_4_C S.U's Given on Coordinates for Calc-flagged ....         H4 Note
PLAT166_ALERT_4_C S.U's Given on Coordinates for Calc-flagged ....         H5 Note
PLAT166_ALERT_4_C S.U's Given on Coordinates for Calc-flagged ....         H6 Note
PLAT166_ALERT_4_C S.U's Given on Coordinates for Calc-flagged ....         H7 Note
PLAT166_ALERT_4_C S.U's Given on Coordinates for Calc-flagged ....         H8 Note
PLAT166_ALERT_4_C S.U's Given on Coordinates for Calc-flagged ....         H9 Note
PLAT220_ALERT_2_C NonSolvent   Resd 1  C   Ueq(max)/Ueq(min) Range        6.0 Ratio
PLAT222_ALERT_3_C NonSolvent Resd 1  H   Uiso(max)/Uiso(min) Range       10.0 Ratio
PLAT230_ALERT_2_C Hirshfeld Test Diff for    Cl2      --C18      .        5.8 s.u.
PLAT230_ALERT_2_C Hirshfeld Test Diff for    S3       --C14      .        5.6 s.u.
PLAT230_ALERT_2_C Hirshfeld Test Diff for    C17      --C21      .        5.5 s.u.
PLAT242_ALERT_2_C Low    'MainMol' Ueq as Compared to Neighbors of        C25 Check
PLAT245_ALERT_2_C U(iso) H4        Smaller than U(eq) C11       by      0.021 Ang**2
PLAT351_ALERT_3_C Long   C-H (X0.96,N1.08A)  C14      - H10      .       1.14 Ang.
PLAT351_ALERT_3_C Long   C-H (X0.96,N1.08A)  C23      - H8       .       1.11 Ang.
PLAT410_ALERT_2_C Short Intra H...H Contact  H11      ..H14      .       1.91 Ang.
                                                      x,y,z  =      1_555 Check
PLAT410_ALERT_2_C Short Intra H...H Contact  H12      ..H13      .       1.91 Ang.
                                                      x,y,z  =      1_555 Check
PLAT703_ALERT_1_C Torsion Calc -161.66(14), Rep -161.48(19), Dev..       1.29 Sigma
              O(1)-S(1)-N(1)-C(23  1_555  1_555  1_555  1_555      #    2 Check
PLAT703_ALERT_1_C Torsion Calc -103.18(15), Rep   -102.9(2), Dev..       1.87 Sigma
              O(1)-S(1)-C(15-C(19  1_555  1_555  1_555  1_555      #    4 Check
PLAT703_ALERT_1_C Torsion Calc  174.83(11), Rep  174.61(15), Dev..       2.00 Sigma
              O(7)-S(1)-N(1)-C(12  1_555  1_555  1_555  1_555      #    5 Check
PLAT703_ALERT_1_C Torsion Calc   13.04(17), Rep     13.3(2), Dev..       1.53 Sigma
              N(1)-S(1)-C(15-C(19  1_555  1_555  1_555  1_555      #   10 Check
PLAT703_ALERT_1_C Torsion Calc  -70.53(13), Rep  -70.67(18), Dev..       1.08 Sigma
              C(15-S(1)-N(1)-C(12  1_555  1_555  1_555  1_555      #   11 Check
PLAT703_ALERT_1_C Torsion Calc    -12.1(3), Rep    -12.5(4), Dev..       1.33 Sigma
              C(20-O(4)-C(25-C(22  1_555  1_555  1_555  1_555      #   16 Check
PLAT703_ALERT_1_C Torsion Calc -179.58(16), Rep   -179.4(2), Dev..       1.13 Sigma
              C(25-O(4)-C(20-C(11  1_555  1_555  1_555  1_555      #   17 Check
PLAT703_ALERT_1_C Torsion Calc   74.32(19), Rep     74.6(2), Dev..       1.47 Sigma
              S(1)-N(1)-C(12-C(16  1_555  1_555  1_555  1_555      #   23 Check
PLAT703_ALERT_1_C Torsion Calc    -79.3(2), Rep    -79.6(3), Dev..       1.50 Sigma
              C(23-N(1)-C(12-C(16  1_555  1_555  1_555  1_555      #   27 Check
PLAT703_ALERT_1_C Torsion Calc -179.13(14), Rep -178.91(18), Dev..       1.57 Sigma
              C(15-C(9)-C(18-CL(2  1_555  1_555  1_555  1_555      #   28 Check
PLAT703_ALERT_1_C Torsion Calc     -0.9(3), Rep     -0.4(3), Dev..       1.67 Sigma
              C(15-C(9)-C(18-C(21  1_555  1_555  1_555  1_555      #   29 Check
PLAT703_ALERT_1_C Torsion Calc      1.0(3), Rep      0.5(3), Dev..       1.67 Sigma
              C(18-C(9)-C(15-C(19  1_555  1_555  1_555  1_555      #   31 Check
PLAT703_ALERT_1_C Torsion Calc      0.4(3), Rep     -0.1(3), Dev..       1.67 Sigma
              C(13-C(10-C(16-C(12  1_555  1_555  1_555  1_555      #   32 Check
PLAT703_ALERT_1_C Torsion Calc  177.08(16), Rep    177.3(2), Dev..       1.38 Sigma
              C(16-C(10-C(13-O(5)  1_555  1_555  1_555  1_555      #   34 Check
PLAT703_ALERT_1_C Torsion Calc     -2.3(3), Rep     -1.9(4), Dev..       1.33 Sigma
              C(16-C(10-C(13-C(20  1_555  1_555  1_555  1_555      #   35 Check
```

```
PLAT703_ALERT_1_C Torsion Calc -178.68(16), Rep   -178.4(2), Dev..       1.75 Sigma
              N(1)-C(12-C(16-C(10  1_555  1_555  1_555  1_555      #   40 Check
PLAT703_ALERT_1_C Torsion Calc      1.6(3), Rep      2.0(4), Dev..       1.33 Sigma
              C(11-C(12-C(16-C(10  1_555  1_555  1_555  1_555      #   42 Check
PLAT703_ALERT_1_C Torsion Calc      2.0(3), Rep      2.4(4), Dev..       1.33 Sigma
              O(5)-C(13-C(20-O(4)  1_555  1_555  1_555  1_555      #   44 Check
PLAT703_ALERT_1_C Torsion Calc -178.65(16), Rep   -178.4(2), Dev..       1.56 Sigma
              C(10-C(13-C(20-O(4)  1_555  1_555  1_555  1_555      #   46 Check
PLAT703_ALERT_1_C Torsion Calc -109.95(16), Rep   -110.2(2), Dev..       1.56 Sigma
              S(3)-C(14-C(16-C(10  1_555  1_555  1_555  1_555      #   48 Check
PLAT703_ALERT_1_C Torsion Calc -178.52(13), Rep -178.74(18), Dev..       1.69 Sigma
              S(1)-C(15-C(19-C(17  1_555  1_555  1_555  1_555      #   57 Check
PLAT703_ALERT_1_C Torsion Calc  178.65(16), Rep    178.9(2), Dev..       1.56 Sigma
              C(9)-C(15-C(19-C(14  1_555  1_555  1_555  1_555      #   58 Check
```

Alert level G

```
PLAT005_ALERT_5_G No Embedded Refinement Details Found  in the CIF     Please Do !
PLAT128_ALERT_4_G Alternate Setting for Input Space Group  P21/a        P21/c Note
PLAT199_ALERT_1_G Reported _cell_measurement_temperature ..... (K)        293 Check
PLAT200_ALERT_1_G Reported   _diffrn_ambient_temperature ..... (K)        293 Check
PLAT793_ALERT_4_G Model has Chirality at C14         (Centro SpGr)          S Verify
PLAT808_ALERT_5_G No Parseable SHELXL Style Weighting Scheme Found     Please Check
PLAT882_ALERT_1_G No Datum for _diffrn_reflns_av_unetI/netI ......     Please Do !
PLAT883_ALERT_1_G Absent Datum for _atom_sites_solution_primary ..     Please Do !
```

---

1 **ALERT level A** = Most likely a serious problem - resolve or explain
20 **ALERT level B** = A potentially serious problem, consider carefully
45 **ALERT level C** = Check. Ensure it is not caused by an omission or oversight
8 **ALERT level G** = General information/check it is not something unexpected

40 ALERT type 1 CIF construction/syntax error, inconsistent or missing data
17 ALERT type 2 Indicator that the structure model may be wrong or deficient
4 ALERT type 3 Indicator that the structure quality may be low
11 ALERT type 4 Improvement, methodology, query or suggestion
2 ALERT type 5 Informative message, check

It is advisable to attempt to resolve as many as possible of the alerts in all categories. Often the minor alerts point to easily fixed oversights, errors and omissions in your CIF or refinement strategy, so attention to these fine details can be worthwhile. In order to resolve some of the more serious problems it may be necessary to carry out additional measurements or structure refinements. However, the purpose of your study may justify the reported deviations and the more serious of these should normally be commented upon in the discussion or experimental section of a paper or in the "special_details" fields of the CIF. checkCIF was carefully designed to identify outliers and unusual parameters, but every test has its limitations and alerts that are not important in a particular case may appear. Conversely, the absence of alerts does not guarantee there are no aspects of the results needing attention. It is up to the individual to critically assess their own results and, if necessary, seek expert advice.

Publication of your CIF in IUCr journals

A basic structural check has been run on your CIF. These basic checks will be run on all CIFs submitted for publication in IUCr journals (*Acta Crystallographica*, *Journal of Applied Crystallography*, *Journal of Synchrotron Radiation*); however, if you intend to submit to *Acta Crystallographica Section C* or *E* or *IUCrData*, you should make sure that full publication checks are run on the final version of your CIF prior to submission.

Publication of your CIF in other journals

Please refer to the *Notes for Authors* of the relevant journal for any special instructions relating to CIF submission.

Validation response form

Please find below a validation response form (VRF) that can be filled in and pasted into your CIF.

```
# start Validation Reply Form
_vrf_DIFMN02__143527_1
;
PROBLEM: The minimum difference density is < -0.1*ZMAX*1.00
RESPONSE: ...
;
_vrf_REFLT02__143527_1
;
PROBLEM: The number of reflections greater than the sigma threshold
RESPONSE: ...
;
_vrf_DIFMN03__143527_1
;
PROBLEM: The minimum difference density is < -0.1*ZMAX*0.75
RESPONSE: ...
;
_vrf_DIFMX02__143527_1
;
PROBLEM: The maximum difference density is > 0.1*ZMAX*0.75
RESPONSE: ...
;
_vrf_PLAT098__143527_1
```

```
;
PROBLEM: Large Reported Min.  (Negative) Residual Density      -3.40 eA-3
RESPONSE: ...
;
_vrf_PLAT213__143527_1
;
PROBLEM: Atom C22             has ADP max/min Ratio .....         4.6 prolat
RESPONSE: ...
;
_vrf_PLAT230__143527_1
;
PROBLEM: Hirshfeld Test Diff for    S1       --O1       .         8.8 s.u.
RESPONSE: ...
;
_vrf_PLAT241__143527_1
;
PROBLEM: High   'MainMol' Ueq as Compared to Neighbors of         C22 Check
RESPONSE: ...
;
_vrf_PLAT242__143527_1
;
PROBLEM: Low    'MainMol' Ueq as Compared to Neighbors of          O5 Check
RESPONSE: ...
;
_vrf_PLAT360__143527_1
;
PROBLEM: Short  C(sp3)-C(sp3) Bond  C22      - C25      .        1.32 Ang.
RESPONSE: ...
;
_vrf_PLAT703__143527_1
;
PROBLEM: Torsion Calc  -50.01(14), Rep  -50.40(19), Dev..        2.79 Sigma
RESPONSE: ...
;
_vrf_PLAT029__143527_1
;
PROBLEM: _diffrn_measured_fraction_theta_full value Low .      0.962 Why?
RESPONSE: ...
;
_vrf_PLAT166__143527_1
;
PROBLEM: S.U's Given on Coordinates for Calc-flagged ....          H1 Note
RESPONSE: ...
;
_vrf_PLAT220__143527_1
;
PROBLEM: NonSolvent   Resd 1  C   Ueq(max)/Ueq(min) Range         6.0 Ratio
RESPONSE: ...
;
_vrf_PLAT222__143527_1
;
PROBLEM: NonSolvent Resd 1  H   Uiso(max)/Uiso(min) Range        10.0 Ratio
RESPONSE: ...
;
_vrf_PLAT245__143527_1
;
PROBLEM: U(iso) H4        Smaller than U(eq) C11       by      0.021 Ang**2
```

```
RESPONSE: ...
;
_vrf_PLAT351__143527_1
;
PROBLEM: Long   C-H (X0.96,N1.08A)  C14       - H10       .       1.14 Ang.
RESPONSE: ...
;
_vrf_PLAT410__143527_1
;
PROBLEM: Short Intra H...H Contact  H11       ..H14       .       1.91 Ang.
RESPONSE: ...
;
# end Validation Reply Form
```

PLATON version of 04/06/2025; check.def file version of 30/05/2025

Datablock _143527_1 - ellipsoid plot

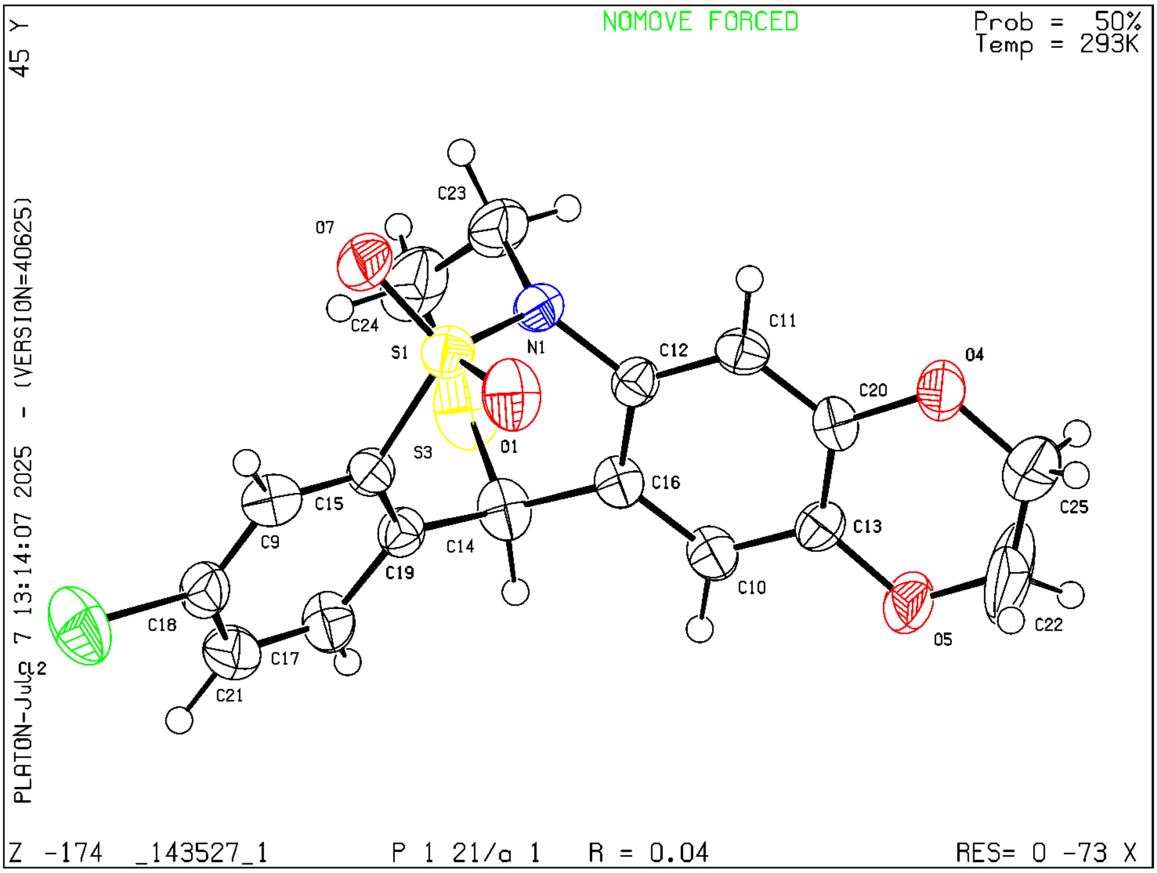

Supplement: File 2 — Crystallographic information files, checkcif and structure report files for compounds 20e, 21g, 23a, 25–27. [file compound 26 checkcif.pdf › compound 26 checkcif.pdf]
